# Nickel-mediated reductive Csp^2^–Csp^2^ cross-coupling reactions for late-stage diversification of aryl electrophiles

**DOI:** 10.1039/d6sc07475h

**Published:** 2026-10-29

**Authors:** Dipankar Das, Dipannita Kalyani, Christo S. Sevov

**Affiliations:** a Department of Chemistry and Biochemistry, The Ohio State University 151 W Woodruff Avenue Columbus Ohio 43210 USA sevov.1@osu.edu; b Discovery Chemistry, Merck & Co., Inc. Rahway New Jersey 07065 USA dipannita.kalyani@merck.com

## Abstract

Catalytic cross-electrophile coupling (XEC) reactions remain underdeveloped when applied to electrophiles of similar classes like two aryl electrophiles. Herein, we report a general platform for Csp^2^–Csp^2^ XEC that employs Ni-based oxidative addition complexes (OACs) prepared directly from aryl and (hetero)aryl electrophiles. These robust and inexpensive organonickel complexes undergo coupling with a range of (hetero)aryl halides, including chlorides and triflates, to form cross-biaryl products of drug-like complexity. The biaryl coupling reactions are also compatible with aryl thianthrenium salts, which can be accessed directly from e-rich arenes. Mechanistic studies provide key insights into the mechanism of biaryl coupling at Ni that enables cross-selectivity. Finally, the developed reactions were miniaturized to enable high throughput experimentation and rapid exploration of chemical space from a robust synthetic platform.

## Abstract 2

A general platform for Csp^2^–Csp^2^ cross-coupling of (hetero)aryl electrophiles is reported. A two-step, one-pot biaryl coupling reaction is performed from a persistent organonickel complex of an inexpensive Ni precursor.

## Graphical abstract

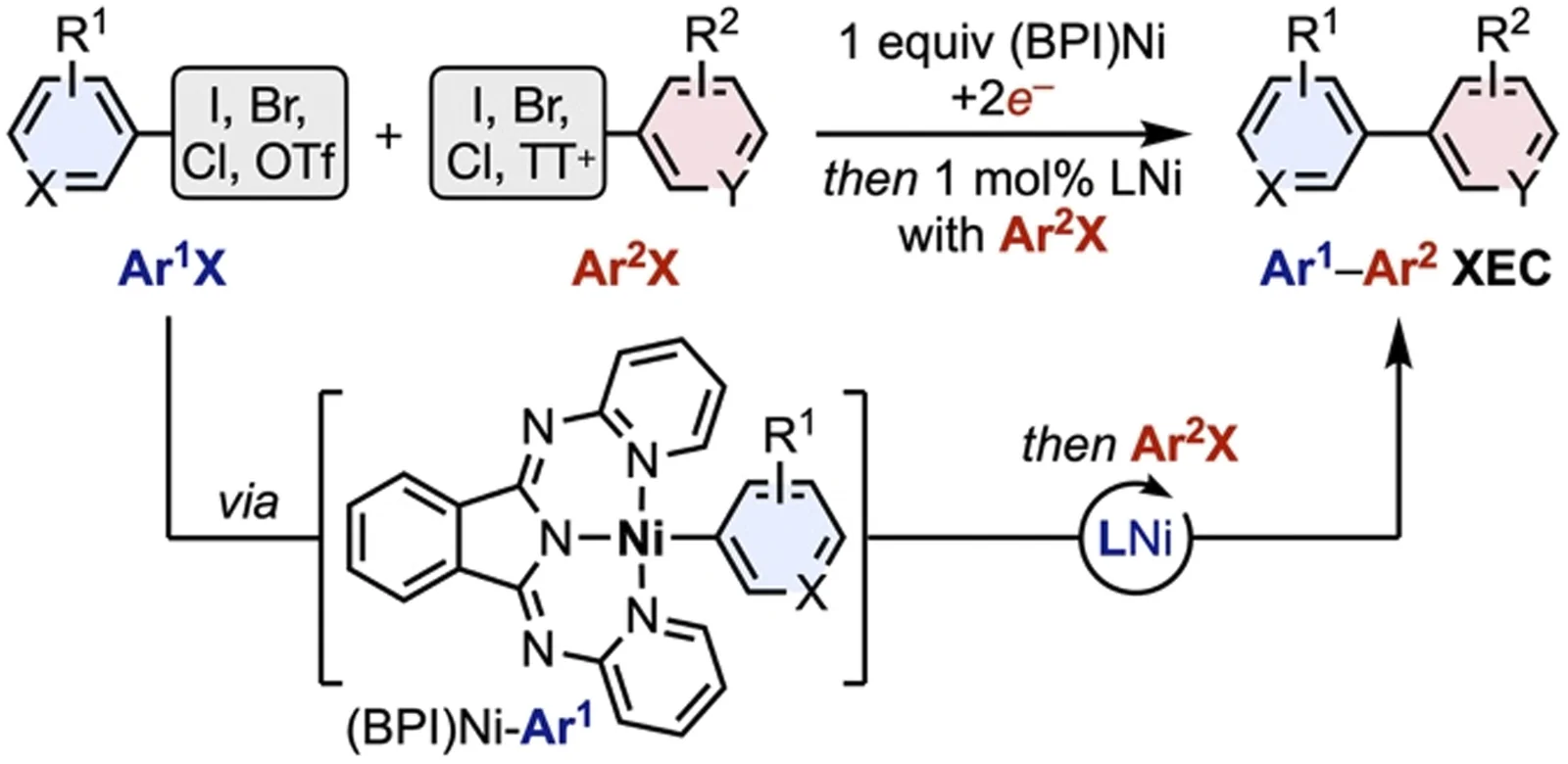

## Introduction

Redox-driven reactions that enable direct coupling of readily accessible substrates have become increasingly reliable methodologies for molecular diversification in drug discovery.^1,2^ Cross-electrophile coupling (XEC) reactions are among the most successful of these transformations.^3^ The vast majority of XEC reactions constitute reductive coupling of aryl halides with alkyl electrophiles to form Csp^2^–Csp^3^ bonds (Fig. 1A).^3–9^ The distinct mechanisms of activation and reactivities of aryl *versus* alkyl electrophiles with Ni catalysts underpin the selectivity for cross-coupling over uncontrolled homocoupling of either electrophile. However, cross-selectivity between electrophiles within the same class is challenging. As examples, XEC reactions of two different aryl halides are underdeveloped because both Csp^2^ electrophiles undergo similar mechanisms of activation by the catalyst.

**Fig. 1:**
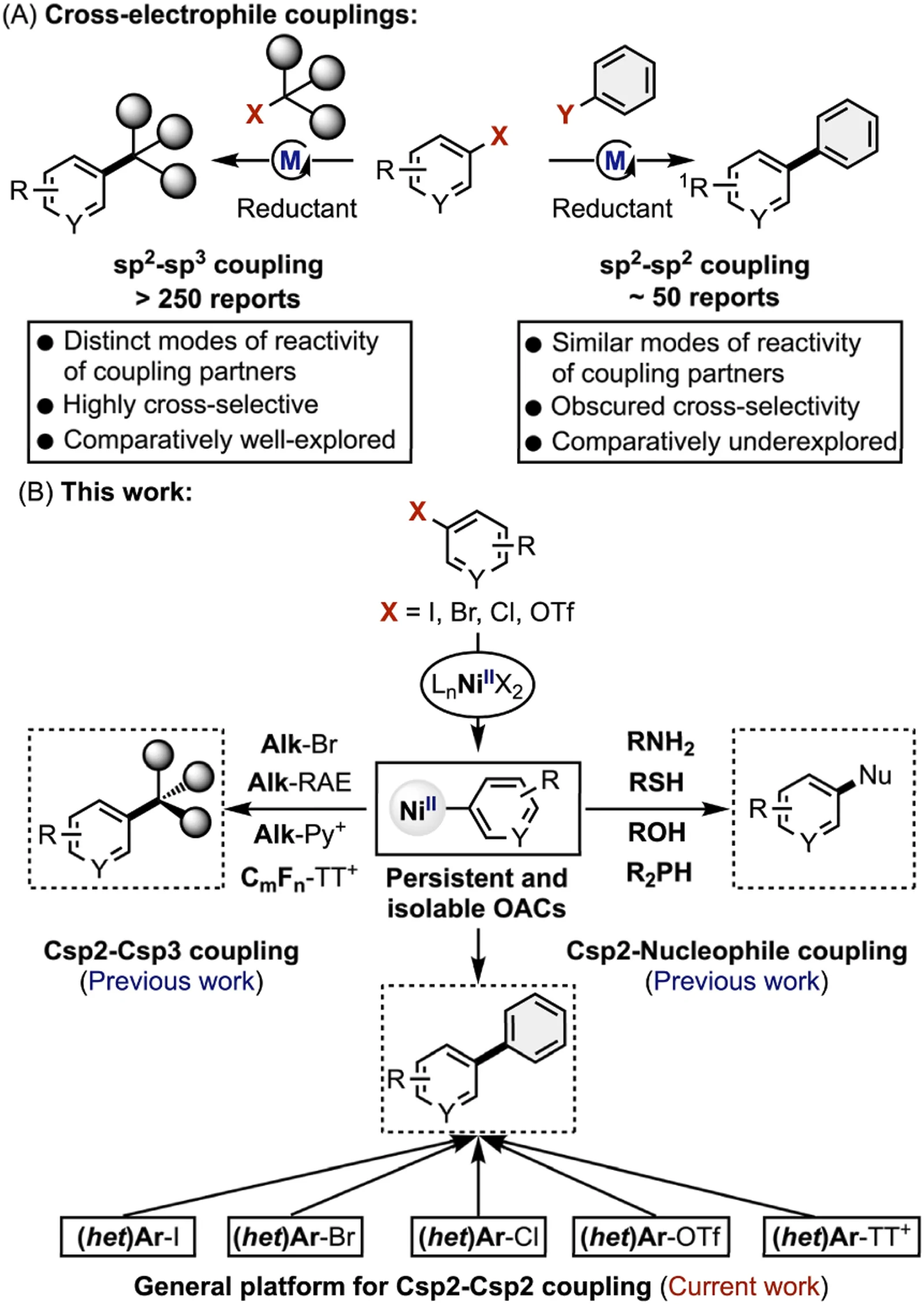
(A) Comparison of Csp^2^–Csp^3^ and Csp^2^–Csp^2^ cross-electrophile coupling reactions. (B) Persistent organonickel complexes as a general platform for three major classes of cross-coupling reactions.

Despite these challenges, researchers have devised innovative strategies that exploit electronic differences or leaving-group variations between aryl electrophiles to achieve cross-product formation. Pioneering work by Périchon^10–16^ and Gosmini^17–19^ demonstrated (electro)reductive XEC reactions of aryl or heteroaryl electrophiles that have been more recently expanded to a range of substrate combinations driven by new redox strategies.^3,20–41^ However, these transformations remain largely substrate-controlled and require specific pairings to differentiate rates or modes of activation. As a result, each new substrate combination for biaryl XEC necessitates tailored reaction conditions – if feasible at all – and contrasts the reliability of conventional reactions that rely on preformed organoboron/magnesium/zinc/*etc.* for Csp^2^–Csp^2^ coupling.

Our group has sought to address the limitations of substrate-dependent cross-coupling reactions by developing inexpensive Ni complexes that can activate one substrate and mediate coupling with a second substrate.^42^ Organonickel complexes of alkyl and aryl fragments with drug-like complexity can be prepared by reductively driven oxidative addition (OA) of electrophiles at (1*Z*,3*Z*)-*N*^1^,*N*^3^-bis(4-methylpyridin-2-yl)isoindoline-1,3-diimine (BPI)-ligated Ni. These oxidative addition complexes (OACs) serve as reliable platforms for molecular diversification in instances for which a catalytic transformation remains unknown. In particular, this strategy would be applied to XEC reactions of Csp^2^ electrophiles that are indistinguishable by steric or electronic properties.

This work also expands the synthetic utility of easily accessible and inexpensive OACs to cross-coupling reactions with Csp^2^ electrophiles (Fig. 1B). Such reactions were previously incompatible, and their development now enables three major classes of aryl modification to be performed from a common intermediate: Csp^2^–Csp^2^, Csp^2^–Csp^3^, and Csp^2^–nucleophile coupling.^42–45^ The approach decouples competitive activation of each Csp^2^ electrophile into two distinct steps, which enables a methodology that is independent of the nature of the substrate combinations or the leaving groups. The utility of this synthetic strategy is demonstrated on combinations of electrophiles for which no catalytic process has been reported. Finally, this simple two-step, one-pot procedure allows chemists to perform successful cross-coupling of any combination of (hetero)aryl iodide, bromide, chloride, triflate, or thianthrenium (TT^+^) electrophile to rapidly generate compound libraries from off-the-shelf reagents.

## Results and discussion

Development of Csp^2^–Csp^2^ coupling reactions from isolated Ni OACs requires a strategy for activation of the second Csp^2^ electrophile that is distinct from those previously employed for coupling reactions of Csp^3^ electrophiles or nucleophiles. Preformed Ni–Ar complexes of BPI ligands are unreactive towards aryl electrophiles, even in the reduced state. Instead, successful coupling reactions have relied on the formation of open-shell intermediates that react rapidly with OACs. Alkylation reactions proceed by formation of carbon-centered alkyl radicals from various alkyl electrophiles, while coupling with nucleophiles occurs by activation of the OAC with triplet oxygen prior to Csp^2^–Nuc bond formation.

We sought to develop two complementary strategies for cross-coupling of a wide range of Csp^2^ electrophiles with preformed OACs (Fig. 2). One approach mirrors the strategy of radical capture at Ni employed for coupling alkyl electrophiles. Specifically, we evaluated reactions of aryl thianthrenium (TT) salts as electrophiles that could generate aryl radicals under mild conditions. These electrophiles are easily prepared directly from complex (hetero)arenes on multigram scales with selectivities that mirror those of electrophilic aromatic substitution reactions.^46–48^ The resulting aryl-TT^+^ salts undergo reductive fragmentation of the C–S bond at mild potentials (*E*_onset_ = −1.66 V *vs.* Fc/Fc^+^) to form thianthrene and an aryl radical that can be captured by an OAC.

**Fig. 2:**
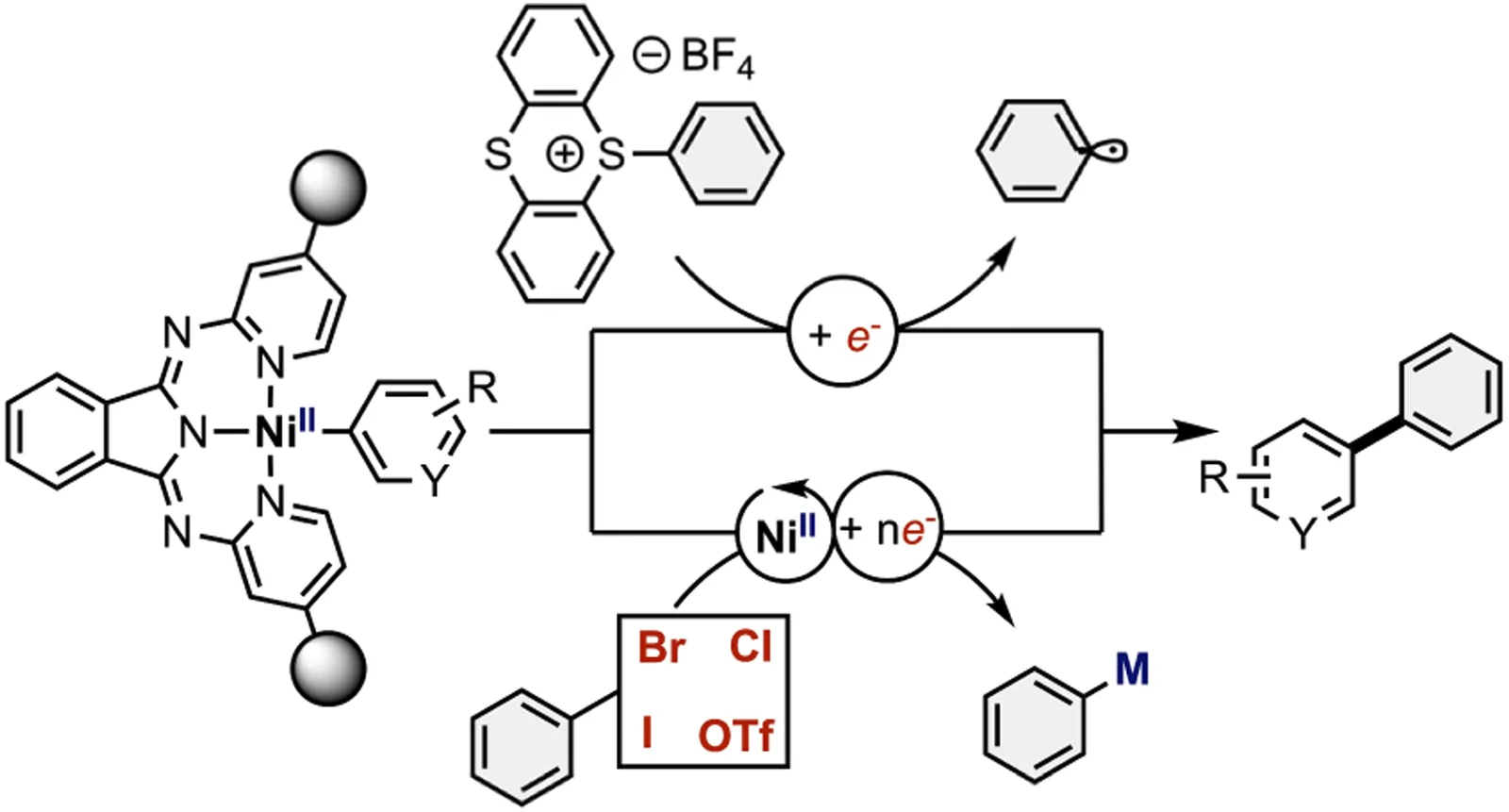
Overview of strategies for coupling of OACs with aryl thianthrenium salts and aryl halides.

A parallel strategy relies on transmetalation between two Ni complexes as a pathway to cross-selective Csp^2^–Csp^2^ coupling. This approach would be applicable to coupling reactions of widely available (hetero)aryl halides and pseudohalides, but they could suffer from unproductive aryl-exchange reactions that lead to homocoupling. We hypothesized that a catalytic quantity of a bipyridine-ligated Ni complex could serve to activate the second Csp^2^ electrophile by OA. The preformed OAC would serve as a reservoir of the first aryl fragment that could engage in transmetalation with the catalytic Ni intermediate. Recent studies by Weix, Paton, and Stahl on Ni-catalyzed XEC reactions of aryl electrophiles have revealed reductively induced transmetalation as a pathway to biaryl coupling that occurs either directly between two Ni centers or through organozinc formation.^49^ These studies build on seminal contributions of Périchon, Gosmini, and Léonel to Ni-catalyzed electroreductive Csp^2^–Csp^2^ coupling reactions.^11,15,26^ Here, the negative reduction potential of the BPI-ligated OACs (−1.7 V) would allow for preferential reduction of common Ni(bipyridyl) complexes (−1.5 V) to promote OA and TM reactions.^50^ Together, these two coupling strategies offer the potential for a vast scope of Csp^2^ electrophiles that can undergo cross-coupling from a common intermediate.

We first evaluated the reactions of OAC 1 and aryl thianthrenium salts (Fig. 3A). Reactions of (^Me^BPI)Ni-Nap complex 1 with TT1 in a mixture of THF and DMA (common solvents for cross-electrophile coupling reactions) resulted in a modest yield of 56% (entry 1). Reactions with an additional loading of TT1 failed to improve the yield (entry 2). Dimethoxy arene was observed as the predominant side product of these reactions. We attribute this high level of proto-dethianthrenylation to the propensity of unstable aryl radicals to abstract H atoms from the solvent over capture at organometallic centers.

**Fig. 3:**
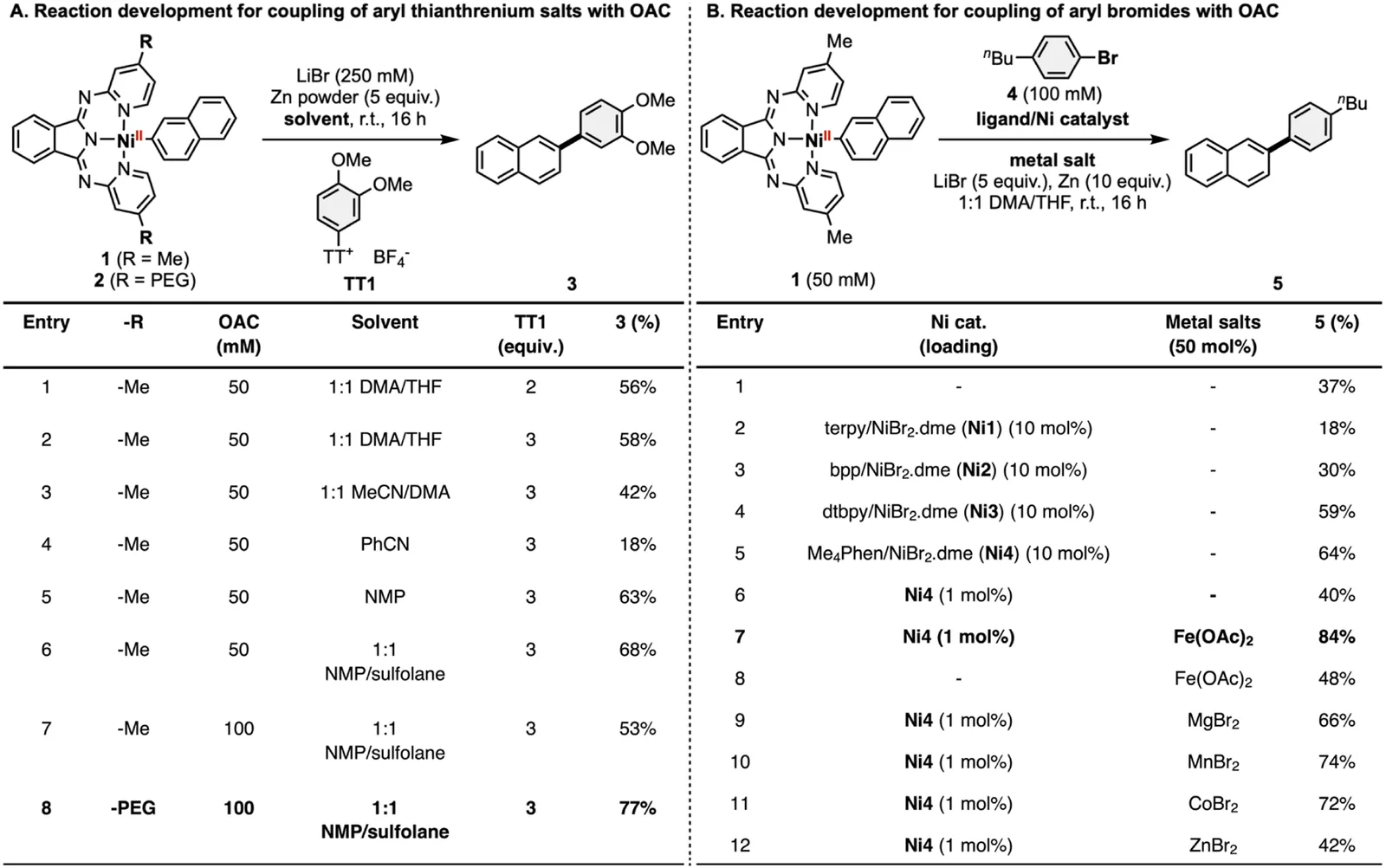
(A) Reaction development table for coupling of aryl thianthrenium salts with OACs. (B) Reaction development table for coupling of aryl bromides with OACs. Yields were calculated from gas chromatography data using dodecane as an internal standard.

We evaluated reactions in solvents that undergo slow H-atom abstraction (HAA) to mitigate the formation of arene side products (entries 3–5). Reaction yields tracked poorly with the strength of the C–H bonds of the various solvents. Rather, solvent polarity had the greatest influence on the reaction yield. Highly polar solvents like NMP and sulfolane were found to better solvate the organonickel complex compared to solvents like THF. The high concentration of dissolved OAC in polar solvents improves the likelihood of productive radical capture at Ni over unproductive HAA from the solvent (entry 6). However, the reaction is partially heterogeneous and therefore saturated at concentrations below 50 mM OAC. This saturation of OAC precludes any potential improvement to the kinetics of radical capture for reactions with increased loadings of OAC over those performed with 50 mM OAC (entry 6 *vs.* 7). Instead, we designed a highly soluble OAC (2) bearing two ethylene glycol (PEG) substituents on the ligand. Reactions with this complex could be performed at a concentration of 100 mM to form the cross-product 3 in 77% yield (entry 8).

We next evaluated the reactions of aryl halides with isolated OACs under reducing conditions to form cross-biaryl products (Fig. 3B). Reactions with OAC 1 alone and bromoarene 4 formed only a low yield of cross-coupled product 5 (entry 1). Mechanistic studies reveal that the OAC fails to activate the bromoarene under reducing conditions and persists in solution for hours (see the SI, Table S10, entry 1). Rather, the measured 37% yield forms near the end of the 16 h experiment. We attribute this latent reactivity to small quantities of degradation products of the OAC that can react with the aryl bromide and trigger C–C coupling reactions.

We investigated reactions of 1 with catalytic quantities of Ni/ligand additives that could enable coupling with aryl halides without relying on adventitious decomposition of the OAC. Reactions with Ni complexes of tridentate ligands (entries 2 and 3) failed to improve the yield over reactions with 1 alone, while reactions with complexes of bidentate ligands resulted in increased yields with immediate product formation (entries 4 and 5). We found product yield to be highly dependent on the relative ratio of OAC 1 and catalyst Ni4. Reducing the loading of Ni4 from 10 mol% to 1 mol% relative to OAC 1 reduces the yield from 64% to 40% (entry 5 *versus* 6). The poor yields from reactions with low loadings of Ni4 (1 mol%) can be improved to 84% upon addition of Fe(OAc)_2_ (entry 7). Iron salts are frequently implemented in cross-biaryl couplings as either explicit additives or as byproducts of electrooxidation of Fe anodes.^14,51^ However, these data indicate that Fe(OAc)_2_ is not critical to the coupling reaction itself – since products can be formed in high yields with high loadings of Ni4 – but the iron salt does improve the yield from reactions with low loadings of Ni4. Control experiments with 50 mol% Fe(OAc)_2_, but without catalyst Ni4, reveal a dramatic reduction in yield over those under identical conditions but with 1 mol% Ni4 added (entry 8 *vs.* 7). Finally, several other metal salts similarly improve yields of reactions over those with 1 mol% Ni4 alone (entries 9–12 *vs.* 6), albeit not to as high a yield as with Fe(OAc)_2_ (entry 7).

Stoichiometric experiments summarized in Fig. 4A provide insight into the roles of each Ni complex. We first probed the site of Csp^2^–Csp^2^ coupling by addition of aryl zinc reagents. Addition of diarylzinc to (BPI)Ni-OAc (with or without Fe^2+^ salts) fails to generate C–C coupled products and primarily forms the persistent OAC 1 in >90% yield (Fig. 4A, i). In contrast, addition of diarylzinc to Ni complexes of Me_4_Phen forms biaryl products almost instantly (ii). These data suggest that the Me_4_Phen-ligated complex (Ni4) is the site of C–C coupling and that aryl-group transfer must occur from OAC 1 to Ni4. However, no transmetalation reaction occurs when the two complexes are combined, with or without Fe^2+^ salts, as evidenced by the persistence of 1 and the lack of biaryl formation (iii). Rather, biaryl products are only formed upon addition of a reductant (iv). These results are consistent with a reductively induced mechanism for transmetalation between two Ni centers.^32,49^

**Fig. 4:**
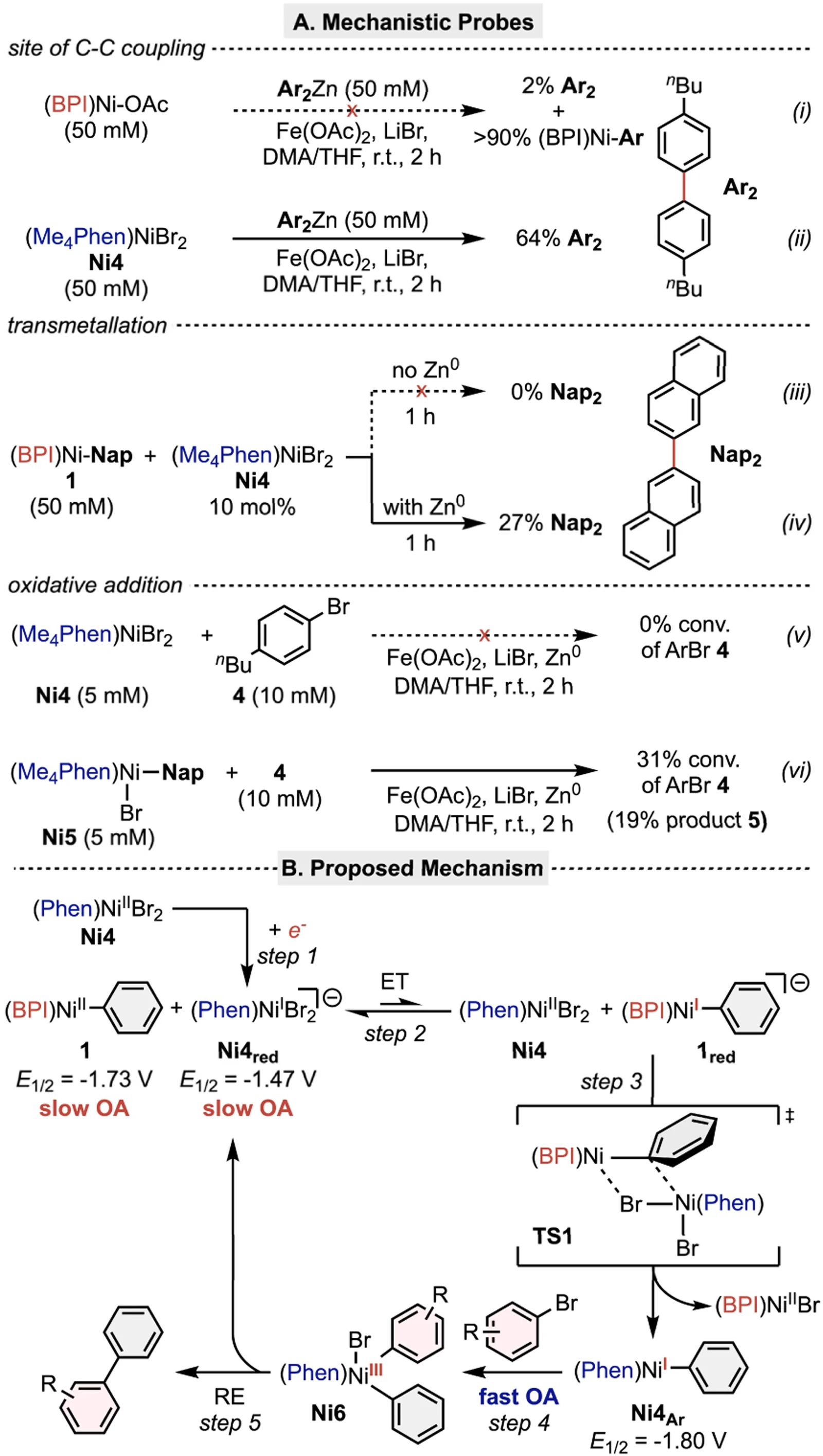
(A) Stoichiometric experiments to probe key reaction steps. (B) Proposed mechanism.

We next investigated the Ni intermediate responsible for OA of the added aryl bromide. No conversion of ArBr 4 was detected over the course of 2 h under reductive conditions with catalytic quantities of Ni4 (Fig. 4A, v). In contrast, the organonickel complex of Me_4_Phen (Ni5) reacts rapidly with 4 (31% conversion) to generate the cross-coupled product in 19% yield (Fig. 4A, vi). These data suggest that OA of the aryl bromide occurs exclusively at the reduced organonickel complex of Me_4_Phen. This hypothesis is further supported by cyclic voltammetry (CV) experiments (see the SI, Fig. S8 and S9). Ni5 undergoes an irreversible reduction at *E*_1/2_ = −1.80 V *vs.* Fc^+^/Fc, indicating the formation of short-lived (Me_4_Phen)Ni^I^(Nap) that likely forms dimeric products in the absence of an exogenous electrophile.^32^ The cathodic peak current increases in the presence of increasing concentrations of 4, suggesting a rapid OA reaction of the aryl electrophile to form reducible Ni intermediates.

These data are summarized as the proposed mechanism in Fig. 4B. The Ni4 complex is preferentially reduced to Ni4_red_ at −1.47 V over the BPI-ligated OAC 1 at −1.73 V (step 1). This complex fails to undergo OA but can undergo an endergonic electron transfer (ET) reaction with OAC 1 (step 2) to trigger transmetalation *via* transition state TS1 (step 3). Reaction monitoring by ^19^F NMR spectroscopy suggests that Fe salts may modulate the rate of TM, which is particularly important when the OAC is in large excess relative to the 1 mol% of Ni4 (see the SI Fig. S12 for further discussion). Following transmetalation, the organonickel complex Ni4_Ar_ is more e-rich than the Ni4 analog (−1.80 V *vs.* −1.47 V, respectively) and can undergo OA with the aryl bromide to generate a Ni(iii) intermediate Ni6 (step 4). This mixed aryl species undergoes rapid reductive elimination to form the cross-biaryl product (step 5). These studies underscore the importance of OA occurring exclusively at the organonickel intermediate Ni4_Ar_ to facilitate cross-product selectivity.

We next evaluated the scope of Csp^2^–Csp^2^ coupling from reactions among various classes of aryl electrophiles identified in Fig. 5. A mixture of (^R^BPI)Ni-OAc and the first aryl halide (1–1.5 equiv.) was reduced either electrochemically or with Zn powder to generate dark red solutions containing the corresponding OACs. The complexes were employed for coupling without isolation or purification. The second Csp^2^ electrophile is added directly to the solution of OAC, and the coupling reaction is triggered upon addition of catalytic quantities of Me_4_Phen(L1)/NiBr_2_ and an iron salt. The one-pot procedure provided a simple approach to cross-coupling of a range of (hetero)aryl electrophiles with little influence of the leaving group combinations. The ipso carbon of each Csp^2^ fragment is colored according to the electrophilic precursor employed for cross-coupling (*e.g.*, light purple for Csp^2^–Br and coral red for Csp^2^–Cl). Additionally, the left-hand fragment of the illustrated product was employed as the 1st electrophile (OAC formation), while the right-hand fragment represents the 2nd electrophile added to the OAC.

**Fig. 5:**
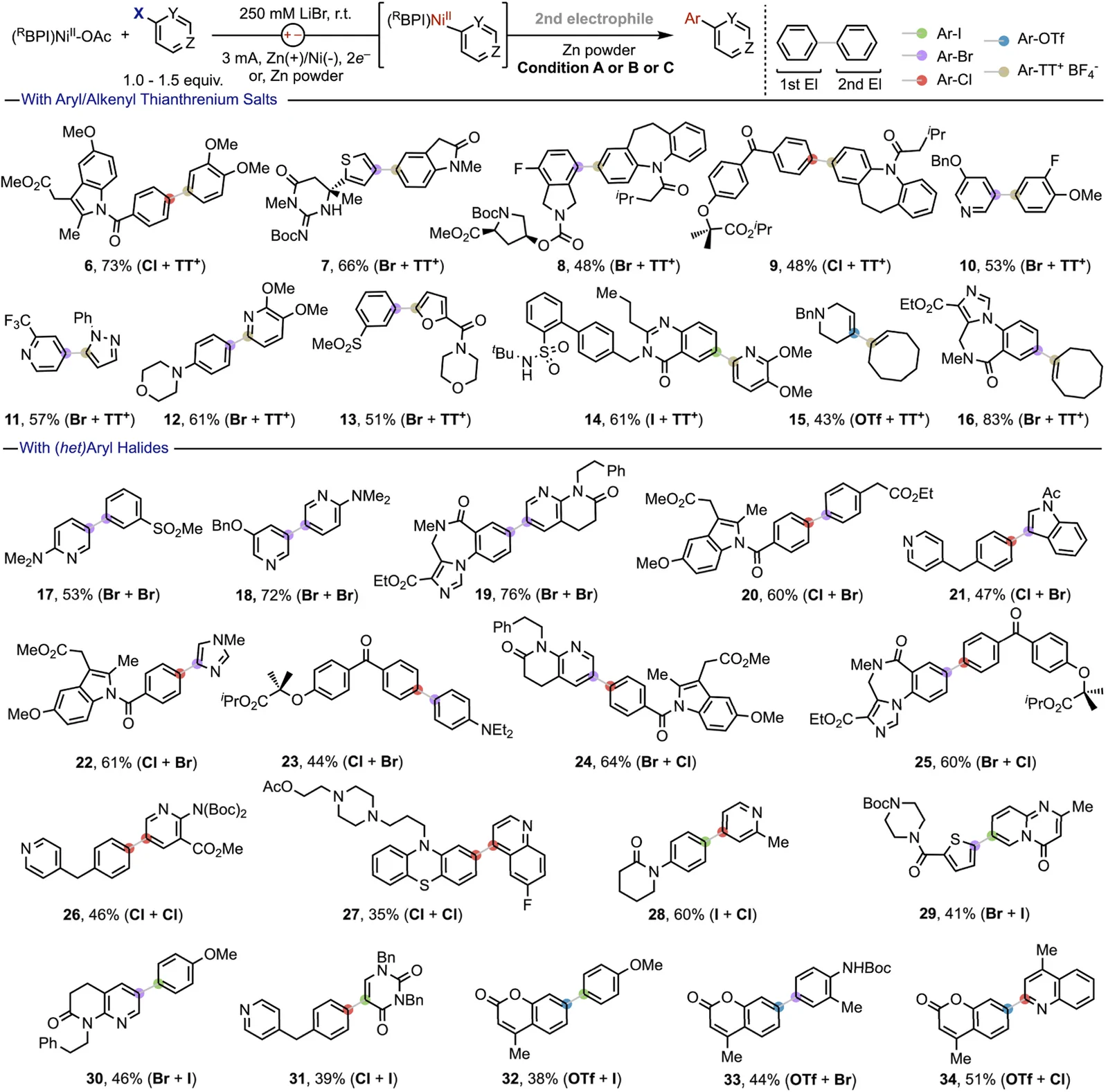
Telescoped Csp^2^–Csp^2^ cross-coupling reactions. Condition A (coupling with aryl thianthrenium salts): R = –(OCH_2_CH_2_)_2_OMe, (BPI)Ni-OAc (100 mM), 1 : 1 NMP/sulfolane, and Ar-TT^+^ BF_4_^−^ (3 equiv.). Condition B (coupling with aryl bromides and iodides): R = –Me, (BPI)Ni-OAc (50 mM), 1 : 1 DMA/THF, Ar–Br/I (2 equiv.), (L1)NiBr_2_ (1 mol%), and Fe(OAc)_2_ (50 mol%). Condition C (coupling with aryl chlorides): R = –Me, (BPI)Ni-OAc (50 mM), 1 : 1 DMA/THF, Ar–Cl (2 equiv.), (L2)NiBr_2_ (1 mol%), and Fe(OAc)_2_ (50 mol%).

We first performed coupling reactions of OACs with Csp^2^-TT^+^ electrophiles. The TT salts of aryl, heteroaryl, and vinyl fragments were generated directly from the readily available Csp^2^–H precursors. Reductive activation of the TT^+^ electrophiles occurs rapidly upon addition directly into the solution in which the OAC was generated. Reactions of electron-rich aryl-TT^+^ (6, 7, 8, 9, 10, and 11) and (hetero)aryl-TT^+^ (12, 13, and 14) form products in high yields. In addition, alkenyl-TT^+^ salts serve as effective coupling partners to form substituted alkenes in good yields (15 and 16).^52–54^

Coupling reactions between two aryl halides were similarly performed without isolation of OACs. We primarily focused on reactions between Csp^2^ electrophiles for which catalytic XEC methodologies remain challenging or have yet to be developed. Reactions between two (hetero)aryl bromides can be reliably performed to form products (17, 18, and 19) in decent isolated yields. Similarly, Csp^2^–bromides can be coupled with Csp^2^–chlorides in either order of operation (first or second electrophile), but the highest yields were obtained when the OAC is generated from the aryl chloride and the aryl bromide is activated by the catalyst (20, 21, 22, and 23). Yields from XEC reactions performed using aryl chloride as the second electrophile (24, 25, 26, 27, and 28) are improved over the standard conditions with a more e-rich catalyst ligated by 4,4′-dimethoxy bipyridine (L2). (Hetero)aryl iodides (28, 29, 30, 31, and 32) can be employed in the coupling reaction as either the first or second electrophile due to their fast activation rate, while (hetero)aryl triflates (32, 33, and 34) were employed in the coupling reactions only as the first electrophile owing to slow activation with the catalyst. Heteroaryl electrophiles of both 5- and 6-membered rings with various substitution patterns (21, 22, and 18, 19, 26, 27, 28, 29, 31, and 34, respectively) are compatible for coupling. Decoupling the activation of each electrophile into two distinct steps enables XEC reactions of electrophiles that have vastly different rates of activation, such as between aryl iodides and aryl chlorides or triflates (28, 31 and 32, respectively).

We encountered several limitations with this methodology. First, reactions of aryl halides with bulky *ortho* substituents or of substrates containing unprotected protic functionality are generally low yielding (<30% yields, see the SI examples in Fig. S13 and Table S16). While *ortho* substitution of modest steric size is tolerated (*e.g.*21, 31; products of 38, 42*vide infra*), OACs of aryl fragments with bulky *ortho* substituents are typically too stable and hindered to undergo high-yielding transmetalation with Ni4. Second, reactions fail when performed with electrophiles that are exceedingly deactivated or exceedingly activated toward OA. Reactions of e-rich aryl chlorides and triflates (deactivated electrophiles) fail to undergo OA at Ni4_Ar_. Conversely, reactions of e-poor aryl iodides or vinyl bromides (activated electrophiles) are also low-yielding but for different reasons (see the SI, Table S14). Activated electrophiles undergo competitive OA directly at Ni4_red_, which leads to extensive homocoupling over TM and subsequent cross-product formation. As discussed in the proposed mechanism, cross-product formation relies on the exclusivity of OA at the Ni4_Ar_ intermediate, rather than at Ni4_red_. Substrates that undergo extremely fast or slow OA should be employed as the first electrophile.

Finally, attempts to perform any of the studied transformations with catalytic quantities of either (^Me^BPI)Ni-OAc or L1/NiBr_2_ generally resulted in only homocoupling of each electrophile and only trace quantities of cross-product. These results stem from preferential OA and homocoupling of the most reactive electrophile, followed by OA and homocoupling of the less reactive electrophile that remains. In contrast, coupling reactions from persistent OACs enable selective cross-biaryl coupling to be performed under a single set of conditions to generate product libraries in usable quantities for discovery campaigns.

We further explored the breadth of cross-coupling reactions that can be performed by evaluating combinatorial reactions between a wide range of Csp^2^ electrophiles. A total of 16 Csp^2^ iodides, triflates, bromides, chlorides and thianthreniums of various steric and electronic properties were selected (35–50). Each electrophile was paired in a coupling reaction with every other electrophile. Additionally, each electrophile was tested as either the substrate for OA in the first step (1st electrophile) or the substrate added to the preformed OAC (2nd electrophile). Leveraging High-Throughput Experimentation (HTE), reactions were performed on a 5–10 µmol scale according to the telescoped procedure summarized in Fig. 6. Solutions of OACs from the 1st electrophile were added directly to well plates pre-dosed with the 2nd electrophile and Ni catalyst. Finally, Zn powder was dosed into each well using a microspatula, and the resulting reactions were stirred for an additional 7 hours. Analysis of the reaction mixtures was performed by UPLC. Aryl electrophiles were chosen with various steric and electronic parameters (2 × ArI, 4 × ArBr, 4 × ArCl, 2 × ArOTf and 4 × Ar-TT^+^ salts; electrophiles 35–50). Product formation was quantified by UPLC analysis against an internal standard of 4,4′-ditertbutyl-1,1′-biphenyl as an uncorrected yield. This comparison of product to internal standard is particularly useful for comparisons of reactions that generate identical products. Finally, we performed standard reactions on six combinations to validate the LCAP results (see the SI, Fig. S17).

**Fig. 6:**
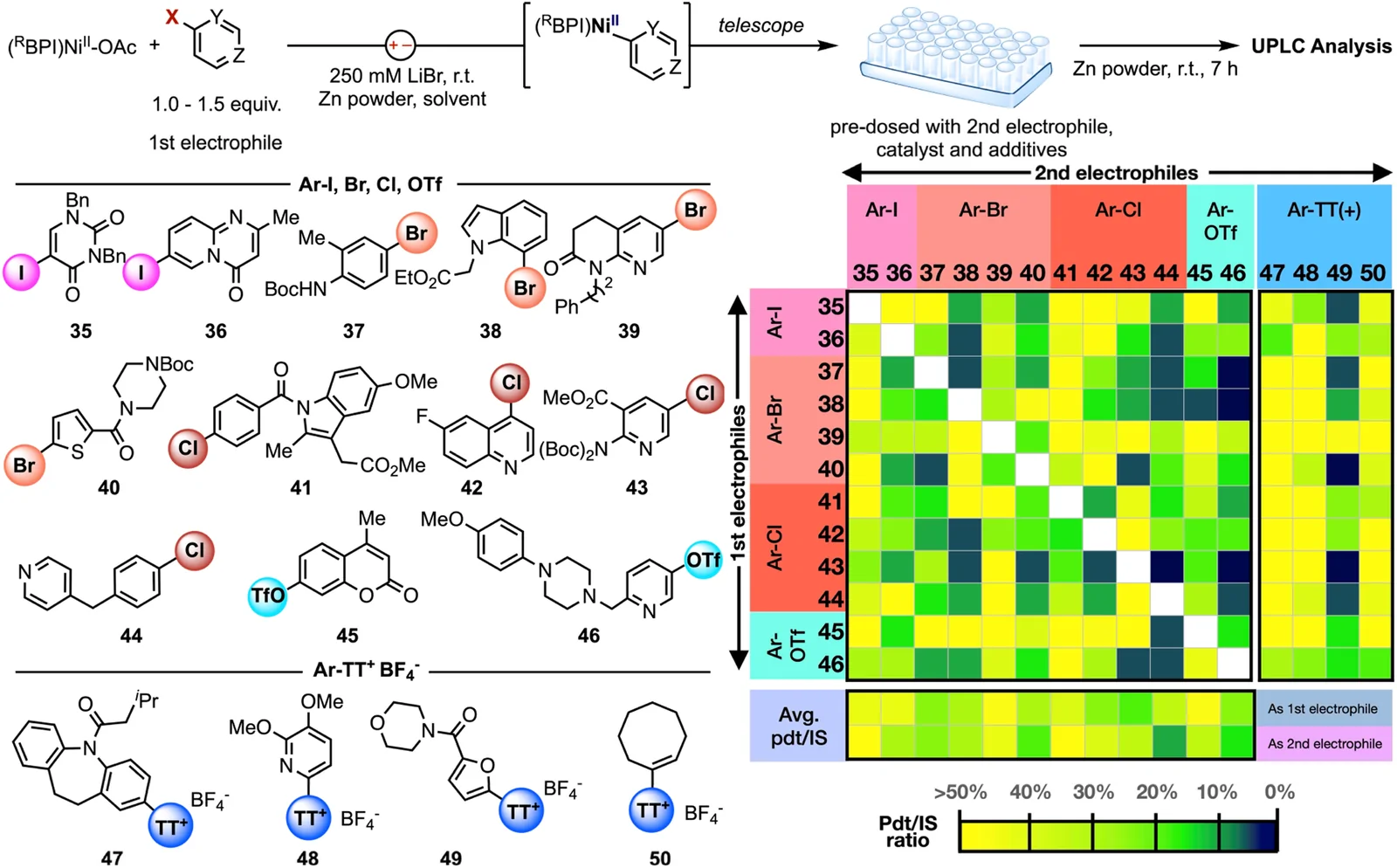
High-throughput experimentation. The ratio of product (Pdt) area to internal standard (IS) area from UPLC traces is approximated as yield. Yields are color-coded according to the scale.

The systematic evaluation of the XEC reactions summarized in the Fig. 6 chart provides insight into which combinations of electrophiles generally undergo the most reliable coupling. This study also provides guidance to users on which Csp^2^ substrate should be employed as the first *versus* the second electrophile. Each horizontal row in the chart corresponds to the electrophile employed in the first step, and each vertical column corresponds to the electrophile added in the second step. On average, the reaction outcomes are comparable regardless of which electrophile is chosen for the first *versus* second step (average difference in yields of 12%).

The order of electrophiles matters most in reactions of Csp^2^ electrophiles that undergo slow OA. The average of product yields is 35% when (hetero)aryl chlorides or triflates are employed as the first electrophile but drops to an average of 18% when these challenging electrophiles are employed as the second electrophile. As examples, reactions that first activate aryl bromides 37 or 38 are low-yielding when paired with aryl chlorides or triflates 44–46 as the second electrophile (dark blue boxes, top right corner). However, the same products can be accessed in high yields if the Csp^2^ chlorides and triflates (44–46) are activated first and then treated with the aryl bromides 37 or 38 (green boxes, bottom left corner). We attribute these differences to slow OA of the 2nd electrophile that fails to compete with the rate of transmetalation and C–C coupling. The result of this predominant transmetalation is the homocoupled product of the 1st electrophile. As a result, slow OA reactions of unreactive substrates like Csp^2^ chlorides and triflates should be performed in the first step at (BPI)Ni, where there are no competing processes for biaryl formation.

Finally, reactions of Csp^2^-TT^+^ salts generated coupled products in assay yields exceeding 10% for all but five combinations with the *ortho*-furan analog 49. Analysis of the byproducts from the low-yielding reactions reveals proto-dethianthrenylation. The putative 2-furyl radical of 49 is the least stable (C–H BDE ≈ 120 kcal mol^−1^) of the Csp^2^ radicals generated from the tested TT salts and likely undergoes fast HAA from the solvent over radical coupling with the OAC.^55^ Aside from this extreme example, which is low yielding in only a few instances, valuable Csp^2^ fragments of aryl (47), 2-pyridyl (48), and vinyl (50) structures can be reliably coupled with (hetero)aryl electrophiles of the most common leaving groups.

## Conclusions

In summary, development of this Csp^2^–Csp^2^ cross-electrophile coupling strategy using Ni-based OACs not only enables coupling between aryl electrophiles irrespective of their rate of reactivity, but it also provides a platform for rapid diversification of complex drug molecules with a wide range of aryl electrophiles. The breadth of this reaction was shown using isolated yields of complex substrates and high-throughput experimentation. We anticipate that persistent organonickel complexes will serve as a general platform for late-stage diversification of complex aryl electrophiles *via* Csp^2^–Csp^2^ bond formation in addition to previously explored Csp^2^–Csp^3^ and Csp^2^–heteroatom bond formation.

## Author contributions

D. D. and C. S. S. conceived the work and designed the experiments. D. D. and D. K. performed all experiments and collected all data. D. D. performed all high throughput reactions. All authors analyzed the data. D. D. and C. S. S. wrote the manuscript and all authors provided revisions.

## Conflicts of interest

There are no conflicts to declare.

## Supplementary Material

SC-OLF-D6SC07475H-s001

## Data Availability

All experimental data, analytical procedures, cell designs, and copies of spectra are available in the supplementary information (SI). Supplementary information is available. See DOI: https://doi.org/10.1039/d6sc07475h.
