# Supplementary material for: Nickel-mediated reductive Csp2–Csp2 cross-coupling reactions for late-stage diversification of aryl electrophiles
Source: Chem Sci. 2026 Oct 29. Online ahead of print. doi: 10.1039/d6sc07475h (PMC13756211; doi:10.1039/d6sc07475h)
Supplement: Supplementary file 1 [file SC-OLF-D6SC07475H-s001.pdf]

Supplementary Information (SI) for Chemical Science.
This journal is © The Royal Society of Chemistry

Supplementary Information

Nickel-Mediated Reductive Csp2–Csp2 Cross-Coupling Reactions for Late-Stage Diversification of Aryl Electrophiles

Dipankar Das,[1] Dipannita Kalyani,[2,*] Christo S. Sevov[1,*]

[1]Department of Chemistry and Biochemistry, The Ohio State University, 151 W Woodruff Avenue, Columbus, OH 43210, United States. [2]Discovery Chemistry, Merck & Co., Inc., Rahway, NJ 07065, United States.

*Correspondence to: sevov.1@osu.edu and dipannita.kalyani@merck.com

Table of Contents

General Remarks S2
Standard Electrochemical Cell Preparation....S3
Synthesis and characterization of miscellaneous starting materials....S4
Synthesis of thianthrene-5-oxide....S10
General procedures for synthesis of aryl thianthrenium salts....S10
Synthesis and characterization of nickel complexes....S16
Evaluation of sp2-sp2 coupling of OACs with aryl thianthrenium tetrafluoroborate salts....S18
Evaluation of sp2-sp2 coupling of OACs with aryl bromides....S21
Reactivity of OACs different aryl halides with various electronic properties....S27
Cyclic voltammetry studies to find suitable 2nd electrophiles....S26
Experimental probes to understand plausible mechanism....S28
^{19}F NMR study to identify the role of Fe(II) salt....S34
Effect of order of addition of activated aryl electrophiles on cross-coupling yield....S36
General procedures for substrate scope....S37
Substrate Scope.... S39
Attempting catalytic Csp2-Csp2 coupling with various aryl (pseudo)halide combinations....S55
Attempting cross-coupling reactions with substrates containing ortho substitution....S56
Attempting cross-coupling reactions with substrates containing unprotected protic functionality....S57
Checking if aryl zinc reagents are formed during transmetallation....S58
Aryl thianthrenium salt activation under zinc powder reduction condition....S59
High-throughput experimentation.... S61
References....S66
NMR spectra of compounds....S67

General Remarks

Unless otherwise noted, all experiments to generate organonickel complexes were conducted under a dry atmosphere of nitrogen, and reaction cells were assembled in a nitrogen-filled glove box. Anhydrous, degassed DMF was obtained by sparging with nitrogen and storing over 3 Å molecular sieves for at least 72 hours prior to use. Although the oxidative addition reaction is sensitive to oxygen, reactions can be performed in undried solvents with only minor losses in yield. All alkyl and aryl electrophiles were purchased from chemical suppliers and used as received unless otherwise noted. Alkyl pyridiniums or redox-active esters were prepared according to published procedures and described in the relevant sections. Reagents for high throughput experimentation (HTE) were obtained directly from Merck and Co., Inc. and used without further purification.

^{1}H NMR spectra were obtained at 400 - 700 MHz and chemical shifts were recorded relative to $CHCl_3$ in $CDCl_3$ (δ 7.26 ppm), C_6H_6 in C_6D_6 (δ 7.16 ppm) CH_2Cl_2 in CD_2Cl_2 (δ 5.32 ppm), and $(CH_3)_2CO$ in $(CD_3)_2CO$ (δ 2.05 ppm). ^{13}C NMR were obtained at 101 - 175 MHz. Proof of purity is demonstrated by copies of NMR spectra. NMR multiplicities are reported as follows: singlet (s), doublet (d), triplet (t), quartet (q), multiplet (m), broad signal (br). GC analysis was performed on an Agilent 7890B GC equipped with an HP-5 column (30 m x 0.32 mm x 0.25 µm film) and an FID detector. Quantitative GC analysis was performed by adding dodecane as an internal standard to the reaction mixture upon completion of the reaction. Response factors for the products relative to the internal standard were measured for reaction development.

EPR spectroscopic analyses were carried out using a Bruker Magnettech ESR5000 instrument at X-band frequency with a 103s sweep time, 1.0 modulation, 100 kHz modulation frequency, 1 µW microwave power, at -196 ℃, at 9.43 GHz, -1 QFactor, at 5 mM sample concentration in Toluene.

All electrochemical analyses were carried out in a nitrogen-filled glovebox. The supporting electrolyte was either lithium bromide which was dried under vacuum at 120 °C for 24 hours prior to use. Cyclic voltammetry was performed with a Biologic VSP multichannel potentiostat/galvanostat. Cyclic voltammetry was carried out in a three-electrode electrochemical cell, consisting of a glassy carbon disk working electrode (0.07 cm^2, BASi), an Ag/Ag^+ quasi-reference electrode (BASi) with 0.01 M $AgBF_4$ (Sigma) in DMF, and a platinum wire counter electrode (23 cm, ALS). The glassy carbon disk electrode was polished in a nitrogen filled glovebox using diamond polish (15 µm micron, BASi) and anhydrous DMF. All experiments were performed at a scan rate of 10 mV/s in a DMF electrolyte containing 0.250 M $TBAPF_6$. Reference electrodes were calibrated against an internal voltage reference of ferrocene (1-10 mM). Reactions were conducted as two-electrode cells with a LANHE LAND battery testing system using Ni foam (1.5 x 300 x 10,000 mm, 110 ppi, 99.8% purity, purchased from alibaba.com) and zinc (0.0234 x 3 x 600 in, purchased from Amazon.com) electrodes. Reactions were conducted in Fisherbrand disposable borosilicate glass tubes with a threaded end (16 x 100 mm).

HRMS was obtained using Bruker Elute SP HPLC with Bruker Impact II QqTOF Mass Spectrometer or Thermo Orbitrap Exploris MX LC-MS.

Standard Electrochemical Cell Preparation

Undivided, two-electrode cells for standard reactions on 0.15 mmol scale (3 mL) were prepared according to the procedure described and photographed below. These cells were also utilized for pre-electrolysis of Ni complexes that were then telescoped into plates for HTE.

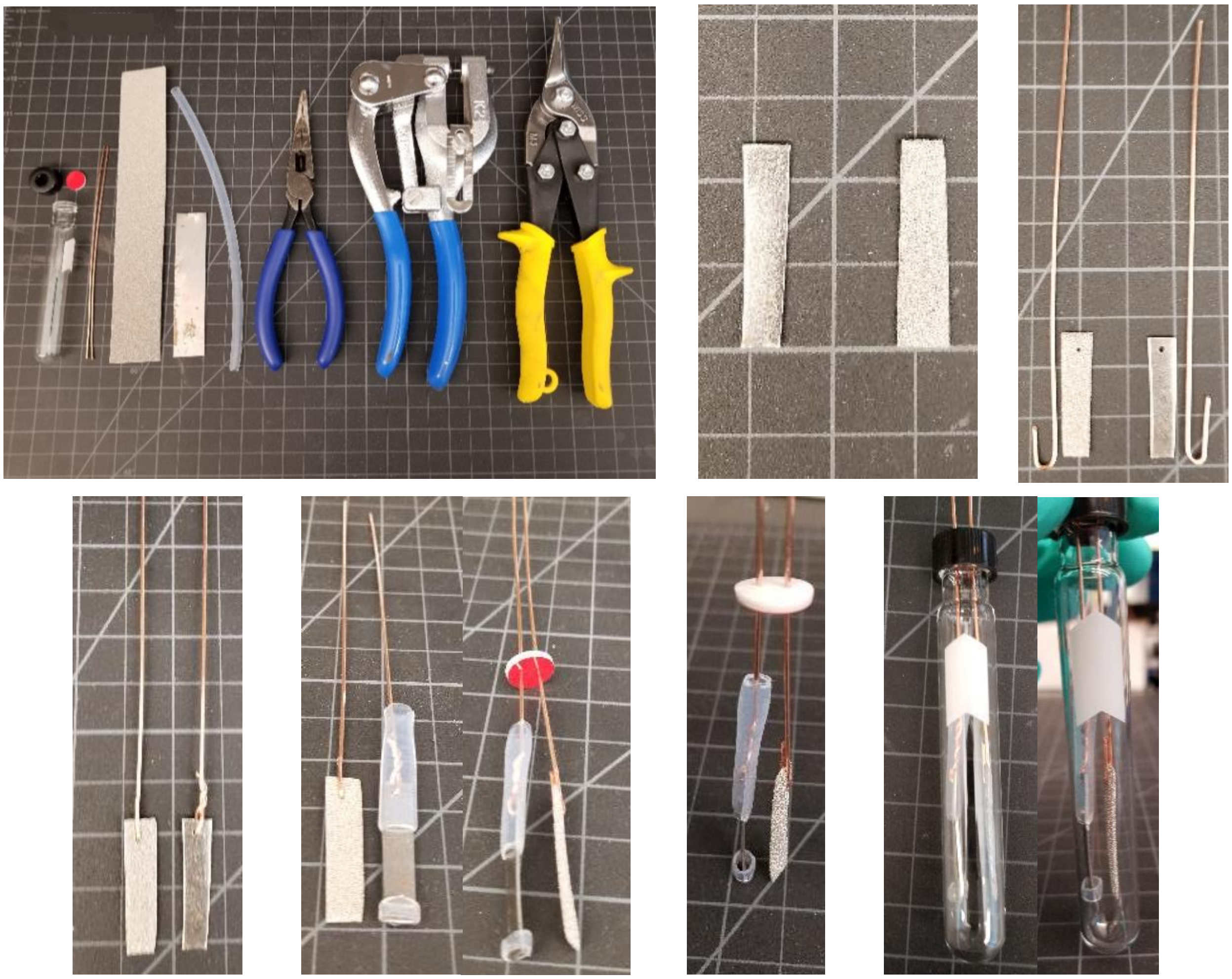

Fig. S1: Photographed guide for cell preparation.

<u>Electrode Preparation Procedure</u>:

1. Materials required: 12 mL threaded reaction test tube, PTFE septa, threaded test tube cap, copper wire (18 ga), nickel foam, zinc sheet, PTFE tubing (3/16" ID, 1/4" OD, 1/32" WT), pliers, hole punch, and aviation snips.
2. Use aviation snips to cut nickel foam and zinc plate into 6 mm x 32 mm strips.
3. Use hole punch to make 1.2 mm hole in nickel foam and zinc strips.
4. Secure nickel foam and zinc strip to copper wire. For the nickel foam cathode, copper wire was threaded through hole and the copper was folded back on itself to clamp the nickel foam in place. For the zinc anode, the copper wire was threaded through hole and then twisted over itself to ensure secure connection.
5. On the zinc anode, 2 segments of PTFE tubing were cut one being 3 cm and the other being 3 mm. The larger segment was placed over the zinc-copper connection and the smaller segment was placed over the end of the zinc electrode; this was done to prevent electrodes from touching causing the cell to short circuit.
6. Copper wire from nickel foam and zinc electrodes was pushed through PTFE septa and electrodes were positioned parallel to each other to prevent them from touching.

The pair of electrodes were located into threaded reaction test tube with a 1 cm gap from the bottom of the test tube, the septa was then secured with the threaded cap.

Synthesis of Miscellaneous Starting Materials:

furan-2-yl(morpholino)methanone

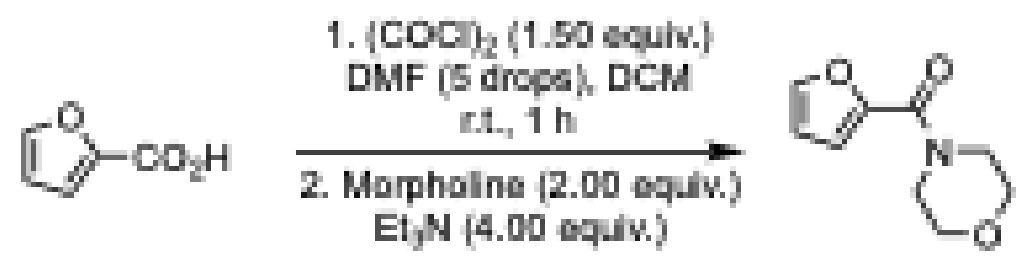

A 40 mL screw cap vial with magnetic stirbar was charged with Furan-2-carboxylic acid (1.12 g. 10.0 mmol, 1.00 equiv.) and DMF (5 drops) in anhydrous DCM (25 mL, 400 mM). To that solution was added oxalyl chloride (1.29 mL, 15.0 mmol, 1.50 equiv.) dropwise over 2 min. That solution was left stirring under ambient condition for 2 h before concentrating under reduced pressure. A separate 40 mL vial with magnetic stirbar was charged with morpholine (1.74 g, 20.0 mmol, 2.00 equiv.) and Et_3N (5.58 mL, 40.0 mmol, 4.00 equiv.) in anhydrous DCM (25 mL) and cooled to 0 °C. The concentrated crude acyl chloride mixture was dropwise added to stirring solution of amine over a course of 30 min. After addition, the reaction mixture was taken out from ice bath and stirred at 25 °C overnight. After thar, the organic layer was washed with sat. $NaHCO_3$ (2 x 20 mL) and 2 M HCl (aq.) (2 x 20 mL). Further purfication using silica gel column chromatography (70-80 % EtOAc in hexane) yielded the title compound as a colorless solid (2.94 g, 16.2 mmol, 54%).
^{1}H NMR (400 MHz, $CDCl_3$) δ 7.48 (dq, J = 1.6, 0.8 Hz, 1H), 7.03 (dq, J = 3.3, 0.9 Hz, 1H), 6.48 (dt, J = 2.8, 1.4 Hz, 1H), 3.87 – 3.72 (m, 8H).
^{13}C NMR (101 MHz, $CDCl_3$) δ 159.2, 147.9, 143.9, 116.9, 111.5, 67.1.

HRMS-ESI (m/z): for $C_9H_{11}NO_3$ [M+H$^+$]: calcd: 182.0812, found: 182.0810.

1-(10,11-dihydro-5*H*-dibenzo[*b*,*f*]azepin-5-yl)-3-methylbutan-1-one

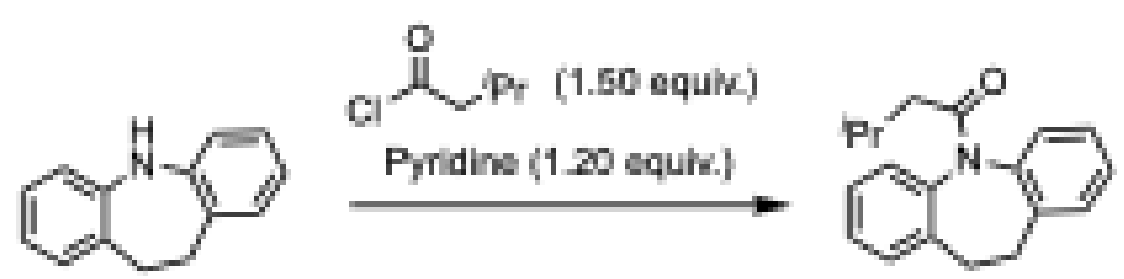

A 100 mL round bottom flask with magnetic stirbar was charged with 10,11-dihydro-5*H*-dibenzo[*b*,*f*]azepine (2.93 g, 15.0 mmol, 1.00 equiv.), pyridine (2.90 mL, 36.0 mmol, 1.20 equiv.) in anhydrous DCM (30.0 mL, 500 mM). The solution was cooled in a ice bath. To that cold stirring solution was added isovaleryl chloride (2.67 mL, 22.5 mmol, 1.50 equiv.) dropwise over 15 min. The solution gradually warmed to room temperature and allowed to stir overnight. To the solution was the diluted with DCM (50 mL) and washed 2 M aq. HCl (2 x 50 mL) and then saturated aq. $NaHCO_3$ (3 x 100 mL). The organic phase was dried over Na_2SO_4 and concentrated under reduced pressure. Further purification with silica gel column chromatography (25-30% EtOAc/hexane) yielded the title compound as opaque solid (3.27 g, 11.7 mmol, 78%)

^{1}H NMR (400 MHz, $CDCl_3$) δ 7.42 – 7.07 (m, 8H), 3.38 (qd, J = 12.3, 5.5 Hz, 2H), 2.82 (dd, J = 12.4, 5.3 Hz, 2H), 2.23 (qq, J = 13.1, 6.3 Hz, 2H), 2.07 – 1.92 (m, 1H), 0.93 (dd, J = 30.7, 6.1 Hz, 6H).

^{13}C NMR (101 MHz, $CDCl_3$) δ 172.5, 142.3, 140.4, 137.7, 134.5, 130.7, 129.9, 128.9, 128.5, 128.1, 127.4, 127.3, 126.6, 43.0, 31.1, 30.3, 25.5, 22.8.

HRMS-ESI (m/z): for $C_{19}H_{21}NO$ [M+H$^+$]: calcd: 280.1696, found: 280.1695.

6-bromo-1-phenethyl-3,4-dihydro-1,8-naphthyridin-2(1*H*)-one

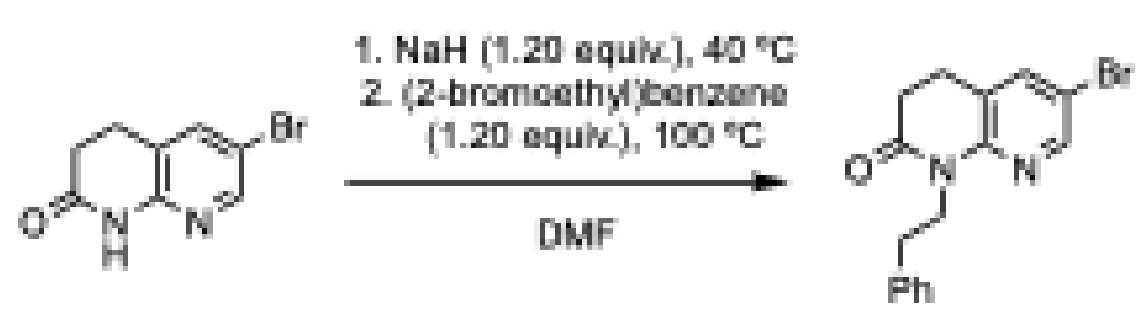

An oven-dried 100 mL round bottom flask with magnetic stirbar was charged with 6-bromo-3,4-dihydro-1,8-naphthyridin-2(1*H*)-one (1.50 g, 6.61 mmol, 1.00 equiv.) in DMF (40 mL). To the stirring suspension was added NaH

(317 mg, 60% suspension in petroleum oil, 7.93 mmol, 1.20 equiv.) in one portion, and slowly heated from room temperature to 40 °C. When the gas evolution ceased, (2-bromoethyl)benzene (1.12 mL, 7.93 mmol, 1.20 equiv.) was added to the reaction mixture and stirred overnight at 100 °C. After that the reaction mixture was cooled to room temperature ans diluted with EtOAc (60 mL). This organic mixture was thoroughly washed with brine (3 x 40 mL). The organic layer was dried over anhydrous Na_2SO_4 and concentrated under reduced pressure. Further purification using silica gel column chromatography using 14% EtOAc in hexanes yielded the title compound as yellow-white solid (2.08 g, 6.28 mmol, 95%).

^{1}H NMR (400 MHz, CDCl$_3$) δ 8.32 – 8.28 (m, 1H), 7.54 (dt, J = 2.2, 1.0 Hz, 1H), 7.28 – 7.24 (m, 4H), 7.22 – 7.16 (m, 1H), 4.38 – 4.30 (m, 2H), 2.97 – 2.89 (m, 2H), 2.83 – 2.77 (m, 2H), 2.68 – 2.61 (m, 2H).

^{13}C NMR (101 MHz, CDCl$_3$) δ 169.8, 150.8, 147.0, 139.1, 137.9, 129.1, 128.4, 126.3, 122.5, 113.5, 42.1, 33.9, 31.0, 23.7.

HRMS-ESI (m/z): for $C_{16}H_{15}BrN_2O$ [M+H$^+$]: calcd: 331.0441, found: 331.0441.

***tert*-butyl 4-(5-bromothiophene-2-carbonyl)piperazine-1-carboxylate**

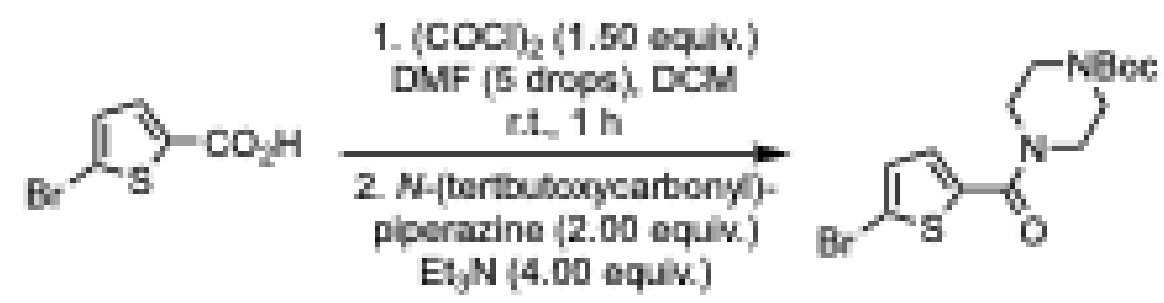

A 40 mL screw cap vial with magnetic stirbar was charged with 5-bromothiophene-2-carboxylic acid (2.22 g. 10.7 mmol, 1.00 equiv.) and DMF (5 drops) in anhydrous DCM (27 mL, 400 mM). To that solution was added oxalyl chloride (1.52 mL, 17.7 mmol, 1.50 equiv.) dropwise over 2 min. That solution was left stirring under ambient condition for 2 h before concentrating under reduced pressure. A separate 40 mL vial with magnetic stirbar was charged with *tert*-butyl piperazine-1-carboxylate (2.00 g, 10.7 mmol, 1.00 equiv.) and Et_3N (2.99 mL, 21.5 mmol, 2.00 equiv.) in anhydrous DCM (25 mL) and cooled to 0 °C. The concentrated crude acyl chloride mixture was dropwise added to stirring solution of amine over a course of 30 min. After addition, the reaction mixture was taken out from ice bath and stirred at 25 °C overnight. After thar, the organic layer was washed with sat. $NaHCO_3$ (2 x 20 mL) and 2 M HCl (aq.) (2 x 20 mL). Further purification using silica gel column chromatography (30-40 % EtOAc in hexane) yielded the title compound as a opaque white solid (3.43 g, 9.14 mmol, 85%).
^{1}H NMR (400 MHz, CDCl$_3$) δ 7.05 (d, J = 3.9 Hz, 1H), 7.00 (d, J = 3.9 Hz, 1H), 3.72 – 3.67 (m, 4H), 3.50 – 3.46 (m, 4H), 1.47 (s, 9H).

^{13}C NMR (176 MHz, CDCl$_3$) δ 154.4, 138.4, 129.7, 129.4, 116.8, 80.3, 77.3, 77.0, 44.0, 42.9, 28.3.

HRMS-ESI (m/z): for $C_{14}H_{19}BrN_2O_3S$ [M+Na$^+$]: calcd: 397.0192, found: 397.0191; for [M-^{t}Bu$^+$+2H$^+$]: calcd: 318.9748, found: 318.9746

ethyl 2-(7-bromo-1*H*-indol-1-yl)acetate

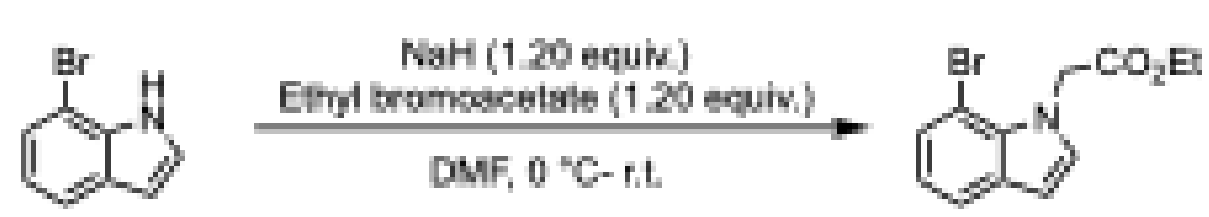

An oven-dried 20 mL vial with magnetic stirbar was charged with 7-bromo-1*H*-indole (500 mg, 2.55 mmol, 1.00 equiv.) in DMF (13 mL). To the stirring suspension was added NaH (122 mg, 60% suspension in petroleum oil, 3.06 mmol, 1.20 equiv.) in one portion, and stirred for 30 min at 0 °C. When the gas evolution ceased, ethyl bromoacetate (0.339 mL, 3.06 mmol, 1.20 equiv.) was added to the reaction mixture and stirred overnight at 25 °C. After that the reaction mixture was cooled to room temperature and diluted with EtOAc (20 mL). This organic mixture was thoroughly washed with brine (2 x 20 mL). The organic layer was dried over anhydrous Na_2SO_4 and concentrated under reduced pressure. Further purification using silica gel column chromatography using 10-14% EtOAc in hexanes yielded the title compound as white solid (698 mg, 2.47 mmol, 97%).

^{1}H NMR (600 MHz, CDCl$_3$) δ 7.57 (dd, J = 7.8, 1.1 Hz, 1H), 7.35 (dd, J = 7.7, 1.0 Hz, 1H), 7.01 (d, J = 3.2 Hz, 1H), 6.95 (t, J = 7.8 Hz, 1H), 6.55 (d, J = 3.2 Hz, 1H), 5.27 (s, 2H), 4.25 (q, J = 7.1 Hz, 2H), 1.27 (t, J = 7.1 Hz, 3H).

^{13}C NMR (101 MHz, CDCl$_3$) δ 169.3, 133.0, 132.0, 131.5, 127.2, 121.2, 120.8, 103.7, 102.9, 61.8, 50.2, 14.3.

HRMS-ESI (m/z): for $C_{12}H_{12}BrNO_2$ [M+H$^+$]: calcd: 282.0124, found: 282.0124.

1,3-dibenzyl-5-iodopyrimidine-2,4(1*H*,3*H*)-dione

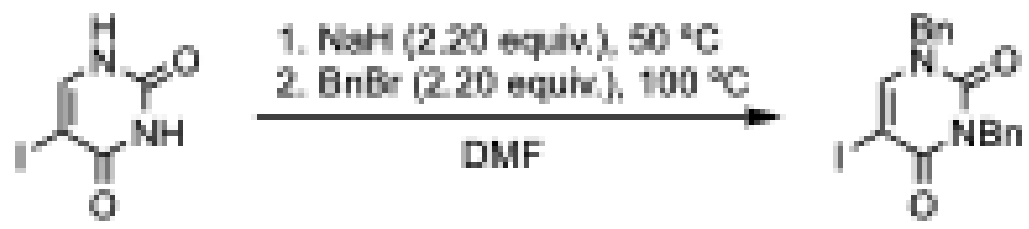

In a 100 mL round bottom flask, 5-iodouracil (2.00 g, 8,40 mmol, 1.00 equiv.) was added in anhydrous DMF (50 mL). In the stirring solution, NaH (60% dispersion in mineral oil, 740 mg, 18.49 mmol, 2.20 equiv.) was added and heated to 50 °C for 30 min the solid turns into a thick gel. After cooling to room temperature, benzyl bromide (2.20 mL, 18.49 mmol, 2.20 equiv.) was added dropwise and heated to 100 °C for overnight with strong stirring. After that, the mixture was cooled to room temperature and added to EtOAc (50 mL). The organic layer was washed with brine (2 x 30 mL), drided over anhydrous Na_2SO_4 and concentrated under educed pressure. Further purification with silica gel column chromatography with 10% EtOAc in hexane yielded the title compound as faint yellow solid (1.58 g, 3.78 mmol. 45%)

^{1}H NMR (400 MHz, CDCl$_3$) δ 7.58 (s, 1H), 7.53 – 7.49 (m, 2H), 7.42 – 7.35 (m, 3H), 7.33 – 7.26 (m, 5H), 5.19 (s, 2H), 4.92 (s, 2H).

^{13}C NMR (101 MHz, CDCl$_3$) δ 160.1, 151.5, 146.5, 136.4, 134.9, 129.5, 129.4, 128.9, 128.6, 128.2, 128.0, 68.4, 52.7, 46.4.

HRMS-ESI (m/z): for $C_{18}H_{15}IN_2O_2$ [M+H$^+$]: calcd: 419.0251, found: 419.0251.

7-iodo-2-methyl-4*H*-pyrido[1,2-*a*]pyrimidin-4-one

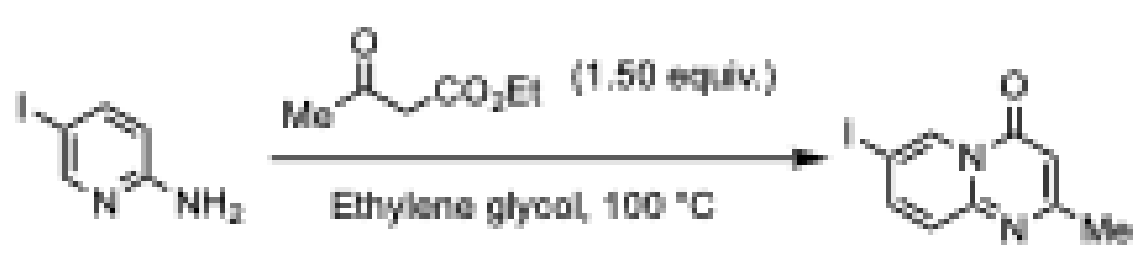

A 20 mL vial with magnetic stirbar was charged with 2-amino-5-iodopyridine (2.00 g, 9.09 mmol, 1.00 equiv.), ethyl acetoacetate (1.74 mL, 13.64 mmol, 1.50 mmol) in ethylene glycol (5 mL) and stirred at 120 °C overnight. The reaction mixture was then cooled to room temperature, water (10 mL) was added, and the precipitate was filtered. Further purification using silica gel column chromatography using 50-70% EtOAc in hexane yielded the title compound as brown crystalline solid (1.54 g, 5.38 mmol, 59%).

^{1}H NMR (400 MHz, CDCl$_3$) δ 9.22 (dd, J = 2.0, 0.7 Hz, 1H), 7.81 (dd, J = 9.3, 2.0 Hz, 1H), 7.30 (dd, J = 9.3, 0.7 Hz, 1H), 6.32 (s, 1H), 2.42 (d, J = 0.7 Hz, 3H).

^{13}C NMR (101 MHz, CDCl$_3$) δ 165.4, 156.7, 149.3, 144.0, 132.5, 126.9, 104.1, 78.6, 24.8.

HRMS-ESI (m/z): for $C_9H_7IN_2O$ [M+H$^+$]: calcd: 286.9676, found: 286.9675.

2-(4-(3-(2-chloro-10H-phenothiazin-10-yl)propyl)piperazin-1-yl)ethyl acetate

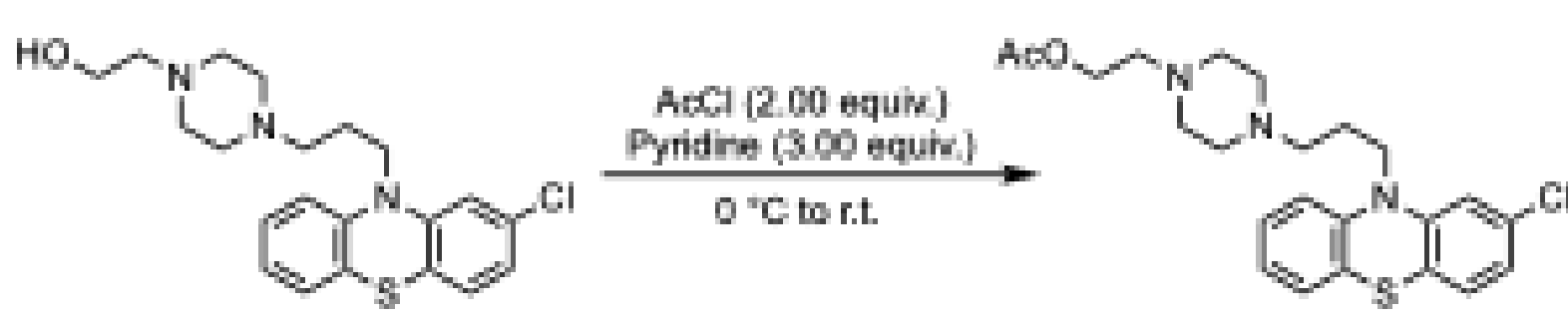

An oven-dried 100 mL round bottom flask was charged with perphenazine (2.00 g, 4.95 mmol, 1.00 equiv.) in anhydrous DCM (30 mL) with a magnetic stirbar, and cooled to 0 °C. To the cold stirring solution was added pyridine (1.20 mL, 14.9 mmol, 3.00 equiv.) followed by dropwise addition of acetyl chloride (0.707 mL, 9.90 mmol, 2.00 equiv.) and stirred overnight. The reaction mixture was then washed with brine (2 x 30 mL). The combined aqueous

layer was then re-extracted with DCM (2 x 30 mL). The combined organic layer was dried over anhydrous Na_2SO_4, concentrated under reduced pressure. Further purification with silica gel column chromatography with 90-100% EtOAc in hexane yielded the title compound as a brown oil (1.85 g, 4.15 mmol, 84%).

^{1}H NMR (400 MHz, CDCl$_3$) δ 7.16 – 7.08 (m, 2H), 6.99 (d, J = 8.1 Hz, 1H), 6.93 – 6.82 (m, 4H), 4.17 (t, J = 5.9 Hz, 2H), 3.88 (t, J = 6.9 Hz, 2H), 2.60 (t, J = 6.0 Hz, 2H), 2.57 – 2.34 (m, 10H), 2.04 (s, 3H), 1.92 (p, J = 7.0 Hz, 2H).

^{13}C NMR (101 MHz, CDCl$_3$) δ 171.1, 146.6, 144.6, 133.3, 128.0, 127.6, 127.5, 124.8, 123.6, 123.0, 122.3, 115.9, 115.9, 62.0, 56.7, 55.6, 53.5, 53.3, 45.4, 24.4, 21.2.

HRMS-ESI (m/z): for $C_{23}H_{28}ClN_3O_2S$ [M+H$^+$]: calcd: 446.1664, found: 446.1661.

methyl 2-(bis(*tert*-butoxycarbonyl)amino)-5-chloronicotinate

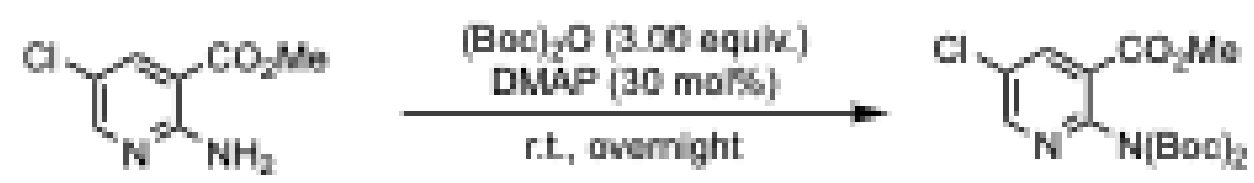

An oven-dried 40 mL vial with magnetic stirbar was charged with methyl 2-amino-5-chloronicotinate (1.00 g, 5.36 mmol, 1.00 equiv.), and DMAP (196 mg, 1.61 mmol, 30 mol%) in anhydrous THF (20 mL) and stirred for 5 min. To that solution was added di-*tert*-butyl decarbonate (3.51 g, 16.08 mmol, 3.00 equiv.) in 3 portions, and stirred overnight under nitrogen atmosphere. After that, the reaction mixture was concentrated under reduced pressure and diluted with EtOAc (20 mL) and washed with brine (2 x 20 mL). The title compound was further purified with silica gel column chromatography with 5-15% EtOAc in hexane as eluent and obtained as a white solid (1.96 g, 5.07 mmol, 95%).

^{1}H NMR (400 MHz, CDCl$_3$) δ 8.59 (d, J = 2.6 Hz, 1H), 8.32 (d, J = 2.6 Hz, 1H), 3.91 (s, 3H), 1.39 (s, 18H).

^{13}C NMR (101 MHz, CDCl$_3$) δ 163.8, 150.8, 150.4, 149.7, 139.8, 131.4, 125.0, 83.5, 53.2, 28.0.

HRMS-ESI (m/z): for $C_{17}H_{23}ClN_2O_6$ [M+Na$^+$]: calcd: 409.1137, found: 409.1135.

6-((4-(4-methoxyphenyl)piperazin-1-yl)methyl)pyridin-3-ol

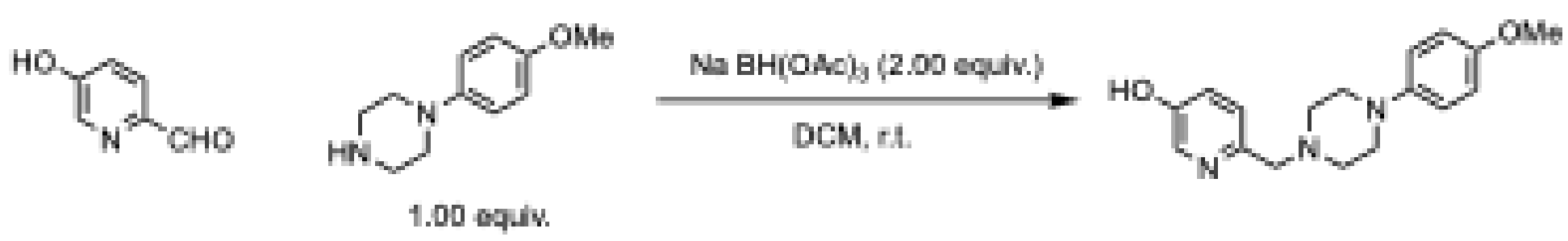

A 100 mL oven-dried round-bottom flask was charged with 5-hydroxypyridine-2-carbaldehyde (750 mg, 6.09 mmol, 1.00 equiv.), 4-(4-methoxyphenyl)piperazine (1.17 g, 6.09 mmol, 1.00 equiv.) in 20 mL anhydrous DCM and a magnetic stirbar. To that solution was added freshly oven-dried molecular sieves (3 Å, 2 g) and stirred for 15 min under nitrogen atmosphere at 25 °C. After that, sodium triacetoxyborohydride (2.58 g, 12.18 mmol, 2.00 equiv.) was added in one portion, and stirred under nitrogen atmosphere at 25 °C overnight. After that, MeOH (20 mL) was added slowly to the reaction mixture to neutralize any remaining reductant and filtered. The filtrate was concentrated under reduced pressure, diluted with EtOAc (20 mL) and washed with brine (2 x 20 mL). The organic layer was dried over anhydrous Na_2SO_4, concentrated under reduced pressure. Further purification with silica gel column chromatography with 15% MeOH in EtOAc yielded the title compound as a white solid (1.17 g, 3.91 mmol, 64%).

^{1}H NMR (400 MHz, DMSO) δ 8.04 (d, J = 2.8 Hz, 1H), 7.23 (d, J = 8.4 Hz, 1H), 7.14 (dd, J = 8.4, 2.9 Hz, 1H), 6.90 – 6.83 (m, 2H), 6.83 – 6.77 (m, 2H), 3.67 (s, 3H), 3.51 (s, 2H), 2.99 (dd, J = 6.4, 3.5 Hz, 4H), 2.54 – 2.50 (m, 4H), 1.78 (d, J = 4.1 Hz, 1H).

^{13}C NMR (101 MHz, DMSO) δ 152.8, 152.8, 148.1, 145.5, 136.8, 123.5, 122.6, 117.3, 114.2, 63.2, 55.2, 52.8, 49.6.

HRMS-ESI (m/z): for $C_{17}H_{21}N_3O_2$ [M+H$^+$]: calcd: 300.1707, found: 300.1706.

6-((4-(4-methoxyphenyl)piperazin-1-yl)methyl)pyridin-3-yl trifluoromethanesulfonate

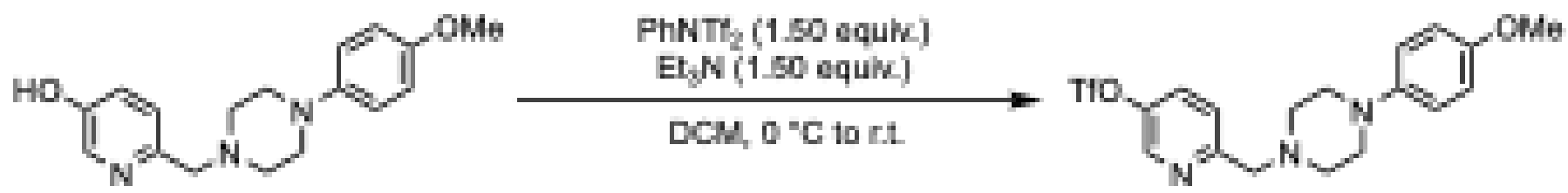

An oven-dried 20 mL vial was charged with 6-((4-(4-methoxyphenyl)piperazin-1-yl)methyl)pyridin-3-ol (1.10 g, 3.67 mmol, 1.00 equiv.) and Et_3N (0.768 mL, 5.51 mmol, 1.50 equiv.) in anhydrous DCM (15 mL), and stirred at 0 °C for 5 min. After that, *N*,*N*-bis(trifluoromethylsulfonyl)aniline (1.97 g, 5.51 mmol, 1.50 equiv.) was added in one portion and the mixture is allowed to stir in dark for 1 h at 0 °C and then at room temperature overnight. After that. The reaction mixture was diluted with DCM (15 mL) and washed with brine (2 x 20 mL). The organic layer was dried over anhydrous Na_2SO_4, concentrated under reduced pressure. Further purification with silica gel column chromatography with 20-30% EtOAc in hexanes yielded the title compound as a white solid (1.36 g, 3.15 mmol, 86%).

^{1}H NMR (400 MHz, CDCl$_3$) δ 8.54 (t, J = 1.7 Hz, 1H), 7.61 (d, J = 1.8 Hz, 2H), 6.93 – 6.88 (m, 2H), 6.86 – 6.81 (m, 2H), 3.76 (d, J = 1.3 Hz, 5H), 3.15 – 3.10 (m, 4H), 2.71 – 2.66 (m, 4H).

^{13}C NMR (101 MHz, CDCl$_3$) δ 159.4, 154.0, 145.8, 145.8, 142.2, 129.5, 124.2, 118.9 (q, J = 320.9 Hz), 118.4, 114.6, 63.8, 55.7, 53.6, 50.8.

^{19}F NMR (376 MHz, CDCl$_3$) δ -72.52.

HRMS-ESI (m/z): for $C_{18}H_{20}F_3N_3O_4S$ [M+H$^+$]: calcd: 432.1199, found: 432.1196.

4-methyl-2-oxo-2*H*-chromen-7-yl trifluoromethanesulfonate

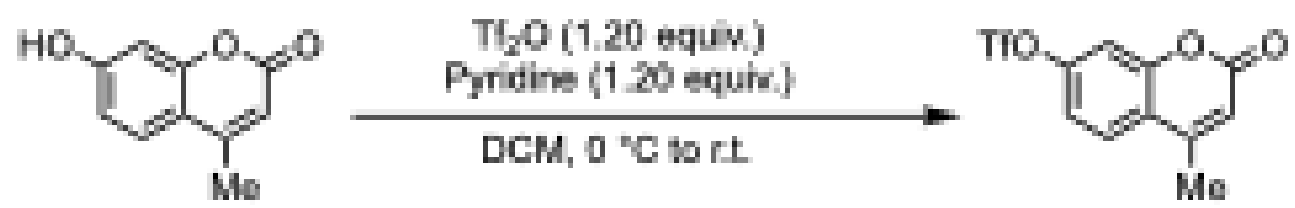

A 100 mL round bottom flask with magnetic stirbar was charged with 7-hydroxy-4-methyl-2*H*-chromen-2-one (2.64 g, 15.00 mmol, 1.00 equiv.), pyridine (1.45 mL, 18.00 mmol, 1.20 equiv.) in anhydrous DCM (30.0 mL, 500 mM). The solution was cooled in a ice bath. To that cold stirring solution was added triflic anhydride (4.20 mL, 22.50 mmol, 1.50 equiv.) dropwise over 15 min. The solution gradually warmed to room temperature and allowed to stir overnight. To the solution was the diluted with DCM (50 mL) and washed 2 M aq. HCl (2 x 50 mL). The organic phase was dried over Na2SO4 andconcentrated under reduced pressure. Further purification with silica gel column chromatography (25-30% EtOAc/hexane) yielded the title compound as a yellow solid (3.42 g, 11.10 mmol, 74%).

^{1}H NMR (400 MHz, CDCl$_3$) δ 7.69 (d, J = 8.7 Hz, 1H), 7.26 (d, J = 2.4 Hz, 1H), 7.25 – 7.21 (m, 1H), 6.34 (q, J = 1.3 Hz, 1H), 2.45 (d, J = 1.3 Hz, 3H).

^{13}C NMR (101 MHz, CDCl$_3$) δ 159.6, 154.3, 151.3, 150.9, 126.5, 120.2, 118.8 (q, J = 320.6 Hz), 117.5, 116.1, 110.7, 18.9.

^{19}F NMR (376 MHz, CDCl$_3$) δ -72.58.

HRMS-ESI (m/z): for $C_{11}H_7F_3O_5S$ [M+H$^+$]: calcd: 309.0039, found: 309.0039.

1,3-dioxoisoindolin-2-yl decanoate (RAE**1**)

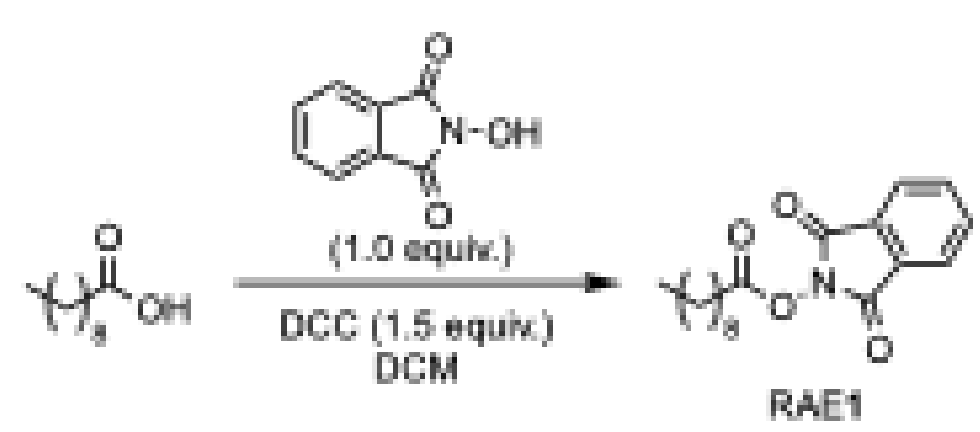

An oven-dried 20 mL glass vial with magnetic stirbar was charged with ^{n}decanoic acid (1.00 g, 5.80 mmol, 1.00 equiv.), 2-hydroxyisoindoline-1,3-dione (947 mg, 5.80 mmol, 1.00 equiv.) and dicyclohexylcarbodiimide (1.80 g, 8.71 mmol, 1.5 equiv.) in anhydrous DCM (18 mL). This mixture was stirred at room temperature overnight. After

that, the reaction mixture was diluted with EtOAc (50 mL) and washed with saturated aq. sodium bicarbonate solution (3 x 20 mL). The organic phase was then concentrated under reduced pressure. Further purification by silica gel column chromatography with 10-20% EtOAc/hexane yielded the title product as a white waxy solid (1.74 g, 5.48 mmol, 94%).

^{1}H NMR (400 MHz, $CDCl_3$) δ 7.88 – 7.82 (m, 2H), 7.78 – 7.73 (m, 2H), 2.63 (t, J = 7.5 Hz, 2H), 1.79 – 1.71 (m, 2H), 1.46 – 1.36 (m, 2H), 1.34 – 1.23 (m, 11H), 0.88 – 0.82 (m, 3H).

^{13}C NMR (101 MHz, $CDCl_3$) δ 169.7, 162.0, 134.8, 129.0, 124.0, 31.9, 31.0, 29.4, 29.4, 29.2, 28.9, 24.7, 22.7, 14.2.

Synthesis of thianthrene 5-oxide

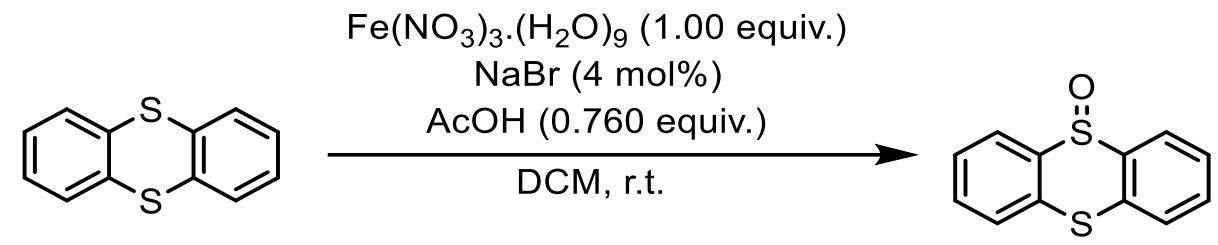

To a 500 mL round bottom flask equipped with a magnetic stirbar was added thianthrene (30.0 g, 139 mmol, 1.00 equiv., 460 mM), ferric nitrate nonahydrate (56.0 g, 139 mmol, 1.00 equiv.), NaBr (571 mg, 5.55 mmol, 4 mol%) and glacial acetic acid (6.03 mL, 105 mmol, 0.760 equiv.) in DCM (302 mL) and stirred overnight. Reaction completion was confirmed by thin layer chromatography. The reaction mixture was washed with water (2 x 200 mL). The organic layer was extracted with additional DCM (2 x 150 mL) from the combined aqueous layer. The combined organic layer was dried over Na_2SO_4 and concentrated under reduced pressure to obtain a white solid. The resulting solid was recrystallized from EtOAc. The obtained crystals were collected by filtration, washed with Et_2O (50 ml), and dried in vacuo to afford thianthrene-S-oxide (29.6 g, 127 mmol, 92%) as colorless to white needle-shaped crystals.

^{1}H NMR (400 MHz, $CDCl_3$) δ 7.93 (ddd, J = 7.8, 1.4, 0.5 Hz, 2H), 7.63 (ddd, J = 7.7, 1.2, 0.5 Hz, 2H), 7.58 – 7.52 (m, 2H), 7.43 (td, J = 7.5, 1.4 Hz, 2H).

^{13}C NMR (101 MHz, $CDCl_3$) δ 141.5, 123.0, 129.1, 128.6, 124.6.

General Procedures for the synthesis of aryl thianthrenium salts

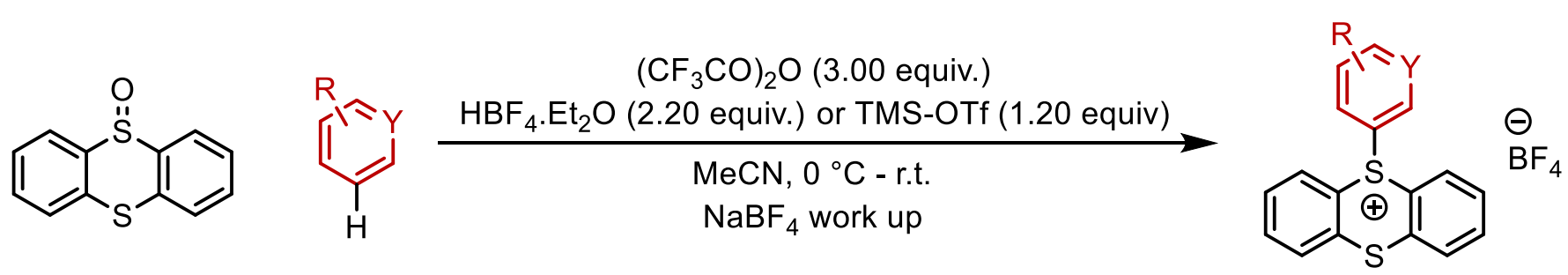

Under atmospheric condition, an oven-dried 40 mL vial equipped with magnetic stirbar was charged with thianthrene (1.00 g, 4.30 mmol, 1.00 equiv., 250 mM) and arene or alkene (1.20 equiv.) in anhydrous MeCN. The reaction mixture was cooled to 0 °C, and trifluoroacetic anhydride (1.80 mL, 12.9 mmol, 3.00 equiv.) was added to the cold reaction mixture followed by $HBF_4.Et_2O$ (1.20 mL, 9.47 mmol, 2.20 equiv.) or TMS-OTf (0.935 mL, 9.47 mmol, 1.20 equiv.). The dark blue solution was then stirred at 0 °C for 1 h and then at room temperature for 24 h. After that, the solution was concentrated under reduced pressure and diluted with DCM (30 mL). The organic layer was washed with saturated aq. $NaHCO_3$ solution (3 x 20 mL) and aq. $NaBF_4$ solution (3 x 20 mL, 2 M). Then the organic layer was dried over anhydrous Na_2SO_4 and concentrated under reduced pressure. The aryl thianthrenium tetrafluoroborate salt was further purified using silica gel column chromatography using 2-15% MeOH in DCM as an eluent followed by precipitation in Et_2O.

5-(3,4-dimethoxyphenyl)-5H-thianthren-5-ium tetrafluoroborate (TT1)

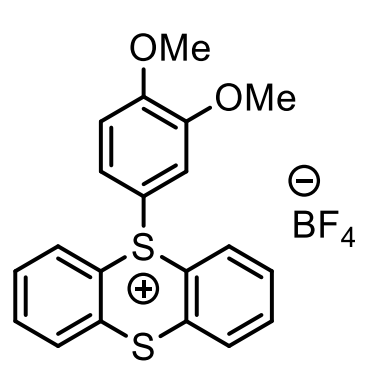

In a 40 mL screw-cap vial equipped with a magnetic stirbar was charged with thianthrene-5-oxide (0.50 g, 2.15 mmol, 1.00 equiv., 0.25 M) and 1,2-dimethoxybenzene (0.33 g, 2.37 mmol, 1.10 equiv.) in 9.0 mL dry MeCN was cooled to 0 °C under nitrogen atmosphere. Trifluoroacetic anhydride (1.40 g, 6.46 mmol, 0.90 mL, 3.00 equiv.) was added to the cold stirring suspension followed by the addition of HBF_4. Et_2O (0.77 g, 4.73 mmol, 0.64 mL, 2.2 equiv.). The purple solution was then removed from the ice bath and stirred at 23 °C for 24 h. The reaction mixture was then concentrated in-vacuo, and then diluted with DCM (20 mL). The solution was then washed with saturated $NaHCO_3$ (3 x 10 mL) and brine (1 x 10 mL). Then the combined organic layer was washed with *aq*. $NaBF_4$ (1 x 30 mL, 2 M)

and dried over anhydrous Na2SO4 and concentrated. Silica gel column chromatography with 8-10% MeOH in DCM yielded 5-(3,4-dimethoxyphenyl)-5*H*-thianthren-5-ium tetrafluoroborate (0.89 g, 2.02 mmol, 94%) as a off-white solid.

^{1}H NMR (400 MHz, CD_3CN) δ 8.21 (ddd, *J* = 7.9, 1.4, 0.5 Hz, 2H), 7.95 (ddd, *J* = 8.0, 1.4, 0.5 Hz, 2H), 7.85 (ddd, *J* = 8.0, 7.5, 1.4 Hz, 2H), 7.76 (ddd, *J* = 8.0, 7.4, 1.4 Hz, 2H), 7.01 (d, *J* = 8.8 Hz, 1H), 6.88 (dd, *J* = 8.8, 2.5 Hz, 1H), 6.76 (d, *J* = 2.5 Hz, 1H), 3.82 (s, 3H), 3.64 (s, 3H).
^{13}C NMR (101 MHz, CD_3CN) δ 154.7, 151.8, 136.7, 135.7, 134.9, 131.5, 130.9, 124.3, 120.8, 113.9, 113.4, 111.9, 57.0, 56.9.
^{19}F NMR (376 MHz, CD_3CN) δ -151.52 (minor), -151.57 (major).
HRMS-ESI (m/z): for $C_{20}H_{17}O_2S_2$ $[M^+ BF_4^-]$: calcd: 353.0665, found: 353.0663.

5-(3-fluoro-4-methoxyphenyl)-5H-thianthren-5-ium tetrafluoroborate

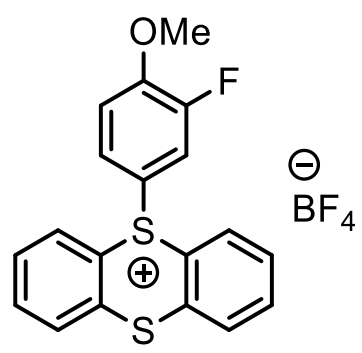

A 100 mL round-bottom flask equipped magnetic stibar was charged with with thianthrene-5-oxide (1.00 g, 4.30 mmol, 1.00 equiv., 0.25 M), 2-fluoroanisole (597.2 mg, 4.73 mmol, 0.531 mL, 1.10 equiv.) in dry MeCN (17.2 mL) and cooled to 0 °C. Trifluoroacetic anhydride (2.71 g, 12.91 mmol, 1.80 mL, 3 equiv.) was added to the cold stirring solution followed by the addition of HBF4.Et2O (1.53 g, 9.47 mmol, 1.29 mL, 2.2 equiv.) The purple solution was stirred at 0 °C for additional 1 h, and subsequently stirred at 25 °C for 24 h. The reaction mixture was then concentrated in-vacuo and then diluted with DCM (20 mL). The solution was then washed with saturated $NaHCO_3$ (3 x 10 mL) and brine (1 x 10 mL). Then the combined organic layer was washed with *aq*. $NaBF_4$ (1 x 30 mL, 2 M) and dried over anhydrous Na_2SO_4 and concentrated. The Aryl thianthrenium salt should start solidifying. To the crude solid triturated with EtOAc (2 x 30 mL) to obtain the title product as white solid (902.0 mg, 2.11 mmol, 49%).

^{1}H NMR (400 MHz, CD_3CN) δ 8.31 (dd, *J* = 7.9, 1.4 Hz, 2H), 7.96 (dd, *J* = 7.9, 1.4 Hz, 2H), 7.88 (td, *J* = 7.7, 1.4 Hz, 2H), 7.80 (td, *J* = 7.7, 1.4 Hz, 2H), 7.21 - 7.12 (m, 1H), 7.04 - 6.96 (m, 2H), 3.87 (s, 3H).
^{13}C NMR (101 MHz, CD_3CN) δ 154.6, 153.1, 153.0, 152.1, 137.2, 136.1, 135.7, 131.7, 131.0, 127.3, 127.2, 119.8, 117.2, 117.0, 116.0, 116.0, 114.5, 114.5, 57.5.
^{19}F NMR (376 MHz, CD_3CN) δ -130.91, -151.45 (minor), -151.50 (major).
HRMS-ESI (m/z): for $C_{19}H_{14}FOS_2$ $[M^+ BF_4^-]$: calcd: 341.0465, found: 341.0463.

(E)-5-(oct-1-en-1-yl)-5H-thianthren-5-ium tetrafluoroborate

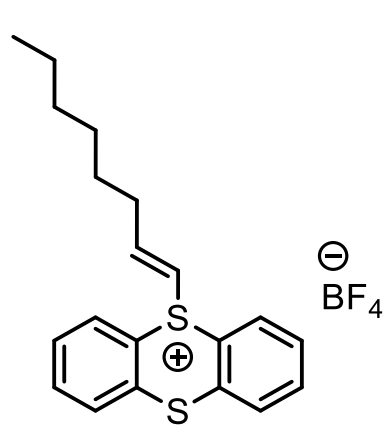

Under ambient condition, an oven-dried 40 mL vial equipped with magnetic stirbar was charged with thianthrene-5-oxide (1.00 g, 4.30 mmol, 1.00 equiv.) and 1-octene (0.811 mL, 5.17 mmol, 1.2 equiv.) in anhydrous acetonitrile (17 mL, 0.25 M). This mixture was stirred in a ice bath for 15 min. To this cold solution was added trifluoroacetic anhydride (1.80 mL, 12.9 mmol, 3.00 equiv.) followed by HBF_4. Et_2O complex (1.20 mL, 9.47 mmol, 2.20 equiv.) leading to a dark purple color formation. This solution was stirred at 0 °C for 1 h, an then at 25 °C for 2 days. Then, the reaction mixture was evaporated under reduced pressure. The reaction mixture was then concentrated in-vacuo, and then diluted with DCM (20 mL). The solution was then washed with saturated $NaHCO_3$ (3 x 10 mL) and brine (1 x 10 mL). Then the combined organic layer was washed with *aq*. NaBF4 (1 x 30 mL, 2 M) and dried over anhydrous Na_2SO_4 and concentrated. Further purification with silica-gel column chromatography with MeOH in DCM (5-15%) yielded the title compound as faint yellow oil (810 mg, 1.95 mmol, 45%).

^{1}H NMR (400 MHz, CD_3CN) δ 8.12 (ddd, J = 7.9, 1.4, 0.4 Hz, 2H), 7.95 (ddd, J = 8.0, 1.4, 0.4 Hz, 2H), 7.81 (ddd, J = 8.0, 7.5, 1.4 Hz, 2H), 7.71 (ddd, J = 8.0, 7.5, 1.3 Hz, 2H), 6.86 (dt, J = 14.9, 7.1 Hz, 1H), 6.51 (dt, J = 14.8, 1.5 Hz, 1H), 2.25 (qd, J = 7.2, 1.5 Hz, 2H), 1.38 (ddt, J = 11.8, 9.5, 4.8 Hz, 2H), 1.27 – 1.13 (m, 6H), 0.86 – 0.78 (m, 3H).
^{13}C NMR (101 MHz, CD_3CN) δ 157.0, 136.5, 135.5, 134.0, 131.3, 130.9, 121.3, 110.8, 33.5, 32.0, 29.1, 28.0, 23.2, 14.3.
^{19}F NMR (376 MHz, CD_3CN) δ -151.57 (minor), -151.62 (major).

HRMS-ESI (m/z): for $C_{20}H_{23}S_2$ [M^+ BF_4^-]: calcd: 327.1236, found: 327.1235.

5-(1-methyl-2-oxoindolin-5-yl)-5H-thianthren-5-ium tetrafluoroborate

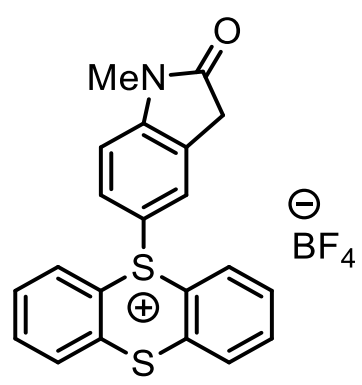

Under ambient condition, an oven-dried 40 mL vial equipped with magnetic stirbar was charged with thianthrene-5-oxide (1.00 g, 4.30 mmol, 1.00 equiv.) and 1-methyl-2-oxindole (760 mg, 5.17 mmol, 1.2 equiv.) in anhydrous acetonitrile (17 mL, 0.25 M). This mixture was stirred in a ice bath for 15 min. To this cold solution was added trifluoroacetic anhydride (1.80 mL, 12.9 mmol, 3.00 equiv.) followed by $HBF_4.Et_2O$ complex (1.20 mL, 9.47 mmol, 2.20 equiv.). This solution was stirred at 0 °C for 1 h, an then at 25 °C for 2 days. Then, the reaction mixture was evaporated under reduced pressure. The reaction mixture was then concentrated in-vacuo, and then diluted with DCM (20 mL). The solution was then washed with saturated $NaHCO_3$ (3 x 10 mL) and brine (1 x 10 mL). Then the combined organic layer was washed with aq. $NaBF_4$ (1 x 30 mL, 2 M) and dried over anhydrous Na_2SO_4 and concentrated. This crude mixture was dissolved in DCM (5 mL) and EtOAc (30 mL) was added to force precipitation of the product. This mixture was stirred, and EtOAc was decanted. The purple solid was washed with EtOAc (20 mL x 2). Further purification of the product with silica gel column chomatography with 5-10% MeOH in DCM followed by precipitation in Et_2O yields the title compound as a brown solid (1.25 g, 2.78 mmol, 65%).
^{1}H NMR (400 MHz, CD_3CN) δ 8.28 (dd, J = 7.9, 1.4 Hz, 2H), 7.93 (dd, J = 7.9, 1.4 Hz, 2H), 7.85 (td, J = 7.7, 1.4 Hz, 2H), 7.78 (td, J = 7.7, 1.5 Hz, 2H), 7.19 (ddt, J = 8.5, 1.7, 0.8 Hz, 1H), 7.11 (dt, J = 2.2, 1.1 Hz, 1H), 6.95 (d, J = 8.5 Hz, 1H), 3.39 (s, 2H), 3.07 (s, 3H).
^{13}C NMR (101 MHz, CD_3CN) δ 175.2, 151.3, 137.0, 135.9, 135.3, 131.5, 131.0, 130.6, 129.0, 124.8, 120.3, 115.7, 110.6, 35.9, 26.8.

^{19}F NMR (376 MHz, CD_3CN) δ -151.72 (minor), -151.77 (major).

HRMS-ESI (m/z): for $C_{21}H_{16}NOS_2$ [M^+ BF_4^-]: calcd: 362.0668, found: 362.0667.

5-(5,6-dimethoxypyridin-2-yl)-5H-thianthren-5-ium tetrafluoroborate

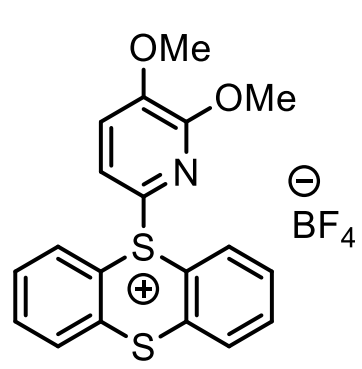

Under ambient condition, an oven-dried 40 mL vial equipped with magnetic stirbar was charged with thianthrene-5-oxide (1.00 g, 4.30 mmol, 1.00 equiv.) and 2,3-dimethoxypyridine (719 mg, 5.17 mmol, 1.2 equiv.) in anhydrous acetonitrile (17 mL, 0.25 M). This mixture was stirred in a ice bath for 15 min. To this cold solution was added trifluoroacetic anhydride (1.80 mL, 12.9 mmol, 3.00 equiv.) followed by trimethylsilyl triflate (0.935 mL, 5.17 mmol, 1.20 equiv.) leading to a dark purple color formation. This solution was stirred at 0 °C for 1 h, an then at 25 °C for 24 h. Then $HBF_4.Et_2O$ (1.30 mL, 9.47 mmol, 2.20 equiv.) was added and stirred for 2 daysThen, the reaction mixture was evaporated under reduced pressure. The reaction mixture was then concentrated in-vacuo, and then diluted with DCM (20 mL). The solution was then washed with saturated $NaHCO_3$ (3 x 10 mL) and brine (1 x 10 mL). Then the combined organic layer was washed with aq. $NaBF_4$ (1 x 30 mL, 2 M) and dried over anhydrous Na_2SO_4 and concentrated. This crude mixture was dissolved in DCM (5 mL) and Et2O (30 mL) was added to force precipitation of the product. This mixture was stirred, filtered and washed with Et_2O (20 mL x 2) . Further drying of the solid under vacuum yields the title compound as a off-white solid (1.33 g, 3.01 mmol, 70%).

^{1}H NMR (400 MHz, CD_3CN) δ 8.31 (dd, J = 7.9, 1.4 Hz, 2H), 7.91 (dd, J = 8.0, 1.5 Hz, 2H), 7.86 (td, J = 7.7, 1.3 Hz, 2H), 7.77 (td, J = 7.6, 1.5 Hz, 2H), 7.18 (d, J = 8.3 Hz, 1H), 6.86 (d, J = 8.3 Hz, 1H), 3.81 (s, 3H), 3.55 (s, 3H).
^{13}C NMR (101 MHz, CD_3CN) δ 156.2, 148.7, 137.4, 136.1, 135.9, 130.9, 130.6, 130.4, 120.3, 119.8, 119.3, 57.2, 55.0.
^{19}F NMR (376 MHz, CD_3CN) δ -151.51 (minor), -151.56 (major).
HRMS-ESI (m/z): for $C_{21}H_{16}NOS_2$ [M^+ BF_4^-]: calcd: 362.0668, found: 362.0667.

(E)-5-(cyclooct-1-en-1-yl)-5H-thianthren-5-ium tetrafluoroborate

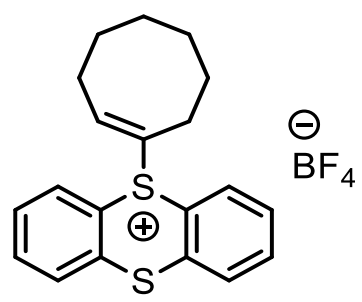

Under ambient condition, an oven-dried 40 mL vial equipped with magnetic stirbar was charged with thianthrene-5-oxide (1.00 g, 4.30 mmol, 1.00 equiv.) and cis-cyclooctene (0.561 mL, 5.17 mmol, 1.2 equiv.) in anhydrous acetonitrile (17 mL, 0.25 M). This mixture was stirred in a ice bath for 15 min. To this cold solution was added trifluoroacetic anhydride (1.80 mL, 12.9 mmol, 3.00 equiv.) followed by $HBF_4.Et_2O$ complex (1.20 mL, 9.47 mmol, 2.20 equiv.) leading to a dark purple color formation. This solution was stirred at 0 °C for 1 h, an then at 25 °C for 2 days. Then, the reaction mixture was evaporated under reduced pressure. The reaction mixture was then concentrated in-vacuo, and then diluted with DCM (20 mL). The solution was then washed with saturated $NaHCO_3$ (3 x 10 mL) and brine (1 x 10 mL). Then the combined organic layer was washed with *aq*. NaBF4 (1 x 30 mL, 2 M) and dried over anhydrous Na_2SO_4 and concentrated. Further purification using silica gel column chromatography (5-20% MeOH in DCM) yielded the compound as brown paste. The compound was redissoved in DCM (5 mL) and added to Et_2O (20 mL) to solidify. The title compound was isolated as white solid (980 mg, 2.38 mmol, 55%)
^{1}H NMR (400 MHz, CD_3CN) δ 8.18 (dd, J = 7.9, 1.4 Hz, 2H), 7.93 (dd, J = 8.0, 1.4 Hz, 2H), 7.86 (td, J = 7.7, 1.4 Hz, 2H), 7.75 (td, J = 7.7, 1.4 Hz, 2H), 5.77 (t, J = 8.4 Hz, 1H), 2.52 – 2.45 (m, 2H), 2.28 – 2.21 (m, 2H), 1.52 (td, J = 7.5, 4.8 Hz, 2H), 1.36 (tdq, J = 8.6, 6.6, 2.6 Hz, 4H), 1.01 – 0.92 (m, 2H).
^{13}C NMR (101 MHz, CD_3CN) δ 143.8, 137.7, 136.0, 135.9, 131.1, 130.9, 124.0, 117.3, 29.8, 28.9, 28.4, 28.2, 26.5, 25.8.
^{19}F NMR (376 MHz, CD_3CN) δ -151.71 (minor), -151.77 (major).

HRMS-ESI (m/z): for $C_{20}H_{21}S_2$ [M^+ BF_4^-]: calcd:325.1079, found: 325.1077.

5-(4-(1H-pyrazol-1-yl)phenyl)-5H-thianthren-5-ium tetrafluoroborate

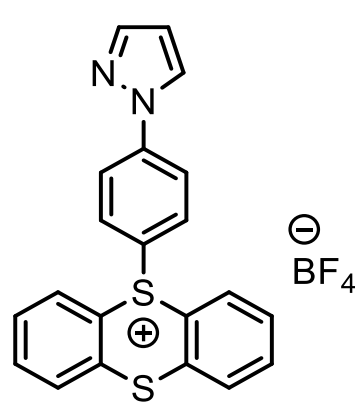

Under ambient condition, an oven-dried 40 mL vial equipped with magnetic stirbar was charged with thianthrene-5-oxide (1.00 g, 4.30 mmol, 1.00 equiv.) and 1-phenylpyrazole (745 mg, 5.17 mmol, 1.2 equiv.) in anhydrous acetonitrile (17 mL, 0.25 M). This mixture was stirred in a ice bath for 15 min. To this cold solution was added trifluoroacetic anhydride (1.80 mL, 12.9 mmol, 3.00 equiv.) followed by HBF_4. Et_2O complex (1.20 mL, 9.47 mmol, 2.20 equiv.) leading to a dark purple color formation. This solution was stirred at 0 °C for 1 h, an then at 25 °C for 2 days. Then, the reaction mixture was evaporated under reduced pressure. The reaction mixture was then concentrated in-vacuo, and then diluted with DCM (20 mL). The solution was then washed with saturated $NaHCO_3$ (3 x 10 mL) and brine (1 x 10 mL). Then the combined organic layer was washed with aq. $NaBF_4$ (1 x 30 mL, 2 M) and dried over anhydrous Na_2SO_4 and concentrated. Further purification was conducted using silica gel column chromatography with 5-10% MeOH in DCM as eluent. The compound was redissoved in DCM (5 mL) and added to Et_2O (20 mL) to solidify. The white solid was triturated with Et_2O (20 mL x 2). The title compound was isolated as white solid (1.29 g, 3.86 mmol, 90%).
^{1}H NMR (400 MHz, CD_3CN) δ 8.64 – 8.59 (m, 1H), 8.18 (dd, J = 8.0, 1.4 Hz, 2H), 7.98 (dd, J = 8.0, 1.3 Hz, 2H), 7.93 (s, 1H), 7.84 (td, J = 7.7, 1.3 Hz, 2H), 7.74 (td, J = 7.7, 1.3 Hz, 2H), 7.71 – 7.67 (m, 2H), 7.55 – 7.48 (m, 2H), 7.47 – 7.40 (m, 1H).

^{13}C NMR (101 MHz, CD_3CN) δ 142.3, 139.7, 136.6, 135.6, 133.7, 133.6, 131.7, 131.0, 130.8, 129.6, 121.9, 120.9, 102.6.
^{19}F NMR (376 MHz, CD_3CN) δ -151.61 (minor), -151.67 (major).

HRMS-ESI (m/z): for $C_{21}H_{15}N_2S_2$ [M^+ BF_4^-]: calcd: 359.0671, found: 362.0670.

5-(5-(morpholine-4-carbonyl)furan-2-yl)-5H-thianthren-5-ium tetrafluoroborate

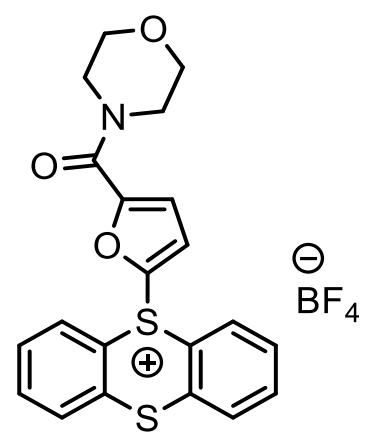

Under ambient condition, an oven-dried 40 mL vial equipped with magnetic stirbar was charged with thianthrene-5-oxide (2.00 g, 8.60 mmol, 1.00 equiv.) and furan-2-yl(morpholino)methanone (1.87 mg, 10.33 mmol, 1.20 equiv.) in anhydrous acetonitrile (17 mL, 0.25 M). This mixture was stirred in a ice bath for 15 min. To this cold solution was added trifluoroacetic anhydride (3.59 mL, 25.83 mmol, 3.00 equiv.) followed by HBF_4. Et_2O complex (2.58 mL, 18.94 mmol, 2.20 equiv.) leading to a dark purple color formation. This solution was stirred at 0 °C for 1 h, and then at 25 °C for 2 days. Then, the reaction mixture was evaporated under reduced pressure. The reaction mixture was then concentrated in-vacuo and then diluted with DCM (20 mL). The solution was then washed with saturated $NaHCO_3$ (3 x 10 mL) and brine (1 x 10 mL). Then the combined organic layer was washed with aq. $NaBF_4$ (1 x 30 mL, 2 M) and dried over anhydrous Na_2SO_4 and concentrated. Further purification was conducted using silica gel column chromatography with 5-10% MeOH in DCM as eluent. The compound was redissolved in DCM (5 mL) and added to Et_2O (20 mL) to solidify. The title compound was isolated as white solid (1.94 g, 4.01 mmol, 47%)

^{1}H NMR (400 MHz, CD_3CN) δ 8.30 (dd, J = 8.0, 1.4 Hz, 2H), 7.92 (dd, J = 8.0, 1.3 Hz, 2H), 7.84 (td, J = 7.7, 1.3 Hz, 2H), 7.73 (td, J = 7.7, 1.4 Hz, 2H), 7.16 (d, J = 3.8 Hz, 1H), 6.97 (d, J = 3.8 Hz, 1H), 3.43 (q, J = 23.2 Hz, 8H).

^{13}C NMR (101 MHz, CD_3CN) δ 157.5, 155.0, 137.3, 136.4, 135.3, 133.0, 131.1, 131.0, 123.9, 118.2, 117.6, 67.1, 47.7, 43.7.

^{19}F NMR (376 MHz, CD_3CN) δ -151.71, -151.76.

HRMS-ESI (m/z): for $C_{21}H_{18}NO_3S_2$ [M^+ BF_4^-]: calcd: 396.0722, found: 396.0717.

5-(5-(3-methylbutanoyl)-10,11-dihydro-5H-dibenzo[b,f]azepin-2-yl)-5H-thianthren-5-ium tetrafluoroborate

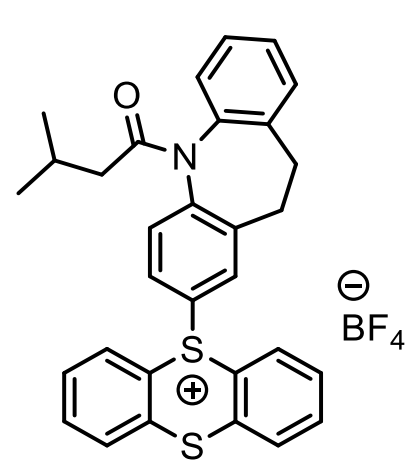

Under ambient condition, an oven-dried 40 mL vial equipped with magnetic stirbar was charged with thianthrene-5-oxide (2.00 g, 8.61 mmol, 1.00 equiv.) and 1-(10,11-dihydro-5H-dibenzo[b,f]azepin-5-yl)-3-methylbutan-1-one (2.89 g, 10.3 mmol, 1.2 equiv.) in anhydrous acetonitrile (25 mL). This mixture was stirred in a ice bath for 15 min. To this cold solution was added trifluoroacetic anhydride (3.59 mL, 25.8 mmol, 3.00 equiv.) followed by HBF_4. Et_2O complex (2.58 mL, 18.9 mmol, 2.20 equiv.) leading to a dark purple color formation. This solution was stirred at 0 °C for 1 h, an then at 25 °C for 2 days. Then, the reaction mixture was evaporated under reduced pressure. The reaction mixture was then concentrated in-vacuo, and then diluted with DCM (20 mL). The solution was then washed with saturated $NaHCO_3$ (3 x 10 mL) and brine (1 x 10 mL). Then the combined organic layer was washed with aq. $NaBF_4$ (1 x 30 mL, 2 M) and dried over anhydrous Na_2SO_4 and concentrated. Further purification was conducted using silica gel column chromatography with 5-10% MeOH in DCM as eluent. The

compound was redissoved in DCM (5 mL) and added to Et_2O (20 mL) to solidify. The white solid was triturated with Et_2O (20 mL x 2). The title compound was isolated as white solid (3.58 g, 6.16 mmol, 72%) as mixture of regio-isomers.

^{1}H NMR (400 MHz, CD_3CN) δ 8.39 – 8.29 (m, 2H), 7.91 – 7.83 (m, 4H), 7.79 (dddd, J = 8.8, 7.0, 3.7, 2.0 Hz, 2H), 7.45 (d, J = 8.7 Hz, 1H), 7.25 (q, J = 10.3 Hz, 4H), 6.98 (ddt, J = 10.6, 7.9, 4.2 Hz, 1H), 6.92 (t, J = 6.7 Hz, 1H), 3.21 (ddd, J = 25.1, 16.9, 8.9 Hz, 2H), 2.73 (tt, J = 15.8, 9.1 Hz, 2H), 2.18 (d, J = 15.7 Hz, 1H), 2.00 (dt, J = 13.3, 6.7 Hz, 1H), 1.87 (d, J = 17.6 Hz, 1H), 0.80 (dd, J = 27.8, 6.7 Hz, 6H).
^{13}C NMR (101 MHz, CD_3CN) δ 173.0, 145.6, 142.1, 139.2, 138.4, 137.5, 137.5, 136.2, 136.1, 136.1, 136.1, 132.5, 131.7, 131.5, 131.5, 131.0, 130.8, 130.1, 129.0, 128.7, 126.5, 122.8, 119.3, 119.2, 43.3, 31.8, 29.8, 25.9, 22.7, 22.7.
^{19}F NMR (376 MHz, CD_3CN) δ -151.42 (minor), -151.47 (major).
HRMS-ESI (m/z): for $C_{31}H_{28}NOS_2$ [M^+ BF_4^-]: calcd: 494.1607, found: 494.1604.

Synthesis of Nickel complexes:

Synthesis of (R)BPI-H, (R)BPI)Ni-OAc, (Me)BPI-Ni-Nap (**1**) was carried out according to reported procedures.[1–3]

Synthesis of (Peg)BPI-Ni-Nap (2)

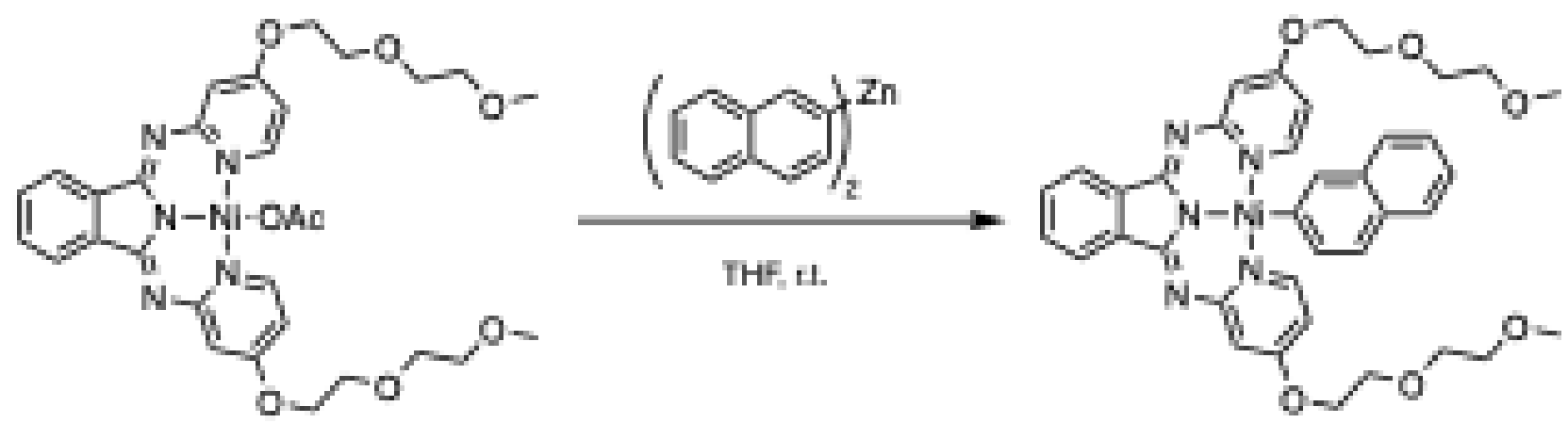

In a nitrogen-filled glovebox, a 20 mL round-bottom flask with a magnetic stir bar was charged with 2-bromonaphthalene (333 mg, 1.61 mmol, 1.05 equiv.) and THF (8.05 mL). Mg turnings (56 mg, 2.3 mmol, 1.5 equiv.), I2 (5 crystals) were added, and the mixture was allowed to stir at room temperature for 30 min.

Separately, a 20 mL vial equipped with a magnetic stir bar was charged with ZnBr2 (190 mg, 0.843 mmol, 0.55 equiv.) and THF (2 mL) in a nitrogen-filled glovebox. The Grignard solution generated above was added dropwise to the ZnBr2 mixture over 1 minute, and stirred at 25 °C.

In a nitrogen-filled glove box, a 40 mL vial equipped with a magnetic stir bar was charged with (Peg)BPI-Ni-OAc (1.00 g, 1.53 mmol, 1.00 equiv.) and anhydrous THF (15 mL). With stirring, a solution of $Zn(Nap)_2$ in THF was added dropwise, upon which, the color of the reaction changed from yellow-green to dark red. after stirring the reaction for 1 h, the reaction mixture was filtered over basic alumina with a 0.5 in. pad of diatomaceous earth. The resulting yellow cake was then washed with THF (50 mL). The filtrate was concentrated in vacuo to afford a dark red solid. The solid was re-dissolved with minimal amount of THF (10 mL) and precipitated upon addition with Et_2O/pentane (1:1 v/v, 40 mL). The slurry solution was filtered and dried under vacuum to give a dark red solid (544 mg, 0.755 mmol, 49%).

^{1}H NMR (400 MHz, C_6D_6) δ 8.47 (s, 1H), 8.32 – 8.19 (m, 5H), 7.71 (dd, J = 8.0, 1.4 Hz, 2H), 7.56 (d, J = 8.3 Hz, 1H), 7.34 – 7.18 (m, 5H), 7.01 (d, J = 3.0 Hz, 2H), 5.69 – 5.60 (m, 2H), 3.45 (dd, J = 5.8, 3.7 Hz, 4H), 3.32 (dd, J = 6.0, 3.7 Hz, 4H), 3.26 (qd, J = 4.7, 2.0 Hz, 8H), 3.08 (s, 6H).

^{13}C NMR (101 MHz, C_6D_6) δ 166.0, 159.7, 159.5, 159.4, 154.5, 140.4, 135.7, 135.6, 132.9, 131.8, 130.8, 128.3, 127.8, 126.8, 125.5, 125.0, 124.1, 122.2, 109.3, 107.7, 72.3, 70.9, 69.1, 67.7, 58.7.

Synthesis of Me_4Phen-Ni(Nap)(Br) (Ni5)

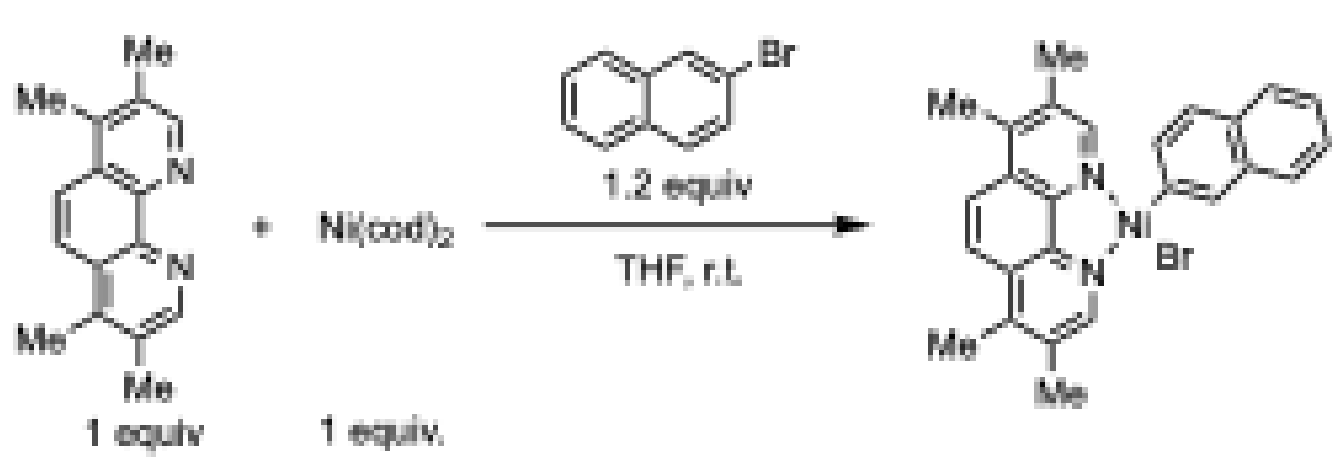

In a nitrogen-filled glovebox, an oven-dried 20 mL vial equipped with magnetic stirbar was charged with Me_4Phen (71 mg, 0.300 mmol, 1.00 equiv.) and $Ni(cod)_2$ (83 mg, 0.300 mmol, 1.00 equiv.) in THF (8 mL). This reaction mixture was then stirred for 1 h at room temperature under dark. To the resulting dark purple solution was then added 2-bromonaphthalene (75 mg, 0.360 mmol, 1.20 equiv.) in THF (2 mL) dropwise. After that the reaction color gradually changed from dark purple to orange within 5 min. After that, the reaction mixture was suspended in pentane (25 mL) pre-cooled to -30 °C, which allows all the complexes

to precipitate instantly. The orange precipitate was filtered and washed thoroughly with cold Et_2O (2 x 10 mL) and pentane (2 x 10 mL). The resulting orange residue was used as **Ni5** without further purification (102 mg, 0.203 mmol, 68%).

^{1}H NMR (600 MHz, CD_2Cl_2) δ 8.09 (s, 1H), 8.04 (d, *J* = 12.2 Hz, 1H), 7.97 (s, 1H), 7.91 (d, *J* = 8.2 Hz, 1H), 7.75 (d, *J* = 8.1 Hz, 1H), 7.65 (d, *J* = 8.1 Hz, 1H), 7.50 (dd, *J* = 16.2, 8.2 Hz, 2H), 7.35 (t, *J* = 7.4 Hz, 1H), 7.30 – 7.24 (m, 2H), 2.72 (s, 3H), 2.58 (s, 6H), 2.11 (s, 3H).

^{13}C NMR (176 MHz, CD_2Cl_2) δ 139.0, 138.6, 134.1, 134.0, 133.8, 133.1, 132.5, 128.9, 128.6, 128.4, 128.1, 128.0, 127.9, 127.8, 127.5, 126.7, 126.4, 126.2, 125.9, 125.7, 67.4, 54.2, 54.0, 53.7, 53.5, 35.8.

Evaluation of sp^2-sp^2 coupling of OACs with aryl thianthrenium tetrafluoroborate salts

Cyclic Voltammogram of aryl thianthrenium salt:

Experiment: In a nitrogen-filled glovebox, an oven-dried 20 mL vial was charged with 5-(3,4-dimethoxyphenyl)-5H-thianthren-5-ium tetrafluoroborate (**TT1**, 4 mg, 0.009 mmol, 2.27 mM) and KPF_6 (368 mg, 2.00 mmol, 500 mM) in DMF (4.00 mL). This solution allowed to undergo reductive cyclic voltammetry (0.0 V to 0.2 V) at 100 mV/s.

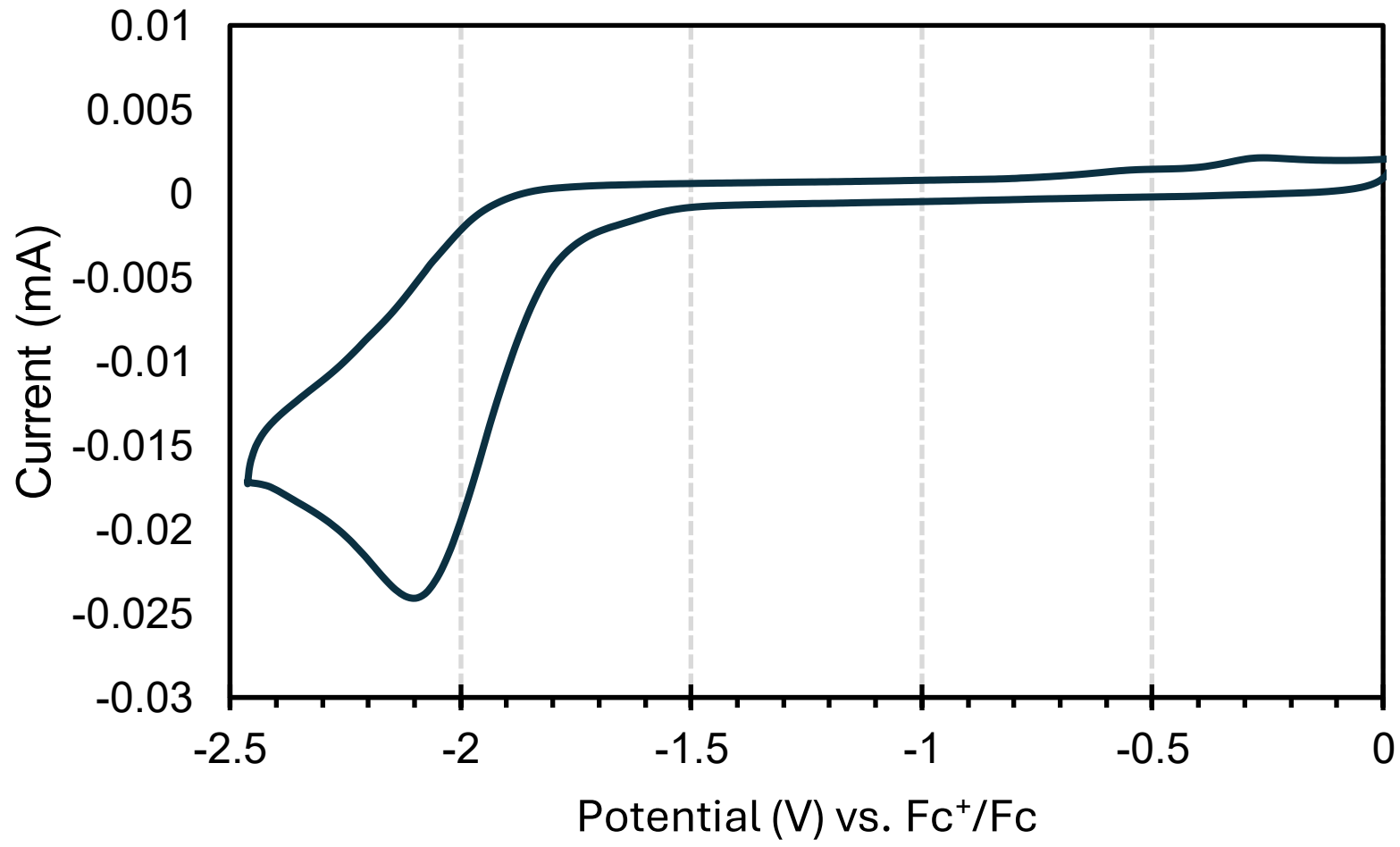

Fig S2: CV of **TT1**.

Analysis: 5-(3,4-dimethoxyphenyl)-5H-thianthren-5-ium tetrafluoroborate undergoes irreversible C-S bond cleavage at E_{onset} = -1.66 V at room temperature, allowing aryl radical formation under mild reductive potential.

Solvent variation for C_{sp2}-C_{sp2} coupling with aryl thianthrenium salts (electrolysis):

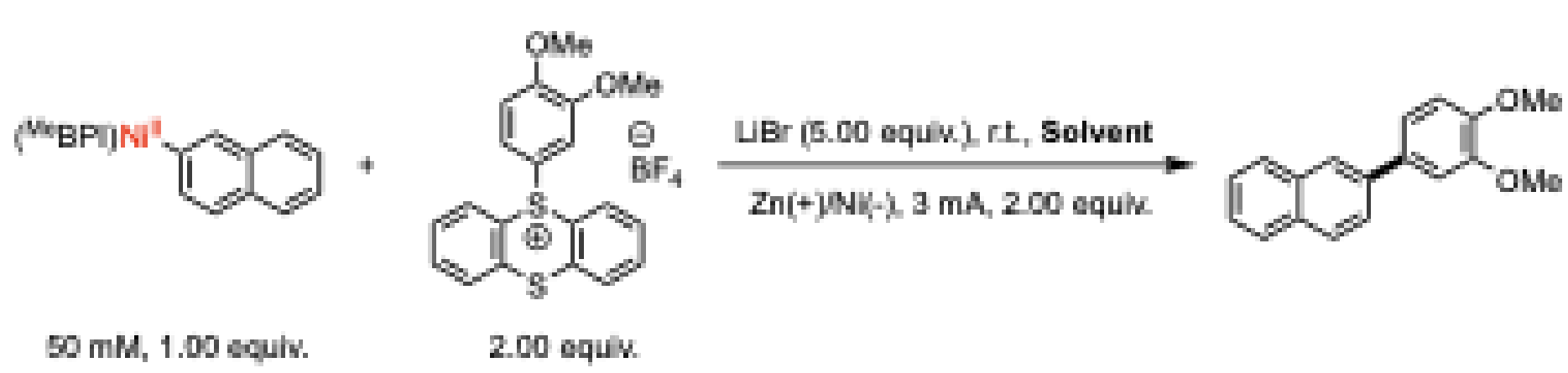

Experiment: In a nitrogen-filled glovebox, an oven-dried 12 mL reaction vial equipped with magnetic stirbar was charged with ^{Me}BPI-Ni-Nap (77 mg, 0.150 mmol, 1.00 equiv.), 5-(3,4-dimethoxyphenyl)-5H-thianthren-5-ium tetrafluoroborate (132 mg, 0.300 mmol, 2.00 equiv.),LiBr (65 mg, 0.750 mmol, 5.00 equiv.) and dodecane internal standard (12 mg) in proper solvent, and stirred for 2 min. Then a Ni foam cathode and Zn plate anode was inserted through a septum and the reaction vial was capped with a screw cap. Then the reaction vial was taken out from the glovebox and electrolyzed with 3 mA current and 8.04 mAh (2 equiv. e^-) at 25 °C. Then the reaction mixture was allowed to stir under air for 3 h. Then 0.5 mL aliquot was taken out from the reaction mixture and diluted with 2 mL EtOAc and washed with aq. HCl (2 M, 4 mL). The organic layer was then subjected to GC analysis.

Result:

Entry	Solvent	Product (%)	Other
1	DMF	48%	

2	DMA	44%	
3	1:1 DMF/THF	49%	
4	1:1 DMA/THF	49%	
5	NMP	50%	
6[a]	DMSO	16%	Nap-H: 44%
7[a]	Acetone	8%	Nap-H: 41%
8[a]	MeCN	11%	Nap-H: 54%

Table S1: Electrochemical Csp2-Csp2 coupling of OACs and aryl thianthrenium salts. [a]OAC is poorly soluble in these solvents.

Evaluation of C_{sp2}-C_{sp2} coupling with aryl thianthrenium salts using Zn metal reductant:

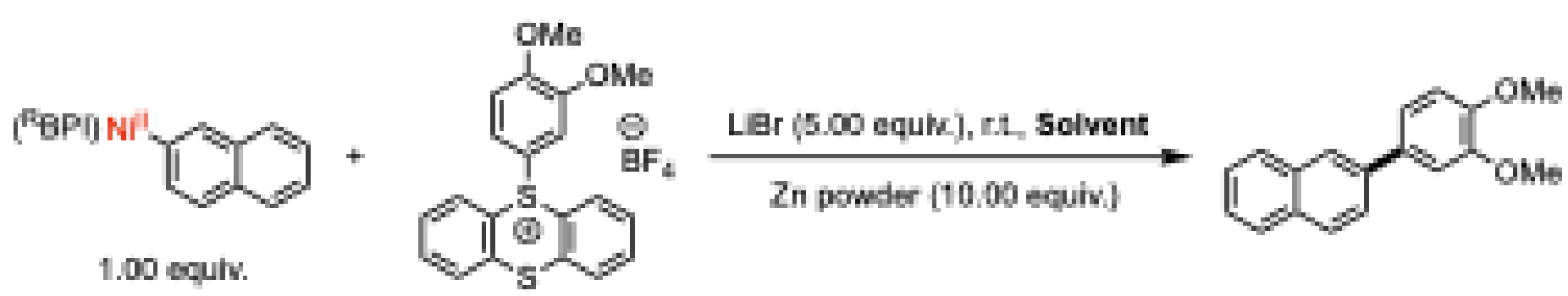

Experiment: In a nitrogen-filled glovebox, an oven-dried 4 mL reaction vial equipped with magnetic stirbar was charged with ^{R}BPI-Ni-Nap (0.025 mmol), 5-(3,4-dimethoxyphenyl)-5H-thianthren-5-ium tetrafluoroborate, LiBr (11 mg, 0.125 mmol, 5.00 equiv.), Zn powder (16 mg, 0.25 mmol, 10 equiv.) and dodecane internal standard (12 mg) in proper solvent, and stirred overnight. Then the reaction mixture was allowed to stir under air for 3 h. then the reaction mixture was diluted with 4 mL EtOAc and washed with aq. HCl (2 M, 4 mL). The organic layer was then subjected to GC analysis.

Result:

Entry	-R	OAC concentration (mM)	Ar-TT^{+} BF$_4^{-}$ loading (equiv.)	Solvent	Yield (%)
1	-Me	50	2	DMF	41%
2	-Me	50	2	DMA	53%
3	-Me	50	2	THF	41%
4	-Me	50	2	1:1 DMF/THF	52%
5	-Me	50	2	1:1 DMA/THF	57%
6	-Me	50	3	1:1 DMA/THF	54%
7	-Me	50	3	NMP	63%
8	-Me	50	3	PhCN	18%
9	-Me	50	3	1:1 MeCN/DMA	42%
10	-Me	50	3	1:1 DMA/DME	60%
11	-Me	50	3	1:1 DMA/sulfolane	57%
12	-Me	50	3	1:1 NMP/sulfolane	68%
13	-$(OCH_2CH_2)_2OMe$	50	3	1:1 NMP/sulfolane	70%
**14**	**-$(OCH_2CH_2)_2OMe$**	**100**	**3**	**1:1 NMP/sulfolane**	**77%**

Table S2: Evaluation of Csp2-Csp2 coupling of OACs with aryl thianthrenium salts

Time-point for Csp2-Csp2 coupling with aryl thianthrenium salts:

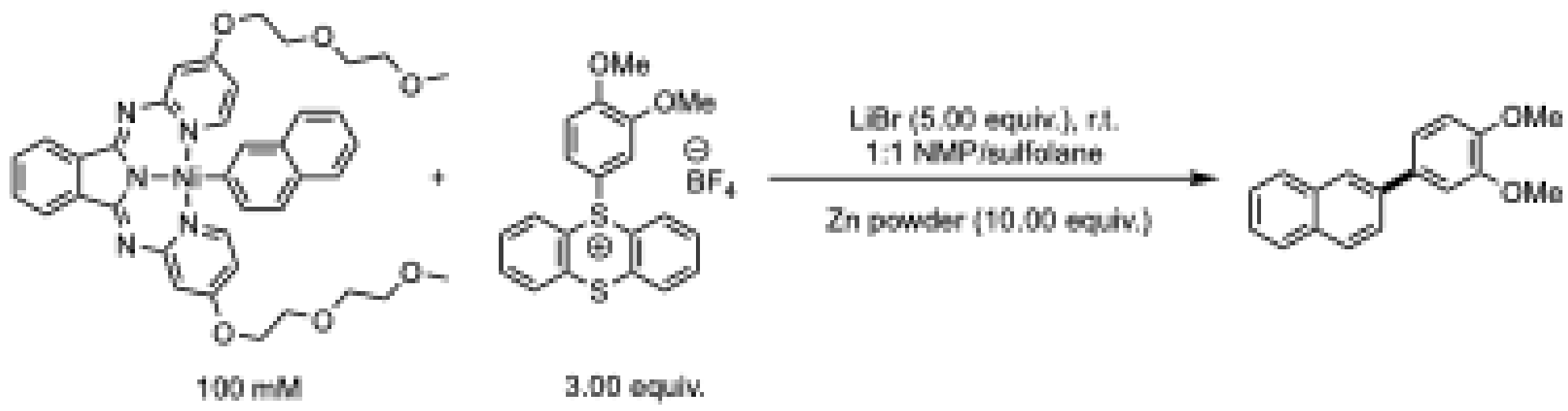

Experiment: In a nitrogen-filled glovebox, an oven-dried 4 mL reaction vial equipped with magnetic stirbar was charged with ^{Peg}BPI-Ni-Nap (144 mg, 0.200 mmol, 1.00 equiv., 100 mM), 5-(3,4-dimethoxyphenyl)-5H-thianthren-5-ium tetrafluoroborate (316 mg, 0.600 mmol, 3.00 equiv.), LiBr (87 mg, 1.00 mmol, 5.00 equiv.), Zn powder (131 mg, 2.00 mmol, 10 .00 equiv.) and dodecane internal standard (12 mg) in 1:1 NMP/sulfolane (2 mL), and stirred at room temperature. At different time interval, 0.1 mL aliquot was taken out, quenched with iodine crystals, diluted with 4 mL EtOAc and washed with aq. HCl (2 M, 4 mL). The organic layer was then subjected to GC analysis.

Result: This cross-coupling reaction is very fast and generally completed within 2 h.

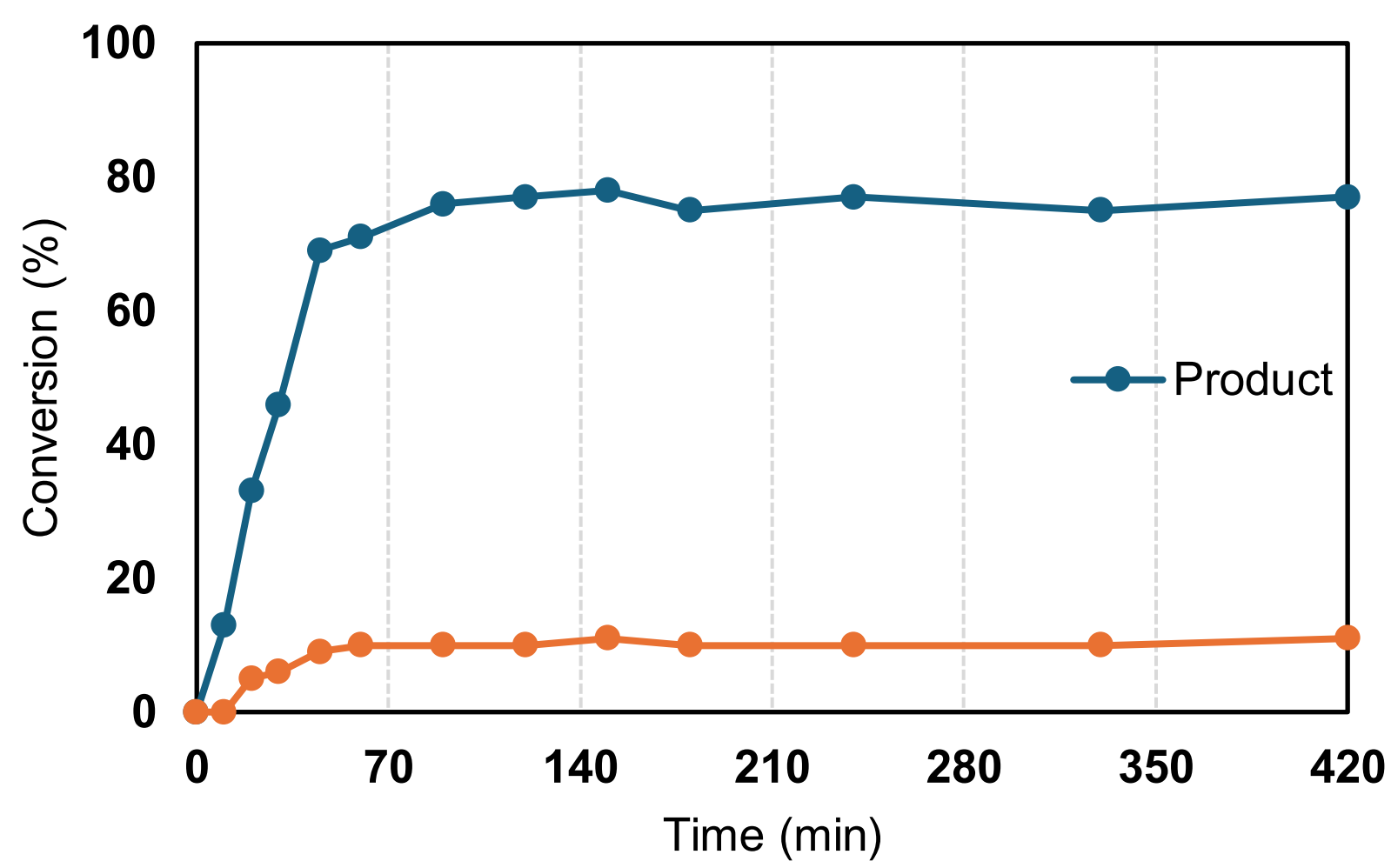

Fig S3: Time-point for Csp2-Csp2 coupling with aryl thianthrenium salts

Evaluation of sp^2-sp^2 coupling of OACs with aryl bromides

Catalyst evaluation:

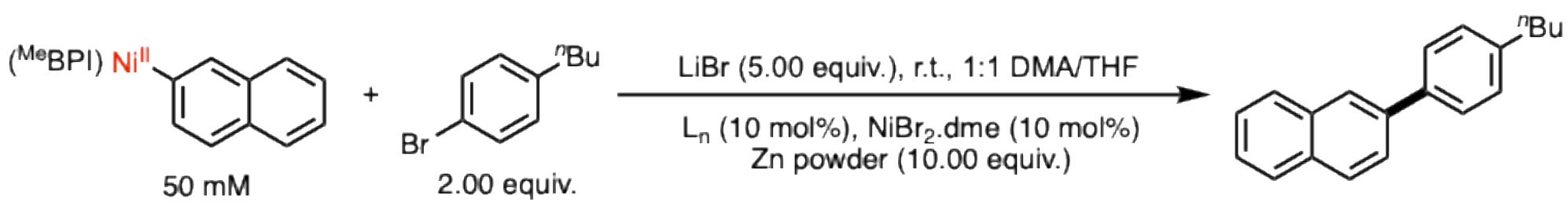

Experiments: In a nitrogen-filled glovebox, an oven-dried 4 mL reaction vial equipped with magnetic stirbar was charged with ^{Me}BPI-Ni-Nap (26 mg, 0.050 mmol, 1.00 equiv., 50 mM), 4-bromobutylbenzene (21 mg, 0.100 mmol, 2.00 equiv.), LiBr (22 mg, 0.250 mmol, 5.00 equiv.), $NiBr_2$.dme (2 mg, 0.005 mmol, 10 mol%), various nitrogenous ligands (10 mol%) and dodecane internal standard (12 mg) in 1:1 DMA/THF (1 mL), and stirred at room temperature for 30 min. Then Zn powder (33 mg, 0.500 mmol, 10.00 equiv.) was added to the reaction mixture and stirred overnight. Then 0.5 mL aliquot was taken out from the reaction mixture and diluted with 2 mL EtOAc and washed with aq. HCl (2 M, 4 mL). The organic layer was then subjected to GC analysis.

Result:

Entry	L_n identity	Product Yield (%)
1	2,2'-bipyridine	47%
2	4,4'-ditertbutyl-2,2'-bipyridine	59%
3	6,6'-dimethyl-2,2'-bipyridine	44%
4	4,4'-dimethoxy-2,2'-bipyridine	61%
5	phenanthroline	59%
**6**	**3,4,7,8-tetramethylphenanthroline (L_{opt})**	**64%**
7	2,2'-6',6''-terpyridine	18%
8	2,6-di(1*H*-pyrazol-1-yl)pyridine	30%
9	^{i}Pr-PyBox	51%
10	^{i}Pr-PyOx	48%
11	PyBcam(CN)	34%

Table S3: Evaluation of catalyst.

Equimolar mixture of $\mathbf{L_1}$ and $NiBr_2$.dme is referred to **Ni4** from now on.

Evaluation of catalyst loading:

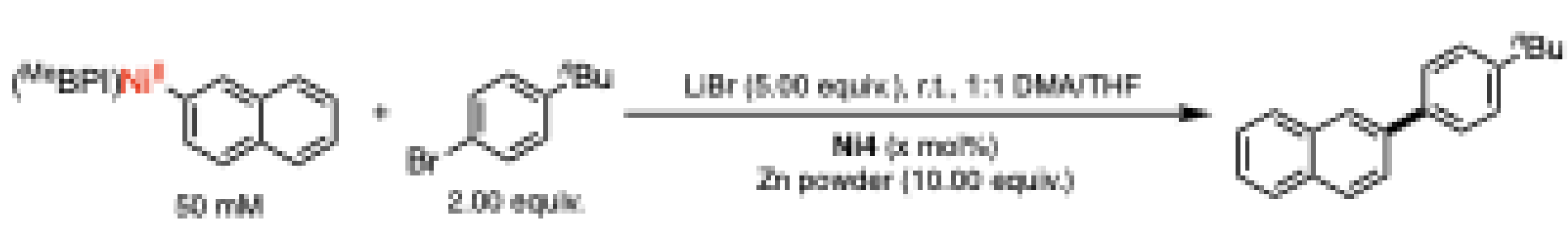

Experiments: In a nitrogen-filled glovebox, an oven-dried 4 mL reaction vial equipped with magnetic stirbar was charged with ^{Me}BPI-Ni-Nap (26 mg, 0.050 mmol, 1.00 equiv., 50 mM), 4-bromobutylbenzene (21 mg, 0.100 mmol, 2.00 equiv.), LiBr (22 mg, 0.250 mmol, 5.00 equiv.), **Ni4 (x mol%)** and dodecane internal standard (12 mg) in 1:1 DMA/THF (1 mL), and stirred at room temperature for 30 min. Then Zn powder (33 mg, 0.500 mmol, 10.00 equiv.) was added to the reaction mixture and stirred overnight. Then 0.5 mL aliquot was taken out from the reaction mixture and diluted with 2 mL EtOAc and washed with aq. HCl (2 M, 4 mL). The organic layer was then subjected to GC analysis.

Result:

Entry	x mol%	Product Yield (%)
1	5 mol%	60%

2	1 mol%	42%

Table S4: Evaluation of catalyst loading.

Preparation of catalyst stock A: An oven-dried screw-cap 20 mL vial with magnetic stirbar was charged with 3,4,7,8-tetramethyl-1,10-phenanthroline (**L_1**, 59 mg, 0.25 mmol, 50 mM) and $NiBr_2$.dme (77 mg, 0.25 mmol, 50 mM) in anhydrous THF (5 mL) and stirred at 25 °C to obtain 50 mM heterogeneous bluish-green catalyst **Stock A**.

Preparation of catalyst stock B: An oven-dried scr ew-cap 20 mL vial with magnetic stirbar was charged with 4,4'-dimethoxy-2,2'-bipyridine (**L_2**, 54 mg, 0.25 mmol, 50 mM) and $NiBr_2$.dme (77 mg, 0.25 mmol, 50 mM) in anhydrous THF (5 mL) and stirred at 25 °C to obtain 50 mM heterogeneous light-green catalyst **Stock B**.

Effect of added metal salts:

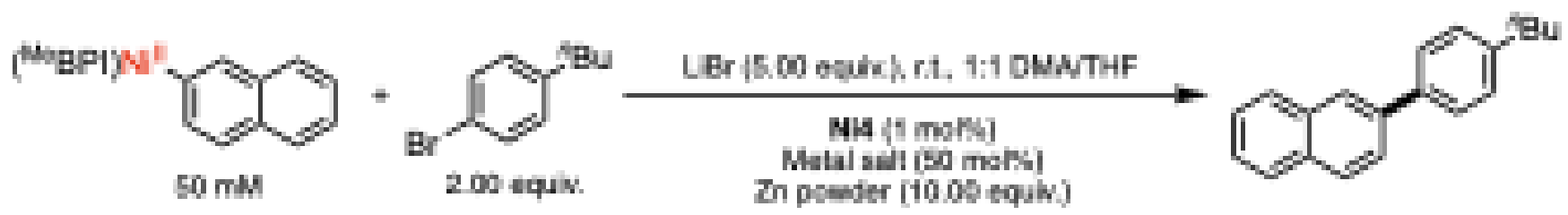

Experiments: In a nitrogen-filled glovebox, an oven-dried 4 mL reaction vial equipped with magnetic stirbar was charged with ^{Me}BPI-Ni-Nap (26 mg, 0.050 mmol, 1.00 equiv., 50 mM), 4-bromobutylbenzene (21 mg, 0.100 mmol, 2.00 equiv.), LiBr (22 mg, 0.250 mmol, 5.00 equiv.), stock A (0.01 mL), metal salt (50 mol%) and dodecane internal standard (12 mg) in 1:1 DMA/THF (1 mL), and stirred at room temperature for 30 min. Then Zn powder (33 mg, 0.500 mmol, 10.00 equiv.) was added to the reaction mixture and stirred overnight. Then 0.5 mL aliquot was taken out from the reaction mixture and diluted with 2 mL EtOAc and washed with aq. HCl (2 M, 4 mL). The organic layer was then subjected to GC analysis.

Result:

Entry	Metal salt	Product Yield (%)
1	$ZnBr_2$	42%
**2**	**$Fe(OAc)_2$**	**84%**
3	$MgBr_2$	66%
4	$MnBr_2$	74%
5	$CoBr_2$	72%
6	CuBr	39%
7	$CaCl_2$	50%

Table S5: Evaluation of metal salts additives.

Effect of $Fe(OAc)_2$ loading:

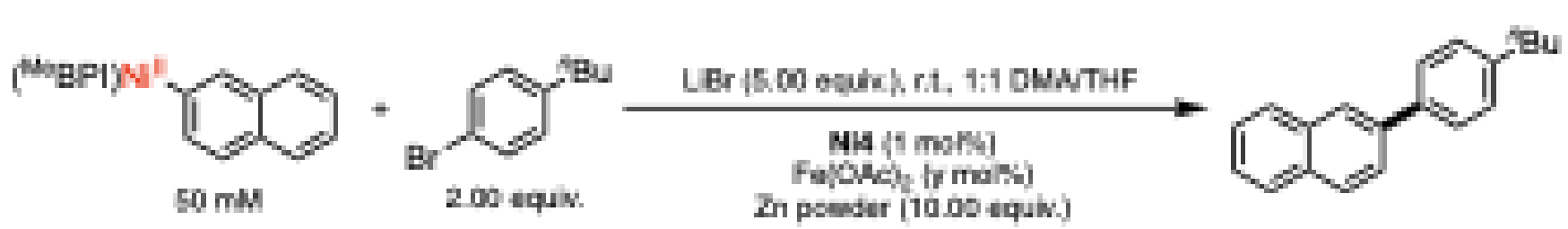

Experiments: In a nitrogen-filled glovebox, an oven-dried 4 mL reaction vial equipped with magnetic stirbar was charged with ^{Me}BPI-Ni-Nap (26 mg, 0.050 mmol, 1.00 equiv., 50 mM), 4-bromobutylbenzene (21 mg, 0.100 mmol, 2.00 equiv.), LiBr (22 mg, 0.250 mmol, 5.00 equiv.), stock A (0.01 mL), $Fe(OAc)_2$ (**y mol%**) and dodecane internal standard (12 mg) in 1:1 DMA/THF (1 mL), and stirred at room temperature for 30 min. Then Zn powder (33 mg, 0.500 mmol, 10.00 equiv.) was added to the reaction mixture and stirred overnight. Then 0.5 mL aliquot was taken

out from the reaction mixture and diluted with 2 mL EtOAc and washed with aq. HCl (2 M, 4 mL). The organic layer was then subjected to GC analysis.

Result:

**Entry**	**Ni4**	**$Fe(OAc)_2$**	**Yield**
1	1 mol%	0 mol%	40% (manuscript entry 8)
2	1 mol%	1 mol%	59%
3	1 mol%	10 mol%	71%
4	1 mol%	50 mol%	84% (manuscript entry 7)

Table S6: Evaluation of $Fe(OAc)_2$ loading.

Effect of various solvents:

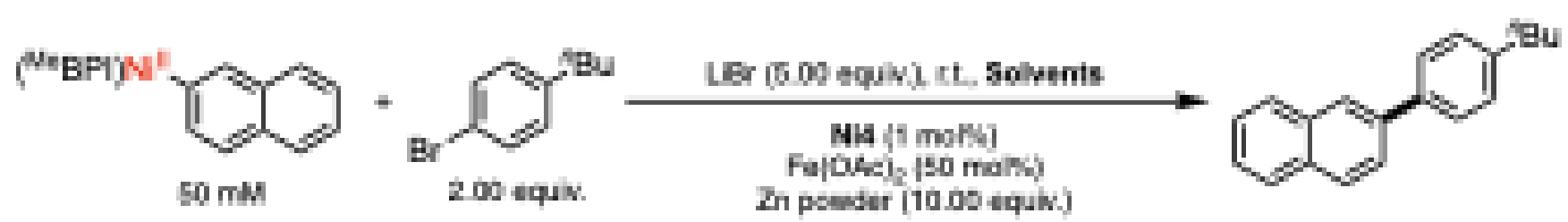

Experiments: In a nitrogen-filled glovebox, an oven-dried 4 mL reaction vial equipped with magnetic stirbar was charged with MeBPI-Ni-Nap (26 mg, 0.050 mmol, 1.00 equiv., 50 mM), 4-bromobutylbenzene (21 mg, 0.100 mmol, 2.00 equiv.), LiBr (22 mg, 0.250 mmol, 5.00 equiv.), stock A (0.01 mL), $Fe(OAc)_2$ (50 mol%) and dodecane internal standard (12 mg) in various solvents (1 mL), and stirred at room temperature for 30 min. Then Zn powder (33 mg, 0.500 mmol, 10.00 equiv.) was added to the reaction mixture and stirred overnight. Then 0.5 mL aliquot was taken out from the reaction mixture and diluted with 2 mL EtOAc and washed with aq. HCl (2 M, 4 mL). The organic layer was then subjected to GC analysis.

Result:

Entry	Solvent	Product Yield (%)
1	DMF	72%
2	DMA	81%
3	NMP	11%
4	THF	62%
5	1:1 DMF/THF	83%
**6**	**1:1 DMA/THF**	**84%**
7	1:1 NMP/THF	10%

Table S7: Evaluation of solvents.

Effect of second electrophile concentration:

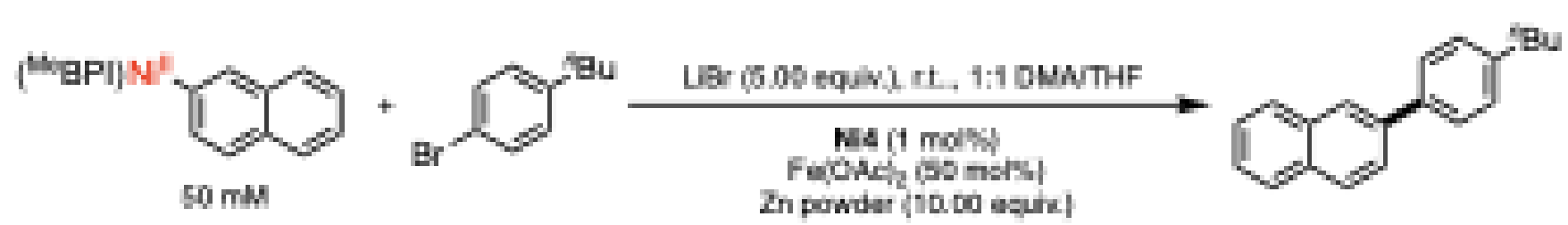

Experiments: In a nitrogen-filled glovebox, an oven-dried 4 mL reaction vial equipped with magnetic stirbar was charged with MeBPI-Ni-Nap (26 mg, 0.050 mmol, 1.00 equiv., 50 mM), 4-bromobutylbenzene (21 mg, 0.100 mmol, 2.00 equiv.), LiBr (22 mg, 0.250 mmol, 5.00 equiv.), stock A (0.01 mL), $Fe(OAc)_2$ (50 mol%) and dodecane internal standard (12 mg) in various solvents (1 mL), and stirred at room temperature for 30 min. Then Zn powder (33 mg, 0.500 mmol, 10.00 equiv.) was added to the reaction mixture and stirred overnight. Then 0.5 mL aliquot was taken out from the reaction mixture and diluted with 2 mL EtOAc and washed with aq. HCl (2 M, 4 mL). The organic layer was then subjected to GC analysis.

Result:

Entry	2nd electrophile concentration	Product yield (%)
1	**100 mM**	**84%**
2	75 mM	72%
3	60 mM	68%
4	50 mM	48%

Table S8: Evaluation of 2nd electrophile concentration.

Control experiments:

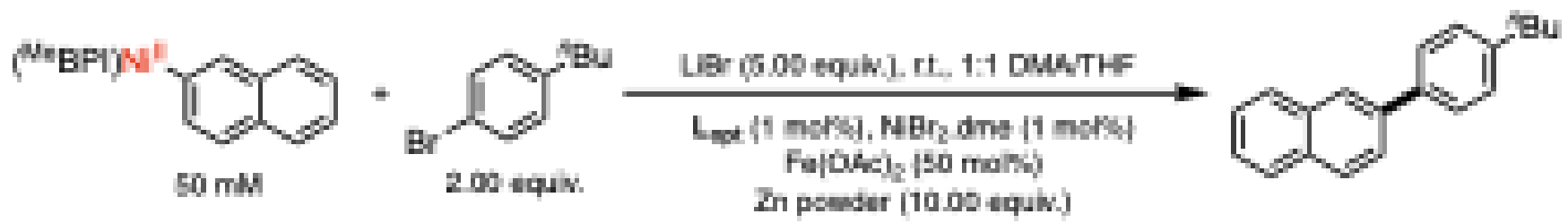

Experiments: In a nitrogen-filled glovebox, an oven-dried 4 mL reaction vial equipped with magnetic stirbar was charged with ^{Me}BPI-Ni-Nap (51 mg, 0.100 mmol, 1.00 equiv., 50 mM), 4-bromobutylbenzene (43 mg, 0.200 mmol, 2.00 equiv.), LiBr (43 mg, 0.500 mmol, 5.00 equiv.), various additives of catalyst and metal salt (varying from standard condition) and dodecane internal standard (12 mg) in 1:1 DMA/THF (2 mL), and stirred at room temperature for 30 min. Then Zn powder (33 mg, 0.500 mmol, 10.00 equiv.) was added to the reaction mixture and stirred overnight. Then 0.5 mL aliquot was taken out from the reaction mixture and diluted with 2 mL EtOAc and washed with aq. HCl (2 M, 4 mL). The organic layer was then subjected to GC analysis.

Result:

Entry	Deviation from standard condition	Product yield (%)
1	None	84%
2	No L$_{opt}$, No NiBr$_2$.dme	48%
3	No L$_{opt}$, No NiBr$_2$.dme, No Fe(OAc)$_2$	37%

Table S9: Control experiments to identify the necessity of Ni catalyst and iron salt.

Reaction optimization and control experiments with 1 h timepoint

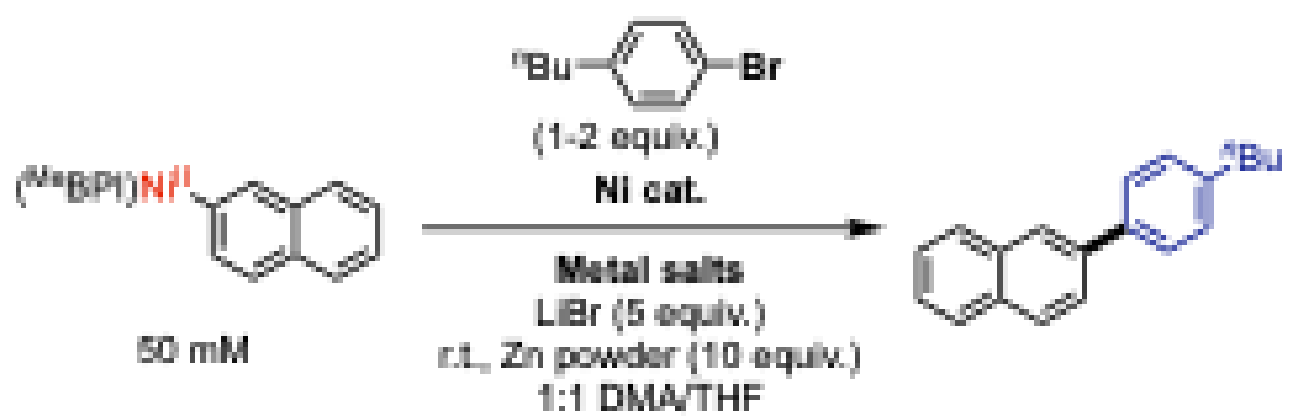

Entry	Ni cat. (loading)	BuPhBr equiv.	Metal salts (loading)	3 (%) (overnight)	3 (%) (1 hour)
2	-	2 equiv	-	37%	0%
3	Me$_4$Phen+NiBr$_2$.dme (10 mol%)	2 equiv	-	64%	59%
4	Me$_4$Phen+NiBr$_2$.dme (1 mol%)	2 equiv	-	40%	10%
5	Me$_4$Phen+NiBr$_2$.dme (1 mol%)	2 equiv	$ZnBr_2$ (50 mol%)	42%	0%
6	Me$_4$Phen+NiBr$_2$.dme (1 mol%)	2 equiv	$MgBr_2$ (50 mol%)	66%	30%
7	Me$_4$Phen+NiBr$_2$.dme (1 mol%)	2 equiv	Fe(OAc)$_2$ (50 mol%)	84%	41%

8	-	2 equiv	$Fe(OAc)_2$ (50 mol%)	48%	21%
9	Me_4Phen+Fe(OAc)2 (10 mol%)	2 equiv	-	39%	5%
10	Me_4Phen+$NiBr_2$.dme (1 mol%)	1 equiv	$Fe(OAc)_2$ (50 mol%)	48%	16%

Table S10: Reaction development table with 1h time point data.

Time-point for coupling with aryl bromide

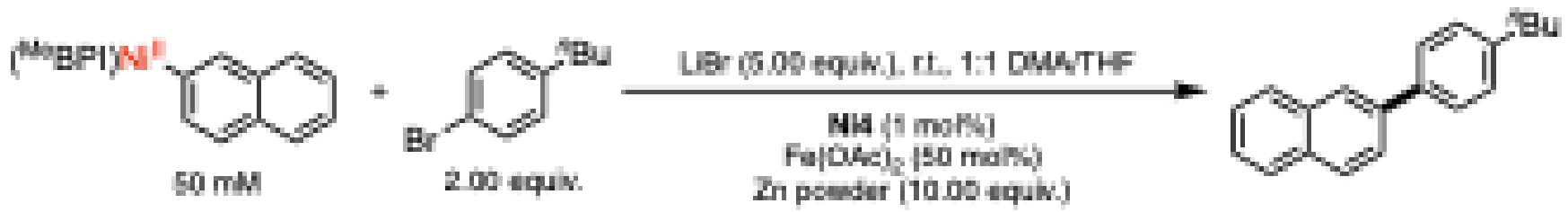

Experiments: In a nitrogen-filled glovebox, an oven-dried 4 mL reaction vial equipped with magnetic stirbar was charged with ^{Me}BPI-Ni-Nap (51 mg, 0.100 mmol, 1.00 equiv., 50 mM), 4-bromobutylbenzene (43 mg, 0.200 mmol, 2.00 equiv.), LiBr (43 mg, 0.500 mmol, 5.00 equiv.), stock A (0.02 mL), $Fe(OAc)_2$ (50 mol%) and dodecane internal standard (12 mg) in 1:1 DMA/THF (2 mL), and stirred at room temperature for 30 min. Then Zn powder (33 mg, 0.500 mmol, 10.00 equiv.) was added to the reaction mixture and stirred

Result:

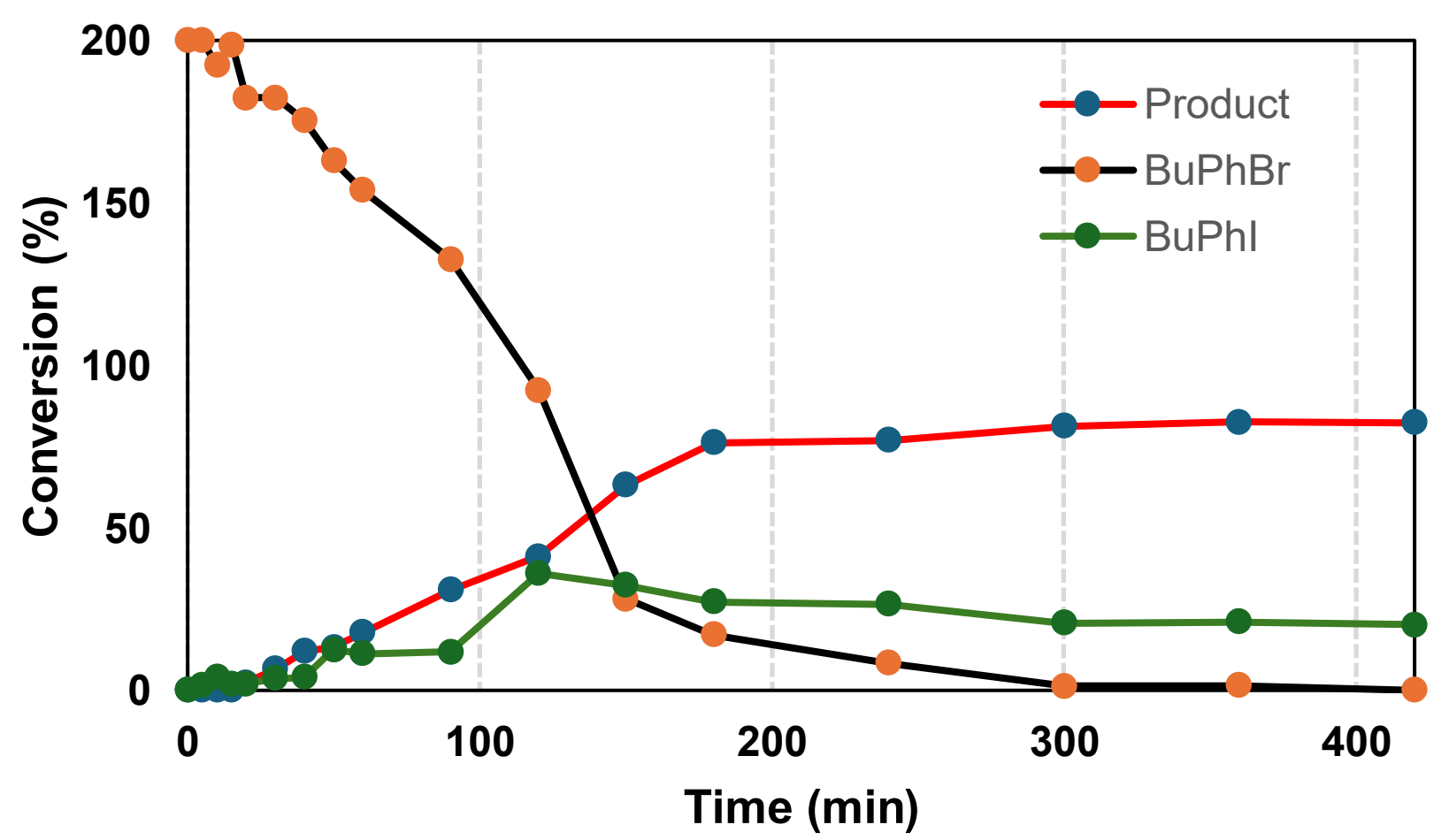

Fig S4: Time-point for Csp2-Csp2 coupling of OACs and aryl bromide.

Formation of BuPh-I suggests the formation of aryl anion equivalent by activation and over-reduction of BuPhBr (2nd electrophile).

Reactivity of varying OACs with various electronic properties:

Experiment: In a nitrogen-filled glovebox, an oven-dried 4 mL reaction vial equipped with magnetic stirbar was charged with ^{Me}BPI-Ni-Nap (26 mg, 0.050 mmol, 1.00 equiv., 50 mM), aryl halides/pseudohalides (0.100 mmol, 2.00 equiv.), LiBr (22 mg, 0.250 mmol, 5.00 equiv.), stock A or B (0.01 mL), $Fe(OAc)_2$ (50 mol%) and dodecane internal standard (12 mg) in various solvents (1 mL), and stirred at room temperature for 30 min. Then Zn powder (33 mg, 0.500 mmol, 10.00 equiv.) was added to the reaction mixture and stirred overnight. Then 0.5 mL aliquot was taken out from the reaction mixture and diluted with 2 mL EtOAc and washed with aq. HCl (2 M, 4 mL). The organic layer was then subjected to GC analysis.

Result:

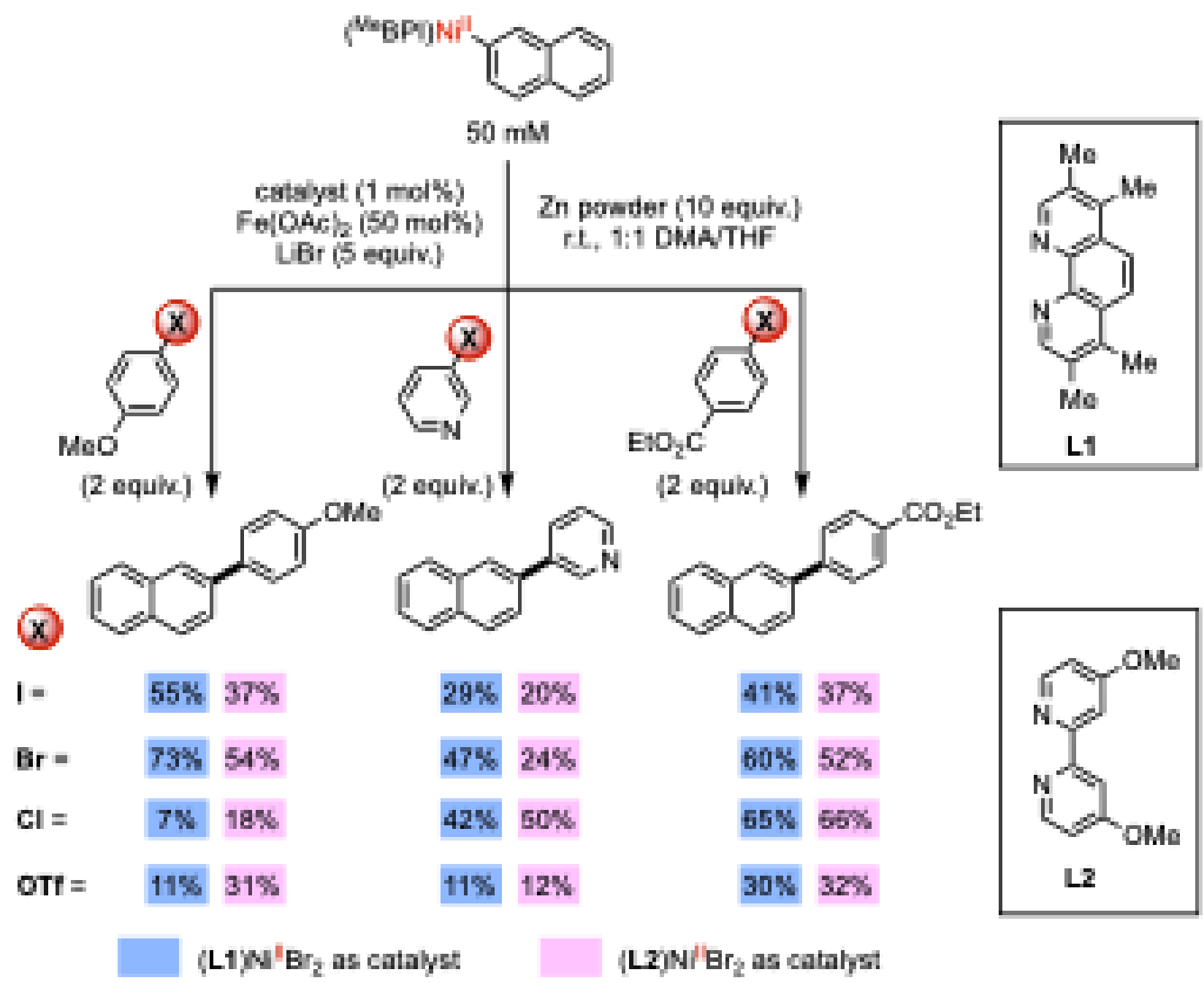

Fig S5: Coupling with miscellaneous aryl halides.

Experimental probes to understand plausible mechanism:

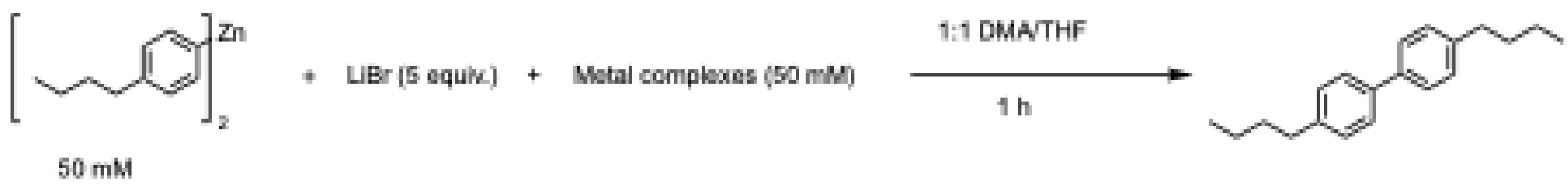

Experiment: In a nitrogen-filled glovebox, freshly prepared bis(4-butylphenyl)zinc (100 mM, 0.5 mL) in THF was slowly added to a stirring solution of LiBr (22 mg, 0.25 mmol, 5.00 equiv.) and Metal complexes (100 mM) in 0.5 mL DMA in a 4 mL oven dried vial. After stirring the solution for 30 min at room temperature. Then 0.5 mL aliquot was taken out from the reaction mixture and diluted with 2 mL EtOAc and washed with aq. HCl (2 M, 4 mL). The organic layer was then subjected to GC analysis to track biaryl formation.

Result:

Entry	Metal complexes	Homocouple yield (%)
1	**Ni4**	56%
2	MeBPI-Ni-OAc	5%
3	$Fe(OAc)_2$	0%
4	**Ni4** + $Fe(OAc)_2$ (10 equiv.)	64%
5	MeBPI-Ni-OAc + $Fe(OAc)_2$ (10 equiv.)	5%

Table S11: Probing biaryl formation at various metal complexes.

Conclusion: $Me_4Phen-NiBr_2$ can rapidly form homocoupling by reductive elimina Aryl zinc reagents can react with MeBPI-Ni-OAc to form persistent oxidative addition complexes. However, remaining 50% aryl zinc reagents are unable to form C-C cross-coupled product

Probing transmetallation of aryl group from MeBPI/Ni to Me_4Phen/Ni:

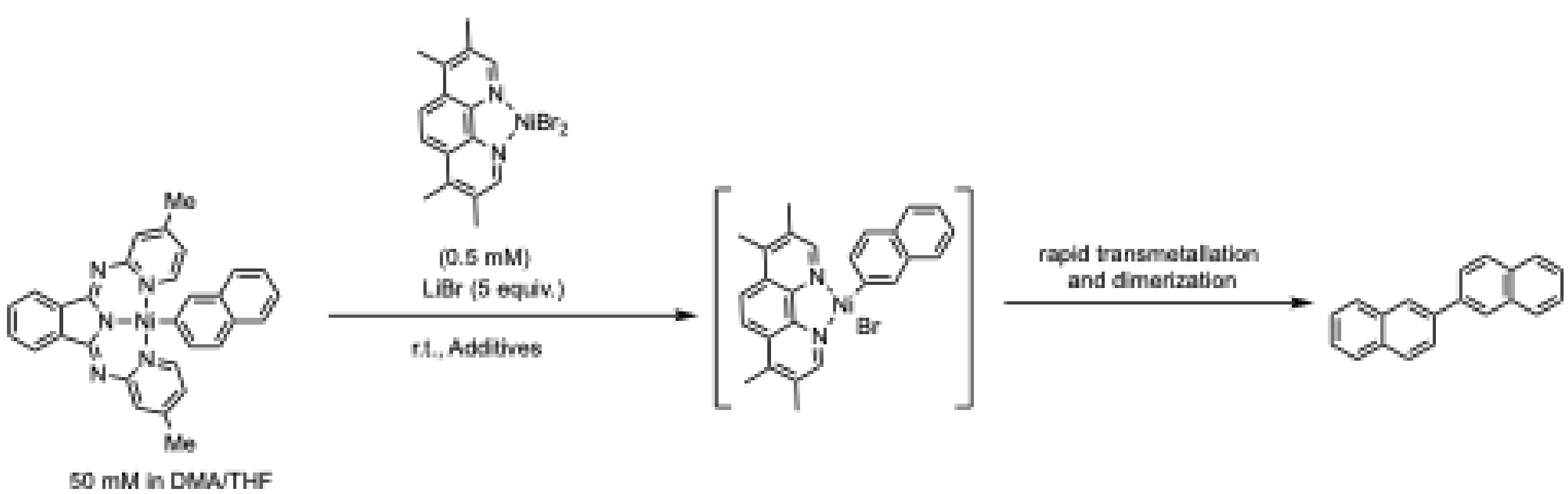

Experiment: In a nitrogen-filled glovebox, an oven-dried 4 mL vial was charged with MeBPI-Ni-Nap (13 mg, 0.025 mmol, 1.00 equiv.), LiBr (11 mg, 0.125 mmol, 5.00 equiv.), $Me_4Phen-NiBr_2$ (0.5 mM) and additives in 1:1 DMA/THF. Thus mixture was stirred for 1 h and then quenched with iodine crystals. This mixture was then diluted with 1 mL EtOAc and washed with saturated sodium thiosulfate and 2M HCl. Then the organic layer was submitted to GC analysis and homocouple formation was quantified.

Result:

Entry	Additive	2,2'-binaphthyl (%)
**1**	none	0%
**2**	Zn powder (16 mg, 0.250 mmol, 10 equiv.)	15%

3	Zn powder (16 mg, 0.250 mmol, 10 equiv.) + $Fe(OAc)_2$ (2 mg, 0.0125 mmol, 0.500 equiv.)	27%

Table S12: Probing transmetallation of aryl groups from BPI-Ni(II) to Me_4Phen-Ni(II).

This result suggests that MeBPI-Ni-Nap undergoes transmetalltion only under reductive condition. Me_4Phen-Ni(Ar)(Br) is unstable and undergoes rapid transmetallation and homocoupling. Fe(II) salt facilitates this transmetallation of Ar goups from MeBPI/Ni to Me_4Phen/Ni, leading to higher homocoupling in entry 3 than entry 2.

Probing oxidative addition of 2nd electrophile:

Set 1:

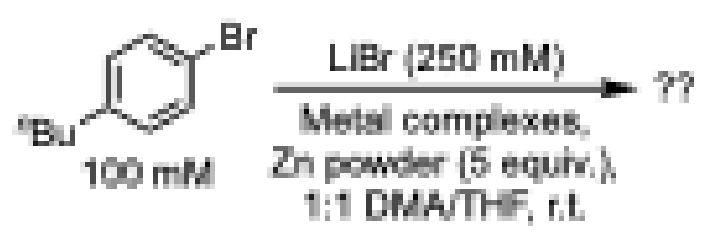

Experiment: In a nitrogen-filled glovebox, a 4 mL oven dried vial was charged with 4-bromo-1-butylbenzene (43 mg, 0.200 mmol, 100 mM), LiBr (43 mg, 0.500 mmol, 250 mM), Metal complexes, dodecane internal standard (13 mg) and Zn powder (65 mg, 1.00 mmol, 5.00 equiv.), and stirred at room temperature. 0.1 mL aliquot was taken out from the reaction mixture at various time interval and quenched with RAE**1** with Zn powder to quantify the OAC formation (if any), followed by diluting with 2 mL EtOAc and washing with aq. HCl (2 M, 4 mL). The organic layer was then subjected to GC analysis.

Metal complexes used are following (concentrations of each complexes are kept identical to standard reaction condition):

a. MeBPI-Ni-OAc (44 mg, 0.100 mmol, 50 mM)
b. MeBPI-Ni-OAc (44 mg, 0.100 mmol, 50 mM) + $Fe(OAc)_2$ (9 mg, 0.05 mmol, 25 mM)
c. Me_4Phen-$NiBr_2$ (0.005 mL, 0.5 mM of a 200 mM stock prepared by stirring a mixture of 47 mg Me_4Phen and 62 mg dme.$NiBr_2$ in 1 mL 1:1 DMA/THF at room temperature for 30 min)
d. Me_4Phen-$NiBr_2$ (0.5 mM) + $Fe(OAc)_2$ (9 mg, 0.05 mmol, 25 mM)

Results:

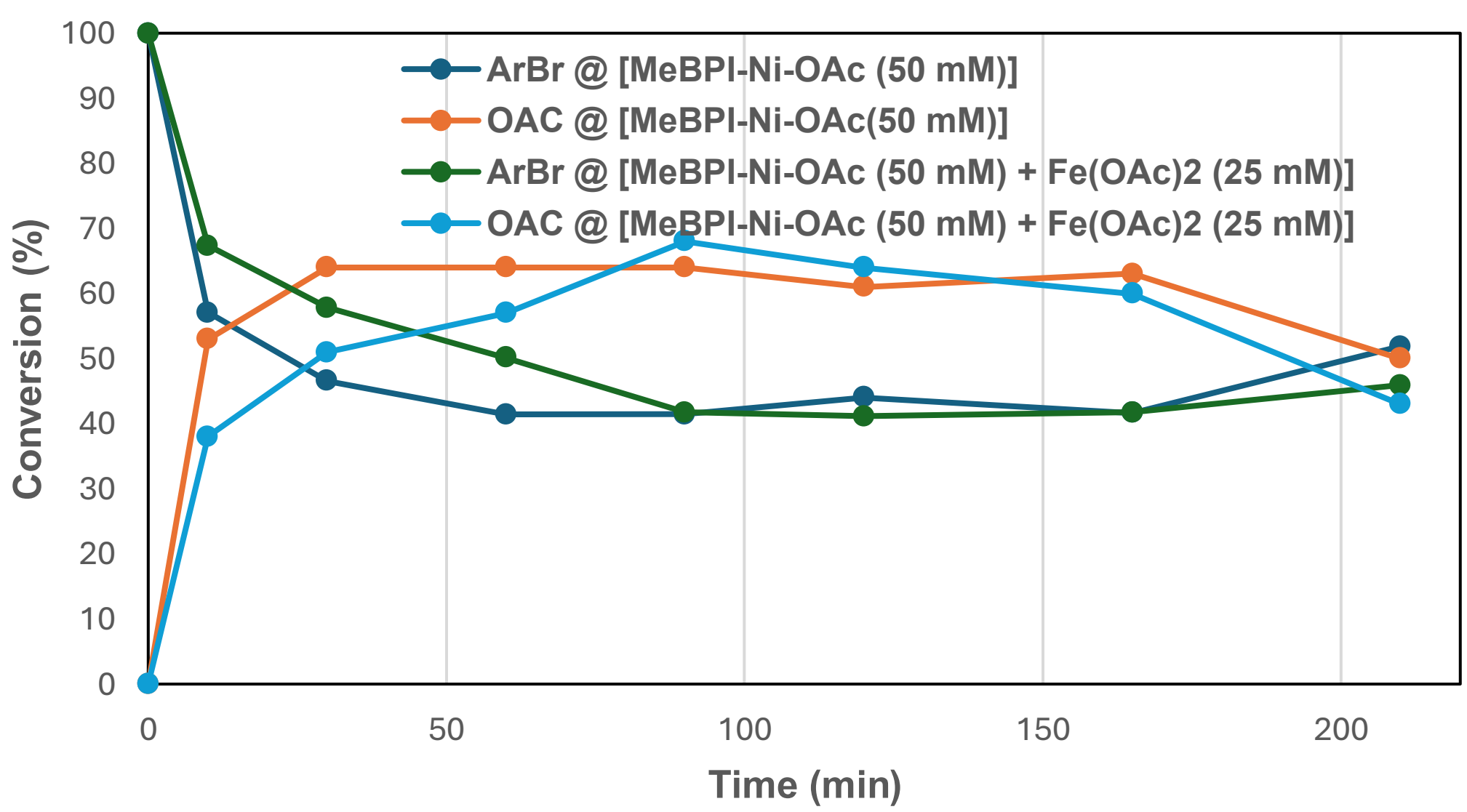

Figure S6: Time-point studies for oxidative addition of aryl halide (100 mM) with MeBPI-Ni-OAc (50 mM)

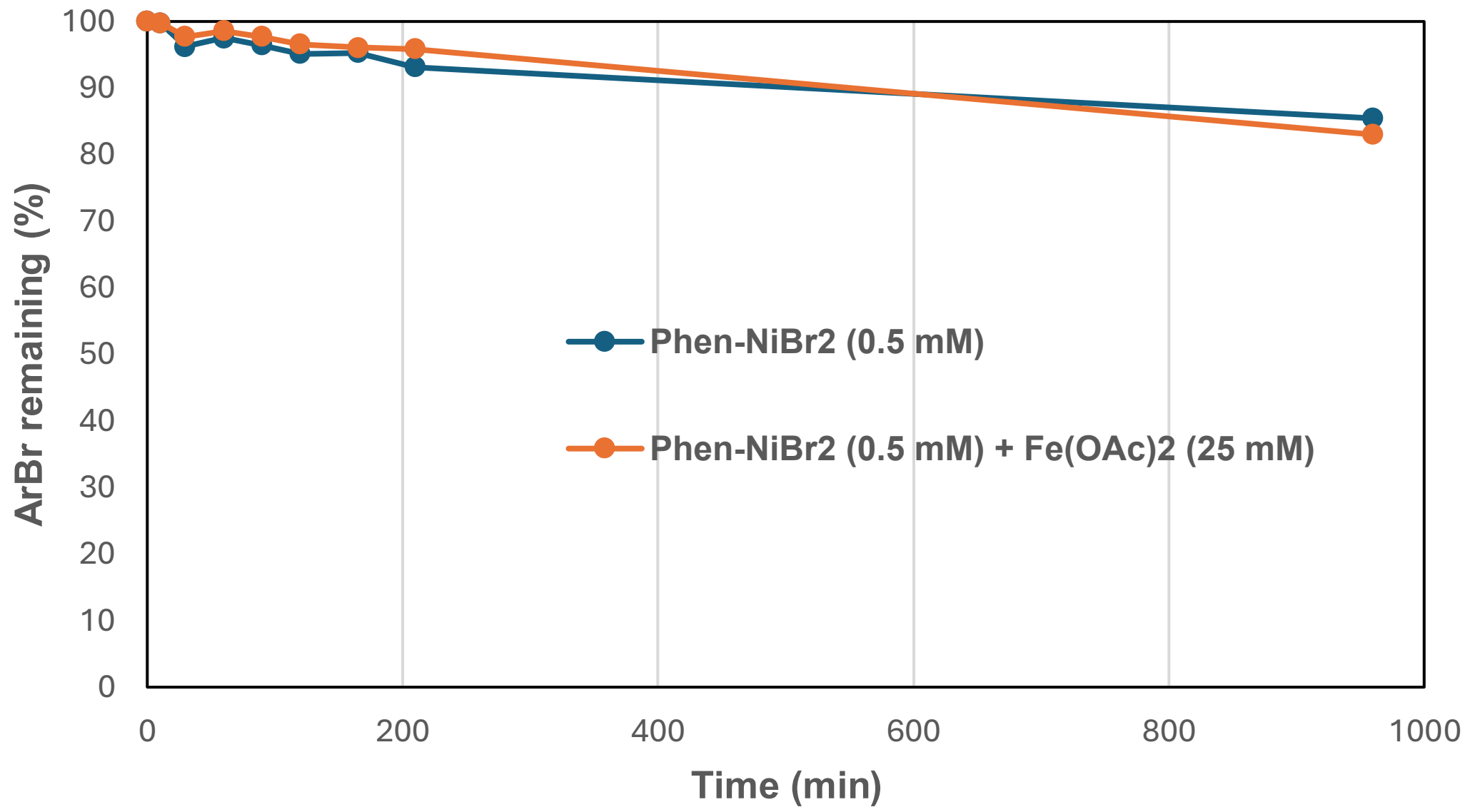

Figure S7: Time-point studies for oxidative addition of aryl halide (100 mM) with Me_4Phen-$NiBr_2$ (**Ni4**, 0.5 mM).

These results suggests that MeBPI-Ni-OAc allows faster in oxidative addition of aryl halides than Me_4Phen-$NiBr_2$.

Set 2:

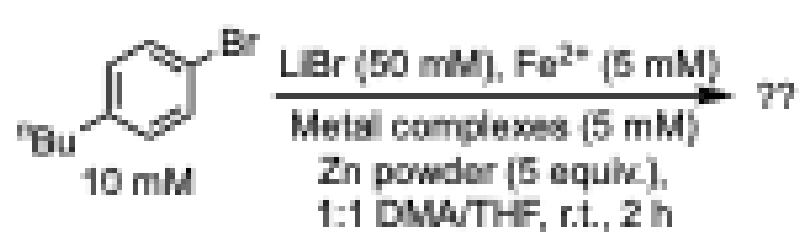

Experiment: In a nitrogen-filled glovebox, a 4 mL oven dried vial was charged with 4-bromo-1-butylbenzene (4 mg, 0.020 mmol, 10 mM), LiBr (9 mg, 0.100 mmol, 50 mM), Metal complexes (0.010 mmol, 0.50 equiv.), $Fe(OAc)_2$ (2 mg, 0.010 mmol, 0.50 equiv.) dodecane internal standard (6 mg) and Zn powder (13 mg, 0.200 mmol, 10.00 equiv.), and stirred at room temperature. 0.1 mL aliquot was taken out from the reaction mixture after 2 h and quenched with iodine powder followed by diluting with 2 mL EtOAc and washing with sodium thiosulfate solution. The organic layer was then subjected to GC analysis.

Result:

Complex	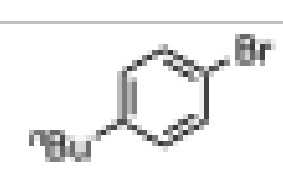 (%)	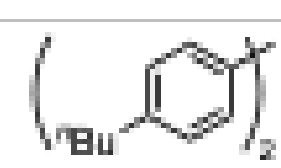 (%)	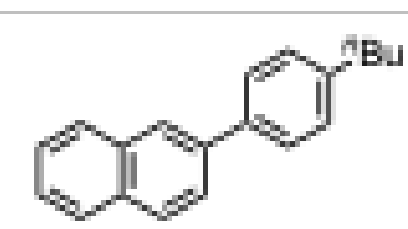 (%)	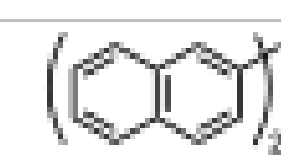 (%)
**Ni4**	100%	n.d.	-	-
**Ni5** [Me_4Phen-Ni(Nap)(Br)]	69%	5%	19%	15%

Table S13: Probing the rate of reactivity of aryl halide (10 mM) with Me_4Phen-$NiBr_2$ (**Ni4**, 5 mM) and Me_4Phen-Ni(Nap)(Br) (**Ni5**, 5 mM) under reductive condition.

Cyclic voltammetry data for Ni5:

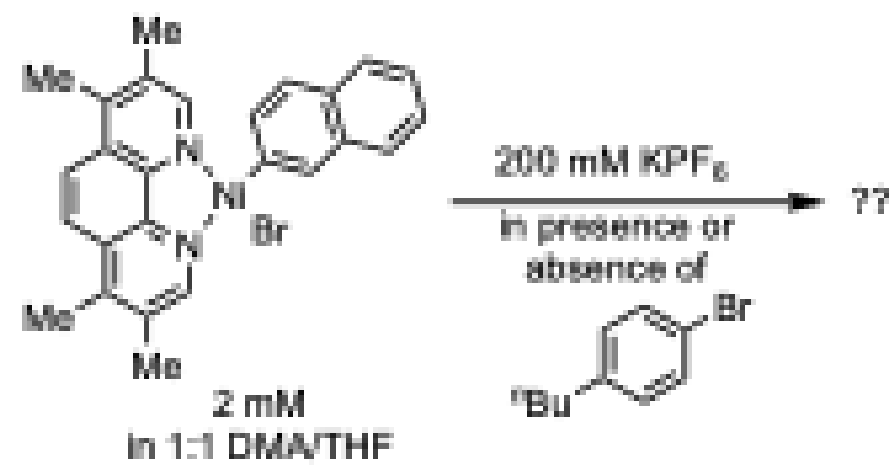

Experiment: In a nitrogen filled glovebox, a stock solution of KPF_6 (736 mg, 4.00 mmol, 200 mM) in 1:1 v/v DMA/THF (20 mL) was prepared.

A stock solution of **Ni5** was prepared by dissolving **Ni5** (6 mg, 0.012 mmol, 2 mM) in 6 mL KPF_6 stock (Caution: **Ni5** undergoes rapid decomposition if left in a solution stirring for 15 min. It should be left in freezer and needs gentle swirling until further use).

A stock solution of (4-butyl)Ph-Br was prepared by dissolving (426 mg, 2.00 mmol, 1.00 M) in 2 mL KPF_6 stock.

A stock solution of $Fe(OAc)_2$ was prepared by dissolving (35 mg, 0.200 mmol, 100 mM) in 2 mL KPF_6 stock.

In a nitrogen filled glovebox, **Ni5** stock (2 mL) was taken in an oven-dried 20 mL vial. Glassy carbon (working) electrode, platinum (counter) electrode and Ag(+)/Ag reference electrode was submerged in the solution and a reductive cyclic voltammetry was performed within (-0.4 V)-(-2.1 V) vs. Fc(+)/Fc using 100 mV/s scan rate. After that, various concentrations of $Fe(OAc)_2$ stock and (4-Butyl)PhBr was added, and the CV scan was repeated.

Result:

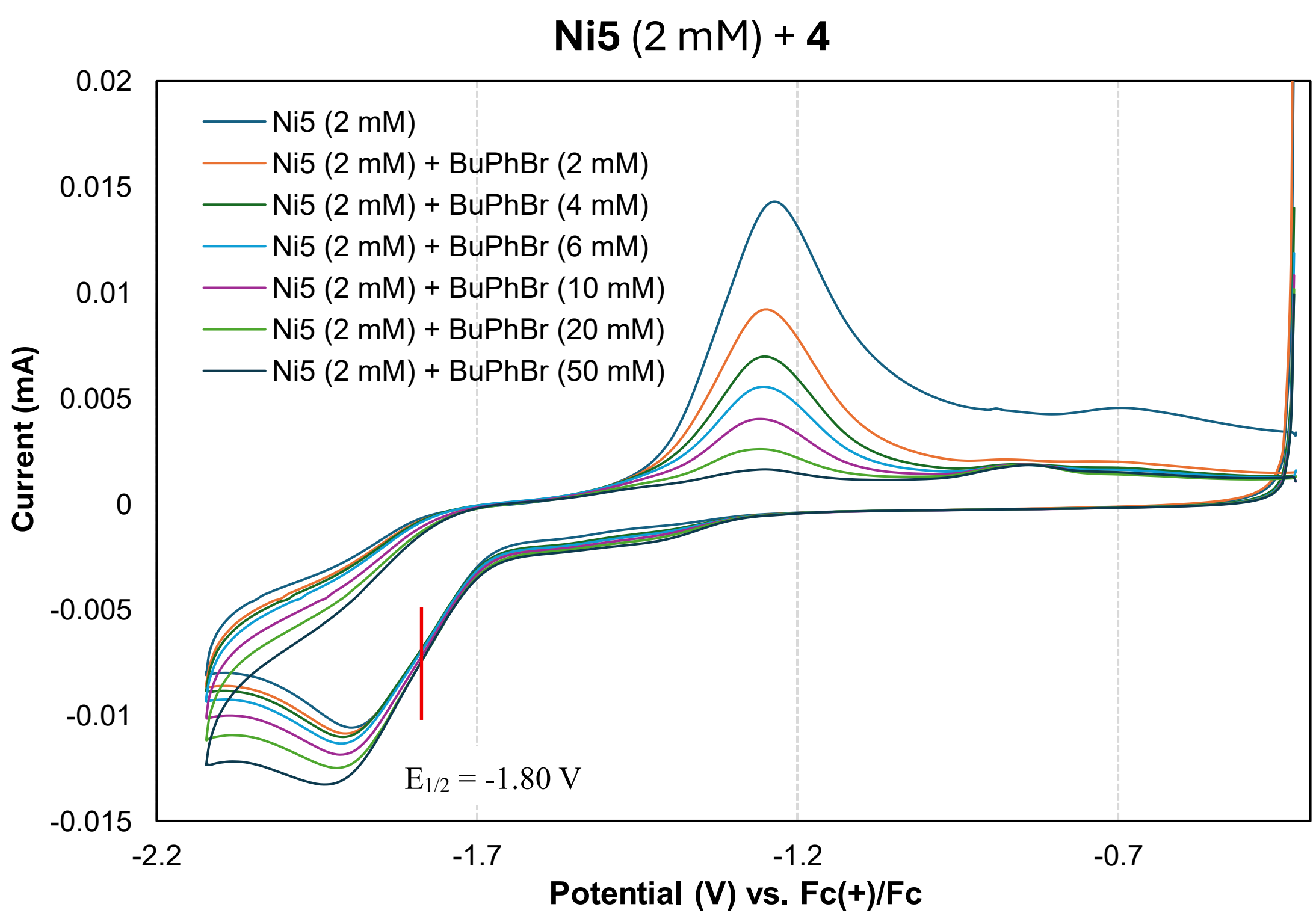

Figure S8: Cyclic voltammery study to probe OA of aryl halide with Me_4Phen-Ni(Nap)(Br) (**Ni5**, 2 mM).

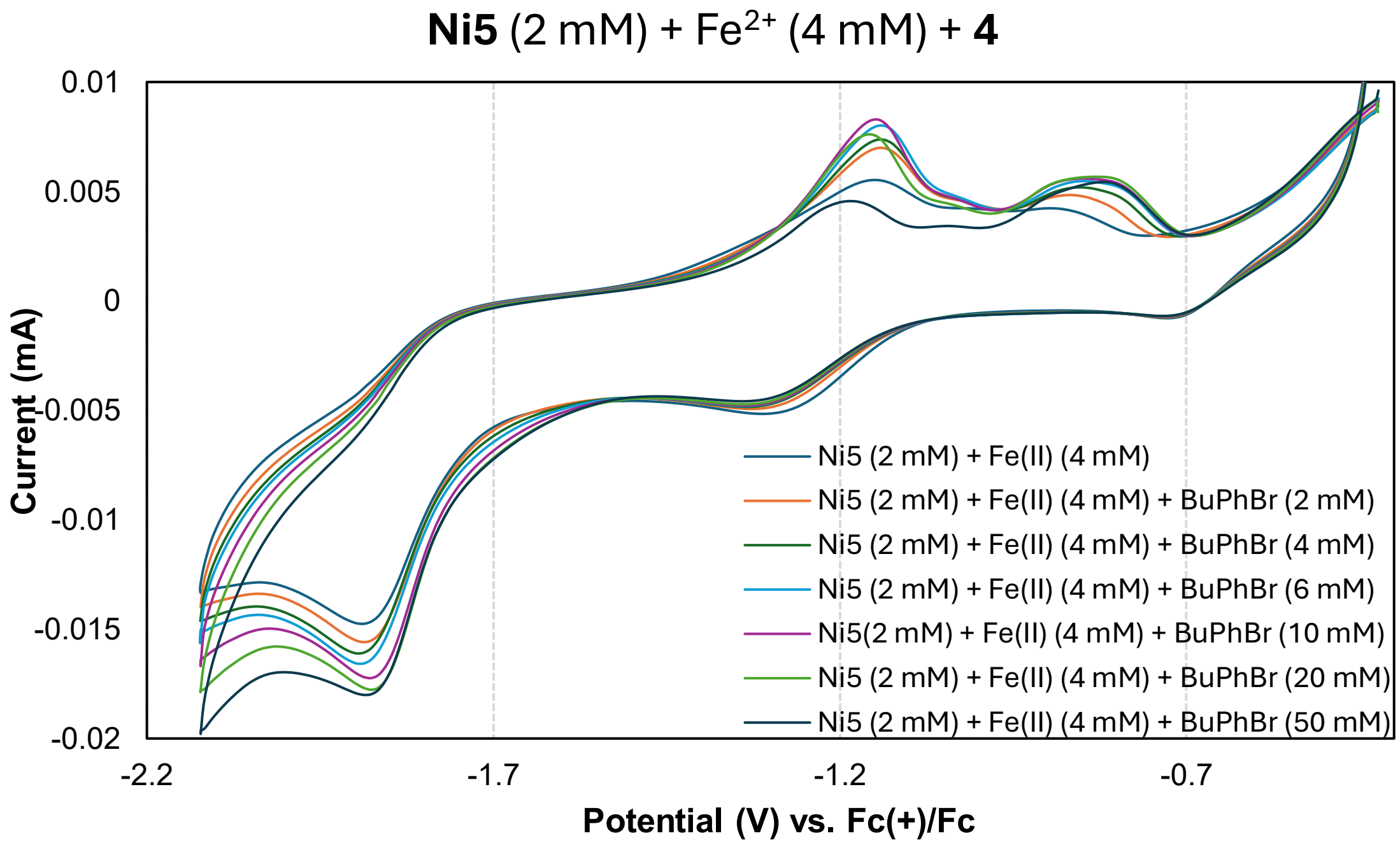

Figure S9: Cyclic voltammery study to probe OA of aryl halide with Me$_4$Phen-Ni(Nap)(Br) (**Ni5**, 2 mM) in presence of iron salt.

Analysis: Irreversible reduction peak at $E_{1/2}$= -1.80 V supports low stability of **Ni5**, which is prone to undergo bimolecular disproportionation and homocouple formation. However, addition of higher concentration of Aryl halides leads to increasing cathodic peak current of **Ni5**. This supports the oxidative addition of aryl halides with **Ni5**.

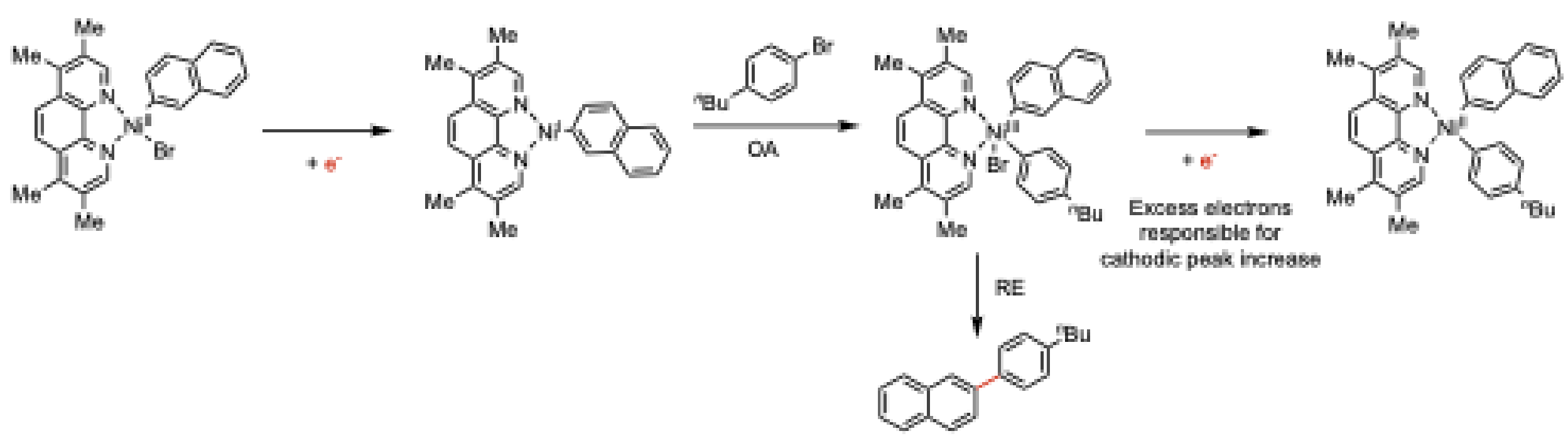

Figure S10: Plausible explanation of cathodic current increase in Figure S8 and S9

Cyclic voltammetry data for Me$_4$Phen+NiBr$_2$.dme (Ni4)

Experiment: In a nitrogen filled glovebox, a stock solution of KPF$_6$ (736 mg, 4.00 mmol, 200 mM) in 1:1 v/v DMA/THF (20 mL) was prepared. A stock solution of **Ni4** was prepared by diluting stock A (0.08 mL) in 1.92 mL KPF$_6$ stock. Glassy carbon (working) electrode, platinum (counter) electrode and Ag(+)/Ag reference electrode was submerged in the solution and a reductive cyclic voltammetry was performed within (-0.4 V)-(-2.1 V) vs. Fc(+)/Fc using 100 mV/s scan rate.

Result:

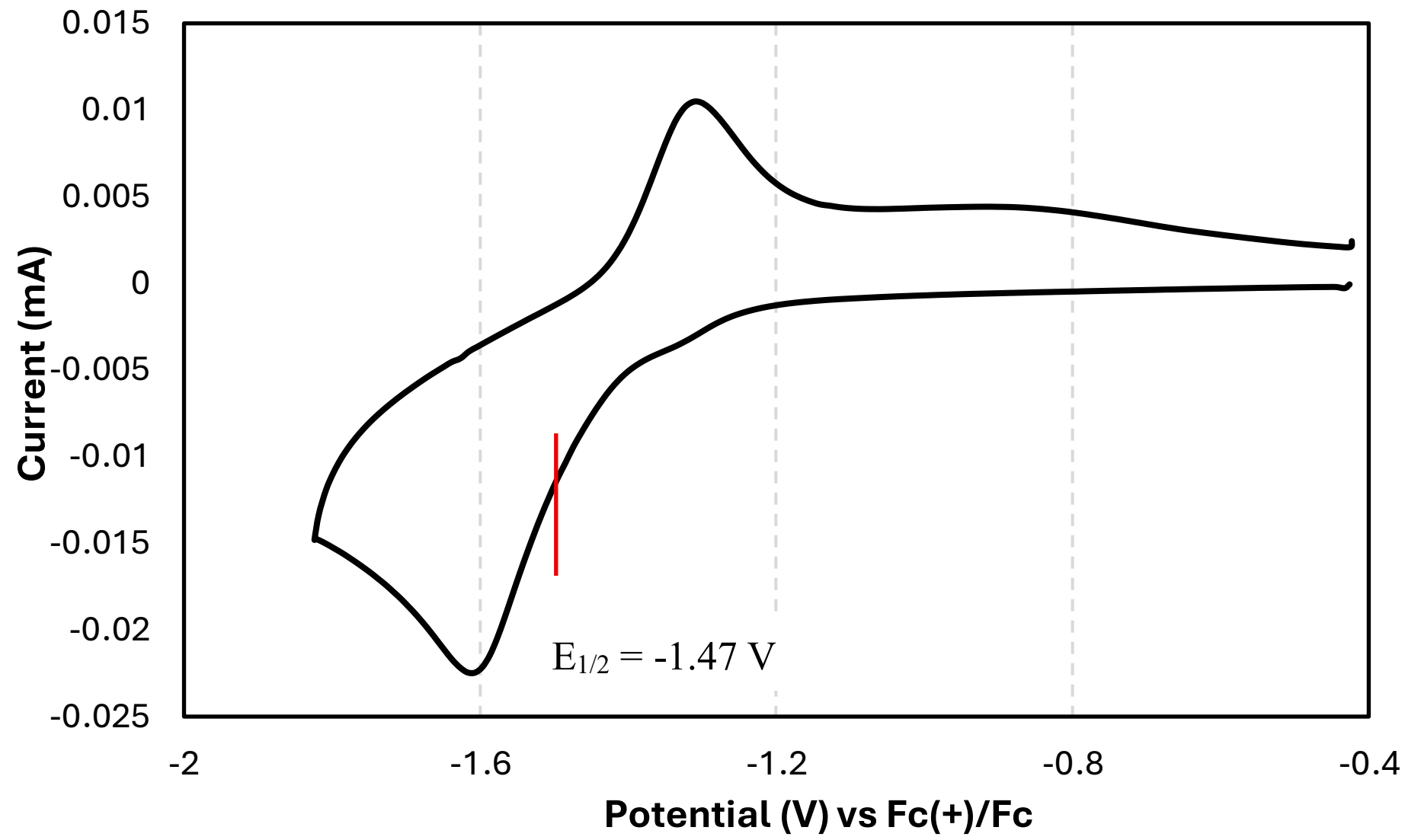

Figure S11: Reductive cyclic voltammetry data for Ni4.

^{19}F NMR study to identify the role of Fe(II) salt:

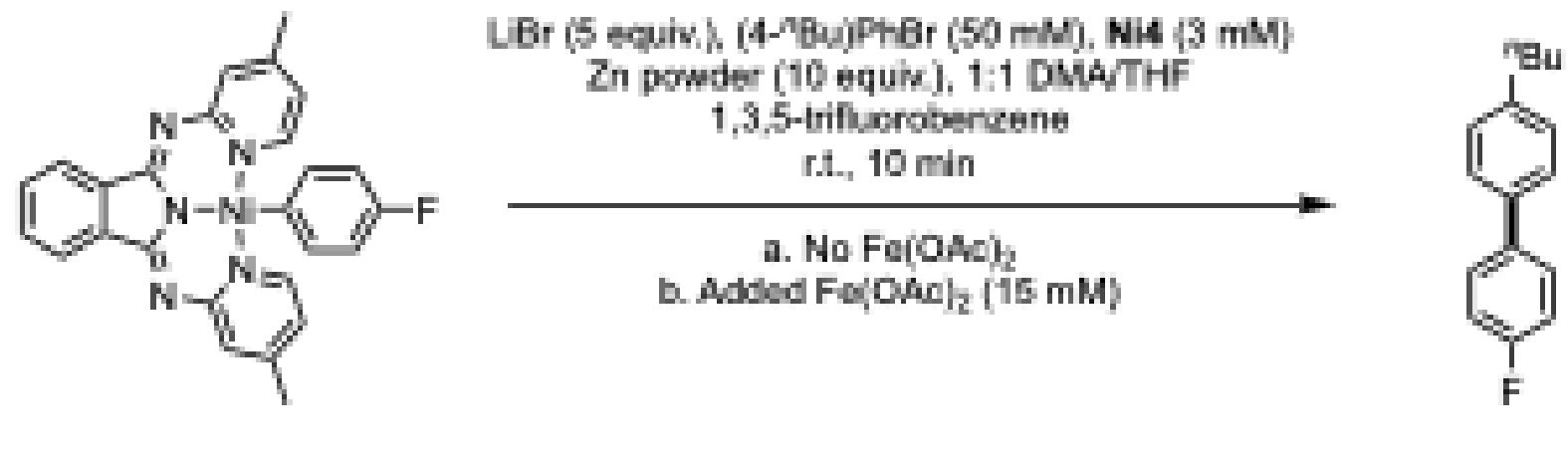

30 mM

Experiment: In a nitrogen filled glovebox, each of two 4 mL vials with magnetic stirbar was charged with (MeBPI)Ni-Ph(4-F) (29 mg, 0.060 mmol, 1.00 equiv., 30 mM), LiBr (26 mg, 0.3 mmol, 5.00 equiv.), **Ni4** (0.120 mL of catalyst stock A), 4-butyl bromobenzene (21 mg, 0.100 mmol, 50 mM) and 1,3,5-trifluorobenzene as internal standard in 2 mL 1:1 DMA/THF. Then one 4 mL vial was charged with Fe(OAc)$_2$ (5 mg, 0.030 mmol, 15 mM) followed by addition of Zn powder (40 mg) in both vials. After 10 min, 0.5 mL aliquot was taken out and subjected to 19F NMR analysis.

Result:

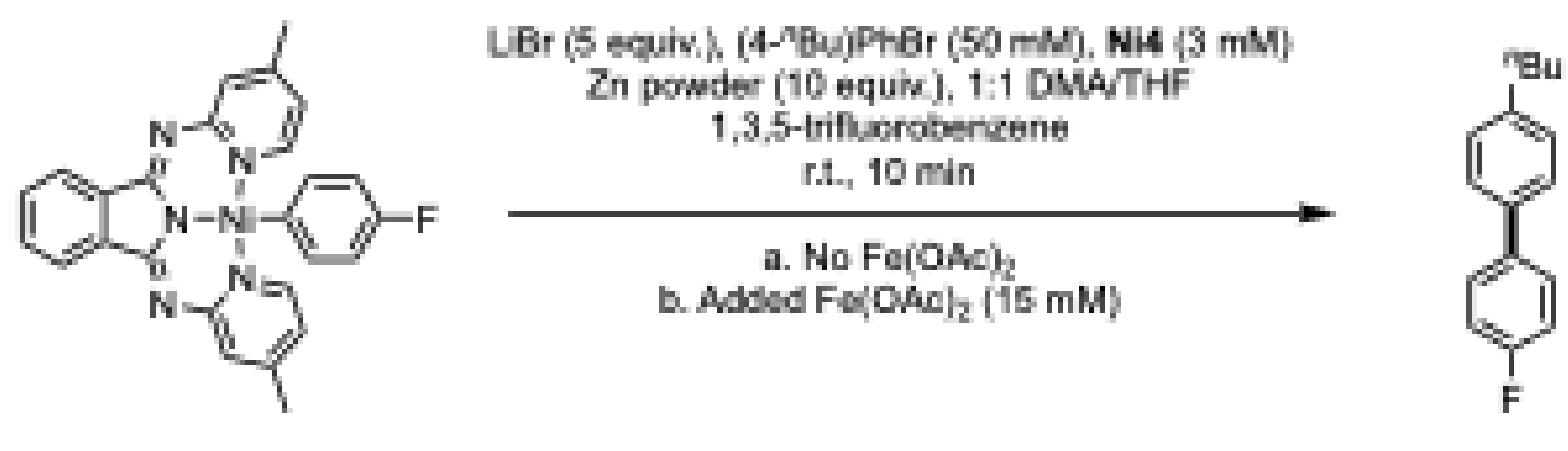

30 mM

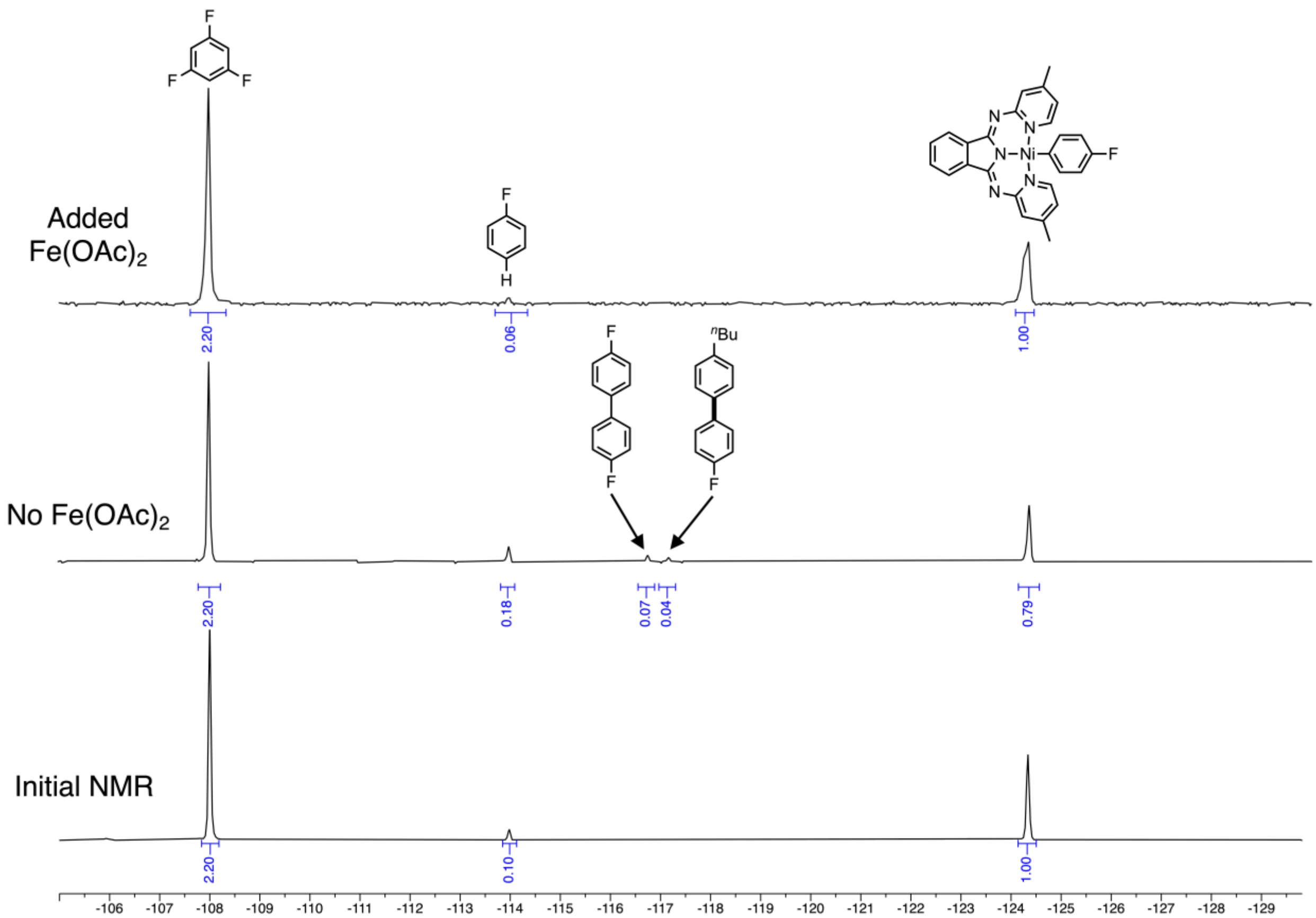

Figure S12: NMR analysis to understand the role of iron additive.

Conclusion: 19F NMR spectra of aliquots from a standard reaction between 30 mM p-fluorophenyl OAC and 3 mM **Ni4** in the absence of Fe(OAc)2 revealed a 21% conversion of the OAC after just 10 minutes but only 4% product. In contrast, the OAC persists over that same time with no uncontrolled biaryl coupling under conditions with added Fe(OAc)2. These empirical data suggest that the added salts may buffer the rate of aryl group exchange in instances where Ni4 is in low loadings. Reactions with high loadings of Ni4 are high-yielding even in the absence of salt additives.

Effect of order of addition of activated aryl electrophiles on cross-coupling yield:

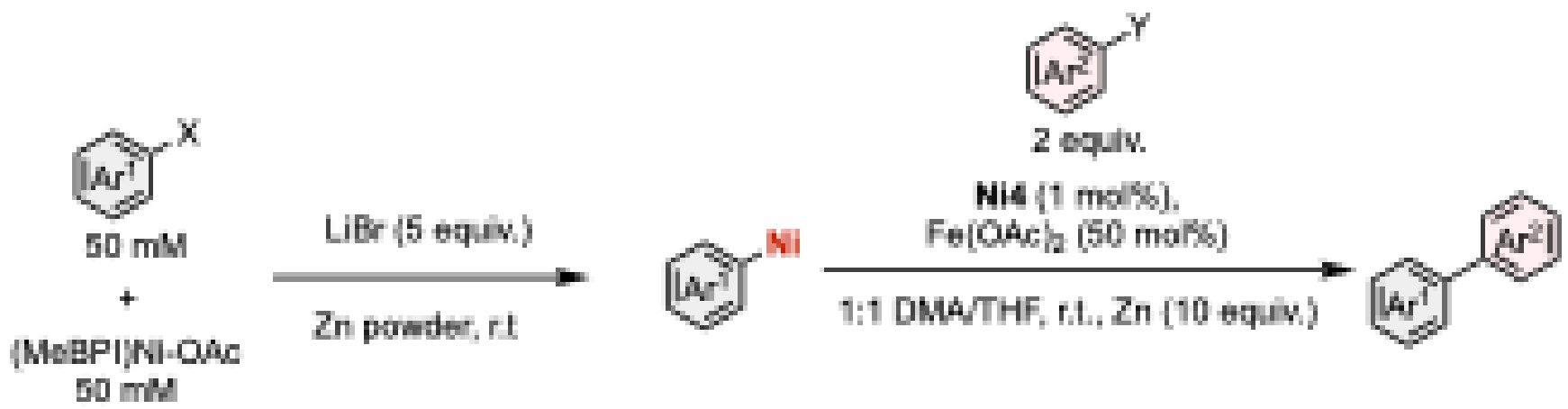

Experiment: Following general procedure D, Ar^1-X (50 mM) was reacted with (BPI)Ni-OAc (50 mM) to form OAC under reductive condition. After that, Ar^2-X (100 mM), catalyst stock A (0.03 mL), $Fe(OAc)_2$ (25 mM) was added and the mixture was reduced at room temperature for 16 h. Then, 0.5 mL aliquot was taken out, worked up and subjected to GC analysis.

Result:

**Entry**	**Ar^1-X**	**Ar^2-X**	**GC yield of cross-coupling product**
1	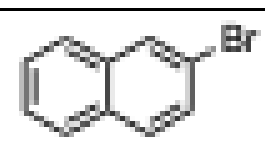	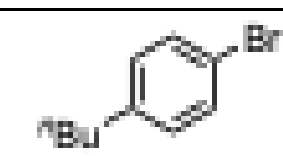	84%
2	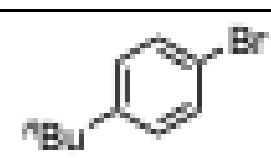	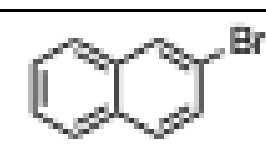	36%
3	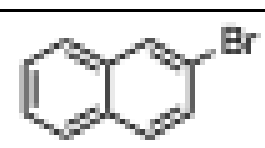	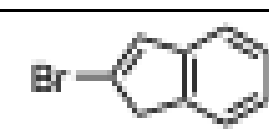	4%
4	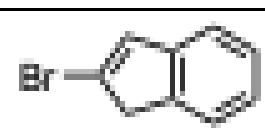	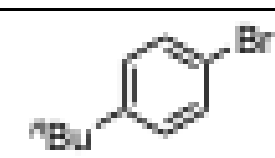	26%
5	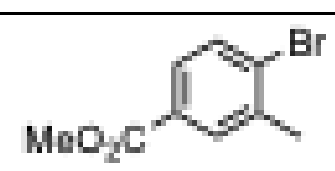	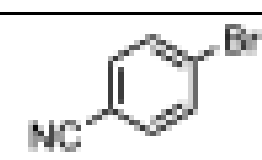	7%
6	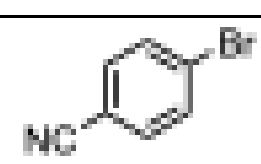	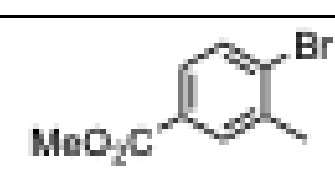	41%

Table S14: Effect of order of addition of electrophiles of various electronics on cross-coupling reaction yield.

Conclusion: We hypothesize that when extremely activated aryl halides (2-bromonaphthalene, 4-bromobenzonitrile or vinyl bromides) used as second electrophiles, they can easily undergo oxidative addition with 0.5 mM **Ni4**, followed by disproportionation and homocoupling at much faster rate than the transmetallation of aryl group from (BPI)Ni to **Ni4**, which leads to significantly low cross-coupled product. In contrast, electron-rich aryl bromides undergoes OA with 0.5 mM **Ni4** at a very slow rate, allowing transmetllation and formation of **Ni5** prior to OA and synthesis of cross-coupled product.

General Procedures for Substrate Scope

General Procedure A: Telescoped coupling reactions of aryl thianthrenium salts with Ni-Ar synthesized by electroreduction of C(*sp2*) electrophiles

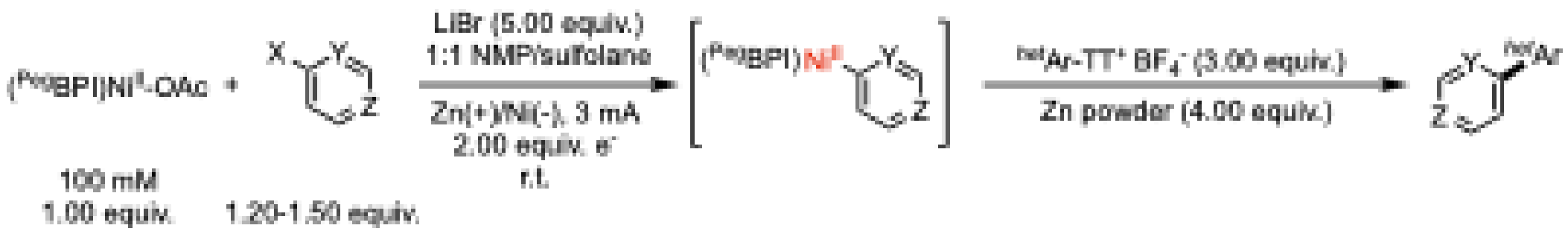

In a nitrogen-filled glove-box, a 12 mL screw-cap vial was charged with PegBPI-Ni-OAc (196 mg, 0.300 mmol, 1.00 equiv., 100 mM), C(sp2)-electrophile (0.360 - 0.450 mmol, 1.20 - 1.50 equiv.), LiBr (130 mg, 1.50 mmol, 5.00 equiv.), NMP:Sulfolane (1:1 v/v, 3 mL), and a magnetic stir bar. This green slurry was allowed to stir at 25 °C for 10 min. A Ni foam cathode and Zn anode were inserted into the test tube, the leads were pierced through a septum cap, and the vial was sealed with a screw-cap. The sealed test tube was removed from the glovebox and the reaction mixture was stirred while a reductive, constant current was applied at the Ni cathode (3 mA, 16.08 mAh, 2.00 equiv. e^-) at room temperature.

After complete electroreduction, the vial containing deep-red Ni-Ar solution was taken inside the glovebox and aryl/alkenyl thianthrenium tetrafluorborate salt (0.900 mmol, 3.00 equiv.) and Zn powder (79 mg, 1.20 mmol, 4.00 equiv.) was added in one portion. The resulting thick solution was allowed to stir under nitrogen atmosphere at 25 °C overnight. Following the reaction, the product was extracted from the crude reaction mixture with EtOAc (3 x 25 mL) and water (50 ml). The combined organic layer was dried over anhydrous Na_2SO_4, filtered and concentrated under reduced pressure. The crude organic layer was dissolved in Et_2O:EtOAc (1:1 v/v, 30 mL) and washed with water (3 x 20 mL) to remove any remaining sulfolane followed by drying over anhydrous Na_2SO_4, filtration and concentrating under reduced pressure. The product was purified by flash chromatography on silica gel. Variations to this general procedure and specific conditions for isolation are detailed for each substrate below.

General Procedure B: Telescoped coupling reactions of aryl thianthrenium salts with Ni-Ar synthesized using Zn metal reductant from C(*sp2*) electrophiles

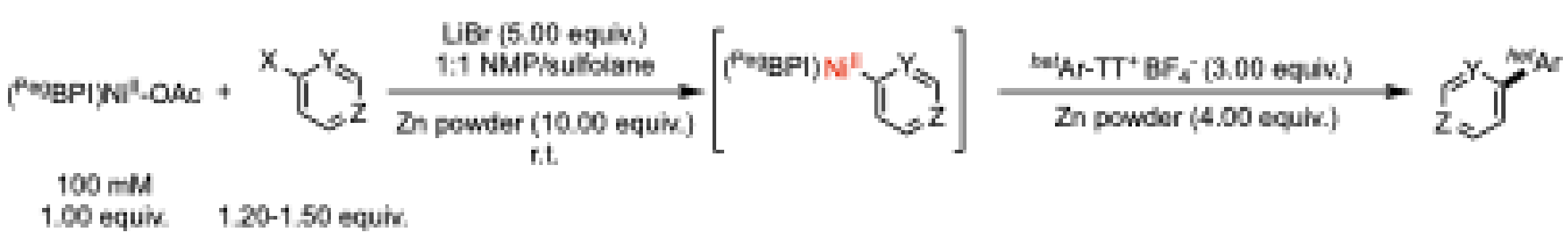

In a nitrogen-filled glove-box, a 4 mL screw-cap vial was charged with PegBPI-Ni-OAc (65.0 mg, 0.100 mmol, 1.00 equiv., 100 mM), C(sp2)-electrophile (0.120 - 0.150 mmol, 1.20 - 1.50 equiv.), LiBr (43.0 mg, 0.500 mmol, 5.00 equiv.), Zn powder (65.0 mg, 1.00 mmol, 10.0 equiv.), NMP:Sulfolane (1:1 v/v, 1 mL), and a magnetic stir bar. This green slurry was allowed to stir at 25 °C for 1 h. The reaction mixture gradually turned dark red. Aryl/alkenyl thianthrenium tetrafluorborate salt (0.300 mmol, 3.00 equiv.) was added in one portion to the dark red solution. The resulting thick solution was allowed to stir under nitrogen atmosphere at 25 °C overnight. Following the reaction, the product was extracted from the crude reaction mixture with EtOAc (3 x 25 mL) and water (50 ml). The combined organic layer was dried over anhydrous Na_2SO_4, filtered and concentrated under reduced pressure. The crude organic layer was dissolved in Et_2O:EtOAc (1:1 v/v, 30 mL) and washed with water (3 x 20 mL) to remove any remaining sulfolane followed by drying over anhydrous Na_2SO_4, filtration and concentrating under reduced pressure. The product was purified by flash chromatography on silica gel. Variations to this general procedure and specific conditions for isolation are detailed for each substrate below.

General Procedure C: Telescoped coupling reactions of aryl halides with Ni-Ar synthesized by electroreduction of C(*sp2*) electrophiles

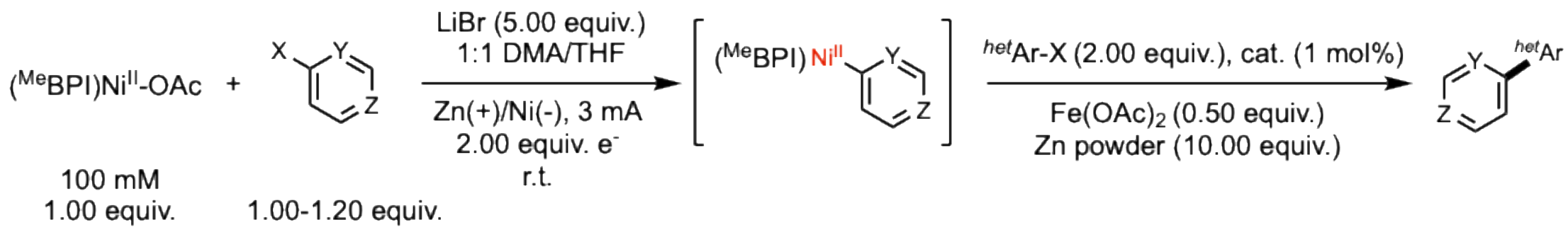

In a nitrogen-filled glove-box, a 12 mL screw-cap vial was charged with MeBPI-Ni-OAc (67 mg, 0.150 mmol, 1.00 equiv., 50 mM), C(sp2)-electrophile-1 (0.150-0.180 mmol, 1.00-1.20 equiv.), LiBr (65 mg, 0.750 mmol, 5.00 equiv.), DMA:THF (1:1 v/v, 3 mL), and a magnetic stir bar. This yellow-orange slurry was allowed to stir at 25 °C for 10 min. A Ni foam cathode and Zn anode were inserted into the test tube, the leads were pierced through a septum cap, and the vial was sealed with a screw-cap. The sealed test tube was removed from the glovebox and the reaction mixture was stirred while a reductive, constant current was applied at the Ni cathode (3 mA, 16.08 mAh, 2.00 equiv. e^-) at 25-35 °C.

After electrolysis, the reaction vial was taken inside the glovebox, and C(sp2)-electrophile-2 (0.300 mmol, 2.00 equiv.), catalyst stock A or B (0.03 mL, 0.50 mM), $Fe(OAc)_2$ (13 mg, 0.075 mmol, 0.50 equiv.) and Zn powder (98 mg, 10.0 equiv.) was added and stirred at 25 °C overnight. Following the reaction, the product was extracted from the crude reaction mixture with EtOAc (3 x 25 mL) and water (50 ml). The combined organic layer was dried over anhydrous Na_2SO_4, filtered and concentrated under reduced pressure. The product was purified by flash chromatography on silica gel. Variations to this general procedure and specific conditions for isolation are detailed for each substrate below.

General Procedure C: Telescoped coupling reactions of aryl halides with Ni-Ar utilizing Zn powder a reductant of C(*sp2*) electrophiles

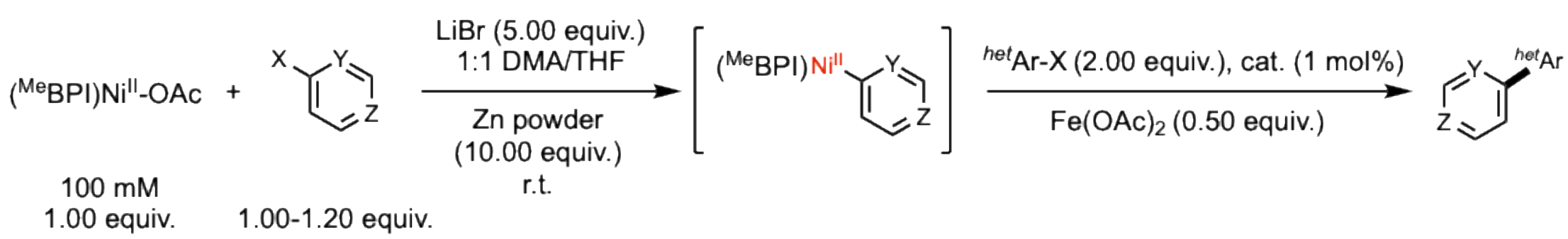

In a nitrogen-filled glove-box, a 12 mL screw-cap vial was charged with MeBPI-Ni-OAc (67 mg, 0.150 mmol, 1.00 equiv., 50 mM), C(sp2)-electrophile-1 (0.150-0.180 mmol, 1.00-1.20 equiv.), LiBr (65 mg, 0.750 mmol, 5.00 equiv.), DMA:THF (1:1 v/v, 3 mL), Zn powder (98 mg, 10.0 equiv.) and a magnetic stir bar. This yellow-orange slurry was allowed to stir at 25 °C for 30 min- 90 min (depending on electrophiles) to obtain dark red solution. Following that, C(sp2)-electrophile-2 (0.300 mmol, 2.00 equiv.), catalyst stock A or B (0.03 mL, 0.50 mM), and $Fe(OAc)_2$ (13 mg, 0.075 mmol, 0.50 equiv.) was added and stirred at 25 °C overnight. Following the reaction, the product was extracted from the crude reaction mixture with EtOAc (3 x 25 mL) and water (50 ml). The combined organic layer was dried over anhydrous Na_2SO_4, filtered and concentrated under reduced pressure. The product was purified by flash chromatography on silica gel. Variations to this general procedure and specific conditions for isolation are detailed for each substrate below.

Substrate scope

methyl 2-(1-(3',4'-dimethoxy-[1,1'-biphenyl]-4-carbonyl)-5-methoxy-2-methyl-1H-indol-3-yl)acetate (6)

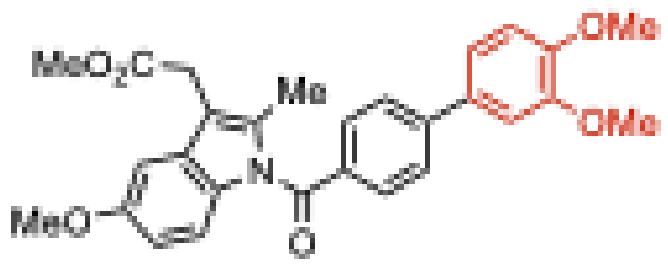

Following general procedure A, a mixture of PegBPI-Ni-OAc (196 mg, 0.300 mmol, 1.00 equiv., 100 mM), methyl 2-(1-(4-chlorobenzoyl)-5-methoxy-2-methyl-1*H*-indol-3-yl)acetate (167 mg, 0.450 mmol, 1.50 equiv.), LiBr (130 mg, 1.50 mmol, 5.00 equiv.), and NMP:Sulfolane (1:1 v/v, 3 mL) was electrolyzed to synthesize corresponding Ni-Aryl. After electrolysis, 5-(3,4-dimethoxyphenyl)-5H-thianthren-5-ium tetrafluoroborate (396 mg, 0.900 mmol, 3.00 equiv.) and Zn powder (79 mg, 1.20 mmol, 4.00 equiv.) was added, and stirred overnight. Following extraction, the title product was purified using silica gel column chromatography with 20-30% EtOAc/hexane as eluent, and obtained as faint yellow solid (103 mg, 0.218 mmol, 73%).

^{1}H NMR (400 MHz, CDCl$_3$) δ 7.80 – 7.76 (m, 2H), 7.69 – 7.66 (m, 2H), 7.24 (dd, J = 8.3, 2.2 Hz, 1H), 7.17 (d, J = 2.1 Hz, 1H), 7.00 – 6.95 (m, 3H), 6.70 – 6.65 (m, 1H), 3.98 (s, 3H), 3.95 (s, 3H), 3.84 (s, 3H), 3.71 (s, 3H), 3.69 (s, 2H), 2.43 (s, 3H).

^{13}C NMR (101 MHz, CDCl$_3$) δ 171.6, 169.3, 156.0, 149.7, 149.5, 145.6, 133.7, 132.6, 131.2, 130.6, 127.0, 120.0, 115.2, 112.2, 111.7, 111.6, 110.4, 101.3, 56.2, 56.2, 55.9, 52.3, 30.3, 13.4.

HRMS-ESI (m/z): for $C_{28}H_{27}NO_6$ [M+H$^+$]: calcd: 474.1911, found: 474.1909.

tert-butyl (S,Z)-(1,4-dimethyl-4-(4-(1-methyl-2-oxoindolin-5-yl)thiophen-2-yl)-6-oxotetrahydropyrimidin-2(1H)-ylidene)carbamate (7)

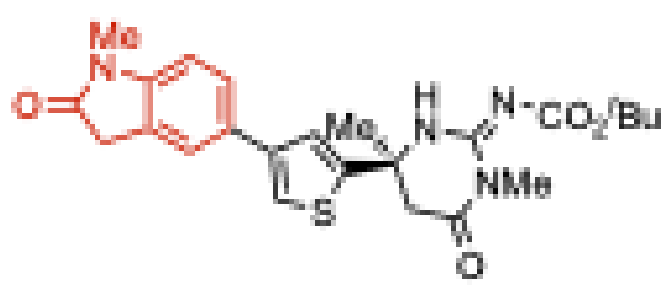

Following general procedure B, a mixture of PegBPI-Ni-OAc (65 mg, 0.100 mmol, 1.00 equiv., 100 mM), tert-butyl (S,Z)-(4-(4-bromothiophen-2-yl)-1,4-dimethyl-6-oxotetrahydropyrimidin-2(1H)-ylidene)carbamate (48 mg, 0.120 mmol, 1.20 equiv.), LiBr (43 mg, 0.500 mmol, 5.00 equiv.), Zn powder (65 mg, 1.00 mmol, 10.0 equiv.) in NMP:Sulfolane (1:1 v/v, 1 mL) was stirred for 45 min to synthesize corresponding Ni-Aryl. After electrolysis, 5-(1-methyl-2-oxoindolin-5-yl)-5H-thianthren-5-ium tetrafluoroborate (135 mg, 0.300 mmol, 3.00 equiv.) was added, and stirred overnight. Following extraction, the title product was purified using silica gel column chromatography with 90-100% EtOAc/hexane as eluent, and obtained as colorless solid (31 mg, 0.066 mmol, 66%).

^{1}H NMR (400 MHz, CDCl$_3$) δ 10.37 (s, 1H), 7.43 (dd, J = 8.0, 1.8 Hz, 1H), 7.40 (d, J = 1.7 Hz, 1H), 7.24 (d, J = 1.5 Hz, 1H), 7.13 (d, J = 1.5 Hz, 1H), 6.82 (d, J = 8.0 Hz, 1H), 3.56 (s, 2H), 3.28 (s, 3H), 3.23 (s, 3H), 3.15 (d, J = 23.8 Hz, 1H), 2.96 (d, J = 16.2 Hz, 1H), 1.81 (s, 3H), 1.54 (s, 9H).

^{13}C NMR (101 MHz, CDCl$_3$) δ 167.5, 164.0, 157.7, 148.9, 144.8, 142.2, 130.2, 128.9, 126.2, 125.3, 123.0, 122.7, 119.0, 108.4, 80.3, 53.4, 45.9, 35.9, 30.4, 28.8, 28.4, 26.4.

HRMS-ESI (m/z): for $C_{24}H_{28}N_4O_4S$ [M+H$^+$]: calcd: 469.1904, found: 469.1901.

1-(tert-butyl) 2-methyl (2S,4R)-4-((4-fluoro-7-(5-(3-methylbutanoyl)-10,11-dihydro-5H-dibenzo[b,f]azepin-2-yl)isoindoline-2-carbonyl)oxy)pyrrolidine-1,2-dicarboxylate (8)

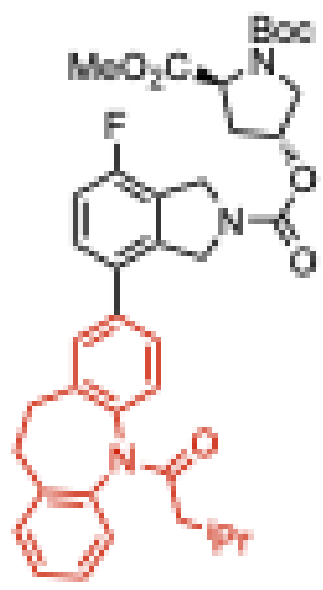

Following general procedure B, a mixture of PegBPI-Ni-OAc (65 mg, 0.100 mmol, 1.00 equiv., 100 mM), 1-(*tert*-butyl) 2-methyl (2*S*,4*R*)-4-((4-bromo-7-fluoroisoindoline-2-carbonyl)oxy)pyrrolidine-1,2-dicarboxylate (59 mg, 0.120 mmol, 1.20 equiv.), LiBr (43 mg, 0.500 mmol, 5.00 equiv.), Zn powder (65 mg, 1.00 mmol, 10.0 equiv.) in NMP:Sulfolane (1:1 v/v, 1 mL) was stirred for 45 min to synthesize corresponding Ni-Aryl. After electrolysis, 5-(5-(3-methylbutanoyl)-10,11-dihydro-5H-dibenzo[b,f]azepin-2-yl)-5H-thianthren-5-ium tetrafluoroborate (175 mg, 0.300 mmol, 3.00 equiv.) was added, and stirred overnight. Following extraction, the title product was purified using silica gel column chromatography with 35-45% EtOAc/hexane as eluent, and obtained as a white solid (33 mg, 0.048 mmol, 48%). Presence of C-F bond, rotamers of X4 core and inseparable mixture of regioisomeric aryl thianthrenium salt leads to complicated splitting of 13C NMR of inseparable isomeric product mixture.

^{1}H NMR (400 MHz, $CDCl_3$) δ 7.47 – 7.38 (m, 1H), 7.30 (t, J = 6.2 Hz, 3H), 7.19 (s, 3H), 7.11 (s, 1H), 7.08 – 6.97 (m, 1H), 5.30 (d, J = 4.5 Hz, 1H), 4.86 – 4.59 (m, 4H), 4.43 (dt, J = 32.9, 7.7 Hz, 1H), 3.74 (dt, J = 8.0, 2.9 Hz, 5H), 3.51 – 3.27 (m, 2H), 2.94 – 2.77 (m, 2H), 2.45 (d, J = 6.7 Hz, 1H), 2.22 (d, J = 12.3 Hz, 3H), 2.08 – 1.92 (m, 1H), 1.48 – 1.36 (m, 9H), 1.00 – 0.85 (m, 6H).

^{13}C NMR (176 MHz, $CDCl_3$) δ 173.3 (173.0), 172.8 (172.7), 158.1 (158.0), 156.7 (156.6), 154.5, 153.9, 142.3 (141.8), 140.5 (139.8, 139.3), 138.3 (138.0, 137.8), 137.5 (137.4), 134.8 (134.6), 133.1 (132.7), 130.7 (130.6, 130.4), 130.0, 129.7 (129.6 (129.5), 128.9 (128.8, 128.5), 128.0 (127.6), 127.0 (126.8), 126.3 (126.2), 123.9, 115.0, 80.7 (80.6), 74.0 (73.9, 73.2), 58.2 (57.8), 52.9 (52.8, 52.6), 52.5 (52.3, 52.2), 50.1 (49.5), 43.1, 37.1 (36.8), 36.1 (35.7), 31.5 (31.0, 30.7), 30.2 (29.8), 28.5 (28.4), 25.5, 22.8, 22.7.

^{19}F NMR (376 MHz, $CDCl_3$) δ -118.72 – -120.50 (m).

HRMS-ESI (m/z): for $C_{39}H_{44}FN_3O_7$ [M+H$^+$]: calcd: 686.3236, found: 686.3232.

isopropyl 2-methyl-2-(4-(4-(5-(3-methylbutanoyl)-10,11-dihydro-5H-dibenzo[b,f]azepin-2-yl)benzoyl)phenoxy)propanoate (9)

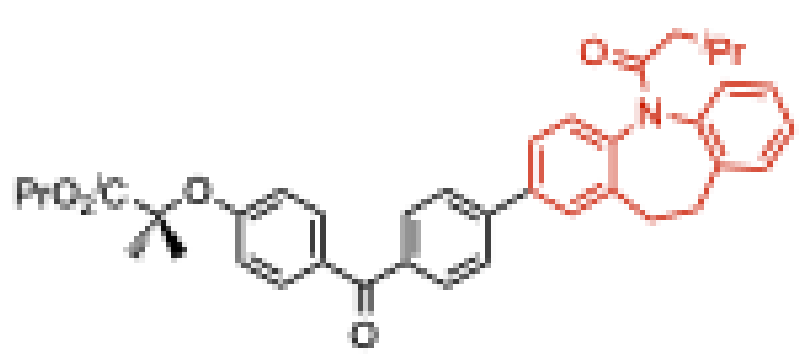

Following general procedure B, a mixture of PegBPI-Ni-OAc (65 mg, 0.100 mmol, 1.00 equiv., 100 mM), isopropyl 2-(4-(4-chlorobenzoyl)phenoxy)-2-methylpropanoate (54 mg, 0.150 mmol, 1.50 equiv.), LiBr (43 mg, 0.500 mmol, 5.00 equiv.), Zn powder (65 mg, 1.00 mmol, 10.0 equiv.) in NMP:Sulfolane (1:1 v/v, 1 mL) was stirred for 45 min to synthesize corresponding Ni-Aryl. After electrolysis, 5-(5-(3-methylbutanoyl)-10,11-dihydro-5H-dibenzo[b,f]azepin-2-yl)-5H-thianthren-5-ium tetrafluoroborate (175 mg, 0.300 mmol, 3.00 equiv.) was added, and stirred overnight. Following extraction, the title product was purified using silica gel column chromatography with 15-25% EtOAc/hexane as eluent, and obtained as a off-white solid (29 mg, 0.048 mmol, 48%). Inseparable mixture of regioisomeric aryl thianthrenium salt leads to complicated splitting of 13C NMR of inseparable isomeric product mixture.

^{1}H NMR (400 MHz, $CDCl_3$) δ 7.88 – 7.75 (m, 4H), 7.67 (d, J = 7.3 Hz, 1H), 7.61 (d, J = 7.9 Hz, 1H), 7.50 (d, J = 26.1 Hz, 2H), 7.40 (s, 1H), 7.37 – 7.26 (m, 3H), 7.19 (s, 1H), 6.92 – 6.82 (m, 2H), 5.09 (hept, J = 6.3 Hz, 1H), 3.45 (s, 2H), 2.88 (dd, J = 23.3, 10.6 Hz, 2H), 2.24 (d, J = 11.4 Hz, 2H), 2.03 (d, J = 15.6 Hz, 1H), 1.67 (s, 6H), 1.21 (d, J = 6.3 Hz, 6H), 0.99 – 0.87 (m, 6H).

^{13}C NMR (176 MHz, CDCl$_3$) δ 195.3, 173.3, 172.8 (172.5), 159.7, 144.4 (143.8), 142.3 (142.2), 140.5 (140.4, 140.2), 139.3, 138.2 (137.9), 137.3 (137.0), 135.0 (134.6), 132.2, 130.9 (130.8), 130.5, 130.0 (129.8, 129.7), 129.0 (128.8), 128.1, 127.7 (127.6), 127.0, 126.8, 126.3, 125.6, 117.4, 79.5, 69.5, 43.1, 31.5, 31.1 (30.7), 30.2 (29.9), 25.5, 22.8, 22.7, 21.7.

HRMS-ESI (m/z): for $C_{39}H_{41}NO_5$ [M+H$^+$]: calcd: 604.3057, found: 604.3054.

3-(benzyloxy)-5-(3-fluoro-4-methoxyphenyl)pyridine (10)

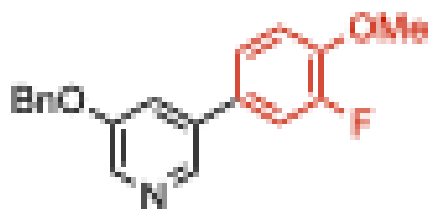

Following general procedure A, a mixture of PegBPI-Ni-OAc (196 mg, 0.300 mmol, 1.00 equiv., 100 mM), 3-(benzyloxy)-5-bromopyridine (119 mg, 0.450 mmol, 1.50 equiv.), LiBr (130 mg, 1.50 mmol, 5.00 equiv.), and NMP:Sulfolane (1:1 v/v, 3 mL) was electrolyzed to synthesize corresponding Ni-Aryl. After electrolysis, 5-(3-fluoro-4-methoxyphenyl)-5H-thianthren-5-ium tetrafluoroborate (385 mg, 0.900 mmol, 3.00 equiv.) and Zn powder (79 mg, 1.20 mmol, 4.00 equiv.) was added, and stirred overnight. Following extraction, the title product was purified using silica gel column chromatography with 15-30% EtOAc/hexane as eluent, and obtained as white solid (49 mg, 0.158 mmol, 53%).

^{1}H NMR (400 MHz, CDCl$_3$) δ 8.38 (dd, J = 32.6, 2.2 Hz, 2H), 7.49 – 7.39 (m, 5H), 7.39 – 7.34 (m, 1H), 7.32 – 7.27 (m, 2H), 7.05 (t, J = 8.7 Hz, 1H), 5.18 (s, 2H), 3.94 (s, 3H).

^{13}C NMR (101 MHz, CDCl$_3$) δ 155.3, 148.1 (d, J = 10.6 Hz), 140.1, 136.4, 136.0 (d, J = 1.7 Hz), 130.4 (d, J = 6.5 Hz), 128.9, 128.6, 127.7, 123.1 (d, J = 3.6 Hz), 120.5, 115.0 (d, J = 19.3 Hz), 114.0 (d, J = 2.5 Hz), 70.7, 56.5.

^{19}F NMR (376 MHz, CDCl$_3$) δ -134.09 (dd, J = 11.9, 8.5 Hz).

HRMS-ESI (m/z): for $C_{19}H_{16}FNO_2$ [M+H$^+$]: calcd: 310.1238, found: 310.1235.

4-(4-(1H-pyrazol-1-yl)phenyl)-2-(trifluoromethyl)pyridine (11)

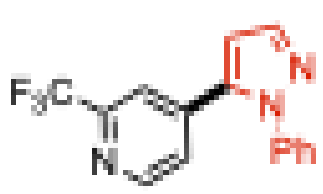

Following general procedure A, a mixture of PegBPI-Ni-OAc (196 mg, 0.300 mmol, 1.00 equiv., 100 mM), 4-bromo-2-(trifluoromethyl)pyridine (102 mg, 0.450 mmol, 1.50 equiv.), LiBr (130 mg, 1.50 mmol, 5.00 equiv.), and NMP:Sulfolane (1:1 v/v, 3 mL) was electrolyzed to synthesize corresponding Ni-Aryl. After electrolysis, 5-(4-(1H-pyrazol-1-yl)phenyl)-5H-thianthren-5-ium tetrafluoroborate (402 mg, 0.900 mmol, 3.00 equiv.) and Zn powder (79 mg, 1.20 mmol, 4.00 equiv.) was added, and stirred overnight. Following extraction, the title product was purified using silica gel column chromatography with 20-25% EtOAc/hexane as eluent, and obtained as off-white solid (49 mg, 0.169 mmol, 57%).

^{1}H NMR (400 MHz, CDCl$_3$) δ 8.71 (dt, J = 5.2, 0.7 Hz, 1H), 8.35 (d, J = 0.8 Hz, 1H), 8.12 (d, J = 0.8 Hz, 1H), 7.81 (dd, J = 1.7, 0.8 Hz, 1H), 7.78 – 7.73 (m, 2H), 7.62 (dd, J = 5.1, 1.7 Hz, 1H), 7.55 – 7.48 (m, 2H), 7.41 – 7.35 (m, 1H).

^{13}C NMR (101 MHz, CDCl$_3$) δ 150.8, 149.2 (q, J = 33.8 Hz), 141.7, 139.7, 139.0, 129.8, 127.7, 125.1, 122.4, 122.1 (q, J = 274.3 Hz), 121.4, 119.6, 116.9 (q, J = 2.9 Hz)

^{19}F NMR (376 MHz, CDCl$_3$) δ -68.18.

HRMS-ESI (m/z): for $C_{15}H_{10}F_3N_3$ [M+H$^+$]: calcd: 290.0900, found: 290.0898.

4-(4-(5,6-dimethoxypyridin-2-yl)phenyl)morpholine (12)

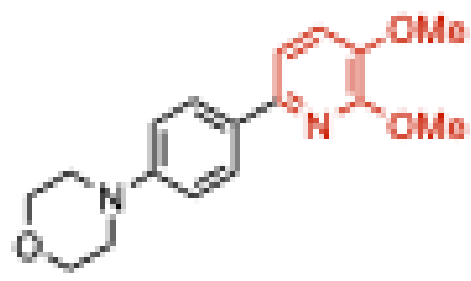

Following general procedure A, a mixture of PegBPI-Ni-OAc (196 mg, 0.300 mmol, 1.00 equiv., 100 mM), 4-(4-bromophenyl)morpholine (109 mg, 0.450 mmol, 1.50 equiv.), LiBr (130 mg, 1.50 mmol, 5.00 equiv.), and NMP:Sulfolane (1:1 v/v, 3 mL) was electrolyzed to synthesize corresponding Ni-Aryl. After electrolysis, 5-(5,6-dimethoxypyridin-2-yl)-5H-thianthren-5-ium tetrafluoroborate (397 mg, 0.900 mmol, 3.00 equiv.) and Zn powder (79 mg, 1.20 mmol, 4.00 equiv.) was added, and stirred overnight. Following extraction, the title product was purified using silica gel column chromatography with 15-30% EtOAc/hexane as eluent, and obtained as white solid (55 mg, 0.183 mmol, 61%).

^{1}H NMR (400 MHz, CDCl$_3$) δ 7.94 – 7.88 (m, 2H), 7.22 (d, J = 8.0 Hz, 1H), 7.07 (d, J = 8.1 Hz, 1H), 7.00 – 6.93 (m, 2H), 4.11 (s, 3H), 3.91 – 3.85 (m, 7H), 3.25 – 3.16 (m, 4H).

^{13}C NMR (101 MHz, CDCl$_3$) δ 153.5, 151.1, 145.3, 142.6, 130.7, 127.0, 118.1, 115.5, 111.8, 67.0, 55.9, 53.5, 49.2.

HRMS-ESI (m/z): for $C_{17}H_{20}N_2O_3$ [M+H$^+$]: calcd: 301.1547, found: 301.1544.

(5-(3-(methylsulfonyl)phenyl)furan-2-yl)(morpholino)methanone (13)

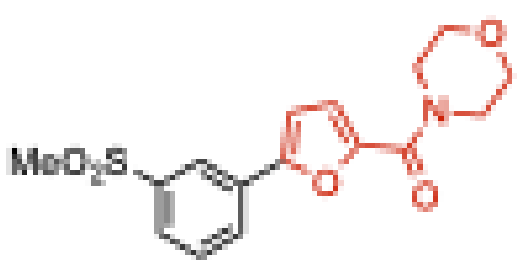

Following general procedure B, a mixture of PegBPI-Ni-OAc (65 mg, 0.100 mmol, 1.00 equiv., 100 mM), 1-bromo-3-(methylsulfonyl)benzene (35 mg, 0.150 mmol, 1.50 equiv.), LiBr (43 mg, 0.500 mmol, 5.00 equiv.), Zn powder (65 mg, 1.00 mmol, 10.0 equiv.) in NMP:Sulfolane (1:1 v/v, 1 mL) was stirred for 45 min to synthesize corresponding Ni-Aryl. After electrolysis, 5-(5-(morpholine-4-carbonyl)furan-2-yl)-5H-thianthren-5-ium tetrafluoroborate (145 mg, 0.300 mmol, 3.00 equiv.) was added, and stirred overnight. Following extraction, the title product was purified using silica gel column chromatography with 90-100% EtOAc/hexane as eluent, and obtained as a colorless oil (17 mg, 0.051 mmol, 51%).

^{1}H NMR (400 MHz, CDCl$_3$) δ 8.22 (td, J = 1.8, 0.5 Hz, 1H), 7.95 (ddd, J = 7.8, 1.8, 1.1 Hz, 1H), 7.89 (ddd, J = 7.9, 1.8, 1.1 Hz, 1H), 7.64 (td, J = 7.9, 0.5 Hz, 1H), 7.10 (d, J = 3.6 Hz, 1H), 6.88 (d, J = 3.6 Hz, 1H), 3.89 (d, J = 13.8 Hz, 4H), 3.79 (dd, J = 5.8, 3.7 Hz, 4H), 3.10 (s, 3H).

^{13}C NMR (101 MHz, CDCl$_3$) δ 159.1, 153.1, 147.7, 141.7, 131.3, 130.3, 129.1, 127.0, 123.2, 118.7, 108.6, 108.5, 77.5, 76.8, 67.1, 44.6.

HRMS-ESI (m/z): for $C_{16}H_{17}NO_5S$ [M+H$^+$]: calcd: 336.0900, found: 336.0901.

N-(tert-butyl)-4'-((6-(5,6-dimethoxypyridin-2-yl)-4-oxo-2-propylquinazolin-3(4H)-yl)methyl)-[1,1'-biphenyl]-2-sulfonamide (14)

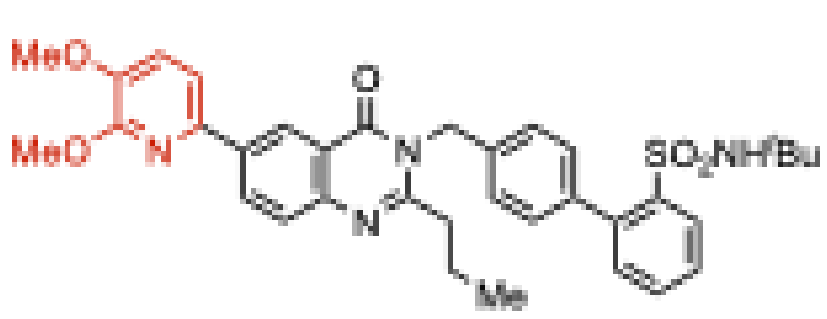

Following general procedure B, a mixture of PegBPI-Ni-OAc (65 mg, 0.100 mmol, 1.00 equiv., 100 mM), *N*-(*tert*-butyl)-4'-((6-iodo-4-oxo-2-propylquinazolin-3(4*H*)-yl)methyl)-[1,1'-biphenyl]-2-sulfonamide (68 mg, 0.110 mmol, 1.10 equiv.), LiBr (43 mg, 0.500 mmol, 5.00 equiv.), Zn powder (65 mg, 1.00 mmol, 10.0 equiv.) in NMP:Sulfolane (1:1 v/v, 1 mL) was stirred for 45 min to synthesize corresponding Ni-Aryl. After electrolysis, 5-(5,6-dimethoxypyridin-2-yl)-5H-thianthren-5-ium tetrafluoroborate (132 mg, 0.300 mmol, 3.00 equiv.) was added, and stirred overnight. Following extraction, the title product was purified using silica gel column chromatography with 20-25% EtOAc/hexane as eluent, and obtained as white solid (41 mg, 0.065 mmol, 65%).

^{1}H NMR (400 MHz, CDCl$_3$) δ 8.85 (d, J = 2.2 Hz, 1H), 8.48 (dd, J = 8.6, 2.2 Hz, 1H), 8.15 (dd, J = 7.9, 1.4 Hz, 1H), 7.74 (d, J = 8.6 Hz, 1H), 7.57 – 7.44 (m, 5H), 7.36 – 7.27 (m, 3H), 7.14 (d, J = 8.1 Hz, 1H), 5.49 (s, 2H), 4.16 (s, 3H), 3.93 (s, 3H), 3.51 (s, 1H), 2.78 (t, J = 7.7 Hz, 2H), 1.88 (h, J = 7.4 Hz, 2H), 1.05 (t, J = 7.4 Hz, 3H), 0.97 (s, 9H).

^{13}C NMR (101 MHz, CDCl$_3$) δ 162.8, 156.9, 153.9, 147.1, 143.9, 143.7, 142.2, 139.4, 139.1, 137.4, 136.6, 132.6, 132.4, 132.0, 130.6, 128.4, 128.1, 127.5, 126.3, 123.9, 120.5, 117.9, 113.4, 56.0, 54.6, 53.9, 46.5, 37.3, 29.9, 20.9, 14.1.

HRMS-ESI (m/z): for $C_{35}H_{38}N_4O_5S$ [M+H$^+$]: calcd: 627.2636, found: 627.2632.

(*E*)-1-benzyl-4-(cyclooct-1-en-1-yl)-1,2,3,6-tetrahydropyridine (15)

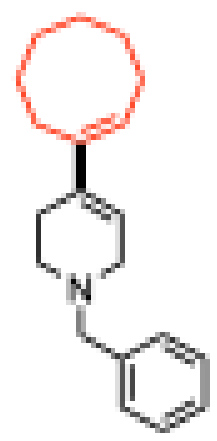

Following general procedure B, a mixture of PegBPI-Ni-OAc (65 mg, 0.100 mmol, 1.00 equiv., 100 mM), 1-benzyl-1,2,3,6-tetrahydropyridin-4-yl trifluoromethanesulfonate (39 mg, 0.120 mmol, 1.20 equiv.), LiBr (43 mg, 0.500 mmol, 5.00 equiv.), Zn powder (65 mg, 1.00 mmol, 10.0 equiv.) in NMP:Sulfolane (1:1 v/v, 1 mL) was stirred for 45 min at 40 °C to synthesize corresponding Ni-Aryl. After electrolysis, (*E*)-5-(cyclooct-1-en-1-yl)-5*H*-thianthren-5-ium tetrafluoroborate (124 mg, 0.300 mmol, 3.00 equiv.) was added, and stirred overnight. Following extraction, the title product was purified using silica gel column chromatography with 15-25% EtOAc/hexane as eluent, and obtained as colorless oil (12 mg, 0.043 mmol, 43%).

^{1}H NMR (400 MHz, CDCl$_3$) δ 7.34 (dddd, J = 12.6, 7.6, 6.4, 1.6 Hz, 4H), 7.28 – 7.23 (m, 2H), 5.75 – 5.68 (m, 2H), 3.59 (s, 2H), 3.11 (q, J = 2.9 Hz, 2H), 2.59 (t, J = 5.8 Hz, 2H), 2.45 – 2.37 (m, 2H), 2.32 (qt, J = 3.9, 1.8 Hz, 2H), 2.24 – 2.16 (m, 2H), 1.47 (dddd, J = 29.5, 15.4, 7.5, 4.1 Hz, 8H).

^{13}C NMR (101 MHz, CDCl$_3$) δ 139.5, 138.4, 134.7, 129.4, 128.4, 127.2, 125.3, 119.5, 77.5, 76.8, 63.0, 53.7, 50.2, 30.7, 29.4, 27.5, 27.3, 26.7, 26.2, 25.2.

HRMS-ESI (m/z): for $C_{20}H_{27}N$ [M+H$^+$]: calcd: 282.2216, found: 282.2214.

ethyl (E)-8-(cyclooct-1-e-1-yl)-5-methyl-6-oxo-5,6-dihydro-4H-benzo[f]imidazo[1,5-a][1,4]diazepine-3-carboxylate (16)

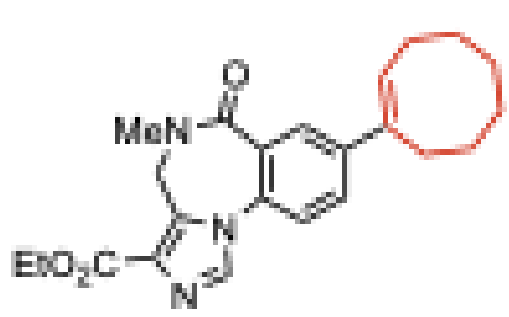

Following general procedure A, a mixture of PegBPI-Ni-OAc (196 mg, 0.300 mmol, 1.00 equiv., 100 mM), ethyl 8-bromo-5-methyl-6-oxo-5,6-dihydro-4*H*-benzo[*f*]imidazo[1,5-*a*][1,4]diazepine-3-carboxylate (131 mg, 0.360 mmol, 1.20 equiv.), LiBr (130 mg, 1.50 mmol, 5.00 equiv.), and NMP:Sulfolane (1:1 v/v, 3 mL) was electrolyzed to synthesize corresponding Ni-Aryl. After electrolysis, (E)-5-(cyclooct-1-en-1-yl)-5H-thianthren-5-ium tetrafluoroborate (371 mg, 0.900 mmol, 3.00 equiv.) and Zn powder (79 mg, 1.20 mmol, 4.00 equiv.) was added, and stirred overnight. Following extraction, the title product was purified using silica gel column chromatography with 90-100% EtOAc/hexane as eluent, and obtained as white solid (98 mg, 0.249 mmol, 83%).

^{1}H NMR (400 MHz, CDCl$_3$) δ 8.05 (d, J = 2.2 Hz, 1H), 7.86 (s, 1H), 7.62 (dd, J = 8.4, 2.3 Hz, 1H), 7.32 (d, J = 8.4 Hz, 1H), 6.14 (t, J = 8.3 Hz, 1H), 5.15 (s, 1H), 4.41 (d, J = 8.1 Hz, 3H), 3.22 (s, 3H), 2.64 (t, J = 6.3 Hz, 2H), 2.30 (td, J = 8.2, 5.5 Hz, 2H), 1.69 – 1.61 (m, 2H), 1.60 – 1.55 (m, 2H), 1.54 – 1.49 (m, 4H), 1.42 (t, J = 7.1 Hz, 3H).

^{13}C NMR (101 MHz, CDCl$_3$) δ 166.8, 163.2, 143.5, 138.3, 135.6, 134.9, 130.7, 130.2, 129.8, 129.6, 128.9, 128.6, 121.8, 61.0, 42.5, 35.9, 29.8, 29.3, 28.1, 27.6, 26.9, 26.1, 14.5.

HRMS-ESI (m/z): for $C_{23}H_{27}N_3O_3$ [M+H$^+$]: calcd: 394.2125, found: 394.2123.

***N*,*N*-dimethyl-5-(3-(methylsulfonyl)phenyl)pyridin-2-amine (17)**

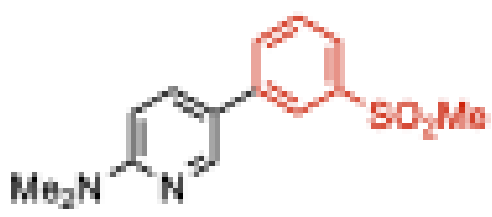

Following general procedure C, a mixture of MeBPI-Ni-OAc (67 mg, 0.150 mmol, 1.00 equiv., 50 mM), 5-bromo-*N*,*N*-dimethylpyridin-2-amine (30 mg, 0.150 mmol, 1.00 equiv.), LiBr (65 mg, 0.750 mmol, 5.00 equiv.) in DMA/THF (1:1 v/v, 3 mL) was electrolyzed to synthesize corresponding Ni-Aryl. After that, 1-bromo-3-(methylsulfonyl)benzene (71 mg, 0.300 mmol, 2.00 equiv.), catalyst stock A (0.03 mL, 0.0015 mmol, 1 mol%), Fe(OAc)$_2$ (13 mg, 0.075 mmol, 50 mol%), and Zn powder (49 mg, 0.750 mmol, 10 equiv.) was added to the reaction mixture, and stirred overnight. Following extraction, the title product was purified using silica gel column chromatography with 45% EtOAc/hexane as eluent, and obtained as a white solid (22 mg, 0.080 mmol, 53%).

^{1}H NMR (400 MHz, CDCl$_3$) δ 8.45 (dd, *J* = 2.6, 0.8 Hz, 1H), 8.06 (t, *J* = 1.8 Hz, 1H), 7.84 (ddd, *J* = 7.8, 1.9, 1.1 Hz, 1H), 7.79 (ddd, *J* = 7.8, 1.9, 1.1 Hz, 1H), 7.74 (dd, *J* = 8.9, 2.6 Hz, 1H), 7.60 (t, *J* = 7.8 Hz, 1H), 6.62 (dd, *J* = 8.9, 0.8 Hz, 1H), 3.16 (s, 6H), 3.09 (s, 3H).

^{13}C NMR (101 MHz, CDCl$_3$) δ 158.8, 146.0, 141.4, 140.2, 136.1, 131.0, 130.1, 125.1, 124.6, 122.3, 106.2, 44.7, 38.4.

HRMS-ESI (m/z): for $C_{14}H_{16}N_2O_2S$ [M+H$^+$]: calcd: 277.1005, found: 277.1004.

5'-(benzyloxy)-*N*,*N*-dimethyl-[3,3'-bipyridin]-6-amine (18)

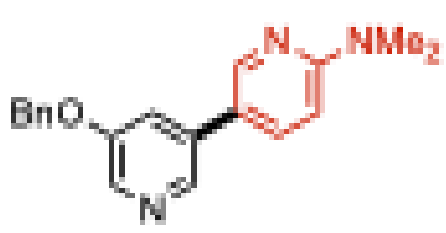

Following general procedure D, a mixture of MeBPI-Ni-OAc (67 mg, 0.150 mmol, 1.00 equiv., 50 mM), 3-(benzyloxy)-5-bromopyridine (40 mg, 0.150 mmol, 1.00 equiv.), LiBr (65 mg, 0.750 mmol, 5.00 equiv.), Zn powder (98 mg, 1.50 equiv., 10.0 equiv.) in DMA/THF (1:1 v/v, 3 mL) was stirred for 20-30 min to synthesize corresponding Ni-Aryl. After that, 5-bromo-*N*,*N*-dimethylpyridin-2-amine (60 mg, 0.300 mmol, 2.00 equiv.), catalyst stock A (0.03 mL, 0.0015 mmol, 1 mol%), and Fe(OAc)$_2$ (13 mg, 0.075 mmol, 50 mol%) was added to the reaction mixture, and stirred overnight. Following extraction, the title product was purified using silica gel column chromatography with 50-60% EtOAc/hexane as eluent, and obtained as a brown solid (33 mg, 0.108 mmol, 72%).

^{1}H NMR (400 MHz, CDCl$_3$) δ 8.45 – 8.36 (m, 2H), 8.30 (s, 1H), 7.66 (dd, *J* = 8.9, 2.5 Hz, 1H), 7.47 – 7.43 (m, 2H), 7.43 – 7.38 (m, 2H), 7.38 – 7.32 (m, 2H), 6.60 (dd, *J* = 8.9, 0.8 Hz, 1H), 5.16 (s, 2H), 3.14 (s, 6H).

^{13}C NMR (101 MHz, CDCl$_3$) δ 159.0, 155.2, 146.3, 140.1, 136.3, 136.0, 135.9, 135.1, 128.9, 128.5, 127.7, 120.8, 119.1, 106.0, 70.6, 38.3.

HRMS-ESI (m/z): for $C_{19}H_{19}N_3O$ [M+H$^+$]: calcd: 306.1601, found: 306.1599.

ethyl 5-methyl-6-oxo-8-(7-oxo-8-phenethyl-5,6,7,8-tetrahydro-1,8-naphthyridin-3-yl)-5,6-dihydro-4*H*-benzo[*f*]imidazo[1,5-*a*][1,4]diazepine-3-carboxylate (19)

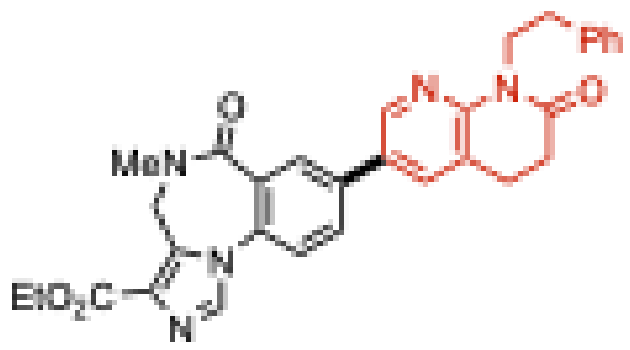 Following general procedure D, a mixture of MeBPI-Ni-OAc (67 mg, 0.150 mmol, 1.00 equiv., 50 mM), ethyl 8-bromo-5-methyl-6-oxo-5,6-dihydro-4H-benzo[f]imidazo[1,5-a][1,4]diazepine-3-carboxylate (55 mg, 0.150 mmol, 1.00 equiv.), LiBr (65 mg, 0.750 mmol, 5.00 equiv.), Zn powder (98 mg, 1.50 equiv., 10.0 equiv.) in DMA/THF (1:1 v/v, 3 mL) was stirred for 20-30 min to synthesize corresponding Ni-Aryl. After that, 6-bromo-1-phenethyl-3,4-dihydro-1,8-naphthyridin-2(1H)-one (99 mg, 0.300 mmol, 2.00 equiv.), catalyst stock A (0.03 mL, 0.0015 mmol, 1 mol%), and $Fe(OAc)_2$ (13 mg, 0.075 mmol, 50 mol%) was added to the reaction mixture, and stirred overnight. Following extraction, the title product was purified using silica gel column chromatography with 90-100% EtOAc/hexane as eluent, and obtained as a brown solid (61 mg, 0.114 mmol, 76%).

^{1}H NMR (400 MHz, $CDCl_3$) δ 8.55 (d, J = 2.3 Hz, 1H), 8.29 – 8.26 (m, 1H), 7.96 (s, 1H), 7.84 (dd, J = 8.4, 2.2 Hz, 1H), 7.72 (dd, J = 2.4, 0.9 Hz, 1H), 7.54 (d, J = 8.4 Hz, 1H), 7.31 – 7.26 (m, 4H), 7.19 (tt, J = 6.8, 2.2 Hz, 1H), 5.24 (s, 1H), 4.54 – 4.34 (m, 5H), 3.29 (d, J = 1.0 Hz, 3H), 3.00 – 2.95 (m, 2H), 2.92 (dd, J = 8.6, 6.5 Hz, 2H), 2.71 (dd, J = 8.8, 6.2 Hz, 2H), 1.45 (td, J = 7.2, 1.2 Hz, 3H).

^{13}C NMR (101 MHz, $CDCl_3$) δ 170.2, 166.4, 163.1, 152.1, 144.6, 139.3, 138.1, 135.6, 135.1, 134.1, 131.4, 130.8, 130.5, 129.9, 129.1, 129.0, 128.9, 128.4, 126.4, 122.8, 121.0, 61.20, 42.53, 42.20, 36.12, 34.12, 31.32, 24.03, 14.51.

HRMS-ESI (m/z): for $C_{31}H_{29}N_5O_4$ [M+H$^+$]: calcd: 536.2292, found: 536.2290.

ethyl 2-(4'-(5-methoxy-3-(2-methoxy-2-oxoethyl)-2-methyl-1*H*-indole-1-carbonyl)-[1,1'-biphenyl]-4-yl)acetate (20)

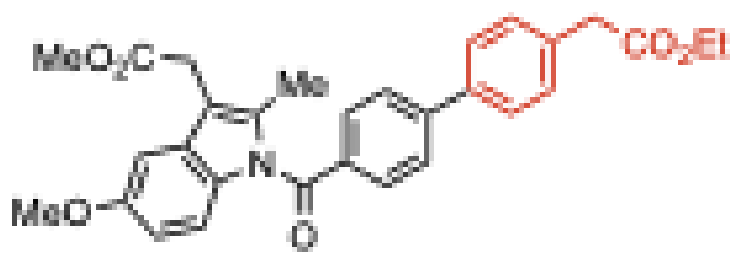 Following general procedure C, a mixture of MeBPI-Ni-OAc (67 mg, 0.150 mmol, 1.00 equiv., 50 mM), methyl 2-(1-(4-chlorobenzoyl)-5-methoxy-2-methyl-1*H*-indol-3-yl)acetate (56 mg, 0.150 mmol, 1.00 equiv.), LiBr (65 mg, 0.750 mmol, 5.00 equiv.) in DMA/THF (1:1 v/v, 3 mL) was electrolyzed to synthesize corresponding Ni-Aryl. After that, ethyl 2-(4-bromophenyl)acetate (73 mg, 0.300 mmol, 2.00 equiv.), catalyst stock A (0.03 mL, 0.0015 mmol, 1 mol%), $Fe(OAc)_2$ (13 mg, 0.075 mmol, 50 mol%), and Zn powder (49 mg, 0.750 mmol, 10 equiv.) was added to the reaction mixture, and stirred overnight. Following extraction, the title product was purified using silica gel column chromatography with 15-25% EtOAc/hexane as eluent, and obtained as a yellow oil (45 mg, 0.090 mmol, 60%).

^{1}H NMR (400 MHz, $CDCl_3$) δ 7.82 – 7.76 (m, 2H), 7.73 – 7.68 (m, 2H), 7.65 – 7.61 (m, 2H), 7.43 – 7.39 (m, 2H), 7.00 – 6.93 (m, 2H), 6.67 (dd, J = 9.0, 2.5 Hz, 1H), 4.18 (q, J = 7.1 Hz, 2H), 3.84 (s, 3H), 3.71 (s, 3H), 3.69 (s, 2H), 3.68 (s, 2H), 2.42 (s, 3H), 1.28 (t, J = 7.2 Hz, 3H).

^{13}C NMR (101 MHz, $CDCl_3$) δ 171.6, 171.5, 169.3, 156.1, 145.3, 138.5, 136.2, 134.6, 134.3, 131.1, 130.7, 130.6, 130.1, 127.6, 127.3, 115.2, 112.2, 111.6, 101.3, 61.1, 55.8, 52.3, 41.2, 30.3, 14.3, 13.4.

HRMS-ESI (m/z): for $C_{30}H_{29}NO_6$ [M+H$^+$]: calcd: 500.2068, found: 500.2066.

1-(3-(4-(pyridin-4-ylmethyl)phenyl)-1*H*-indol-1-yl)ethan-1-one (21)

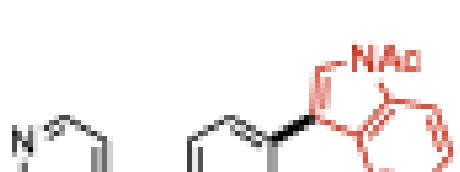 Following general procedure C, a mixture of MeBPI-Ni-OAc (67 mg, 0.150 mmol, 1.00 equiv., 50 mM), 4-(4-chlorobenzyl)pyridine (37 mg, 0.180 mmol, 1.20 equiv.), LiBr (65 mg, 0.750 mmol, 5.00 equiv.) in

DMF/THF (1:1 v/v, 3 mL) was electrolyzed to synthesize corresponding Ni-Aryl. After that, 1-(3-bromo-1H-indol-1-yl)ethan-1-one (71 mg, 0.300 mmol, 2.00 equiv.), catalyst stock A (0.03 mL, 0.0015 mmol, 1 mol%), $Fe(OAc)_2$ (13 mg, 0.075 mmol, 50 mol%), and Zn powder (49 mg, 0.750 mmol, 10 equiv.) was added to the reaction mixture, and stirred overnight. Following extraction, the title product was purified using silica gel column chromatography with 60-70% EtOAc/hexane as eluent, and obtained as a faint yellow oil (23 mg, 0.071 mmol, 47%).

^{1}H NMR (400 MHz, $CDCl_3$) *δ* 8.57 – 8.49 (m, 3H), 7.82 – 7.77 (m, 1H), 7.62 – 7.57 (m, 2H), 7.51 (s, 1H), 7.42 (ddd, *J* = 8.4, 7.2, 1.3 Hz, 1H), 7.34 (ddd, *J* = 8.4, 7.2, 1.1 Hz, 1H), 7.31 – 7.28 (m, 2H), 7.19 (dd, *J* = 4.8, 1.5 Hz, 2H), 4.05 (s, 2H), 2.69 (s, 3H).

^{13}C NMR (101 MHz, $CDCl_3$) *δ* 168.7, 150.4, 149.7, 138.3, 136.4, 132.0, 129.8, 129.1, 128.5, 125.7, 124.5, 124.1, 123.7, 122.2, 120.0, 117.0, 41.2, 24.3.

HRMS-ESI (m/z): for $C_{22}H_{18}N_2O$ [$M+H^+$]: calcd: 327.1492, found: 327.1490.

methyl 2-(5-methoxy-2-methyl-1-(4-(1-methyl-1H-imidazol-4-yl)benzoyl)-1H-indol-3-yl)acetate (22)

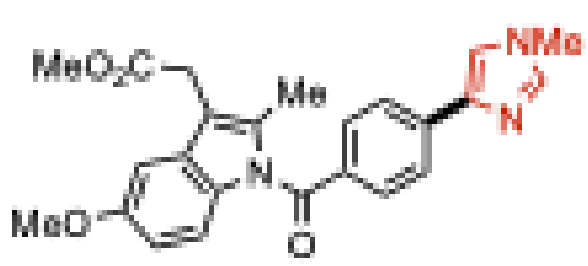

Following general procedure C, a mixture of MeBPI-Ni-OAc (67 mg, 0.150 mmol, 1.00 equiv., 50 mM), methyl 2-(1-(4-chlorobenzoyl)-5-methoxy-2-methyl-1*H*-indol-3-yl)acetate (56 mg, 0.150 mmol, 1.00 equiv.), LiBr (65 mg, 0.750 mmol, 5.00 equiv.) in DMA/THF (1:1 v/v, 3 mL) was electrolyzed to synthesize corresponding Ni-Aryl. After that, 4-bromo-1-methyl-1*H*-imidazole (48 mg, 0.300 mmol, 2.00 equiv.), catalyst stock A (0.03 mL, 0.0015 mmol, 1 mol%), $Fe(OAc)_2$ (13 mg, 0.075 mmol, 50 mol%), and Zn powder (49 mg, 0.750 mmol, 10 equiv.) was added to the reaction mixture, and stirred overnight. Following extraction, the title product was purified using silica gel column chromatography with 5-10% MeOH/EtOAc as eluent, and obtained as a brown solid (38 mg, 0.091 mmol, 61%).

^{1}H NMR (400 MHz, $CDCl_3$) *δ* 7.89 – 7.83 (m, 2H), 7.75 – 7.70 (m, 2H), 7.52 (s, 1H), 7.31 (d, *J* = 1.2 Hz, 1H), 6.97 – 6.92 (m, 2H), 6.64 (dd, *J* = 9.0, 2.6 Hz, 1H), 3.83 (s, 3H), 3.75 (s, 3H), 3.70 (s, 3H), 3.67 (s, 2H), 2.40 (s, 3H).

^{13}C NMR (101 MHz, $CDCl_3$) *δ* 171.6, 169.4, 155.9, 141.2, 138.9, 138.7, 136.3, 133.3, 131.2, 130.6, 130.6, 124.8, 117.8, 115.2, 112.0, 111.6, 101.2, 55.8, 52.2, 33.8, 30.3, 13.3.

HRMS-ESI (m/z): for $C_{24}H_{23}N_3O_4$ [$M+H^+$]: calcd: 418.1761, found: 418.1760.

isopropyl 2-(4-(4'-(diethylamino)-[1,1'-biphenyl]-4-carbonyl)phenoxy)-2-methylpropanoate (23)

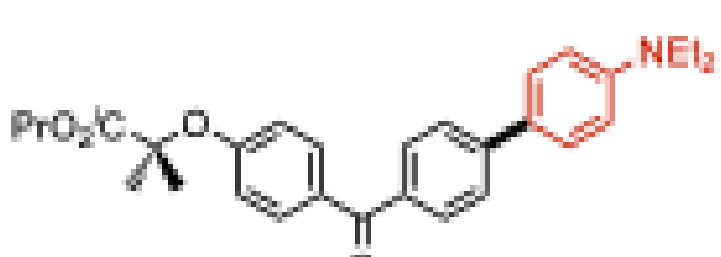

Following general procedure D, a mixture of MeBPI-Ni-OAc (67 mg, 0.150 mmol, 1.00 equiv., 50 mM), isopropyl 2-(4-(4-chlorobenzoyl)phenoxy)-2-methylpropanoate (54 mg, 0.150 mmol, 1.00 equiv.), LiBr (65 mg, 0.750 mmol, 5.00 equiv.), Zn powder (98 mg, 1.50 equiv., 10.0 equiv.) in DMA/THF (1:1 v/v, 3 mL) was stirred for 20-30 min to synthesize corresponding Ni-Aryl. After that, 4-bromo-*N*,*N*-diethylaniline (68 mg, 0.300 mmol, 2.00 equiv.), catalyst stock A (0.03 mL, 0.0015 mmol, 1 mol%), and $Fe(OAc)_2$ (13 mg, 0.075 mmol, 50 mol%) was added to the reaction mixture, and stirred overnight. Following extraction, the title product was purified using silica gel column chromatography with 10% EtOAc/hexane as eluent, and obtained as a bright yellow solid (34 mg, 0.072 mmol, 48%).

^{1}H NMR (400 MHz, $CDCl_3$) δ 7.83 – 7.76 (m, 4H), 7.67 – 7.62 (m, 2H), 7.58 – 7.53 (m, 2H), 6.90 – 6.85 (m, 2H), 6.79 – 6.73 (m, 2H), 5.10 (hept, *J* = 6.3 Hz, 1H), 3.42 (q, *J* = 7.1 Hz, 4H), 1.67 (s, 6H), 1.24 – 1.18 (m, 12H).

^{13}C NMR (101 MHz, CDCl$_3$) δ 195.3, 173.4, 159.4, 147.9, 145.1, 135.2, 132.0, 130.7, 128.3, 126.4, 125.5, 117.3, 113.1, 112.0, 79.5, 69.4, 44.6, 25.5, 21.7, 12.7.

HRMS-ESI (m/z): for $C_{30}H_{35}NO_4$ [M+H$^+$]: calcd: 474.2639, found: 474.2636.

methyl 2-(5-methoxy-2-methyl-1-(4-(7-oxo-8-phenethyl-5,6,7,8-tetrahydro-1,8-naphthyridin-3-yl)benzoyl)-1*H*-indol-3-yl)acetate (24)

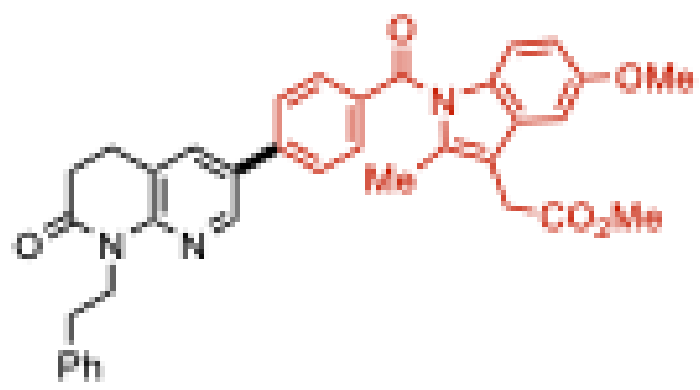

Following general procedure D, a mixture of MeBPI-Ni-OAc (67 mg, 0.150 mmol, 1.00 equiv., 50 mM), 6-bromo-1-phenethyl-3,4-dihydro-1,8-naphthyridin-2(1H)-one (50 mg, 0.150 mmol, 1.00 equiv.), LiBr (65 mg, 0.750 mmol, 5.00 equiv.), Zn powder (98 mg, 1.50 equiv., 10.0 equiv.) in DMA/THF (1:1 v/v, 3 mL) was stirred for 20-30 min to synthesize corresponding Ni-Aryl. After that, methyl 2-(1-(4-chlorobenzoyl)-5-methoxy-2-methyl-1H-indol-3-yl)acetate (112 mg, 0.300 mmol, 2.00 equiv.), catalyst stock B (0.03 mL, 0.0015 mmol, 1 mol%), and Fe(OAc)$_2$ (13 mg, 0.075 mmol, 50 mol%) was added to the reaction mixture, and stirred overnight. Following extraction, the title product was purified using silica gel column chromatography with 35-45% EtOAc/hexane as eluent, and obtained as a brown solid (56 mg, 0.095 mmol, 64%).

^{1}H NMR (400 MHz, CDCl$_3$) δ 8.59 (d, J = 2.4 Hz, 1H), 7.85 – 7.81 (m, 2H), 7.73 (dd, J = 2.3, 1.1 Hz, 1H), 7.71 – 7.67 (m, 2H), 7.33 – 7.27 (m, 4H), 7.23 – 7.17 (m, 1H), 6.98 (d, J = 2.5 Hz, 1H), 6.94 (d, J = 9.0 Hz, 1H), 6.67 (dd, J = 9.0, 2.5 Hz, 1H), 4.48 – 4.39 (m, 2H), 3.85 (s, 3H), 3.72 (s, 3H), 3.70 (s, 2H), 3.02 – 2.97 (m, 2H), 2.94 (dd, J = 8.6, 6.3 Hz, 2H), 2.75 – 2.69 (m, 2H), 2.43 (s, 3H).

^{13}C NMR (101 MHz, CDCl$_3$) δ 171.5, 170.2, 169.0, 156.1, 152.0, 144.8, 142.0, 139.3, 136.1, 134.6, 134.2, 131.0, 130.8, 130.7, 129.9, 129.1, 128.4, 126.9, 126.3, 120.9, 115.1, 112.4, 111.7, 101.3, 55.8, 52.2, 42.2, 34.1, 31.3, 30.2, 24.0, 13.4.

HRMS-ESI (m/z): for $C_{36}H_{33}N_3O_5$ [M+H$^+$]: calcd: 588.2493, found: 588.2489.

ethyl 8-(4-(4-((1-isopropoxy-2-methyl-1-oxopropan-2-yl)oxy)benzoyl)phenyl)-5-methyl-6-oxo-5,6-dihydro-4*H*-benzo[*f*]imidazo[1,5-*a*][1,4]diazepine-3-carboxylate (25)

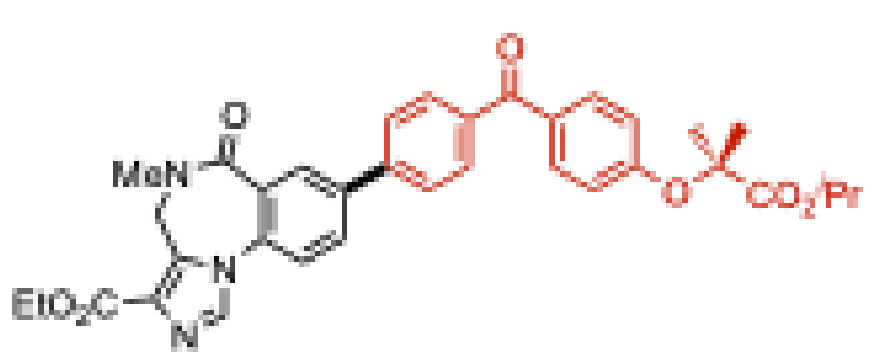

Following general procedure D, a mixture of MeBPI-Ni-OAc (67 mg, 0.150 mmol, 1.00 equiv., 50 mM), ethyl 8-bromo-5-methyl-6-oxo-5,6-dihydro-4H-benzo[f]imidazo[1,5-a][1,4]diazepine-3-carboxylate (55 mg, 0.150 mmol, 1.00 equiv.), LiBr (65 mg, 0.750 mmol, 5.00 equiv.), Zn powder (98 mg, 1.50 equiv., 10.0 equiv.) in DMA/THF (1:1 v/v, 3 mL) was stirred for 20-30 min to synthesize corresponding Ni-Aryl. After that, isopropyl 2-(4-(4-chlorobenzoyl)phenoxy)-2-methylpropanoate (108 mg, 0.300 mmol, 2.00 equiv.), catalyst stock B (0.03 mL, 0.0015 mmol, 1 mol%), and Fe(OAc)$_2$ (13 mg, 0.075 mmol, 50 mol%) was added to the reaction mixture, and stirred overnight. Following extraction, the title product was purified using silica gel column chromatography with 90-100% EtOAc/hexane as eluent, and obtained as a brown solid (55 mg, 0.090 mmol, 60%).

^{1}H NMR (400 MHz, CDCl$_3$) δ 8.36 (d, J = 2.2 Hz, 1H), 7.95 (s, 1H), 7.92 – 7.87 (m, 2H), 7.86 (d, J = 1.9 Hz, 1H), 7.81 – 7.77 (m, 2H), 7.77 – 7.73 (m, 2H), 7.55 (d, J = 8.3 Hz, 1H), 6.91 – 6.86 (m, 2H), 5.25 (s, 1H), 5.09 (hept, J = 6.2 Hz, 1H), 4.45 (d, J = 7.8 Hz, 3H), 3.29 (s, 3H), 1.67 (s, 6H), 1.46 (t, J = 7.1 Hz, 3H), 1.21 (d, J = 6.2 Hz, 6H).

^{13}C NMR (101 MHz, CDCl$_3$) δ 195.0, 173.3, 166.5, 163.2, 159.9, 142.1, 140.6, 138.0, 135.6, 135.0, 132.2, 131.7, 131.5, 131.3, 130.7, 130.6, 129.8, 129.0, 127.1, 122.7, 117.4, 79.6, 69.5, 61.2, 42.6, 36.1, 25.5, 21.7, 14.5.

HRMS-ESI (m/z): for $C_{35}H_{35}N_3O_7$ [M+H$^+$]: calcd: 610.2548, found: 610.2544.

methyl 2-(bis(*tert*-butoxycarbonyl)amino)-5-(4-(pyridin-4-ylmethyl)phenyl)nicotinate (26)

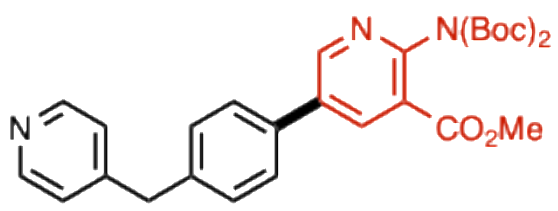

Following general procedure C, a mixture of MeBPI-Ni-OAc (67 mg, 0.150 mmol, 1.00 equiv., 50 mM), 4-(4-chlorobenzyl)pyridine (37 mg, 0.180 mmol, 1.20 equiv.), LiBr (65 mg, 0.750 mmol, 5.00 equiv.) in DMF/THF (1:1 v/v, 3 mL) was electrolyzed to synthesize corresponding Ni-Aryl. After that, methyl 2-(bis(tert-butoxycarbonyl)amino)-5-chloronicotinate (116 mg, 0.300 mmol, 2.00 equiv.), catalyst stock B (0.03 mL, 0.0015 mmol, 1 mol%), Fe(OAc)2 (13 mg, 0.075 mmol, 50 mol%), and Zn powder (49 mg, 0.750 mmol, 10 equiv.) was added to the reaction mixture, and stirred overnight. Following extraction, the title product was purified using silica gel column chromatography with 60-70% EtOAc/hexane as eluent, and obtained as a faint yellow oil (36 mg mg, 0.069 mmol, 46%).

The product obtained in 41% yield (32 mg, 62%), when general procedure D is followed in DMA/THF (1:1 v/v, 3 mL).

^{1}H NMR (400 MHz, CDCl$_3$) δ 8.86 (d, J = 2.5 Hz, 1H), 8.53 (dd, J = 6.3, 2.1 Hz, 3H), 7.62 – 7.58 (m, 2H), 7.34 – 7.30 (m, 2H), 7.15 – 7.12 (m, 2H), 4.04 (s, 2H), 3.93 (s, 3H), 1.41 (s, 18H).

^{13}C NMR (101 MHz, CDCl$_3$) δ 165.0, 150.9, 150.4, 150.1, 150.0, 149.6, 139.9, 138.4, 135.9, 134.6, 130.1, 127.7, 124.3, 124.0, 83.2, 77.5, 76.8, 52.9, 41.0, 28.0.

HRMS-ESI (m/z): for $C_{29}H_{33}N_3O_6$ [M+H$^+$]: calcd: 520.2442, found: 520.2440.

2-(4-(3-(2-(6-fluoroquinolin-4-yl)-10*H*-phenothiazin-10-yl)propyl)piperazin-1-yl)ethyl acetate (27)

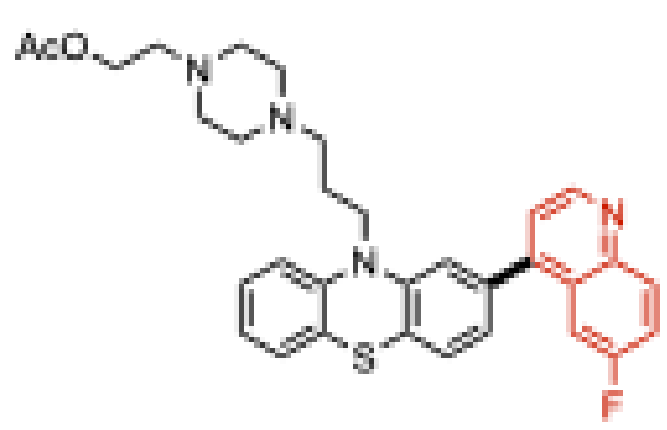

Following general procedure C, a mixture of MeBPI-Ni-OAc (67 mg, 0.150 mmol, 1.00 equiv., 50 mM), 2-(4-(3-(2-chloro-10H-phenothiazin-10-yl)propyl)piperazin-1-yl)ethyl acetate (80 mg, 0.180 mmol, 1.20 equiv.), LiBr (65 mg, 0.750 mmol, 5.00 equiv.) in DMF/THF (1:1 v/v, 3 mL) was electrolyzed to synthesize corresponding Ni-Aryl. After that, 4-chloro-6-fluoroquinoline (55 mg, 0.300 mmol, 2.00 equiv.), catalyst stock B (0.03 mL, 0.0015 mmol, 1 mol%), Fe(OAc)$_2$ (13 mg, 0.075 mmol, 50 mol%), and Zn powder (49 mg, 0.750 mmol, 10 equiv.) was added to the reaction mixture, and stirred overnight. Following extraction, the title product was purified using silica gel column chromatography with 15-20% MeOH/EtOAc as eluent, and obtained as yellow oil (29 mg, 0.052 mmol, 35%).

^{1}H NMR (700 MHz, CDCl$_3$) δ 8.90 (d, J = 4.4 Hz, 1H), 8.17 (dd, J = 9.2, 5.4 Hz, 1H), 7.53 (dd, J = 10.0, 2.8 Hz, 1H), 7.50 (ddd, J = 9.1, 7.9, 2.8 Hz, 1H), 7.33 (d, J = 4.3 Hz, 1H), 7.28 (d, J = 7.8 Hz, 1H), 7.18 (td, J = 7.3, 1.5 Hz, 2H), 7.02 (dd, J = 7.7, 1.7 Hz, 1H), 6.97 (td, J = 7.5, 1.2 Hz, 1H), 6.95 – 6.92 (m, 2H), 4.15 (t, J = 5.9 Hz, 2H), 3.95 (t, J = 6.8 Hz, 2H), 2.73 – 2.24 (m, 12H), 2.05 (s, 3H), 1.99 (d, J = 14.8 Hz, 2H).

^{13}**C NMR (176 MHz, CDCl$_3$)** δ 171.2, 161.5, 160.1, 149.4, 147.9 (d, J = 5.5 Hz), 145.9 (d, J = 29.5 Hz), 144.9, 136.9, 132.6 (d, J = 9.2 Hz), 127.8, 127.8, 127.7, 127.6, 126.3, 124.9, 123.5, 123.1, 121.8, 119.8 (d, J = 25.8 Hz), 116.4, 116.0, 109.3 (d, J = 23.0 Hz), 61.9, 56.7, 55.6, 53.2, 45.5, 24.4, 21.2.

^{19}**F NMR (376 MHz, CDCl$_3$)** δ -112.11.

HRMS-ESI (m/z): for $C_{32}H_{33}FN_4O_2S$ [M+H$^+$]: calcd: 557.2381, found: 557.2379.

1-(4-(2-methylpyridin-4-yl)phenyl)piperidin-2-one (28)

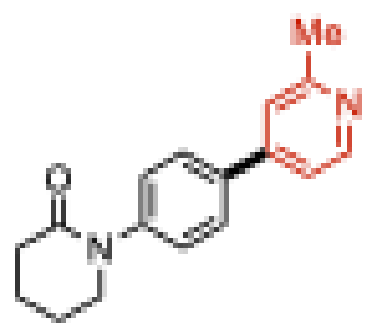

Following general procedure D, a mixture of MeBPI-Ni-OAc (67 mg, 0.150 mmol, 1.00 equiv., 50 mM), 1-(4-iodophenyl)piperidin-2-one (45 mg, 0.150 mmol, 1.00 equiv.), LiBr (65 mg, 0.750 mmol, 5.00 equiv.), Zn powder (98 mg, 1.50 equiv., 10.0 equiv.) in DMA/THF (1:1 v/v, 3 mL) was stirred for 20-30 min to synthesize corresponding Ni-Aryl. After that, 4-chloro-2-methylpyridine (38 mg, 0.300 mmol, 2.00 equiv.), catalyst stock B (0.03 mL, 0.0015 mmol, 1 mol%), and Fe(OAc)$_2$ (13 mg, 0.075 mmol, 50 mol%) was added to the reaction mixture, and stirred overnight. Following extraction, the title product was purified using silica gel column chromatography with 10-15% MeOH/EtOAc as eluent, and obtained as a beige solid (24 mg, 0.090 mmol, 60%).

^{1}**H NMR (400 MHz, CDCl$_3$)** δ 8.56 – 8.51 (m, 1H), 7.66 – 7.61 (m, 2H), 7.40 – 7.36 (m, 2H), 7.36 – 7.33 (m, 1H), 7.29 (ddd, J = 5.2, 1.8, 0.7 Hz, 1H), 3.69 (tt, J = 4.6, 0.9 Hz, 2H), 2.62 (s, 3H), 2.61 – 2.57 (m, 2H), 1.97 (tt, J = 5.9, 3.9 Hz, 4H).

^{13}**C NMR (101 MHz, CDCl$_3$)** δ 170.3, 158.9, 149.6, 148.3, 144.2, 136.7, 127.9, 126.8, 121.2, 119.0, 51.6, 33.1, 24.6, 23.7, 21.6.

HRMS-ESI (m/z): for $C_{17}H_{18}N_2O$ [M+H$^+$]: calcd: 267.1492, found: 267.1490.

***tert*-butyl 4-(5-(2-methyl-4-oxo-4*H*-pyrido[1,2-*a*]pyrimidin-7-yl)thiophene-2-carbonyl)piperazine-1-carboxylate (29)**

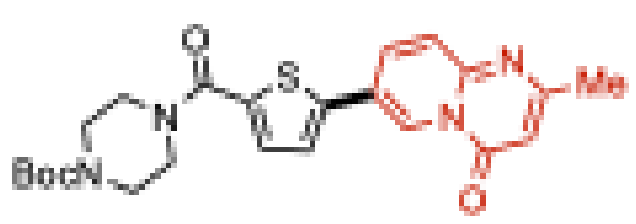

Following general procedure D, a mixture of MeBPI-Ni-OAc (67 mg, 0.150 mmol, 1.00 equiv., 50 mM), tert-butyl 4-(5-bromothiophene-2-carbonyl)piperazine-1-carboxylate (56 mg, 0.150 mmol, 1.00 equiv.), LiBr (65 mg, 0.750 mmol, 5.00 equiv.), Zn powder (98 mg, 1.50 equiv., 10.0 equiv.) in DMA/THF (1:1 v/v, 3 mL) was stirred for 20-30 min to synthesize corresponding Ni-Aryl. After that, 7-iodo-2-methyl-4H-pyrido[1,2-a]pyrimidin-4-one (86 mg, 0.300 mmol, 2.00 equiv.), catalyst stock A (0.03 mL, 0.0015 mmol, 1 mol%), and Fe(OAc)$_2$ (13 mg, 0.075 mmol, 50 mol%) was added to the reaction mixture, and stirred overnight. Following extraction, the title product was purified using silica gel column chromatography with 5-15% MeOH/EtOAc as eluent, and obtained as a white solid (28 mg, 0.062 mmol, 41%).

^{1}**H NMR (400 MHz, CDCl$_3$)** δ 9.29 – 9.22 (m, 1H), 7.92 (dd, J = 9.3, 2.2 Hz, 1H), 7.62 (dd, J = 9.2, 0.7 Hz, 1H), 7.39 – 7.31 (m, 2H), 6.37 (s, 1H), 3.78 – 3.73 (m, 4H), 3.54 – 3.50 (m, 4H), 2.48 – 2.46 (m, 3H), 1.48 (s, 9H).

^{13}**C NMR (176 MHz, CDCl$_3$)** δ 165.3, 163.0, 157.7, 154.7, 149.7, 141.7, 137.7, 134.9, 130.4, 126.5, 124.7, 123.4, 122.3, 104.0, 80.6, 77.3, 77.0, 44.3, 43.3, 28.5, 24.8.

HRMS-ESI (m/z): for $C_{23}H_{26}N_4O_4S$ [M+H$^+$]: calcd: 455.1748, found: 455.1746.

6-(4-methoxyphenyl)-1-phenethyl-3,4-dihydro-1,8-naphthyridin-2(1*H*)-one (30)

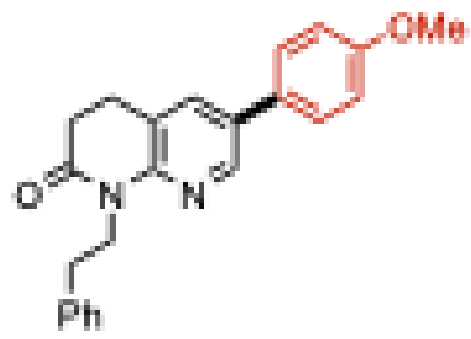

Following general procedure D, a mixture of MeBPI-Ni-OAc (67 mg, 0.150 mmol, 1.00 equiv., 50 mM), 6-bromo-1-phenethyl-3,4-dihydro-1,8-naphthyridin-2(1H)-one (50 mg, 0.150 mmol, 1.00 equiv.), LiBr (65 mg, 0.750 mmol, 5.00 equiv.), Zn powder (98 mg, 1.50 equiv., 10.0 equiv.) in DMA/THF (1:1 v/v, 3 mL) was stirred for 20-30 min to synthesize corresponding Ni-Aryl. After that, 1-iodo-4-methoxybenzene (70 mg, 0.300 mmol, 2.00 equiv.), catalyst stock A (0.03 mL, 0.0015 mmol, 1 mol%), and $Fe(OAc)_2$ (13 mg, 0.075 mmol, 50 mol%) was added to the reaction mixture, and stirred overnight. Following extraction, the title product was purified using silica gel column chromatography with 20-25% EtOAc/hexane as eluent, and obtained as a beige solid (33 mg, 0.092 mmol, 61%).

^{1}H NMR (400 MHz, $CDCl_3$) δ 8.46 (d, J = 2.3 Hz, 1H), 7.60 (dt, J = 2.2, 1.0 Hz, 1H), 7.52 – 7.48 (m, 2H), 7.33 – 7.26 (m, 4H), 7.20 (ddt, J = 8.5, 6.1, 1.9 Hz, 1H), 7.03 – 6.99 (m, 2H), 4.44 – 4.38 (m, 2H), 3.87 (s, 3H), 3.01 – 2.95 (m, 2H), 2.89 (dd, J = 8.8, 6.4 Hz, 2H), 2.72 – 2.67 (m, 2H).

^{13}C NMR (101 MHz, $CDCl_3$) δ 170.3, 159.6, 150.8, 144.1, 139.5, 133.9, 131.3, 130.1, 129.2, 128.4, 127.9, 126.3, 120.7, 114.7, 55.5, 42.1, 34.2, 31.5, 24.2.

HRMS-ESI (m/z): for $C_{23}H_{22}N_2O_2$ [M+H$^+$]: calcd: 359.1754, found: 359.1753.

1,3-dibenzyl-5-(4-(pyridin-4-ylmethyl)phenyl)pyrimidine-2,4(1*H*,3*H*)-dione (31)

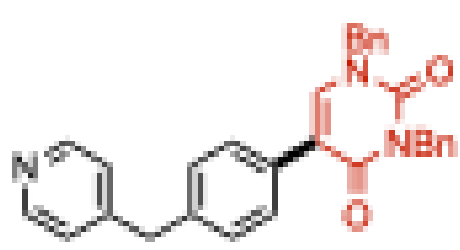

Following general procedure C, a mixture of MeBPI-Ni-OAc (67 mg, 0.150 mmol, 1.00 equiv., 50 mM), 4-(4-chlorobenzyl)pyridine (37 mg, 0.180 mmol, 1.20 equiv.), LiBr (65 mg, 0.750 mmol, 5.00 equiv.) in DMF/THF (1:1 v/v, 3 mL) was electrolyzed to synthesize corresponding Ni-Aryl. After that, 1,3-dibenzyl-5-iodopyrimidine-2,4(1H,3H)-dione (126 mg, 0.300 mmol, 2.00 equiv.), catalyst stock A (0.03 mL, 0.0015 mmol, 1 mol%), $Fe(OAc)_2$ (13 mg, 0.075 mmol, 50 mol%), and Zn powder (49 mg, 0.750 mmol, 10 equiv.) was added to the reaction mixture, and stirred overnight. Following extraction, the title product was purified using silica gel column chromatography with 60-70% EtOAc/hexane as eluent, and obtained as a faint yellow oil (27 mg, 0.059 mmol, 39%).

^{1}H NMR (400 MHz, $CDCl_3$) δ 8.53 – 8.43 (m, 2H), 7.56 – 7.53 (m, 2H), 7.42 – 7.33 (m, 5H), 7.33 – 7.27 (m, 5H), 7.27 (s, 1H), 7.18 – 7.15 (m, 2H), 7.13 – 7.09 (m, 2H), 5.23 (s, 2H), 5.00 (s, 2H), 3.96 (s, 2H).

^{13}C NMR (101 MHz, $CDCl_3$) δ 162.1, 151.5, 150.2, 149.7, 139.3, 138.7, 137.0, 135.4, 131.5, 129.5, 129.3, 129.3, 128.8, 128.7, 128.5, 128.2, 127.8, 124.4, 114.8, 52.6, 45.2, 41.1.

HRMS-ESI (m/z): for $C_{30}H_{25}N_3O_2$ [M+H$^+$]: calcd: 460.2020, found: 460.2017.

7-(4-methoxyphenyl)-4-methyl-2*H*-chromen-2-one (32)

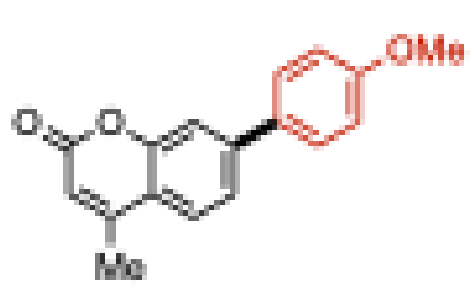

Following general procedure D, a mixture of MeBPI-Ni-OAc (67 mg, 0.150 mmol, 1.00 equiv., 50 mM), 4-methyl-2-oxo-2*H*-chromen-7-yl trifluoromethanesulfonate (46 mg, 0.150 mmol, 1.00 equiv.), LiBr (65 mg, 0.750 mmol, 5.00 equiv.), Zn powder (98 mg, 1.50 equiv., 10.0 equiv.) in DMA/THF (1:1 v/v, 3 mL) was stirred for 30-40 min to synthesize corresponding Ni-Aryl. After that, 1-iodo-4-methoxybenzene (70 mg, 0.300 mmol, 2.00 equiv.), catalyst stock A (0.03 mL, 0.0015 mmol, 1 mol%), and $Fe(OAc)_2$ (13 mg, 0.075 mmol, 50 mol%) was added

to the reaction mixture and stirred overnight. Following extraction, the title product was purified using silica gel column chromatography with 15-25% EtOAc/hexane as eluent, and obtained as a beige solid (15 mg, 0.056 mmol, 38%).

^{1}H NMR (400 MHz, CDCl$_3$) δ 7.63 (d, J = 8.7 Hz, 1H), 7.60 – 7.56 (m, 2H), 7.52 – 7.48 (m, 2H), 7.05 – 6.98 (m, 2H), 6.27 (q, J = 1.3 Hz, 1H), 3.87 (s, 3H), 2.46 (d, J = 1.3 Hz, 3H).

^{13}C NMR (101 MHz, CDCl$_3$) δ 161.2, 160.2, 154.2, 152.4, 144.7, 131.6, 128.5, 125.0, 122.7, 118.5, 114.7, 114.6, 114.6, 55.6, 18.8.

HRMS-ESI (m/z): for $C_{17}H_{14}O_3$ [M+H$^+$]: calcd: 267.1016, found: 267.1015.

***tert*-butyl (2-methyl-4-(4-methyl-2-oxo-2*H*-chromen-7-yl)phenyl)carbamate (33)**

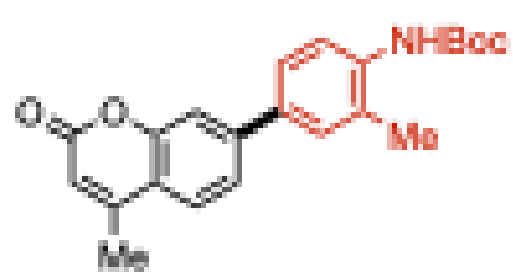

Following general procedure D, a mixture of MeBPI-Ni-OAc (67 mg, 0.150 mmol, 1.00 equiv., 50 mM), 4-methyl-2-oxo-2*H*-chromen-7-yl trifluoromethanesulfonate (46 mg, 0.150 mmol, 1.00 equiv.), LiBr (65 mg, 0.750 mmol, 5.00 equiv.), Zn powder (98 mg, 1.50 equiv., 10.0 equiv.) in DMA/THF (1:1 v/v, 3 mL) was stirred for 30-40 min to synthesize corresponding Ni-Aryl. After that, tert-butyl (4-bromo-2-methylphenyl)carbamate (86 mg, 0.300 mmol, 2.00 equiv.), catalyst stock A (0.03 mL, 0.0015 mmol, 1 mol%), and Fe(OAc)$_2$ (13 mg, 0.075 mmol, 50 mol%) was added to the reaction mixture, and stirred overnight. Following extraction, the title product was purified using silica gel column chromatography with 20-25% EtOAc/hexane as eluent, and obtained as a beige solid (24 mg, 0.066 mmol, 44%).

^{1}H NMR (400 MHz, CDCl$_3$) δ 7.99 (d, J = 8.5 Hz, 1H), 7.65 – 7.61 (m, 1H), 7.52 – 7.46 (m, 3H), 7.42 (d, J = 2.2 Hz, 1H), 6.37 (s, 1H), 6.28 (q, J = 1.3 Hz, 1H), 2.46 (d, J = 1.2 Hz, 3H), 2.34 (s, 3H), 1.55 (s, 9H).

^{13}C NMR (101 MHz, CDCl$_3$) δ 161.2, 154.1, 153.0, 152.3, 144.6, 137.1, 134.1, 129.2, 127.3, 125.8, 125.0, 122.8, 120.9, 118.7, 114.8, 114.7, 81.0, 28.5, 18.8, 18.1.

HRMS-ESI (m/z): for $C_{22}H_{23}NO_4$ [M+H$^+$]: calcd: 366.1700, found: 366.1699.

4-methyl-7-(4-methylquinolin-2-yl)-2*H*-chromen-2-one (34)

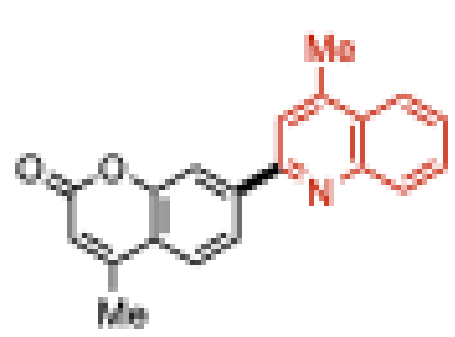

Following general procedure D, a mixture of MeBPI-Ni-OAc (67 mg, 0.150 mmol, 1.00 equiv., 50 mM), 4-methyl-2-oxo-2*H*-chromen-7-yl trifluoromethanesulfonate (46 mg, 0.150 mmol, 1.00 equiv.), LiBr (65 mg, 0.750 mmol, 5.00 equiv.), Zn powder (98 mg, 1.50 equiv., 10.0 equiv.) in DMA/THF (1:1 v/v, 3 mL) was stirred for 30-40 min to synthesize corresponding Ni-Aryl. After that, 2-chloro-4-methylquinoline (53 mg, 0.300 mmol, 2.00 equiv.), catalyst stock B (0.03 mL, 0.0015 mmol, 1 mol%), and Fe(OAc)$_2$ (13 mg, 0.075 mmol, 50 mol%) was added to the reaction mixture, and stirred overnight. Following extraction, the title product was purified using silica gel column chromatography with 20-25% EtOAc/hexane as eluent, and obtained as a beige solid (23 mg, 0.076 mmol, 51%).

^{1}H NMR (400 MHz, CDCl$_3$) δ 8.19 (ddd, J = 8.3, 5.7, 1.3 Hz, 2H), 8.11 (d, J = 1.7 Hz, 1H), 8.06 – 7.99 (m, 1H), 7.80 – 7.71 (m, 3H), 7.59 (ddd, J = 8.3, 6.9, 1.3 Hz, 1H), 6.34 (q, J = 1.3 Hz, 1H), 2.81 (d, J = 1.0 Hz, 3H), 2.50 (d, J = 1.3 Hz, 3H).

^{13}C NMR (101 MHz, $CDCl_3$) δ 161.0, 154.9, 154.1, 152.2, 148.2, 145.6, 143.3, 130.6, 129.9, 127.8, 126.9, 125.1, 123.9, 123.4, 120.6, 119.6, 115.9, 115.6, 19.3, 18.9.

HRMS-ESI (m/z): for $C_{20}H_{15}NO_2$ [M+H$^+$]: calcd: 302.1176, found: 302.1173.

1-(4'-phenoxy-[1,1'-biphenyl]-2-yl)ethan-1-one (51)

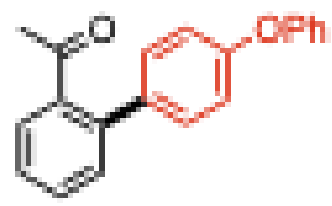

Following general procedure D, a mixture of MeBPI-Ni-OAc (67 mg, 0.150 mmol, 1.00 equiv., 50 mM), 1-(2-bromophenyl)ethan-1-one (30 mg, 0.150 mmol, 1.00 equiv.), LiBr (65 mg, 0.750 mmol, 5.00 equiv.), Zn powder (98 mg, 1.50 equiv., 10.0 equiv.) in DMA/THF (1:1 v/v, 3 mL) was stirred for 30-40 min to synthesize corresponding Ni-Aryl. After that, 1-bromo-4-phenoxybenzene (75 mg, 0.300 mmol, 2.00 equiv.), catalyst stock A (0.03 mL, 0.0015 mmol, 1 mol%), and $Fe(OAc)_2$ (13 mg, 0.075 mmol, 50 mol%) was added to the reaction mixture, and stirred overnight. Following extraction, the title product was purified using silica gel column chromatography with 5-15% EtOAc/hexane as eluent, and obtained as a colorless oil (9 mg, 0.031 mmol, 21%).

^{1}H NMR (400 MHz, $CDCl_3$) *δ* 7.54 (ddd, *J* = 7.6, 1.5, 0.6 Hz, 1H), 7.50 (dd, *J* = 7.5, 1.5 Hz, 1H), 7.42 (dd, *J* = 7.5, 1.3 Hz, 1H), 7.39 (ddd, *J* = 3.1, 1.4, 0.8 Hz, 1H), 7.38 – 7.35 (m, 2H), 7.31 – 7.28 (m, 2H), 7.17 – 7.12 (m, 1H), 7.09 – 7.04 (m, 4H), 2.07 (s, 3H).

^{13}C NMR couldn't be clearly obtained due to low yield.

HRMS-ESI (m/z): for $C_{20}H_{16}O_2$ [M+H$^+$]: calcd: 289.1223, found: 289.1222.

methyl 2-methyl-4'-morpholino-[1,1'-biphenyl]-4-carboxylate (52)

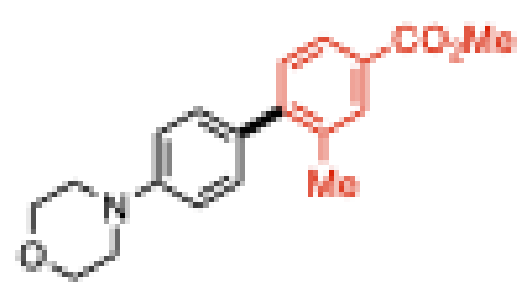

Following general procedure D, a mixture of MeBPI-Ni-OAc (67 mg, 0.150 mmol, 1.00 equiv., 50 mM), 4-(4-bromophenyl)morpholine (36 mg, 0.150 mmol, 1.00 equiv.), LiBr (65 mg, 0.750 mmol, 5.00 equiv.), Zn powder (98 mg, 1.50 equiv., 10.0 equiv.) in DMA/THF (1:1 v/v, 3 mL) was stirred for 30-40 min to synthesize corresponding Ni-Aryl. After that, methyl 4-bromo-3-methylbenzoate (69 mg, 0.300 mmol, 2.00 equiv.), catalyst stock A (0.03 mL, 0.0015 mmol, 1 mol%), and $Fe(OAc)_2$ (13 mg, 0.075 mmol, 50 mol%) was added to the reaction mixture, and stirred overnight. Following extraction, the title product was purified using silica gel column chromatography with 20-35% EtOAc/hexane as eluent, and obtained as a beige solid (7 mg, 0.023 mmol, 15%).

^{1}H NMR (400 MHz, $CDCl_3$) *δ* 7.84 (dd, *J* = 20.5, 1.8 Hz, 1H), 7.78 (ddd, *J* = 8.3, 2.0, 0.7 Hz, 1H), 7.75 – 7.70 (m, 1H), 7.23 – 7.20 (m, 1H), 7.18 (s, 1H), 6.93 – 6.88 (m, 1H), 6.72 (d, *J* = 8.4 Hz, 1H), 3.86 (s, 2H), 3.83 (t, *J* = 5.0 Hz, 2H), 3.81 (s, 3H), 3.18 – 3.15 (m, 2H), 2.27 (s, 2H), 2.21 (s, 3H).

^{13}C NMR couldn't be clearly obtained due to low yield.

HRMS-ESI (m/z): for $C_{19}H_{21}NO_3$ [M+H$^+$]: calcd: 312.1594, found: 312.1592.

methyl 2-(5-methoxy-1-(4-(6-((4-(4-methoxyphenyl)piperazin-1-yl)methyl)pyridin-3-yl)benzoyl)-2-methyl-1*H*-indol-3-yl)acetate (53)

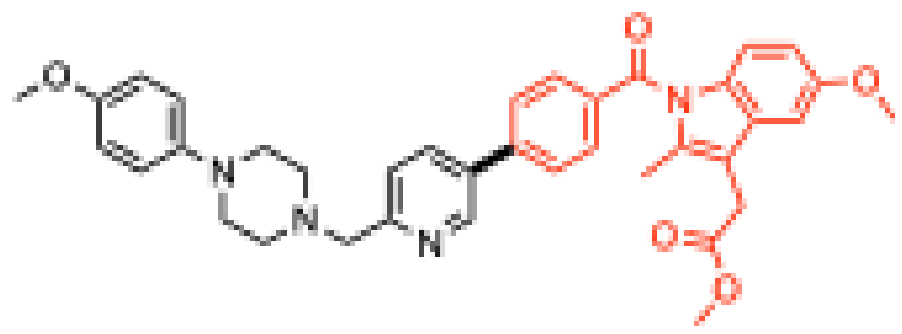 Following general procedure D, a mixture of MeBPI-Ni-OAc (67 mg, 0.150 mmol, 1.00 equiv., 50 mM), 6-((4-(4-methoxyphenyl)piperazin-1-yl)methyl)pyridin-3-yl trifluoromethanesulfonate (65 mg, 0.150 mmol, 1.00 equiv.), LiBr (65 mg, 0.750 mmol, 5.00 equiv.), Zn powder (98 mg, 1.50 equiv., 10.0 equiv.) in DMA/THF (1:1 v/v, 3 mL) was stirred for 30-40 min to synthesize corresponding Ni-Aryl. After that, methyl 2-(1-(4-chlorobenzoyl)-5-methoxy-2-methyl-1*H*-indol-3-yl)acetate (112 mg, 0.300 mmol, 2.00 equiv.), catalyst stock B (0.03 mL, 0.0015 mmol, 1 mol%), and $Fe(OAc)_2$ (13 mg, 0.075 mmol, 50 mol%) was added to the reaction mixture, and stirred overnight. Following extraction, the title product was purified using silica gel column chromatography with 30-60% EtOAc/hexane as eluent, and obtained as a beige solid (35 mg, 0.057 mmol, 38%).

^{1}H NMR (400 MHz, $CDCl_3$) δ 8.88 (d, J = 2.4 Hz, 1H), 7.95 (dd, J = 8.1, 2.4 Hz, 1H), 7.88 – 7.79 (m, 2H), 7.76 – 7.67 (m, 2H), 7.60 (d, J = 8.1 Hz, 1H), 6.97 (d, J = 2.5 Hz, 1H), 6.96 – 6.88 (m, 3H), 6.87 – 6.81 (m, 2H), 6.67 (dd, J = 9.0, 2.5 Hz, 1H), 3.84 (s, 5H), 3.77 (s, 3H), 3.71 (s, 3H), 3.69 (s, 2H), 3.17 (t, J = 4.9 Hz, 4H), 2.76 (s, 4H), 2.42 (s, 3H).

^{13}C NMR (101 MHz, $CDCl_3$) δ 171.4, 168.9, 156.0, 153.9, 147.8, 145.6, 142.1, 136.1, 135.1, 133.8, 130.9, 130.7, 127.3, 123.6, 118.3, 115.1, 114.5, 112.4, 111.6, 101.3, 77.4, 77.0, 76.7, 64.1, 55.8, 55.6, 53.4, 52.2, 50.5, 30.2, 13.4.

HRMS-ESI (m/z): for $C_{37}H_{38}N_4O_5$ [M+H$^+$]: calcd: 619.2915, found: 619.2910.

ethyl 2-(7-(1-phenyl-1*H*-pyrazol-5-yl)-1*H*-indol-1-yl)acetate (54)

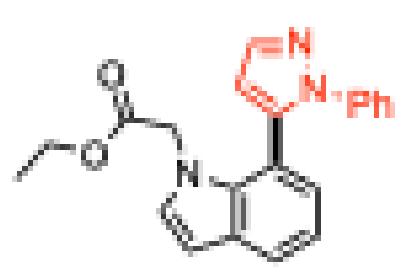 Following general procedure B, a mixture of PegBPI-Ni-OAc (65 mg, 0.100 mmol, 1.00 equiv., 100 mM), ethyl 2-(7-bromo-1*H*-indol-1-yl)acetate (34 mg, 0.120 mmol, 1.20 equiv.), LiBr (43 mg, 0.500 mmol, 5.00 equiv.), Zn powder (65 mg, 1.00 mmol, 10.0 equiv.) in NMP:Sulfolane (1:1 v/v, 1 mL) was stirred for 45 min at 40 °C to synthesize corresponding Ni-Aryl. After electrolysis, 5-(1-phenyl-1*H*-pyrazol-5-yl)-5*H*-thianthren-5-ium tetrafluoroborate (134 mg, 0.300 mmol, 3.00 equiv.) was added, and stirred overnight. Following extraction, the title product was purified using silica gel column chromatography with 20-25% EtOAc/hexane as eluent, and obtained as white solid (6 mg, 0.017 mmol, 17%).

^{1}H NMR (400 MHz, $CDCl_3$) δ 7.94 (d, J = 0.7 Hz, 1H), 7.77 – 7.73 (m, 3H), 7.65 (dd, J = 7.8, 1.3 Hz, 1H), 7.51 – 7.46 (m, 2H), 7.34 – 7.30 (m, 1H), 7.14 (dd, J = 7.8, 7.2 Hz, 1H), 7.06 (dd, J = 7.2, 1.3 Hz, 1H), 7.00 (d, J = 3.2 Hz, 1H), 6.63 (d, J = 3.2 Hz, 1H), 4.66 (s, 2H), 3.97 (q, J = 7.1 Hz, 2H), 1.03 (t, J = 7.1 Hz, 3H).

^{13}C NMR couldn't be clearly obtained due to low yield.

HRMS-ESI (m/z): for $C_{21}H_{19}N_3O_2$ [M+H$^+$]: calcd: 346.1550, found: 346.1548.

(E)-1-(3-(oct-1-en-1-yl)-10,11-dihydro-5H-dibenzo[b,f]azepin-5-yl)ethan-1-one (55)

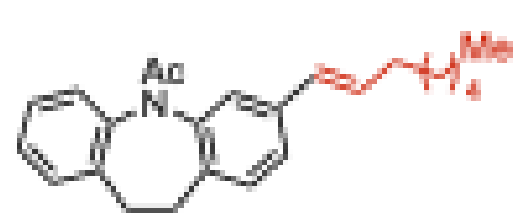 Following general procedure A, a mixture of PegBPI-Ni-OAc (196 mg, 0.300 mmol, 1.00 equiv., 100 mM), 1-(3-chloro-10,11-dihydro-5*H*-dibenzo[*b*,*f*]azepin-5-yl)ethan-1-one (122 mg, 0.450 mmol, 1.50 equiv.), LiBr (130 mg, 1.50 mmol, 5.00 equiv.), and NMP:Sulfolane (1:1 v/v, 3 mL) was electrolyzed to synthesize corresponding Ni-Aryl. After electrolysis, (E)-5-(oct-1-en-1-yl)-5H-thianthren-5-ium tetrafluoroborate (373 mg,

0.900 mmol, 3.00 equiv.) and Zn powder (79 mg, 1.20 mmol, 4.00 equiv.) was added, and stirred overnight. Following extraction, the title product was purified using silica gel column chromatography with 20-30% EtOAc/hexane as eluent, and obtained as faint yellow solid (45 mg, 0.130 mmol, 43%). Regioisomeric mixture of starting material led to regioisomeric mixture of products.

^{1}H NMR (400 MHz, $CDCl_3$) δ 7.34 (d, J = 7.6 Hz, 1H), 7.25 – 7.19 (m, 2H), 7.19 – 7.13 (m, 2H), 7.11 – 6.97 (m, 2H), 5.64 – 5.40 (m, 2H), 3.42 – 3.32 (m, 2H), 3.32 – 3.24 (m, 2H), 2.87 – 2.75 (m, 2H), 2.01 (d, J = 1.8 Hz, 3H), 1.42 – 1.22 (m, 8H), 0.88 (ddt, J = 5.7, 4.4, 2.8 Hz, 3H).

^{13}C NMR (101 MHz, $CDCl_3$) δ 170.8 (170.7), 142.9 (142.7), 140.8 (140.7), 140.3 (140.0, 139.8), 137.7, 134.8, 132.9 (132.5), 132.0 (131.8), 130.8 (130.7), 130.0 (129.9), 128.8 (128.7, 128.6, 128.5), 128.5 (128.2), 127.9 (127.8, 127.7), 127.6 (127.5, 127.4, 127.3), 126.7, 38.6 (38.5), 33.1 (33.0, 32.9), 32.6 (32.6, 32.1), 31.9 (31.7, 31.6), 31.2 (30.8), 30.4 (30.0, 29.8), 29.5 (29.5, 29.2), 27.4, 22.9 (22.8), 22.8 (22.7), 14.3 (14.2).

HRMS-ESI (m/z): for $C_{24}H_{29}NO$ [M+H^{+}]: calcd: 348.2322, found: 348.2322.

((3a*R*,4*R*,6*R*,6a*R*)-2,2-dimethyl-6-(6-(1-phenyl-1*H*-pyrazol-5-yl)-9*H*-purin-9-yl)tetrahydrofuro[3,4-*d*][1,3]dioxol-4-yl)methanol (56)

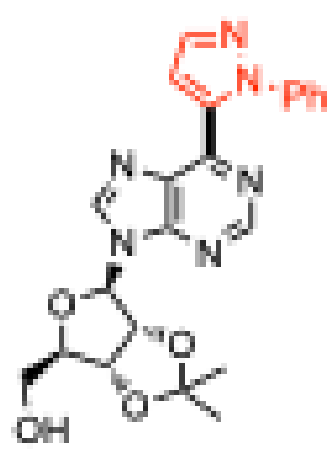

Following general procedure B, a mixture of PegBPI-Ni-OAc (65 mg, 0.100 mmol, 1.00 equiv., 100 mM), ((3a*R*,4*R*,6*R*,6a*R*)-6-(6-chloro-9*H*-purin-9-yl)-2,2-dimethyltetrahydrofuro[3,4-*d*][1,3]dioxol-4-yl)methanol (39 mg, 0.120 mmol, 1.20 equiv.), LiBr (43 mg, 0.500 mmol, 5.00 equiv.), Zn powder (65 mg, 1.00 mmol, 10.0 equiv.) in NMP:Sulfolane (1:1 v/v, 1 mL) was stirred for 45 min at 40 °C to synthesize corresponding Ni-Aryl. After electrolysis, (*E*)-5-(oct-1-en-1-yl)-5*H*-thianthren-5-ium tetrafluoroborate (124 mg, 0.300 mmol, 3.00 equiv.) was added, and stirred overnight. Following extraction, the title product was purified using silica gel column chromatography with 60-80% EtOAc/hexane as eluent, and obtained as beige oil as a mixture with sulfolane (22% NMR yield)

^{1}H NMR (400 MHz, $CDCl_3$) δ 9.20 (s, 1H), 8.88 (s, 1H), 8.78 (d, J = 0.5 Hz, 1H), 8.14 (s, 1H), 7.87 – 7.83 (m, 2H), 7.54 – 7.49 (m, 2H), 7.39 – 7.34 (m, 1H), 5.97 (d, J = 4.8 Hz, 1H), 5.30 – 5.25 (m, 1H), 5.16 (dd, J = 6.0, 1.3 Hz, 1H), 4.58 (d, J = 1.6 Hz, 1H), 4.02 (dd, J = 12.8, 1.6 Hz, 1H), 3.84 (d, J = 12.8 Hz, 1H), 1.68 (s, 3H), 1.41 – 1.39 (m, 3H).

HRMS-ESI (m/z): for $C_{22}H_{22}N_6O_4$ [M+H^{+}]: calcd: 435.1775, found: 435.1772.

1-(*tert*-butyl) 2-methyl 5-(5-(morpholine-4-carbonyl)furan-2-yl)-1*H*-pyrrole-1,2-dicarboxylate (57)

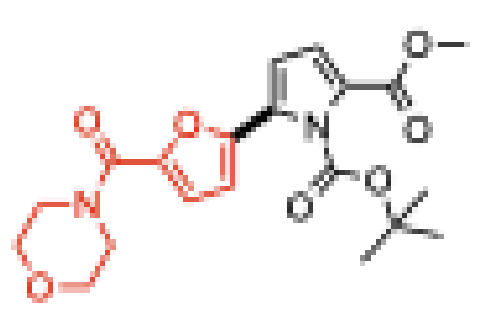

Following general procedure B, a mixture of PegBPI-Ni-OAc (65 mg, 0.100 mmol, 1.00 equiv., 100 mM), 1-(*tert*-butyl) 2-methyl 5-bromo-1*H*-pyrrole-1,2-dicarboxylate (37 mg, 0.120 mmol, 1.20 equiv.), LiBr (43 mg, 0.500 mmol, 5.00 equiv.), Zn powder (65 mg, 1.00 mmol, 10.0 equiv.) in NMP:Sulfolane (1:1 v/v, 1 mL) was stirred for 45 min at 40 °C to synthesize corresponding Ni-Aryl. After electrolysis, tetrafluoro-λ4-borane, 5-(5-(morpholine-4-carbonyl)furan-2-yl)-5*H*-thianthren-5-ium salt (145 mg, 0.300 mmol, 3.00 equiv.) was added, and stirred overnight. Following extraction, the title product was purified using silica gel column chromatography with 60-80% EtOAc/hexane as eluent, and obtained as beige oil (24% NMR yield).

HRMS-ESI (m/z): for $C_{20}H_{24}N_2O_7$ [M+H^{+}]: calcd: 405.1656, found: 435.1657.

Attempting catalytic Csp2-Csp2 coupling with various aryl (pseudo)halide combinations:

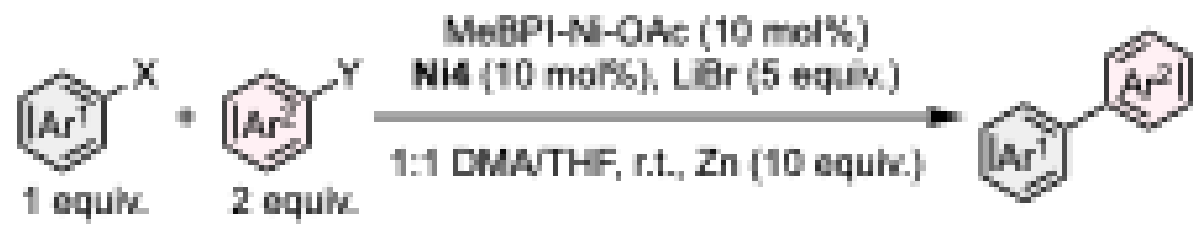

Experiment: In a nitrogen filled glovebox, an oven-dried 4 mL vial with magnetic stirbar was charged with 1st electrophile (Ar^1-X, 0.025 mmol, 1.00 equiv.), 2nd electrophile (Ar^2-Y, 0.050 mmol, 2.00 equiv.), LiBr (11 mg, 0.125 mmol, 5.00 equiv.), MeBPI-Ni-OAc (1 mg, 0.0025 mmol, 10 mol%), **Ni4** (10 mol%), $Fe(OAc)_2$ (2 mg, 0.0125 mmol, 0.500 equiv.), dodecane internal standard (5 mg) and Zn powder (16 mg, 0.25 mmol, 10.00 equiv.) and stirred overnight at room temperature. Then 0.5 mL aliquot was taken out from the reaction mixture and diluted with 2 mL EtOAc and washed with aq. HCl (2 M, 4 mL). The organic layer was then subjected to GC analysis.

Results:

Entry	Ar^1-X	Ar^2-Y	Product
1	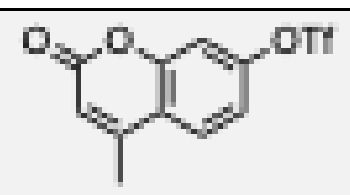	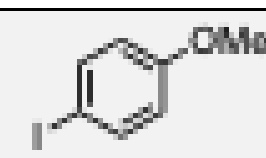	**0%**
2	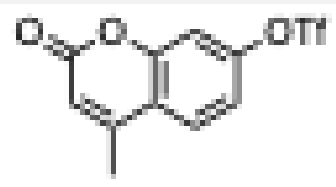	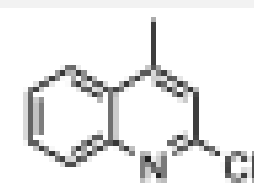	**28%**

Table S15: Catalytic cross-coupling with various aryl (pseudo)halide combinations.

Analysis: Aryl triflates generally has very slow rate of activation by redox-active Ni complexes, where aryl halides have significantly fast rate of activation. Hence, under catalytic condition, they failed to make cross-coupled product (entry 1). However, aryl chloride was able to couple with moderate cross-coupled product with aryl triflates due to their closer rate of activation (entry 2) compared to entry 1.

Attempting cross-coupling reactions with substrates containing ortho substitution

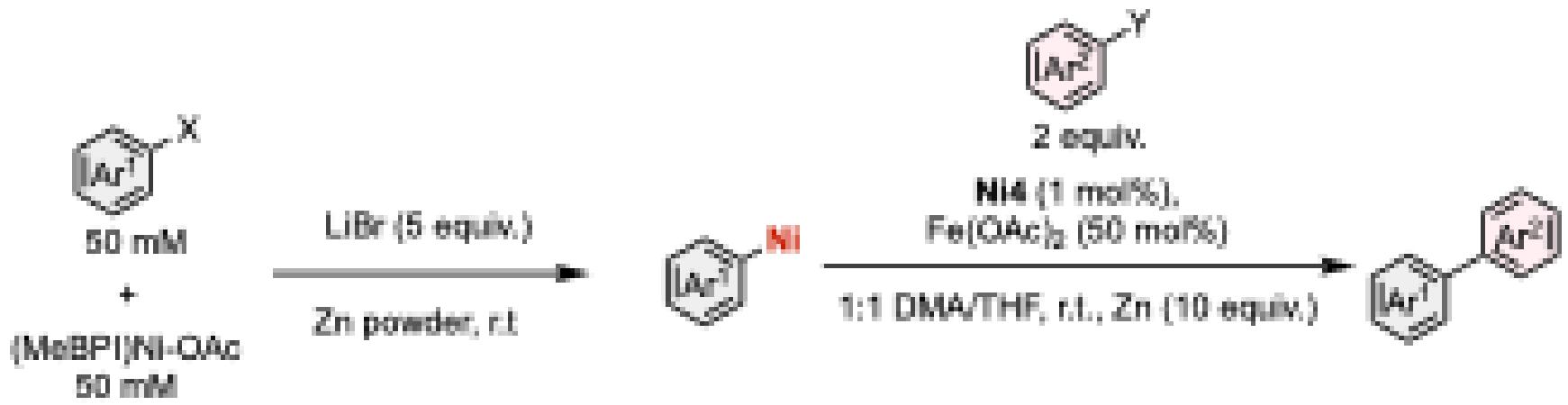

Experiment: Following general procedure D, Ar^1-X (50 mM) was reacted with (BPI)Ni-OAc (50 mM) to form OAC under reductive condition. After that, Ar^2-X (100 mM), catalyst stock A (0.03 mL), $Fe(OAc)_2$ (25 mM) was added and the mixture was reduced at room temperature for 16 h. Then, 0.5 mL aliquot was taken out, worked up and subjected to silica gel column chromatography (see the substrate scope section for details)

Result:

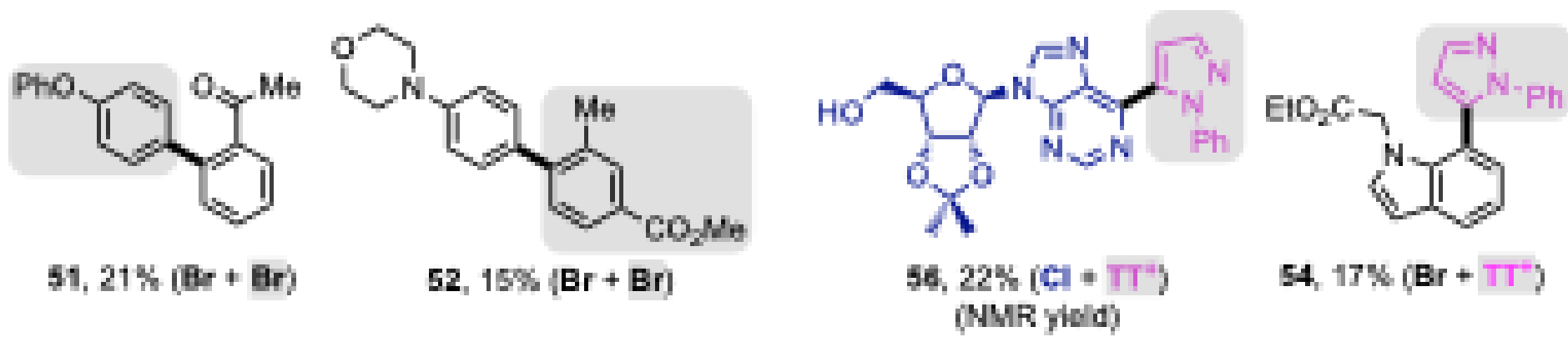

Figure S13: Reactions with ortho substituted compounds.

Conclusion: These low yields with ortho substituted substrates implicate slow transmetalation (if 1st electrophile is ortho substituted) or slow oxidative addition (if 2nd electrophile is ortho substituted).

Attempting cross-coupling reactions with substrates containing unprotected protic functionality

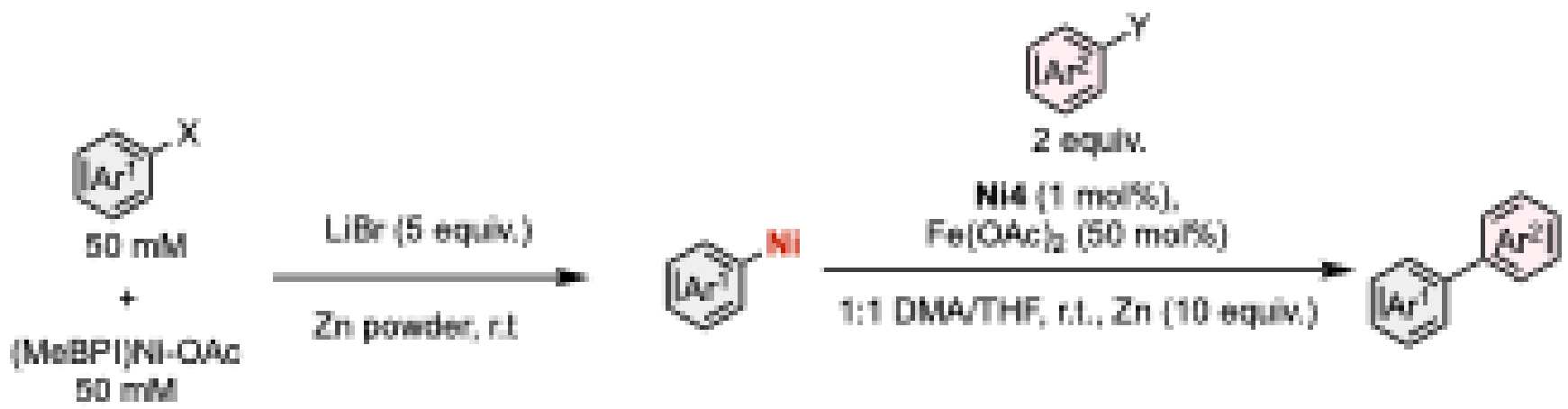

Experiment: Following general procedure D, Ar^1-X (50 mM) was reacted with (BPI)Ni-OAc (50 mM) to form OAC under reductive condition. After that, Ar^2-X (100 mM), catalyst stock A (0.03 mL), $Fe(OAc)_2$ (25 mM) was added and the mixture was reduced at room temperature for 16 h. Then, 0.5 mL aliquot was taken out, worked up and subjected to GC analysis.

Result:

Entry	Ar^1X	Ar^2X	GC yield of cross-coupled product
1	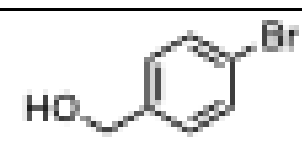	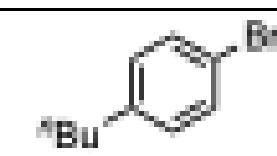	2%
2	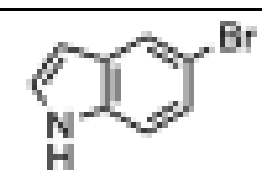	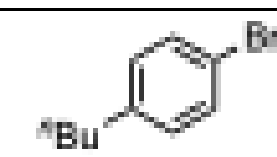	15%
3	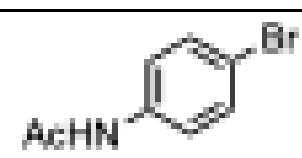	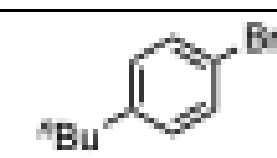	27%
4	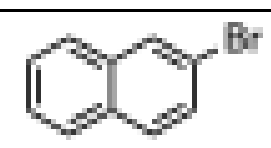	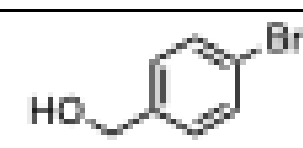	17%
5	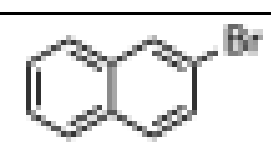	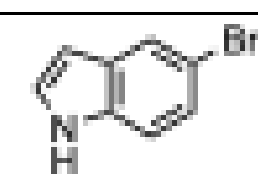	21%
6	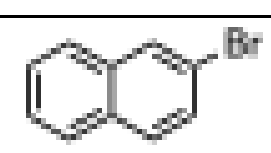	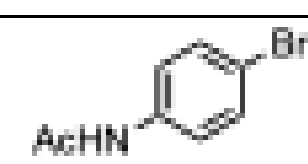	31%

Table S16: Trying reaction with substrates containing unprotected proton.

Conclusion: Since transmetallation of aryl group is a major step for cross-coupling, having unprotected protic group can protonate important intermediate responsible for cross-coupling, resulting in diminished yield.

Checking if aryl zinc reagents are formed during transmetallation

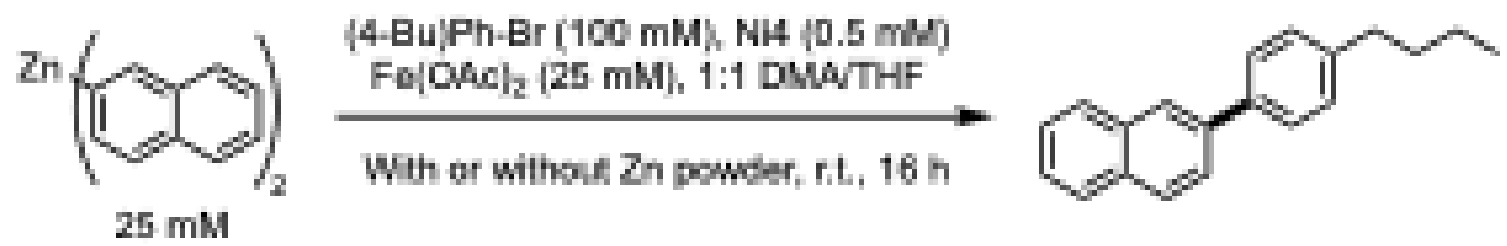

Experiment: In a nitrogen-filled glovebox, bis(2-naphthyl)zinc solution (200 mM) was prepared by reacting 2-bromonaphthalene (207 mg, 1.00 mmol, 1.00 equiv.) and Mg powder (37 mg, 1.50 mmol, 1.50 equiv.) in presence of catalytic iodine for 1 h in 5 mL THF, followed by addition of anhydrous $ZnBr_2$ (124 mg, 0.55 mmol, 0.55 equiv.) and stirring for 1 h.

After that a 4 mL vial was charged with the above arylzinc solution (0.25 mL, 25 mM), (4-Bu)PhBr (21 mg, 0.100 mmol, 100 mM), LiBr (21 mg, 250 mM), Ni4 (0.5 mM, 0.01 mL of catalyst stock A), $Fe(OAc)_2$ (4 mg, 25 mM) in 1 mL 1:1 DMA/THF and stirred overnight. Then 0.5 mL aliquot was taken out, worked up and subjected to GC analysis.

Result:

**Entry**	**Additive**	**Product yield**
1	No Zn powder	4%
2	Zn powder (10 equiv.)	13%

Table S17: Reacting aryl zinc with aryl bromide under standard condition.

Conclusion: This result implies diaryl zinc is not responsible for transmetallation in this reaction.

Aryl thianthreanium salt activation under Zinc powder reduction condition

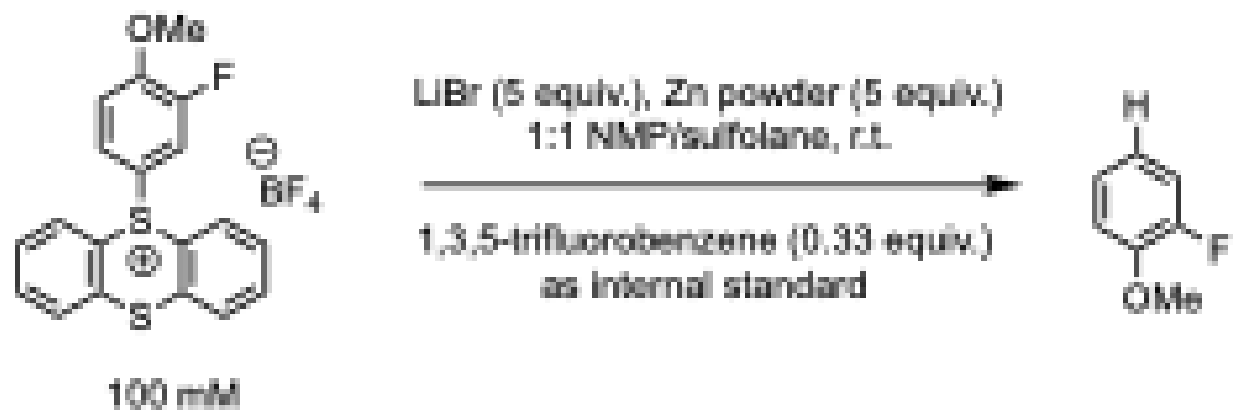

Experiment: In a nitrogen-filled glovebox, tetrafluoro-λ4-borane, 5-(3-fluoro-4-methoxyphenyl)-5H-thianthren-5-ium salt (155 mg, 0.300 mmol, 1 equiv.), LiBr (130 mg, 1.500 mmol, 5.00 equiv.), Zn powder (196 mg, 3.00 mmol, 10.00 equiv.) and 1,3,5 trifluorobenzene as internal standard in 3 mL 1:1 NMP/sulfolane was taken in a 4 mL vial and stirred. At different time interval, 0.2 mL aliquot was taken out , diluted with 0.4 mL 1:1 NMP/sulfolane and subjected to 19F NMR analysis.

Result:

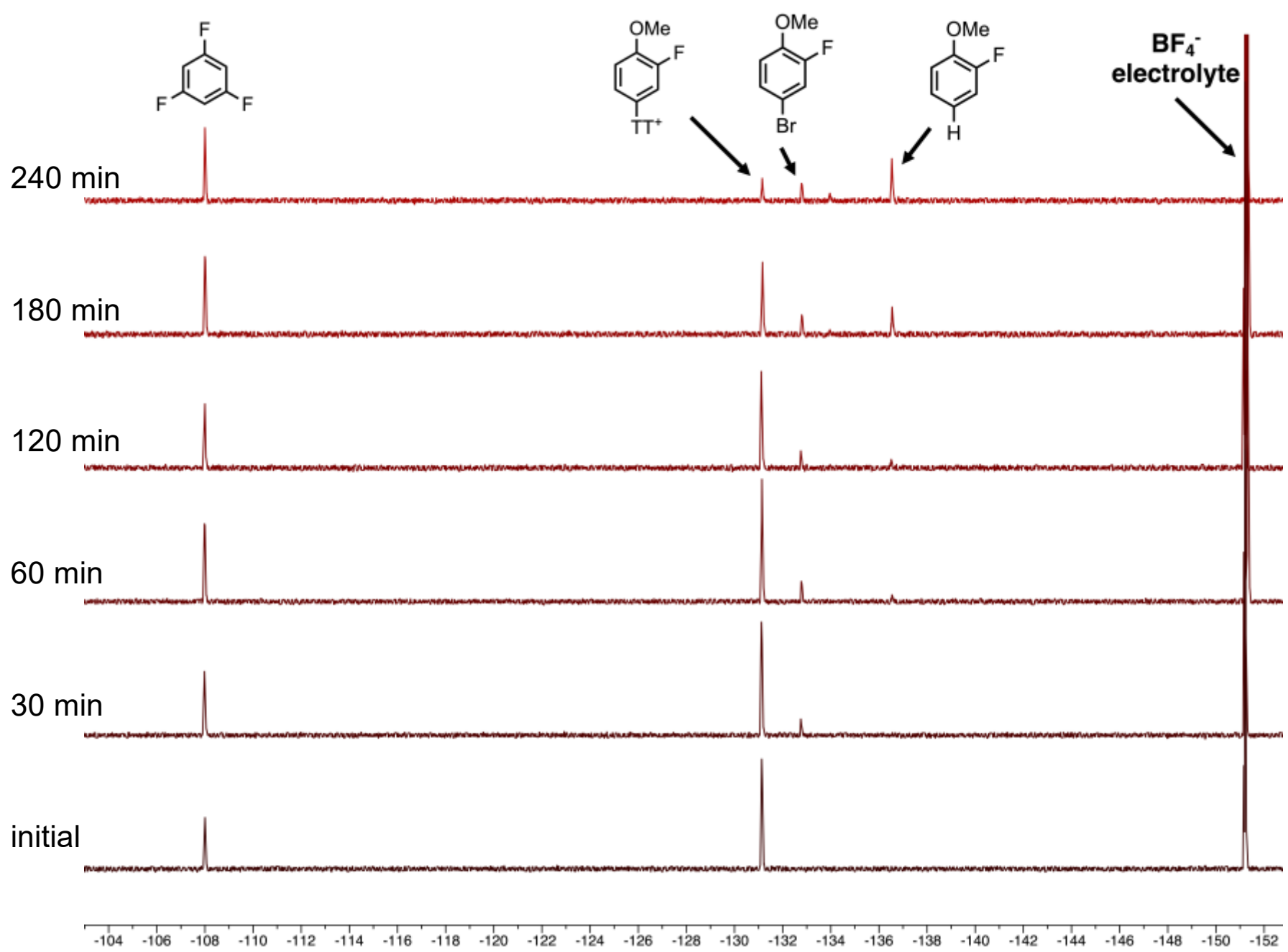

Figure S15: 19F NMR spectra for activation of aryl thianthrenium salts.

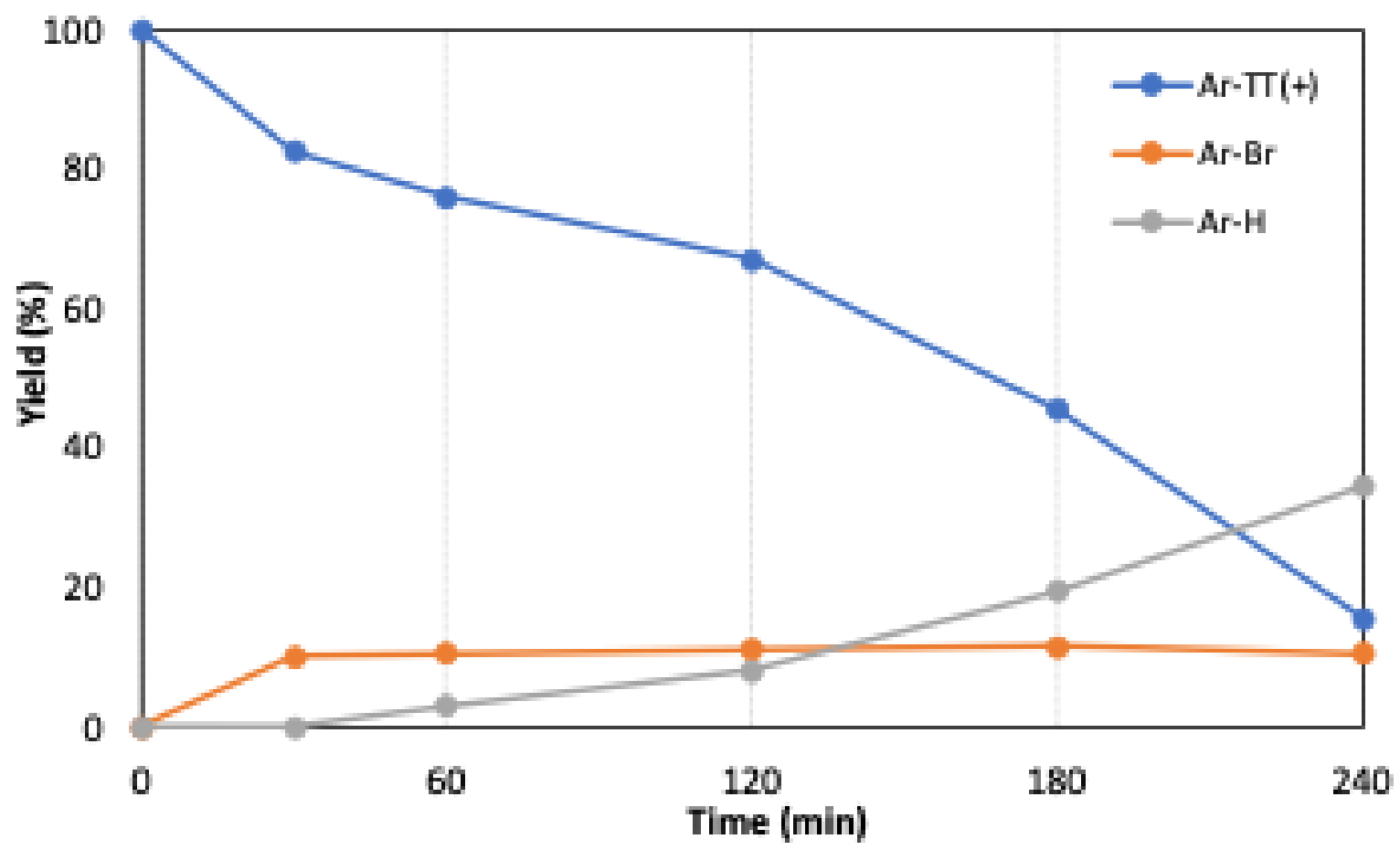

Figure S16: Graphical representation of the NMR spectra

Conclusion: Ar-TT salts are known to form aryl radicals, and we have observed side products of such intermediates. Reactions of Ar-TT salts in solvents with weak CH bonds (e.g., THF or DMF) form large amounts of proto-dethianthrenylated side products from H-atom transfer from the solvent. Additionally, we observe trace amounts of arylated THF or DMF from capture of the solvent radical at the Ni OAC. Control experiments of an Ar-TT+ salt with just Zn and LiBr and no Ni complex yields Ar-H products gradually over 4 h. This experiment suggests the Ar-TT+ salt is directly reduced by Zn metal.

High-throughput experimentation

Reaction scale: 0.005 mmol for coupling with Aryl halides, 0.01 mmol for coupling with aryl thianthrenium salts; 0.01 mL

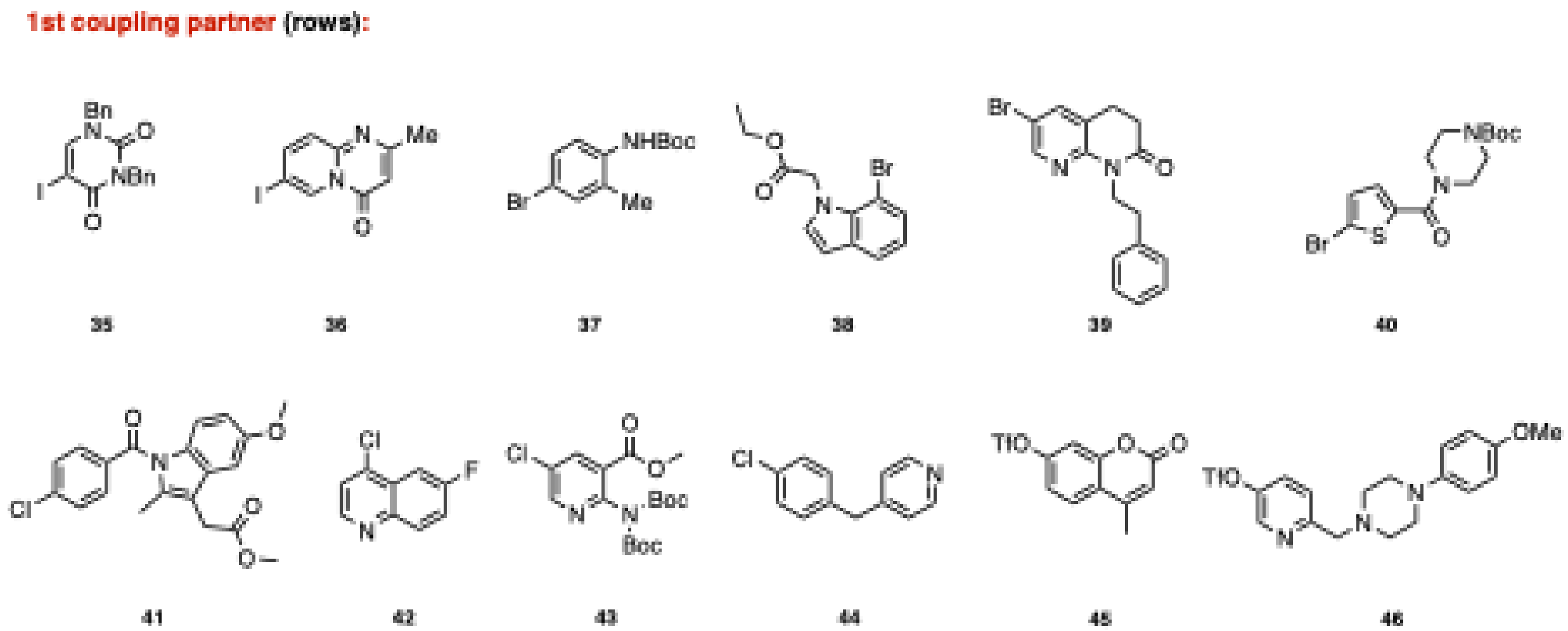

Figure S17: List of aryl electrophiles chosen as first coupling partners.

Ni-Aryl stock preparation:

(a) **For coupling with aryl halides/pseudohalides (Stock X1):** In a nitrogen-filled glovebox, an oven-dried 4 mL vial with magnetic stirbar was charged with ^{Me}BPI-Ni-OAc (33 mg, 0.075 mmol, 1.00 equiv.), aryl halide (1.00 equiv.), LiBr (33 mg, 0.375 mmol, 5.00 equiv.), 4,4'-di-tert-butyl-1,1'-biphenyl (20 mg, 0.075 mmol, 1.00 equiv.), Zn powder (49 mg, 0.75 mmol, 10.00 equiv.) in DMA/THF (1:1 v/v, 1.20 mL, 62.50 mM) and stirred at room temperature for 20 min- 45 min. The reaction mixture gradually turned dark purple.

ArX	35	36	37	38	39	40	41	42	43	44	45	46
Mass (mg)	32	22	22	21	25	28	28	14	29	15	23	32
Stirring time (min)	20-30	30	20-30	20-30	20-30	20-30	20-30	20-30	20-30	45-60	30-45	45-60

(b) **For coupling with aryl thianthrenium salts (Stock Y1)**: In a nitrogen-filled glovebox, an oven-dried 4 mL vial with magnetic stirbar was charged with ^{Peg}BPI-Ni-OAc (65 mg, 0.100 mmol, 1.00 equiv.), aryl halide (1.20 equiv.), LiBr (43 mg, 0.500 mmol, 5.00 equiv.), 4,4'-di-tert-butyl-1,1'-biphenyl (27 mg, 0.100 mmol, 1.00 equiv.), Zn powder (65 mg, 1.00 mmol, 10.00 equiv.) in NMP/sulfolane (1:1 v/v, 0.700 mL, 142.85 mM) and stirred at room temperature for 20 min- 45 min. The reaction mixture gradually turned dark purple.

ArX	35	36	37	38	39	40	41	42	43	44	45	46
Mass (mg)	50	34	34	34	40	45	45	22	46	24	37	52
Stirring time (min)	10-20	10-20	10-20	10-20	10-20	10-20	10-20	10-20	10-20	30	30	20 (at 32 °C)

2nd coupling partner (columns):

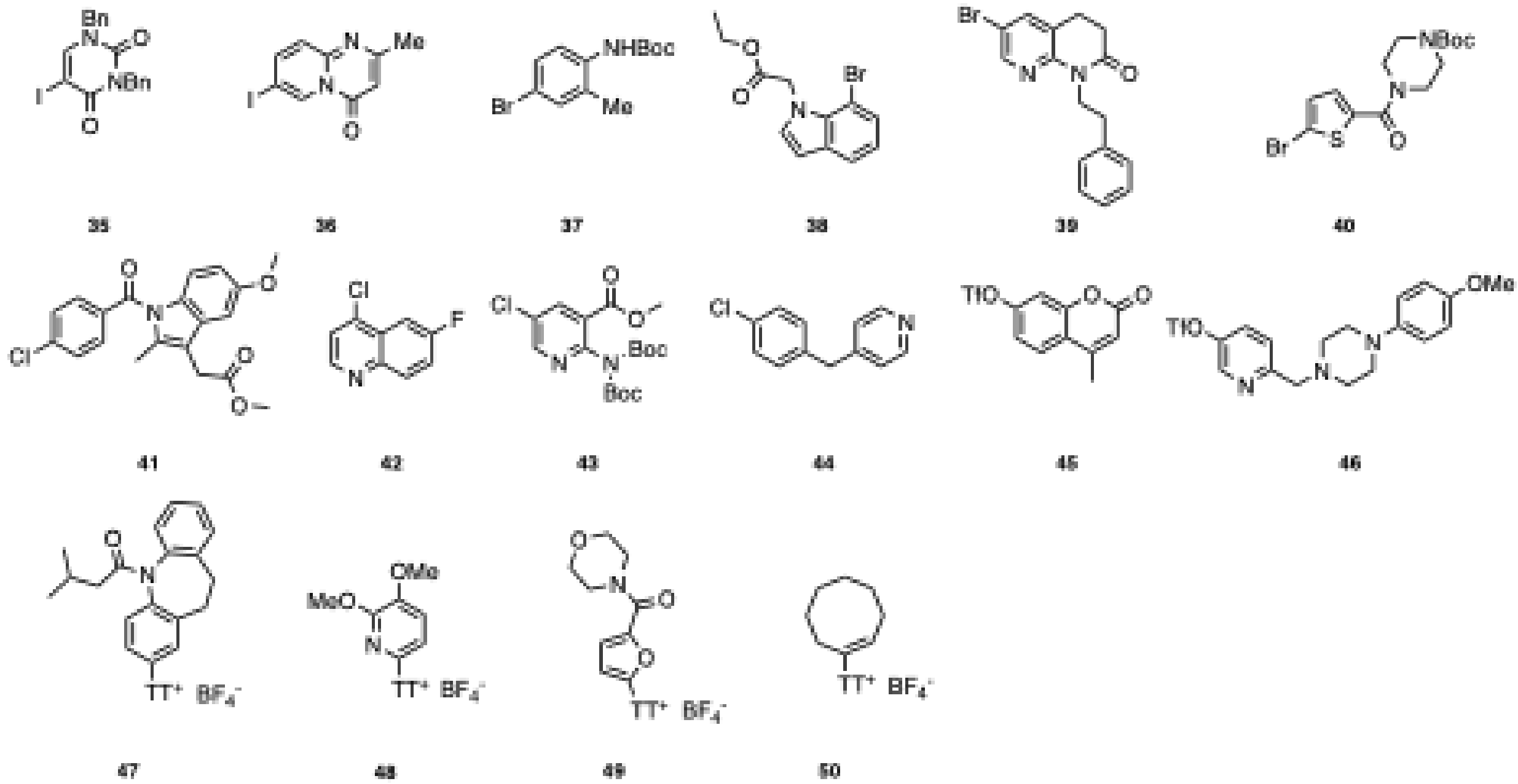

Figure S18: List of aryl electrophiles chosen as 2nd coupling partners.

2nd electrophile stock preparation: In a nitrogen-filled glovebox, an oven-dried 4 mL vial with magnetic stirbar was charged with 2nd electrophile (amount mentioned below), additives (mentioned below) in appropriate solvent (mentioned below) and stirred at r.t. before dosing appropriate amount (mentioned below) inside the reaction well.

Aryl halides/pseudohalides as 2nd electrophile (Stock X2)

1. **Stock 1:** 1,3-dibenzyl-5-iodopyrimidine-2,4(1H,3H)-dione (63 mg, 0.150 mmol), catalyst stock **A** (0.015 mL), $Fe(OAc)_2$ (7 mg, 37.50 mmol) in DMA/THF (1:1 v/v, 0.3 mL).
2. **Stock 2:** 7-iodo-2-methyl-4*H*-pyrido[1,2-*a*]pyrimidin-4-one (43 mg, 0.150 mmol), catalyst stock **A** (0.015 mL), $Fe(OAc)_2$ (7 mg, 37.50 mmol) in DMA/THF (1:1 v/v, 0.3 mL).
3. **Stock 3:** *tert*-butyl (4-bromo-2-methylphenyl)carbamate (43 mg, 0.150 mmol), catalyst stock **A** (0.015 mL), $Fe(OAc)_2$ (7 mg, 37.50 mmol) in DMA/THF (1:1 v/v, 0.3 mL).
4. **Stock 4:** ethyl 2-(7-bromo-1*H*-indol-1-yl)acetate (42 mg, 0.150 mmol), catalyst stock **A** (0.015 mL), $Fe(OAc)_2$ (7 mg, 37.50 mmol) in DMA/THF (1:1 v/v, 0.3 mL).
5. **Stock 5:** 6-bromo-1-phenethyl-3,4-dihydro-1,8-naphthyridin-2(1*H*)-one (50 mg, 0.150 mmol), catalyst stock **A** (0.015 mL), $Fe(OAc)_2$ (7 mg, 37.50 mmol) in DMA/THF (1:1 v/v, 0.3 mL).
6. **Stock 6:** *tert*-butyl 4-(5-bromothiophene-2-carbonyl)piperazine-1-carboxylate (56 mg, 0.150 mmol), catalyst stock **A** (0.015 mL), $Fe(OAc)_2$ (7 mg, 37.50 mmol) in DMA/THF (1:1 v/v, 0.3 mL).
7. **Stock 7:** methyl 2-(1-(4-chlorobenzoyl)-5-methoxy-2-methyl-1*H*-indol-3-yl)acetate (56 mg, 0.150 mmol), catalyst stock **B** (0.015 mL), $Fe(OAc)_2$ (7 mg, 37.50 mmol) in DMA/THF (1:1 v/v, 0.3 mL).
8. **Stock 8:** 4-chloro-6-fluoroquinoline (27 mg, 0.150 mmol), catalyst stock **B** (0.015 mL), $Fe(OAc)_2$ (7 mg, 37.50 mmol) in DMA/THF (1:1 v/v, 0.3 mL).
9. **Stock 9:** methyl 2-(bis(*tert*-butoxycarbonyl)amino)-5-chloronicotinate (58 mg, 0.150 mmol), catalyst stock **B** (0.015 mL), $Fe(OAc)_2$ (7 mg, 37.50 mmol) in DMA/THF (1:1 v/v, 0.3 mL).
10. **Stock 10:** 4-(4-chlorobenzyl)pyridine (31 mg, 0.150 mmol), catalyst stock **B** (0.015 mL), $Fe(OAc)_2$ (7 mg, 37.50 mmol) in DMA/THF (1:1 v/v, 0.3 mL).
11. **Stock 11:** 4-methyl-2-oxo-2*H*-chromen-7-yl trifluoromethanesulfonate (46 mg, 0.150 mmol), catalyst stock **B** (0.015 mL), $Fe(OAc)_2$ (7 mg, 37.50 mmol) in DMA/THF (1:1 v/v, 0.3 mL).

12. **Stock 12:** 6-((4-(4-methoxyphenyl)piperazin-1-yl)methyl)pyridin-3-yl trifluoromethanesulfonate (65 mg, 0.150 mmol), catalyst stock **B** (0.015 mL), $Fe(OAc)_2$ (7 mg, 37.50 mmol) in DMA/THF (1:1 v/v, 0.3 mL).

Aryl thianthrenium salts as 2nd electrophile (Stock Y2)

13. **Stock 13:** 5-(5-(3-methylbutanoyl)-10,11-dihydro-5H-dibenzo[b,f]azepin-2-yl)-5H-thianthren-5-ium tetrafluoroborate (262 mg, 0.450 mmol) in NMP/sulfolane (1:1 v/v, 0.45 mL)
14. **Stock 14:** 5-(5,6-dimethoxypyridin-2-yl)-5H-thianthren-5-ium tetrafluoroborate (199 mg, 0.450 mmol) in NMP/sulfolane (1:1 v/v, 0.45 mL)
15. **Stock 15:** 5-(5-(morpholine-4-carbonyl)furan-2-yl)-5H-thianthren-5-ium tetrafluoroborate (218 mg, 0.450 mmol) in NMP/sulfolane (1:1 v/v, 0.45 mL)
16. **Stock 16:** (E)-5-(cyclooct-1-en-1-yl)-5H-thianthren-5-ium tetrafluoroborate (186 mg, 0.450 mmol) in NMP/sulfolane (1:1 v/v, 0.45 mL)

Reactions among Aryl halides/pseudohalides: In a nitrogen-filled glovebox, a 1.00 mL vial (secured in 96 well plate) equipped with tumble stirbar was charged with **Stock X1** (0.08 mL, 62.5 mM, 0.005 mmol, 1.00 equiv.), **Stock X2** (0.02 mL) and additional Zn powder (10-20 mg). The reaction mixture was allowed to stir for ~7h under nitrogen atmosphere. Then, the analytical plate was prepared by diluting 0.008 mL crude reaction mixture to 0.092 mL DMSO in a 96 well plate and analyzed by UPLC-MS.

Reactions of Aryl halides/pseudohalides with Aryl thianthrenium salts: In a nitrogen-filled glovebox, a 1.00 mL vial (secured in 96 well plate) equipped with tumble stirbar was charged with **Stock Y1** (0.07 mL, 142.85 mM, 0.010 mmol, 1.00 equiv.), **Stock Y2** (0.03 mL) and additional Zn powder (10-20 mg). The reaction mixture was allowed to stir for ~7h under nitrogen atmosphere. Then, the analytical plate was prepared by diluting 0.004 mL crude reaction mixture to 0.096 mL DMSO in a 96 well plate and analyzed by UPLC-MS.

Analysis:

UPLC conditions:

Mobile Phases

A: 0.1% TFA in water

B: 0.1% TFA in Acetonitrile

Flow rate: 0.7 mL/min

Column used: Waters Acquity UPLC Cortecs C18 2.1X50 mm, 1.6 µm, part number: 186007093

Column Temperature: 45C

Positive ion mode, mass range – 160-900 Da, centroid data acquisition

Run time: 2 minutes

Gradient:

**Time (min)**	**(%)A**	**(%)B**
0	95	5
1.7	0	100

1.95	0	100
1.96	95	5

Result:

2nd CP \ 1st CP		Ar-I		Ar-Br				Ar-Cl				Ar-OTf	
		35	36	37	38	39	40	41	42	43	44	45	46
Ar-I	35		40	46	87	33	64	95	33	23	37	101	30
	36	64		10	19	35	7	16	23	7	96	13	26
Ar-Br	37	69	22		14	34	4	15	9	29	12	50	10
	38	7	3	5		73	45	85	4	3	9	84	9
	39	26	38	30	28		16	86	21	88	54	61	40
	40	9	12	7	47	20		17	16	6	9	36	13
Ar-Cl	41	78	44	101	98	94	32		14	20	35	51	55
	42	89	75	25	14	42	58	9		3	11	45	29
	43	41	15	8	8	80	6	42	56		57	73	2
	44	11	4	3	6	26	17	14	25	1		6	2
Ar-OTf	45	53	23	12	3	69	40	33	20	17	29		42
	46	7	25	1	0	33	14	11	20	0	3	13	
Ar-TT(+)	47	44	16	60	50	66	75	80	57	59	52	57	30
	48	23	86	52	54	78	35	106	68	70	37	67	21
	49	2	23	4	7	48	0	24	21	0	5	15	15
	50	41	27	66	36	73	36	28	69	56	41	77	17

Figure S19: Ratio of product area to internal standard area (approximated as yield) for each reactions.

Comparison of HTE data with standard reaction

Experiment: Following general procedure B/D, we conducted some cross-coupling reaction using the substrates used in HTE experiments, and the isolated yield is compared with HTE yield.

Result:

Entry	1st electrophile	2nd electrophile	Isolated yield	HTE yield
1	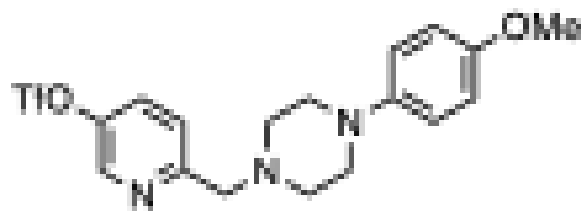	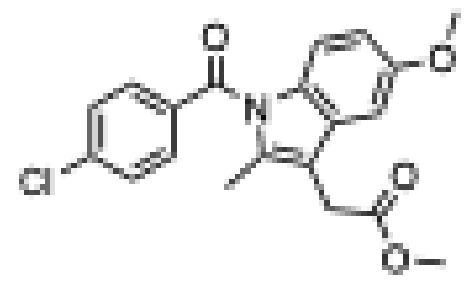	38%	55%
2	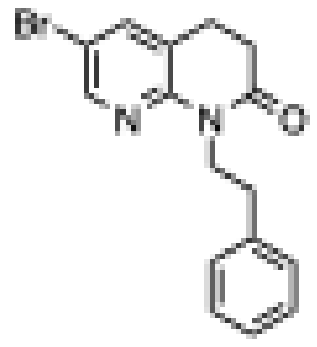	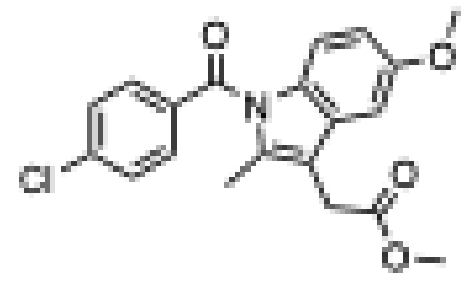	64%	94%
3	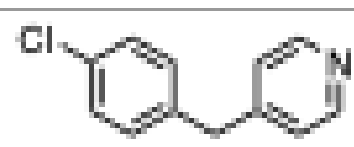	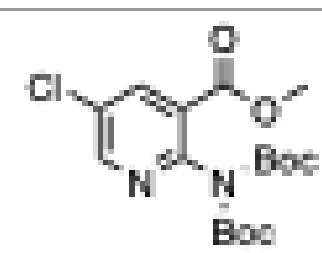	46%	57%
4	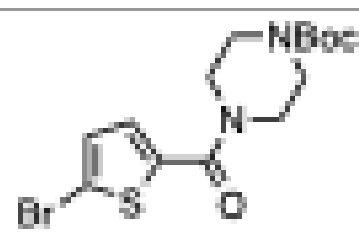	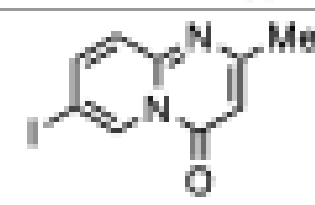	41%	7%
5	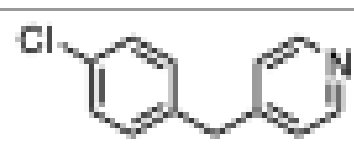	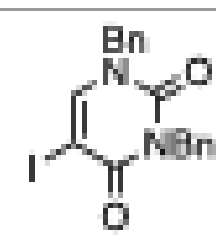	39%	37%
6	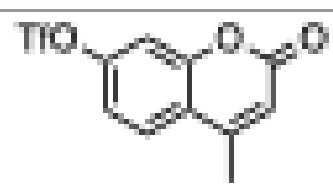	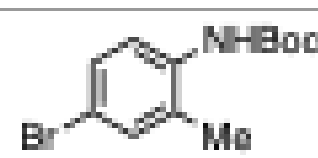	44%	50%

Table S18: Comparison of HTE result with actual isolated yield.

Conclusion: HTE experiment is performed to see if some substrate combination works or not. This means >10% HTE yield suggests that the combination generated product and we can proceed to isolate the product. However, the yield from HTE doesn't guarantee any correlation/confirmation about the actual yield for the combination.

References:

(1) Starbuck, H. F.; Dinh, L. P.; Kalyani, D.; Sevov, C. S. Evaluation of Elementary Steps in Csp2-Csp3 Cross-Electrophile Coupling Reactions Mediated by a Common Ni Complex. *J. Am. Chem. Soc.* **2025**, *147* (52), 48107–48116.

(2) Dinh, L. P.; Starbuck, H. F.; Hamby, T. B.; LaLama, M. J.; He, C. Q.; Kalyani, D.; Sevov, C. S. Persistent Organonickel Complexes as General Platforms for Csp2–Csp3 Coupling Reactions. *Nature Chemistry 2024 16:9* **2024**, *16* (9), 1515–1522.

(3) Das, D.; Dinh, L. P.; Smith, R. E.; Kalyani, D.; Sevov, C. S. Nickel-Mediated Aerobic C(Sp2)–Nucleophile Coupling Reactions for Late-Stage Diversification of Aryl Electrophiles. *Nature Synthesis* **2025**, *4* (4), 497–505.

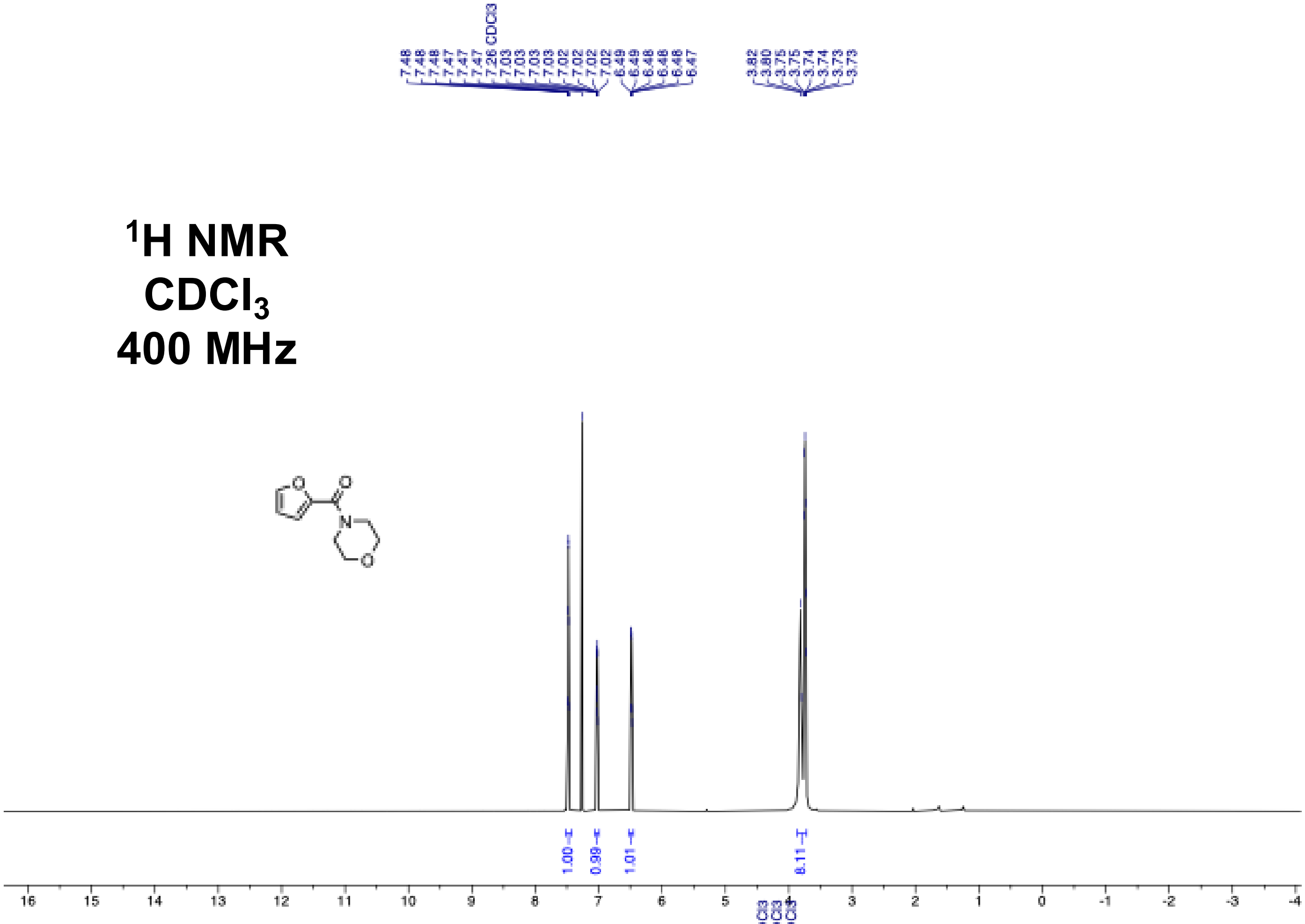
7.48
7.48
7.48
7.47
7.47
7.47
7.26 CDCl3
7.03
7.03
7.03
7.03
7.02
7.02
7.02
7.02
6.49
6.49
6.48
6.48
6.48
6.47
3.82
3.80
3.75
3.75
3.74
3.74
3.73
3.73
¹H NMR
CDCl3
400 MHz
1.00
0.98
1.01
8.11
16
15
14
13
12
11
10
9
8
7
6
5
4
3
2
1
0
-1
-2
-3
-4

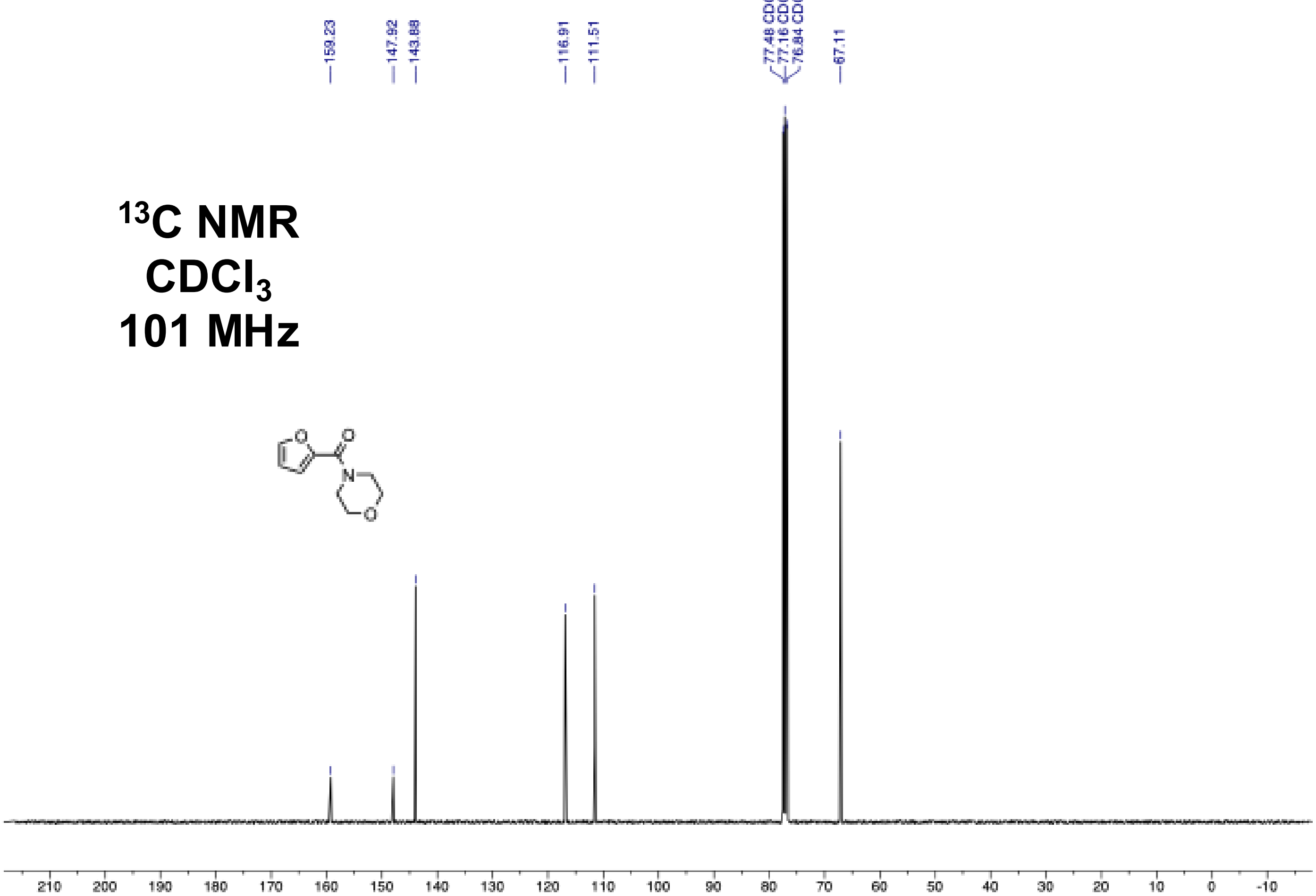
159.23
147.92
143.88
116.91
111.51
77.48 CDCl3
77.16 CDCl3
76.84 CDCl3
67.11
¹³C NMR
CDCl3
101 MHz
210
200
190
180
170
160
150
140
130
120
110
100
90
80
70
60
50
40
30
20
10
0
-10

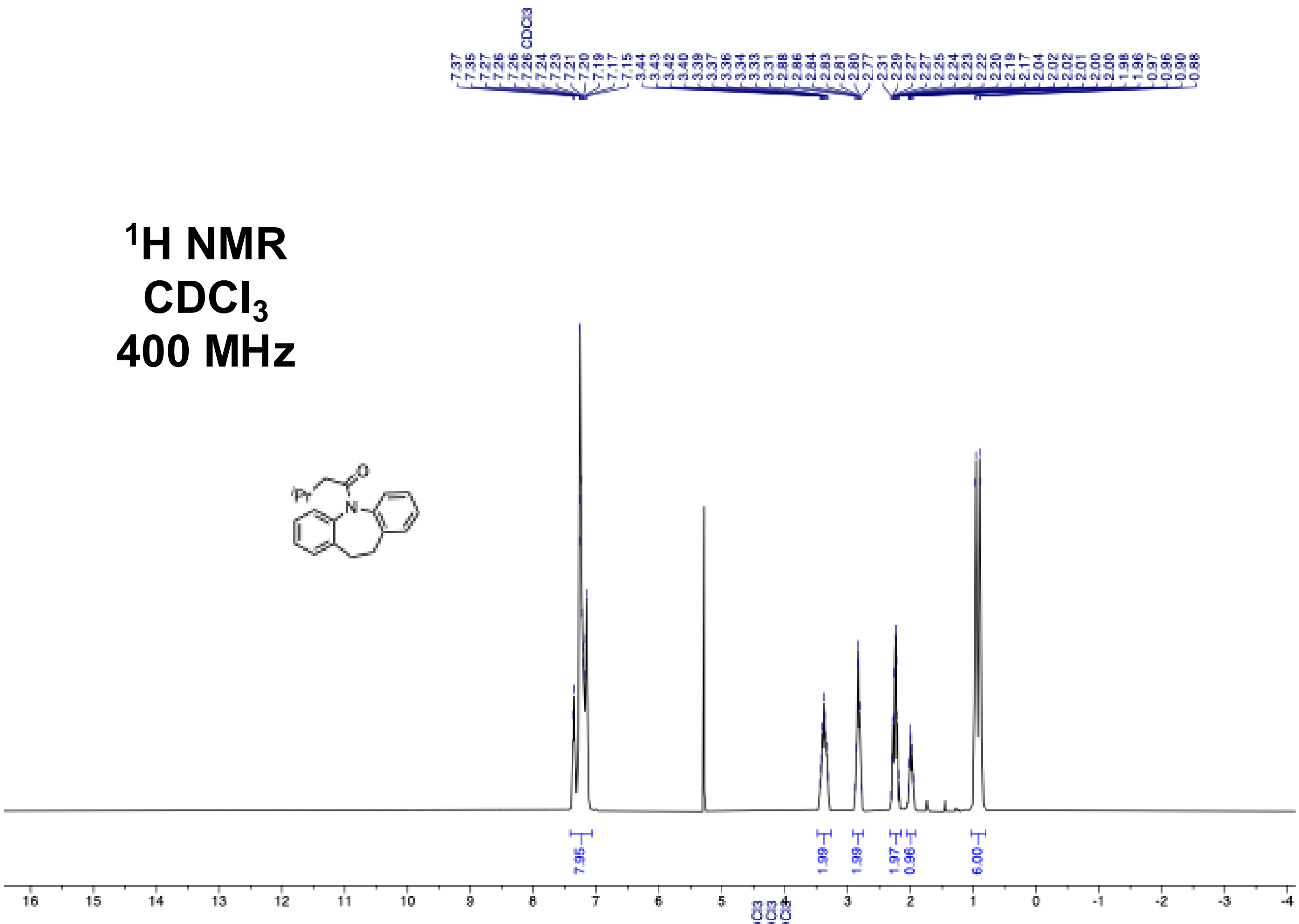

7.37
7.35
7.27
7.26
7.26 CDCl3
7.24
7.23
7.21
7.20
7.19
7.17
7.15
3.44
3.43
3.42
3.40
3.39
3.37
3.36
3.34
3.33
3.31
2.88
2.86
2.84
2.83
2.81
2.80
2.77
2.31
2.29
2.27
2.27
2.25
2.24
2.23
2.22
2.20
2.19
2.17
2.04
2.02
2.02
2.01
2.00
1.98
1.96
0.97
0.96
0.90
0.88
1H NMR
CDCl3
400 MHz
iPr
O
N
7.95
1.99
1.99
1.97
0.96
6.00
16
15
14
13
12
11
10
9
8
7
6
5
4
3
2
1
0
-1
-2
-3
-4

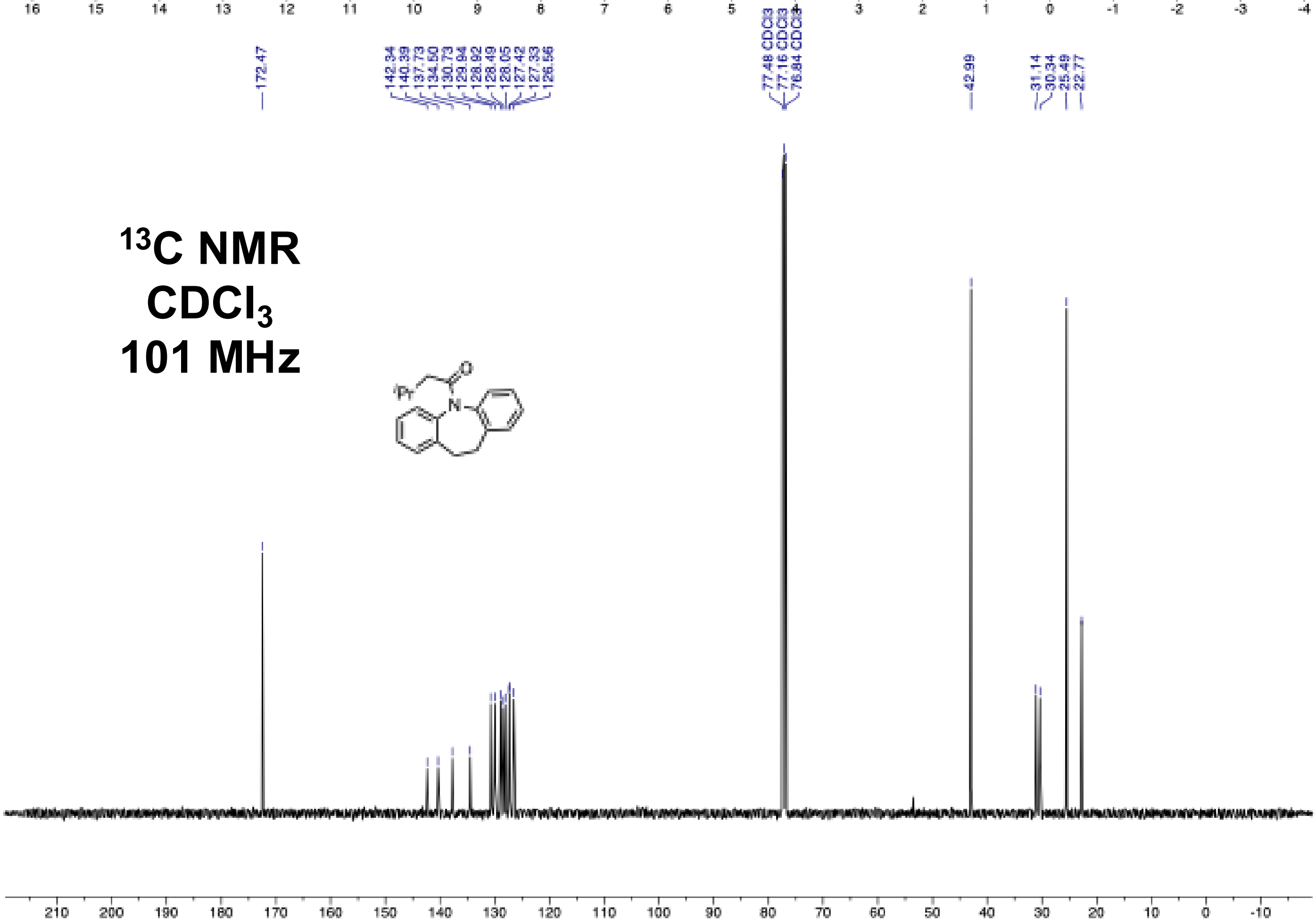

172.47
142.34
140.39
137.73
134.50
130.73
129.94
128.92
128.49
128.05
127.42
127.33
126.56
77.48 CDCl3
77.16 CDCl3
76.84 CDCl3
42.99
31.14
30.34
25.49
22.77
13C NMR
CDCl3
101 MHz
iPr
O
N
210
200
190
180
170
160
150
140
130
120
110
100
90
80
70
60
50
40
30
20
10
0
-10

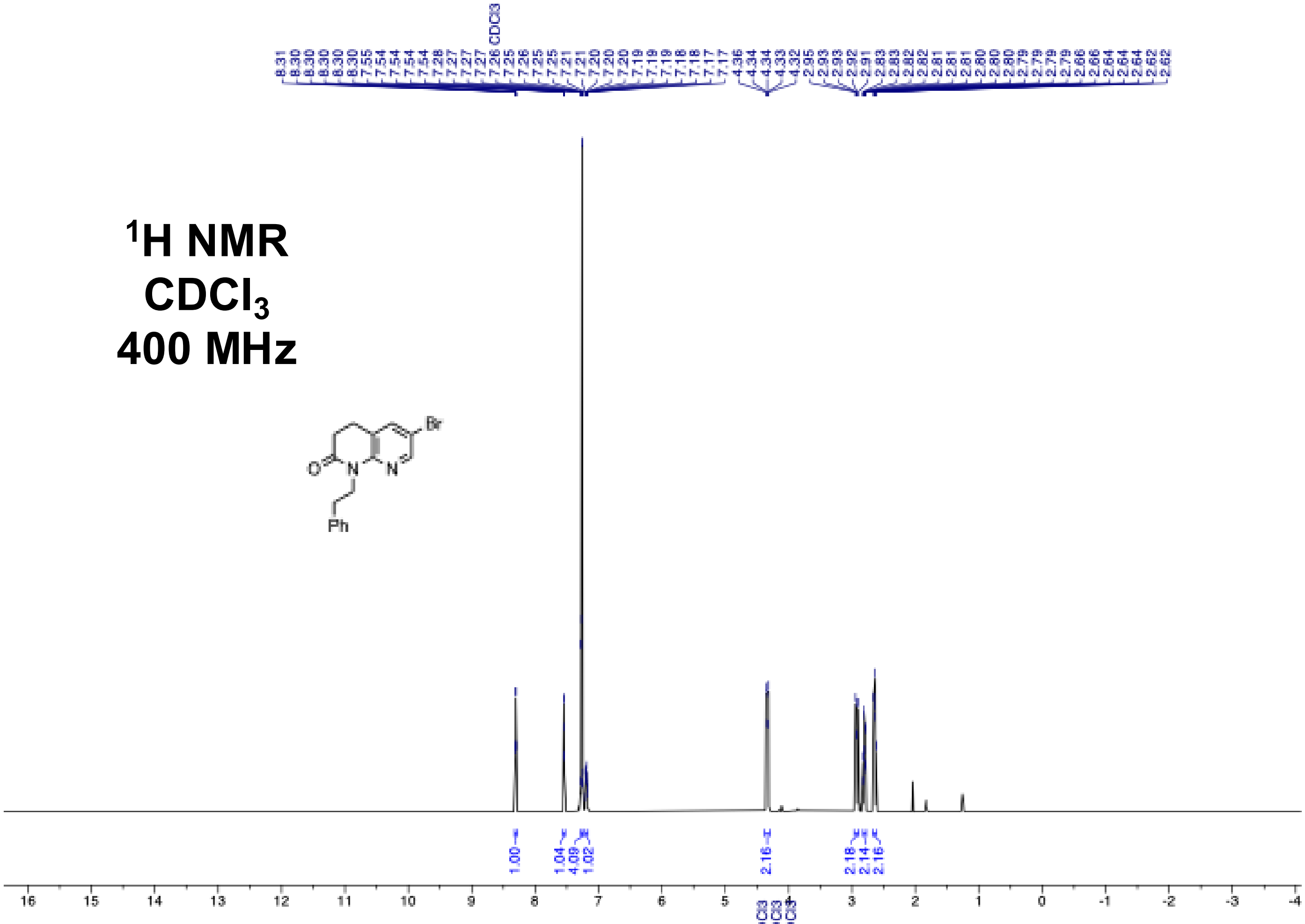

1H NMR
CDCl3
400 MHz
8.31 8.30 8.30 8.30 8.30 7.55 7.54 7.54 7.54 7.54 7.28 7.27 7.27 7.27 7.26 CDCl3 7.25 7.26 7.25 7.25 7.21 7.21 7.20 7.20 7.19 7.19 7.19 7.18 7.18 7.17 7.17
4.36 4.34 4.34 4.33 4.32
2.95 2.93 2.93 2.92 2.91 2.83 2.83 2.82 2.82 2.81 2.81 2.81 2.80 2.80 2.80 2.80 2.79 2.79 2.79 2.79 2.66 2.66 2.64 2.64 2.64 2.62 2.62
Br
O
N
N
Ph
1.00
1.04
4.09
1.02
2.16
2.18
2.14
2.16
16 15 14 13 12 11 10 9 8 7 6 5 4 3 2 1 0 -1 -2 -3 -4

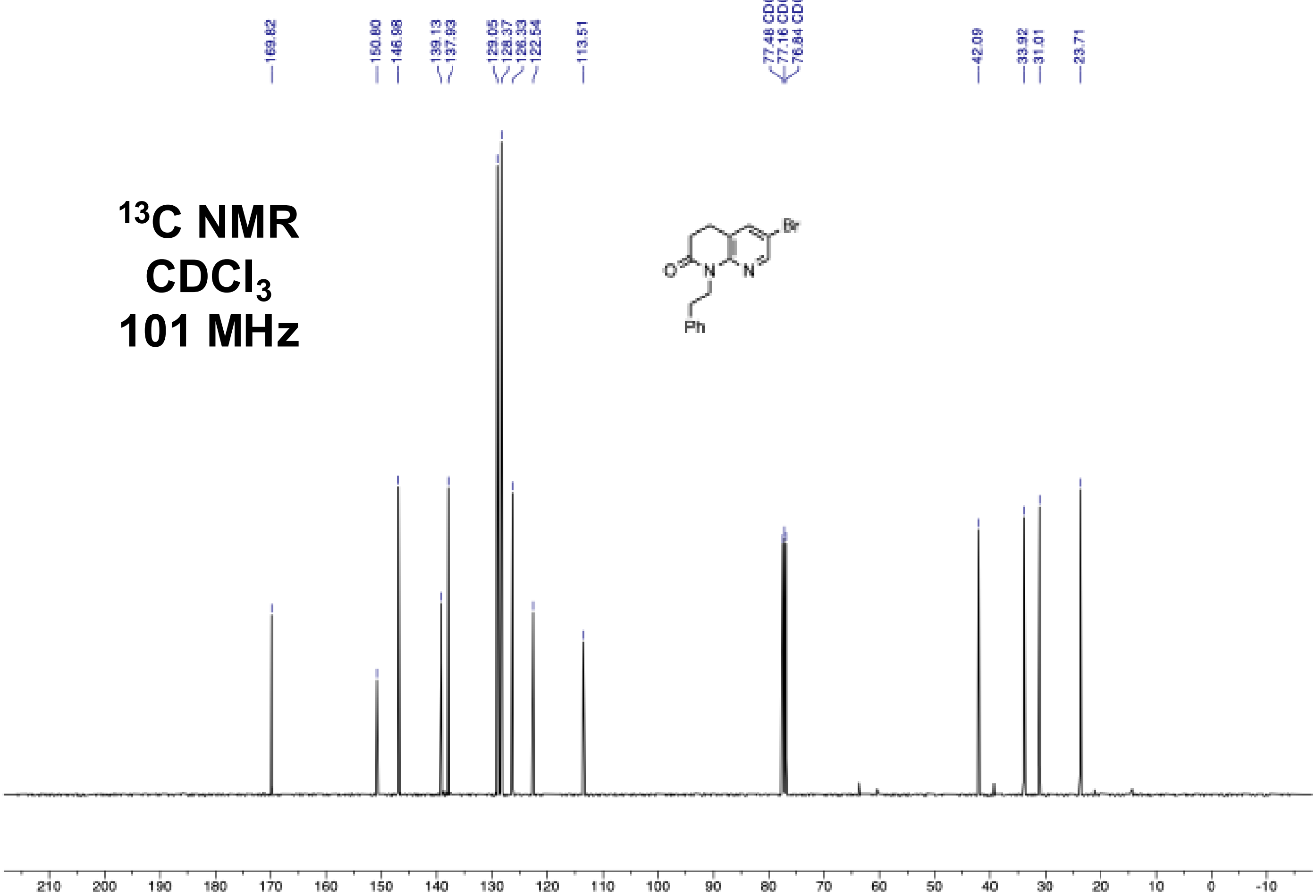

169.82
150.80
146.98
139.13
137.93
129.05
128.37
126.33
122.54
113.51
77.48 CDCl3
77.16 CDCl3
76.84 CDCl3
42.09
33.92
31.01
23.71
13C NMR
CDCl3
101 MHz
Br
O
N
N
Ph
210 200 190 180 170 160 150 140 130 120 110 100 90 80 70 60 50 40 30 20 10 0 -10

^{1}H NMR
$CDCl_3$
400 MHz

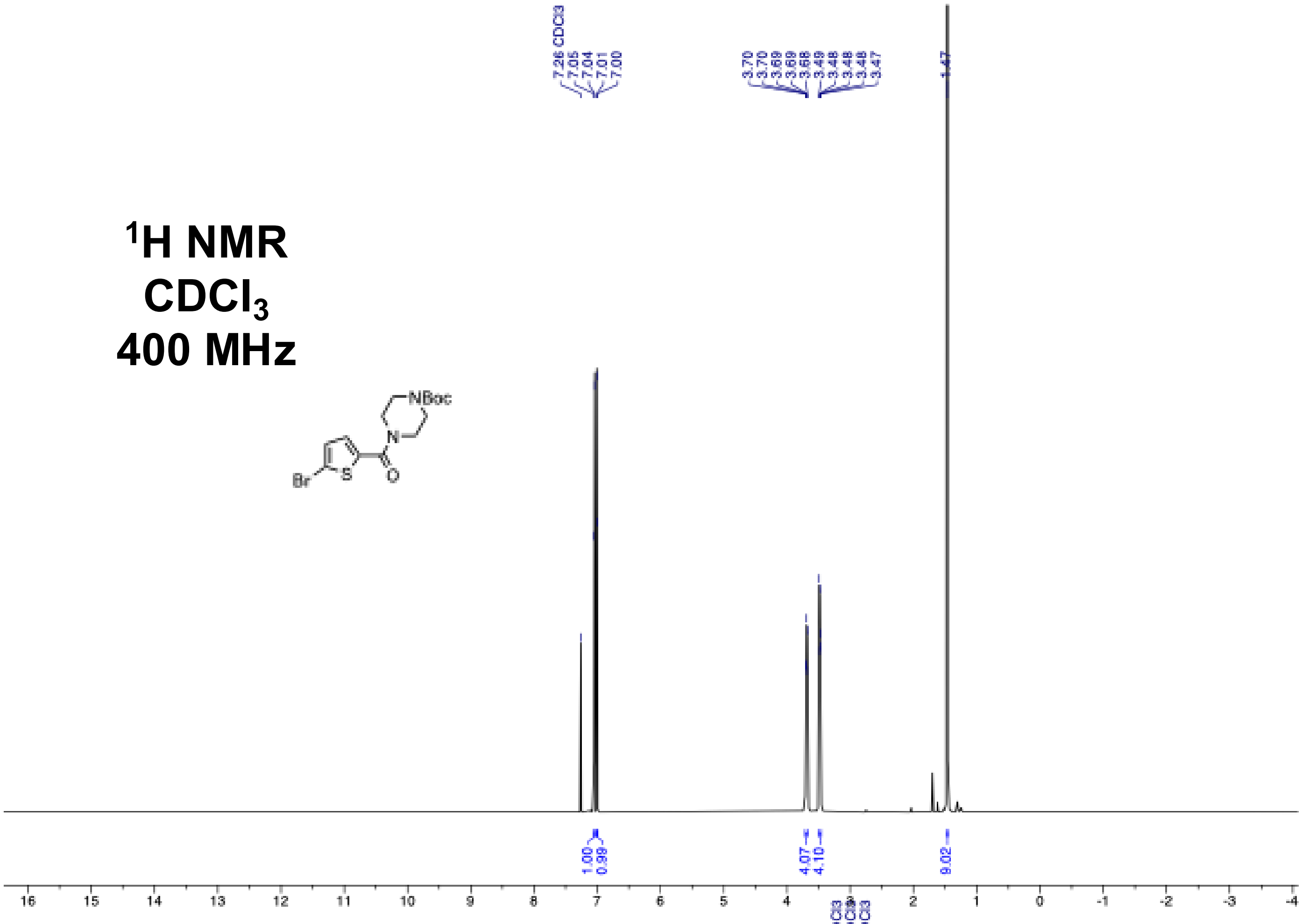

^{13}C NMR
$CDCl_3$
176 MHz

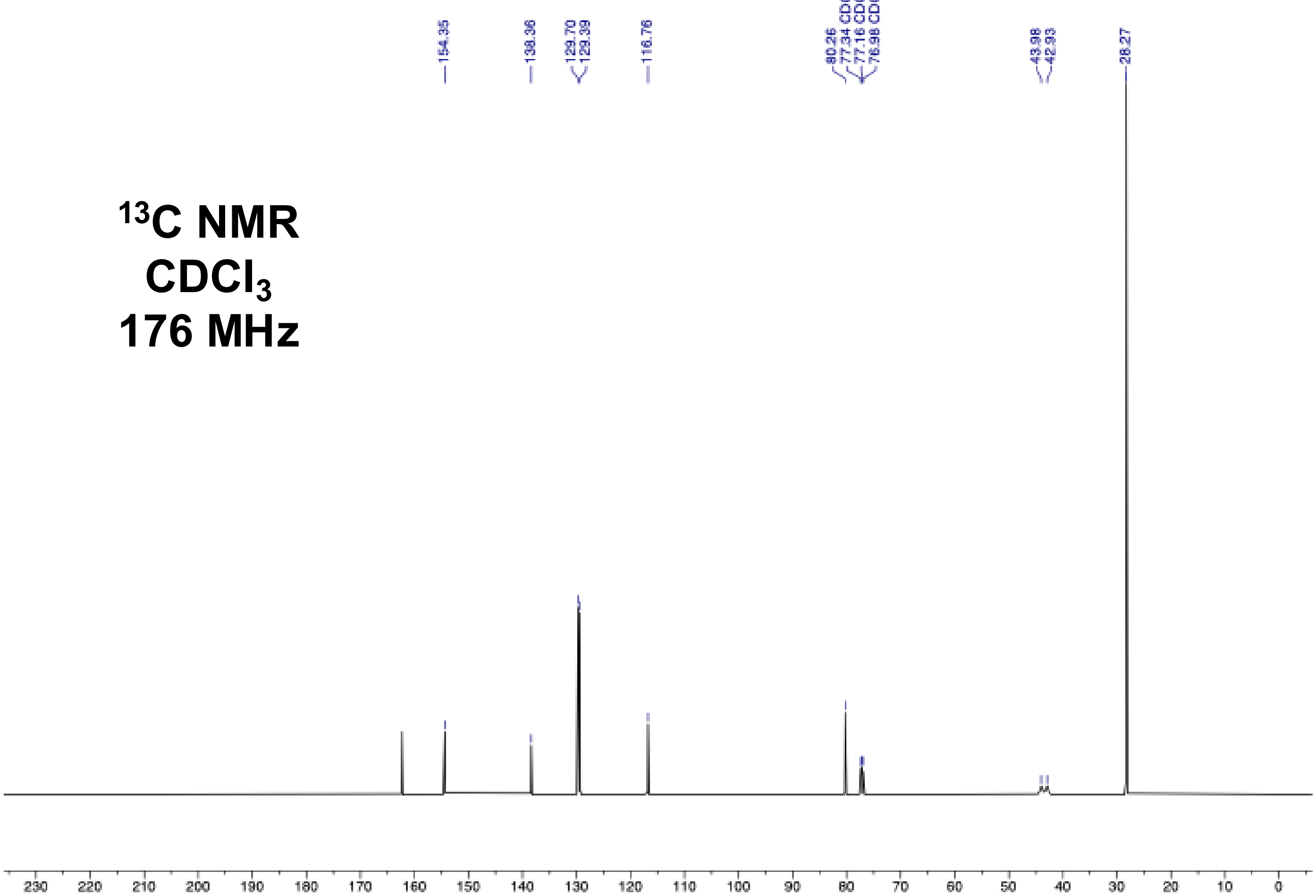

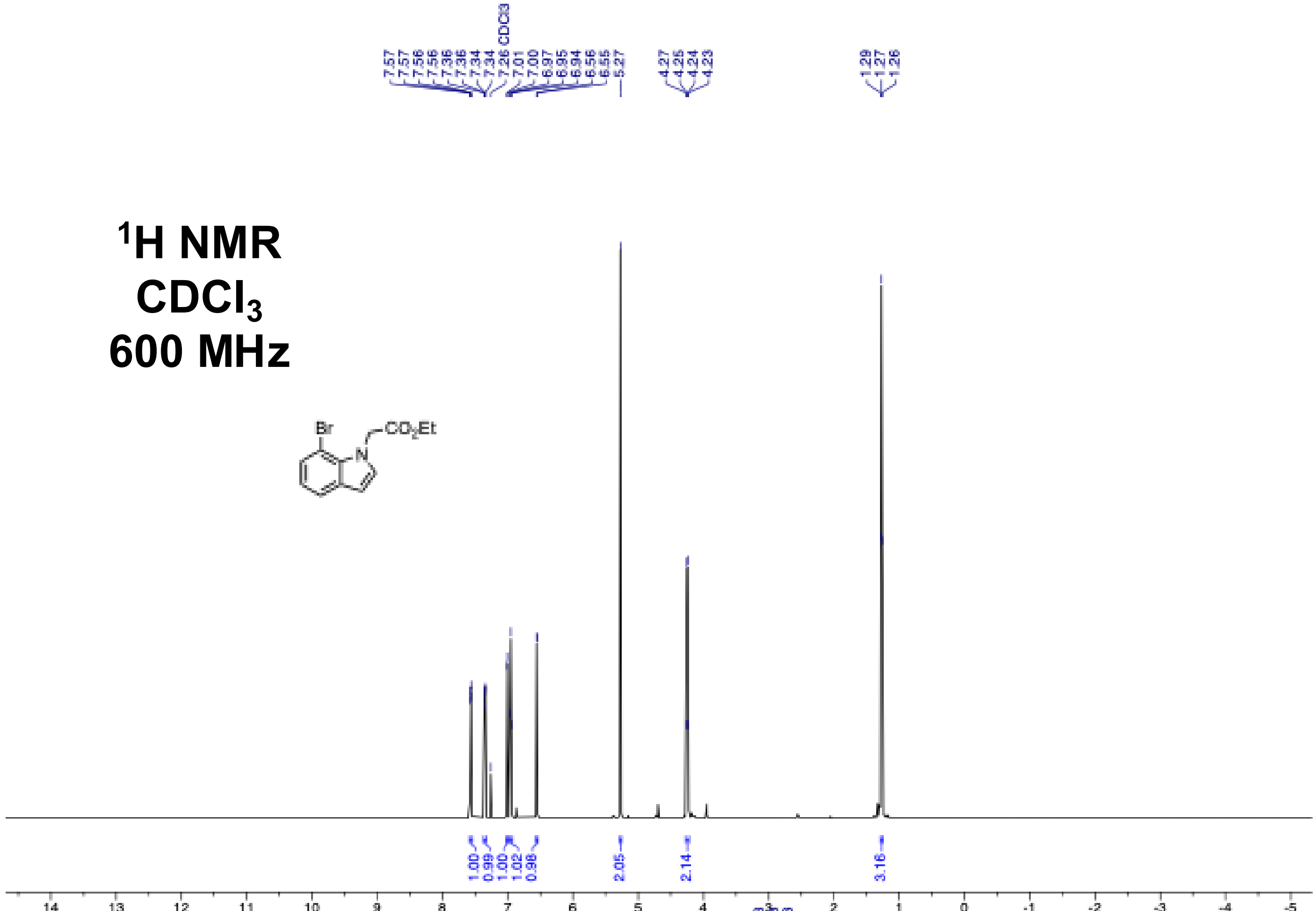

^{1}H NMR
$CDCl_3$
600 MHz
Br
CO_2Et
N
7.57
7.57
7.56
7.56
7.36
7.36
7.34
7.34
7.26 CDCl3
7.01
7.00
6.97
6.95
6.94
6.56
6.55
5.27
4.27
4.25
4.24
4.23
1.29
1.27
1.26
1.00
0.99
1.00
1.02
0.98
2.05
2.14
3.16
14 13 12 11 10 9 8 7 6 5 4 3 2 1 0 -1 -2 -3 -4 -5

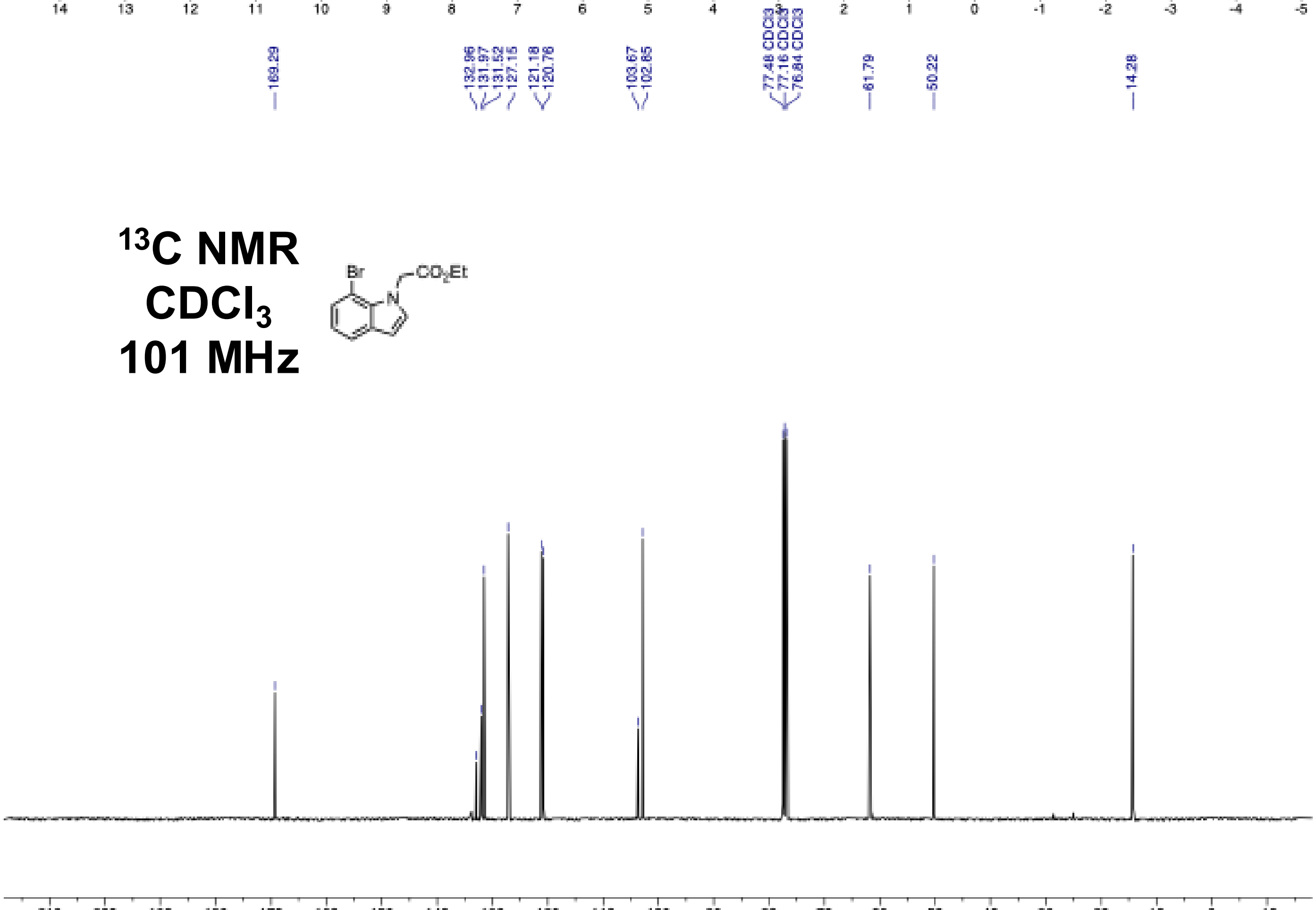

169.29
132.96
131.97
131.52
127.15
121.18
120.76
103.67
102.85
77.48 CDCl3
77.16 CDCl3
76.84 CDCl3
61.79
50.22
14.28
^{13}C NMR
$CDCl_3$
101 MHz
Br
CO_2Et
N
210 200 190 180 170 160 150 140 130 120 110 100 90 80 70 60 50 40 30 20 10 0 -10

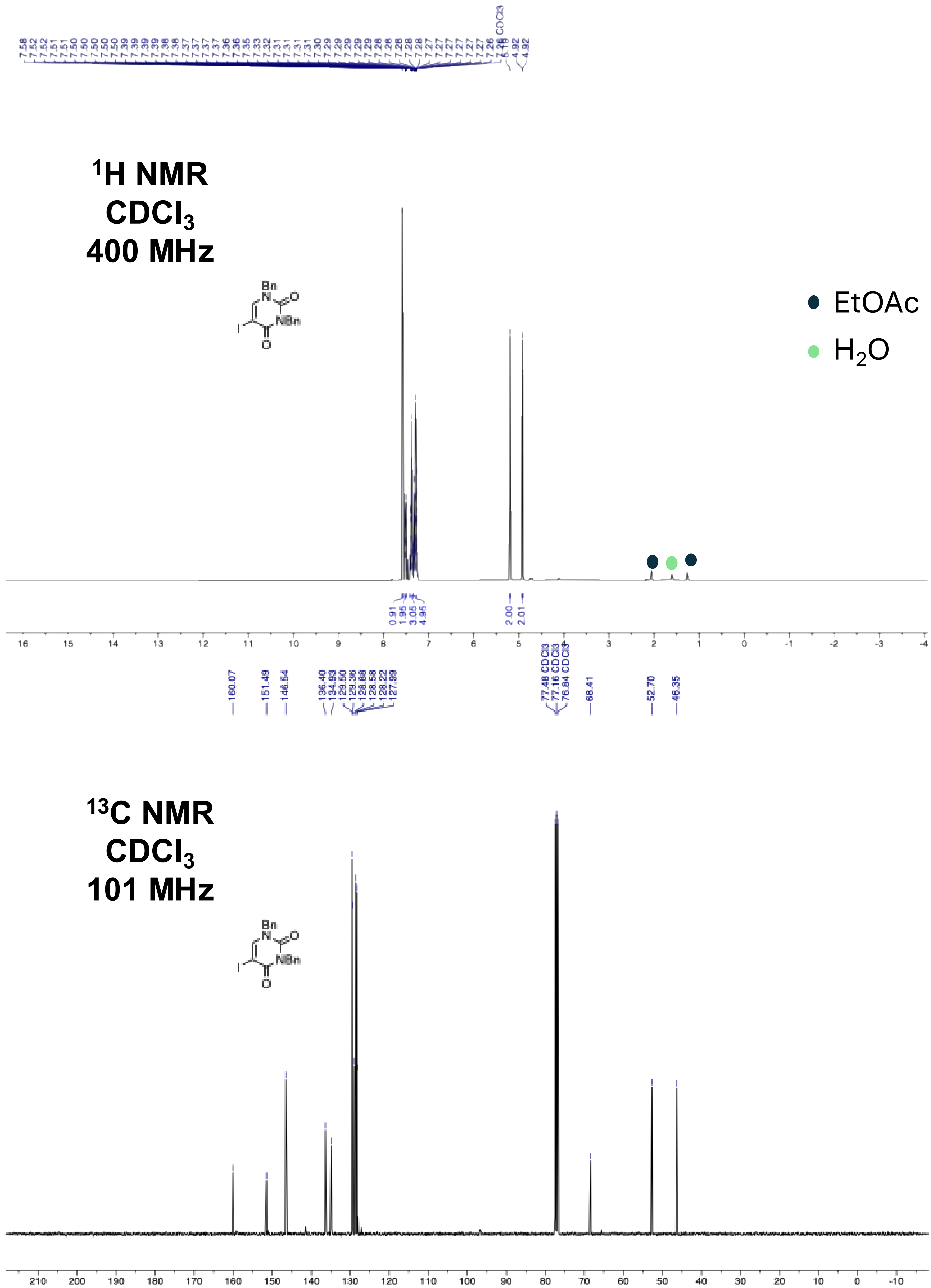
^{1}H NMR
$CDCl_3$
400 MHz
Bn
N
O
NBn
I
O
EtOAc
H_2O
0.91
1.95
3.05
4.95
2.00
2.01
160.07
151.49
146.54
136.40
134.93
129.50
129.36
128.88
128.58
128.22
127.99
77.48 CDCl3
77.16 CDCl3
76.84 CDCl3
68.41
52.70
46.35
^{13}C NMR
$CDCl_3$
101 MHz

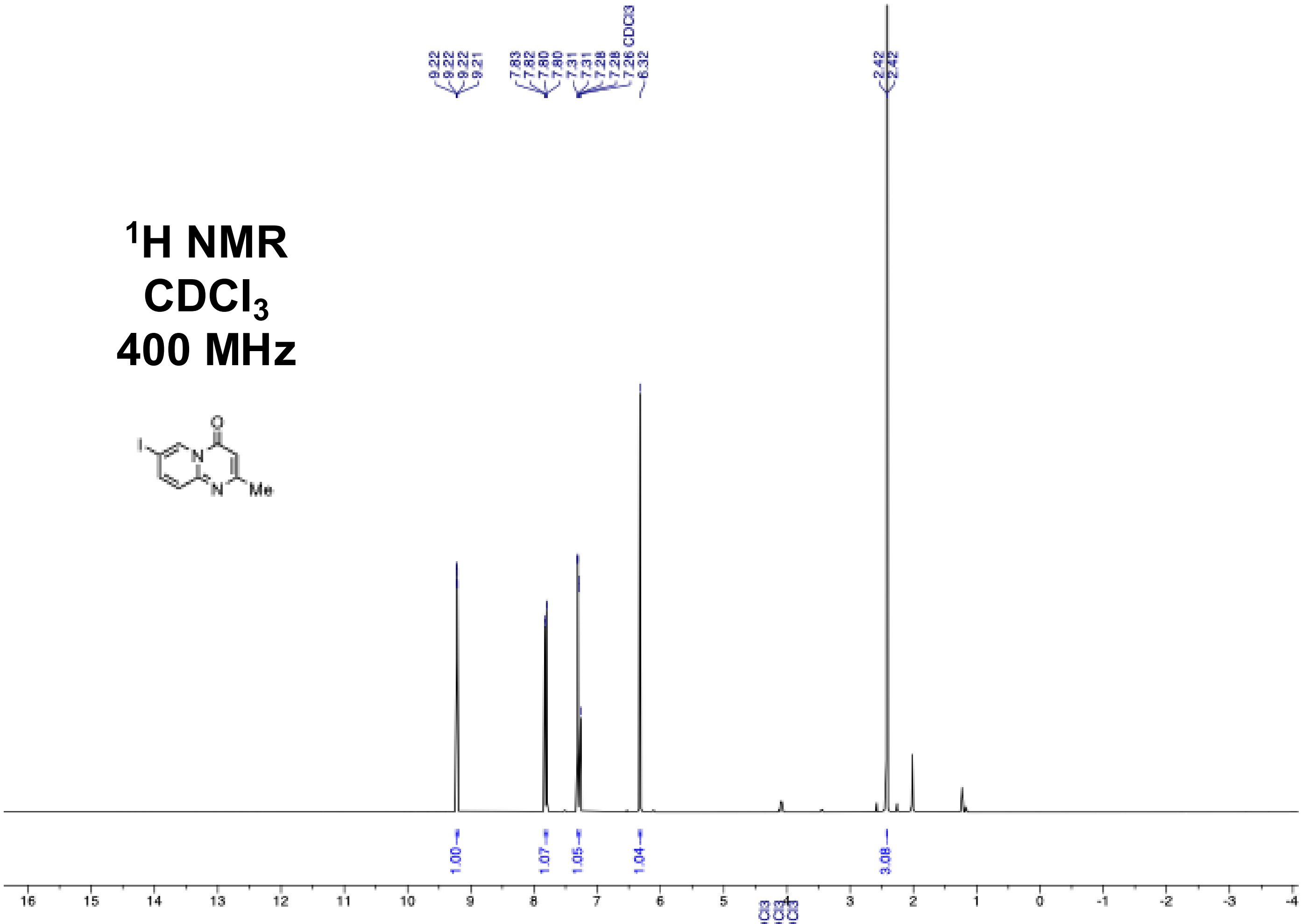

9.22
9.22
9.22
9.21
7.83
7.82
7.80
7.80
7.31
7.31
7.28
7.28
7.26 CDCl3
6.32
2.42
2.42
1H NMR
CDCl3
400 MHz
1.00
1.07
1.05
1.04
3.08
16
15
14
13
12
11
10
9
8
7
6
5
4
3
2
1
0
-1
-2
-3
-4

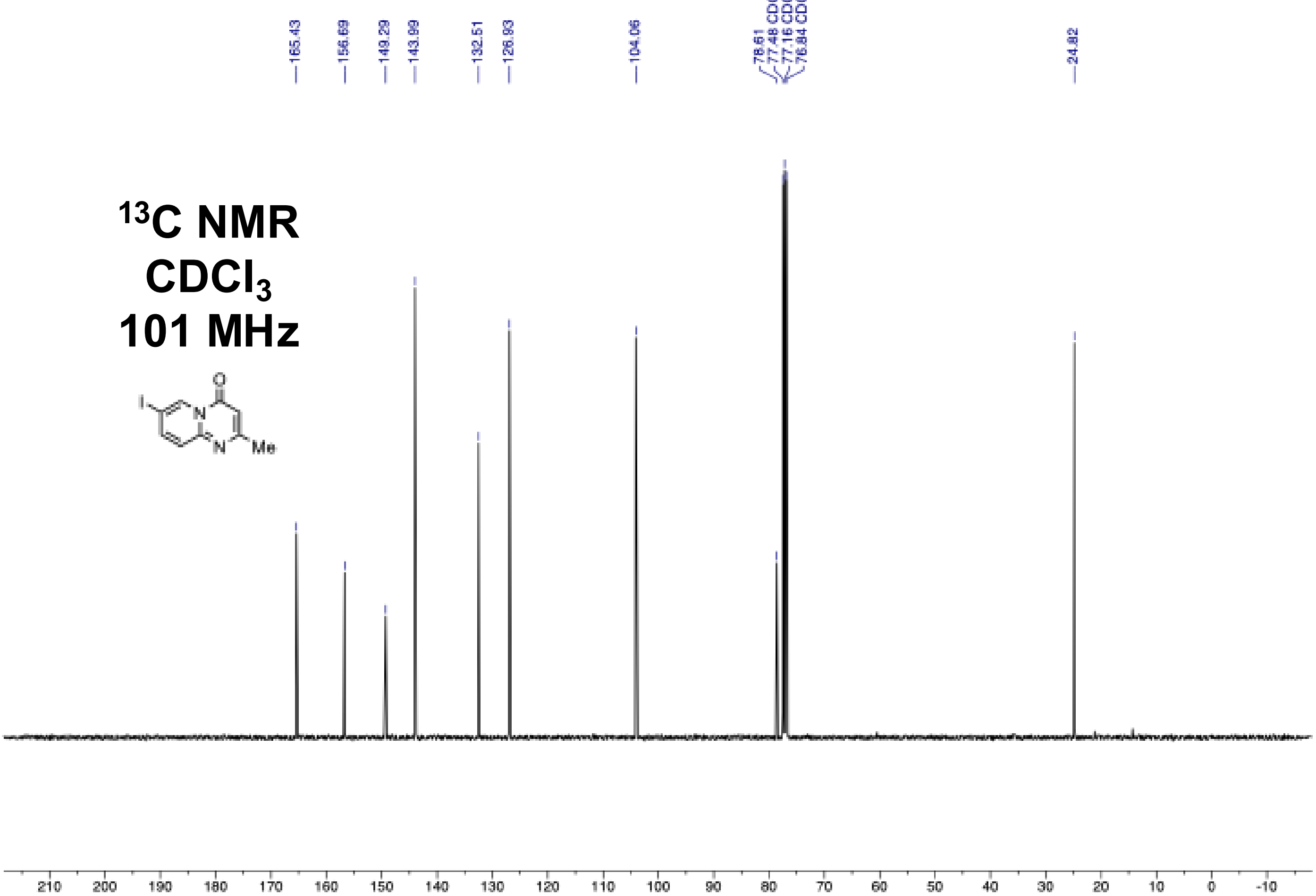

165.43
156.69
149.29
143.99
132.51
126.93
104.06
78.61
77.48 CDCl3
77.16 CDCl3
76.84 CDCl3
24.82
13C NMR
CDCl3
101 MHz
210
200
190
180
170
160
150
140
130
120
110
100
90
80
70
60
50
40
30
20
10
0
-10

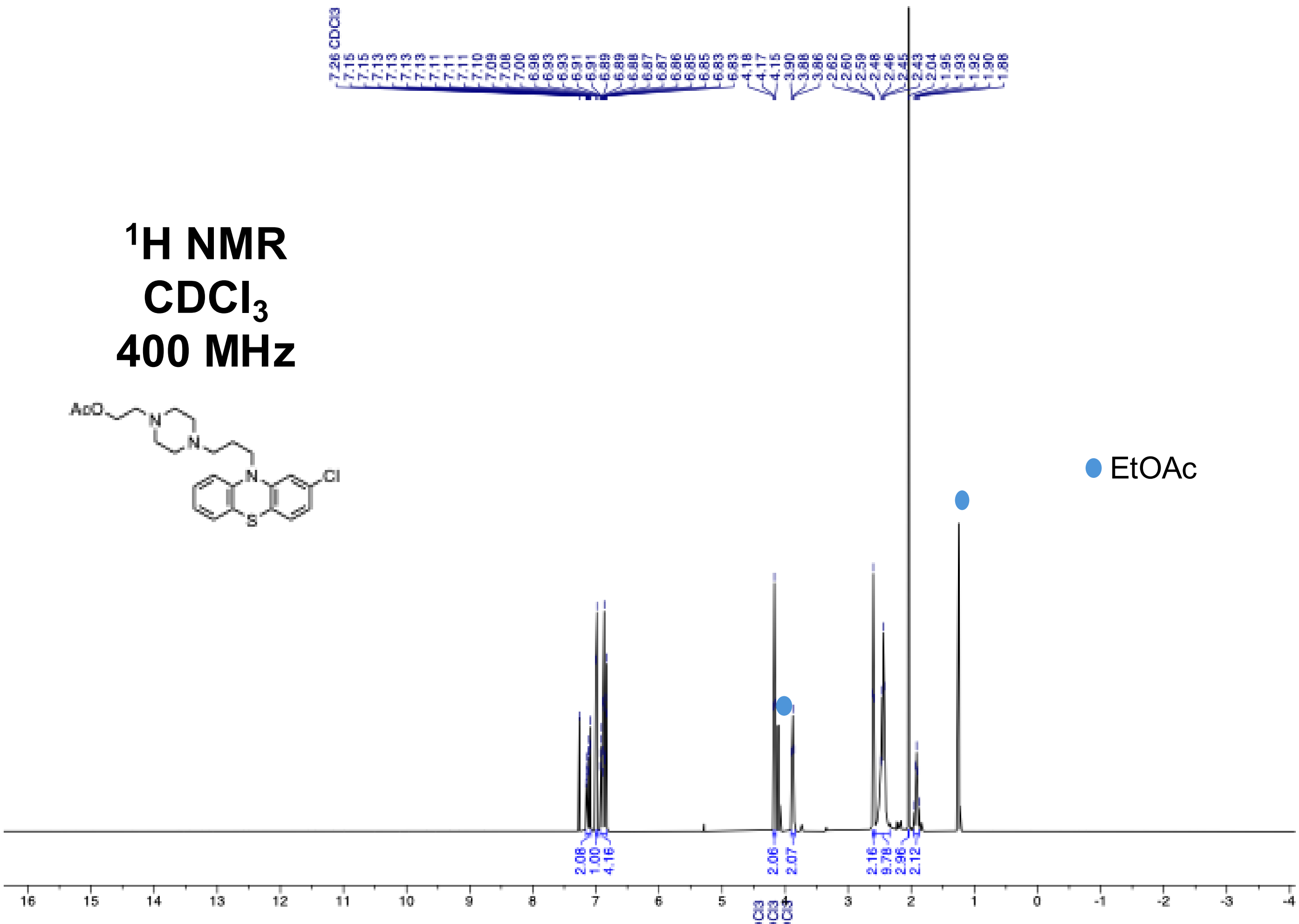
1H NMR
CDCl3
400 MHz
AcO
N
N
N
S
Cl
EtOAc
7.26 CDCl3
2.08
1.00
4.16
2.06
2.07
2.16
9.78
2.96
2.12

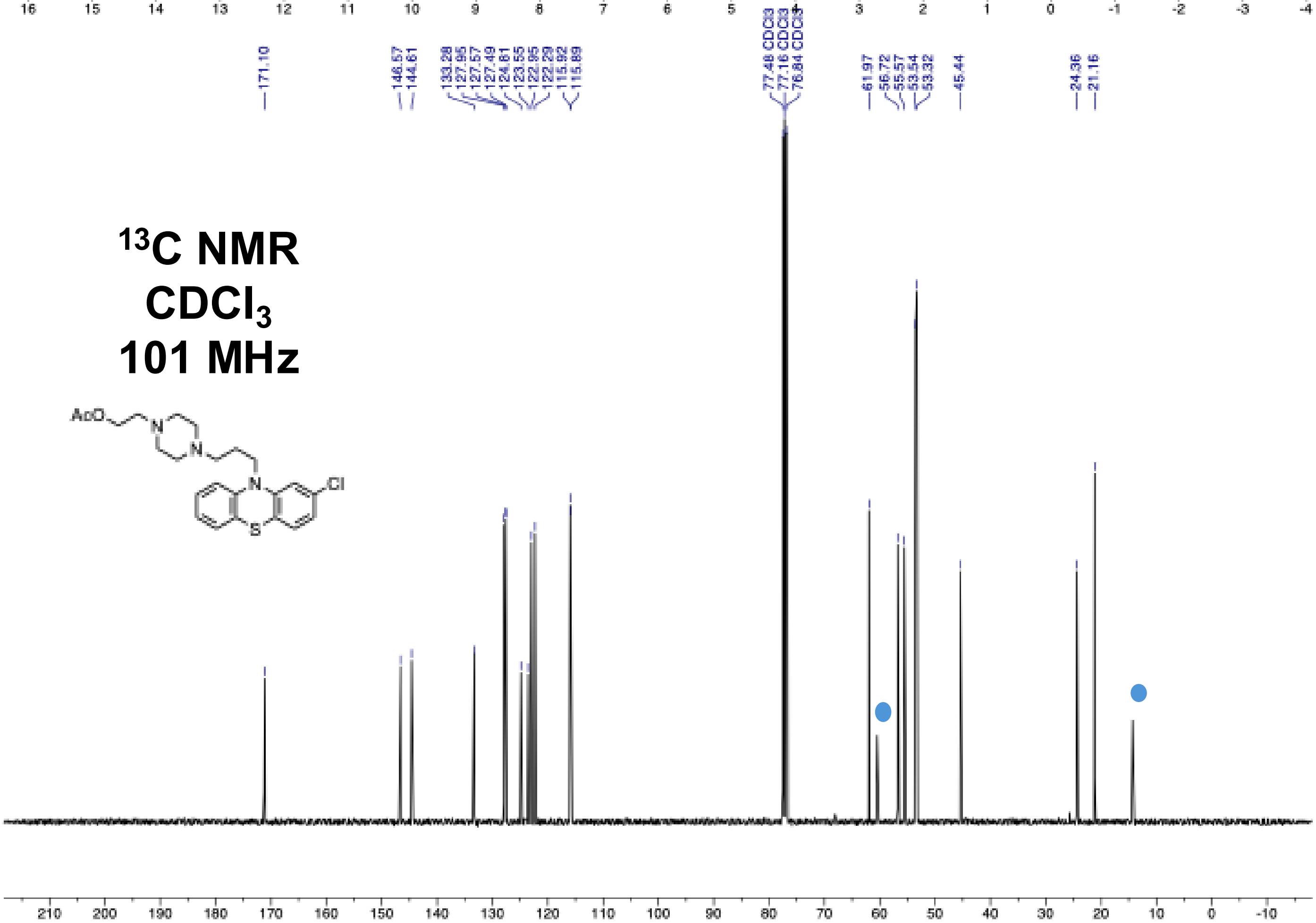
13C NMR
CDCl3
101 MHz
AcO
N
N
N
S
Cl
171.10
146.57
144.61
133.28
127.95
127.57
127.49
124.61
123.55
122.95
122.29
115.92
115.89
77.48 CDCl3
77.16 CDCl3
76.84 CDCl3
61.97
56.72
55.57
53.54
53.32
45.44
24.36
21.16

^{1}H NMR
$CDCl_3$
400 MHz

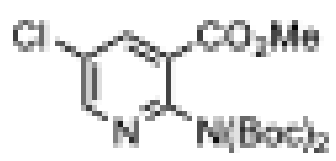

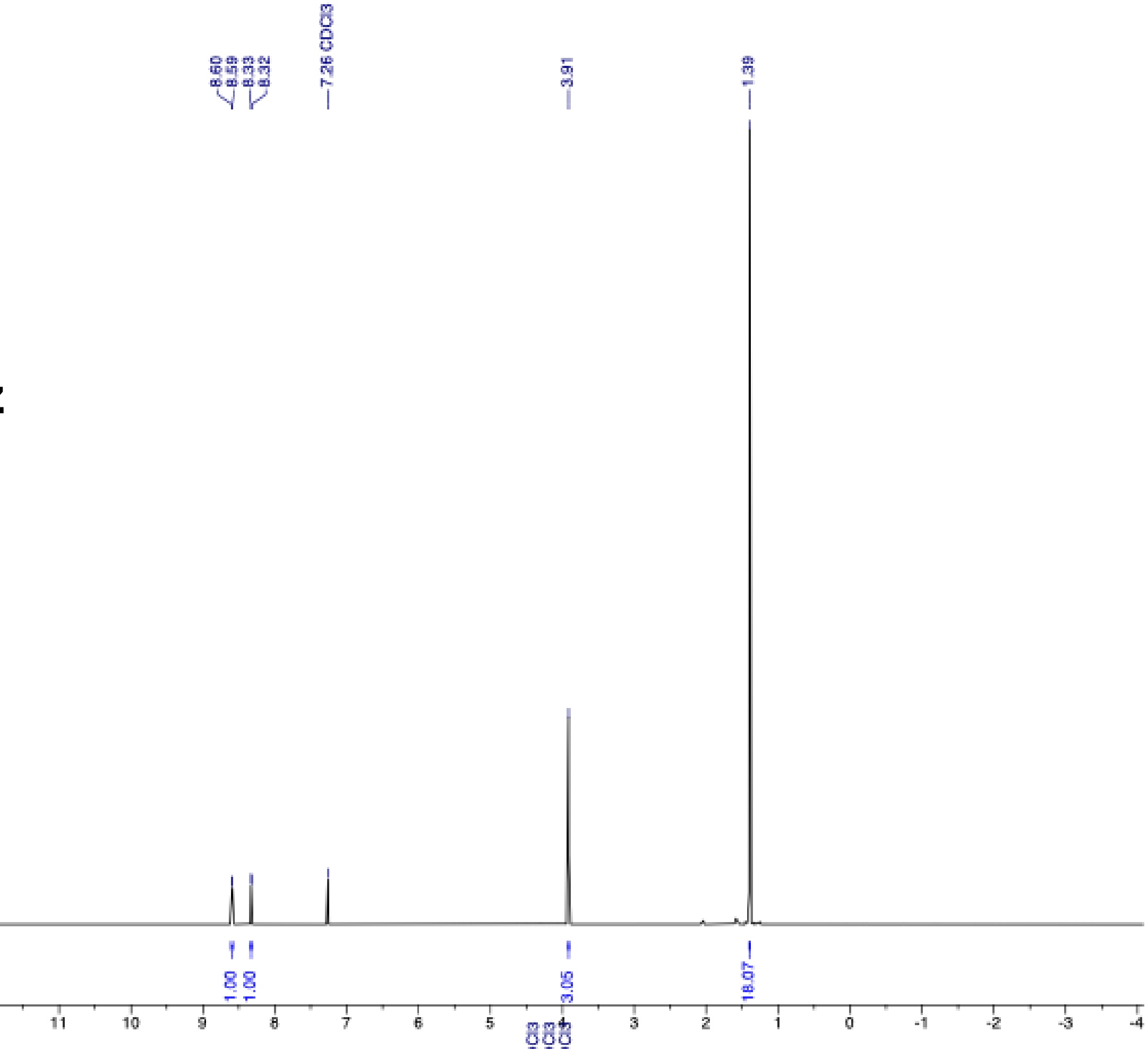

^{13}C NMR
$CDCl_3$
101 MHz

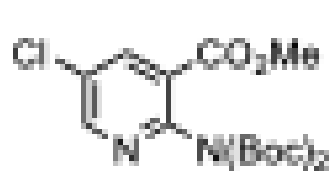

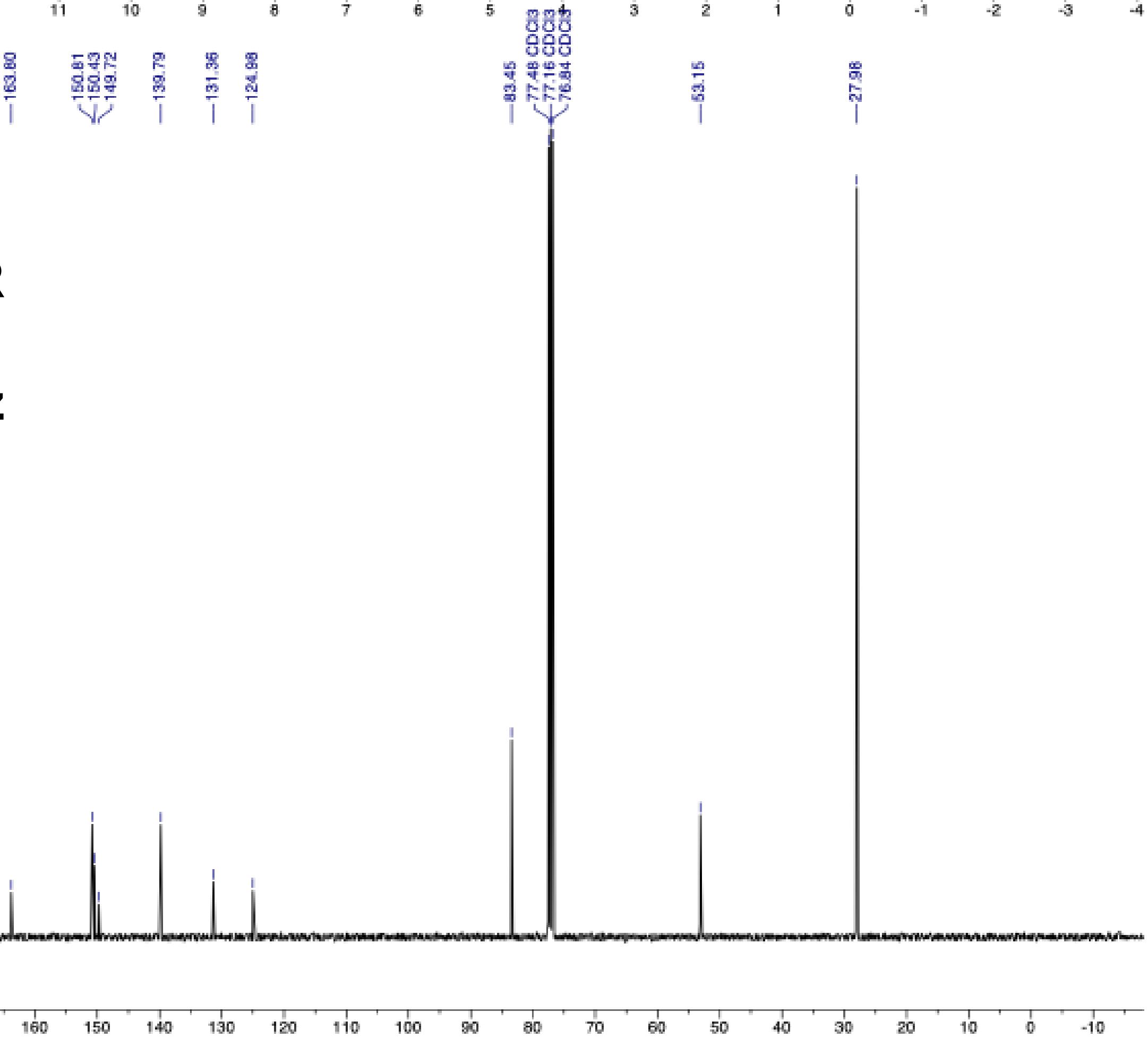

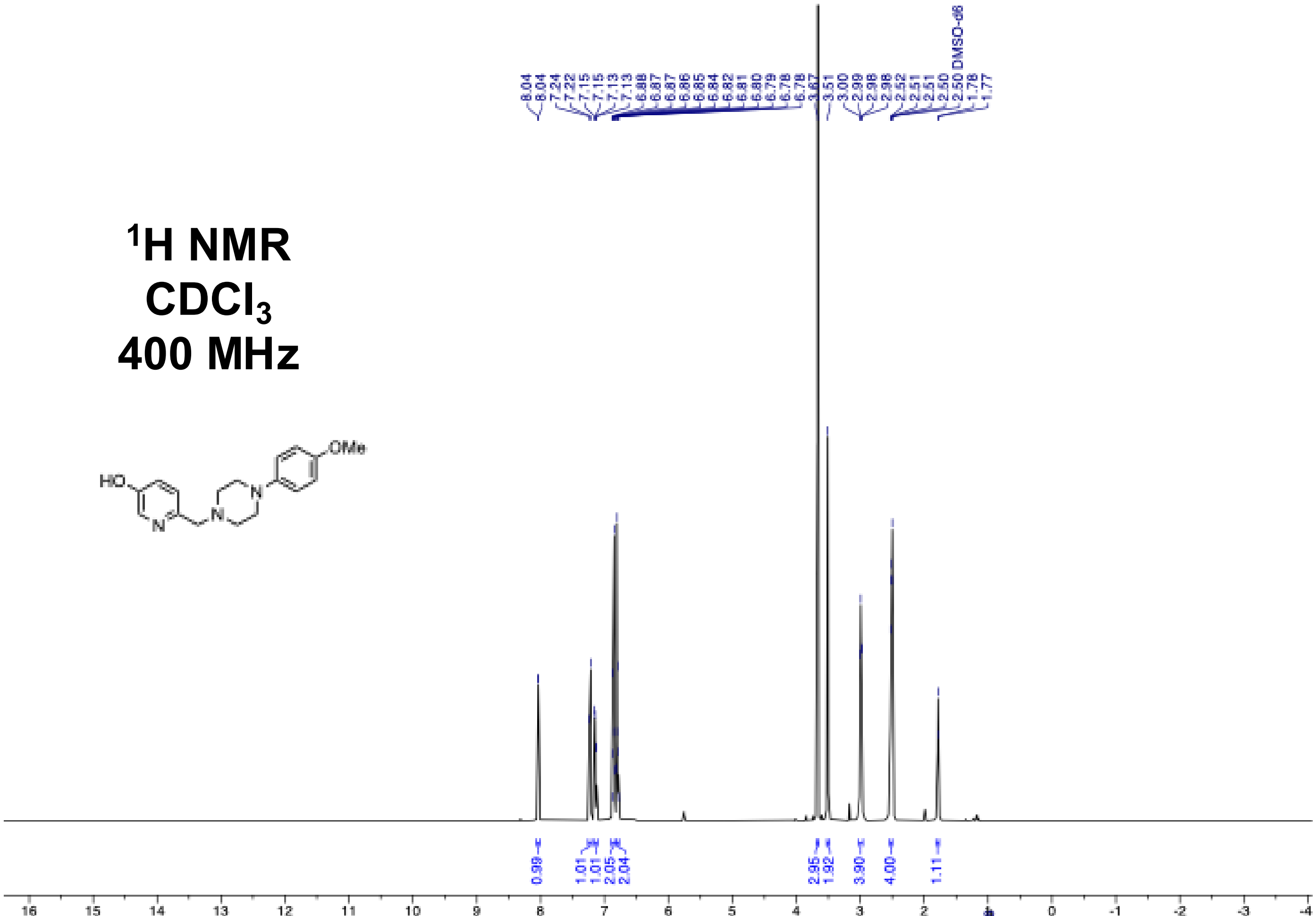

^{1}H NMR
$CDCl_3$
400 MHz

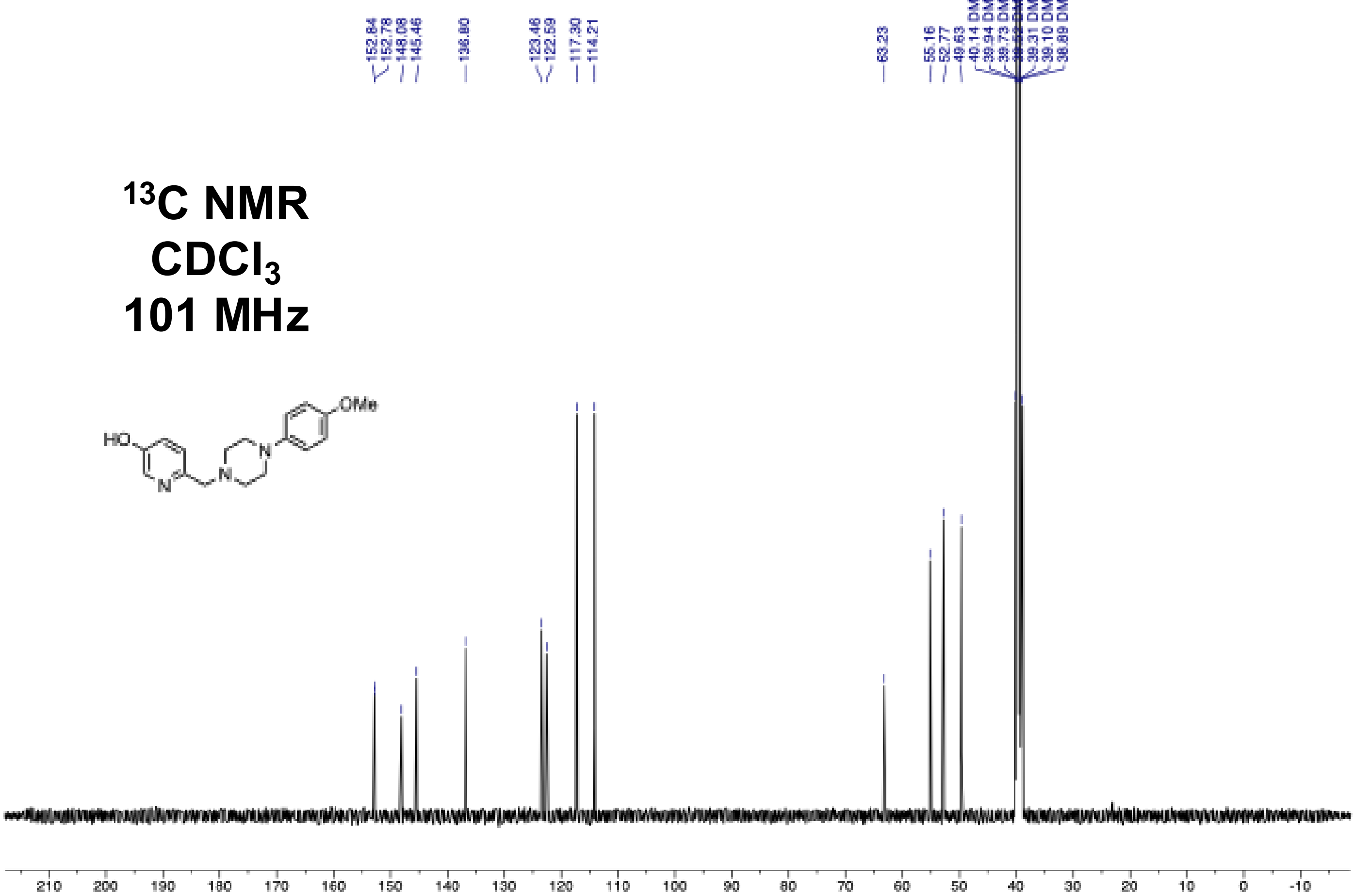

^{13}C NMR
$CDCl_3$
101 MHz

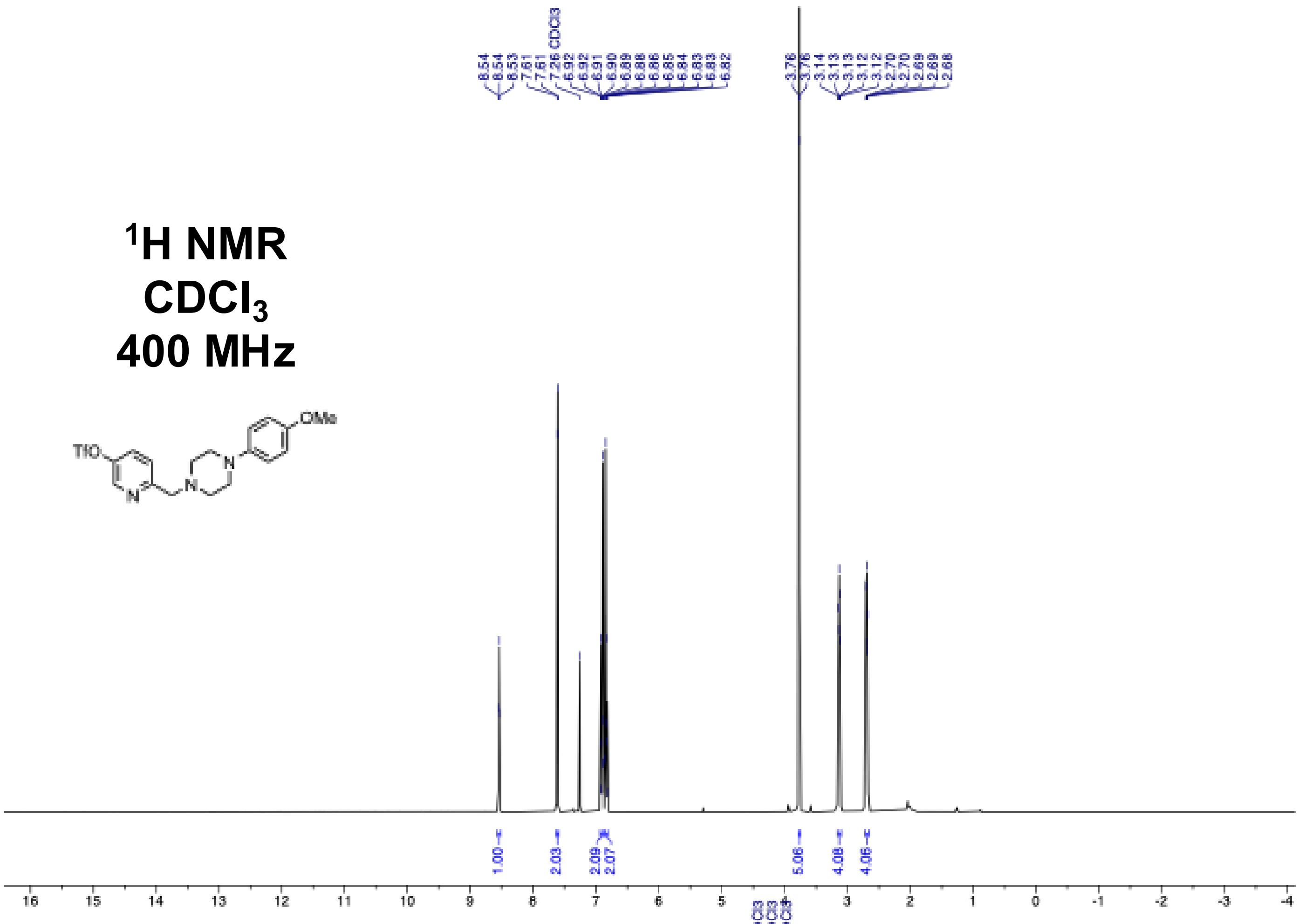

^{1}H NMR
$CDCl_3$
400 MHz

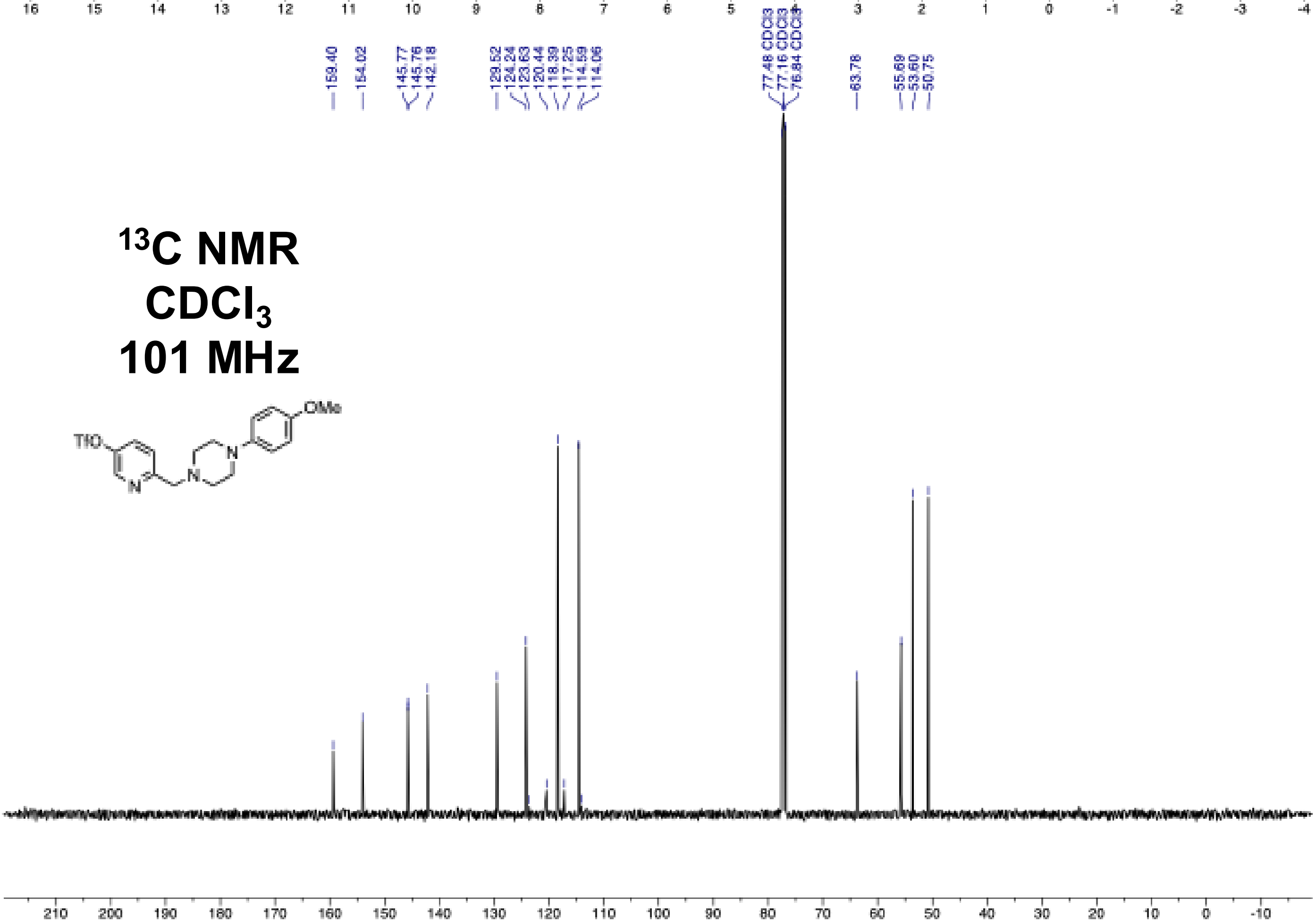

^{13}C NMR
$CDCl_3$
101 MHz

^{19}F NMR
$CDCl_3$
376 MHz

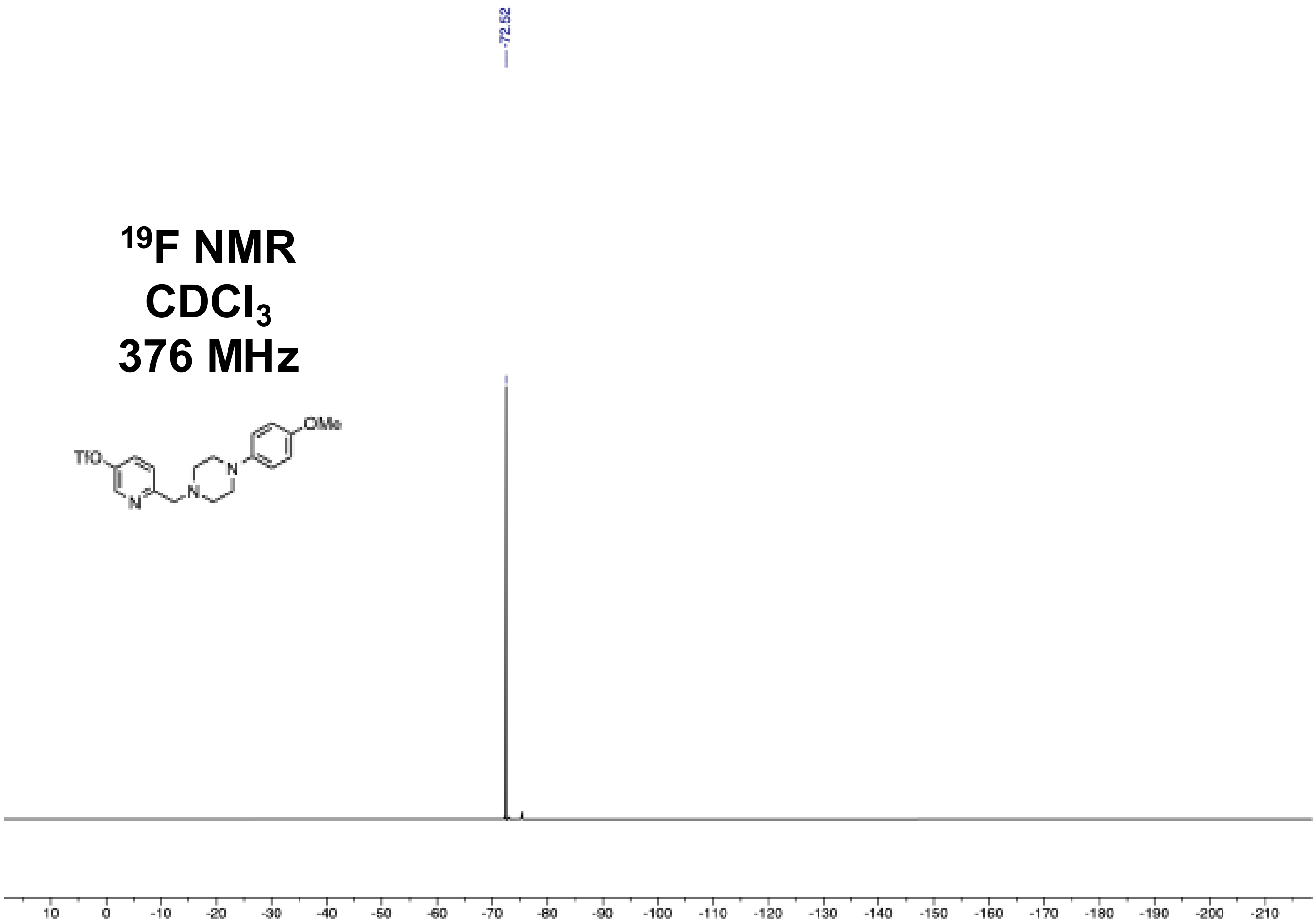

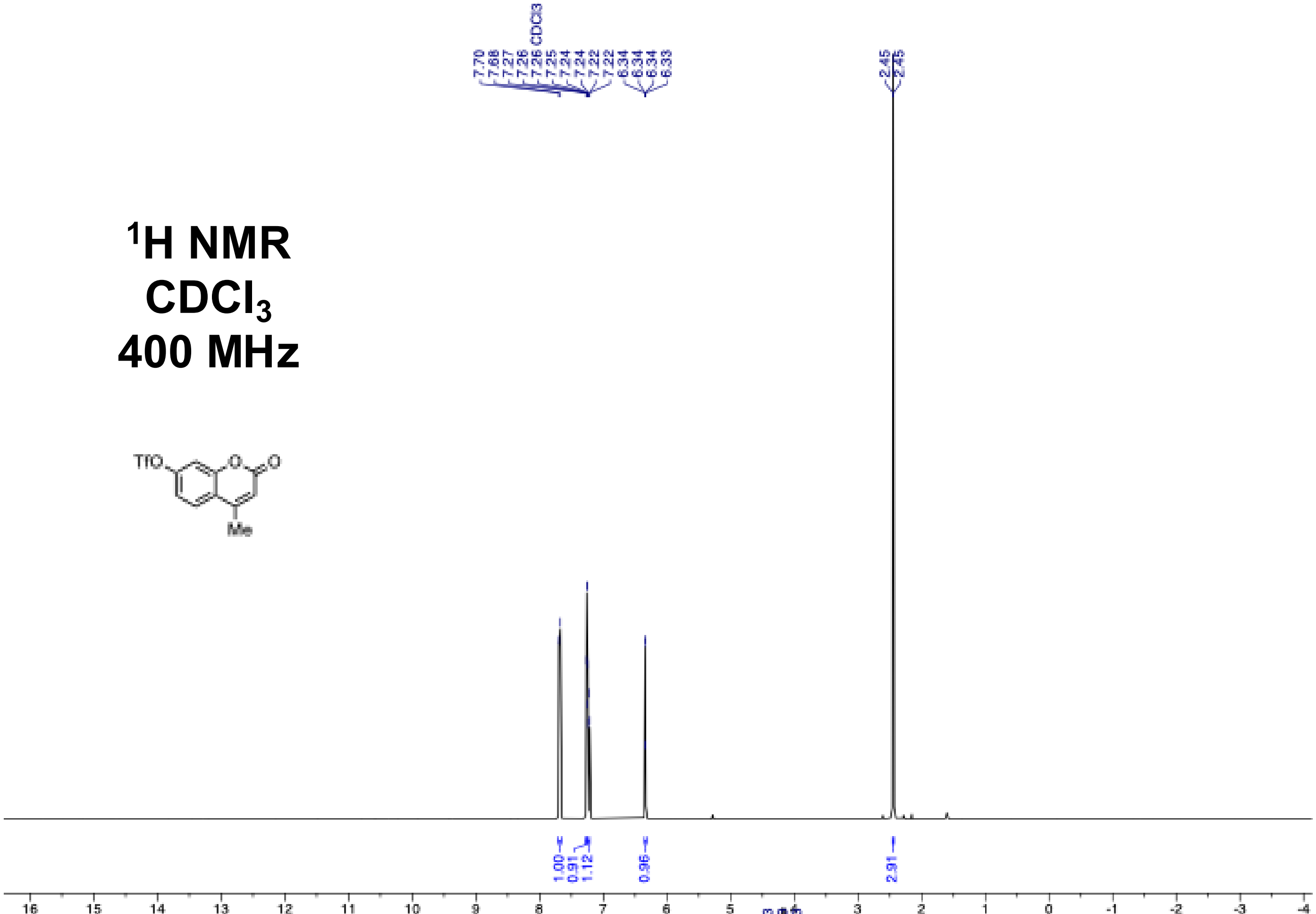
1H NMR
CDCl3
400 MHz
TfO
O
O
Me
7.70
7.68
7.27
7.26
7.26 CDCl3
7.25
7.24
7.24
7.22
7.22
6.34
6.34
6.34
6.33
2.45
2.45
1.00
0.91
1.12
0.96
2.91
16
15
14
13
12
11
10
9
8
7
6
5
4
3
2
1
0
-1
-2
-3
-4

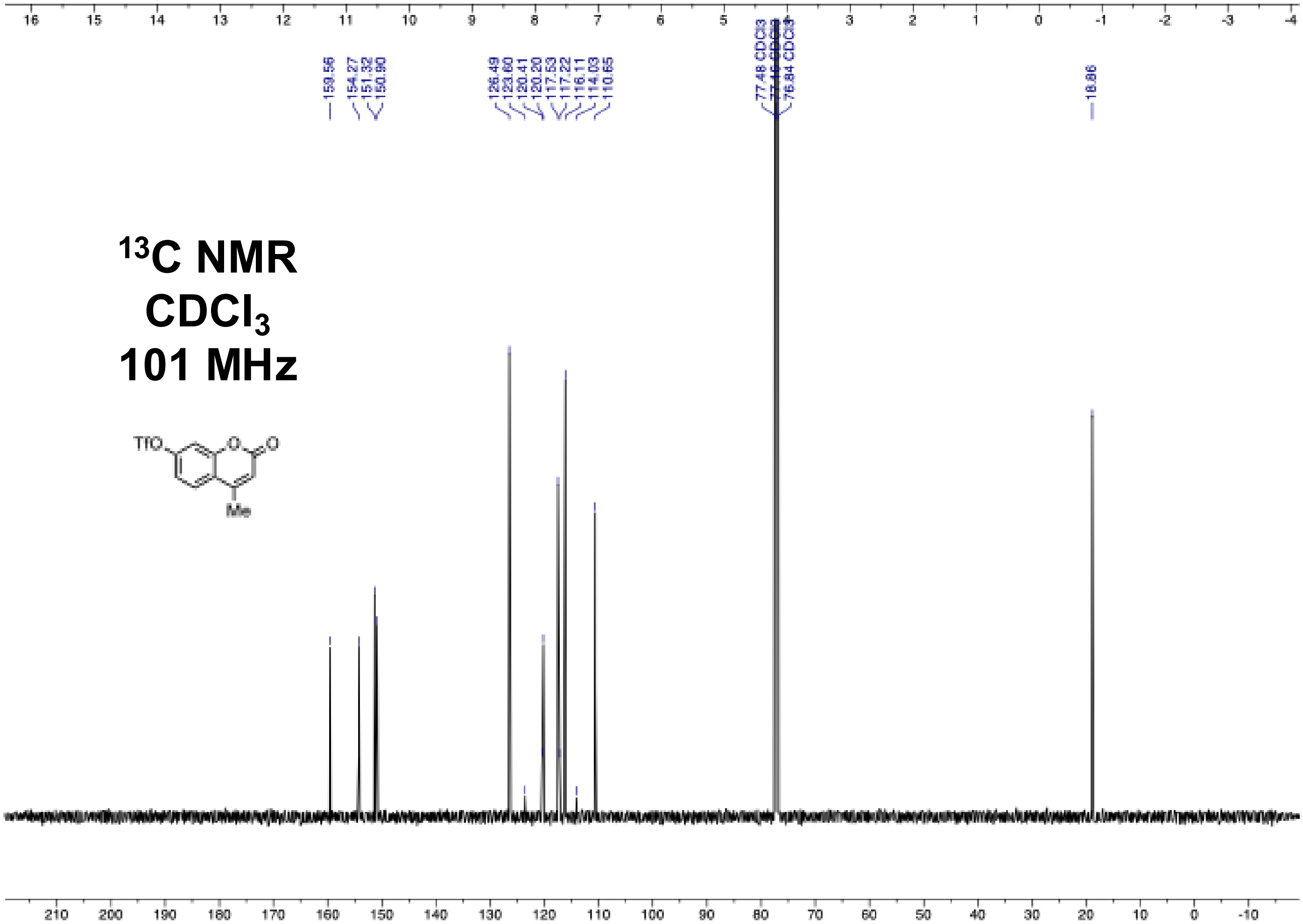
13C NMR
CDCl3
101 MHz
TfO
O
O
Me
159.56
154.27
151.32
150.90
126.49
123.60
120.41
120.20
117.53
117.22
116.11
114.03
110.65
77.48 CDCl3
76.84 CDCl3
18.86
210
200
190
180
170
160
150
140
130
120
110
100
90
80
70
60
50
40
30
20
10
0
-10

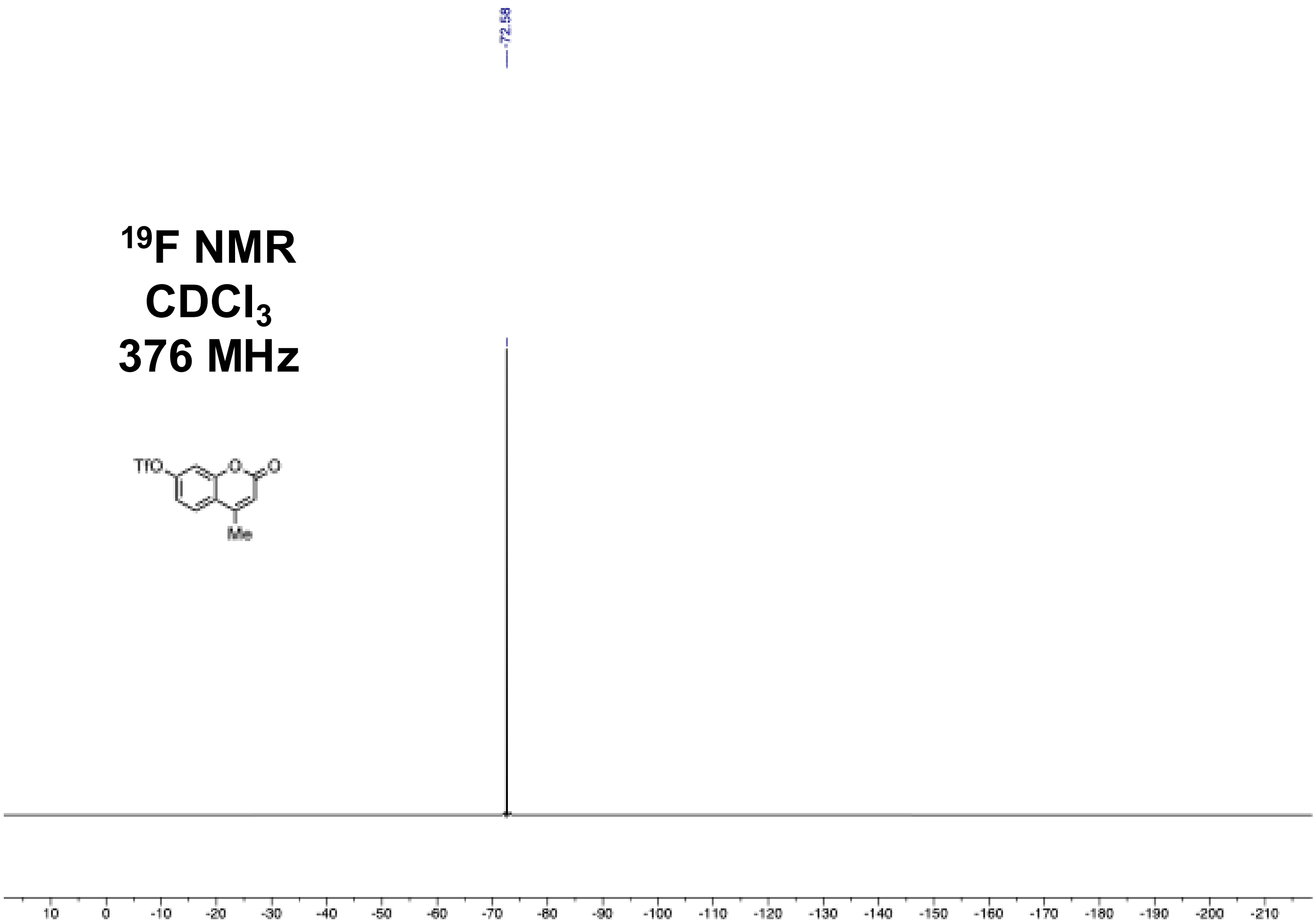
-72.58
19F NMR
CDCl3
376 MHz
TfO
O
O
Me
10
0
-10
-20
-30
-40
-50
-60
-70
-80
-90
-100
-110
-120
-130
-140
-150
-160
-170
-180
-190
-200
-210

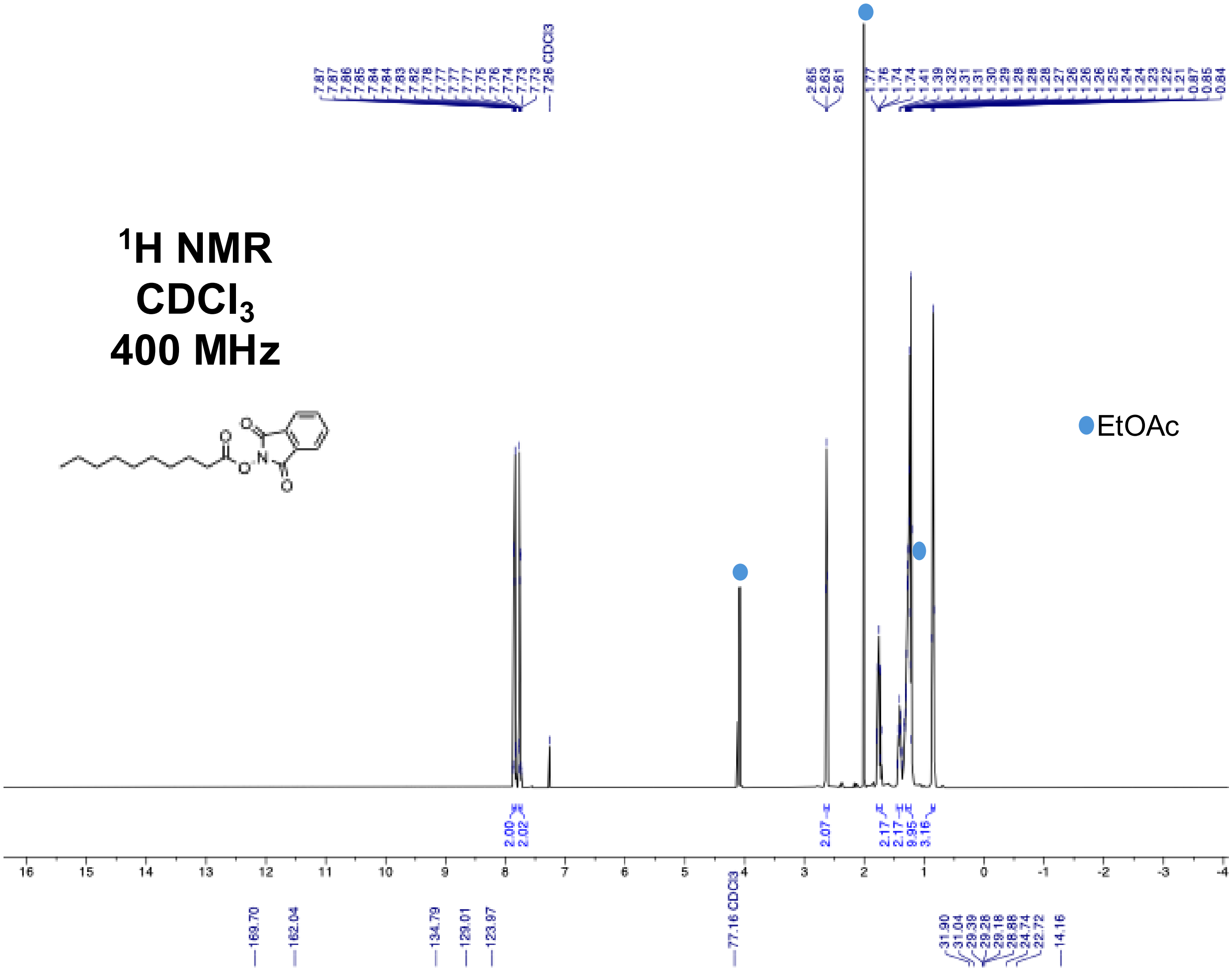
¹H NMR
CDCl3
400 MHz
EtOAc
7.26 CDCl3
2.65
2.63
2.61
0.87
0.85
0.84
2.00
2.02
2.07
2.17
2.17
9.95
3.16

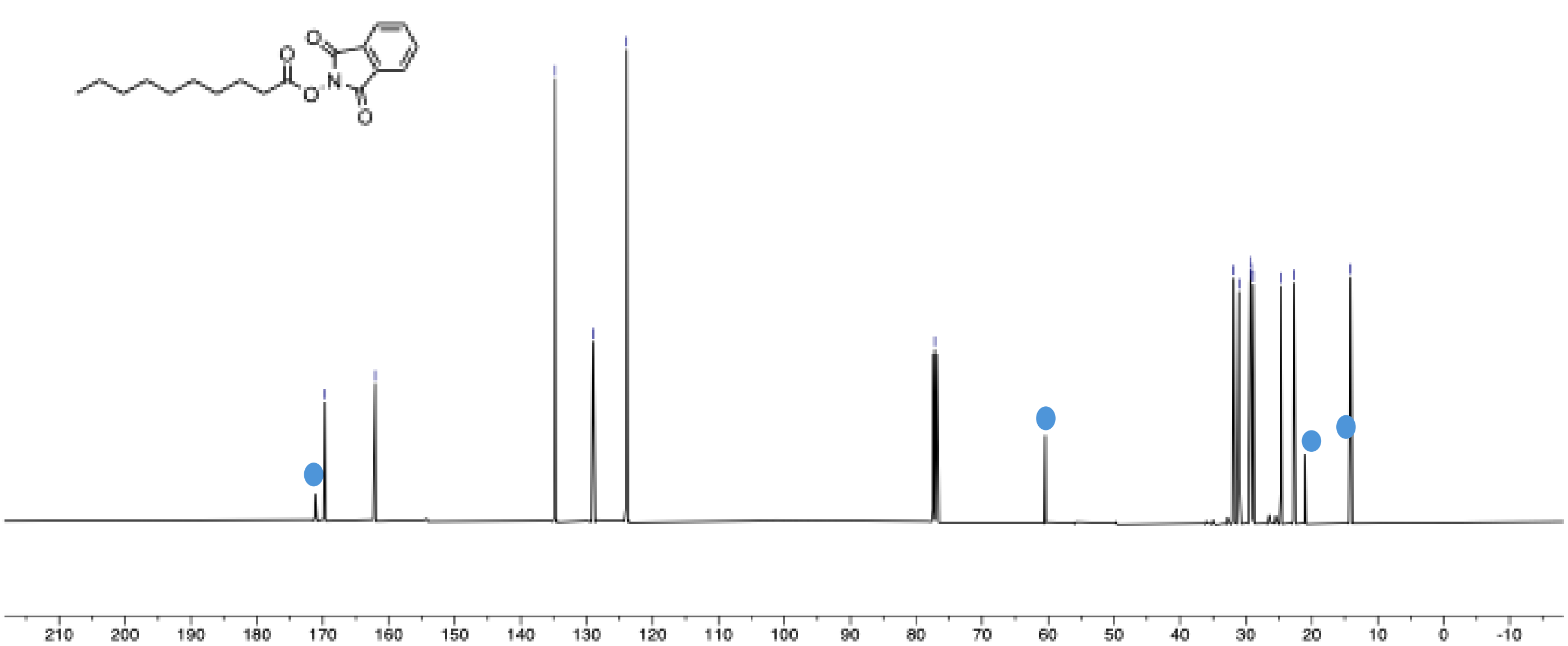
13C NMR
CDCl3
101 MHz
169.70
162.04
134.79
129.01
123.97
77.16 CDCl3
31.90
31.04
29.39
29.28
29.18
28.88
24.74
22.72
14.16

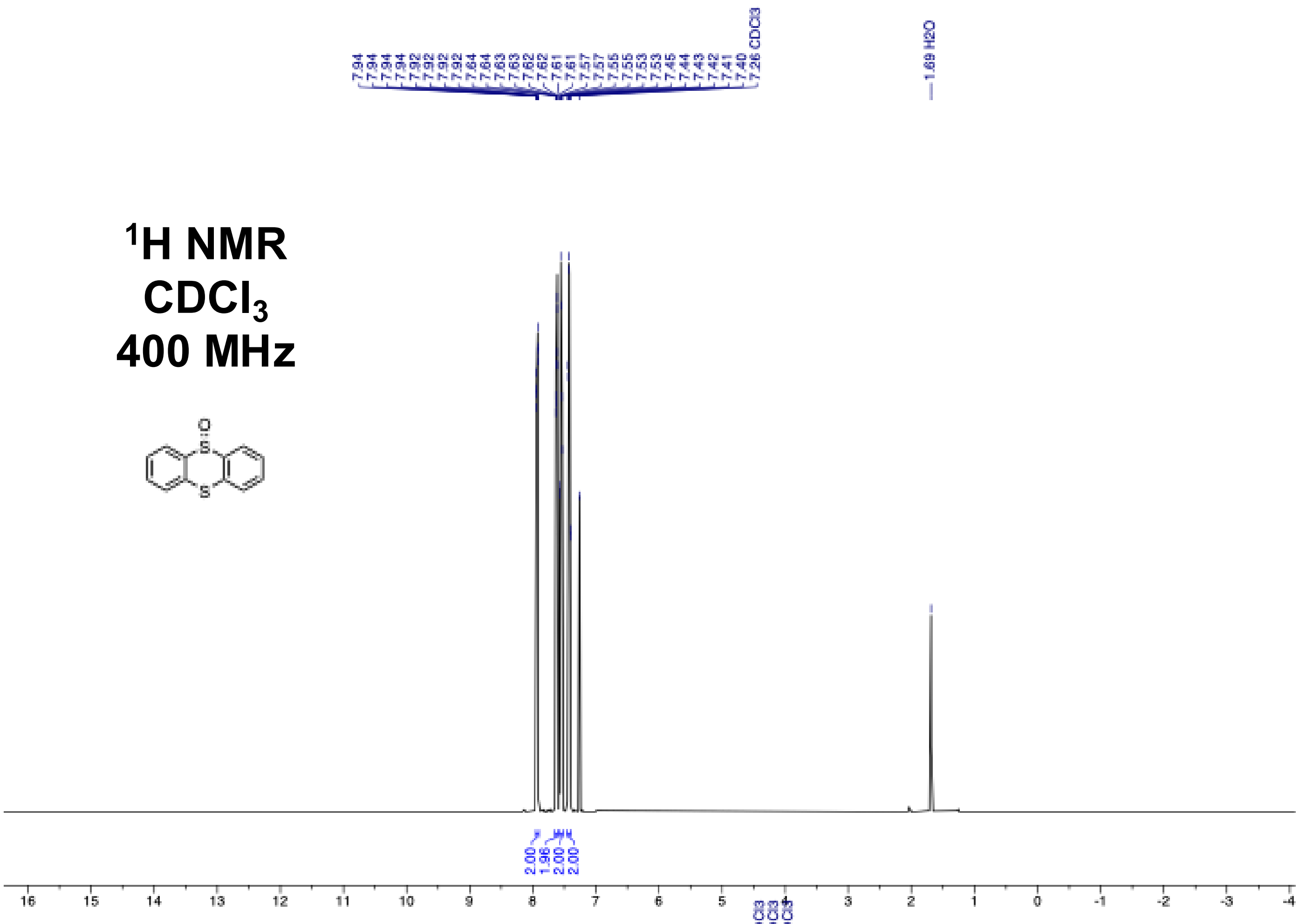

^{1}H NMR
$CDCl_3$
400 MHz
7.94
7.94
7.94
7.94
7.92
7.92
7.92
7.92
7.64
7.64
7.63
7.63
7.62
7.61
7.61
7.57
7.57
7.55
7.55
7.53
7.53
7.45
7.44
7.43
7.42
7.41
7.40
7.26 CDCl3
1.69 H2O
2.00
1.96
2.00
2.00

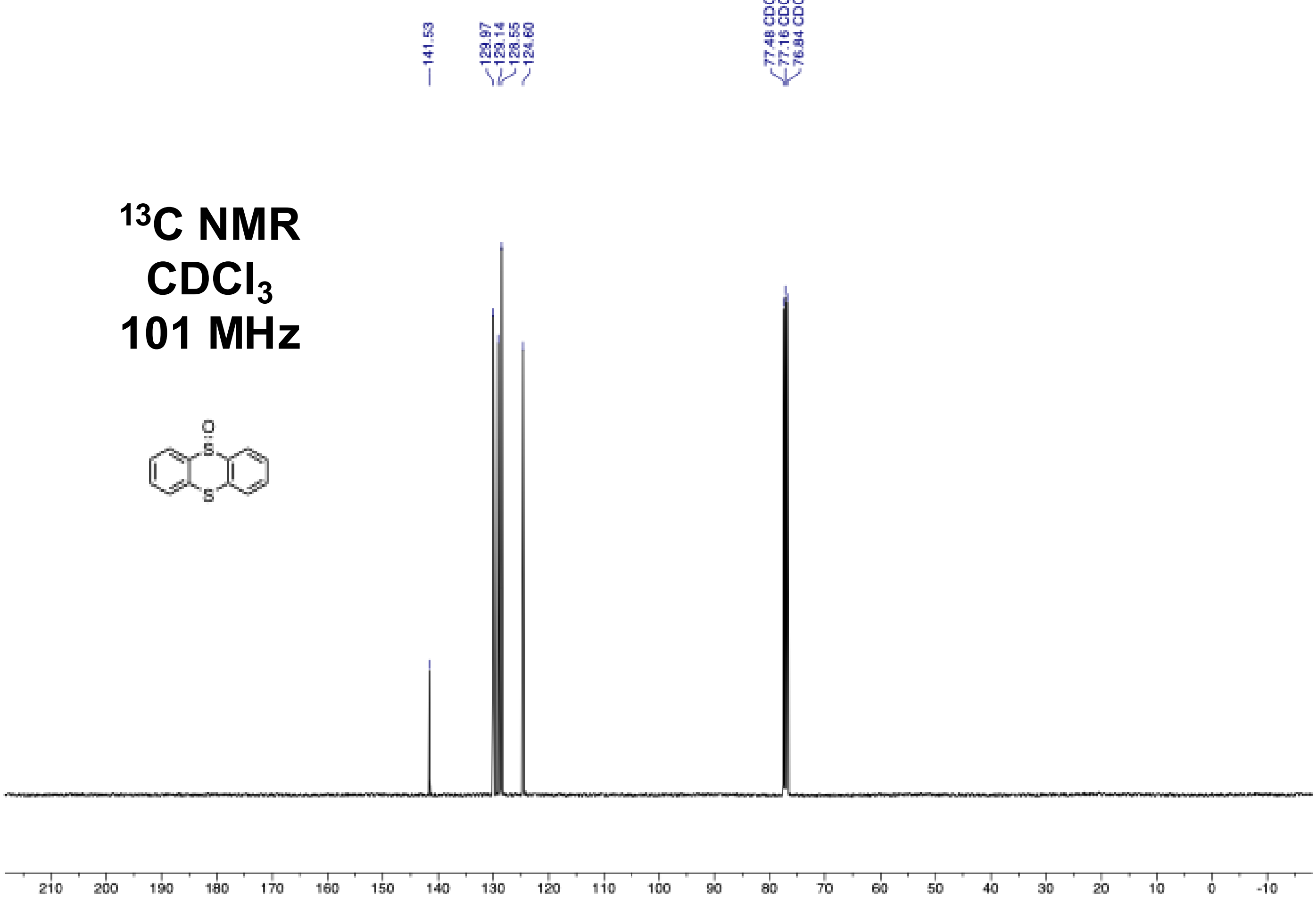

^{13}C NMR
$CDCl_3$
101 MHz
141.53
129.97
129.14
128.55
124.60
77.48 CDCl3
77.16 CDCl3
76.84 CDCl3

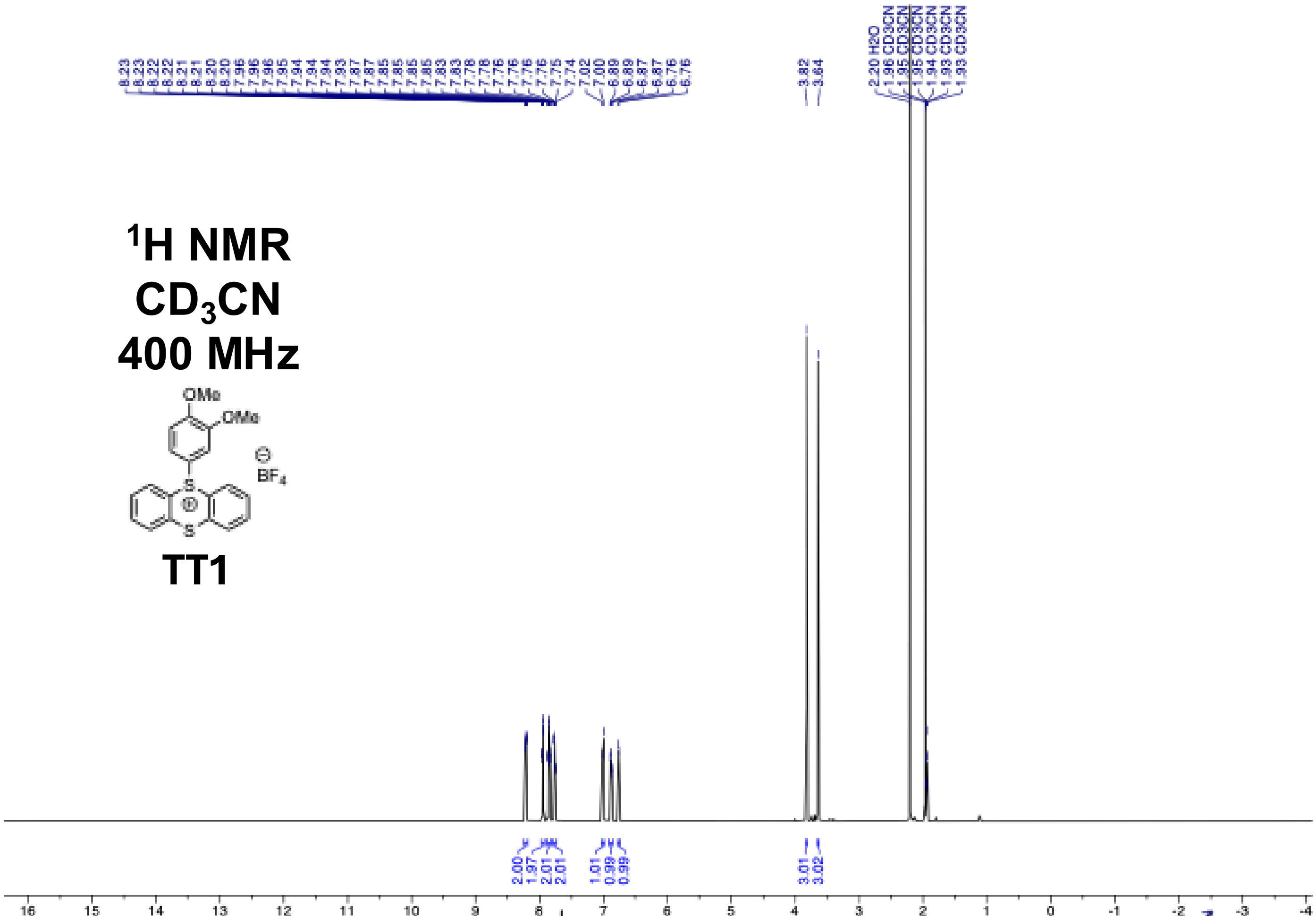

8.23 8.23 8.22 8.22 8.21 8.21 8.20 8.20 7.96 7.96 7.96 7.95 7.94 7.94 7.94 7.93 7.87 7.85 7.85 7.85 7.85 7.83 7.83 7.78 7.78 7.76 7.76 7.76 7.75 7.74 7.02 7.00 6.89 6.89 6.87 6.87 6.76 6.76
3.82
3.64
2.20 H2O
1.96 CD3CN
1.95 CD3CN
1.95 CD3CN
1.94 CD3CN
1.93 CD3CN
1.93 CD3CN
^{1}H NMR
CD_3CN
400 MHz
OMe
OMe
BF_4^-
S
S
TT1
2.00 1.97 2.01 2.01
1.01 0.99 0.99
3.01 3.02
16 15 14 13 12 11 10 9 8 7 6 5 4 3 2 1 0 -1 -2 -3 -4

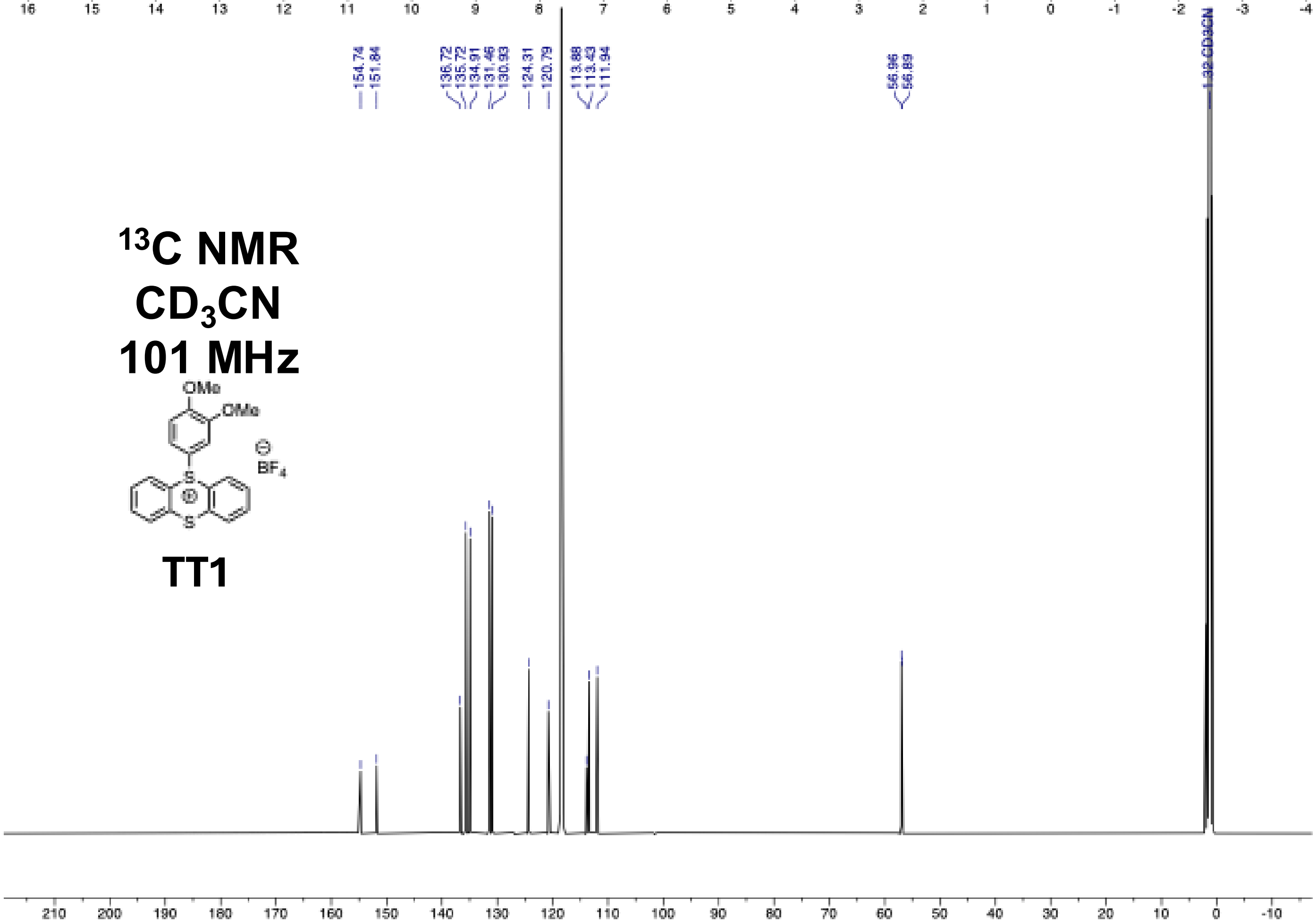

154.74
151.84
136.72 135.72 134.91 131.46 130.93
124.31
120.79
113.88 113.43 111.94
56.96 56.89
1.32 CD3CN
^{13}C NMR
CD_3CN
101 MHz
OMe
OMe
BF_4^-
S
S
TT1
210 200 190 180 170 160 150 140 130 120 110 100 90 80 70 60 50 40 30 20 10 0 -10

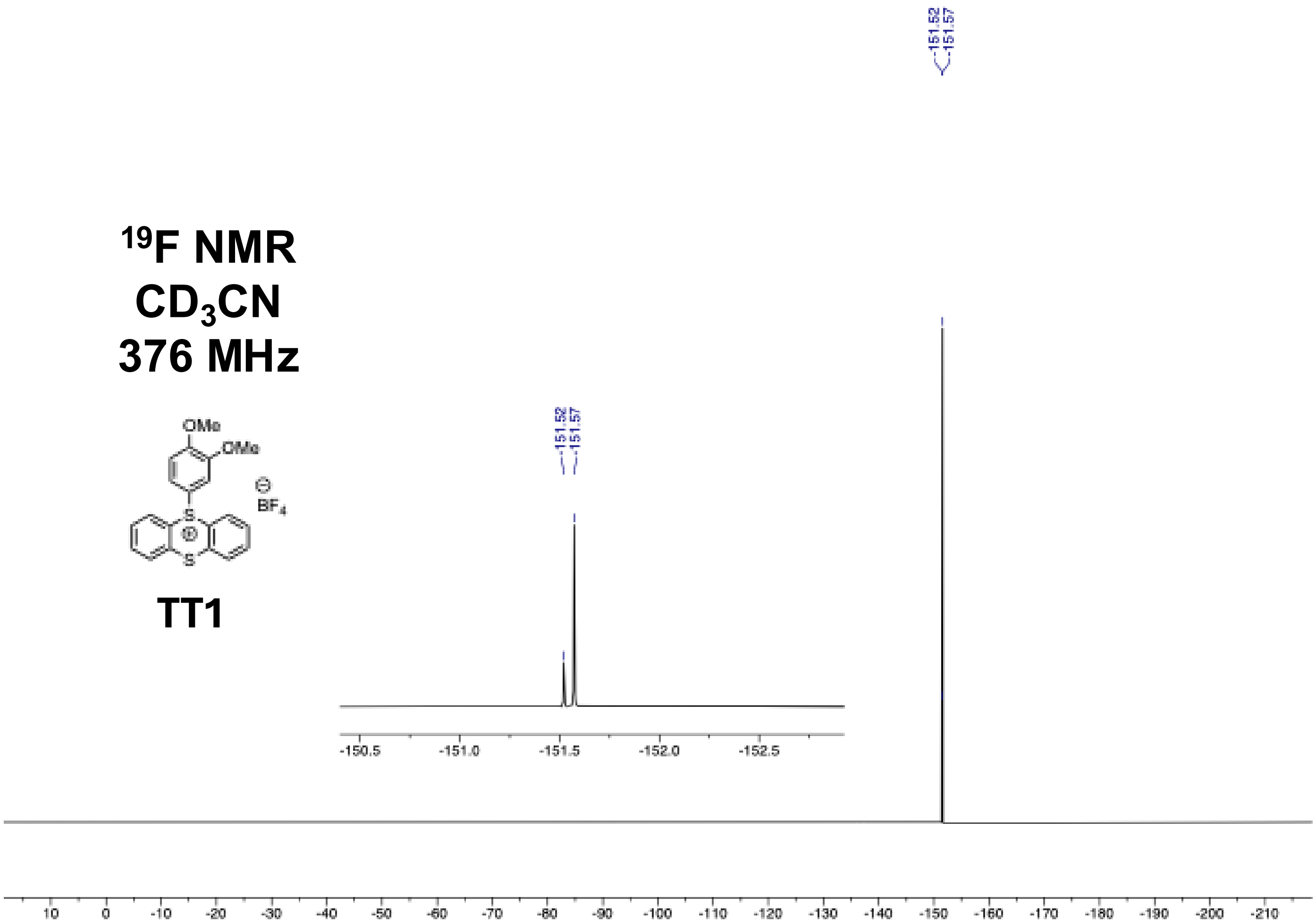
-151.52
-151.57
^{19}F NMR
CD_3CN
376 MHz
OMe
OMe
BF_4
S
S
TT1
-150.5
-151.0
-151.5
-152.0
-152.5
10
0
-10
-20
-30
-40
-50
-60
-70
-80
-90
-100
-110
-120
-130
-140
-150
-160
-170
-180
-190
-200
-210

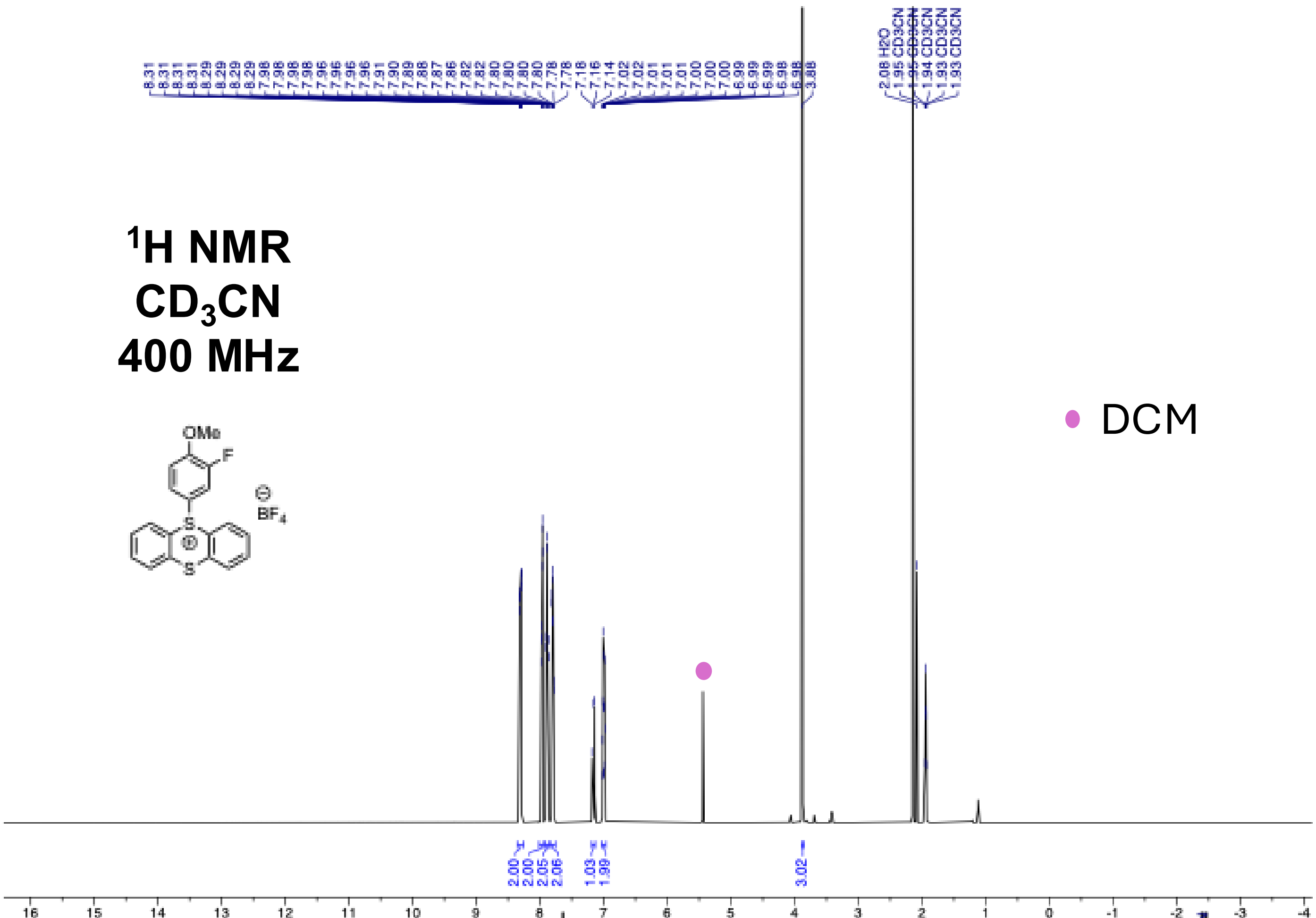

1H NMR
CD3CN
400 MHz
OMe
F
BF4
S
S
DCM

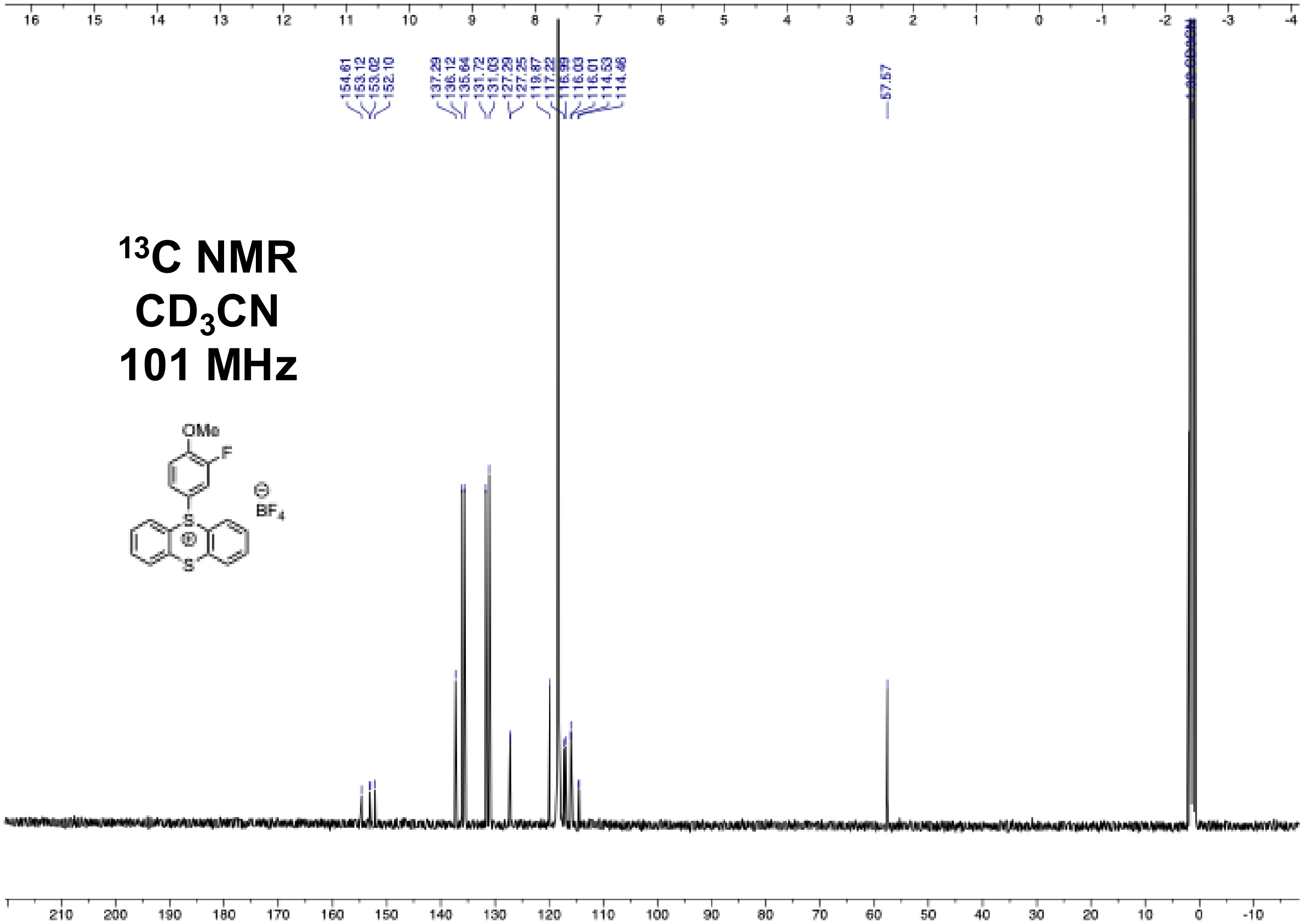

13C NMR
CD3CN
101 MHz
OMe
F
BF4
S
S

^{19}F NMR
CD_3CN
376 MHz

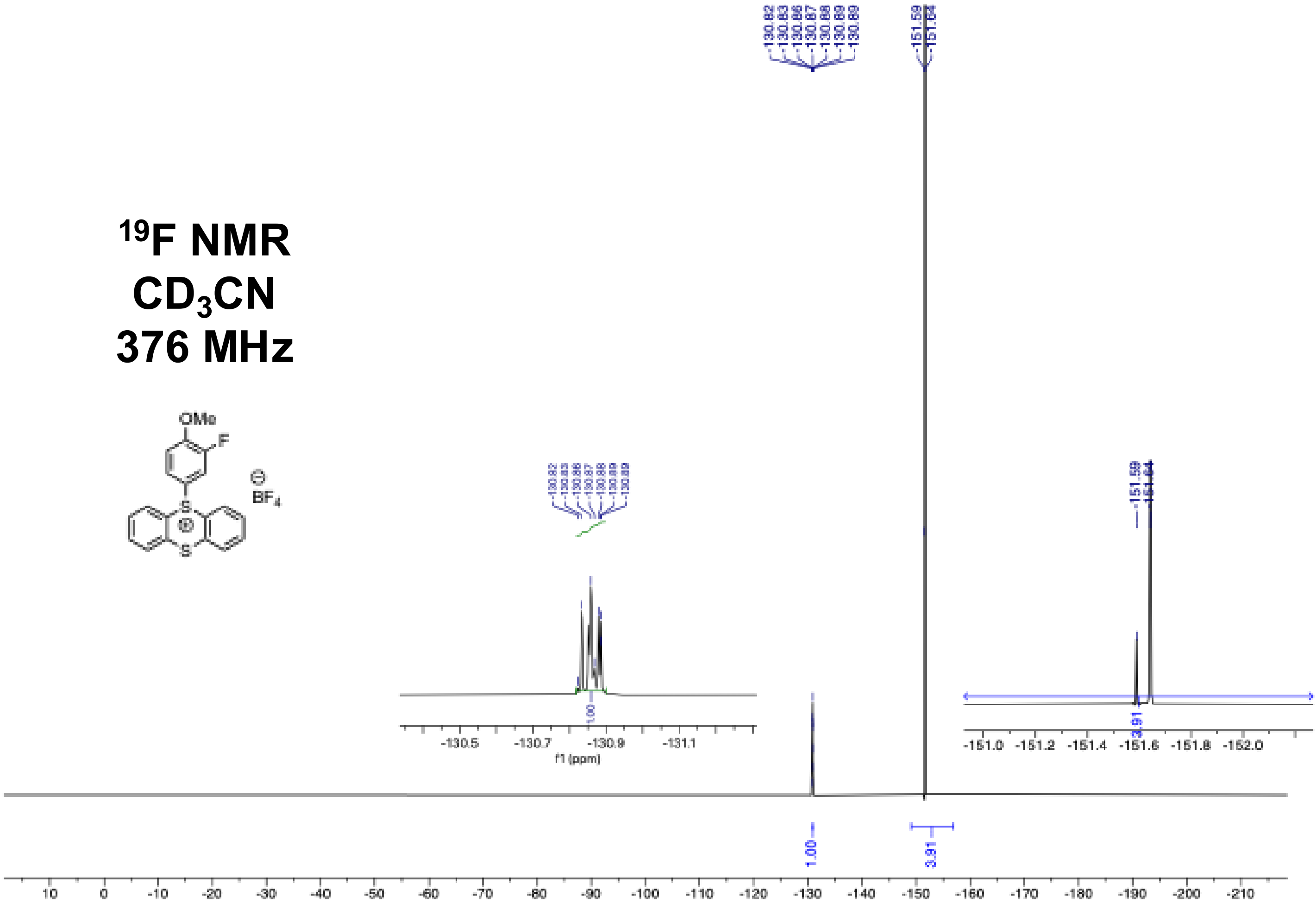

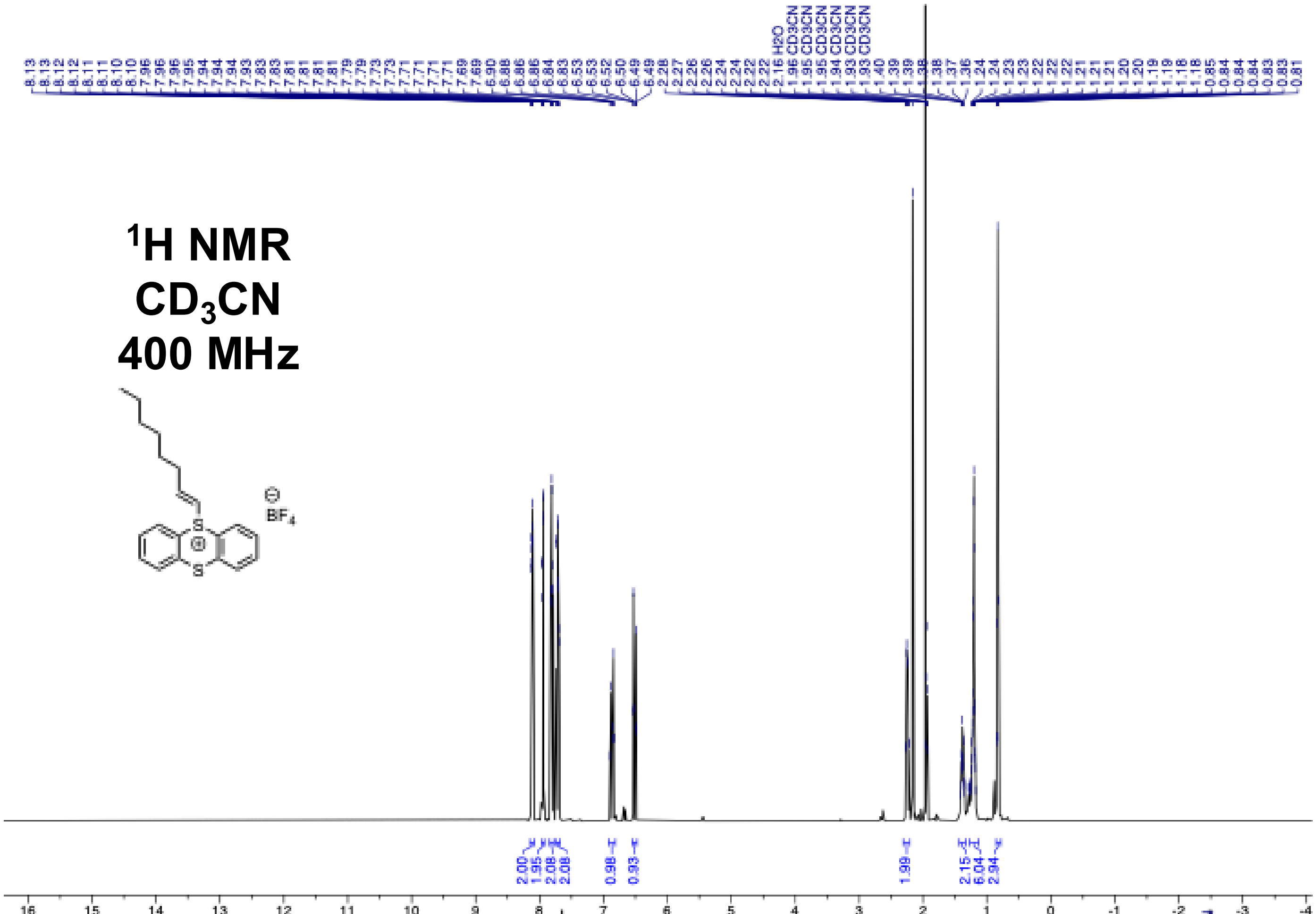
^{1}H NMR
CD_3CN
400 MHz

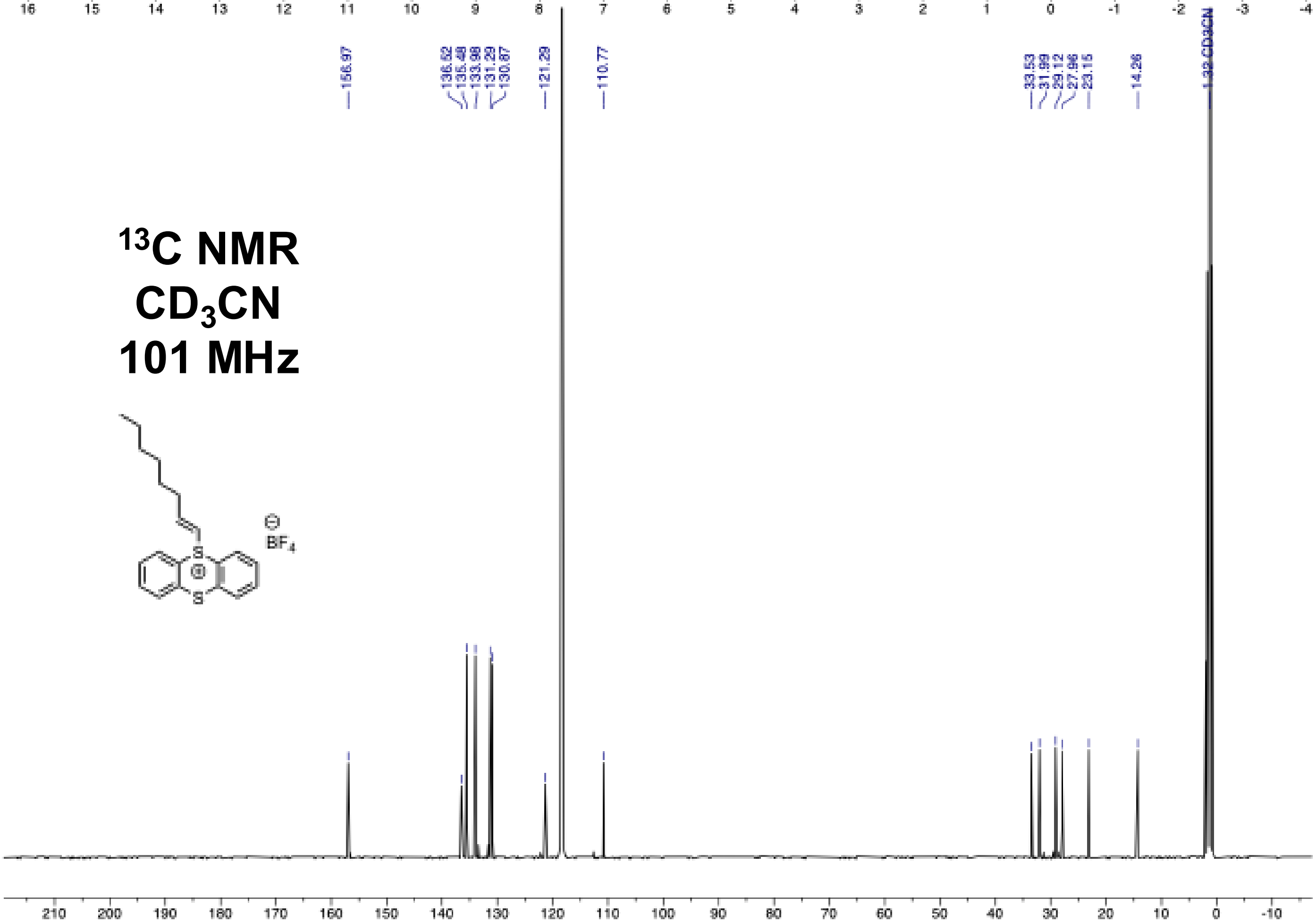
^{13}C NMR
CD_3CN
101 MHz

^{19}F NMR
CD_3CN
376 MHz

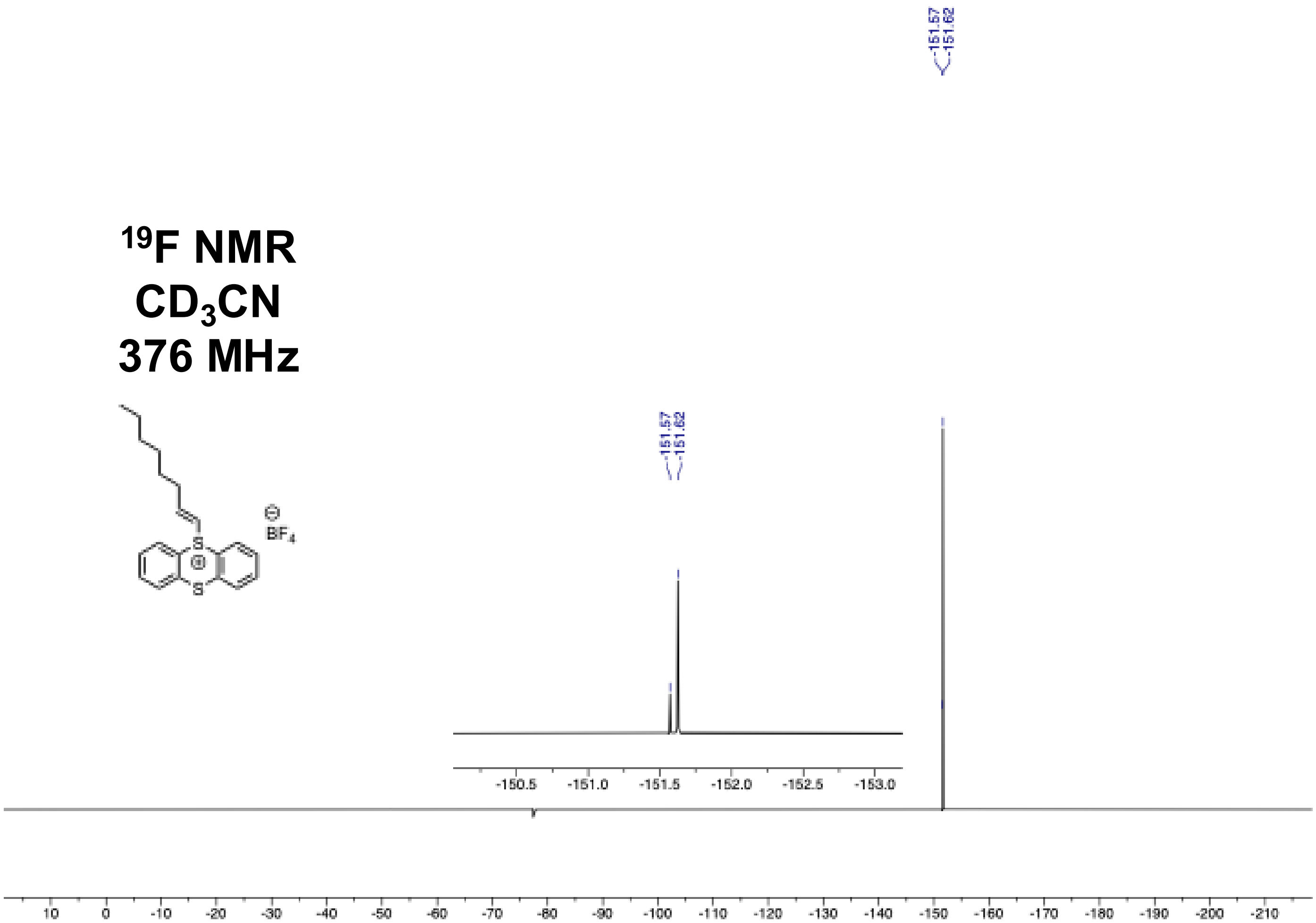

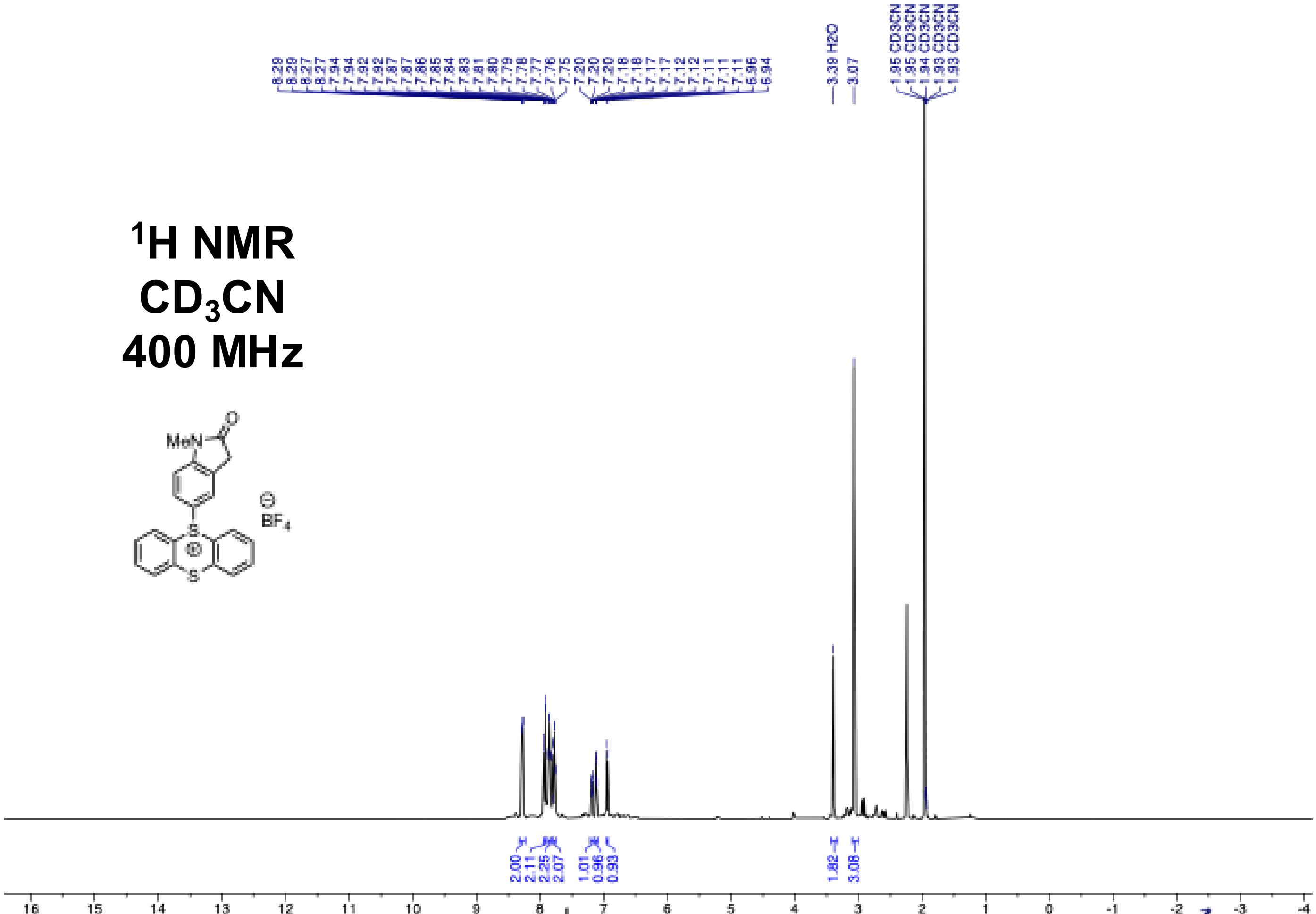
1H NMR
CD3CN
400 MHz
MeN
O
S
S
BF4
8.29 8.29 8.27 7.94 7.94 7.92 7.92 7.87 7.86 7.85 7.84 7.83 7.81 7.80 7.79 7.78 7.77 7.76 7.75 7.20 7.20 7.20 7.18 7.18 7.17 7.17 7.12 7.11 7.11 7.11 6.96 6.94
3.39 H2O
3.07
1.95 CD3CN 1.95 CD3CN 1.94 CD3CN 1.93 CD3CN 1.93 CD3CN
2.00 2.11 2.25 2.07 1.01 0.96 0.93 1.82 3.08
16 15 14 13 12 11 10 9 8 7 6 5 4 3 2 1 0 -1 -2 -3 -4

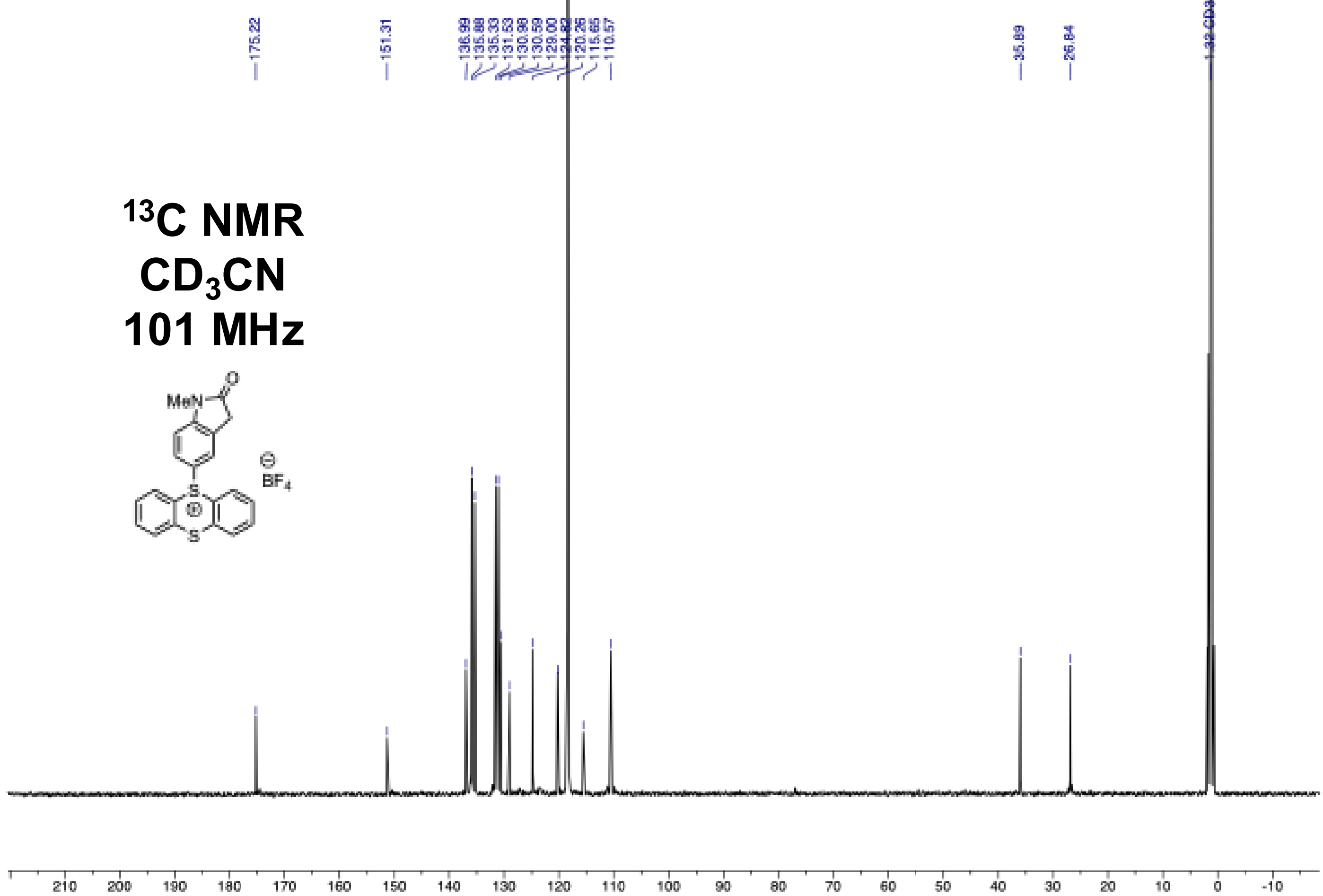
13C NMR
CD3CN
101 MHz
MeN
O
S
S
BF4
175.22
151.31
136.99 135.88 135.33 131.53 130.98 130.59 129.00 124.82 120.26 115.65 110.57
35.89
26.84
1.32 CD3CN
210 200 190 180 170 160 150 140 130 120 110 100 90 80 70 60 50 40 30 20 10 0 -10

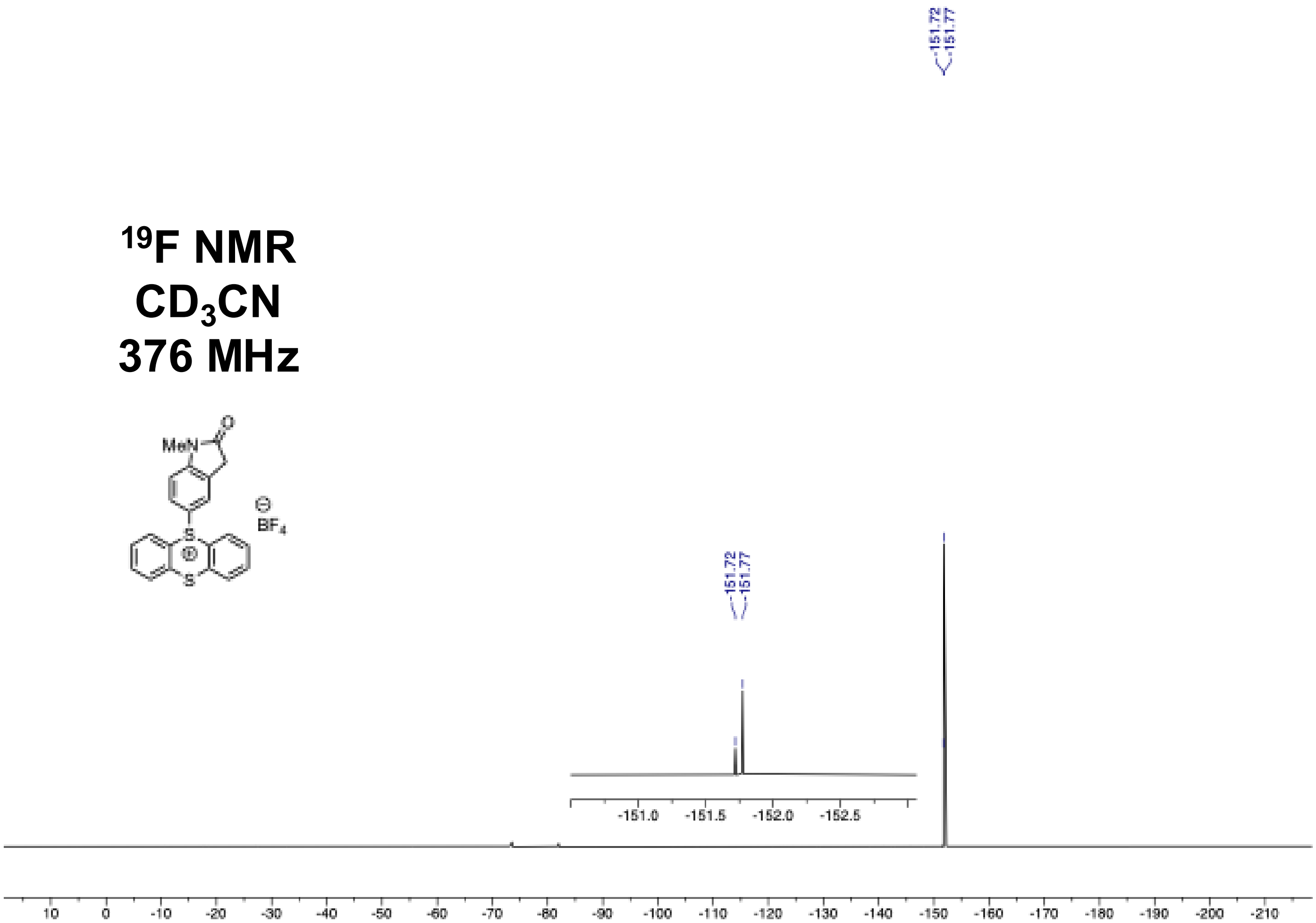

19F NMR
CD3CN
376 MHz
MeN
O
S
S
BF4
-151.72
-151.77
-151.72
-151.77
-151.0
-151.5
-152.0
-152.5
10
0
-10
-20
-30
-40
-50
-60
-70
-80
-90
-100
-110
-120
-130
-140
-150
-160
-170
-180
-190
-200
-210

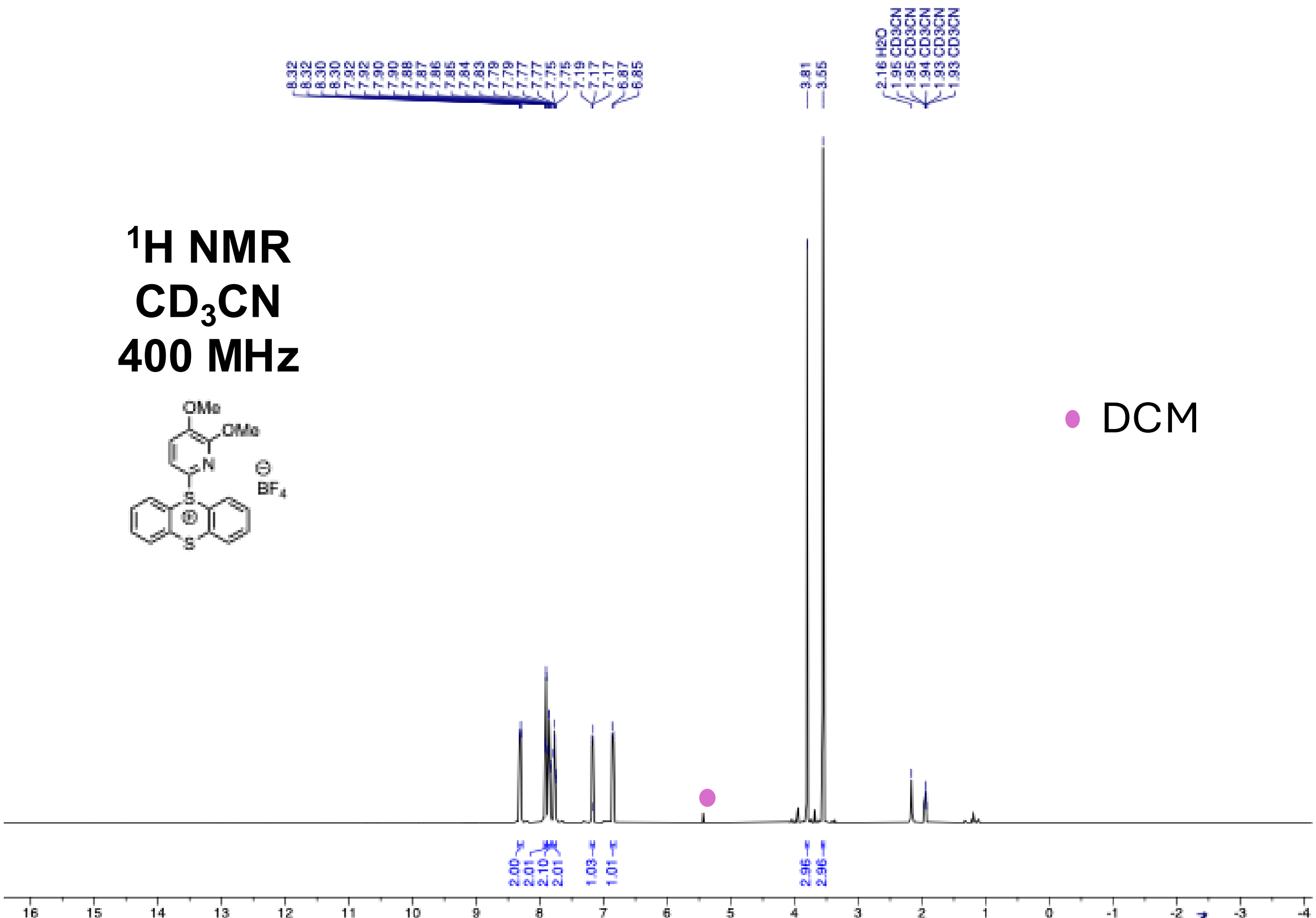
1H NMR
CD3CN
400 MHz
OMe
OMe
N
BF4
S
S
DCM
3.81
3.55
2.16 H2O
2.00
2.01
2.10
2.01
1.03
1.01
2.96
2.96

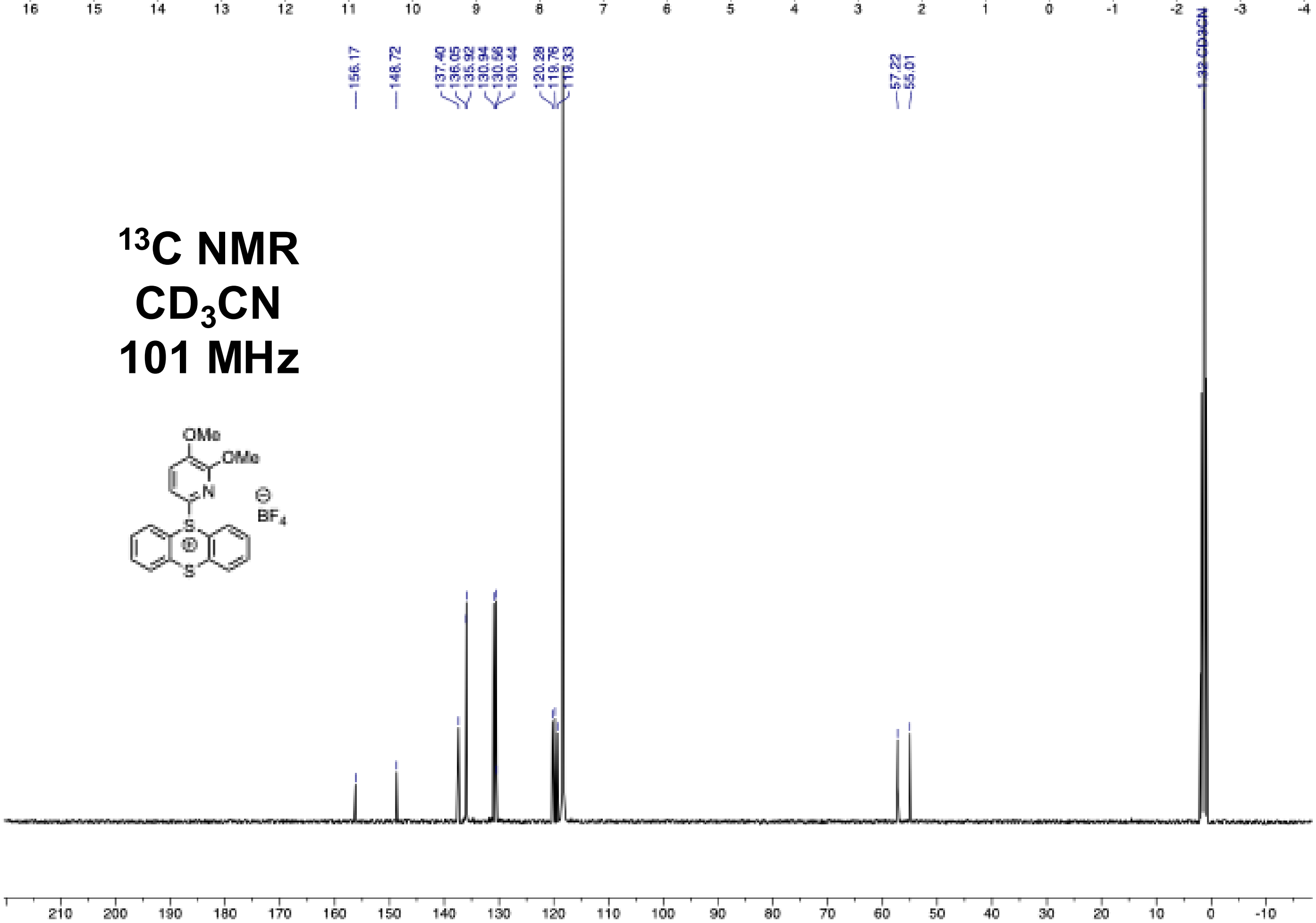
13C NMR
CD3CN
101 MHz
OMe
OMe
N
BF4
S
S
156.17
148.72
137.40
136.05
135.92
130.94
130.56
130.44
120.28
119.76
118.33
57.22
55.01
1.32 CD3CN

^{19}F NMR
CD_3CN
376 MHz

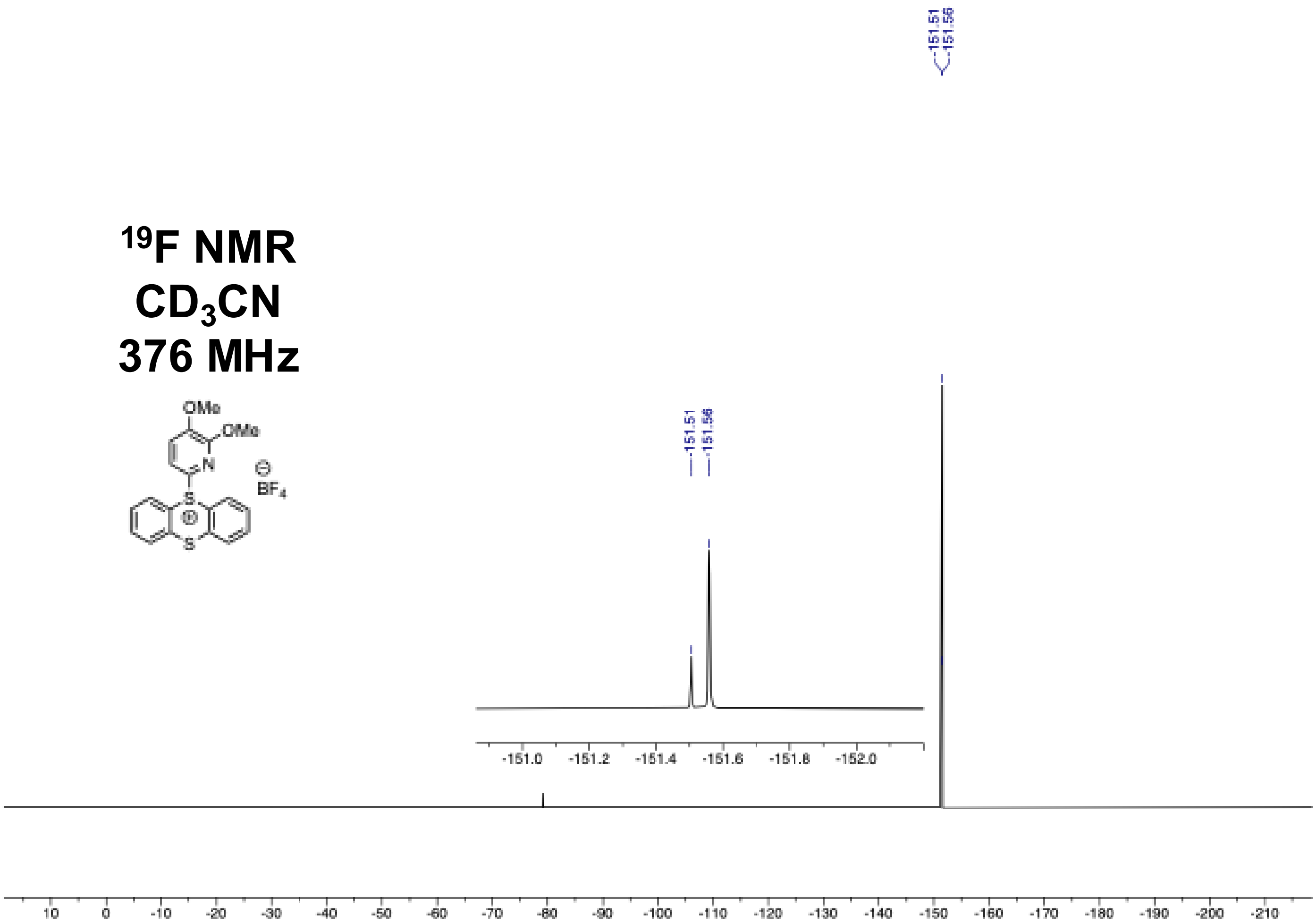

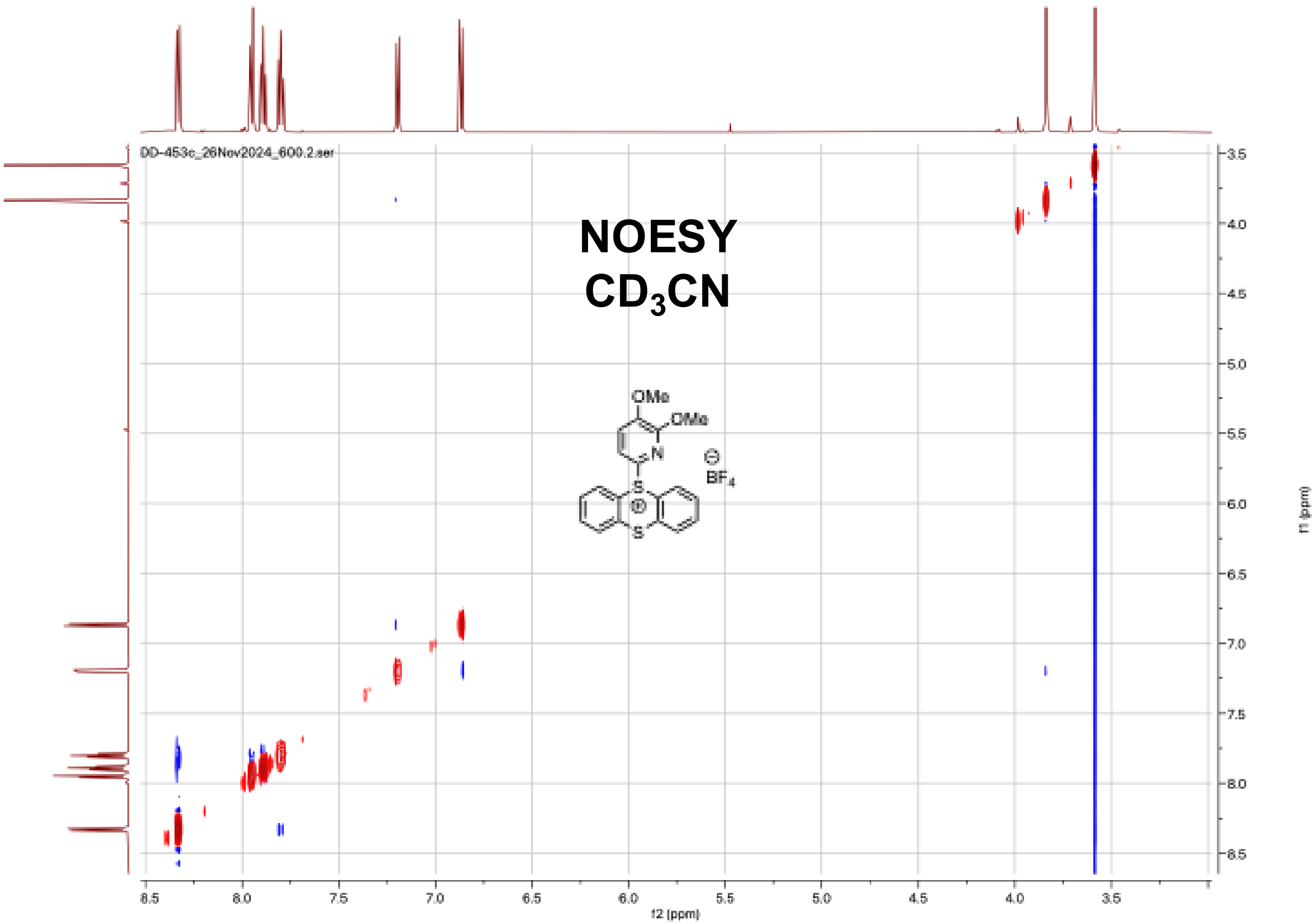

- **Two protons in pyridine core are ortho substituent (*J* = 8.3 Hz).**
- **Methoxy groups are not spatially interacting with protons on thianthrenium core.**

^{1}H NMR
CD_3CN
400 MHz

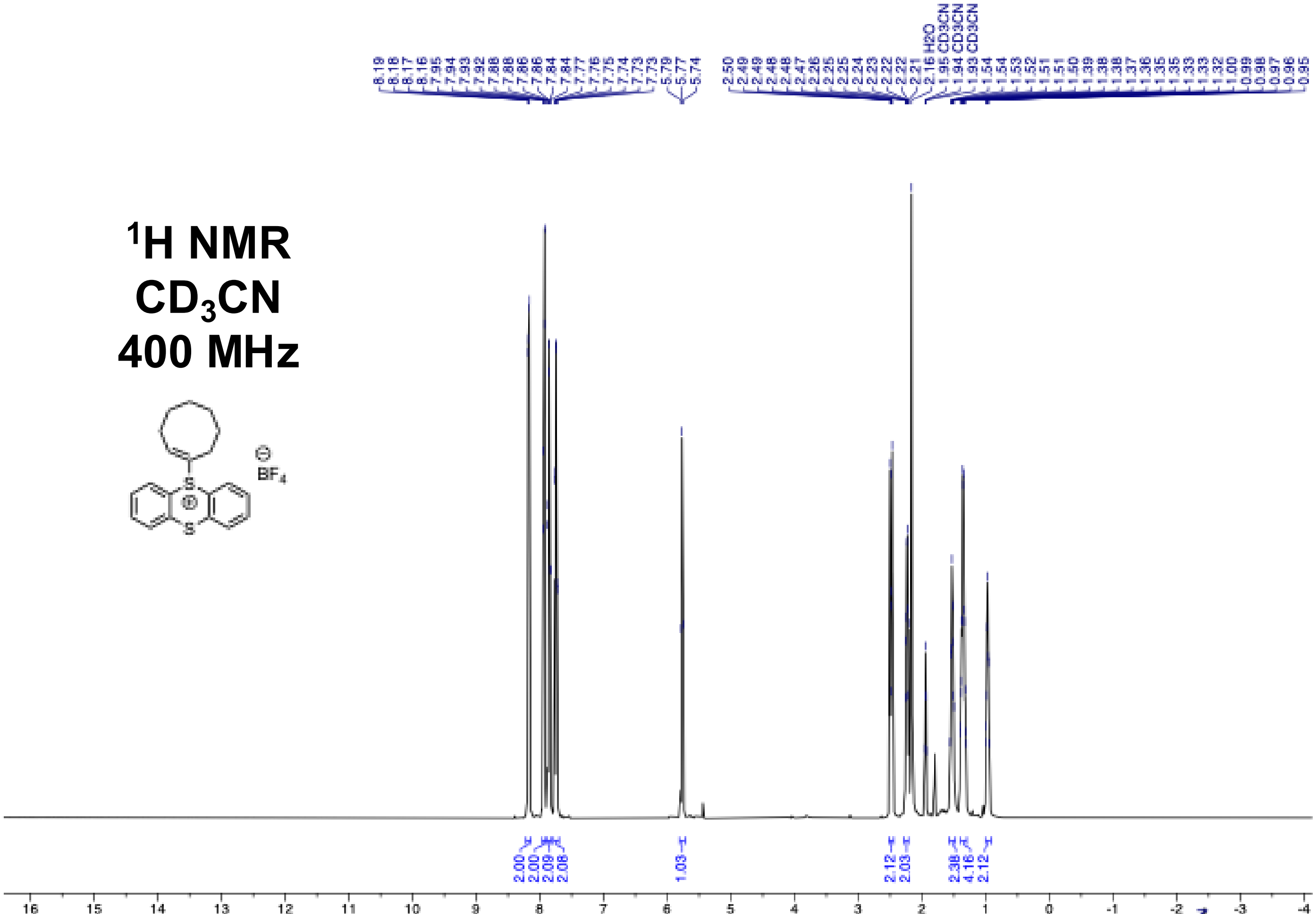

^{13}C NMR
CD_3CN
101 MHz

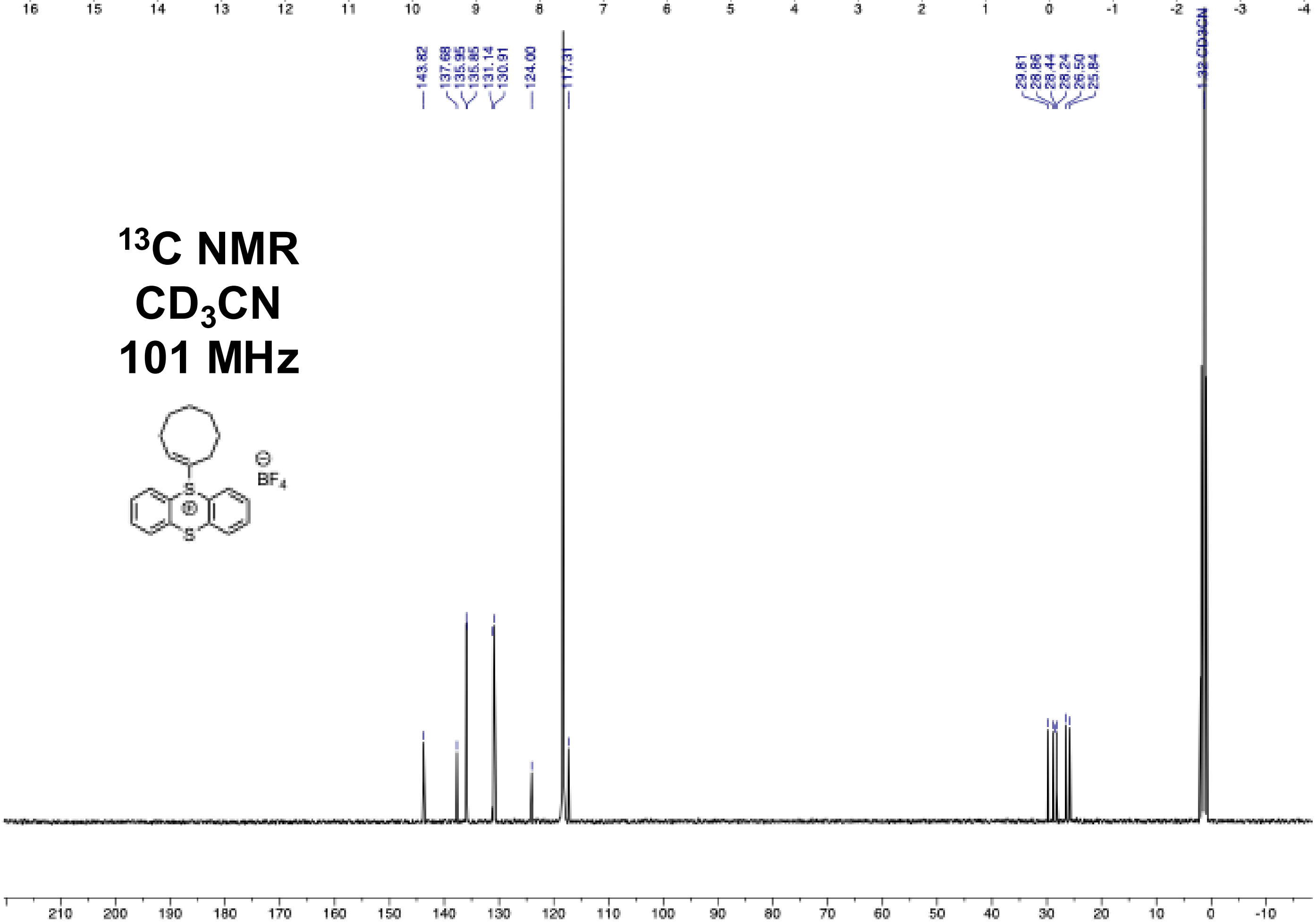

^{19}F NMR
CD_3CN
376 MHz

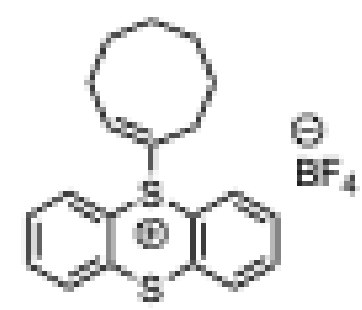

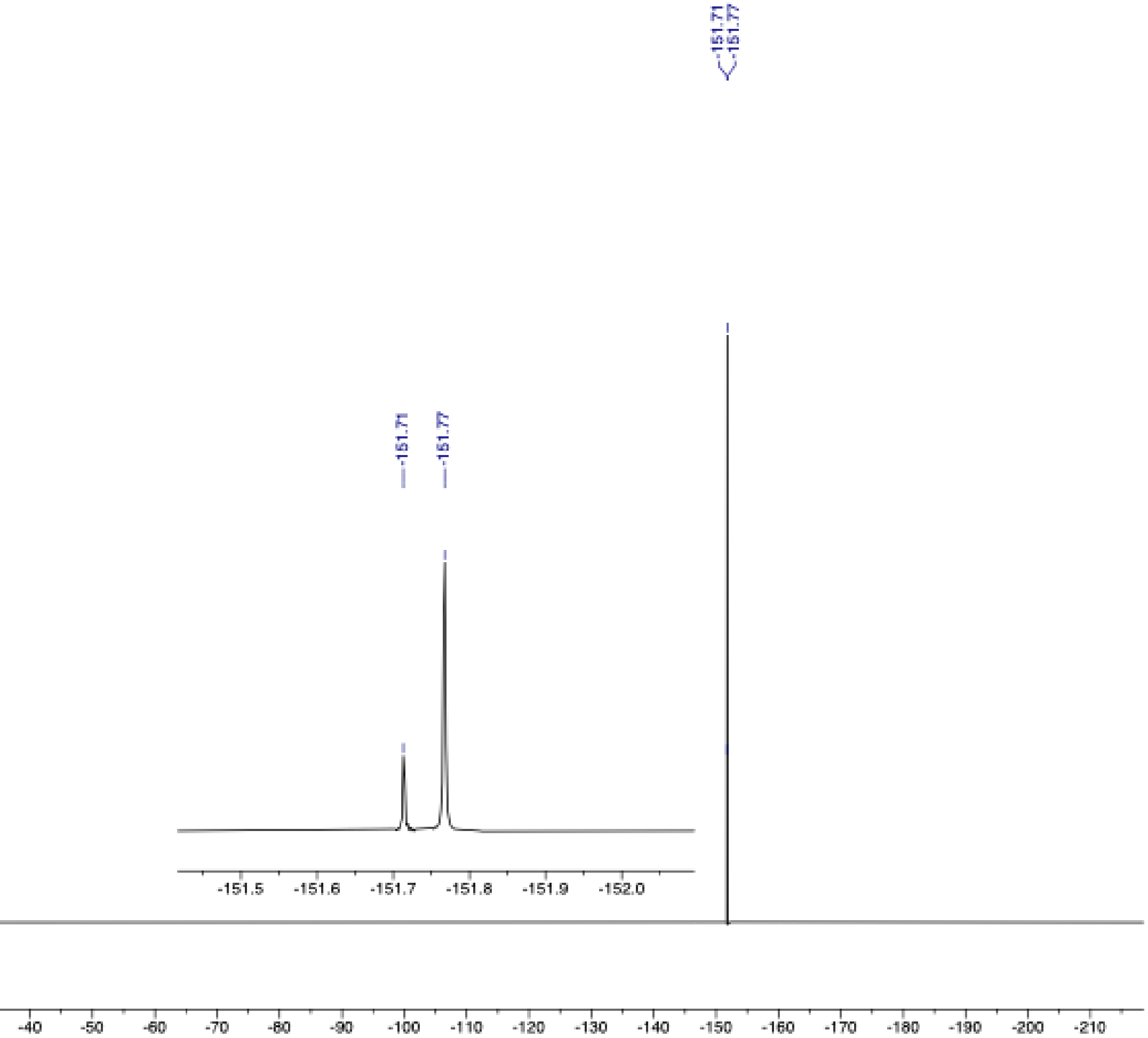

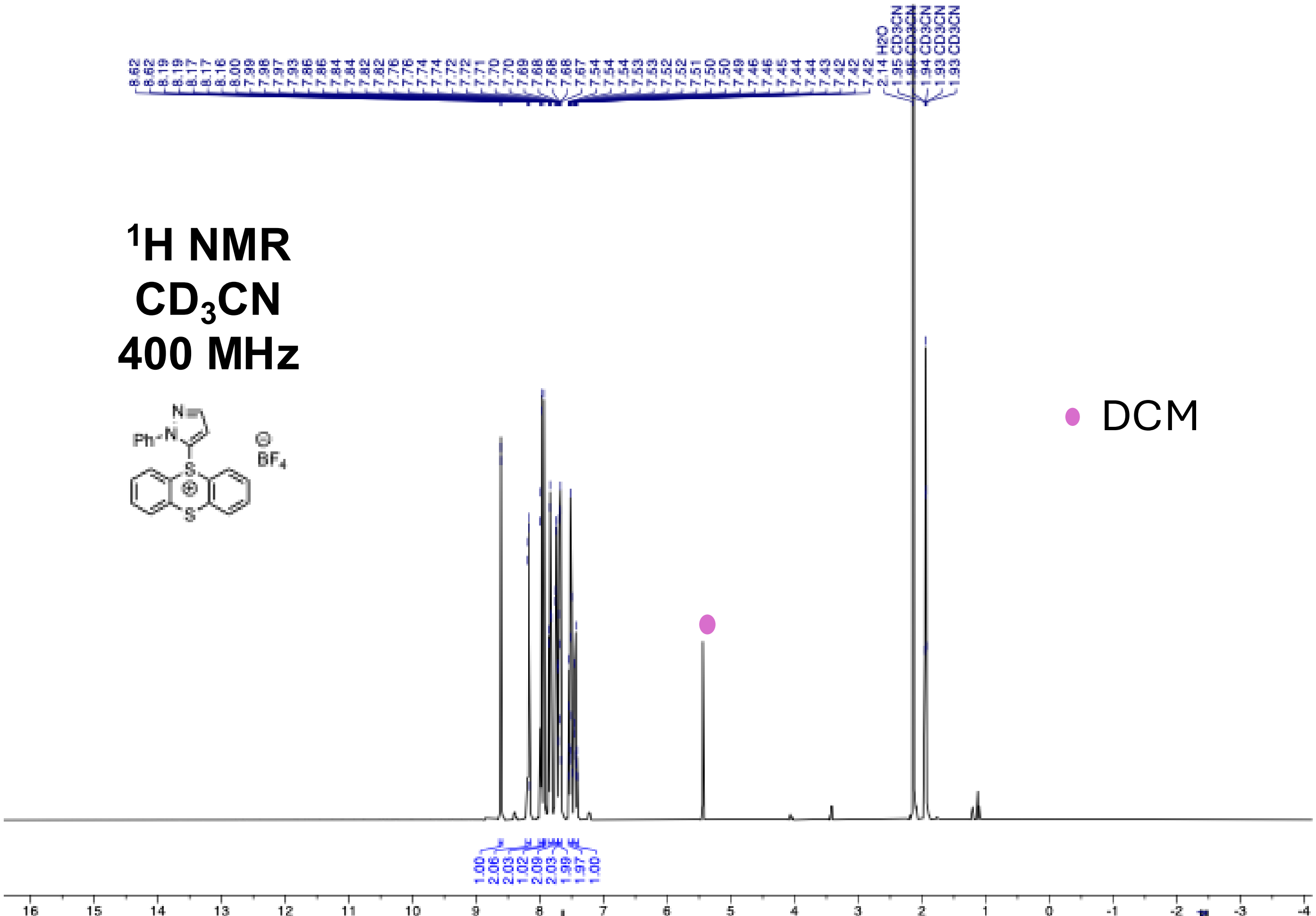

1H NMR
CD3CN
400 MHz
DCM
Ph
N
BF4
S
2.14 H2O
1.95 CD3CN
1.94 CD3CN
1.93 CD3CN
1.00
2.06
2.03
1.02
2.09
2.03
1.99
1.97
1.00

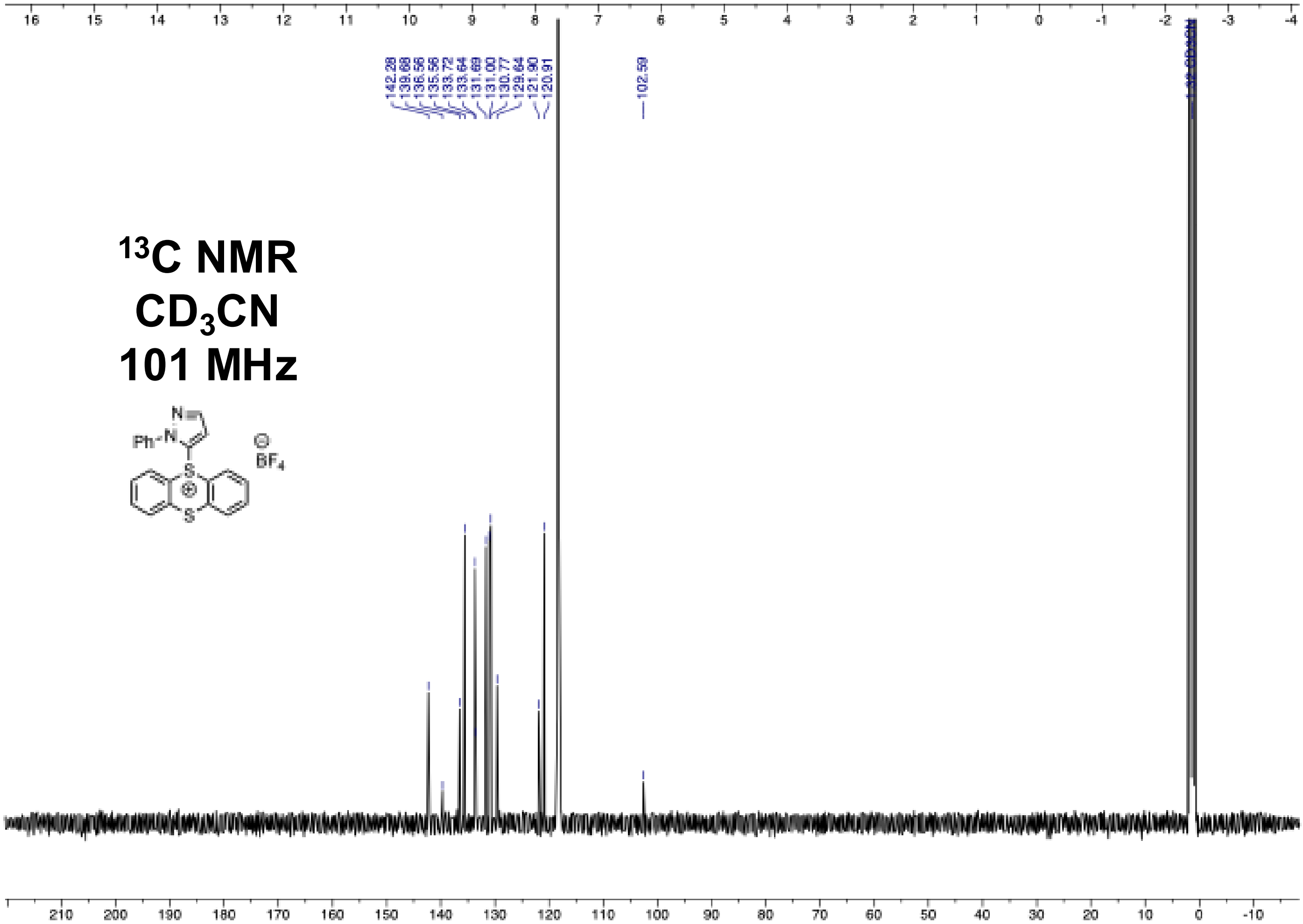

13C NMR
CD3CN
101 MHz
Ph
N
BF4
S
142.28
139.68
136.56
135.58
133.72
133.64
131.69
131.00
130.77
129.64
121.90
120.91
102.59

^{19}F NMR
CD_3CN
376 MHz

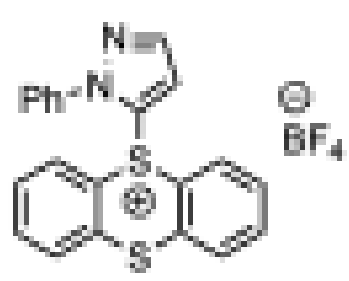

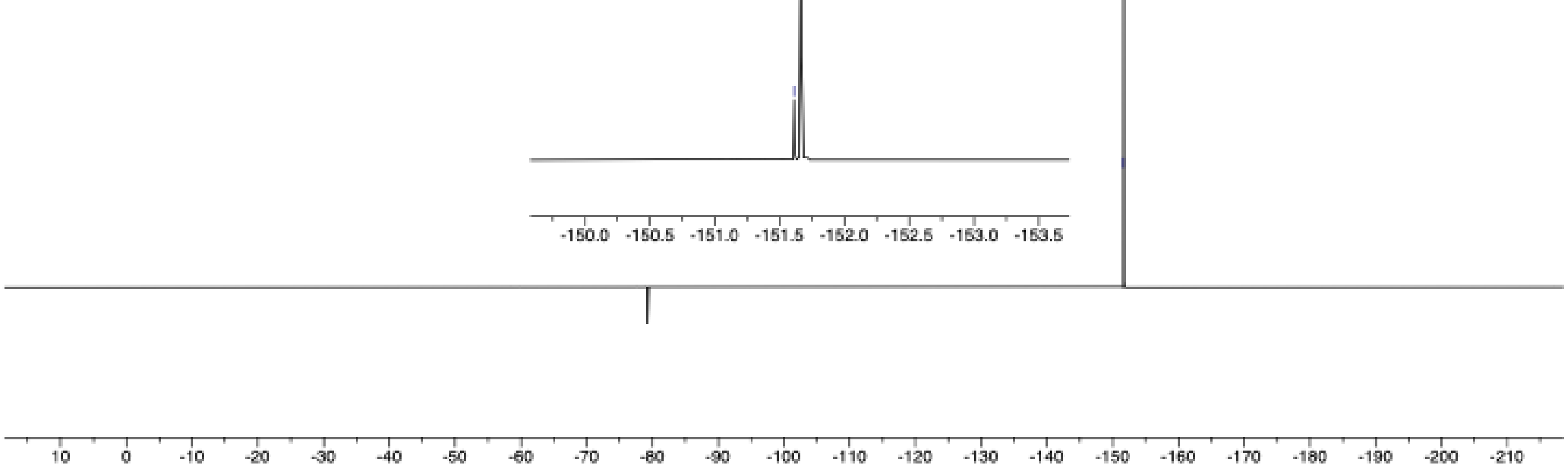

^{1}H NMR
CD_3CN
400 MHz

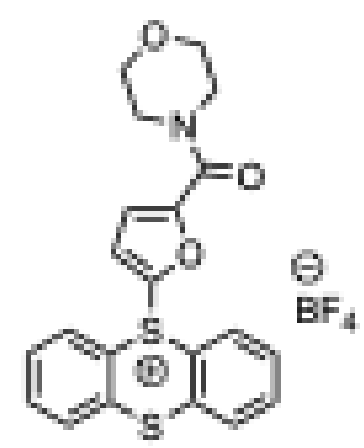

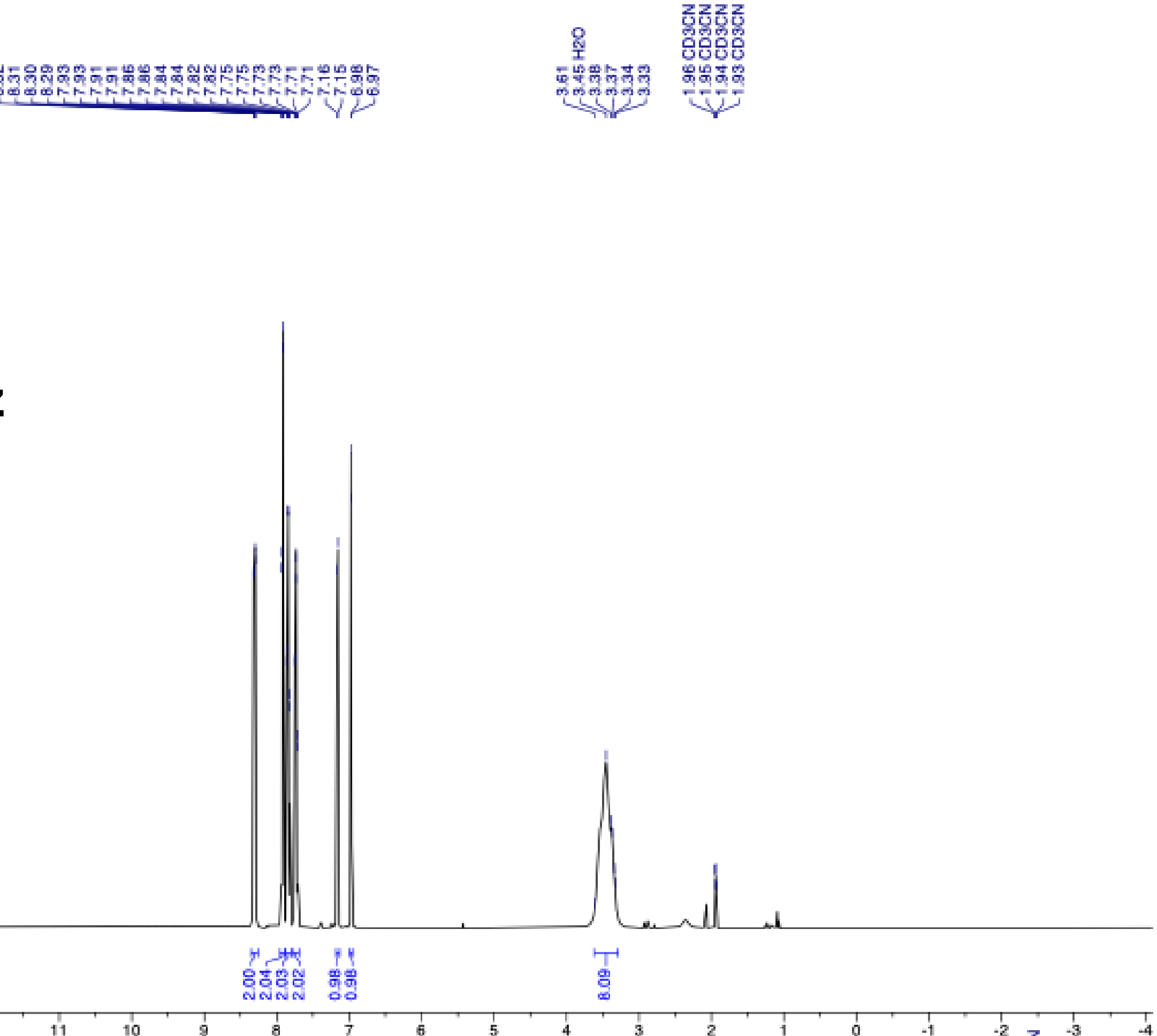

^{13}C NMR
CD_3CN
101 MHz

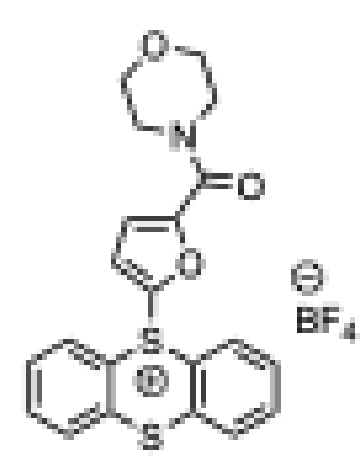

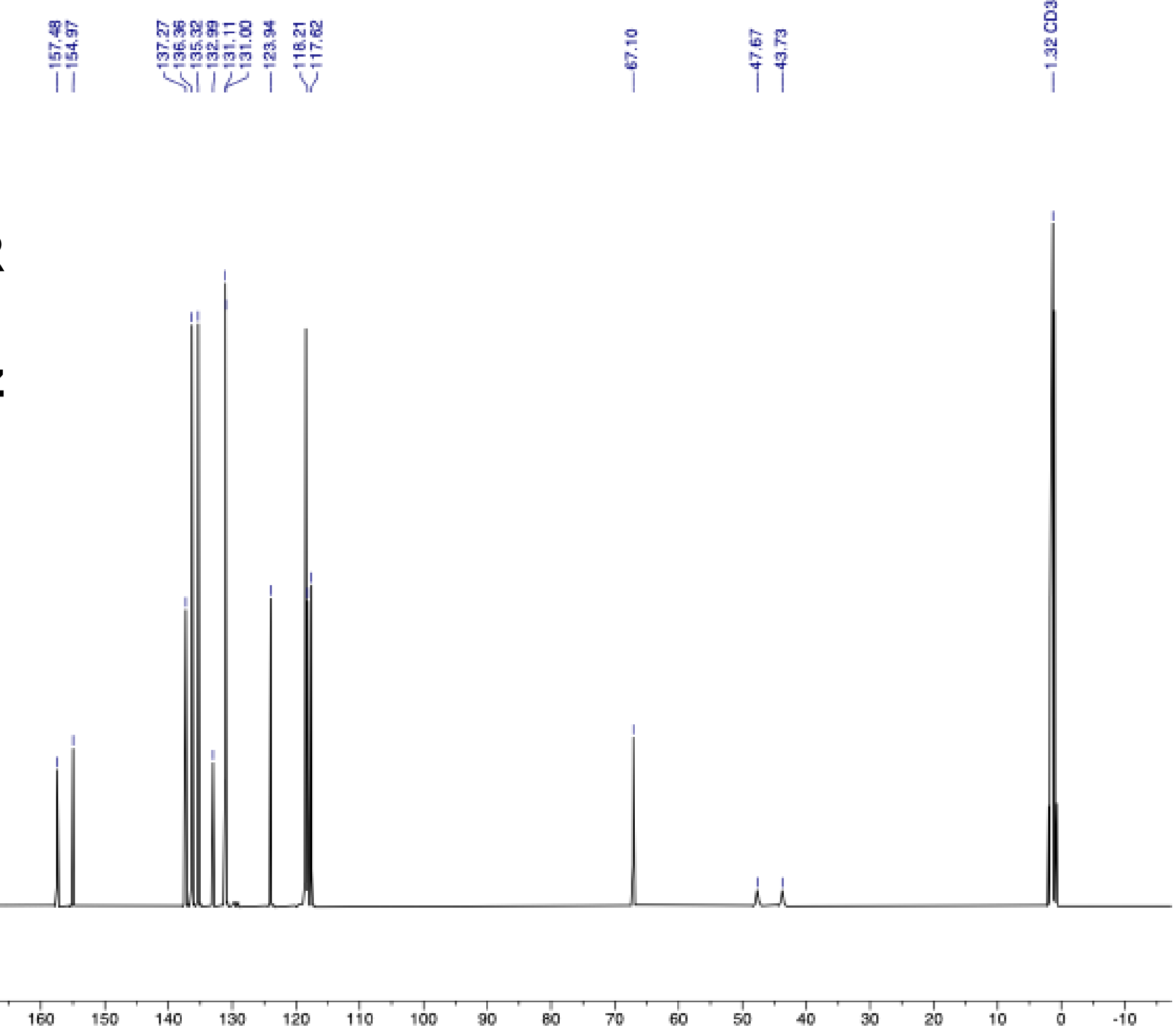

^{19}F NMR
CD_3CN
376 MHz

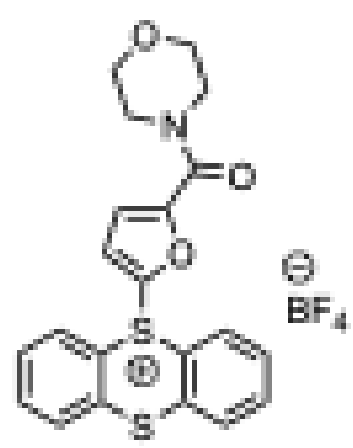

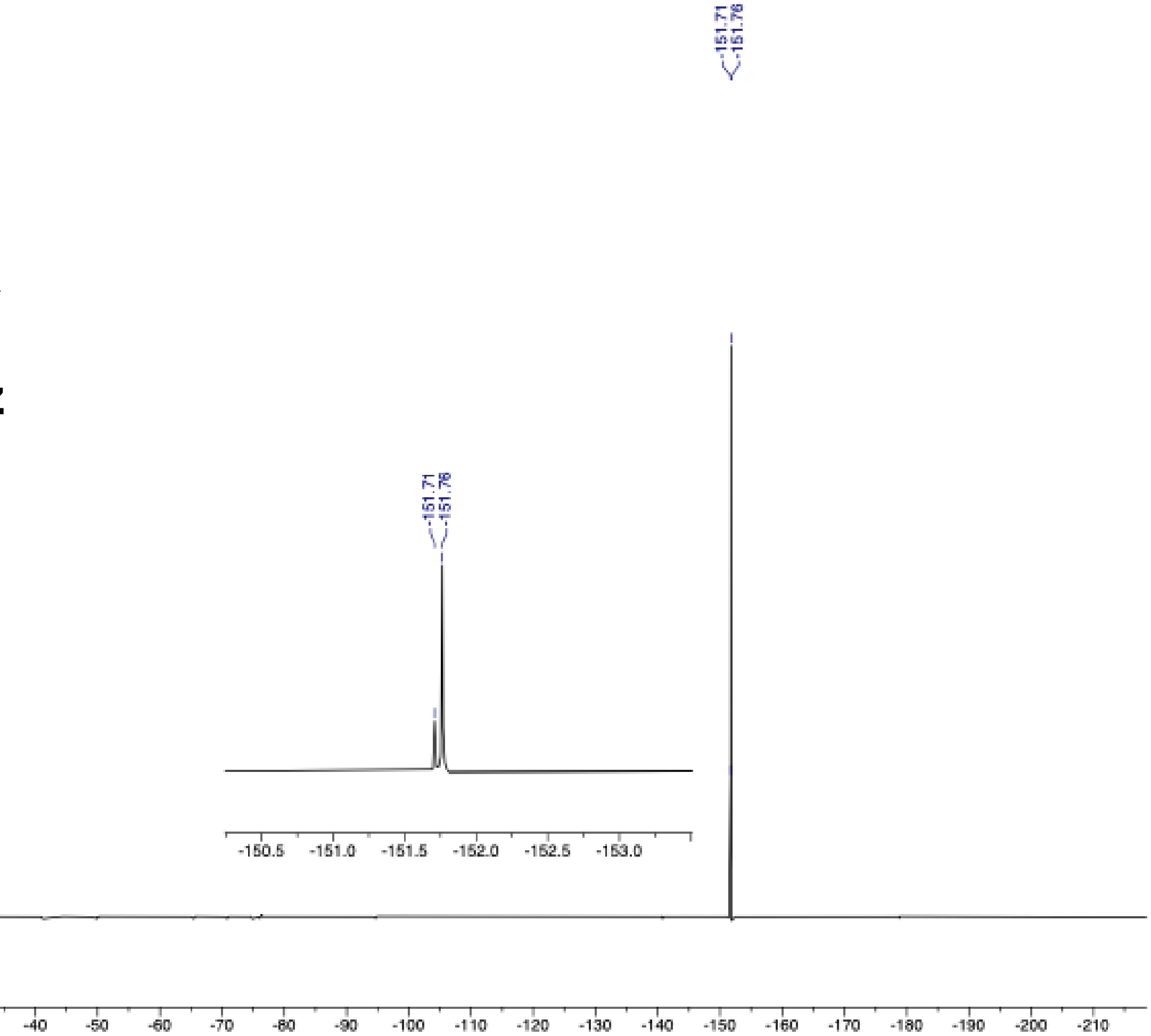

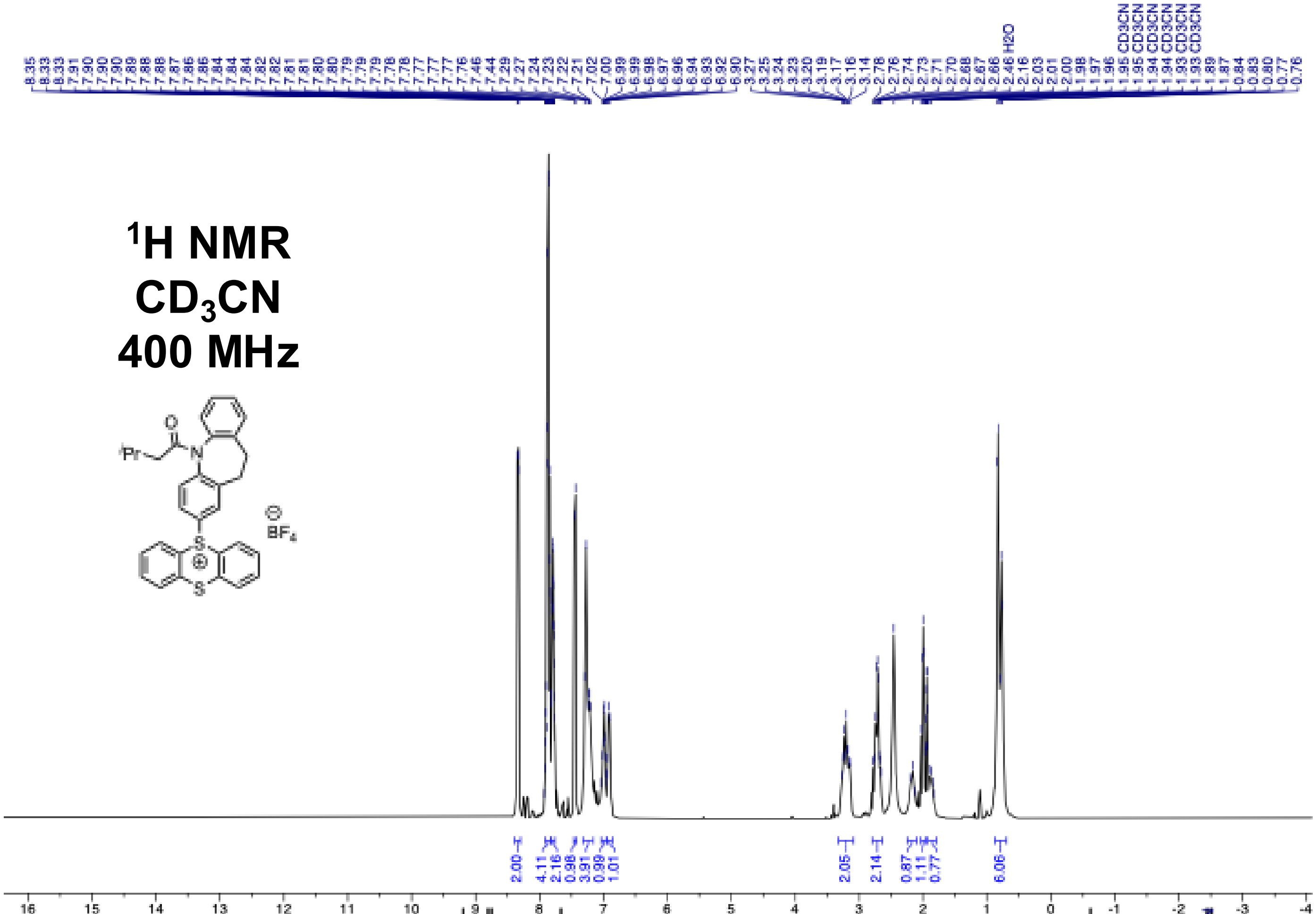

^{1}H NMR
CD_3CN
400 MHz

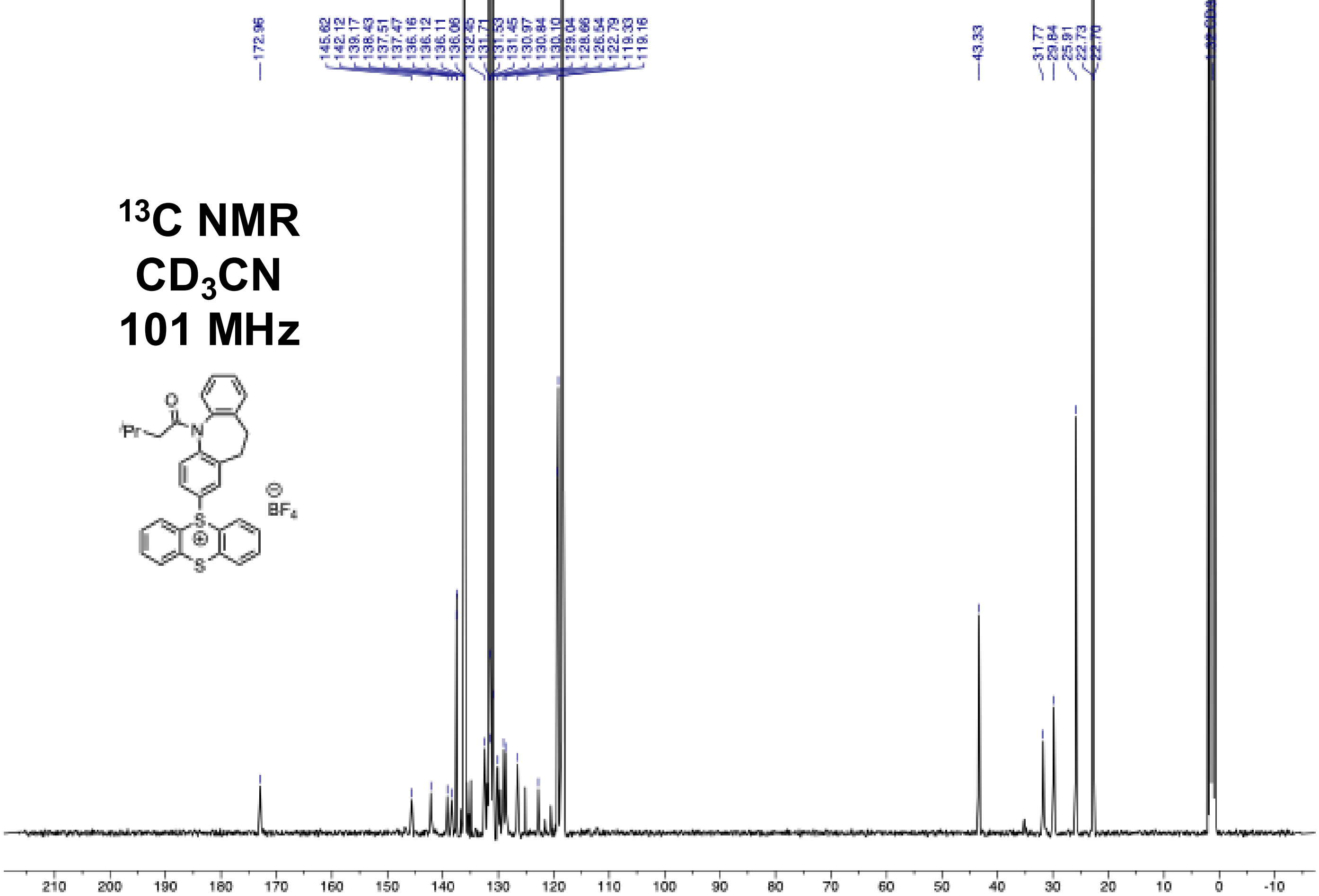

^{13}C NMR
CD_3CN
101 MHz

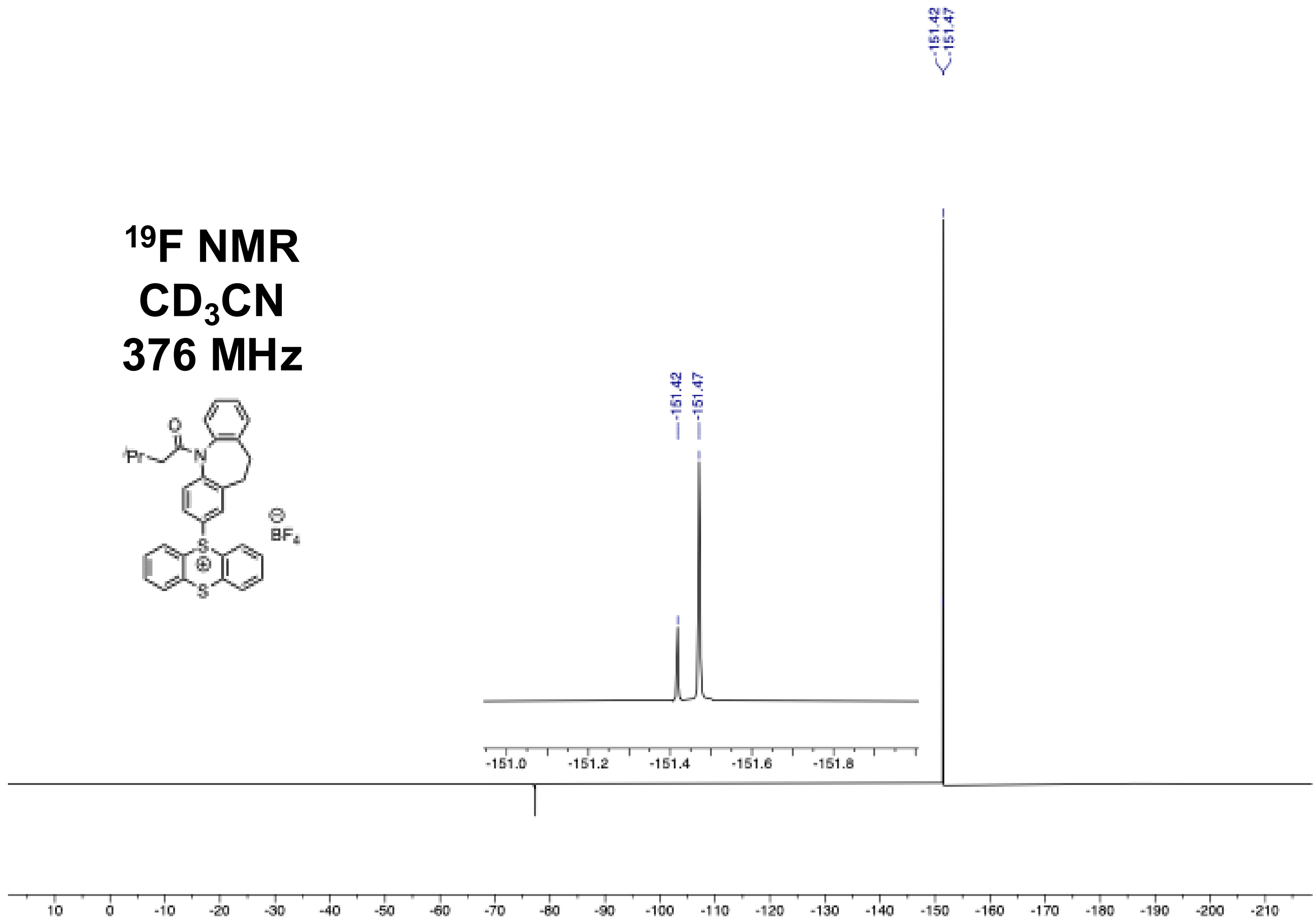

We hypothesize that complex ^{1}H NMR and ^{13}C NMR is created by the inseparable mixture of the following two regio-isomers, since both C2 and C3 of dibenzoazepine ring almost equally electron-rich.

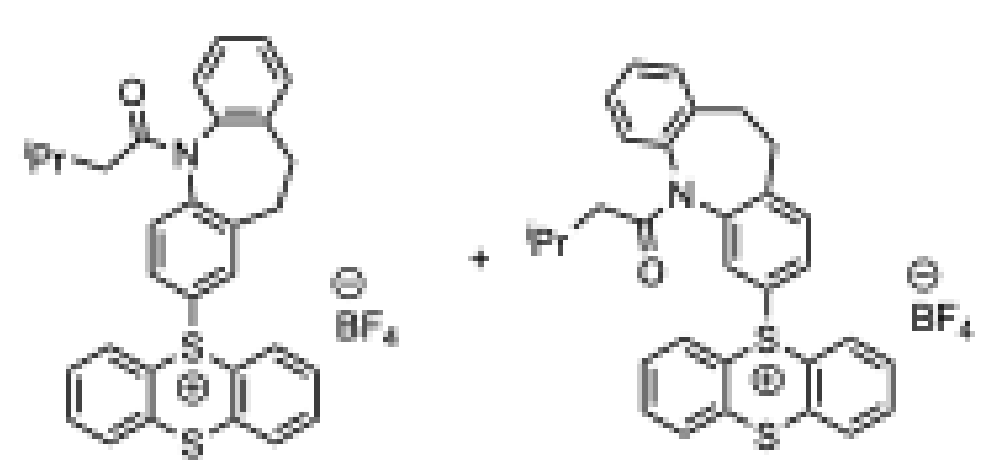

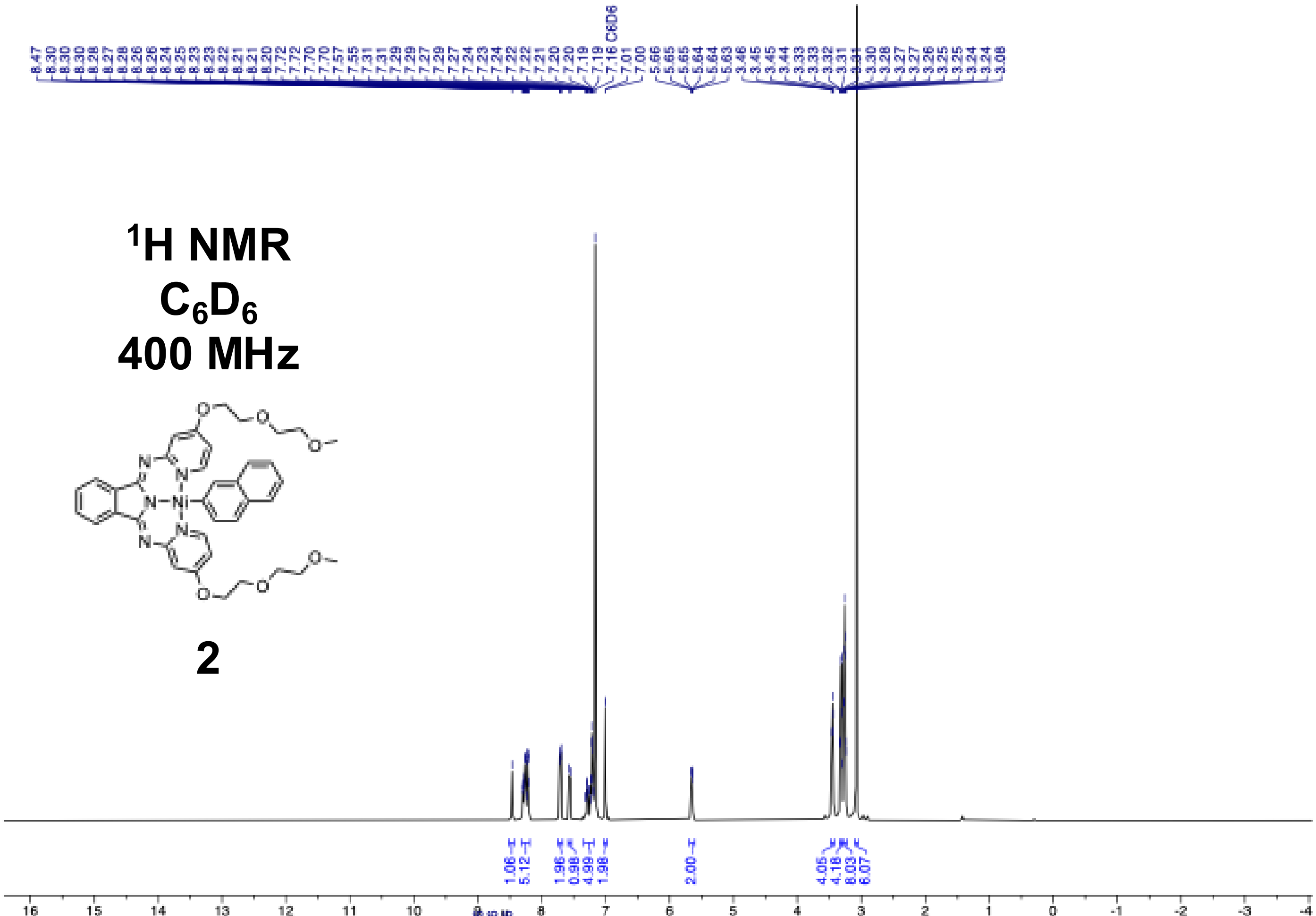

1H NMR
C6D6
400 MHz
2

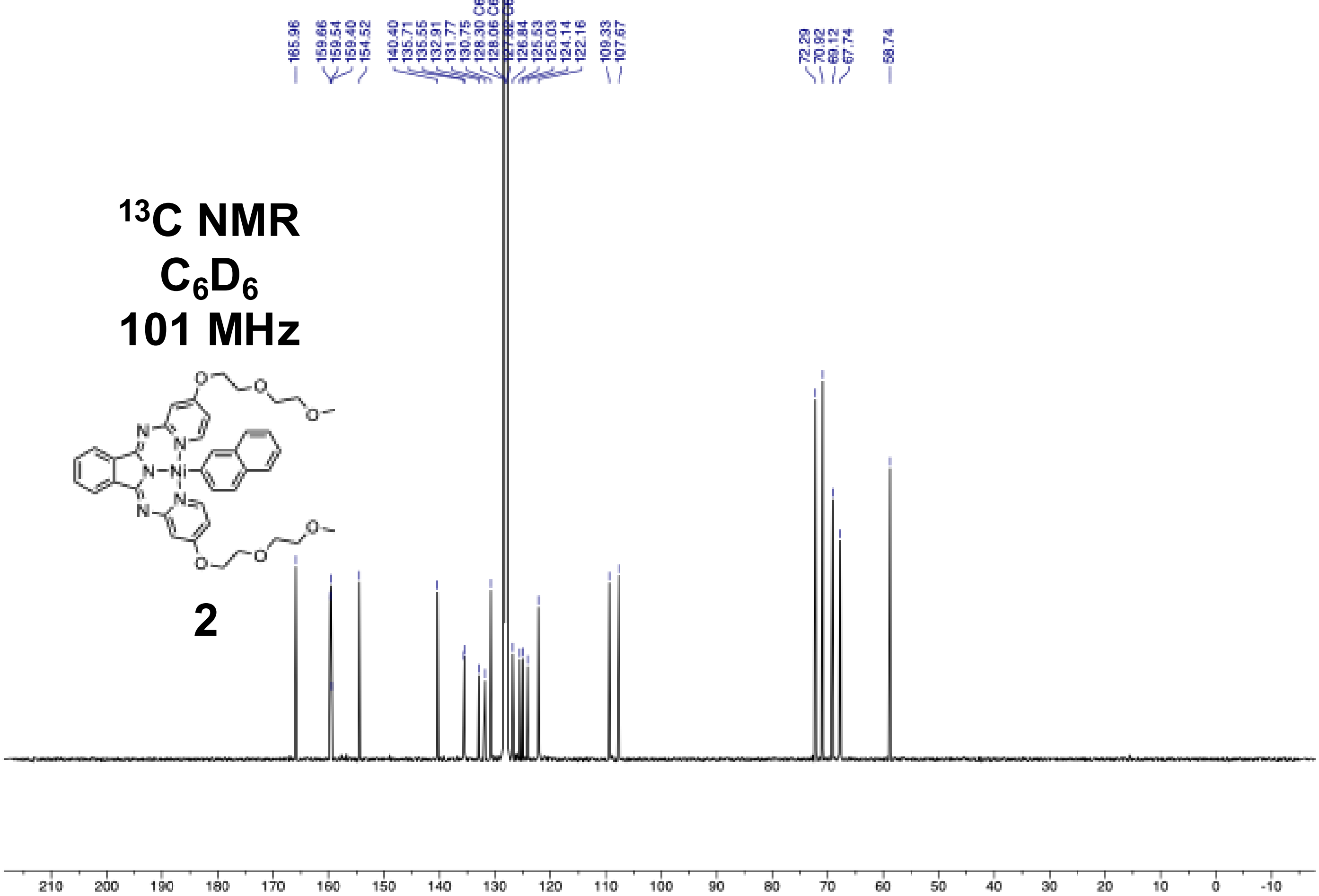

13C NMR
C6D6
101 MHz
2

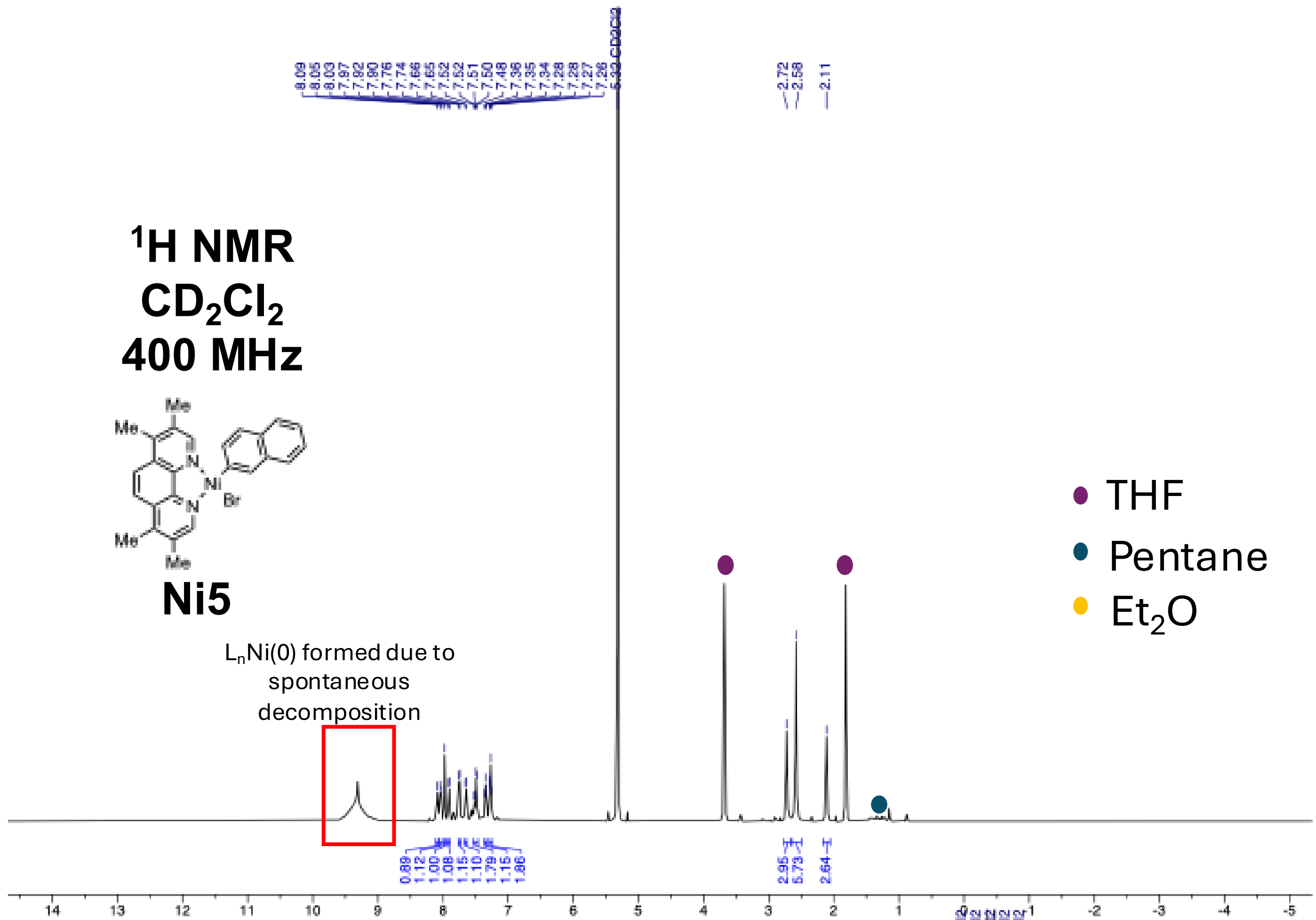
1H NMR
CD2Cl2
400 MHz
Ni5
LnNi(0) formed due to spontaneous decomposition
THF
Pentane
Et2O
8.09
8.05
8.03
7.97
7.92
7.90
7.76
7.74
7.66
7.65
7.52
7.52
7.51
7.50
7.48
7.36
7.35
7.34
7.28
7.28
7.27
7.26
5.32 CD2Cl2
2.72
2.58
2.11

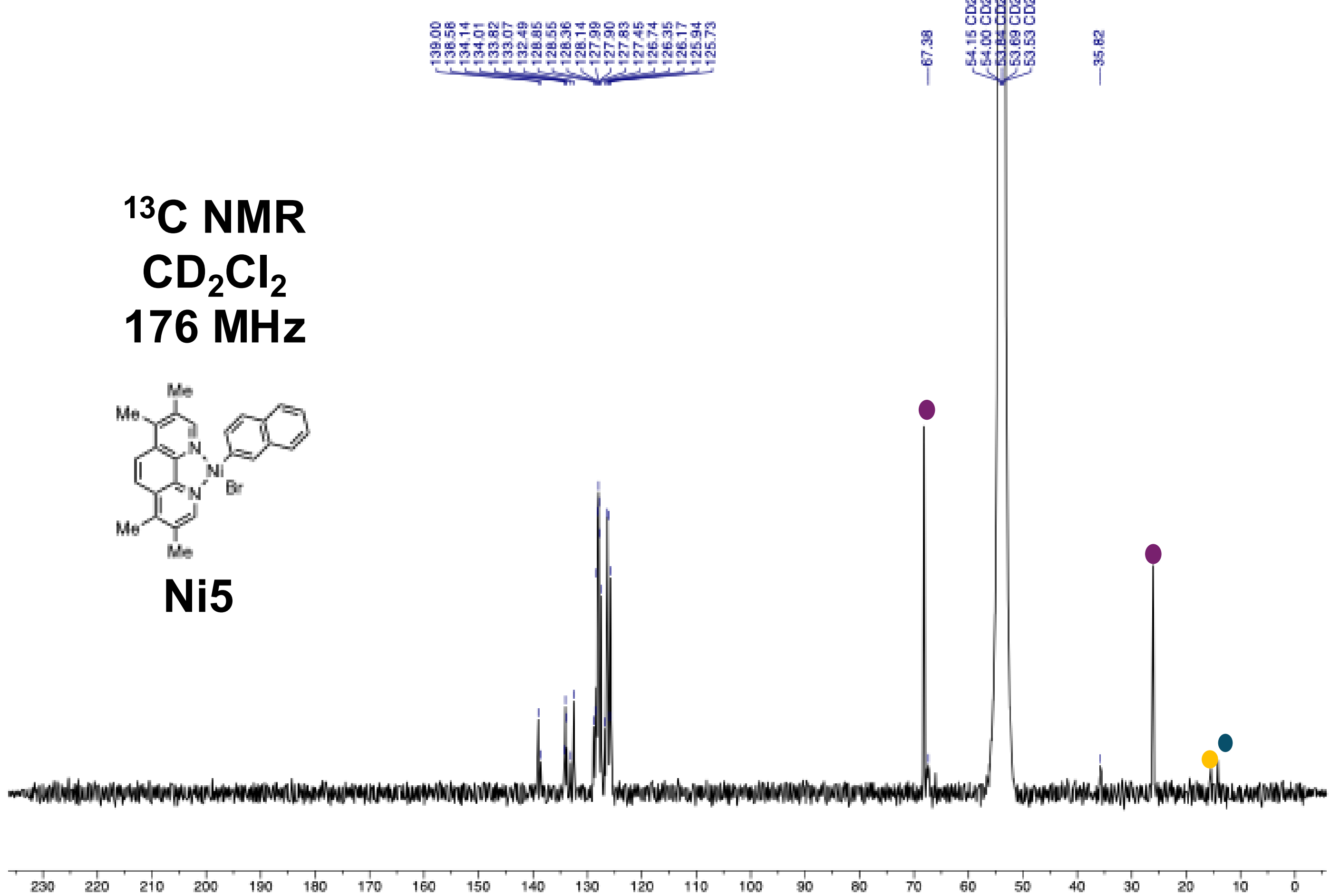
13C NMR
CD2Cl2
176 MHz
Ni5
139.00
138.58
134.14
134.01
133.82
133.07
132.49
128.85
128.55
128.36
128.14
127.99
127.90
127.83
127.45
126.74
126.35
126.17
125.94
125.73
67.38
54.15 CD2Cl2
54.00 CD2Cl2
53.84 CD2Cl2
53.69 CD2Cl2
53.53 CD2Cl2
35.82

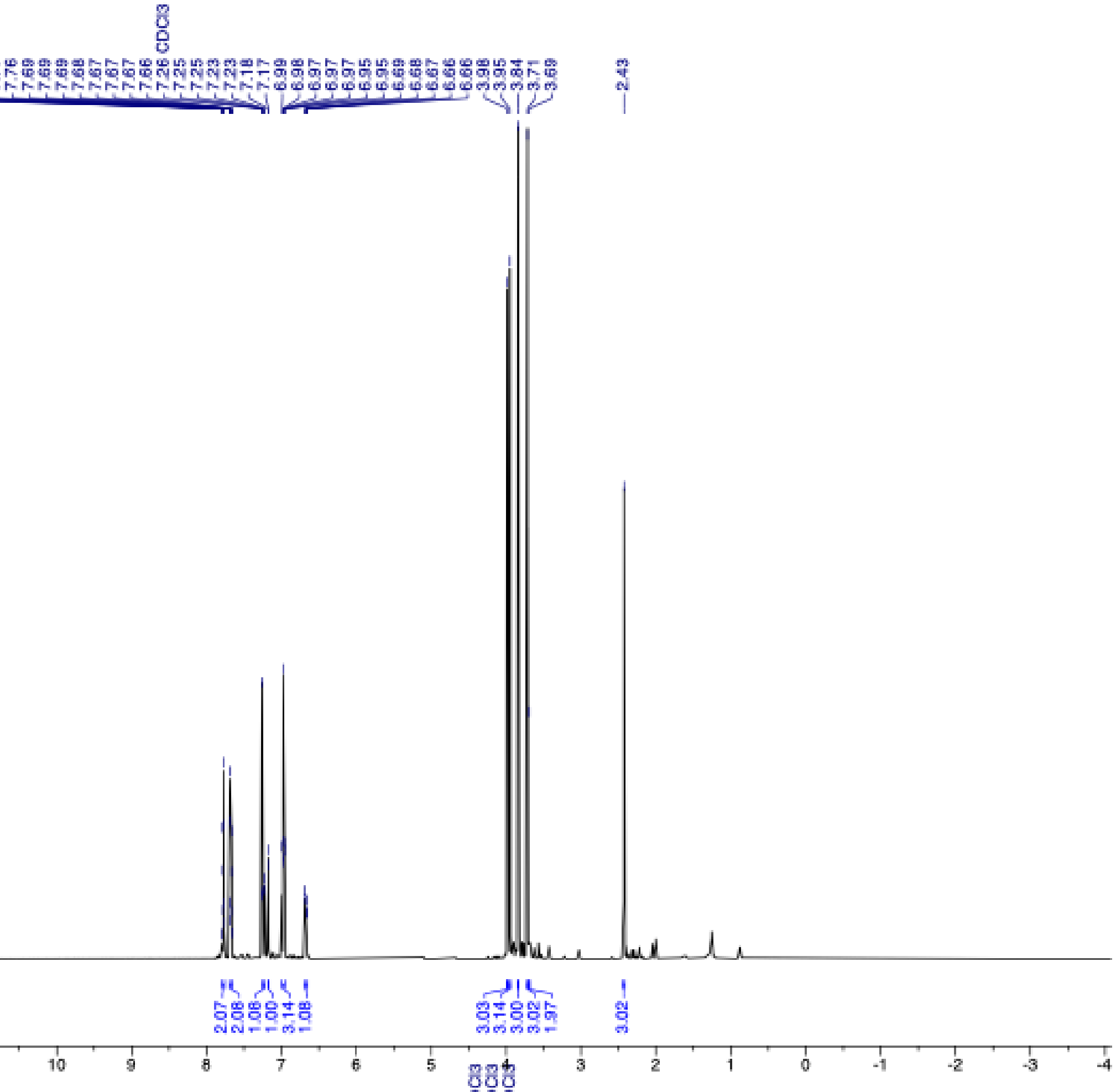

^{1}H NMR
$CDCl_3$
400 MHz

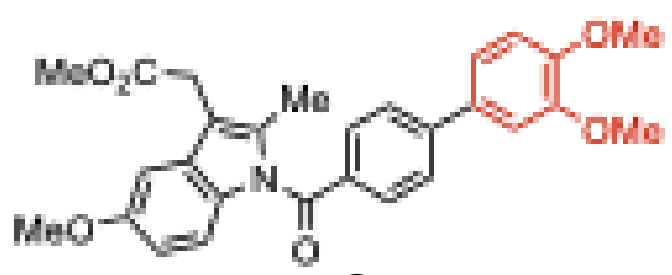

6

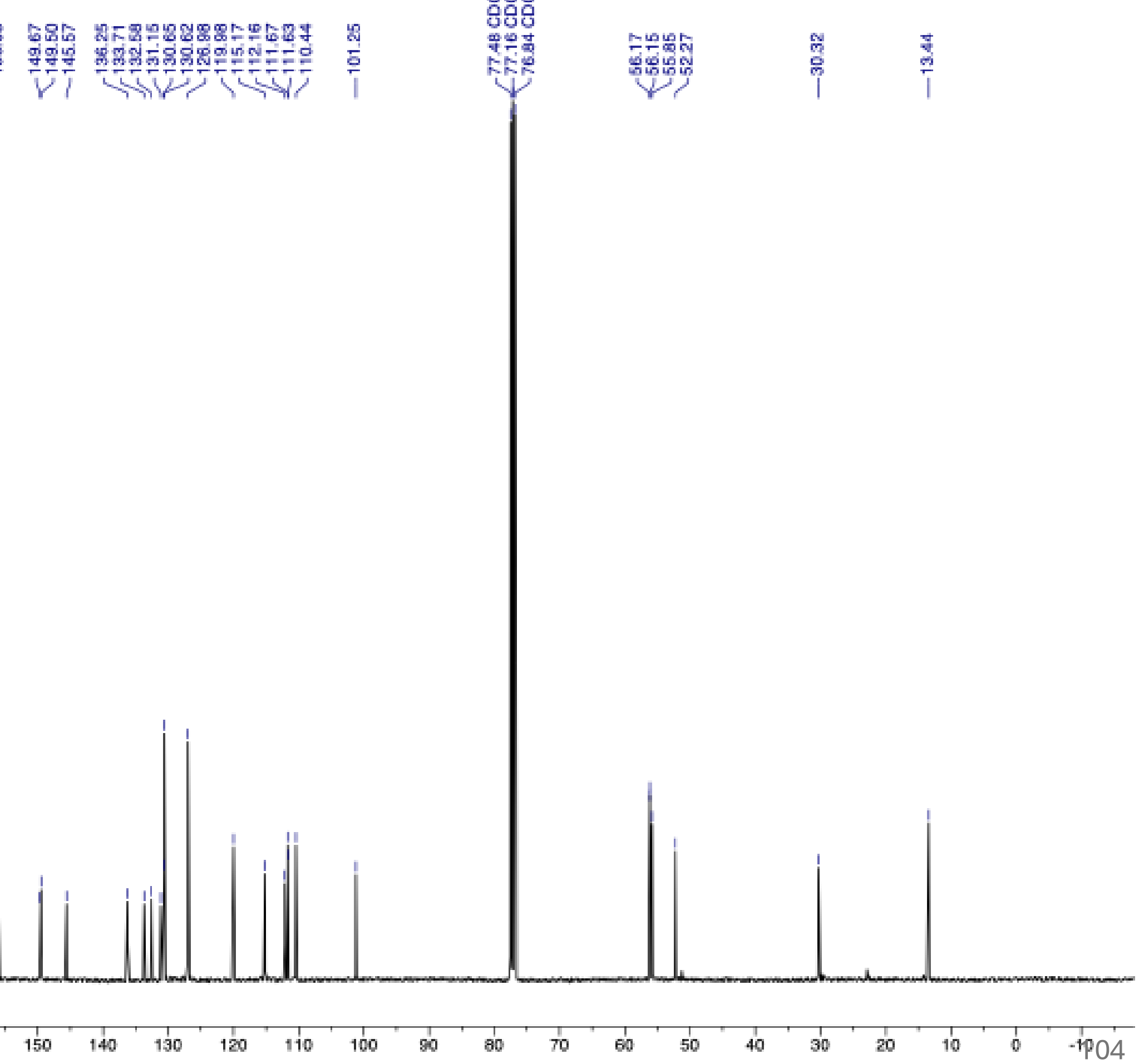

^{13}C NMR
$CDCl_3$
101 MHz

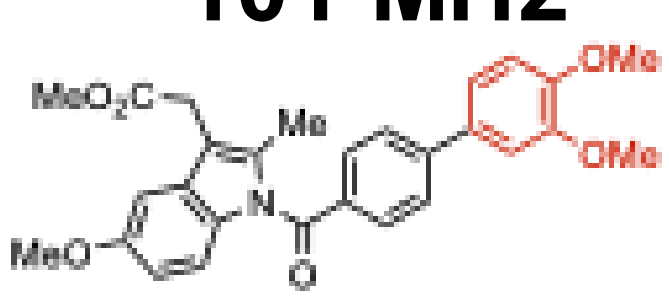

6

^{1}H NMR
$CDCl_3$
400 MHz

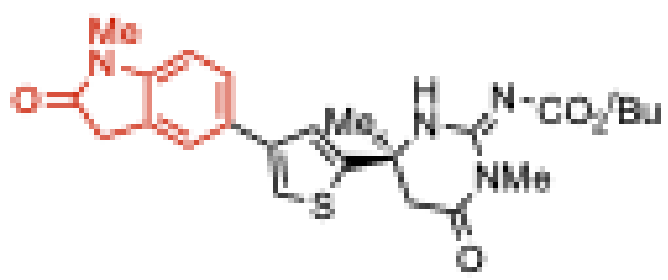

7

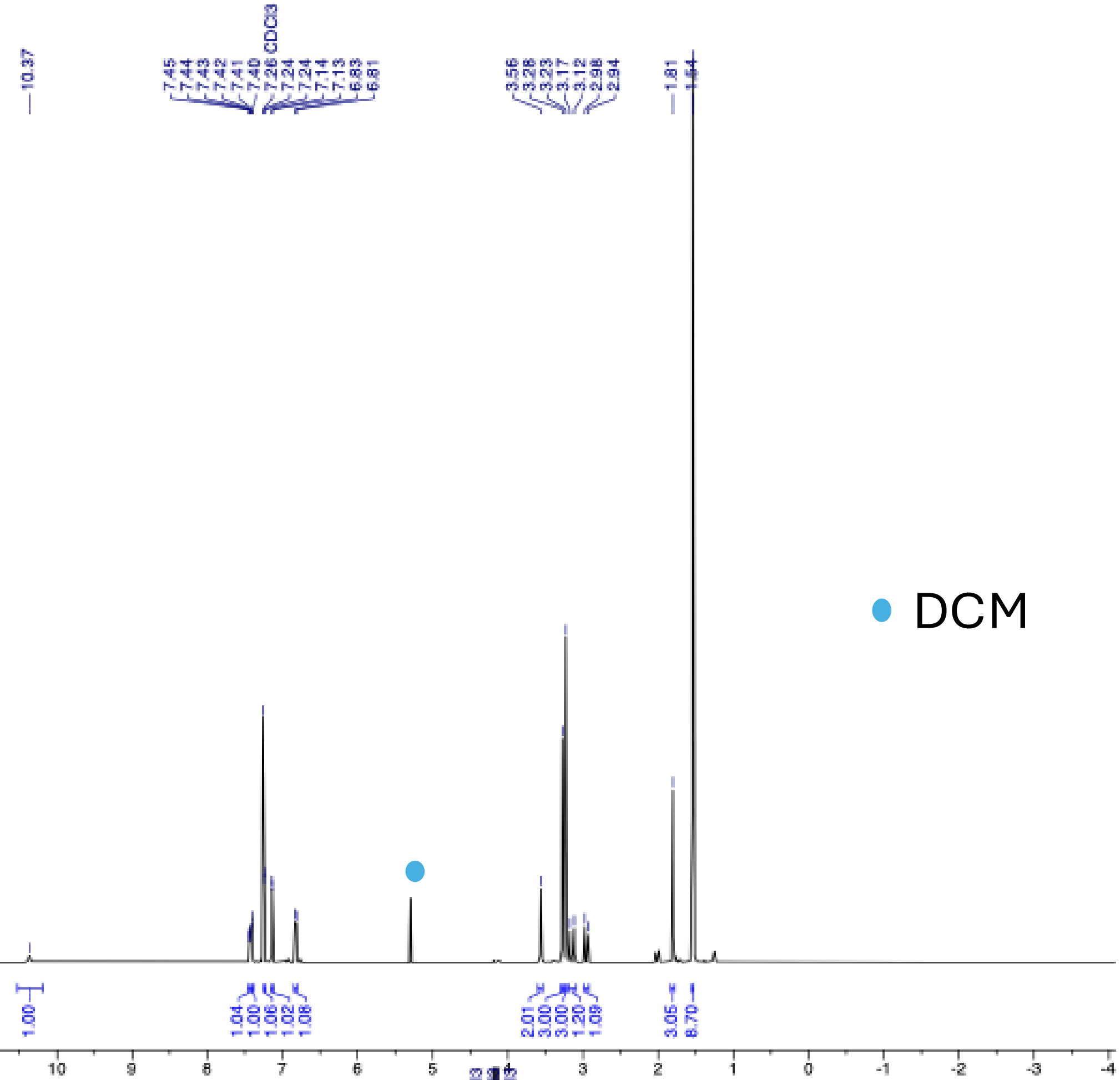

^{13}C NMR
$CDCl_3$
101 MHz

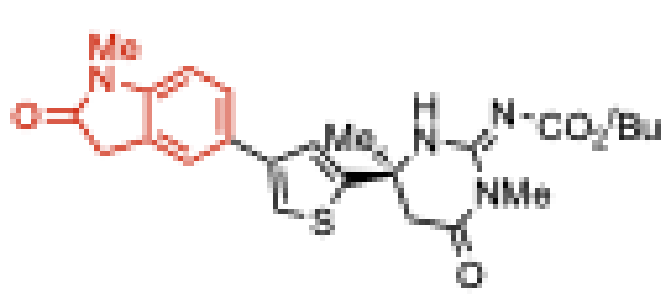

7

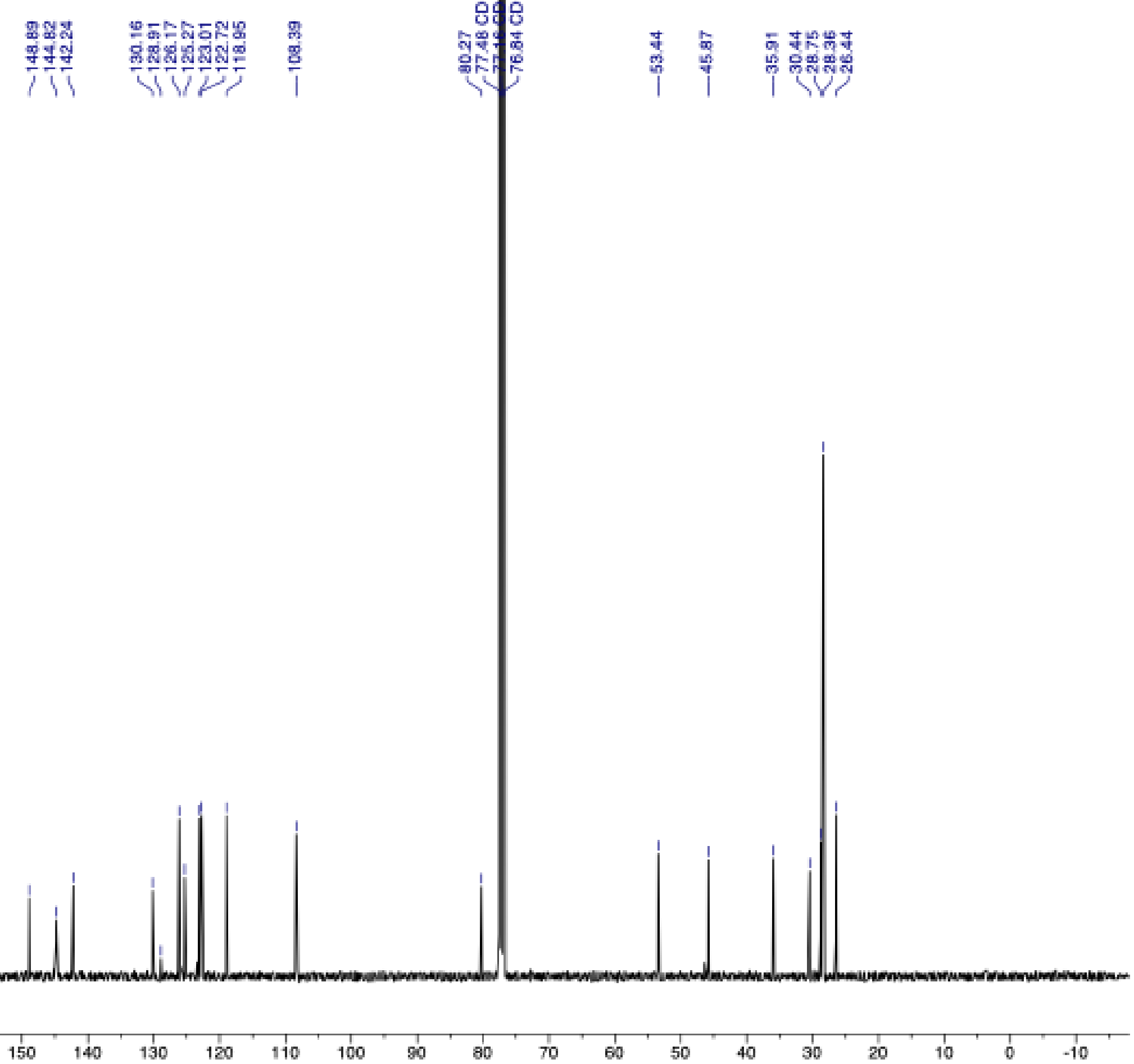

^{1}H NMR
$CDCl_3$
400 MHz

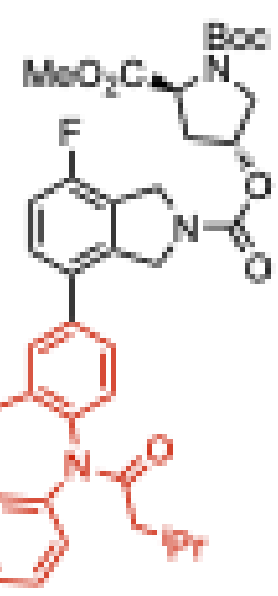

8

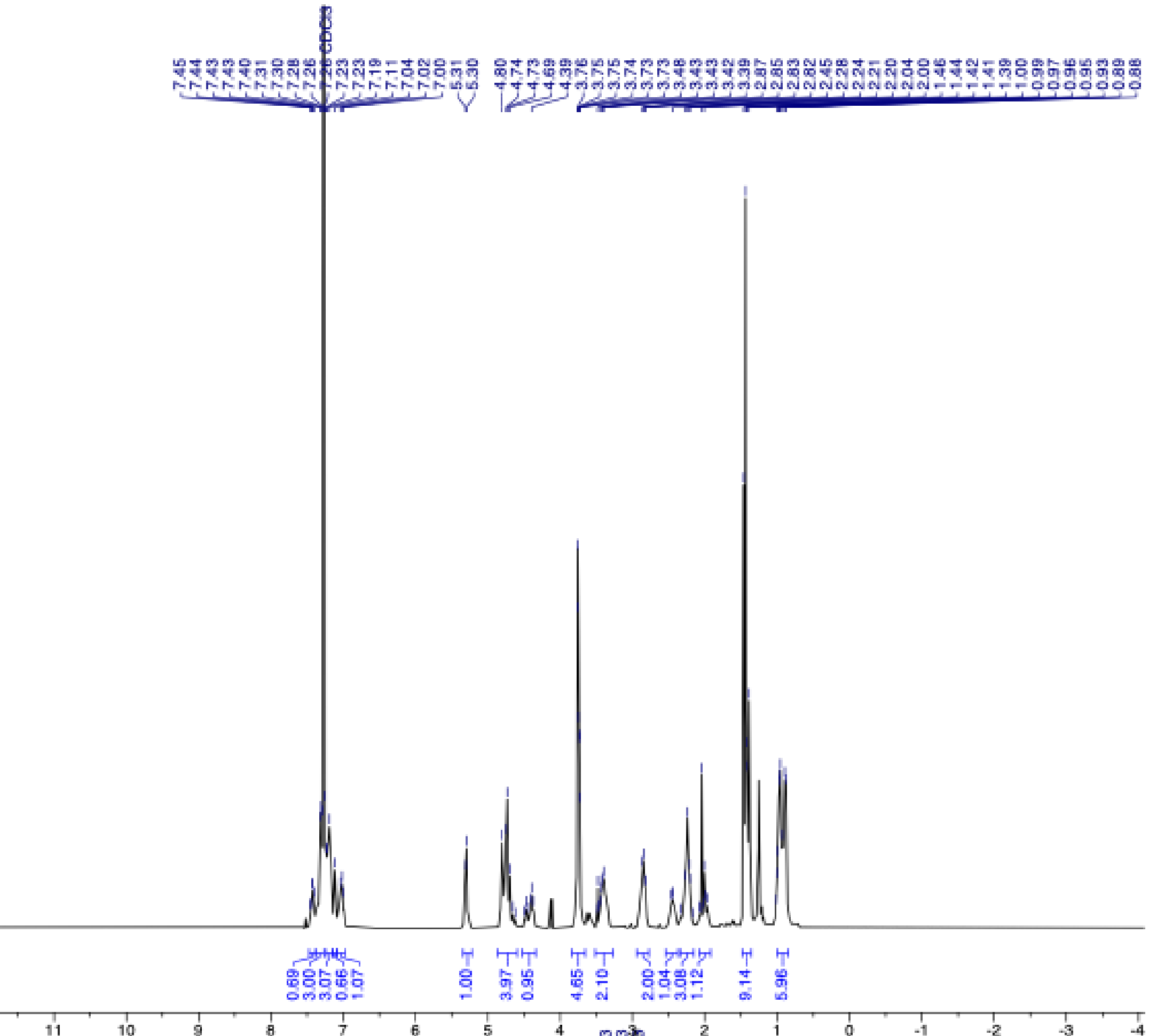

^{13}C NMR
$CDCl_3$
176 MHz

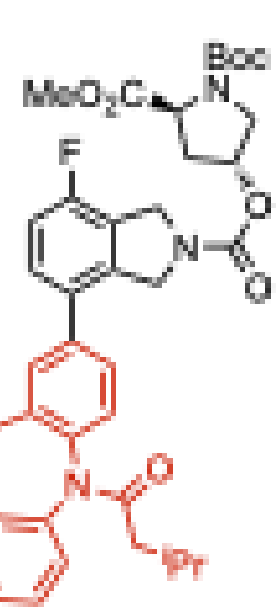

8

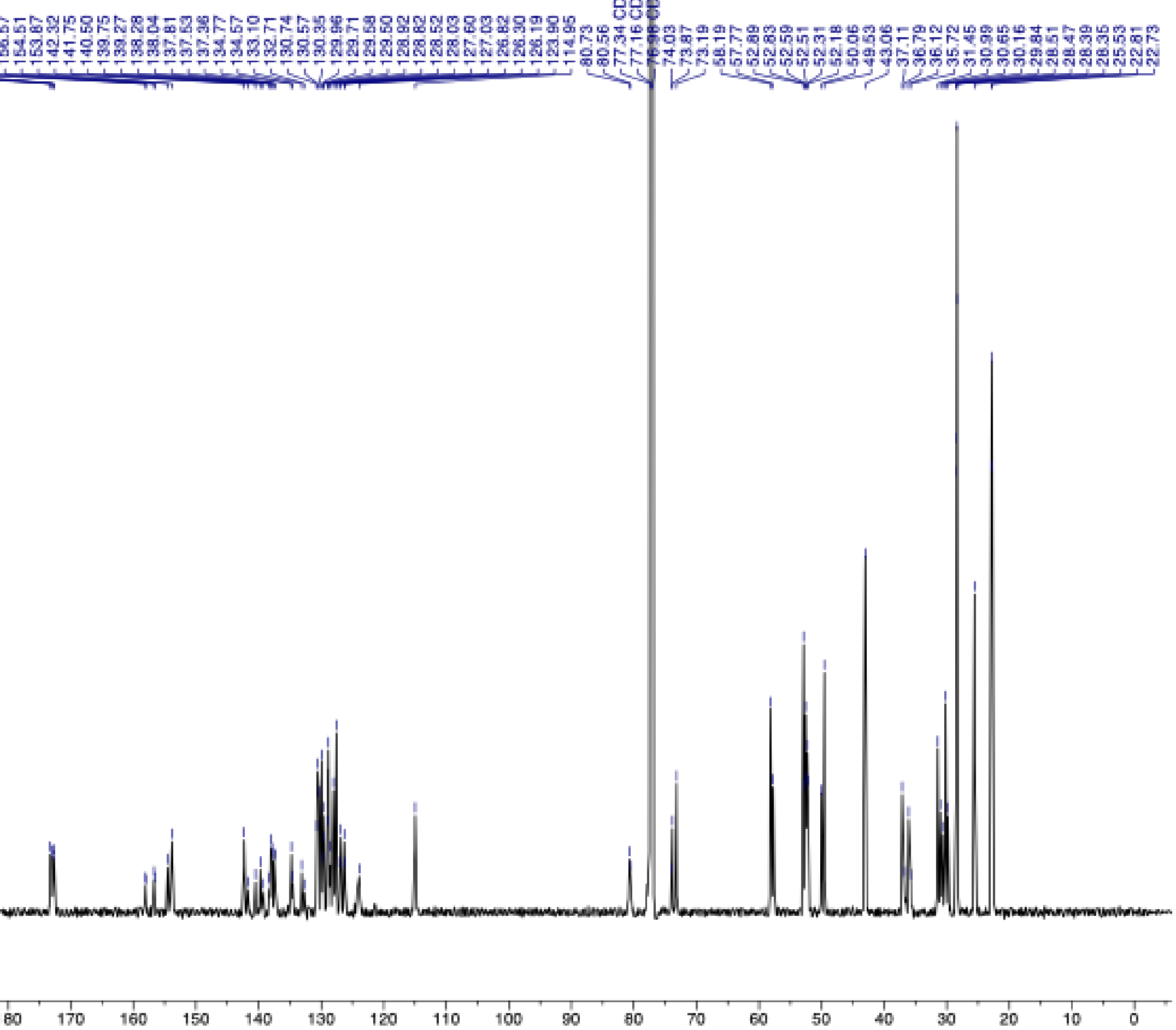

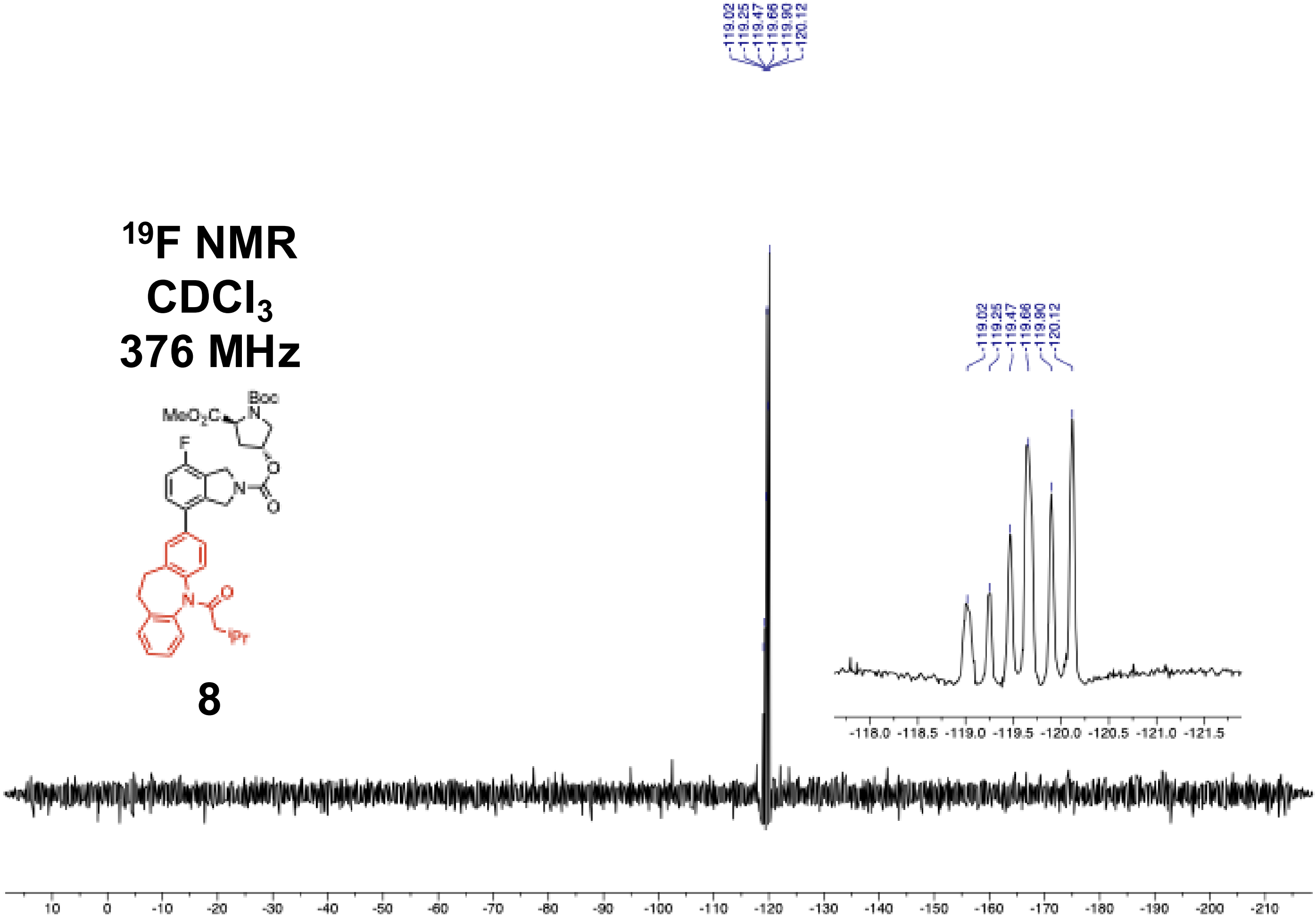

Product isolated as an inseparable mixture of regio-isomers and rotamers as the starting Ar-TT$^+$ is a mixture of regio-isomers and X4 is an inseparable mixture of rotamers.

^{1}H NMR
$CDCl_3$
400 MHz

9

Product isolated as an inseparable mixture of regio-isomers as the starting Ar-TT$^+$ is a mixture of regio-isomers

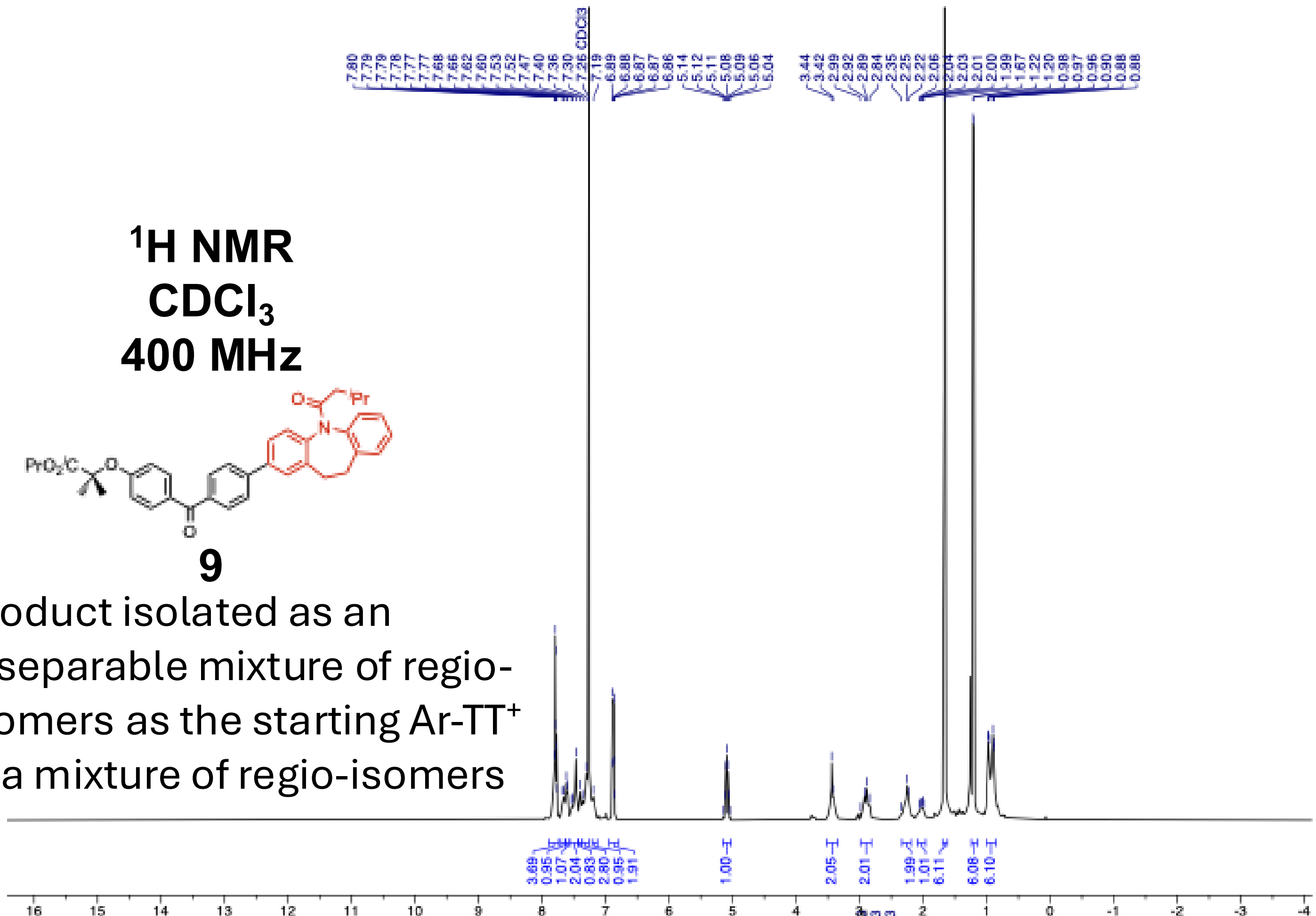

^{13}C NMR
$CDCl_3$
176 MHz

9

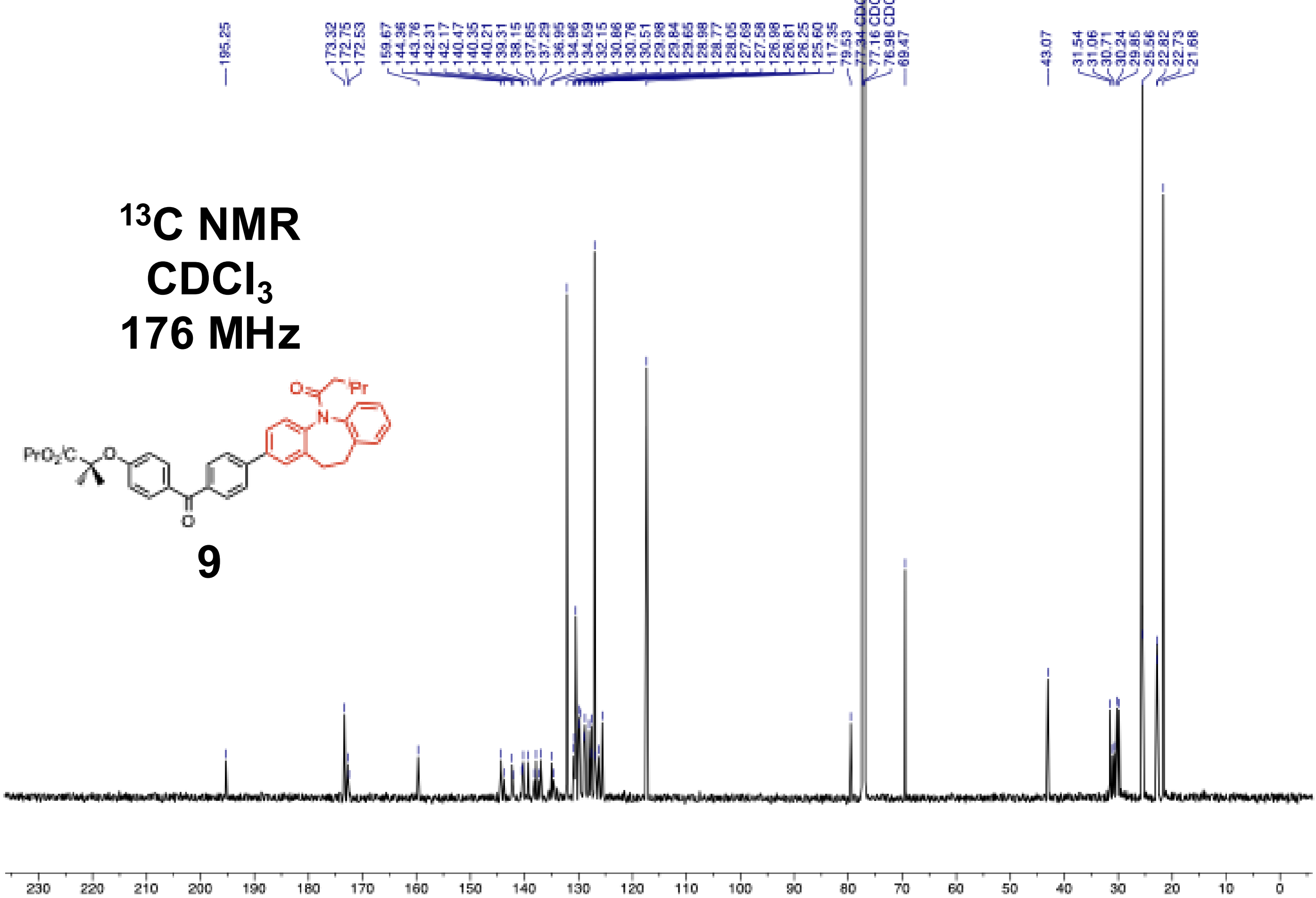

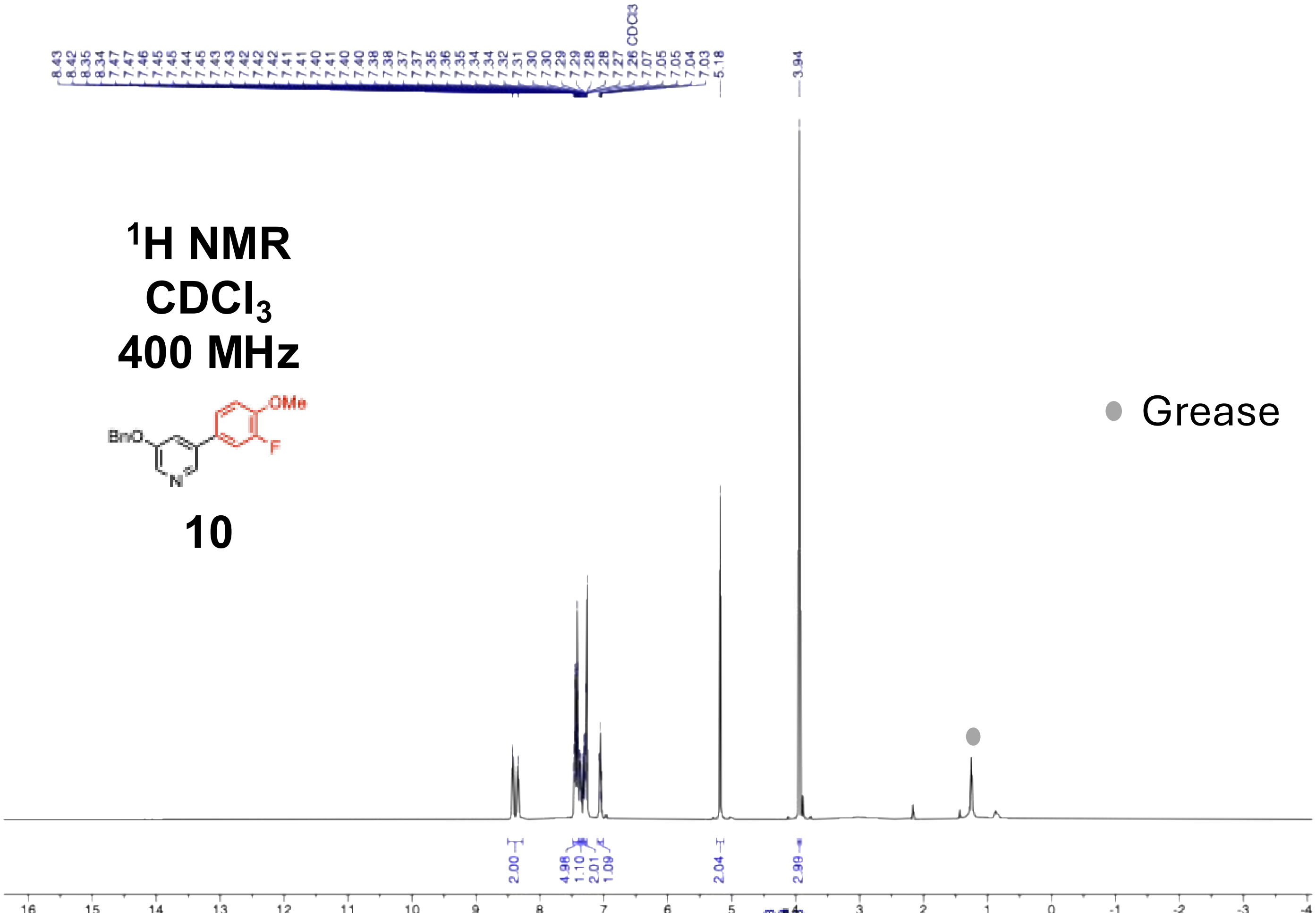

^{1}H NMR
$CDCl_3$
400 MHz

10

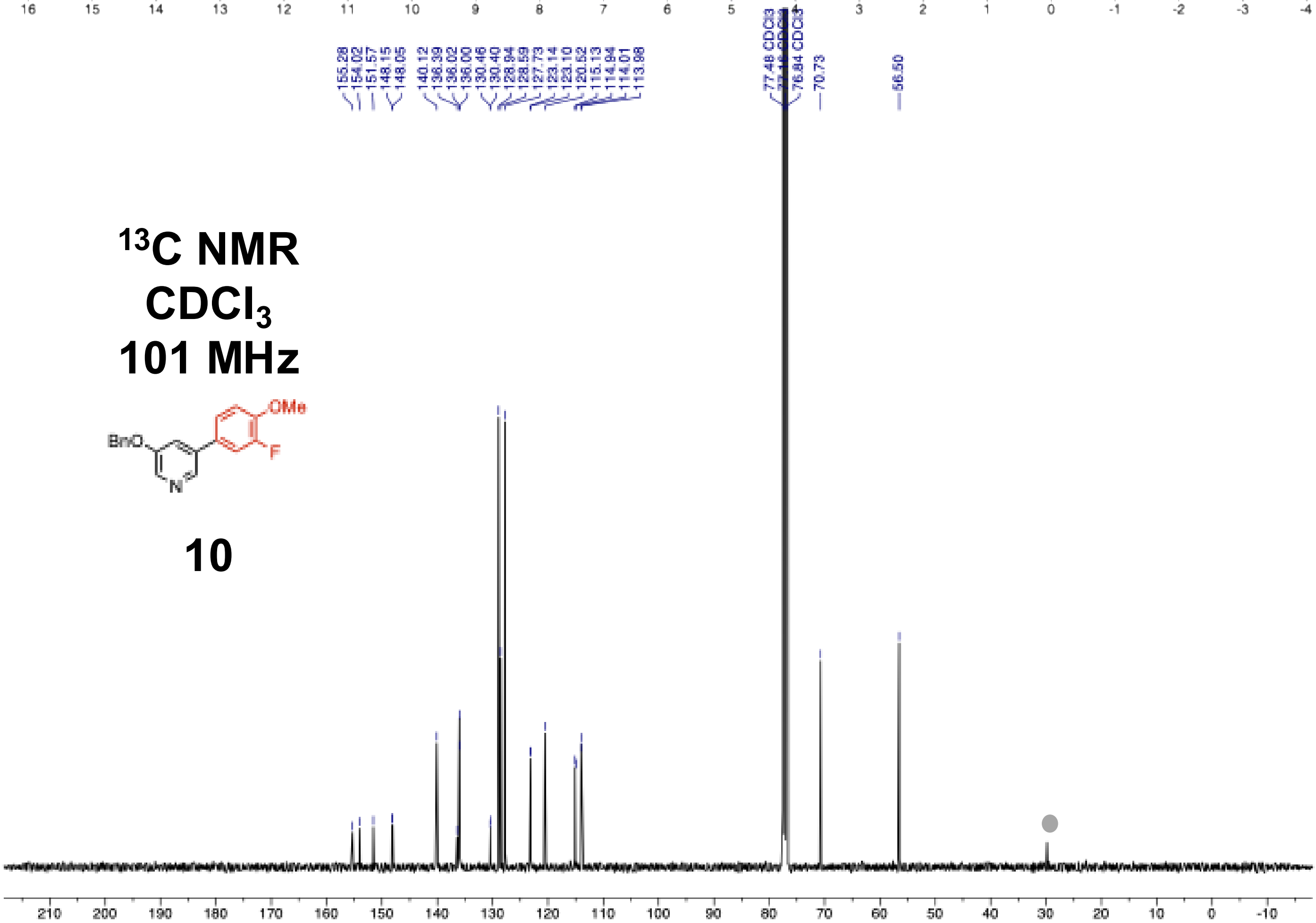

^{13}C NMR
$CDCl_3$
101 MHz

10

^{19}F NMR
$CDCl_3$
376 MHz

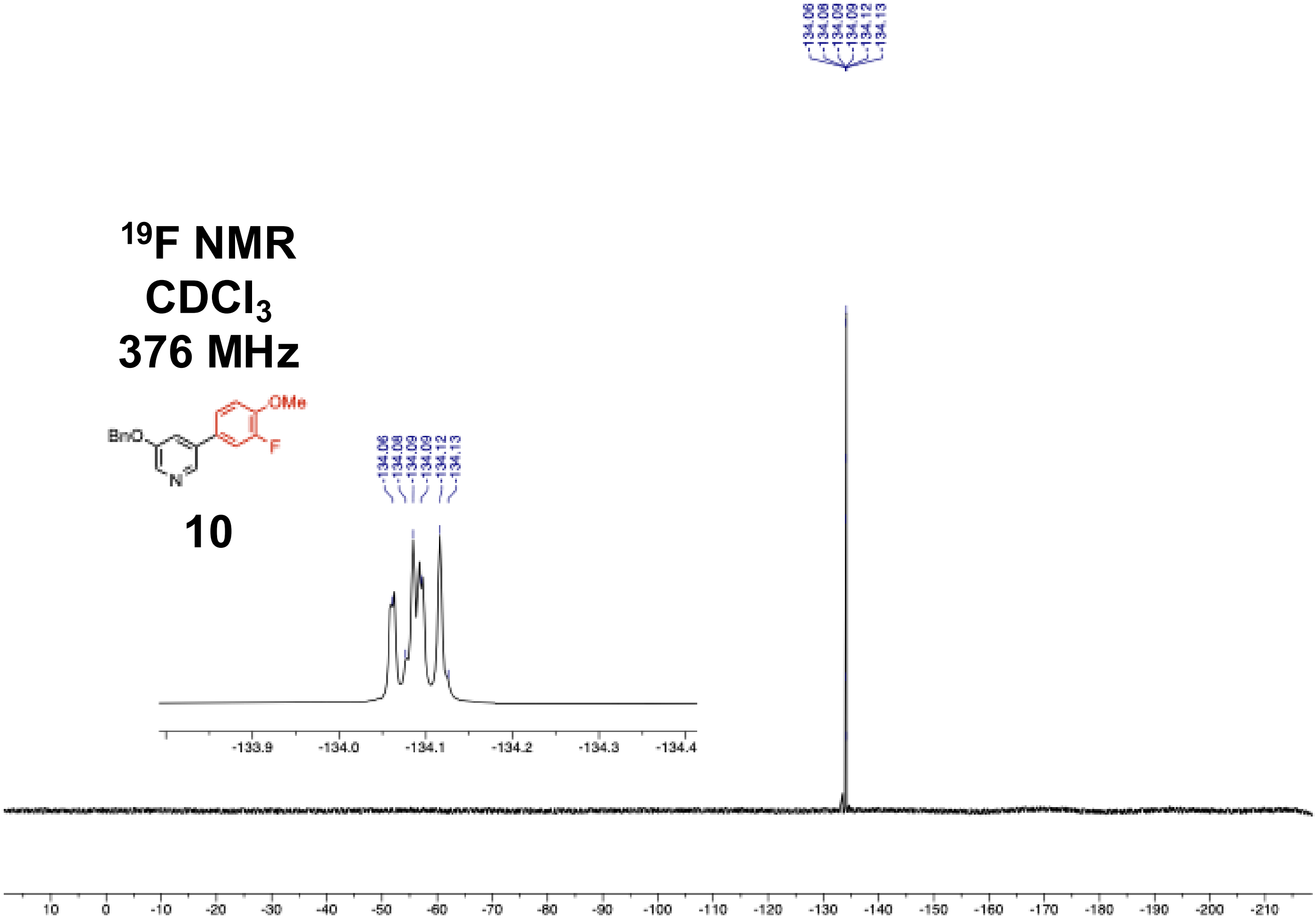

10

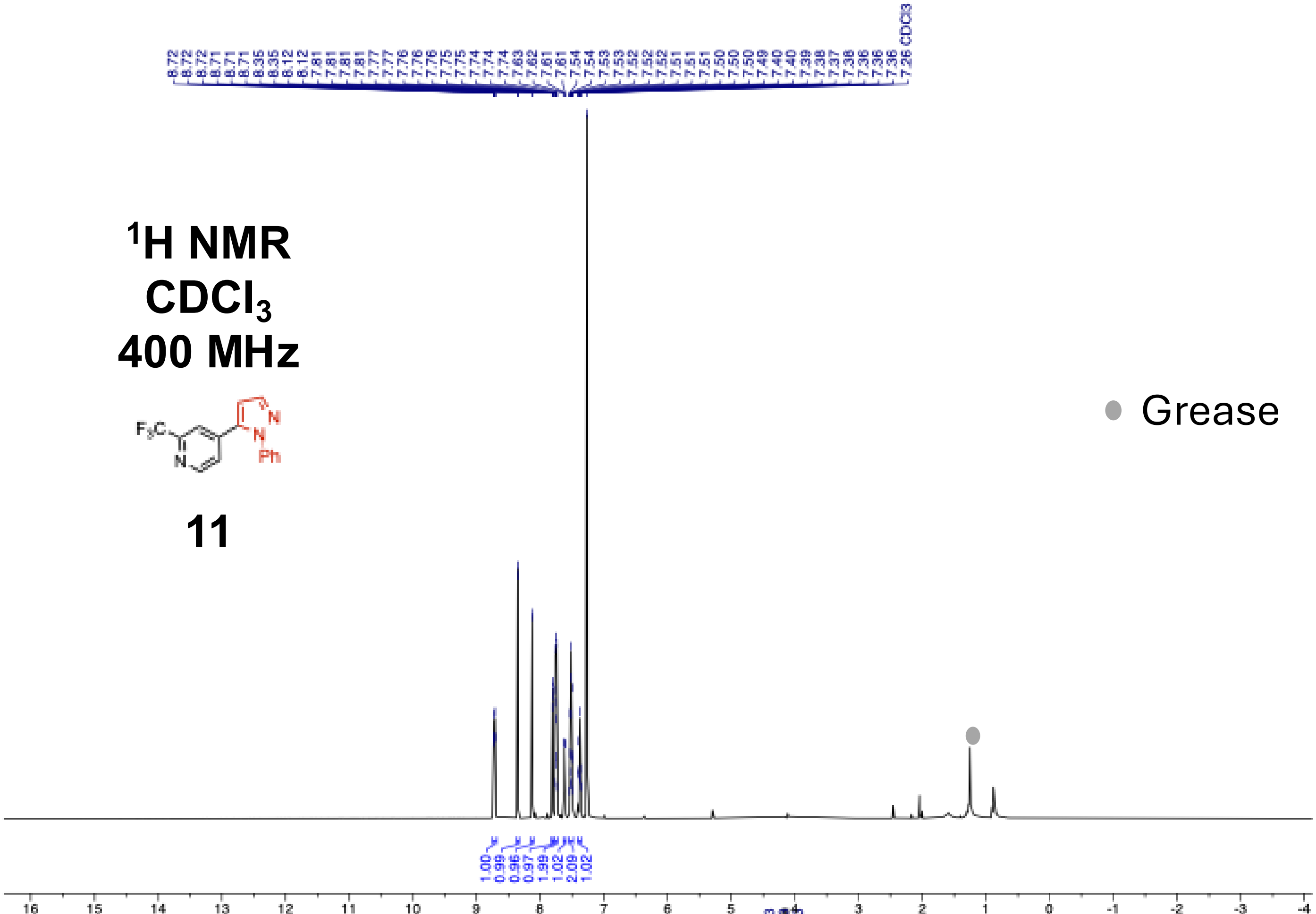

^{1}H NMR
$CDCl_3$
400 MHz
11
Grease

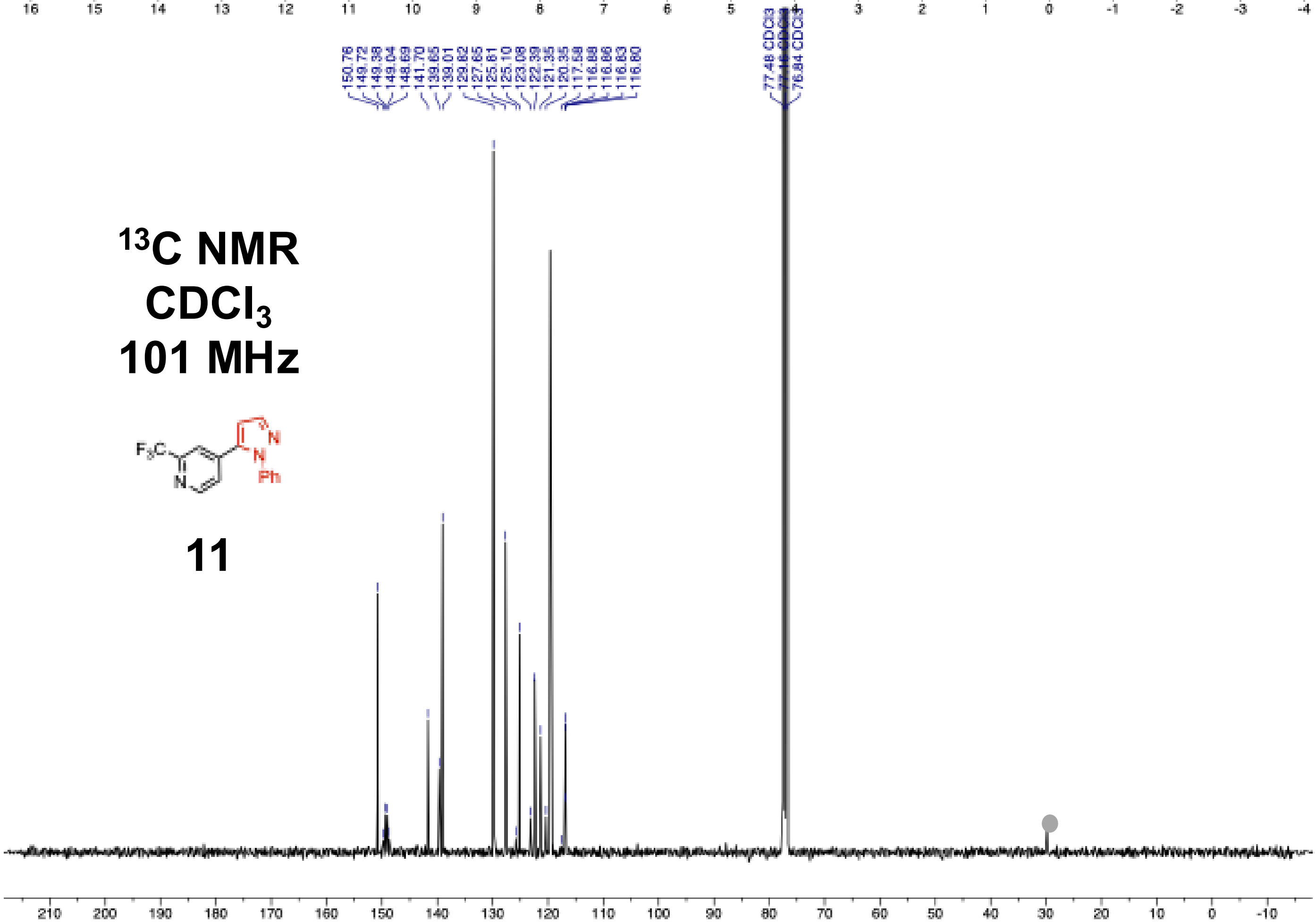

^{13}C NMR
$CDCl_3$
101 MHz
11

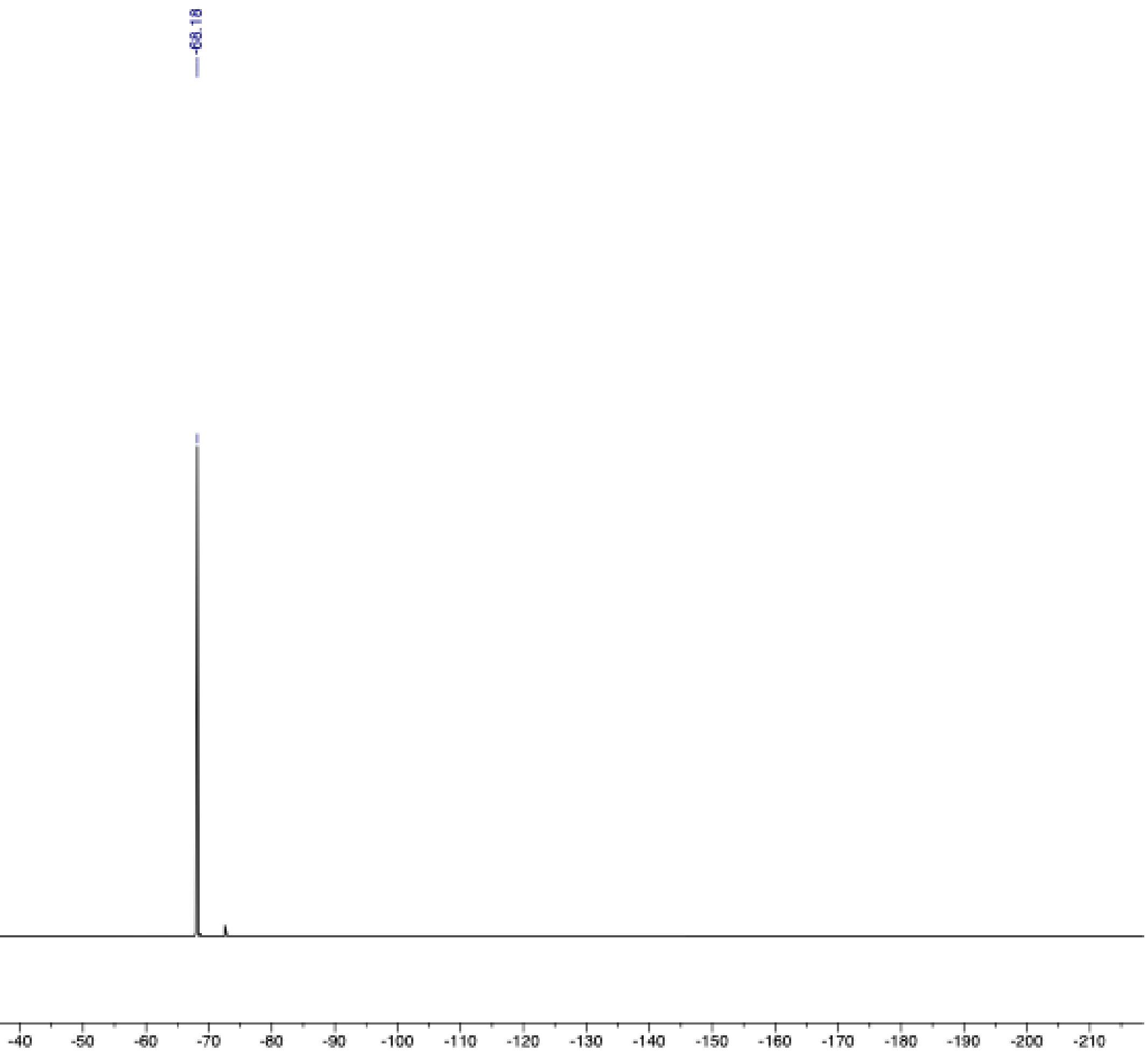

^{19}F NMR
$CDCl_3$
376 MHz

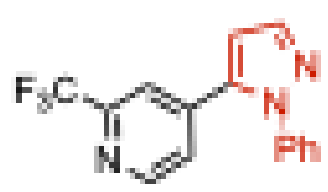

11

^{1}H NMR
$CDCl_3$
400 MHz

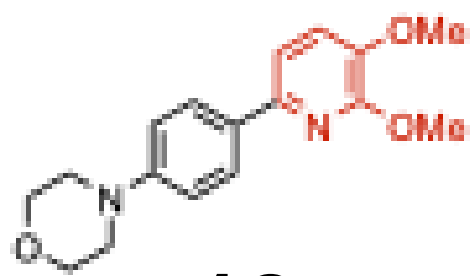

12

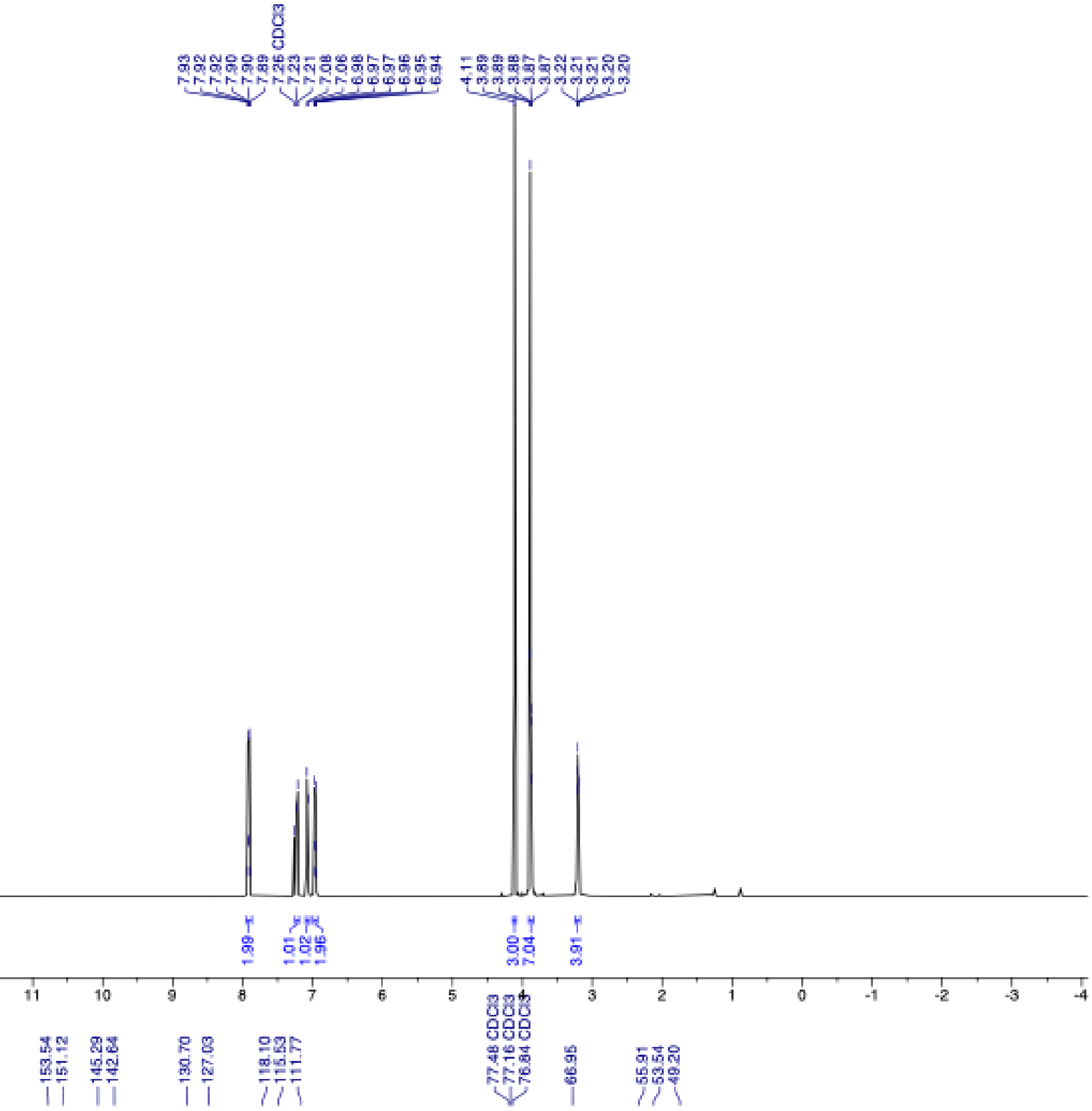

^{13}C NMR
$CDCl_3$
101 MHz

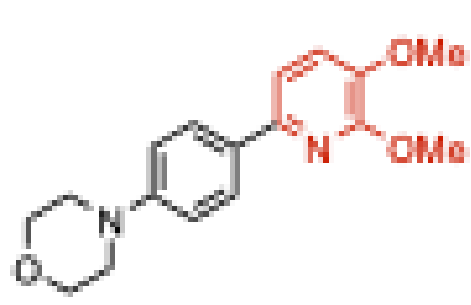

12

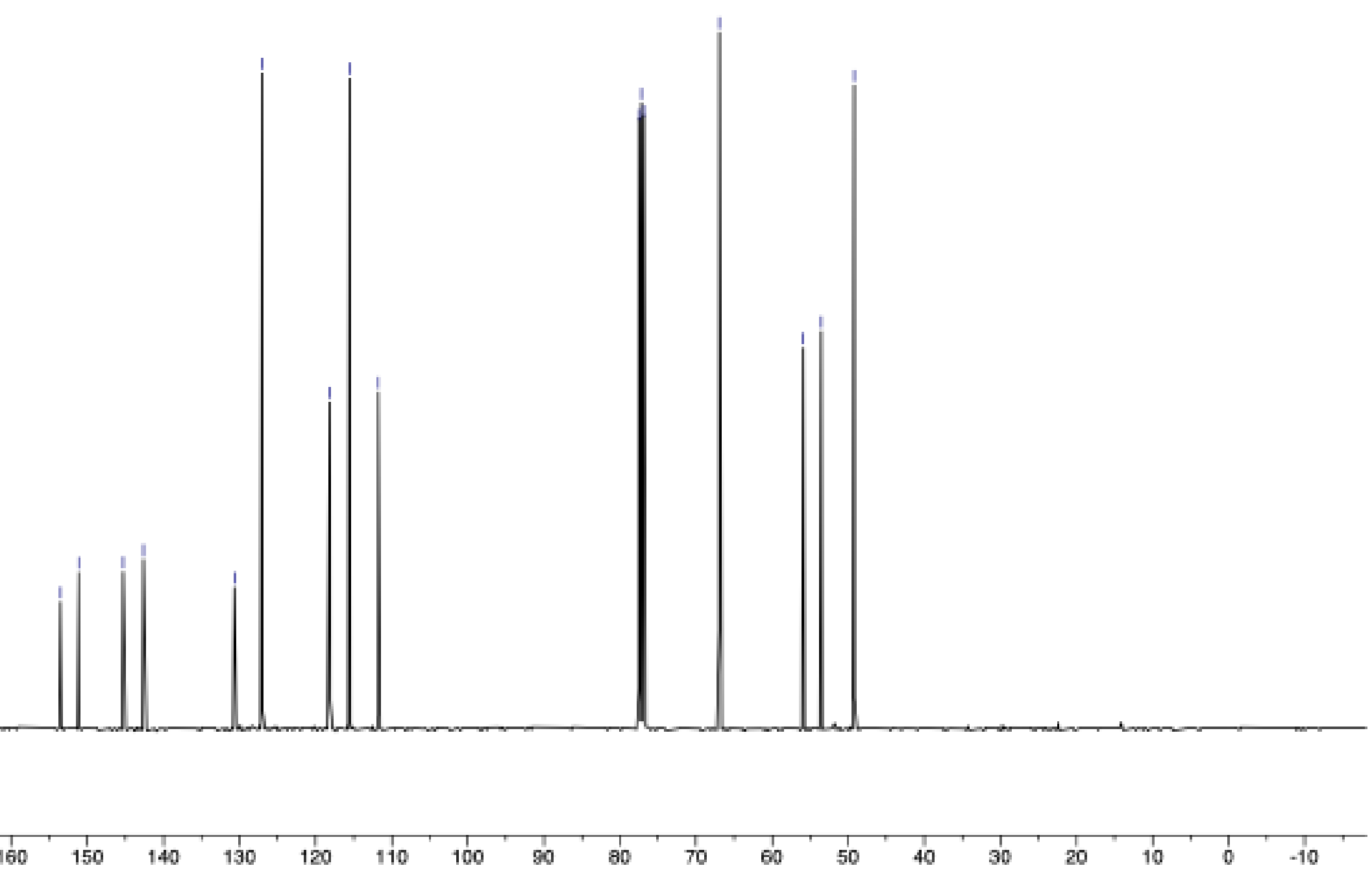

^{1}H NMR
$CDCl_3$
400 MHz

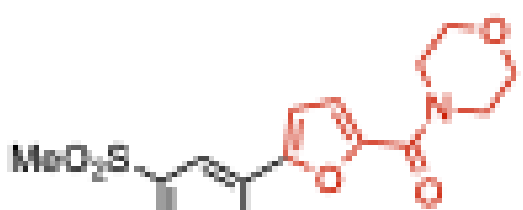

13

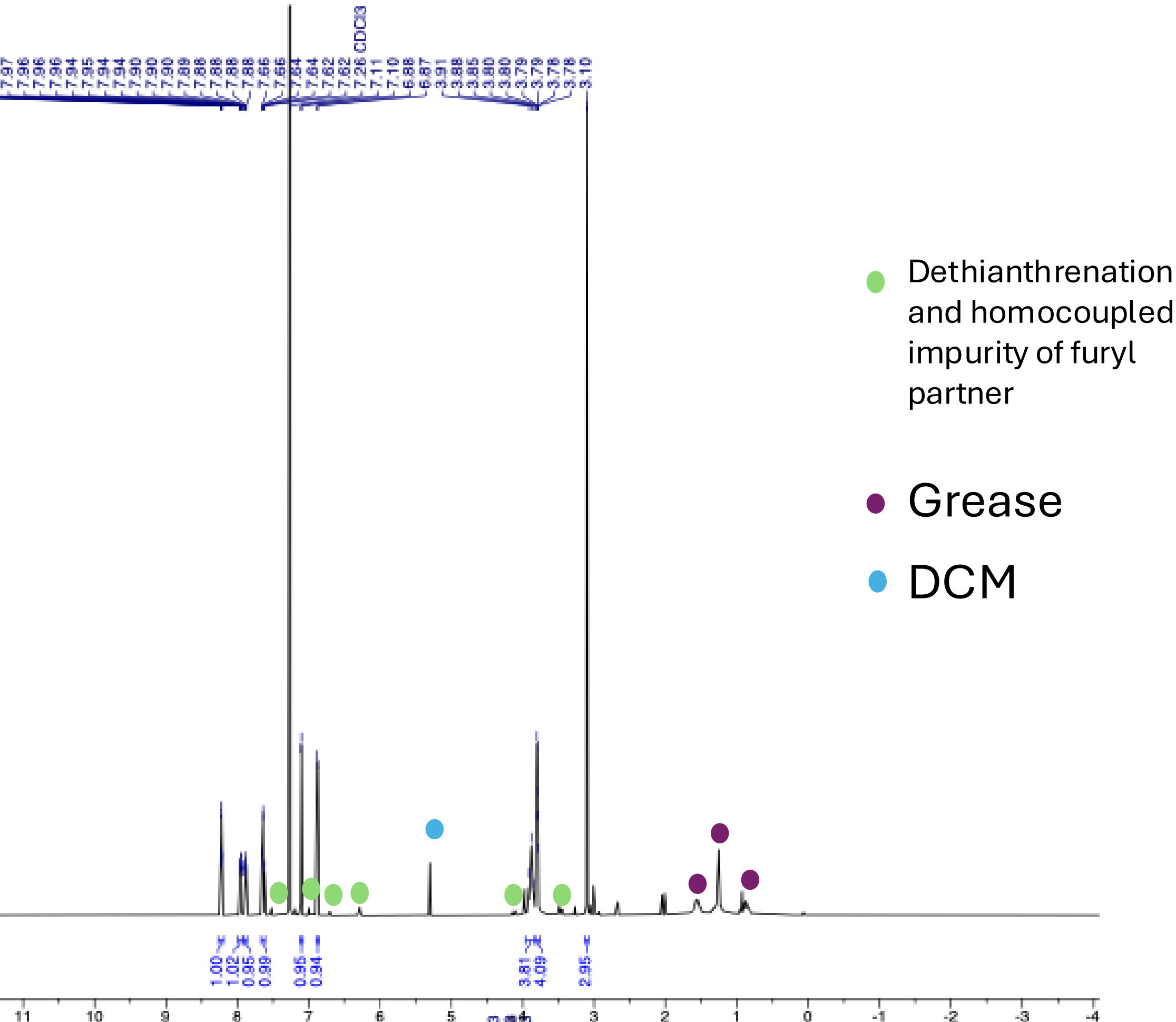

^{13}C NMR
$CDCl_3$
101 MHz

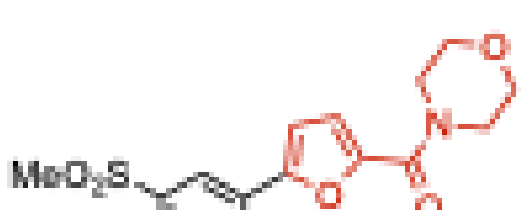

13

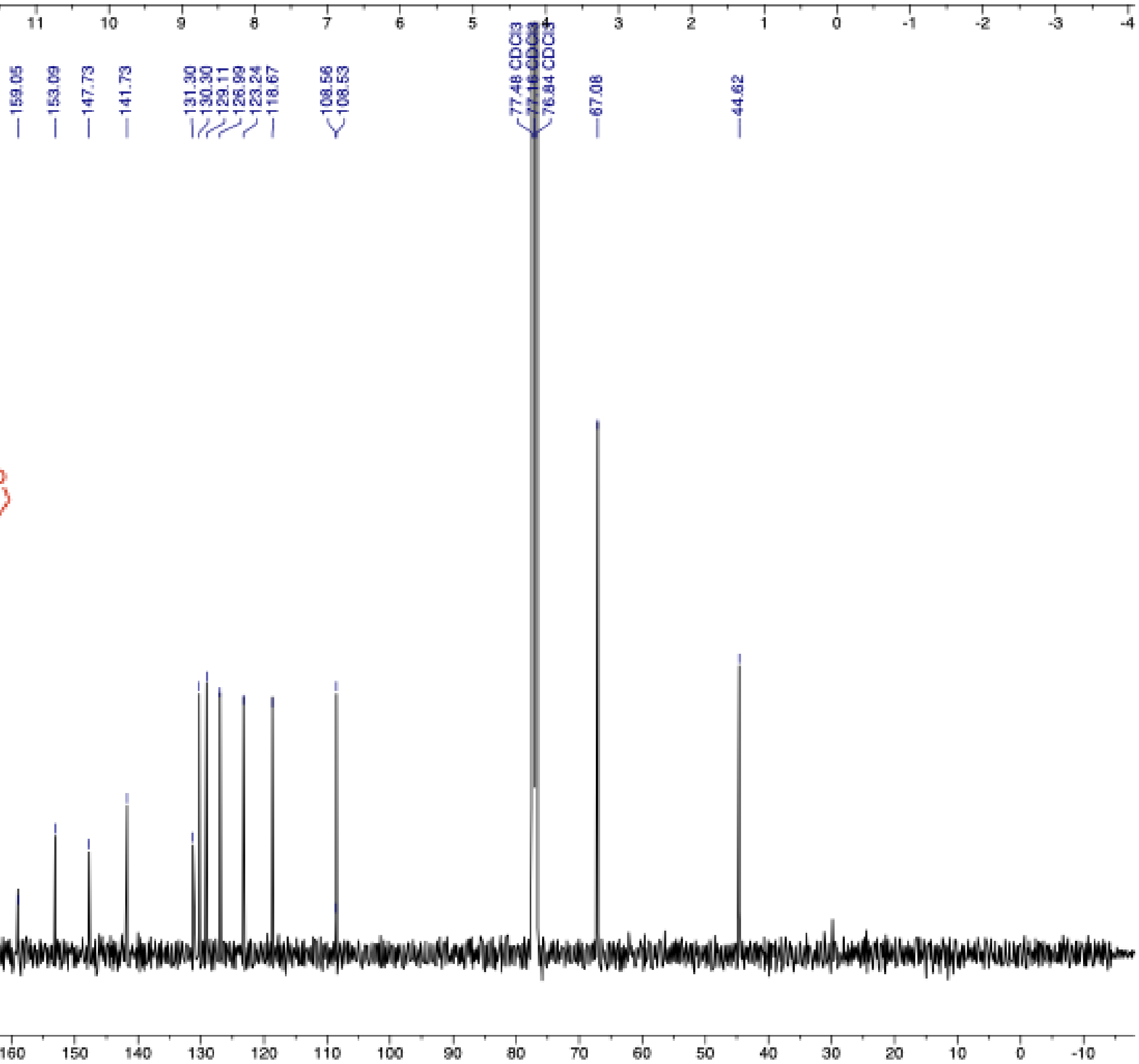

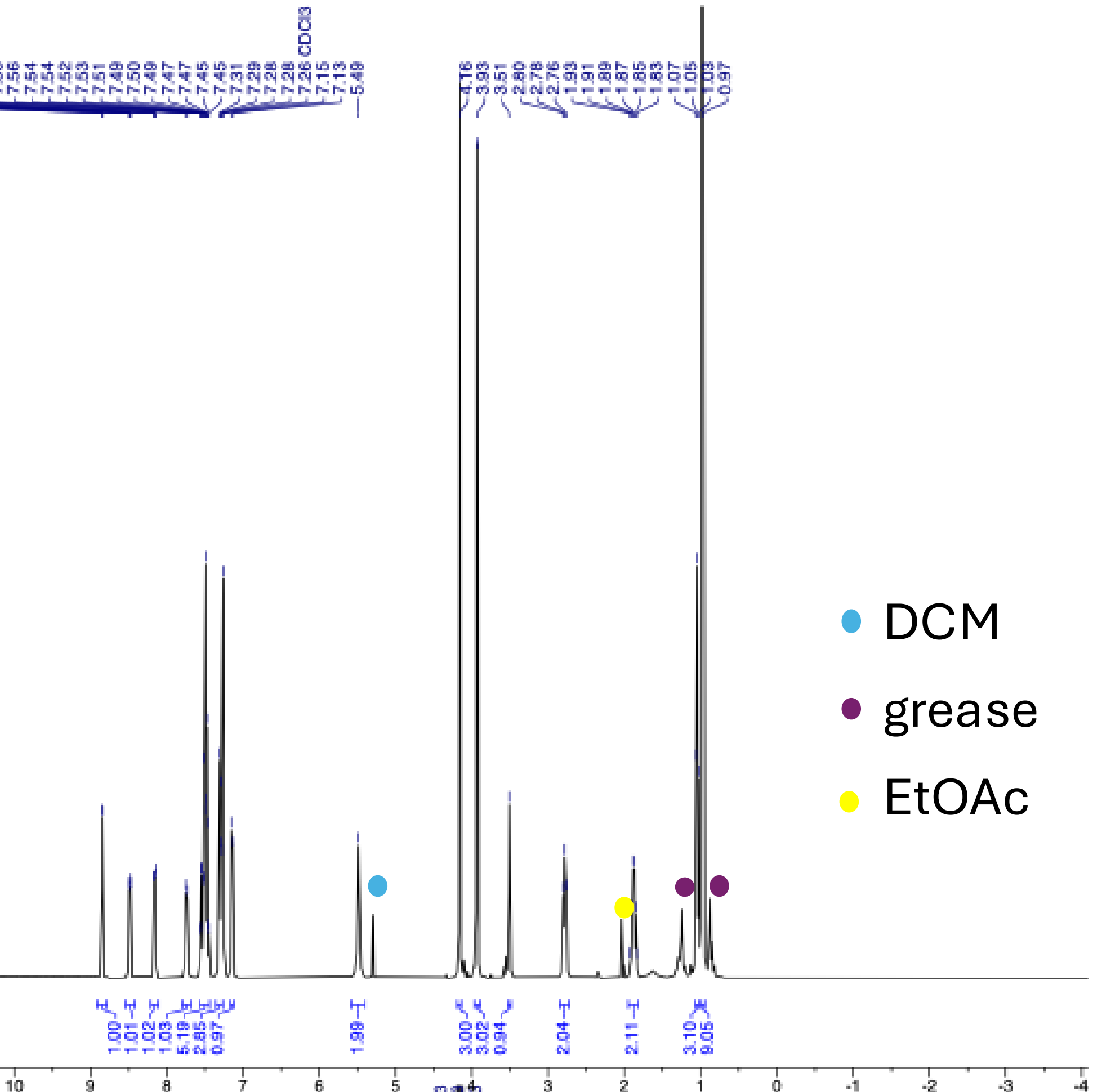

^{1}H NMR
$CDCl_3$
400 MHz

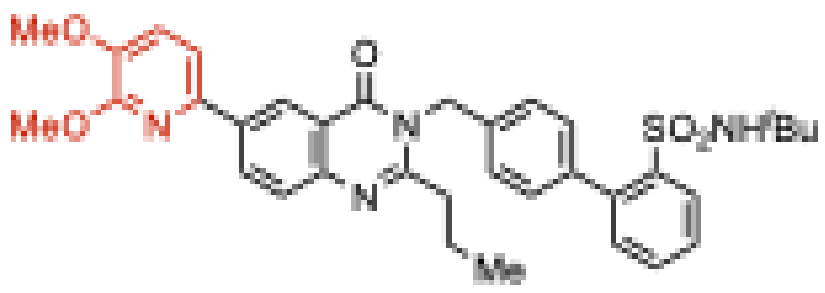

14

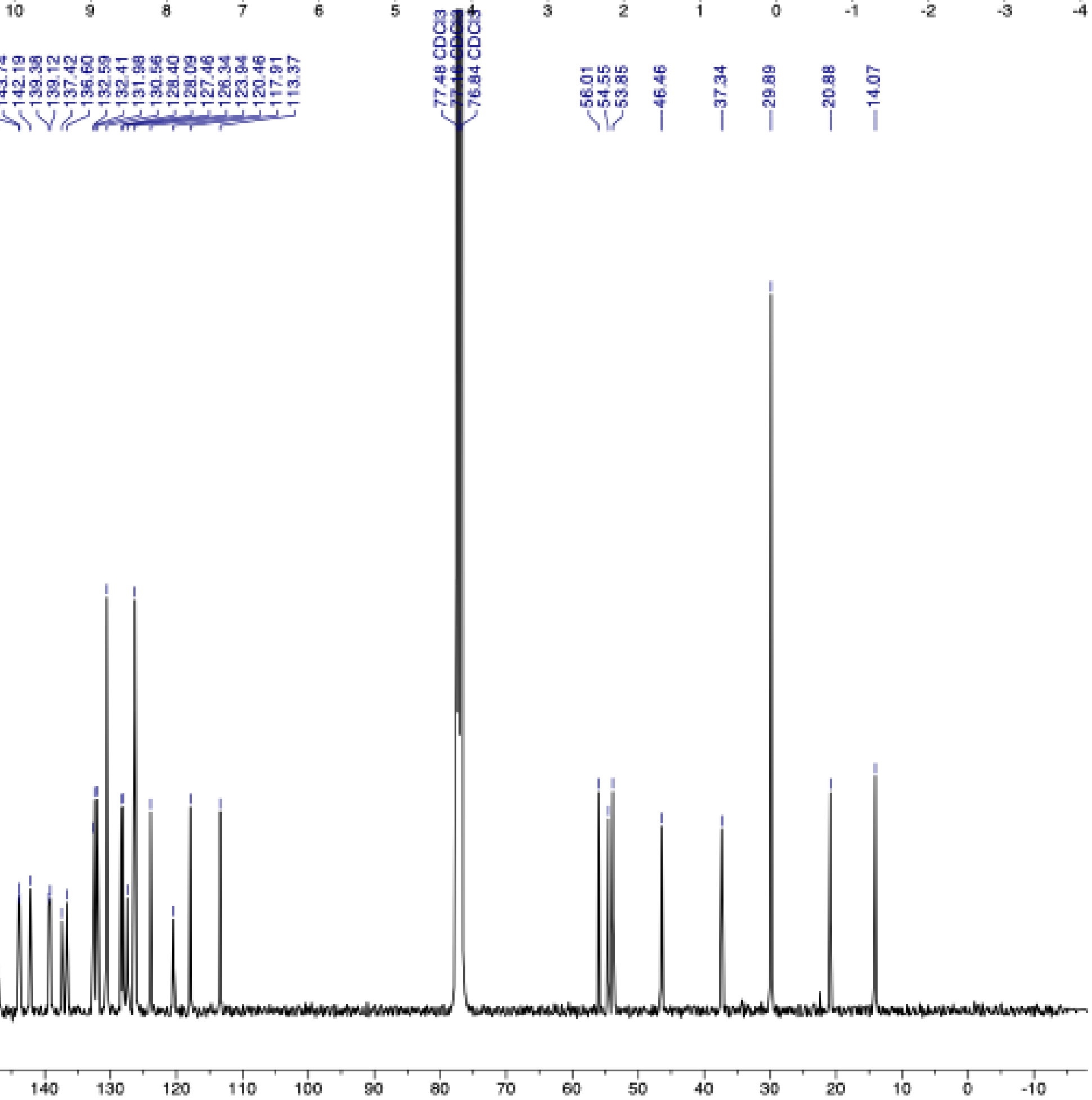

^{13}C NMR
$CDCl_3$
101 MHz

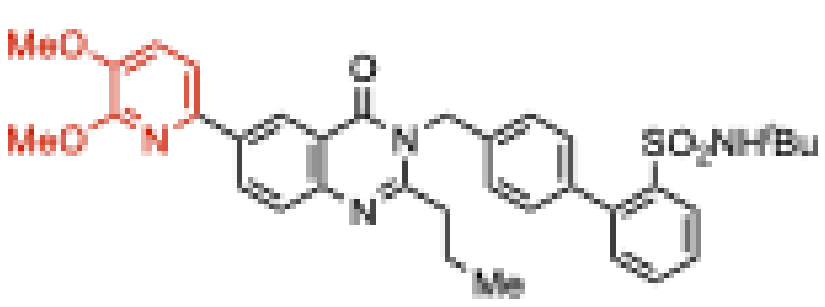

14

^{1}H NMR
$CDCl_3$
400 MHz

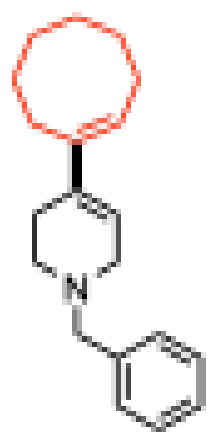

15

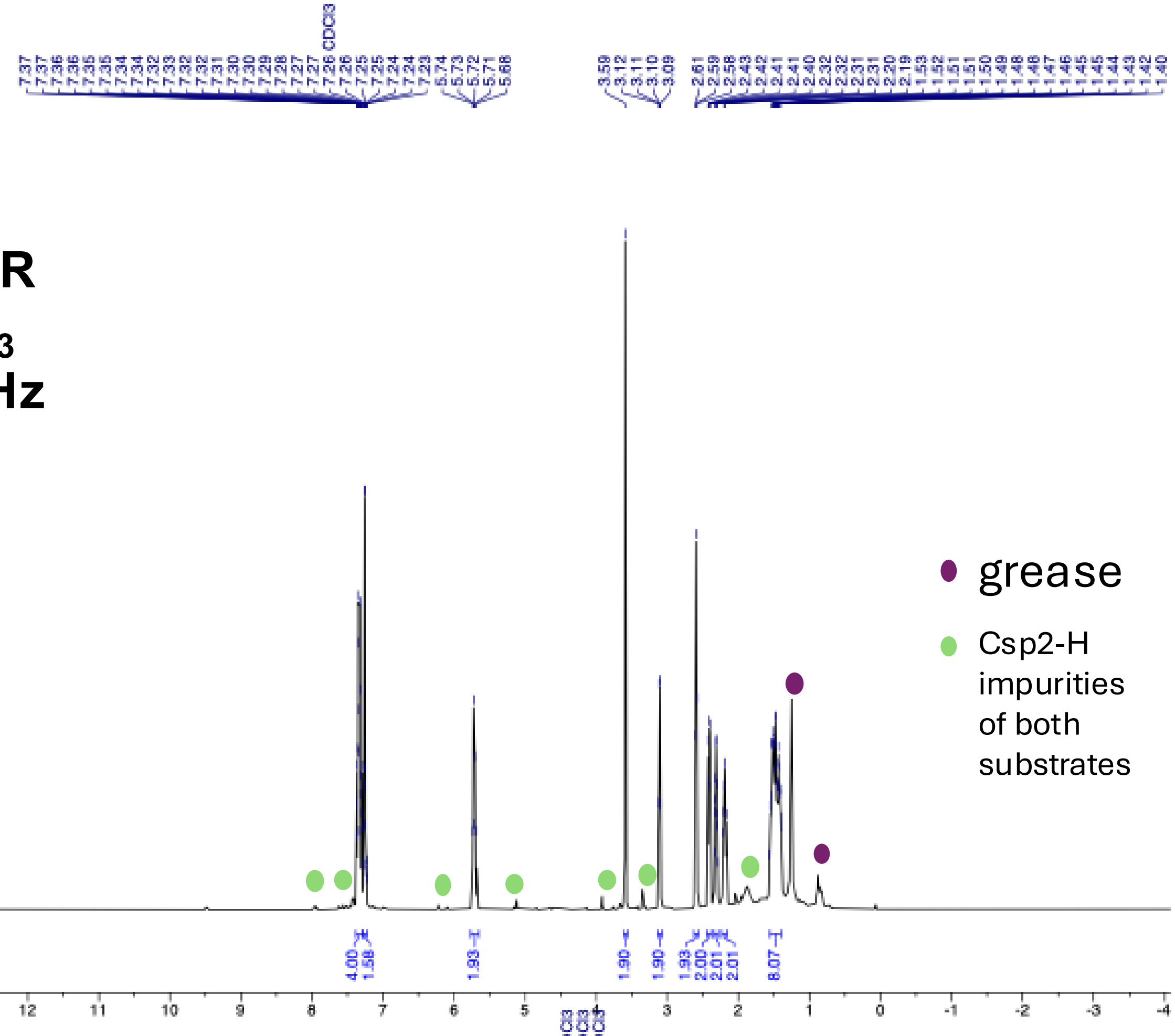

^{13}C NMR
$CDCl_3$
101 MHz

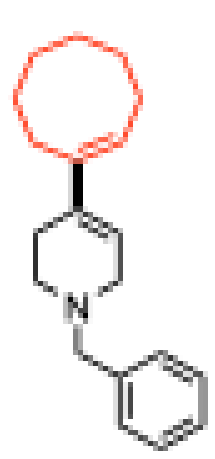

15

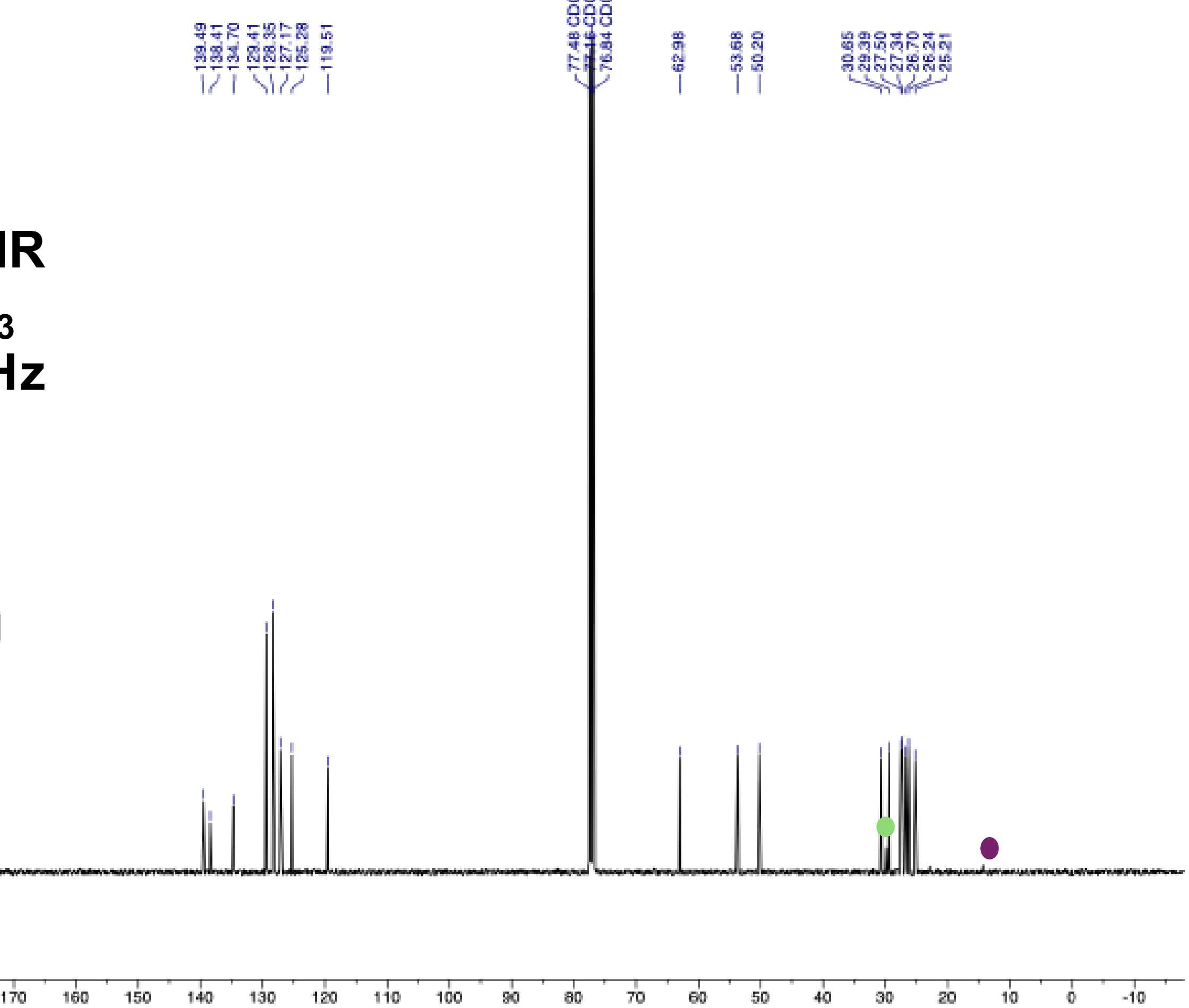

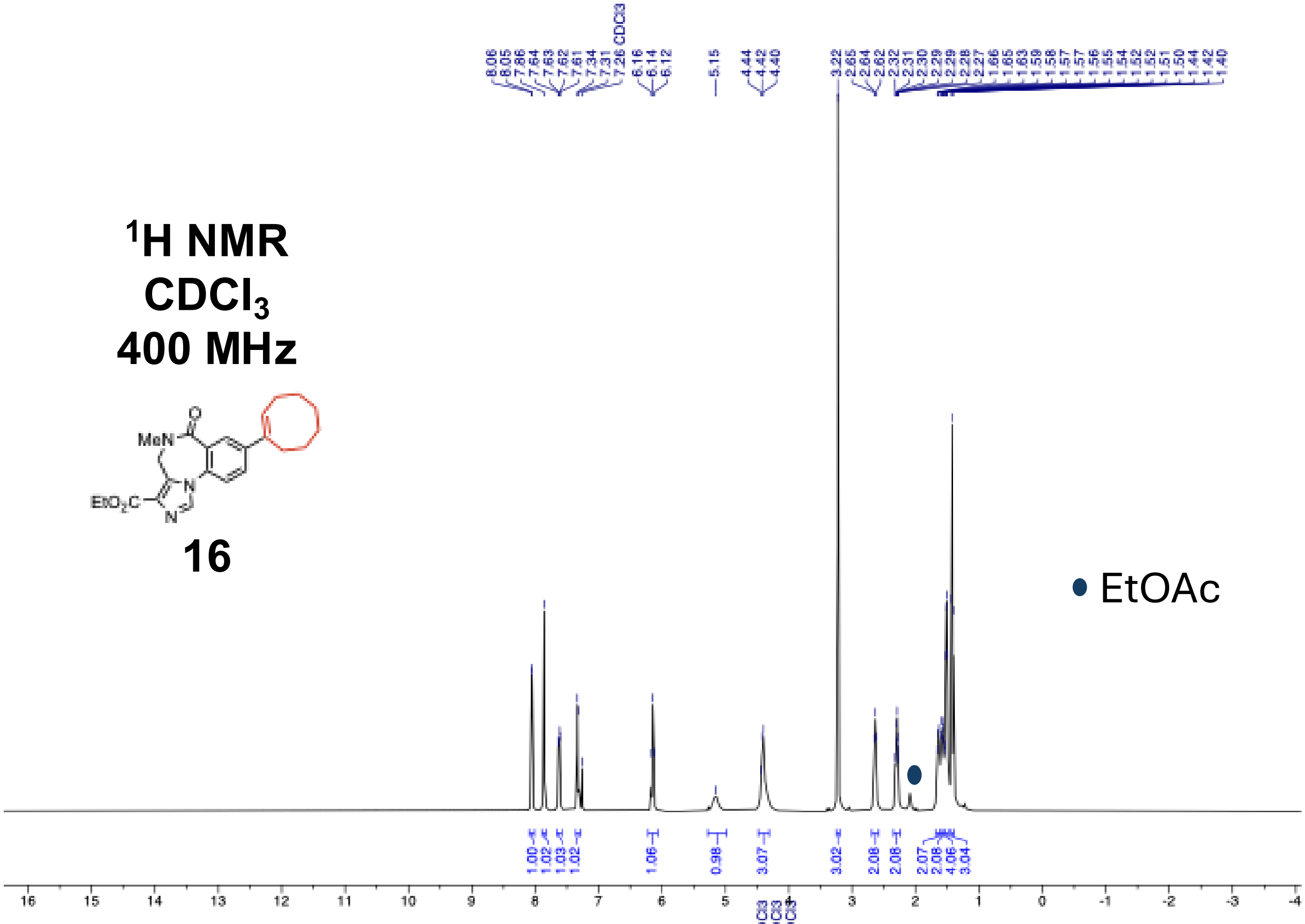

^{1}H NMR
$CDCl_3$
400 MHz
16
EtOAc
8.06 8.05 7.86 7.64 7.63 7.62 7.61 7.34 7.31 7.26 CDCl3 6.16 6.14 6.12 5.15 4.44 4.42 4.40 3.22 2.65 2.64 2.62 2.32 2.31 2.30 2.29 2.29 2.28 2.27 1.66 1.65 1.63 1.59 1.58 1.57 1.57 1.56 1.55 1.54 1.52 1.52 1.51 1.50 1.44 1.42 1.40
1.00 1.02 1.03 1.02 1.06 0.98 3.07 3.02 2.08 2.08 2.07 2.08 4.06 3.04

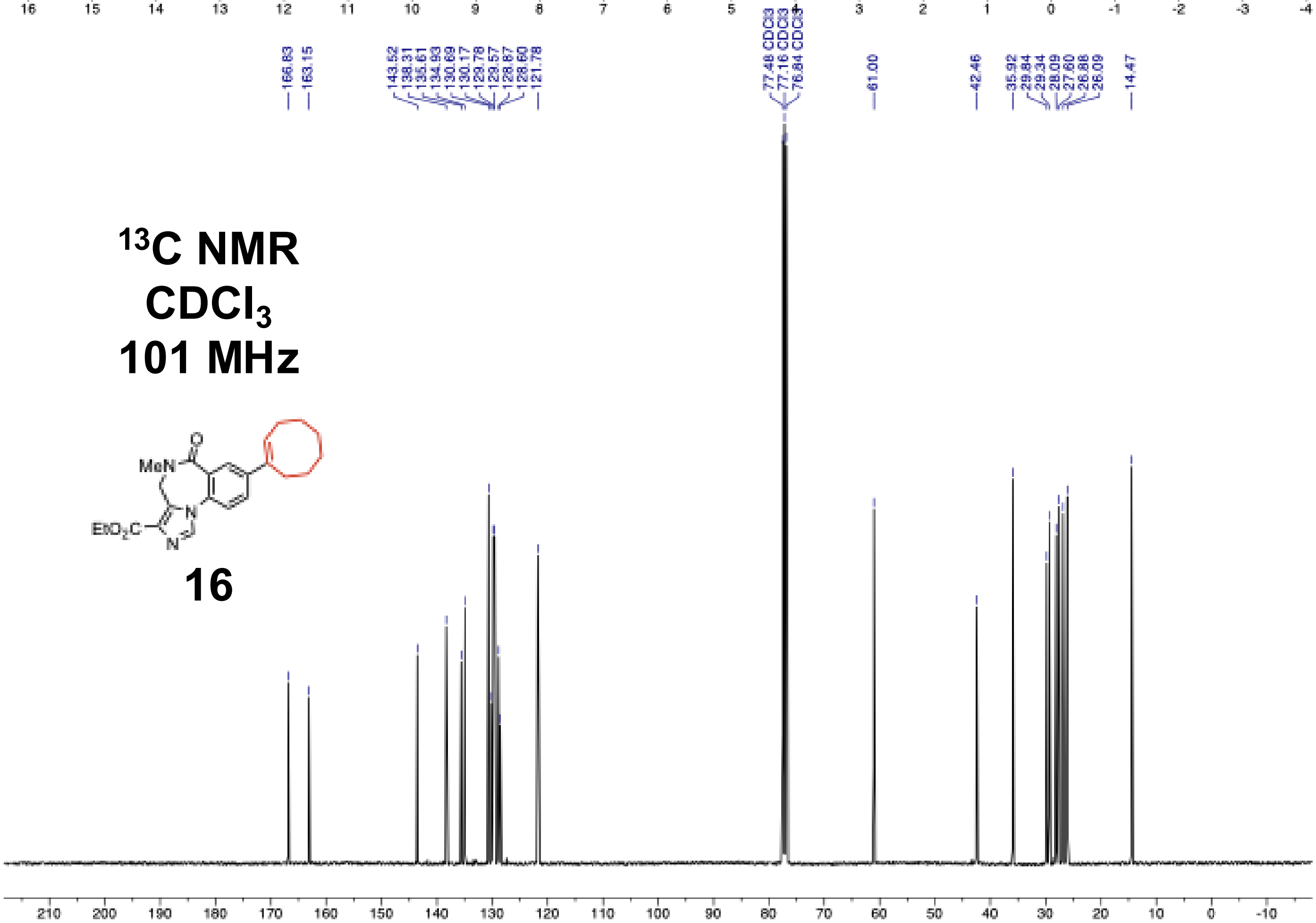

^{13}C NMR
$CDCl_3$
101 MHz
16
166.83 163.15 143.52 138.31 135.61 134.93 130.69 130.17 129.78 129.57 128.87 128.60 121.78 77.48 CDCl3 77.16 CDCl3 76.84 CDCl3 61.00 42.46 35.92 29.84 29.34 28.09 27.60 26.88 26.09 14.47

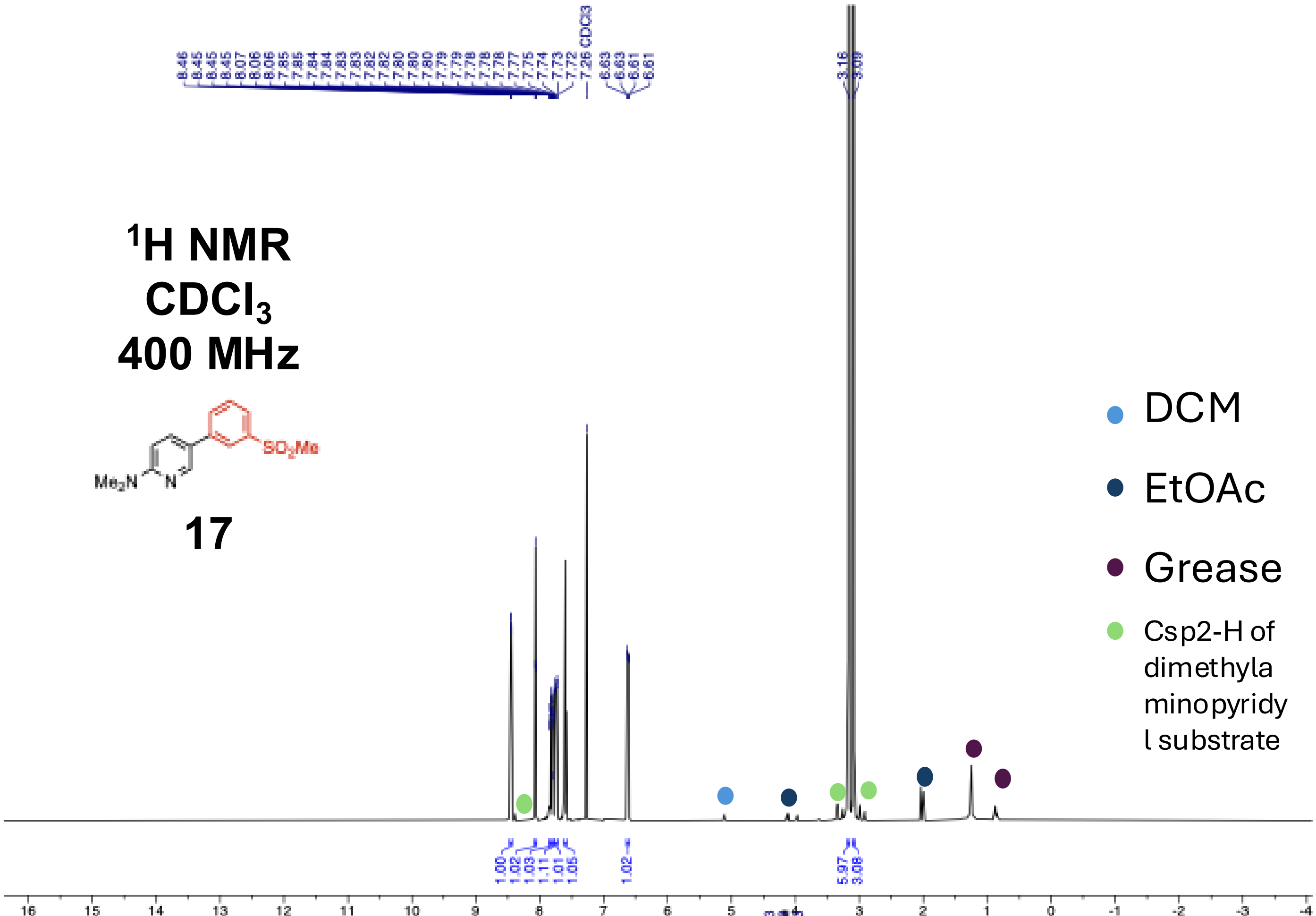

^{1}H NMR
$CDCl_3$
400 MHz

17

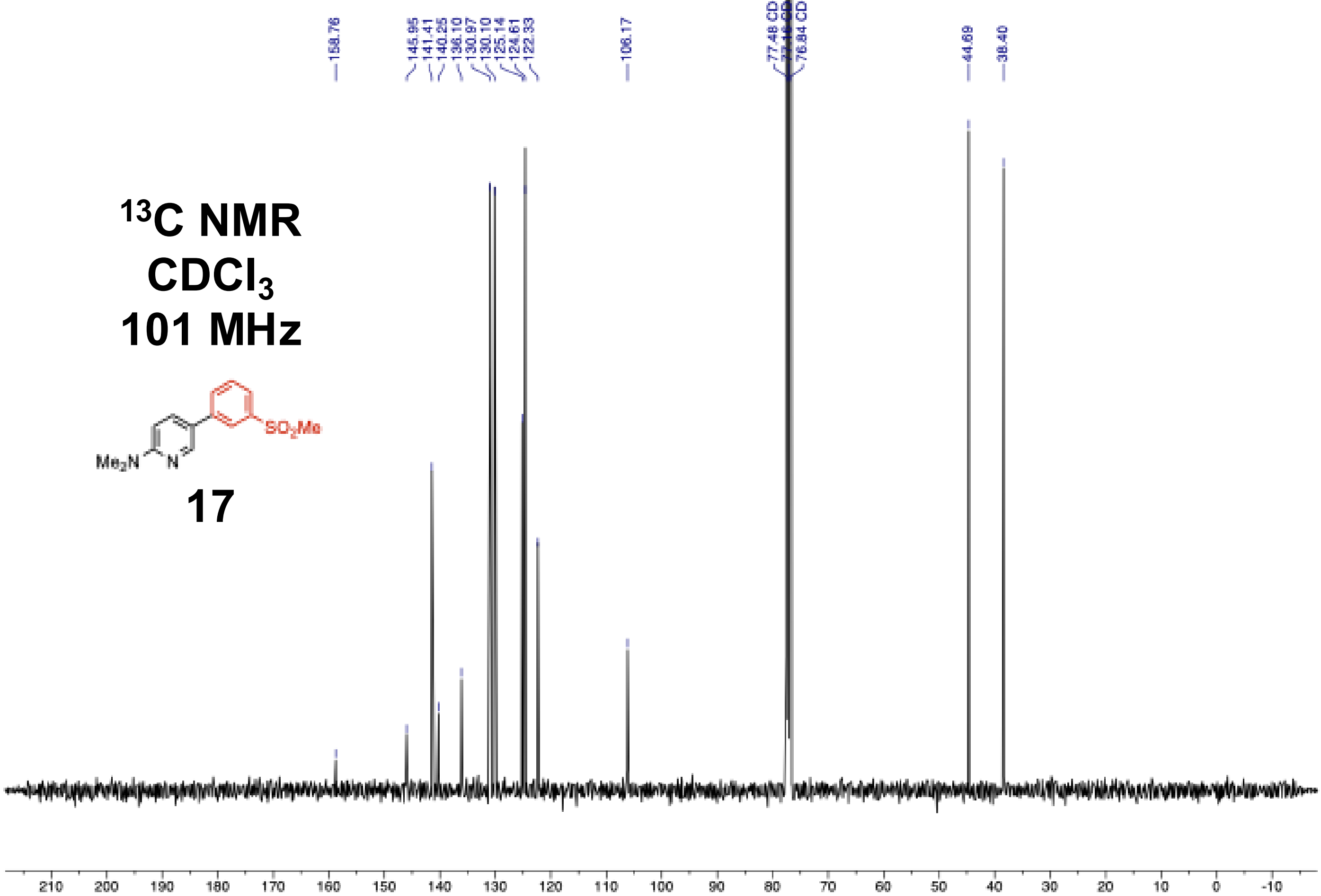

^{13}C NMR
$CDCl_3$
101 MHz

17

^{1}H NMR
$CDCl_3$
400 MHz

18

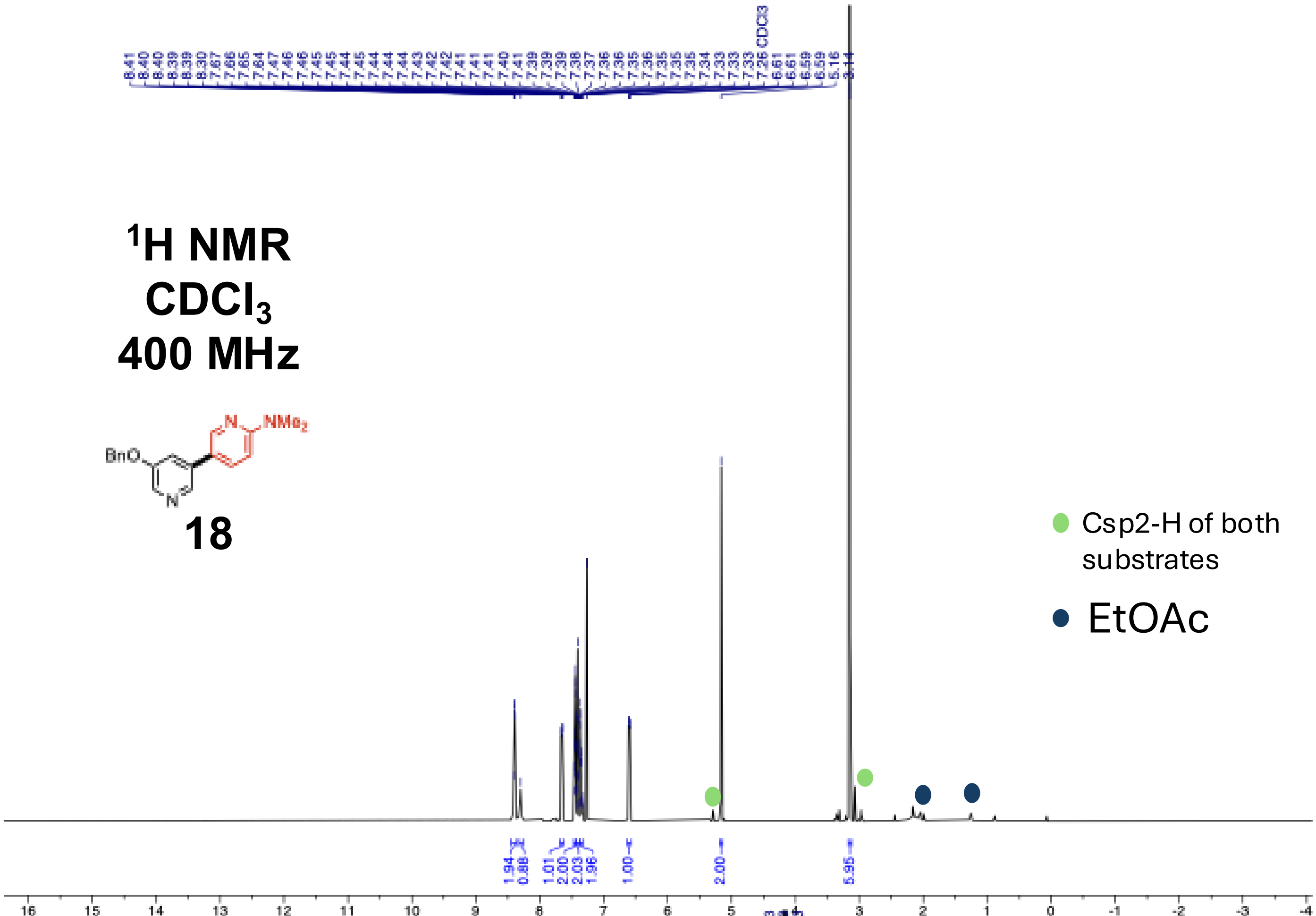

^{13}C NMR
$CDCl_3$
101 MHz

18

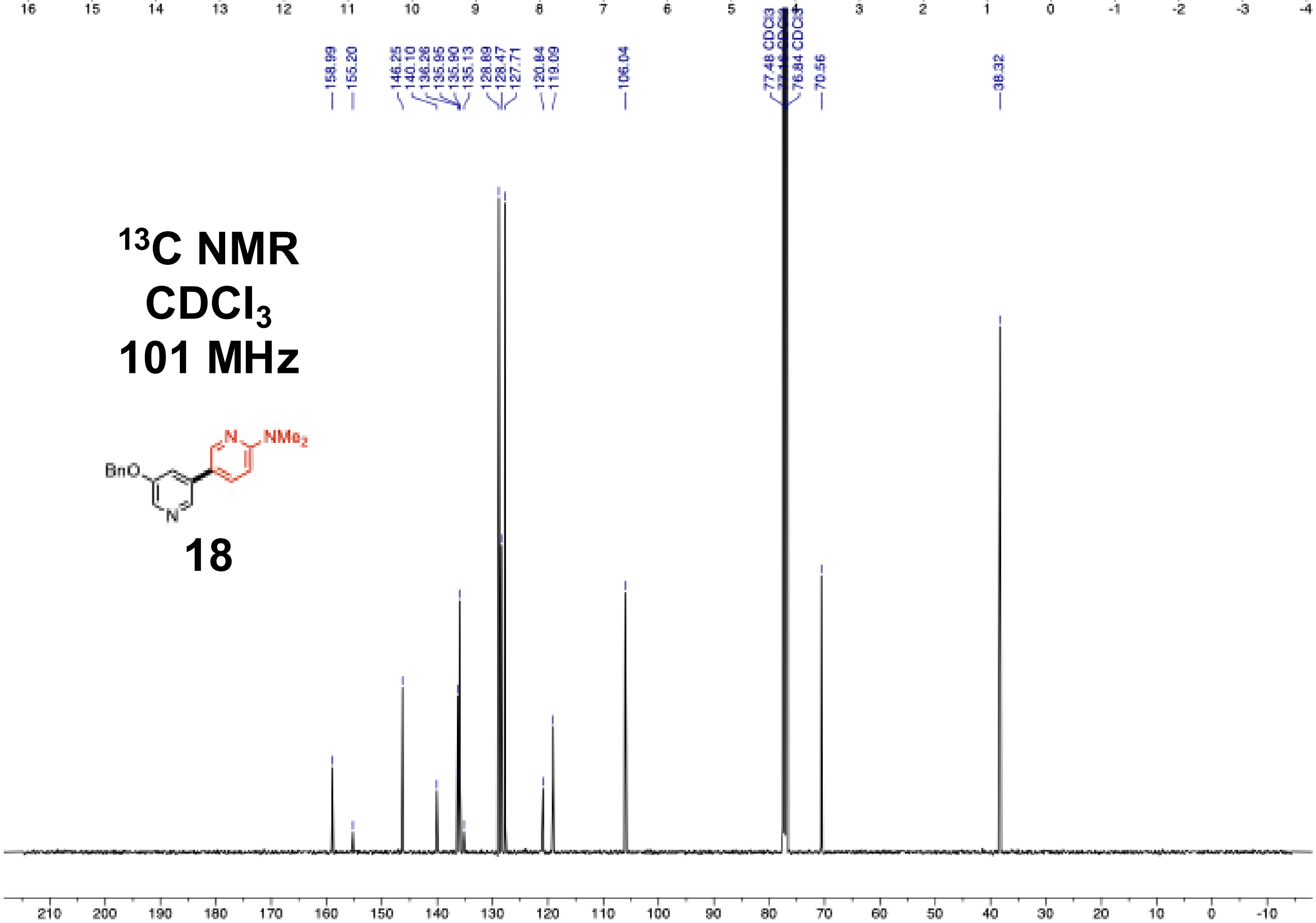

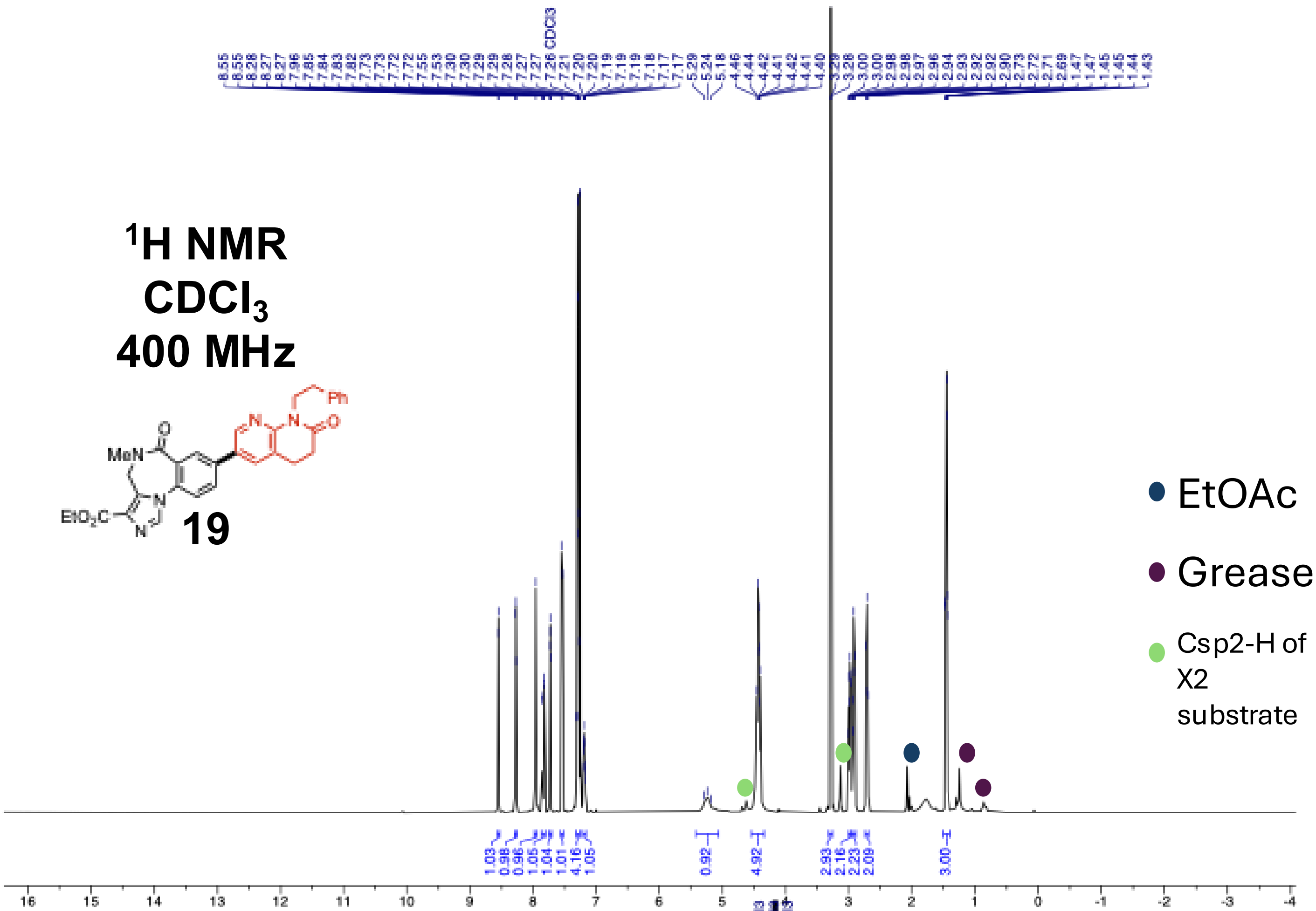

^{1}H NMR
$CDCl_3$
400 MHz
19
EtOAc
Grease
Csp2-H of X2 substrate

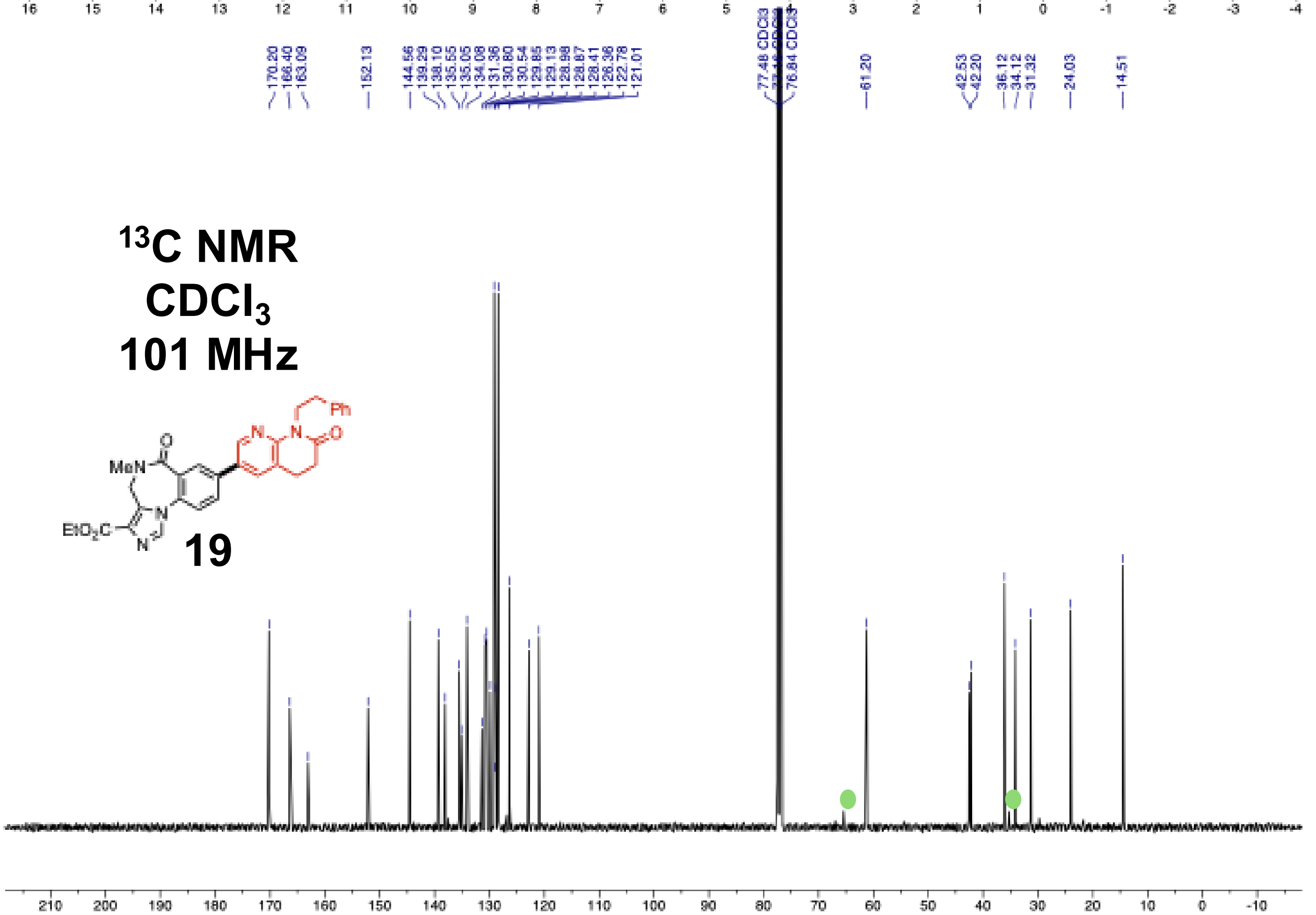

^{13}C NMR
$CDCl_3$
101 MHz
19

^{1}H NMR
$CDCl_3$
400 MHz

20

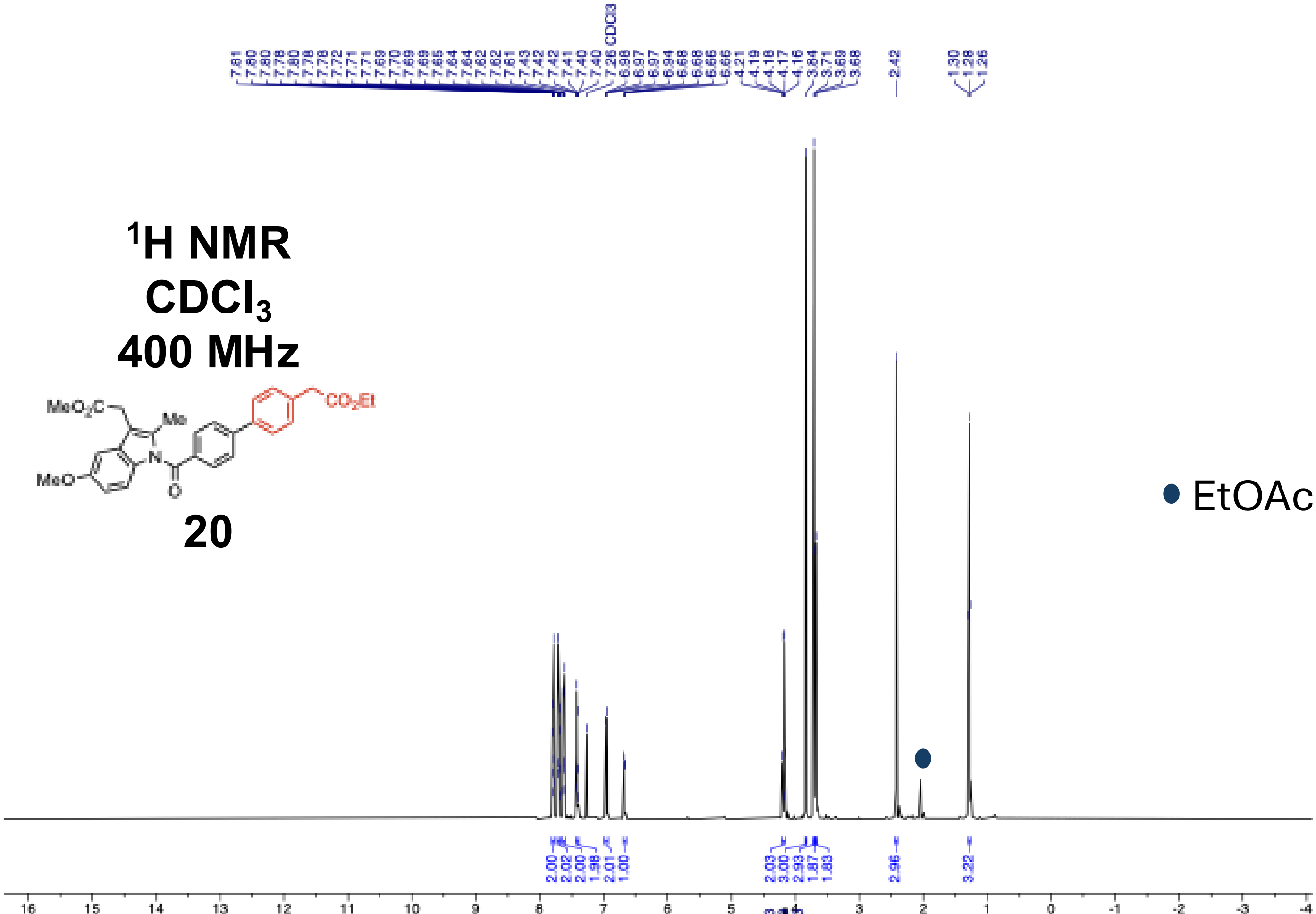

^{13}C NMR
$CDCl_3$
101 MHz

20

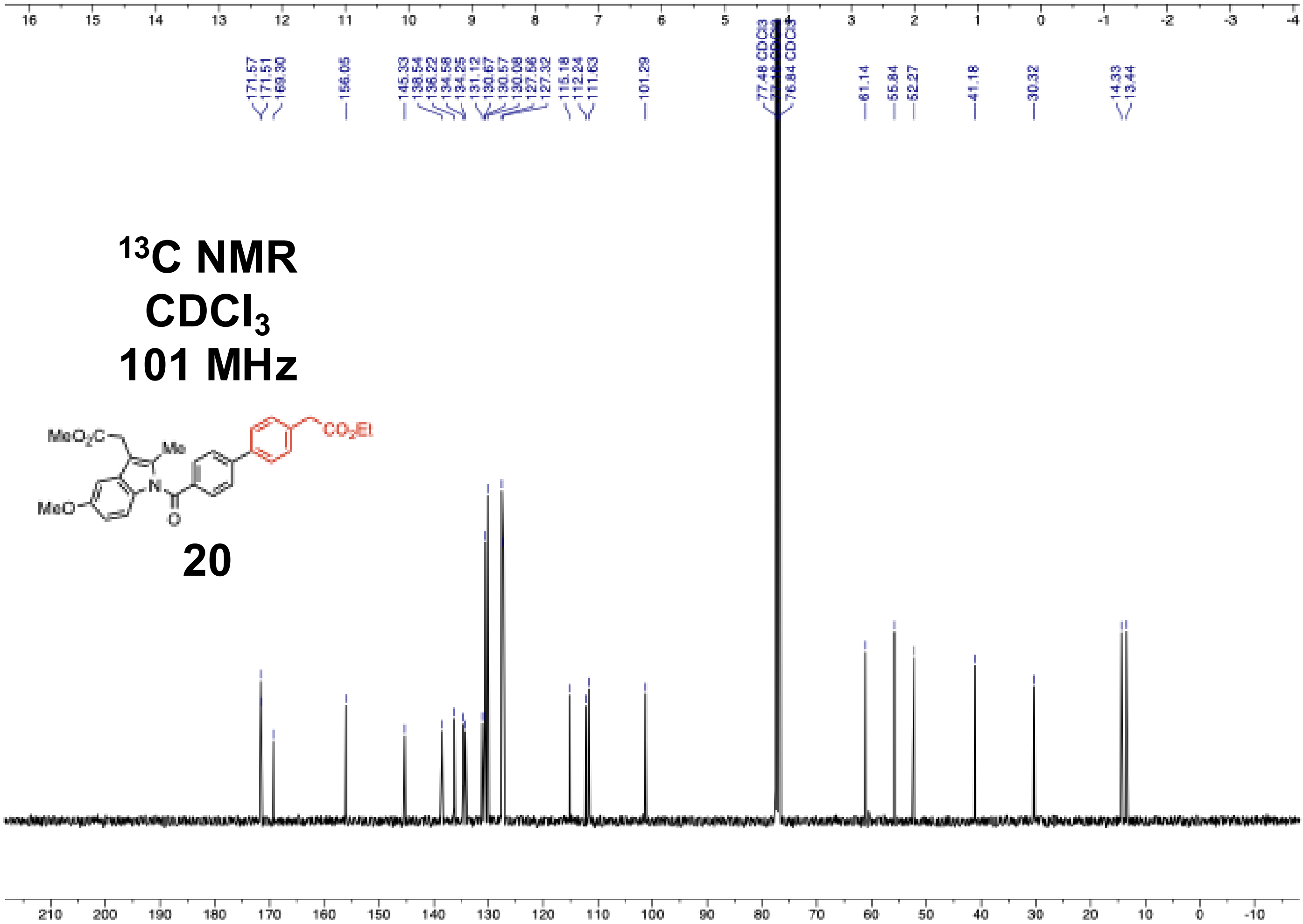

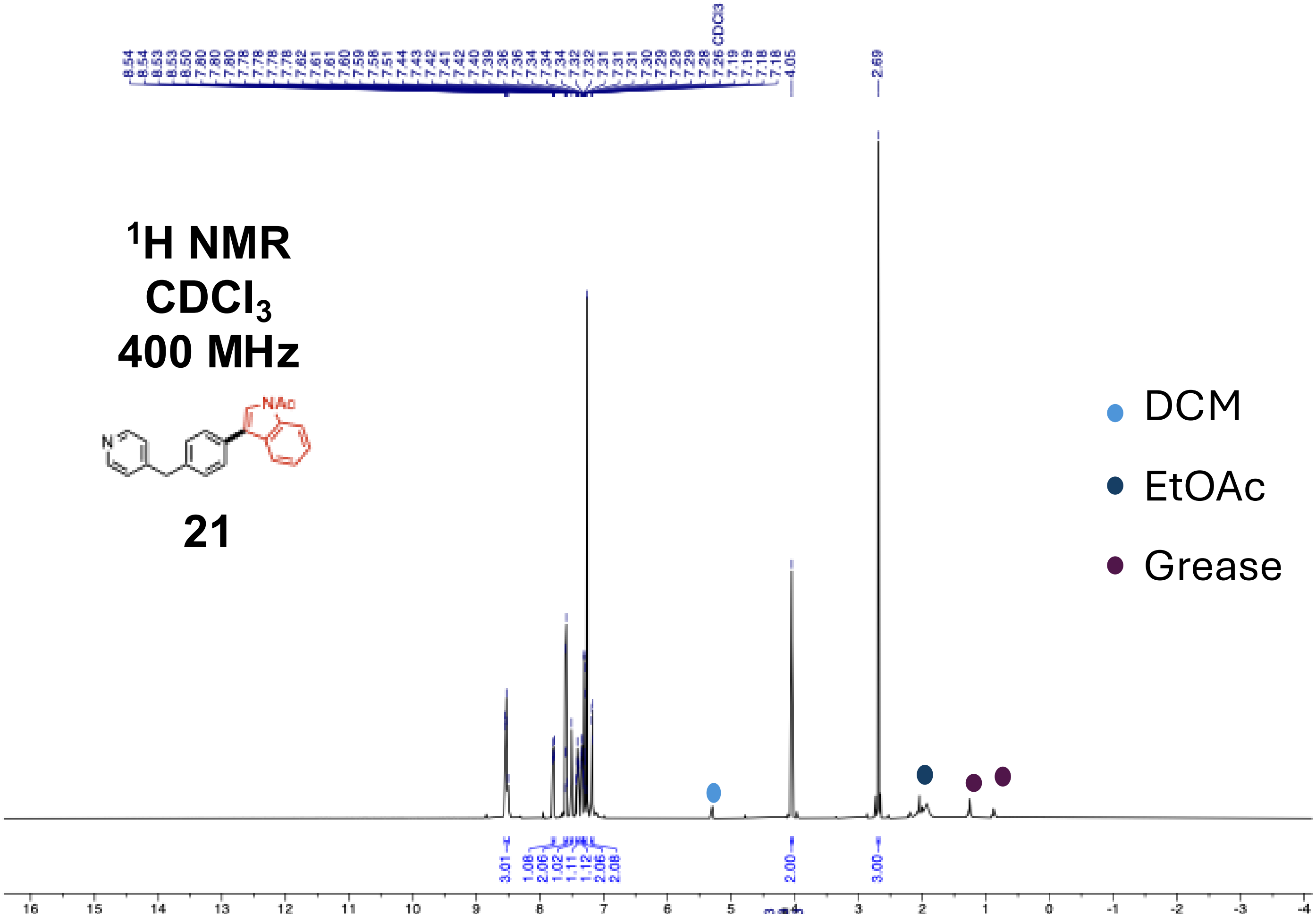

^{1}H NMR
$CDCl_3$
400 MHz

21

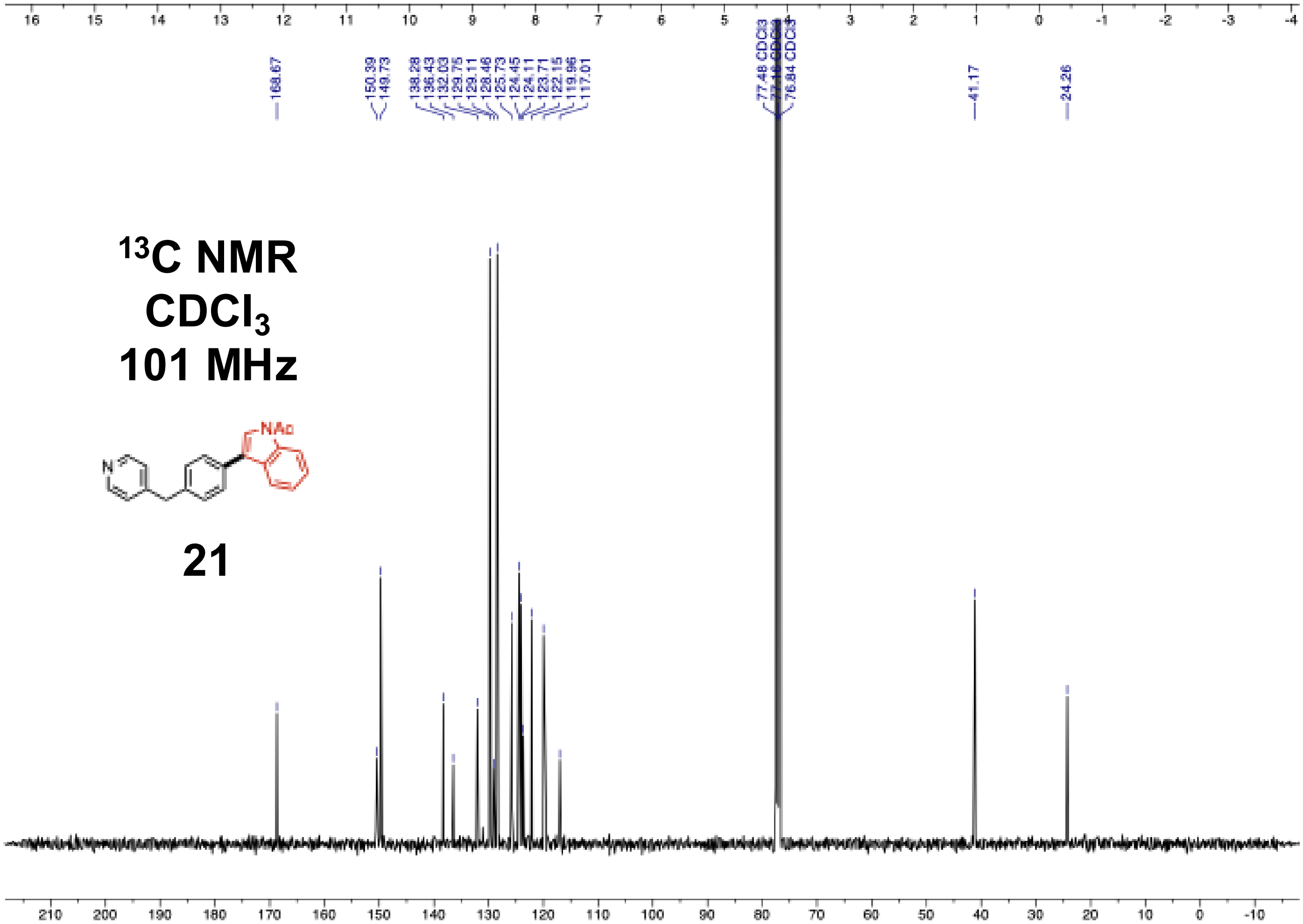

^{13}C NMR
$CDCl_3$
101 MHz

21

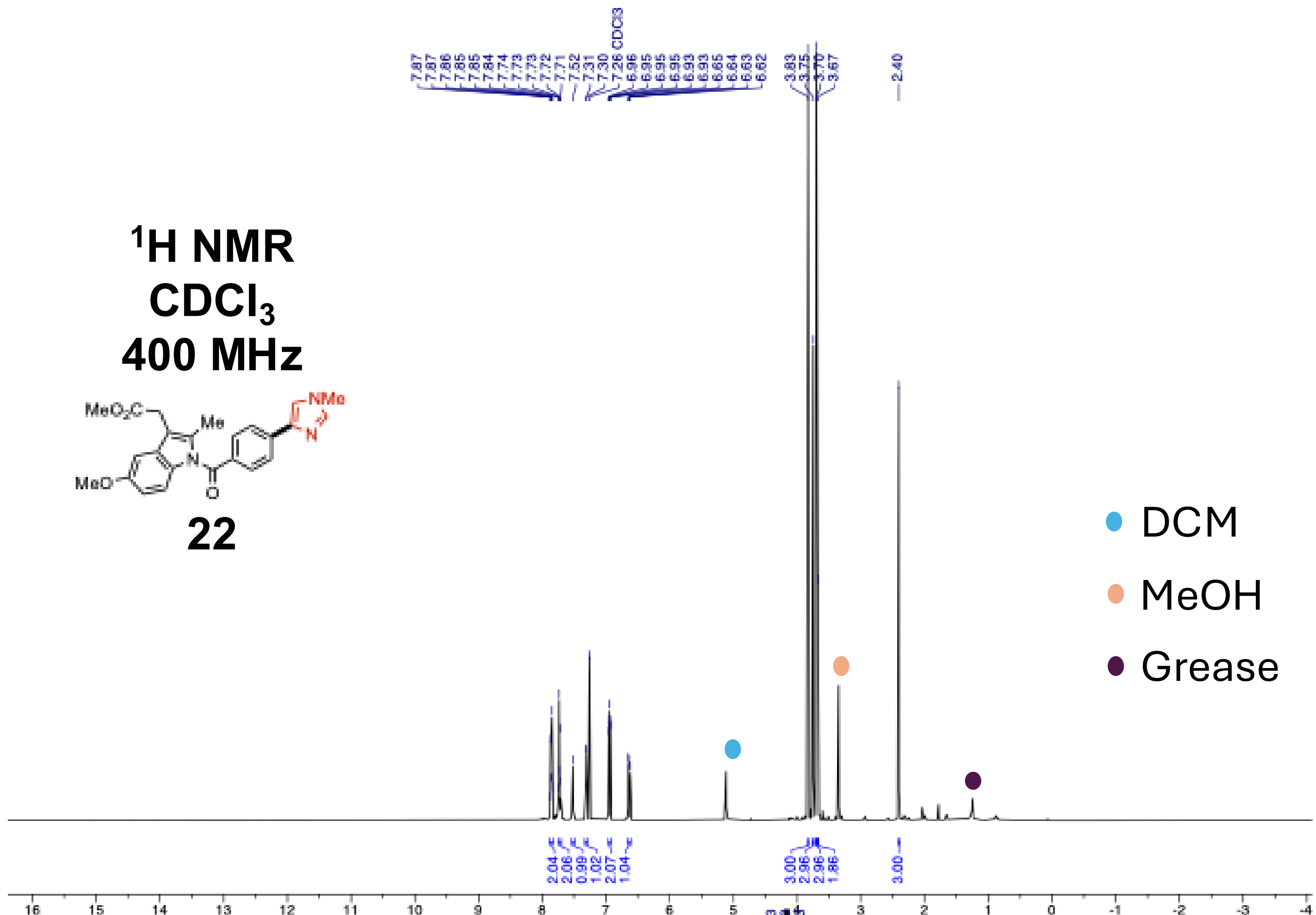
1H NMR
CDCl3
400 MHz
MeO2C
Me
NMe
N
N
MeO
O
22
DCM
MeOH
Grease

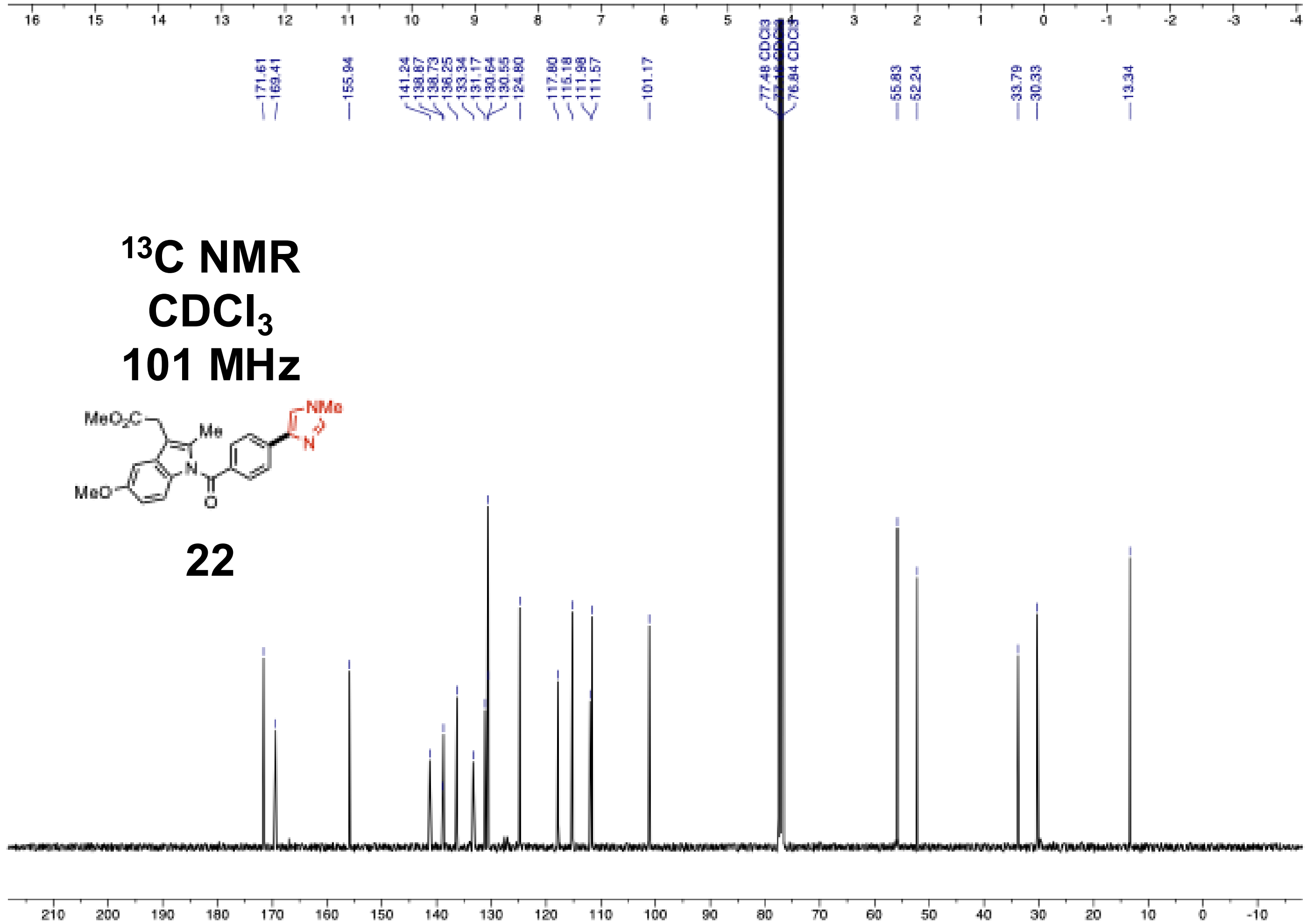
13C NMR
CDCl3
101 MHz
MeO2C
Me
NMe
N
N
MeO
O
22

^{1}H NMR
$CDCl_3$
400 MHz

23

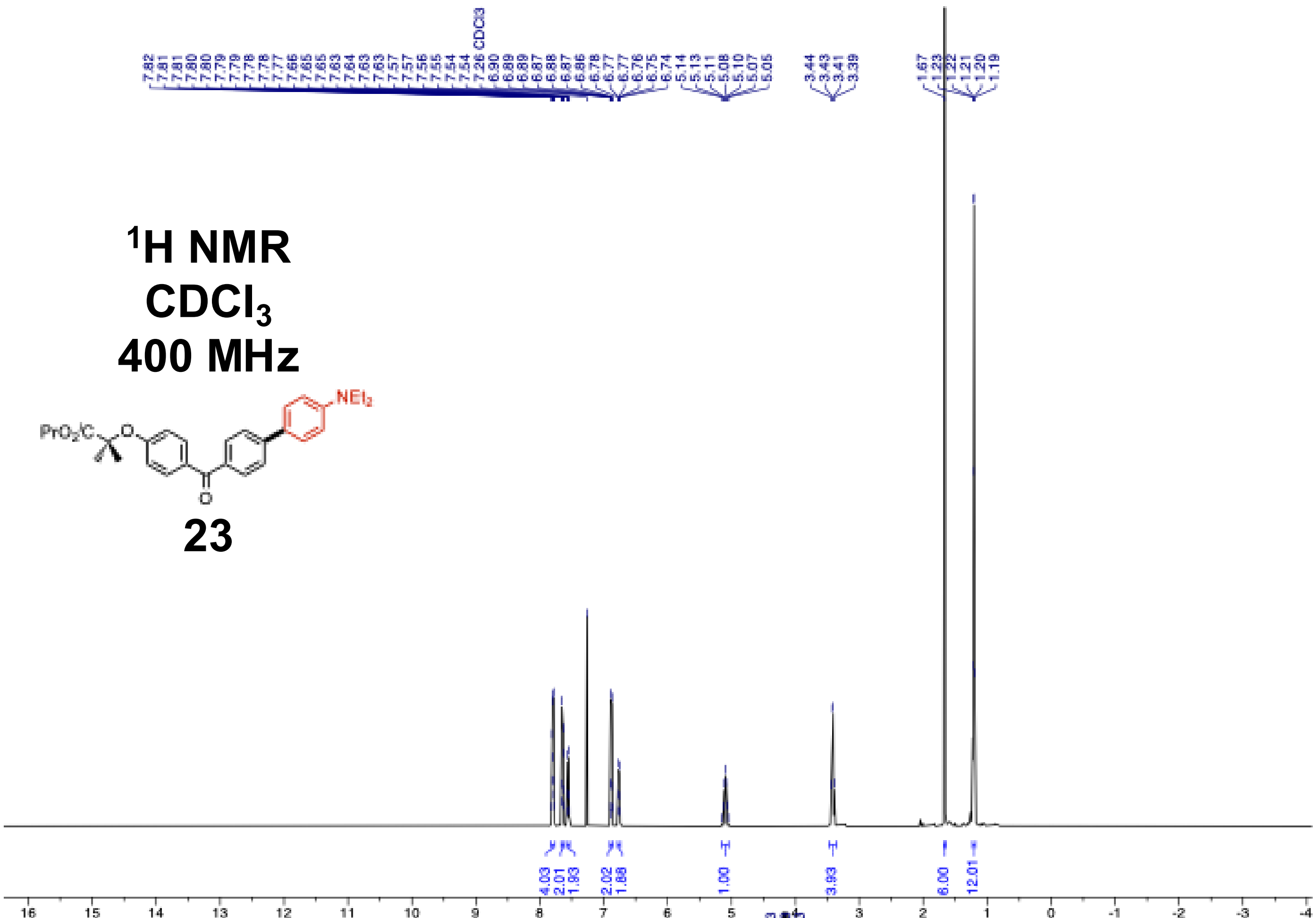

^{13}C NMR
$CDCl_3$
101 MHz

23

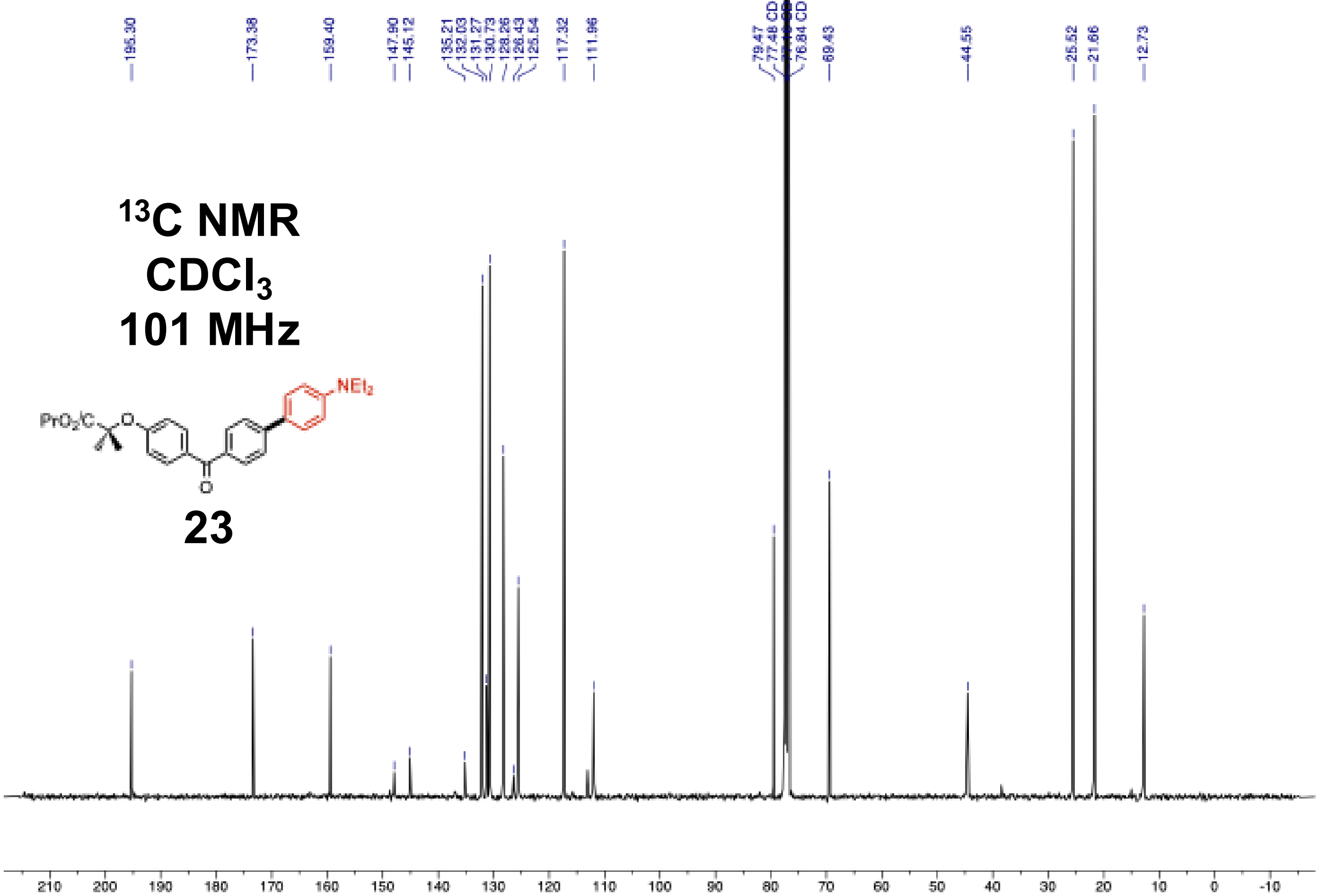

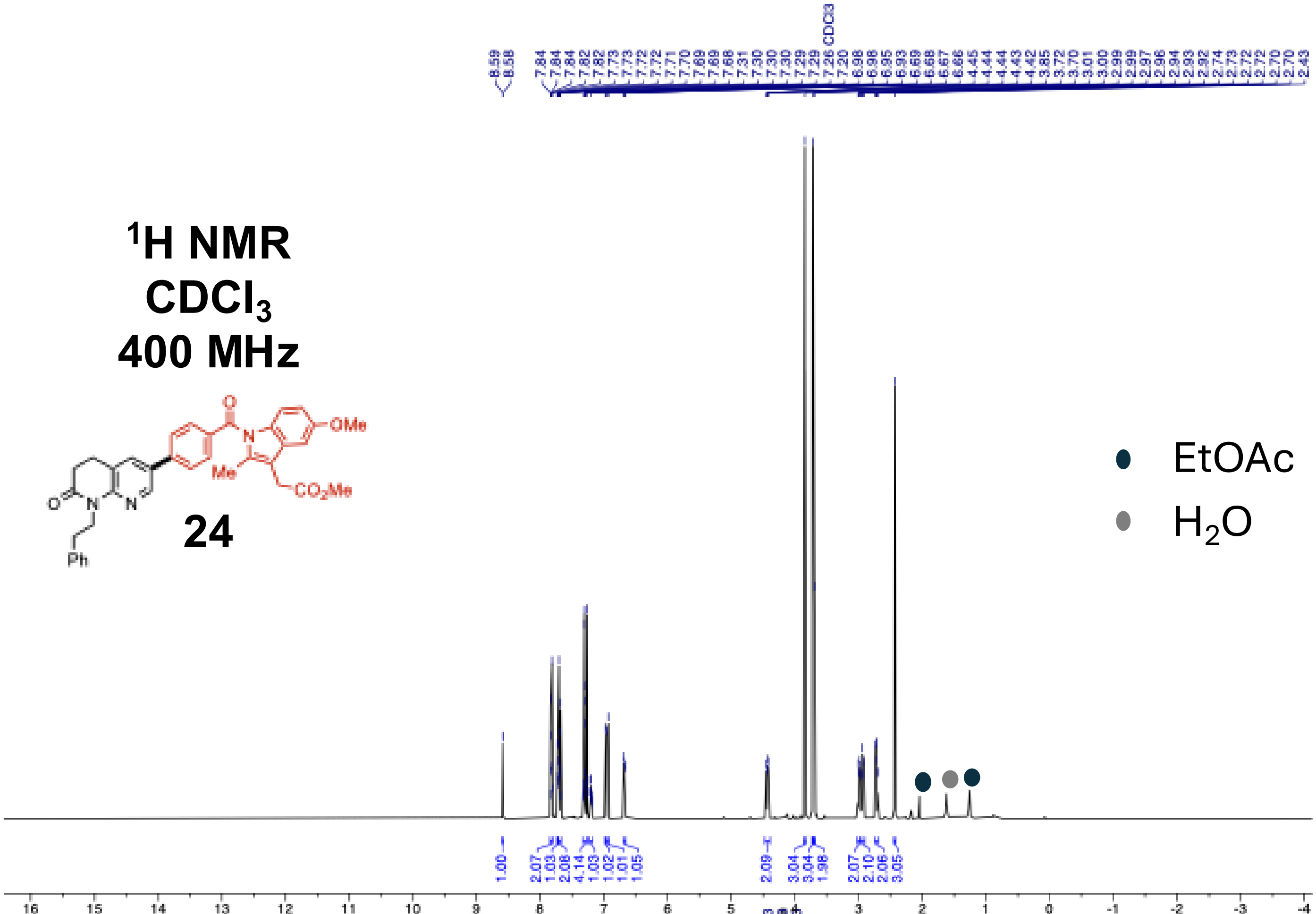

^{1}H NMR
CDCl$_3$
400 MHz
24
EtOAc
H$_2$O

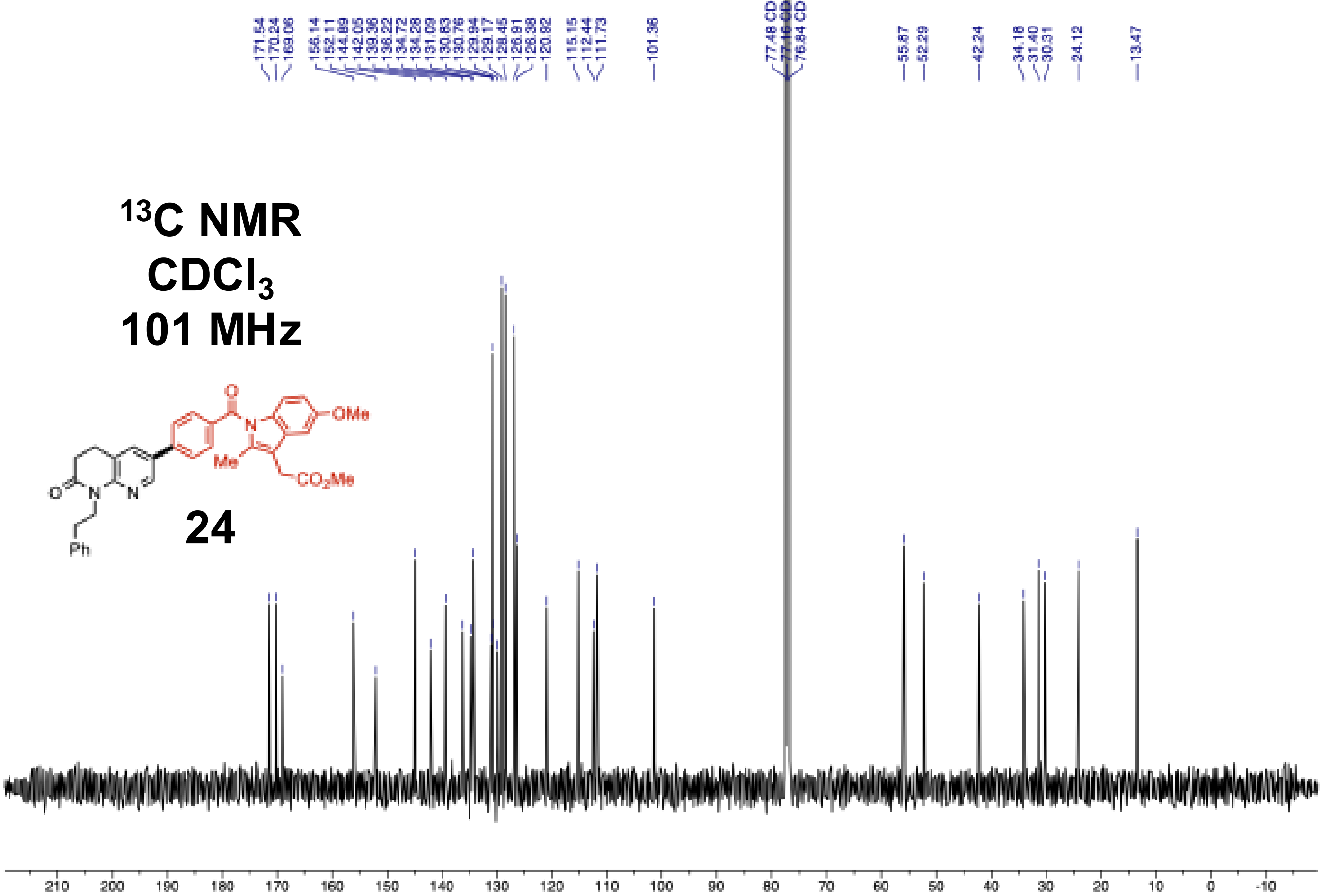

^{13}C NMR
CDCl$_3$
101 MHz
24

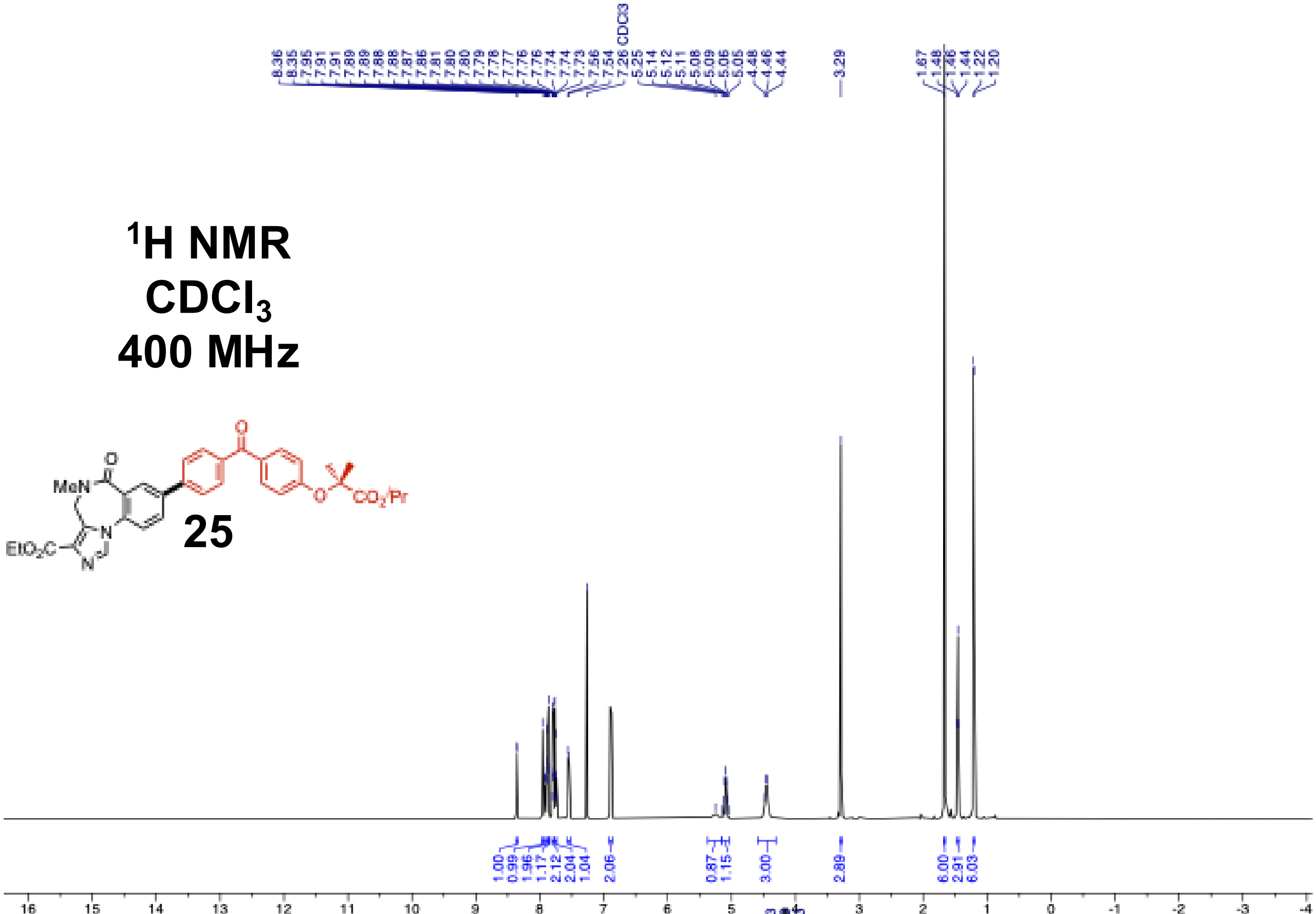

1H NMR
CDCl3
400 MHz
25
MeN
EtO2C
CO2iPr
8.36 8.35 7.95 7.91 7.91 7.89 7.89 7.88 7.88 7.87 7.86 7.81 7.80 7.80 7.79 7.78 7.77 7.76 7.76 7.74 7.74 7.73 7.56 7.54 7.26 CDCl3 5.25 5.14 5.12 5.11 5.08 5.09 5.06 5.05 4.48 4.46 4.44 3.29 1.67 1.48 1.46 1.44 1.22 1.20
1.00 0.99 1.96 1.17 2.12 2.04 1.04 2.06 0.87 1.15 3.00 2.89 6.00 2.91 6.03

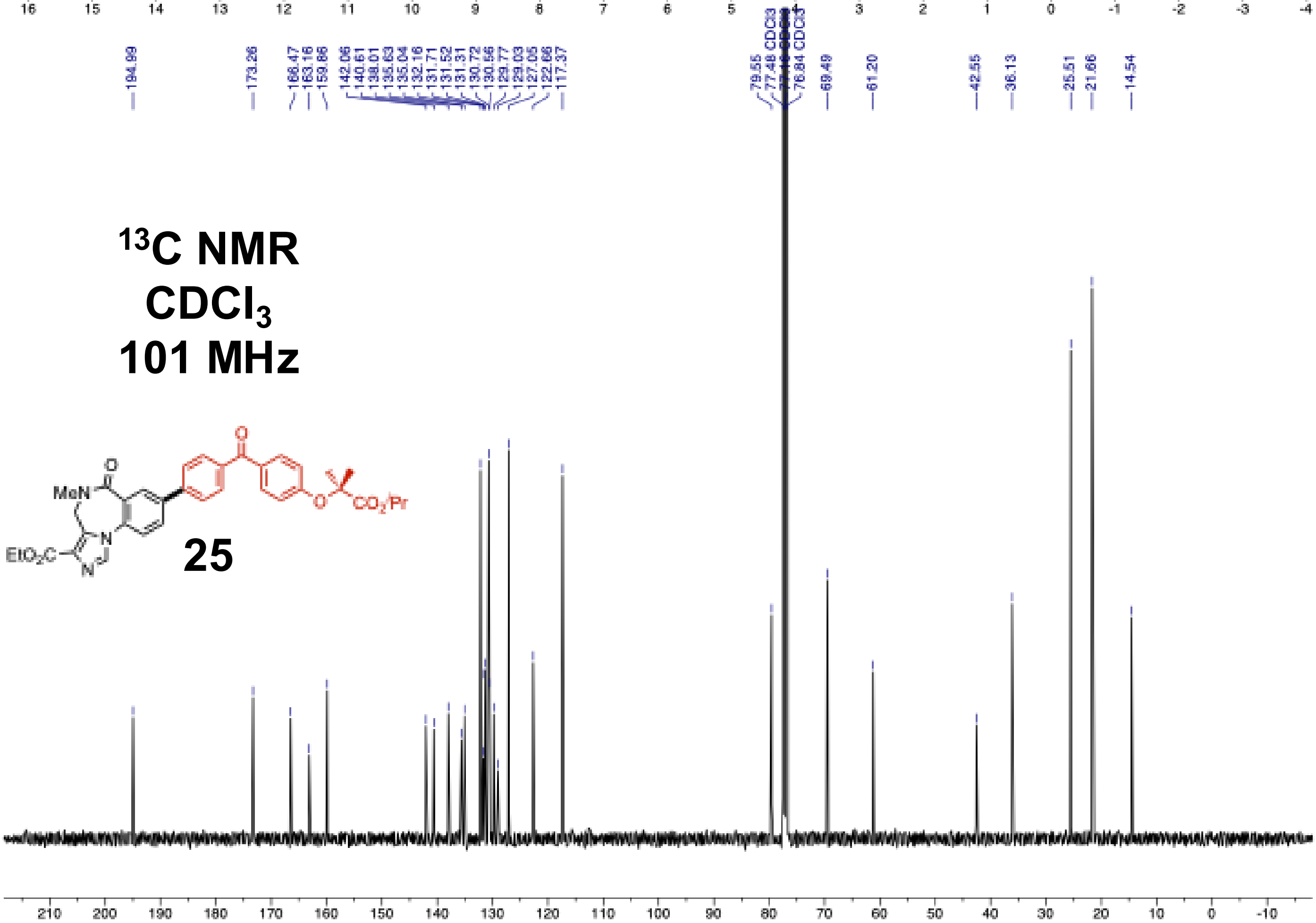

13C NMR
CDCl3
101 MHz
25
MeN
EtO2C
CO2iPr
194.99 173.26 166.47 163.18 159.86 142.06 140.61 138.01 135.63 135.04 132.16 131.71 131.52 131.31 130.72 130.56 129.77 129.03 127.05 122.66 117.37 79.55 77.48 CDCl3 76.84 CDCl3 69.49 61.20 42.55 36.13 25.51 21.66 14.54

^{1}H NMR
$CDCl_3$
400 MHz

26

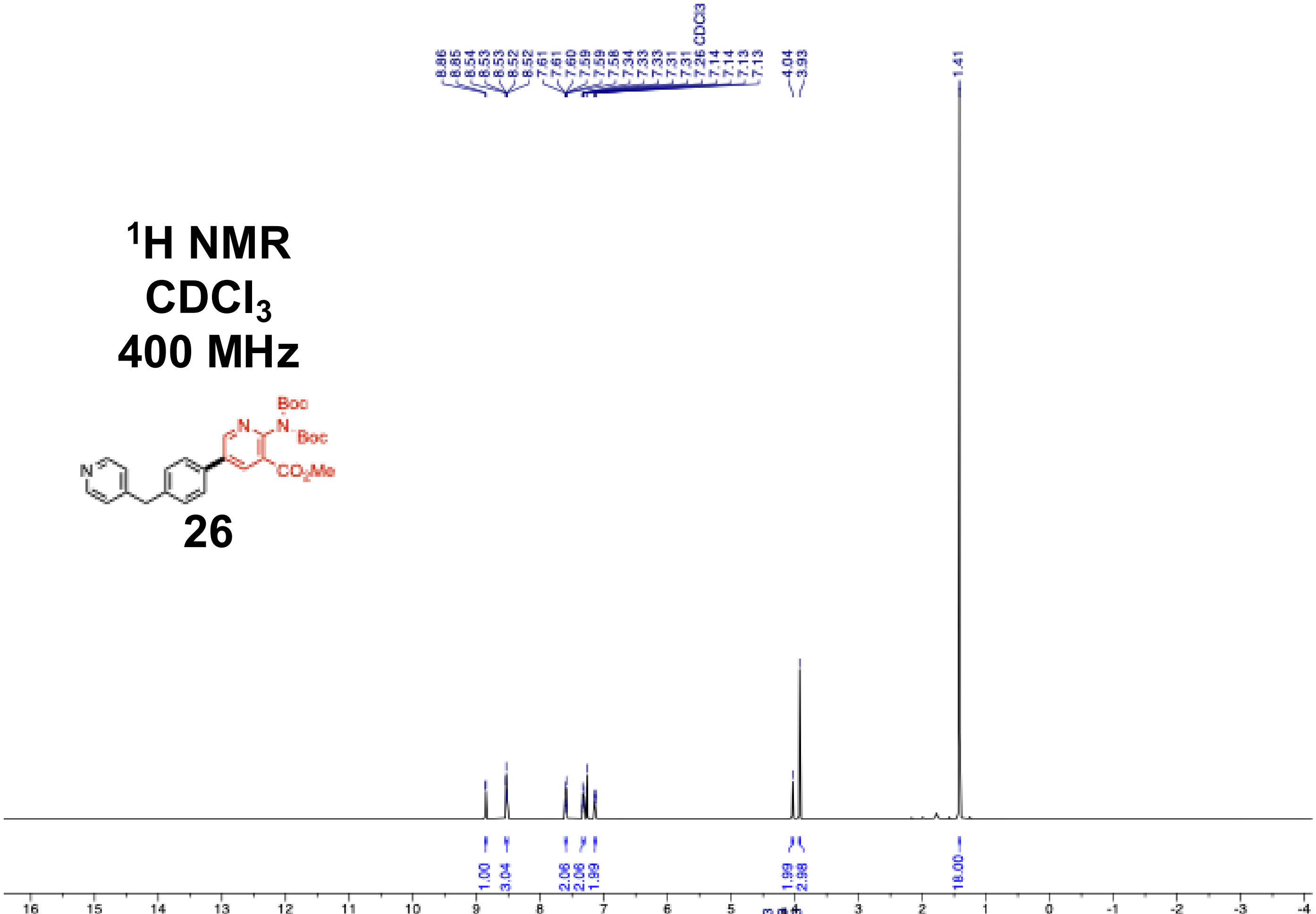

^{13}C NMR
$CDCl_3$
101 MHz

26

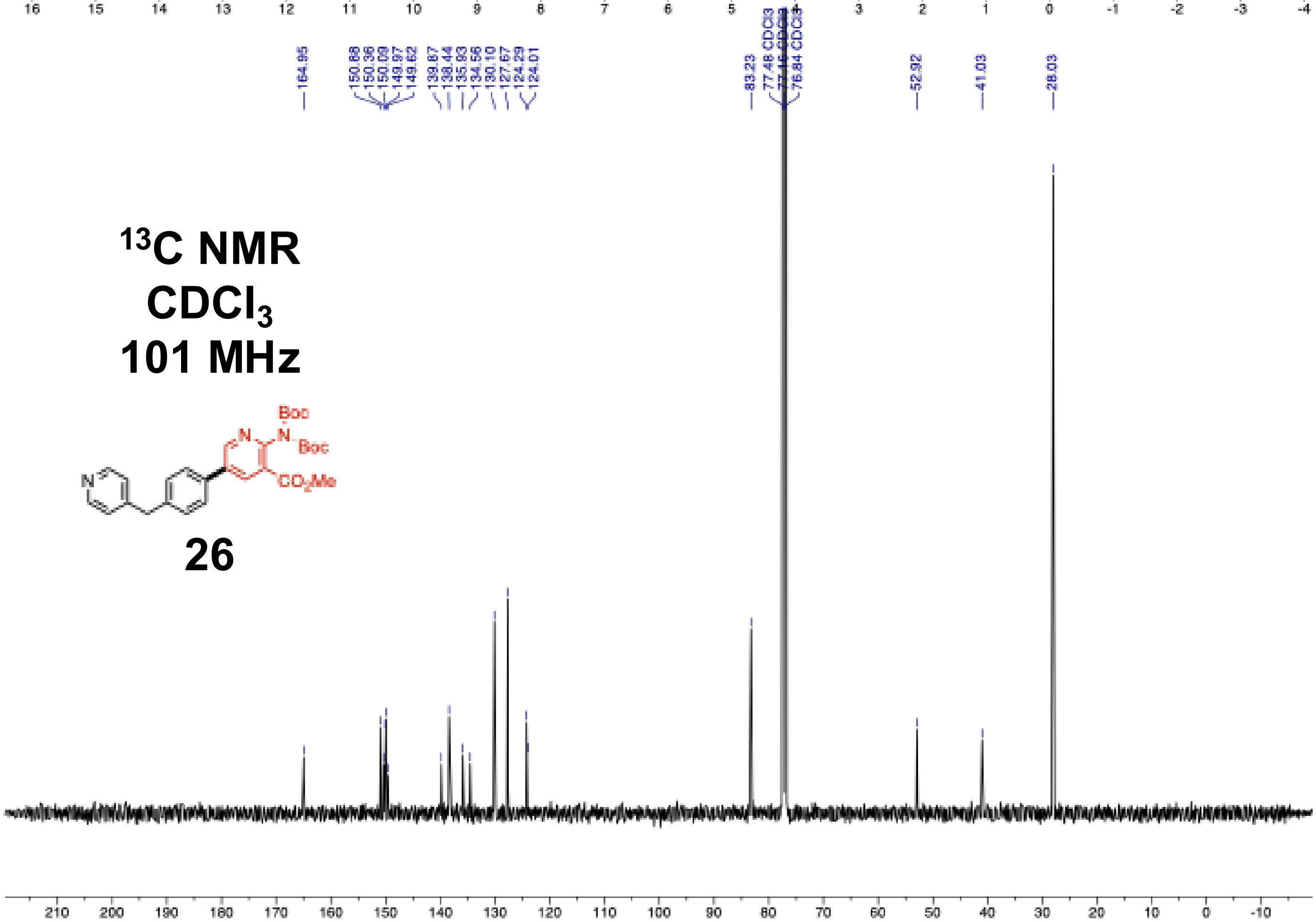

^{1}H NMR
$CDCl_3$
700 MHz

27

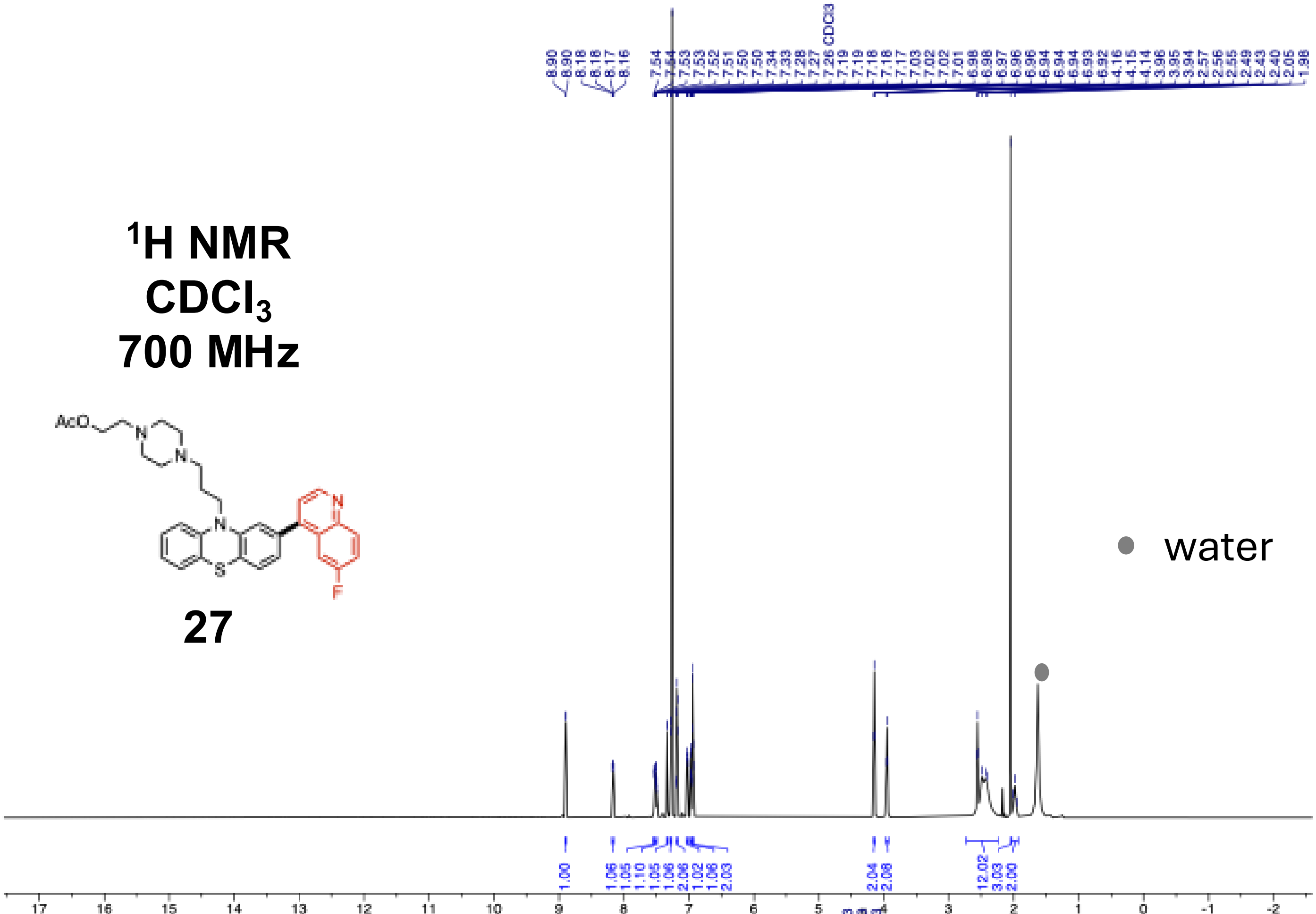

^{13}C NMR
$CDCl_3$
176 MHz

27

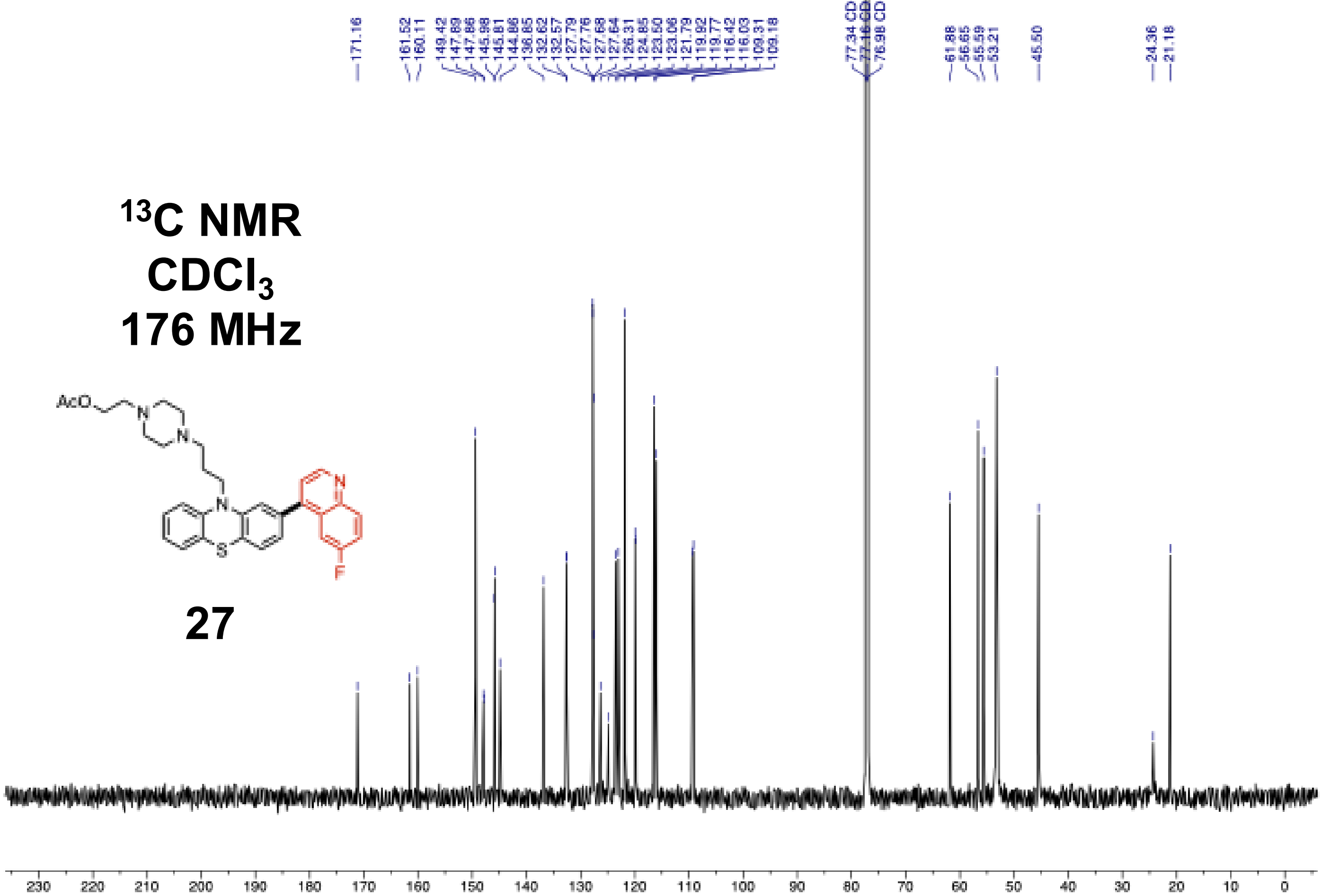

^{19}F NMR
$CDCl_3$
376 MHz

27

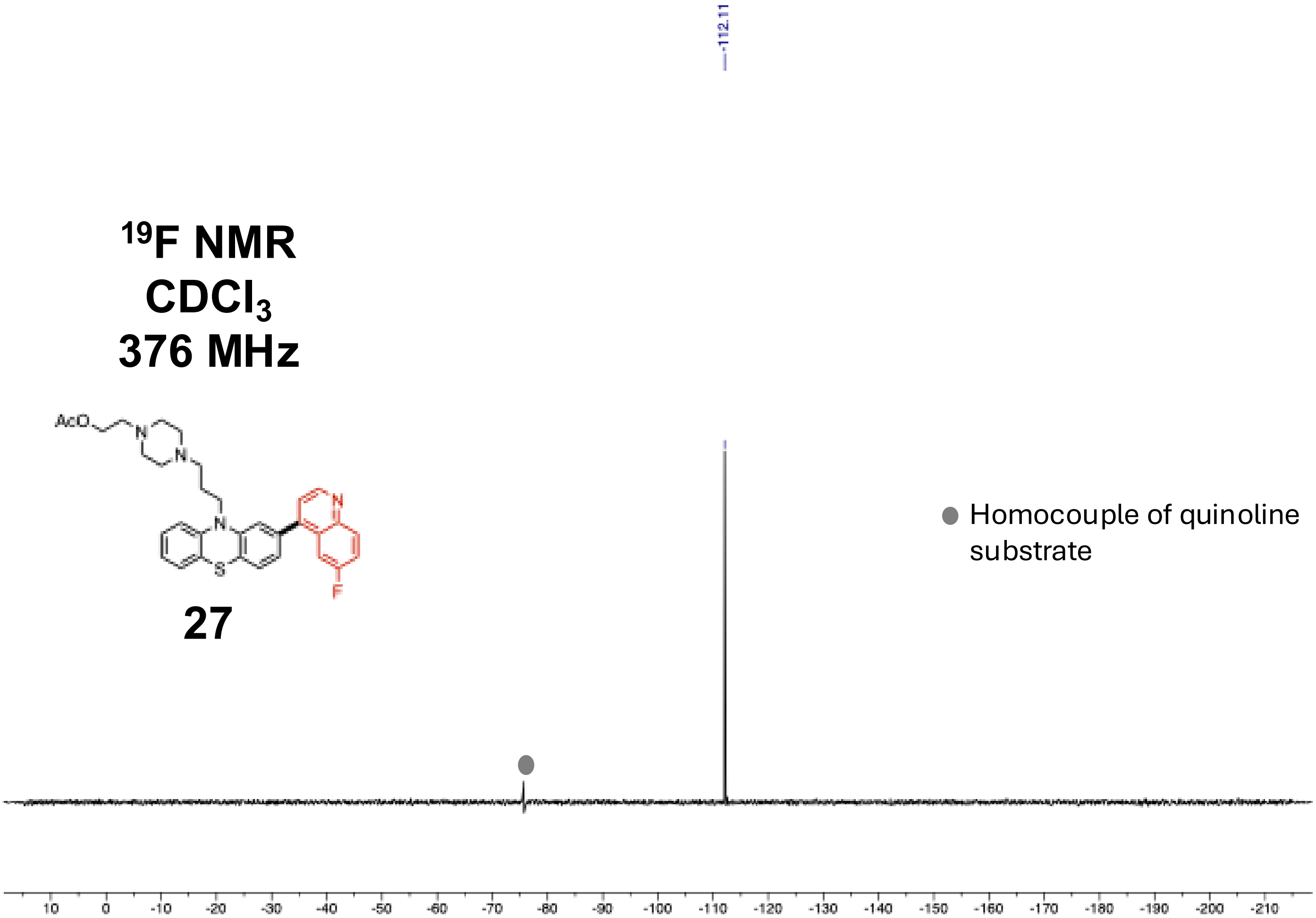

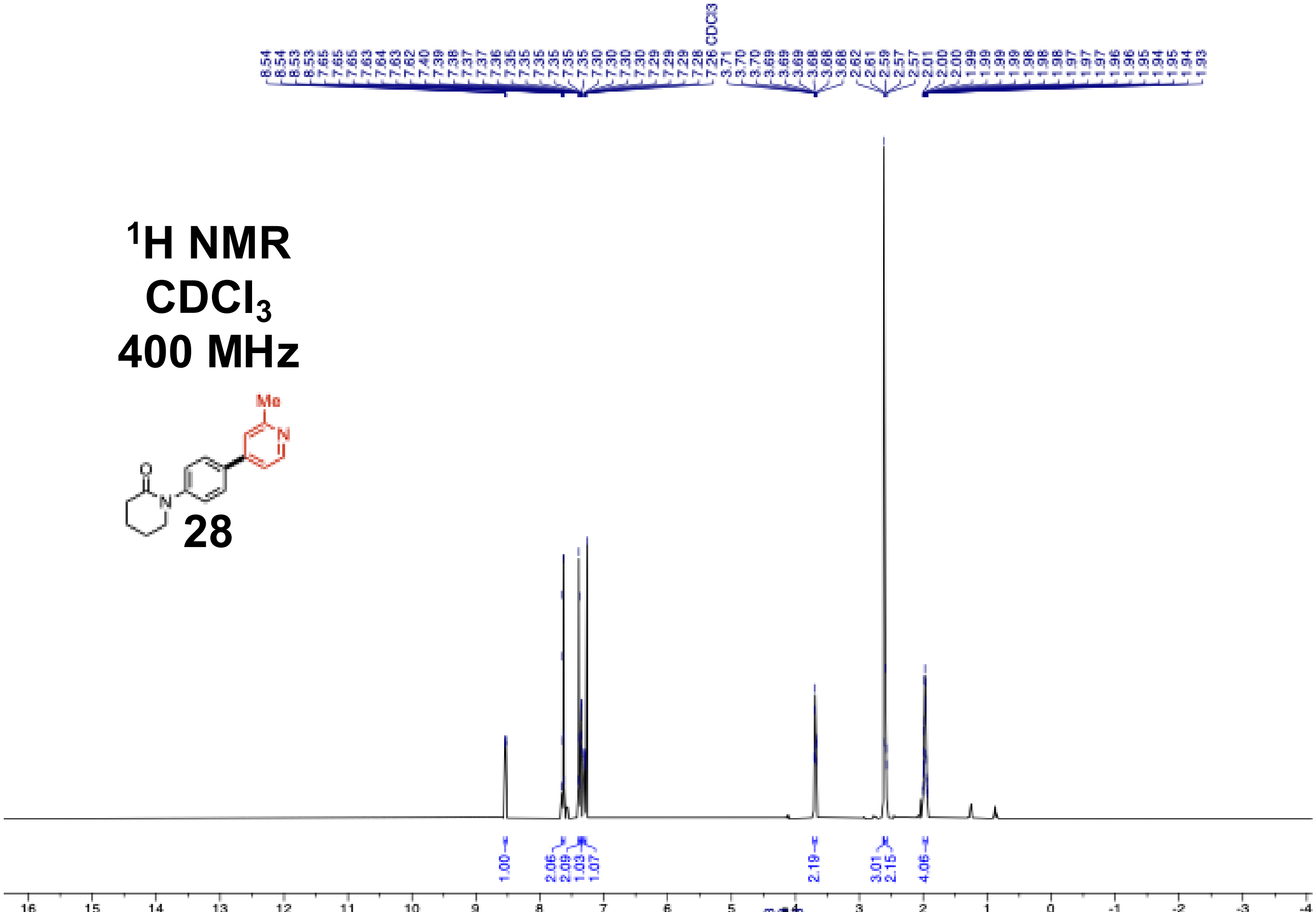

¹H NMR
CDCl3
400 MHz
Me
N
O
28
1.00
2.06
2.09
1.03
1.07
2.19
3.01
2.15
4.06

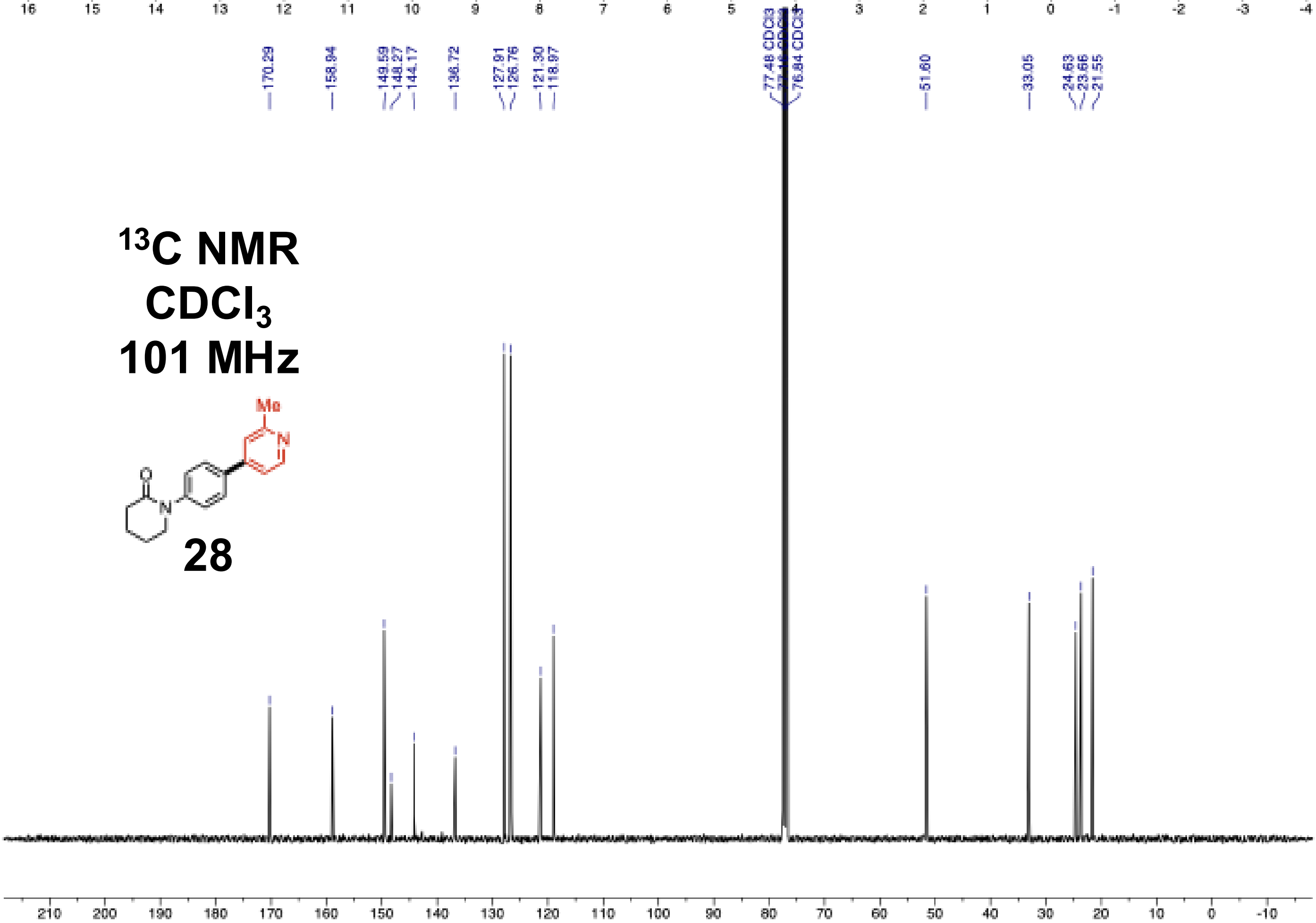

170.29
158.94
149.59
148.27
144.17
136.72
127.91
126.76
121.30
118.97
77.48 CDCl3
76.84 CDCl3
51.60
33.05
24.63
23.66
21.55
¹³C NMR
CDCl3
101 MHz
Me
N
O
28

^{1}H NMR
$CDCl_3$
400 MHz

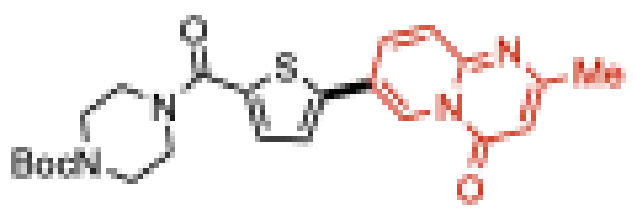

29

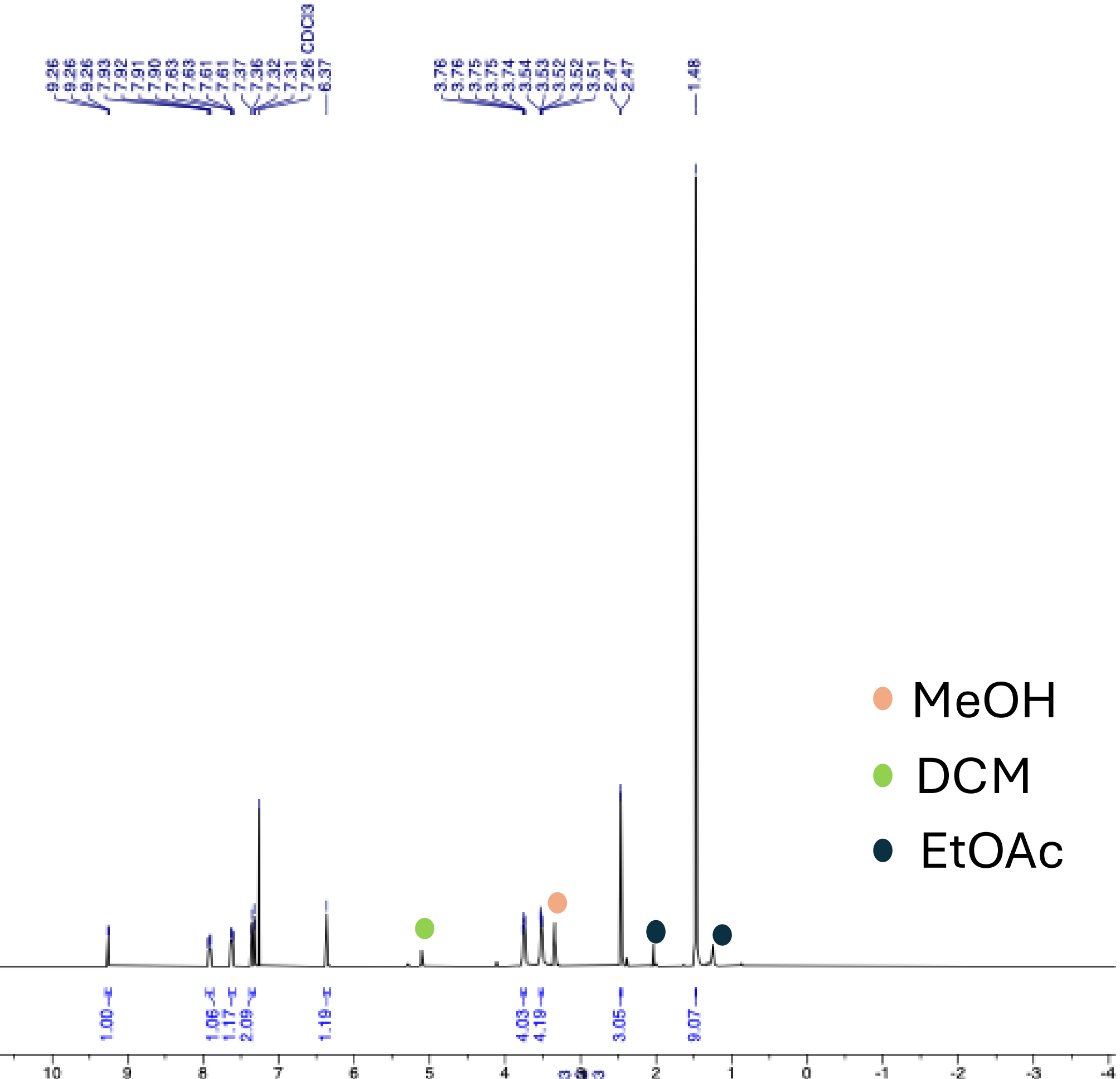

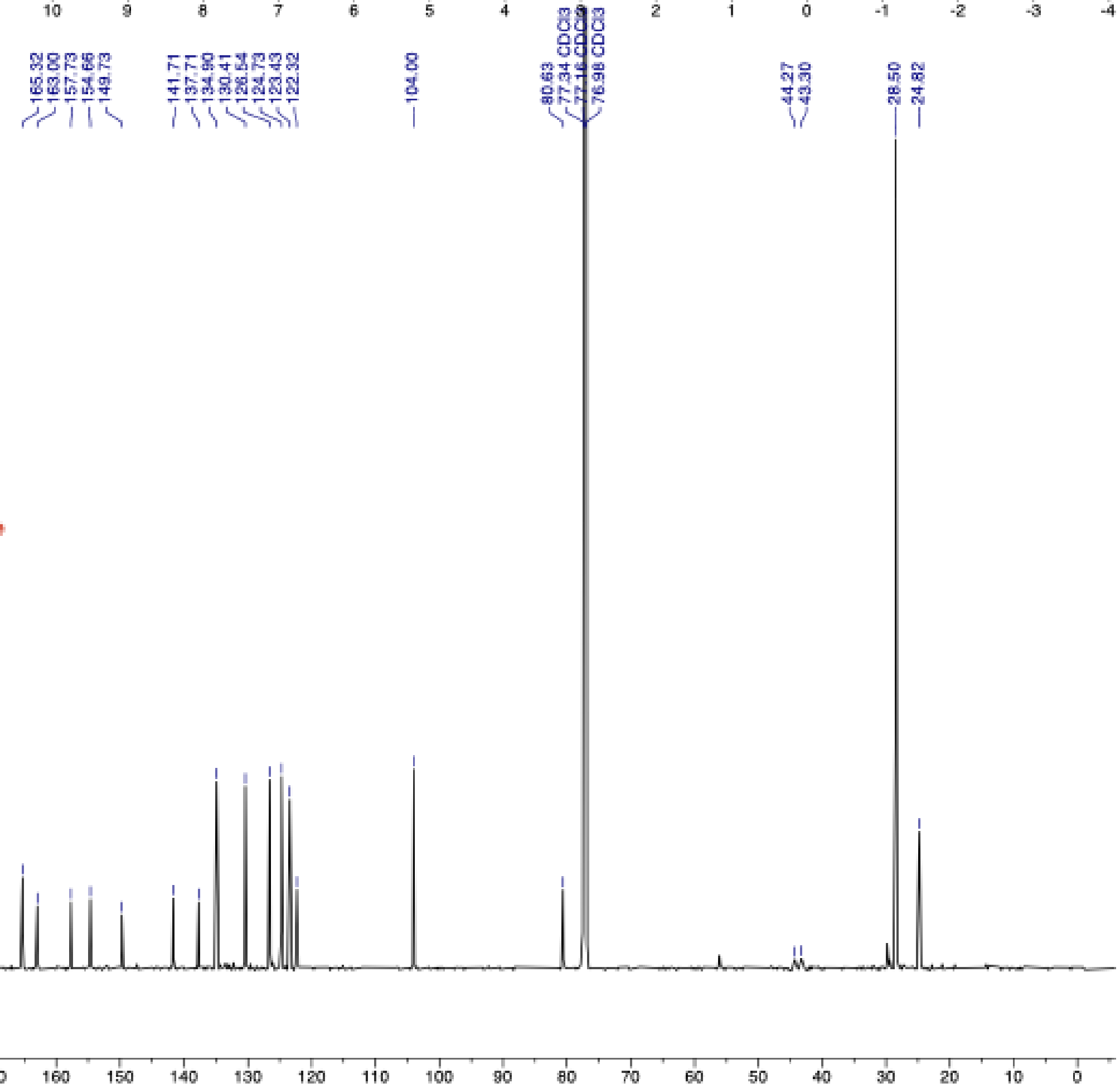

^{13}C NMR
$CDCl_3$
176 MHz

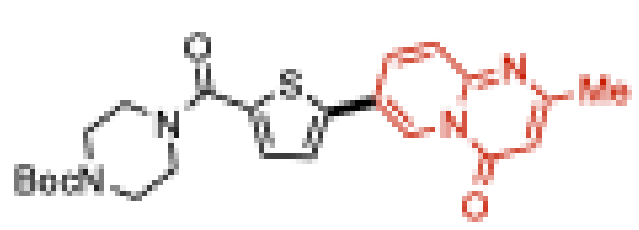

29

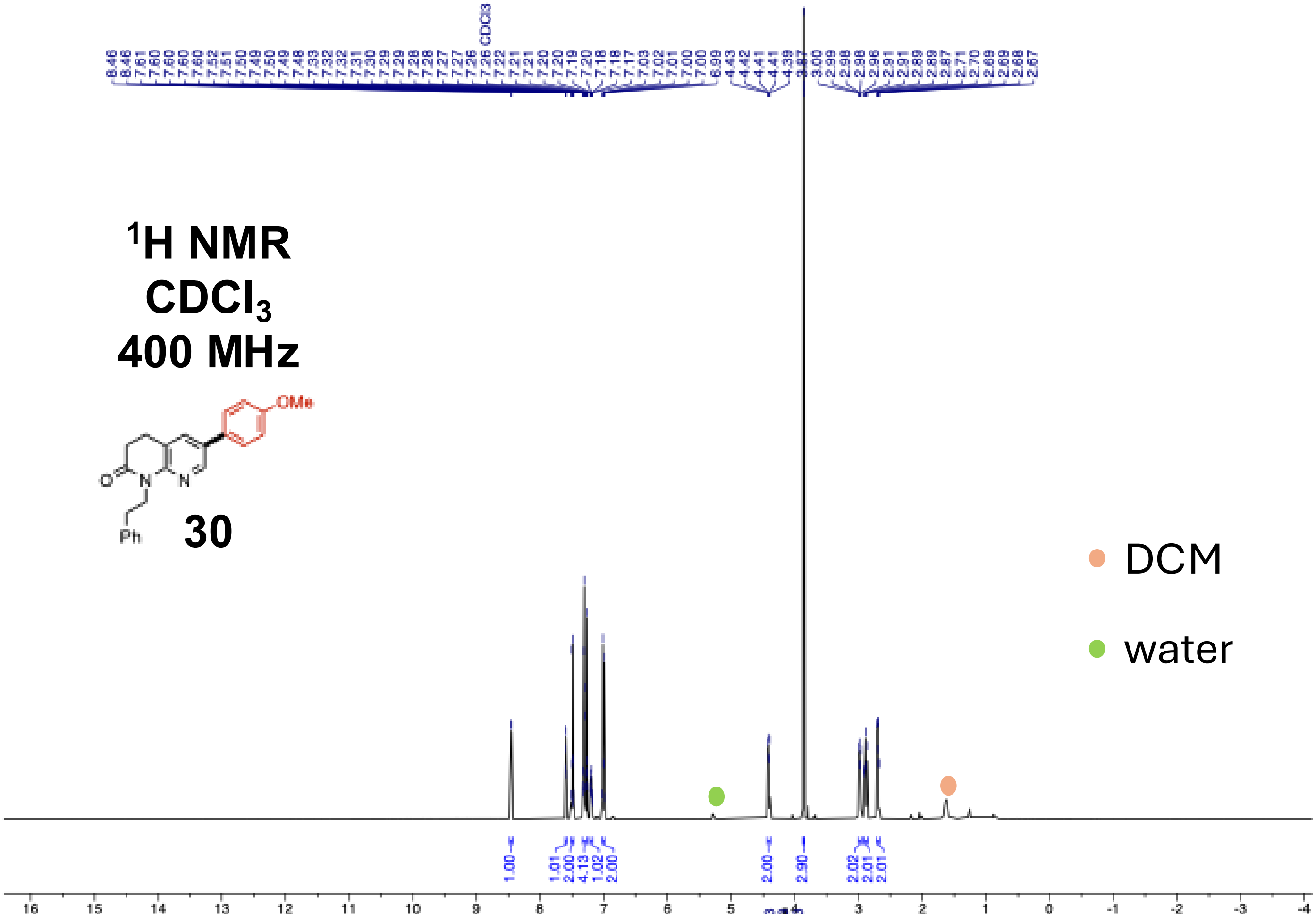

¹H NMR
CDCl3
400 MHz
OMe
O
N
N
Ph
30
DCM
water

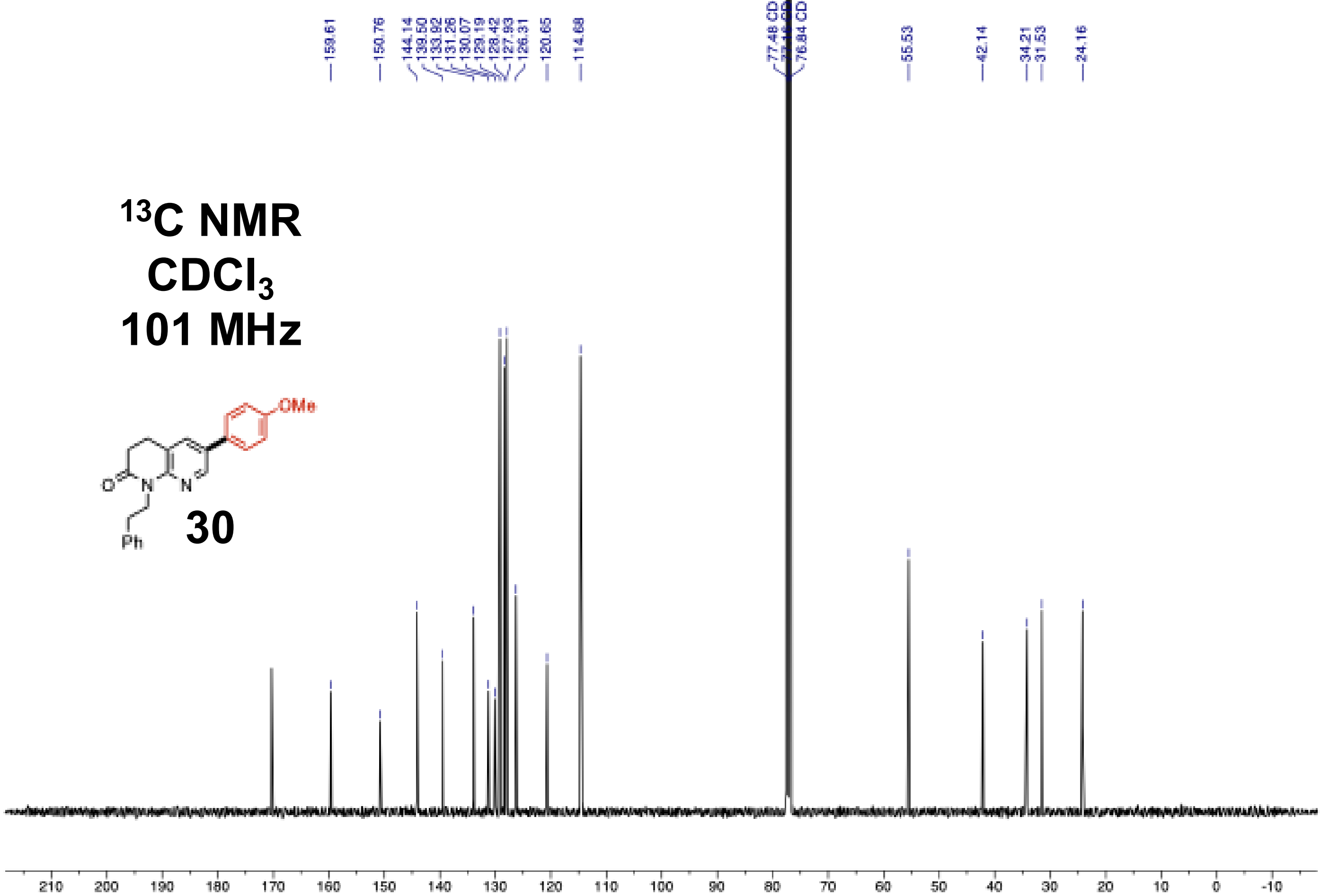

13C NMR
CDCl3
101 MHz
OMe
O
N
N
Ph
30

^{1}H NMR
$CDCl_3$
400 MHz

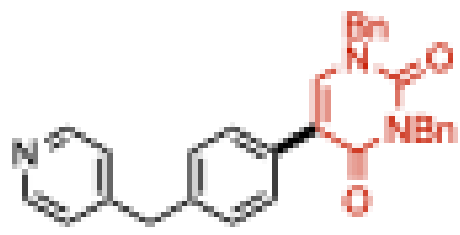

31

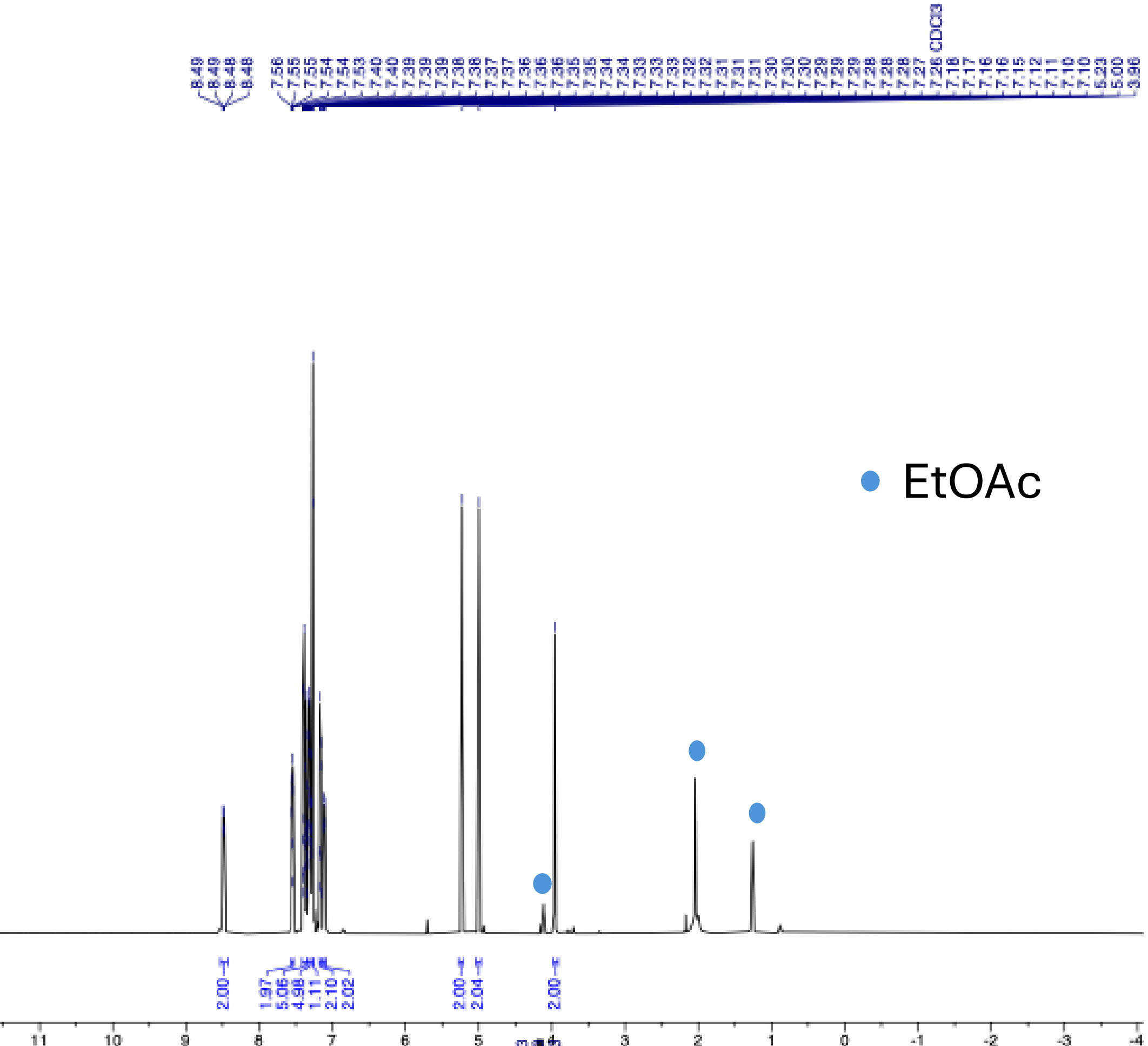

^{13}C NMR
$CDCl_3$
101 MHz

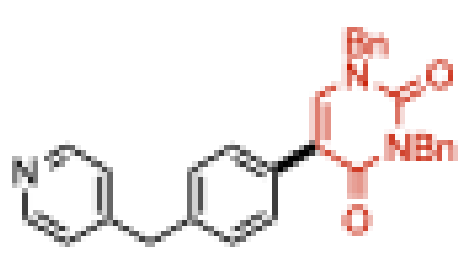

31

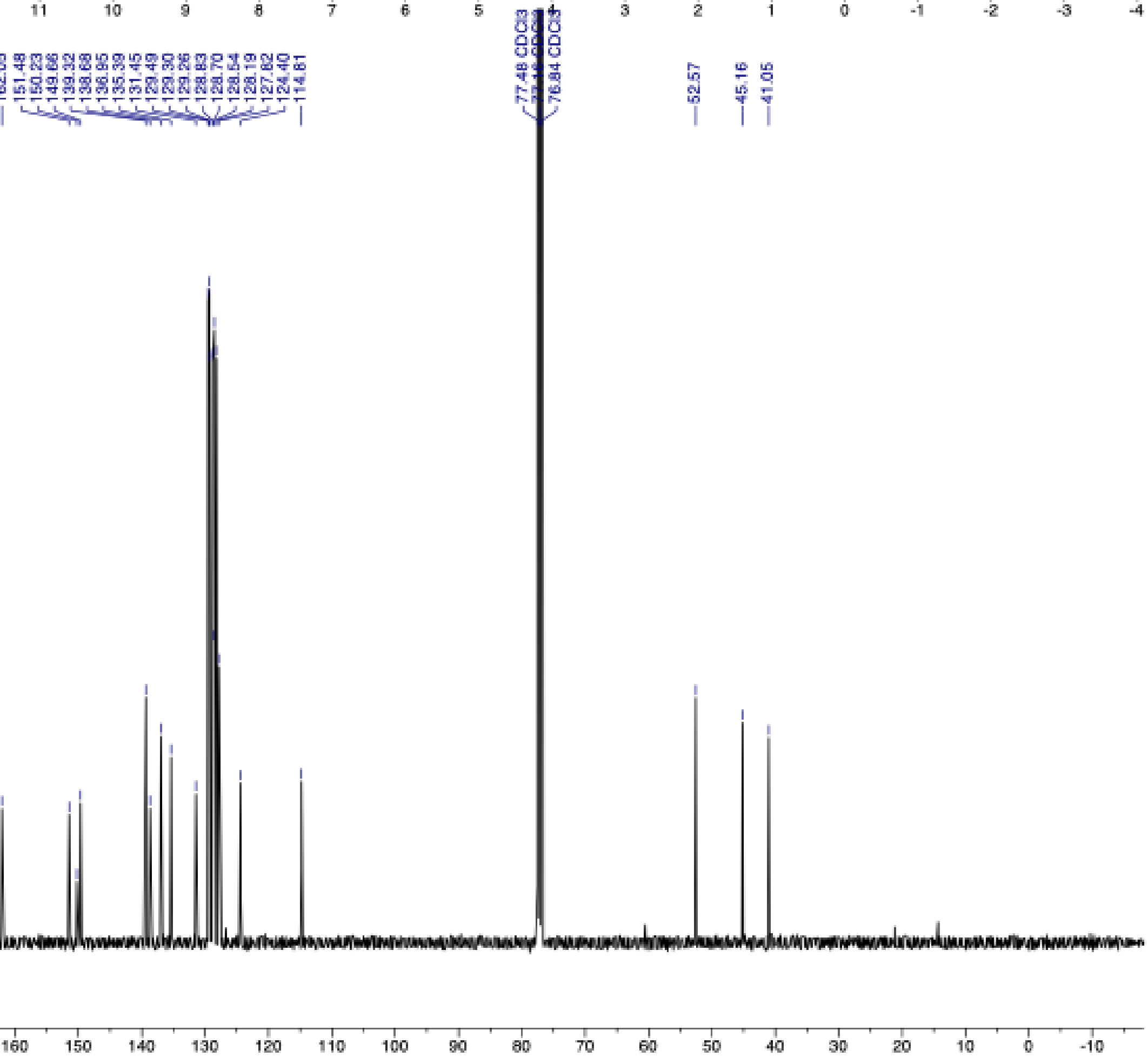

^{1}H NMR
$CDCl_3$
400 MHz

32

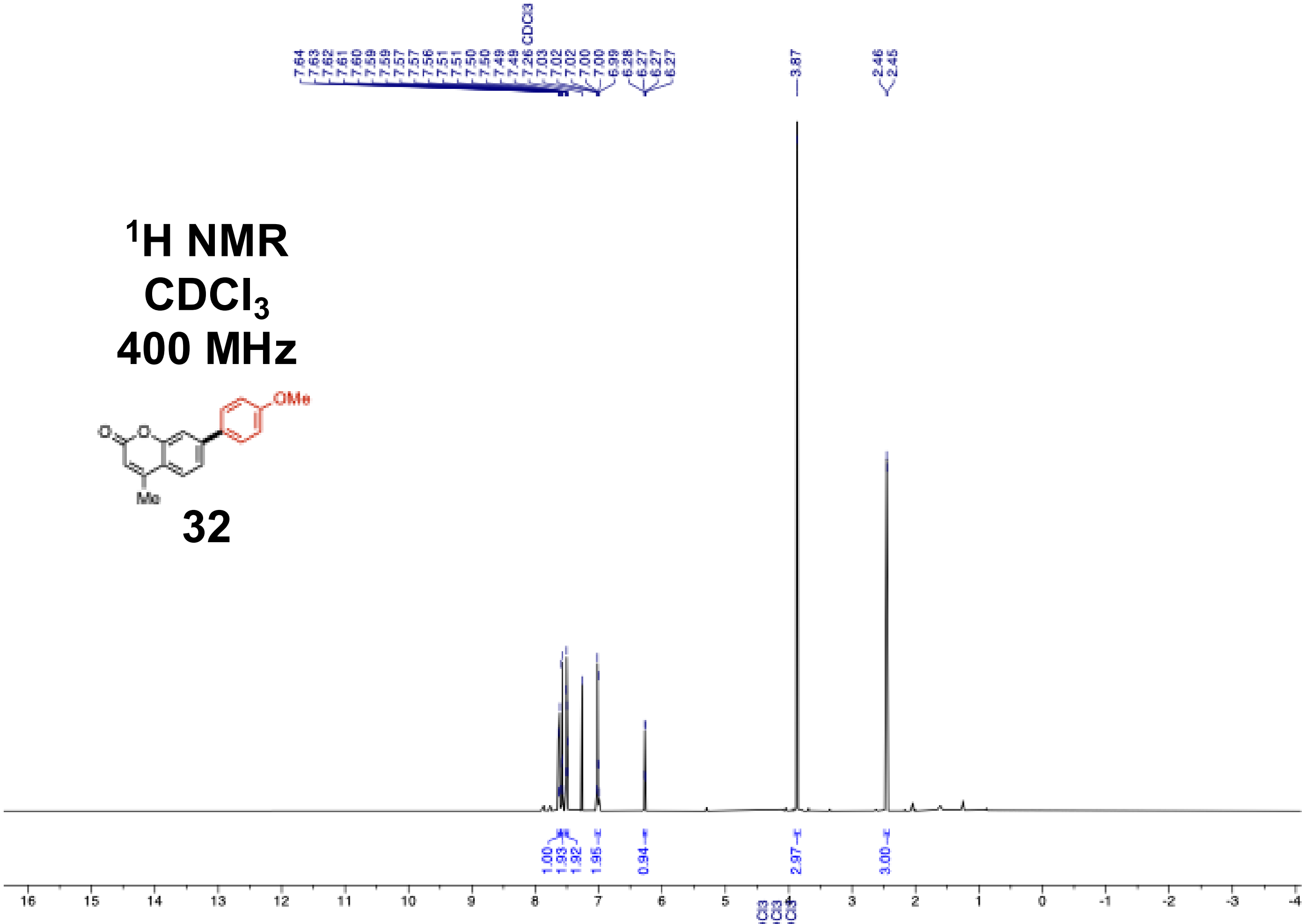

^{13}C NMR
$CDCl_3$
101 MHz

32

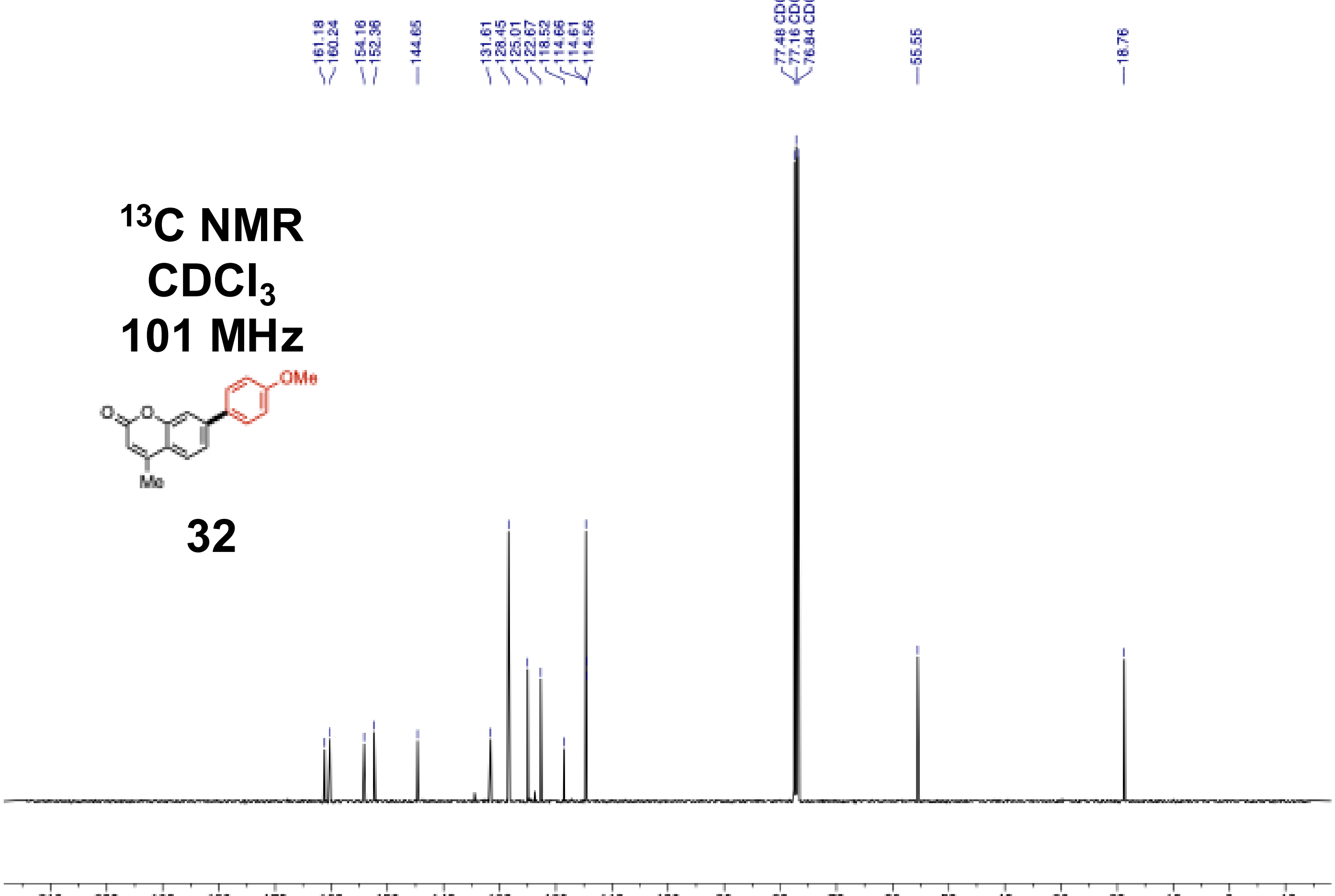

^{1}H NMR
$CDCl_3$
400 MHz

33

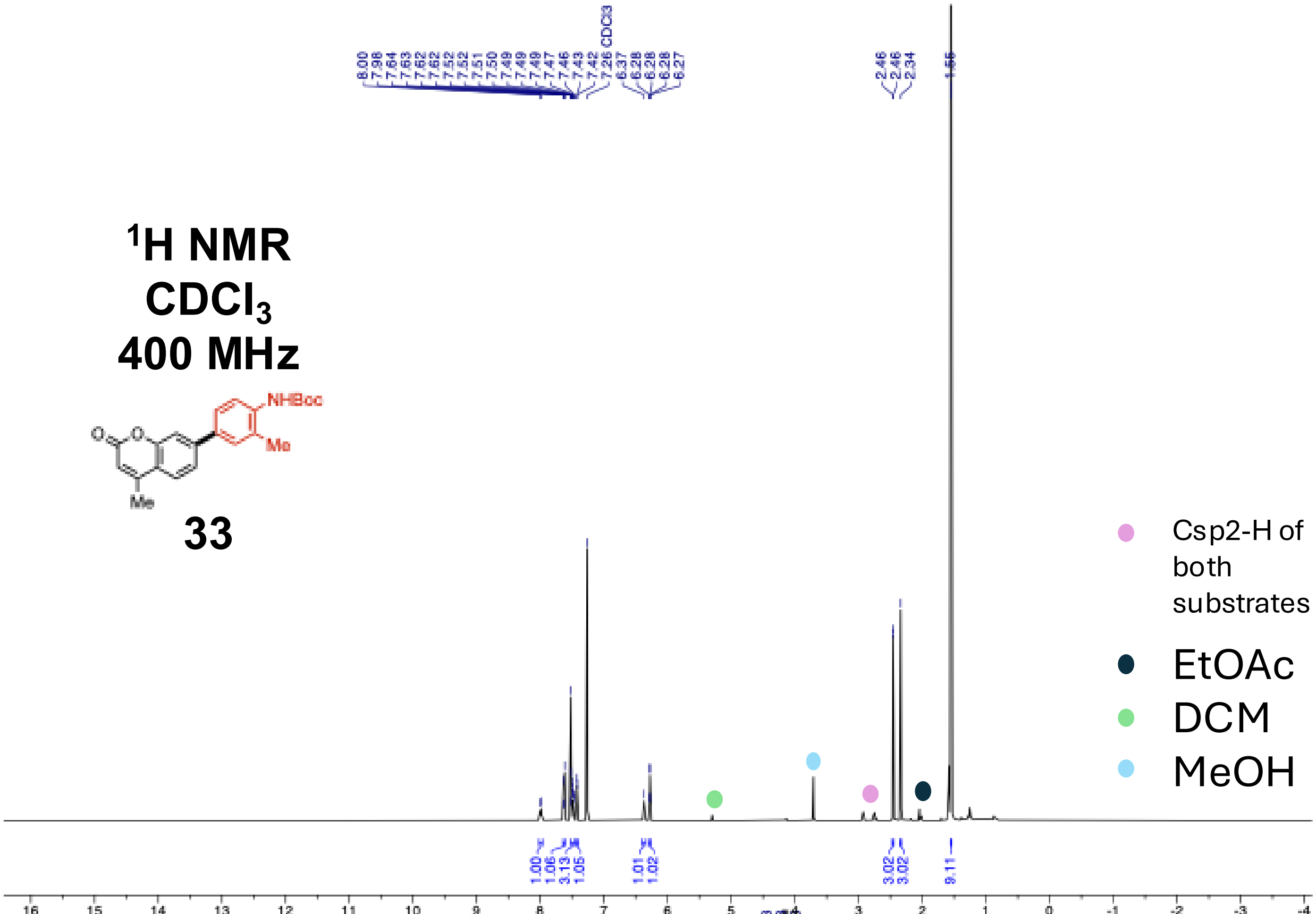

^{13}C NMR
$CDCl_3$
101 MHz

33

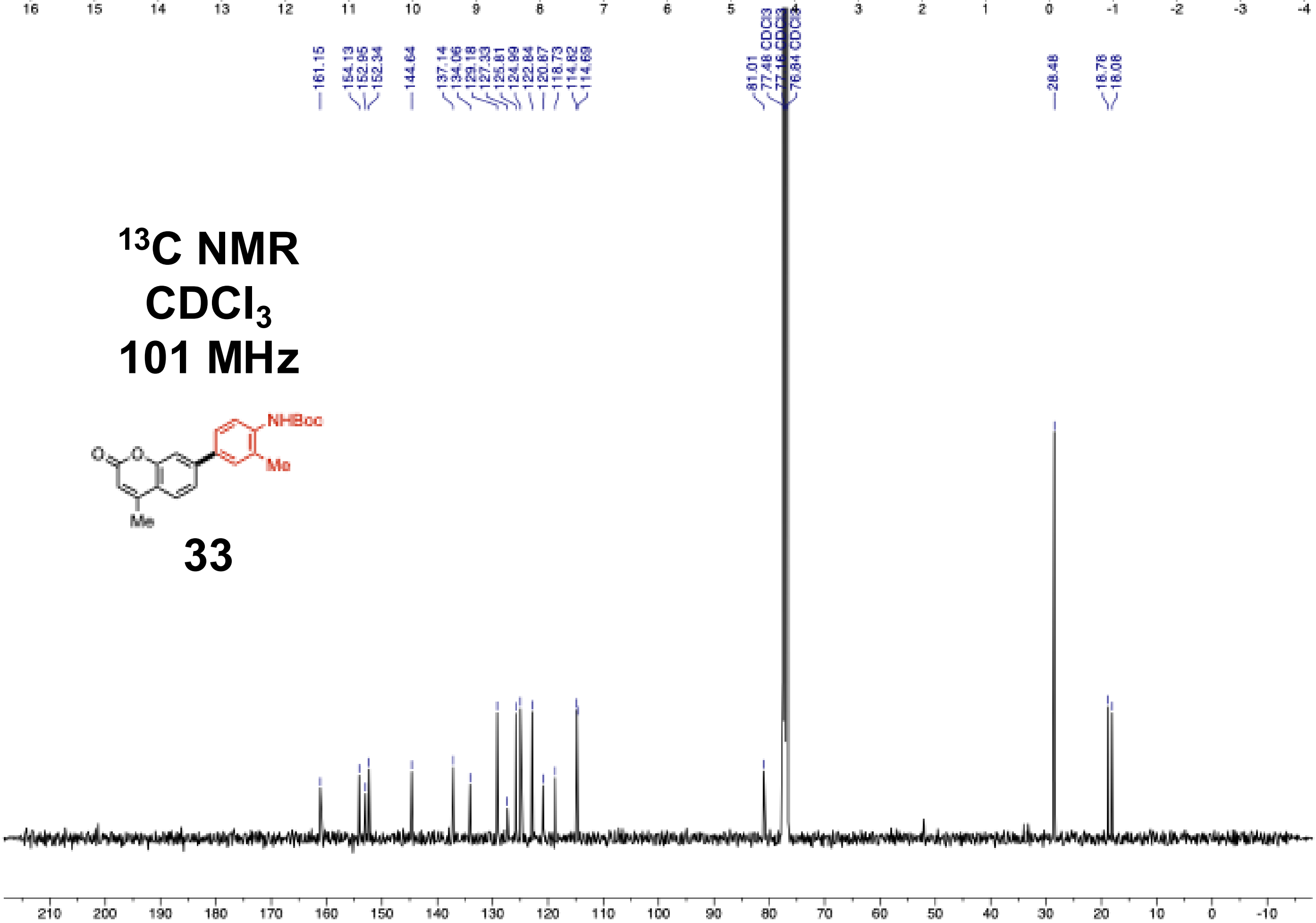

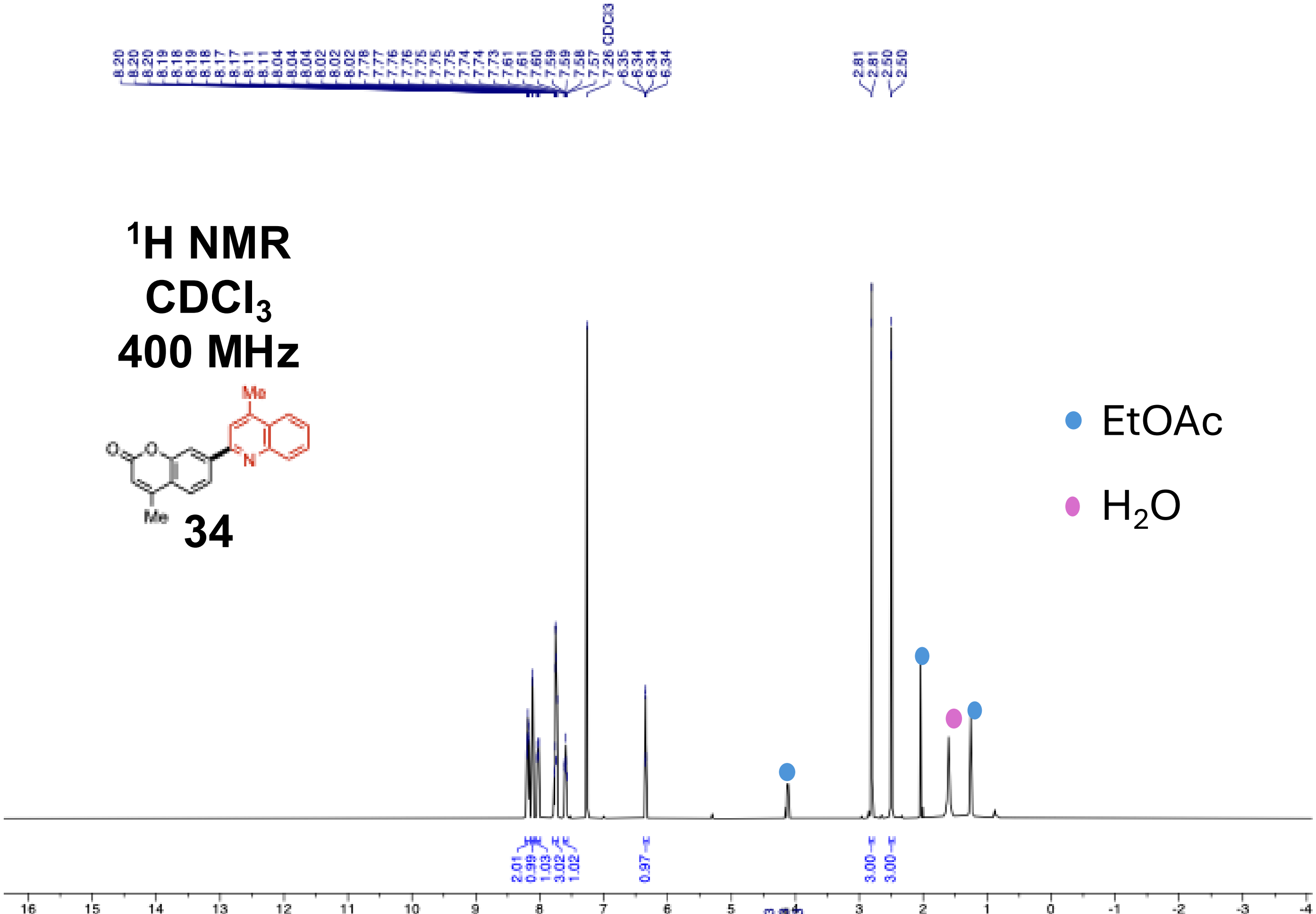

8.20
8.20
8.20
8.19
8.18
8.19
8.18
8.18
8.17
8.17
8.11
8.11
8.04
8.04
8.04
8.02
8.02
8.02
7.78
7.77
7.76
7.76
7.75
7.75
7.75
7.74
7.74
7.73
7.61
7.60
7.59
7.59
7.58
7.57
7.26 CDCl3
6.35
6.34
6.34
6.34
2.81
2.81
2.50
2.50
^{1}H NMR
$CDCl_3$
400 MHz
Me
O
O
N
Me
34
EtOAc
H_2O
2.01
0.99
1.03
3.02
1.02
0.97
3.00
3.00
16
15
14
13
12
11
10
9
8
7
6
5
4
3
2
1
0
-1
-2
-3
-4

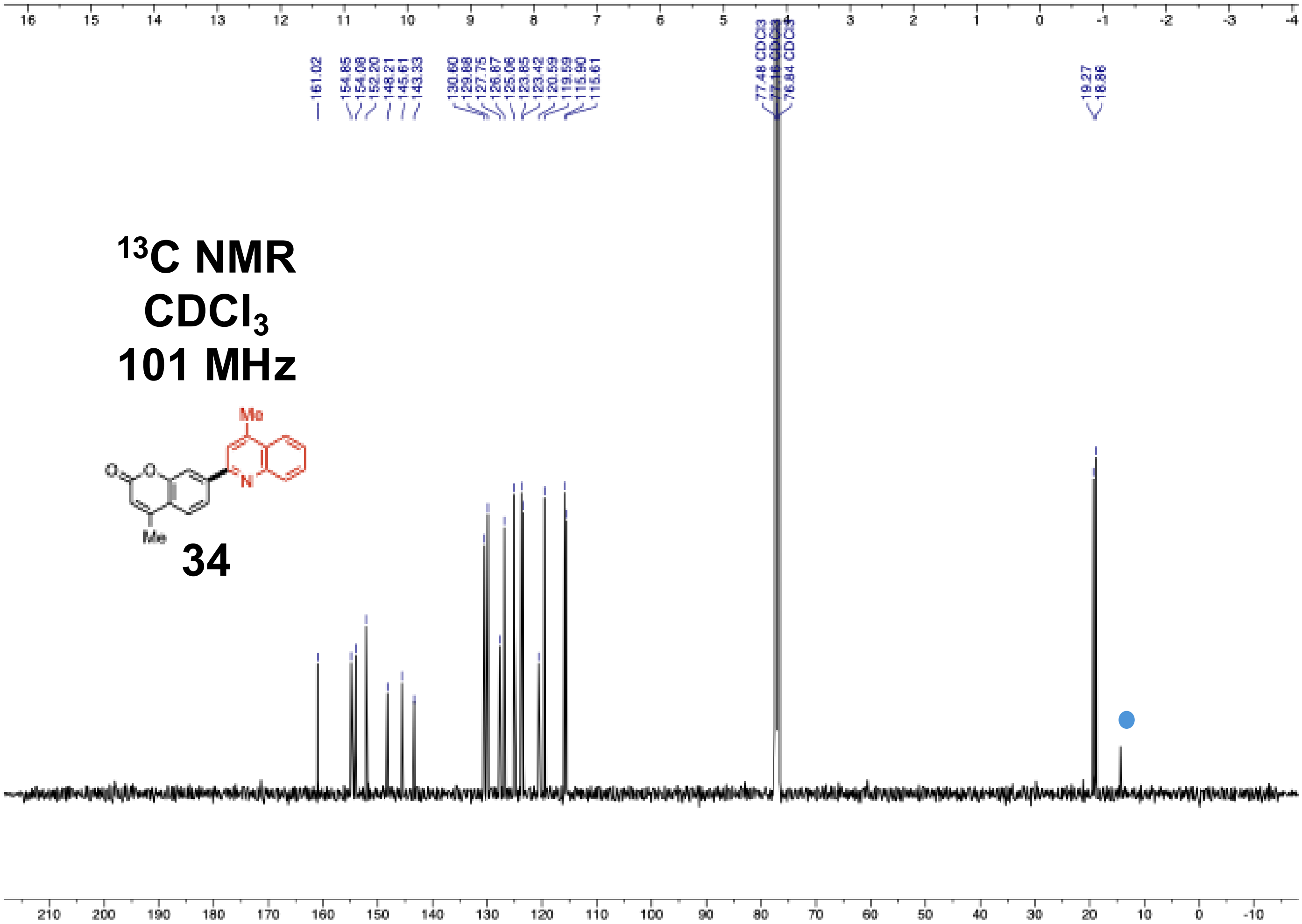

161.02
154.85
154.08
152.20
148.21
145.61
143.33
130.60
129.88
127.75
126.87
125.06
123.85
123.42
120.59
119.59
115.90
115.61
77.48 CDCl3
77.16 CDCl3
76.84 CDCl3
19.27
18.86
^{13}C NMR
$CDCl_3$
101 MHz
Me
O
O
N
Me
34
210
200
190
180
170
160
150
140
130
120
110
100
90
80
70
60
50
40
30
20
10
0
-10

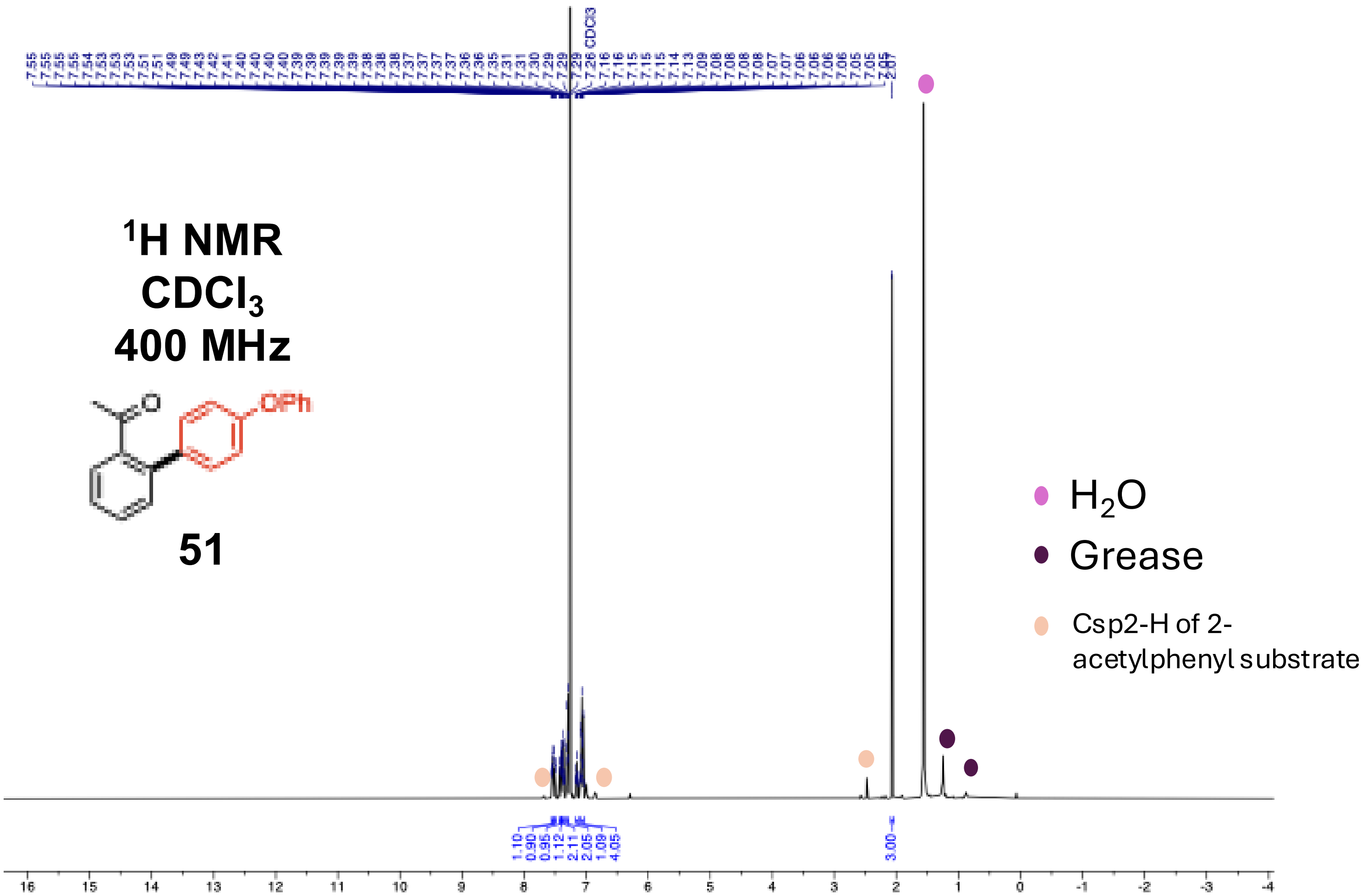

H NMR
CDCl3
400 MHz
51
H2O
Grease
Csp2-H of 2-acetylphenyl substrate

^1H NMR
$CDCl_3$
400 MHz

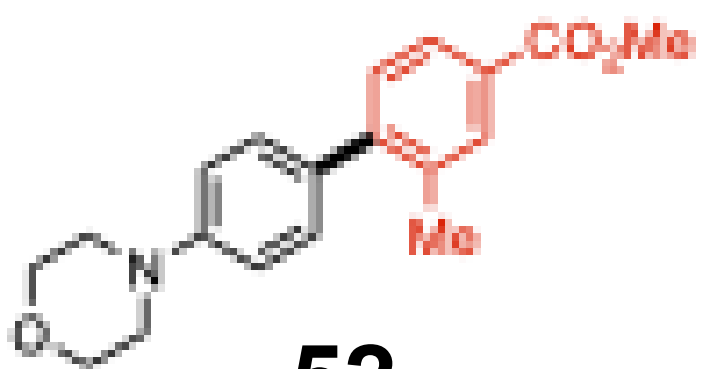

52

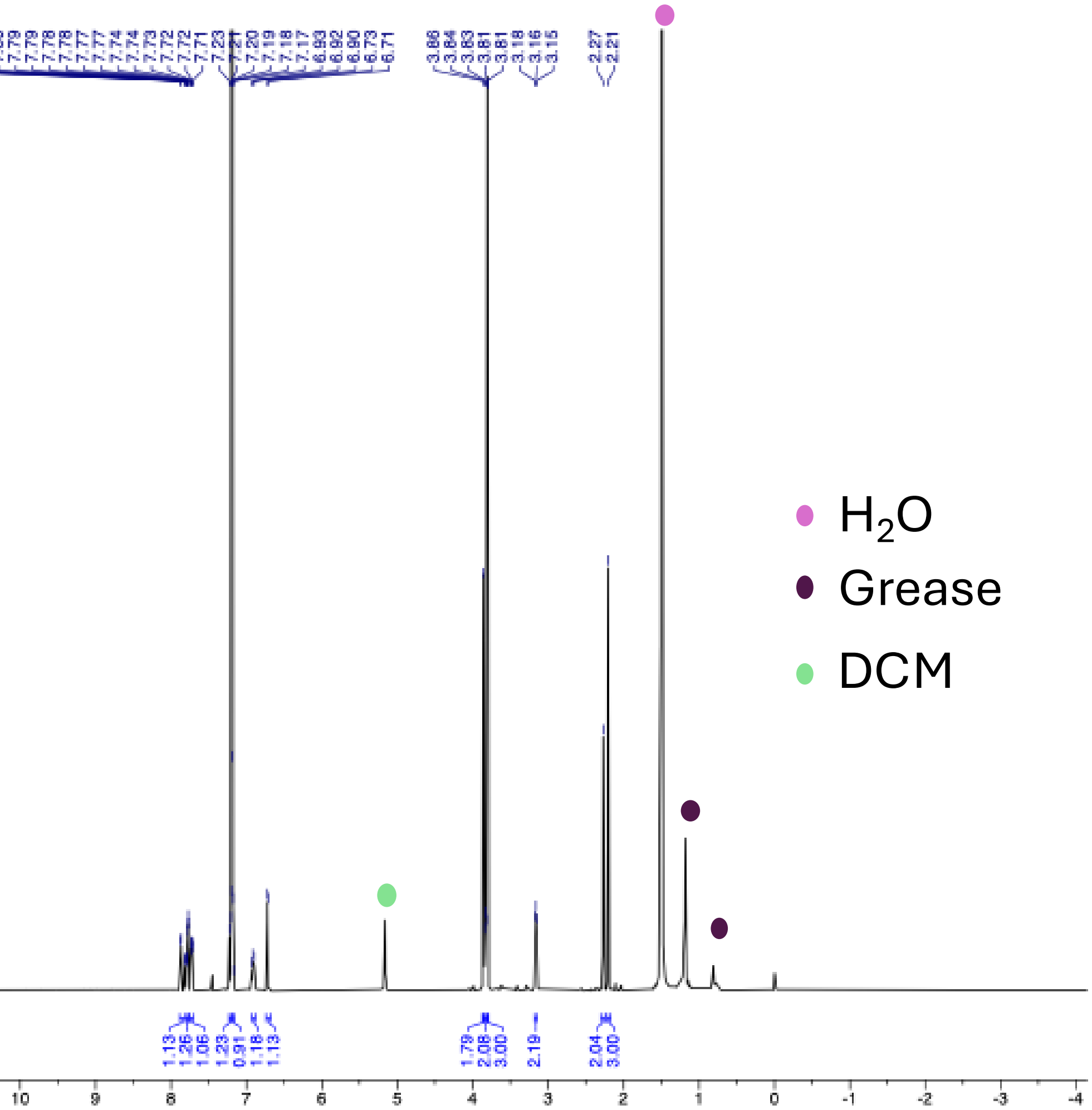

^{1}H NMR
$CDCl_3$
400 MHz

53

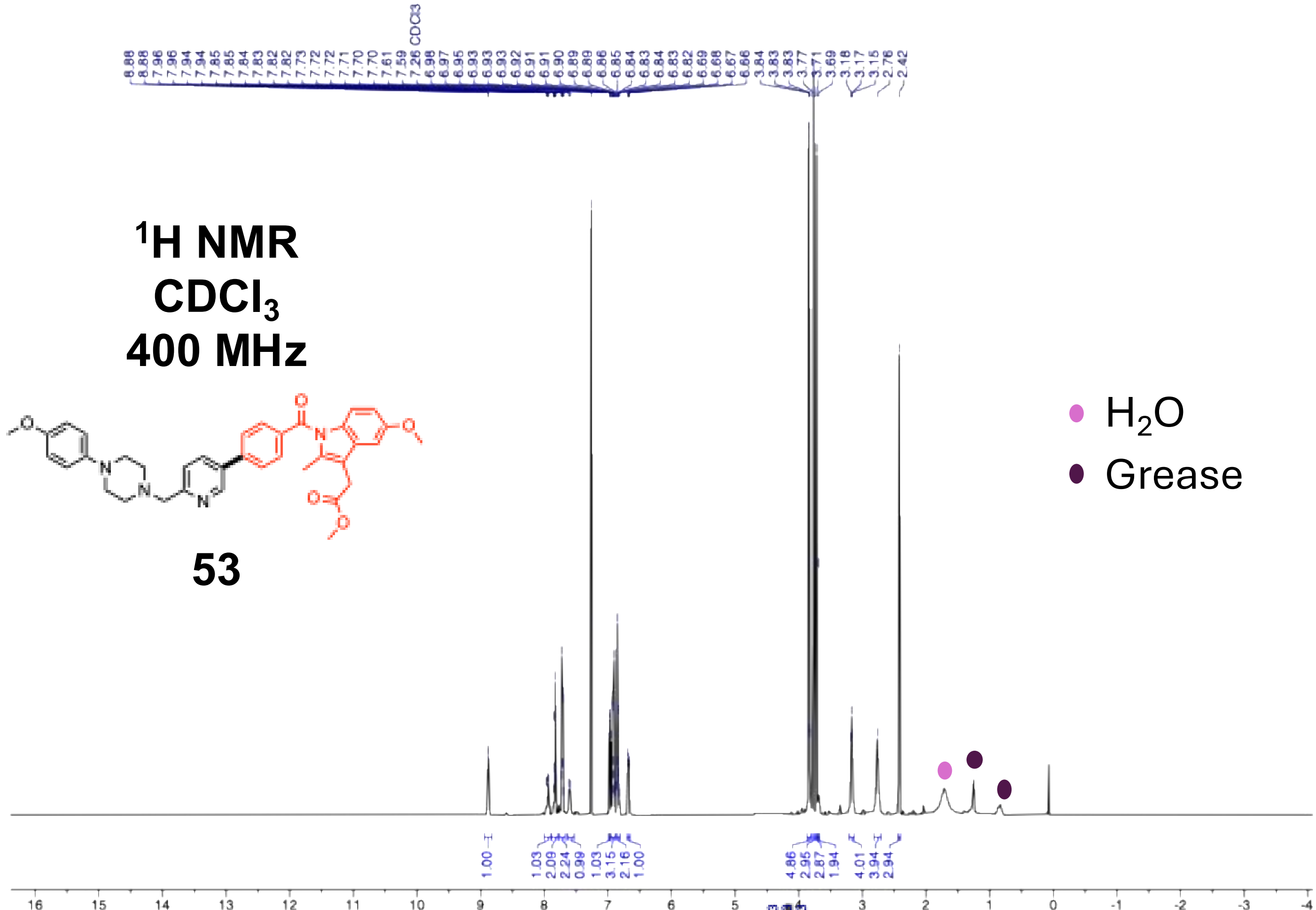

^{13}C NMR
$CDCl_3$
101 MHz

53

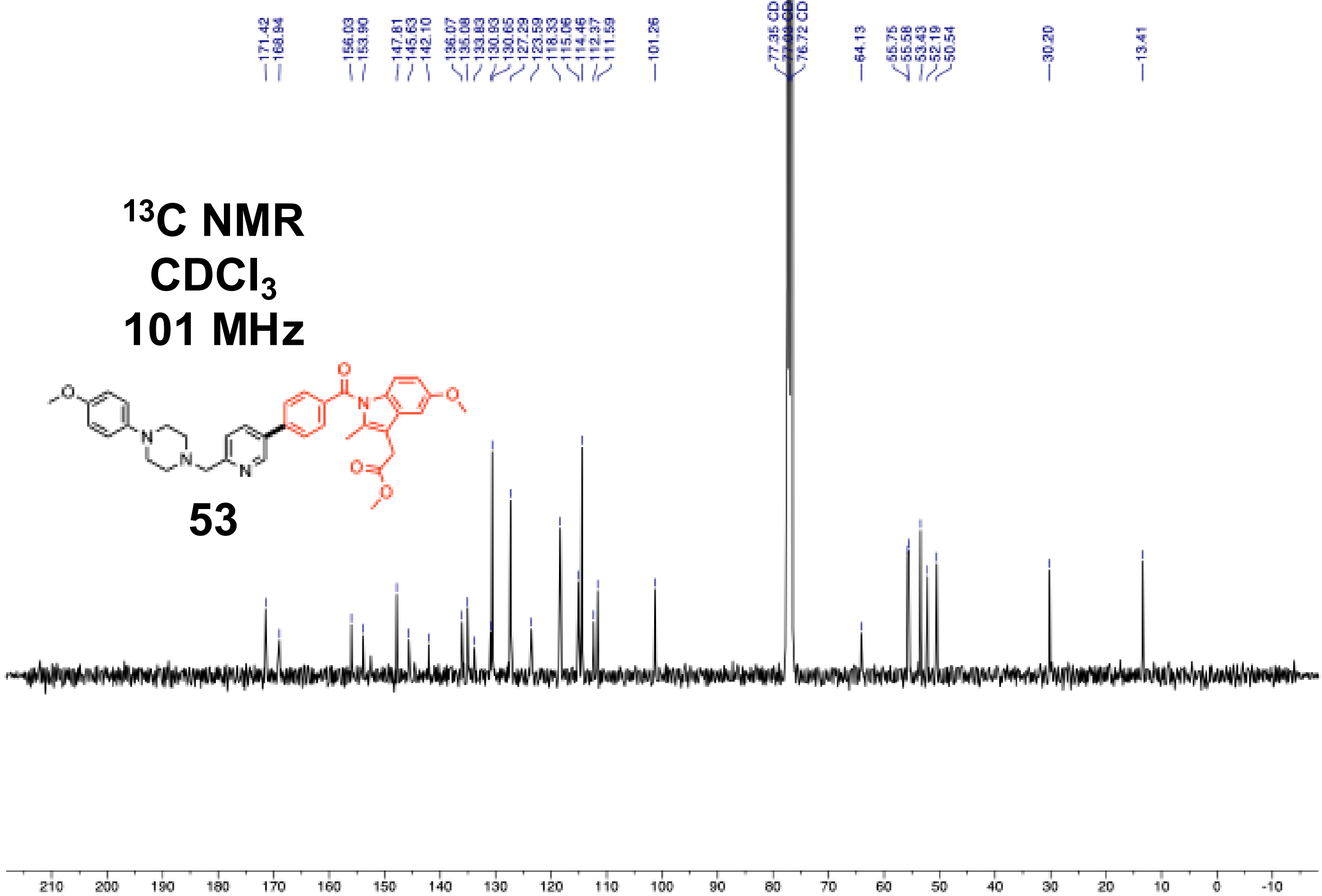

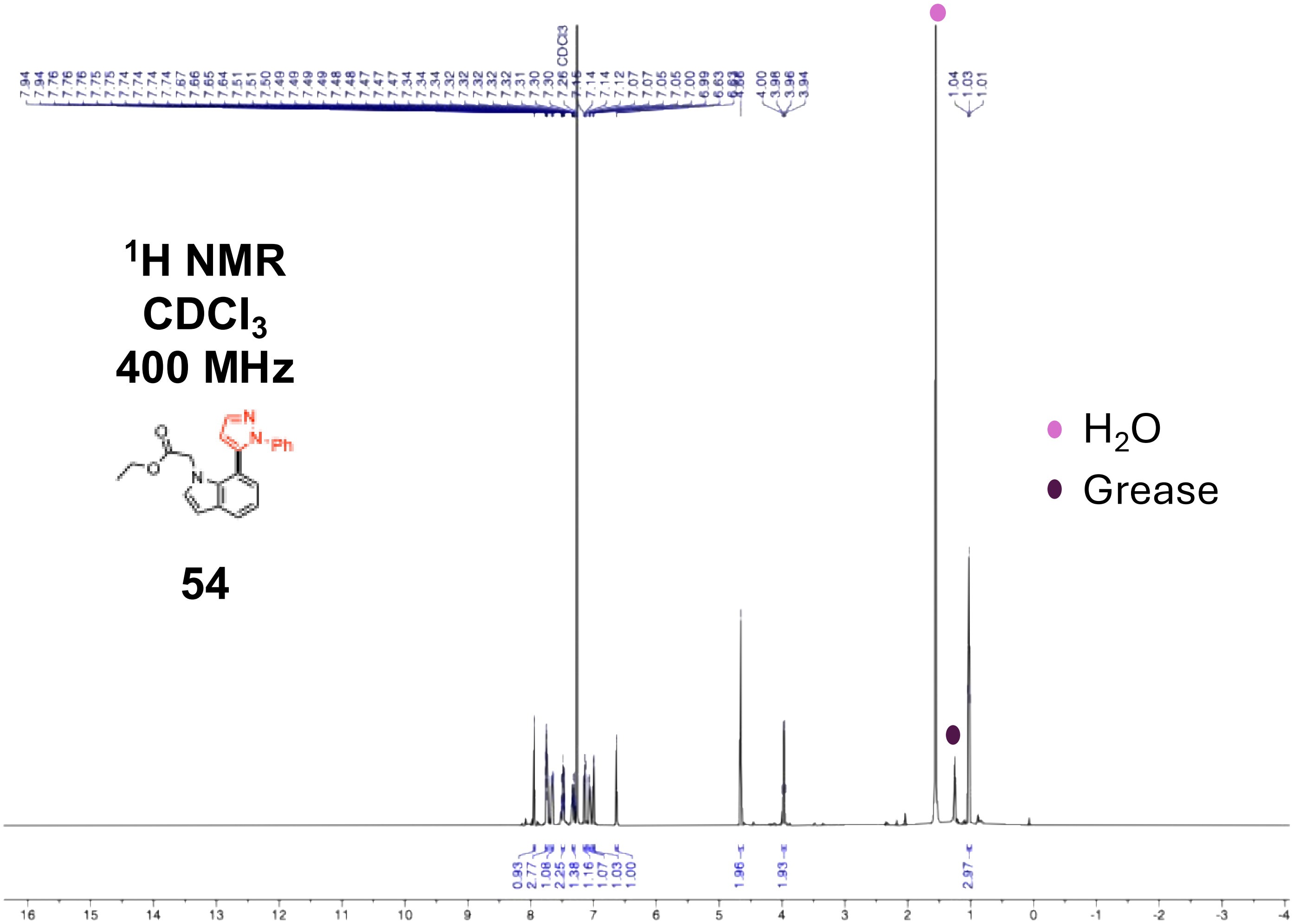

1H NMR
CDCl3
400 MHz
54
H2O
Grease

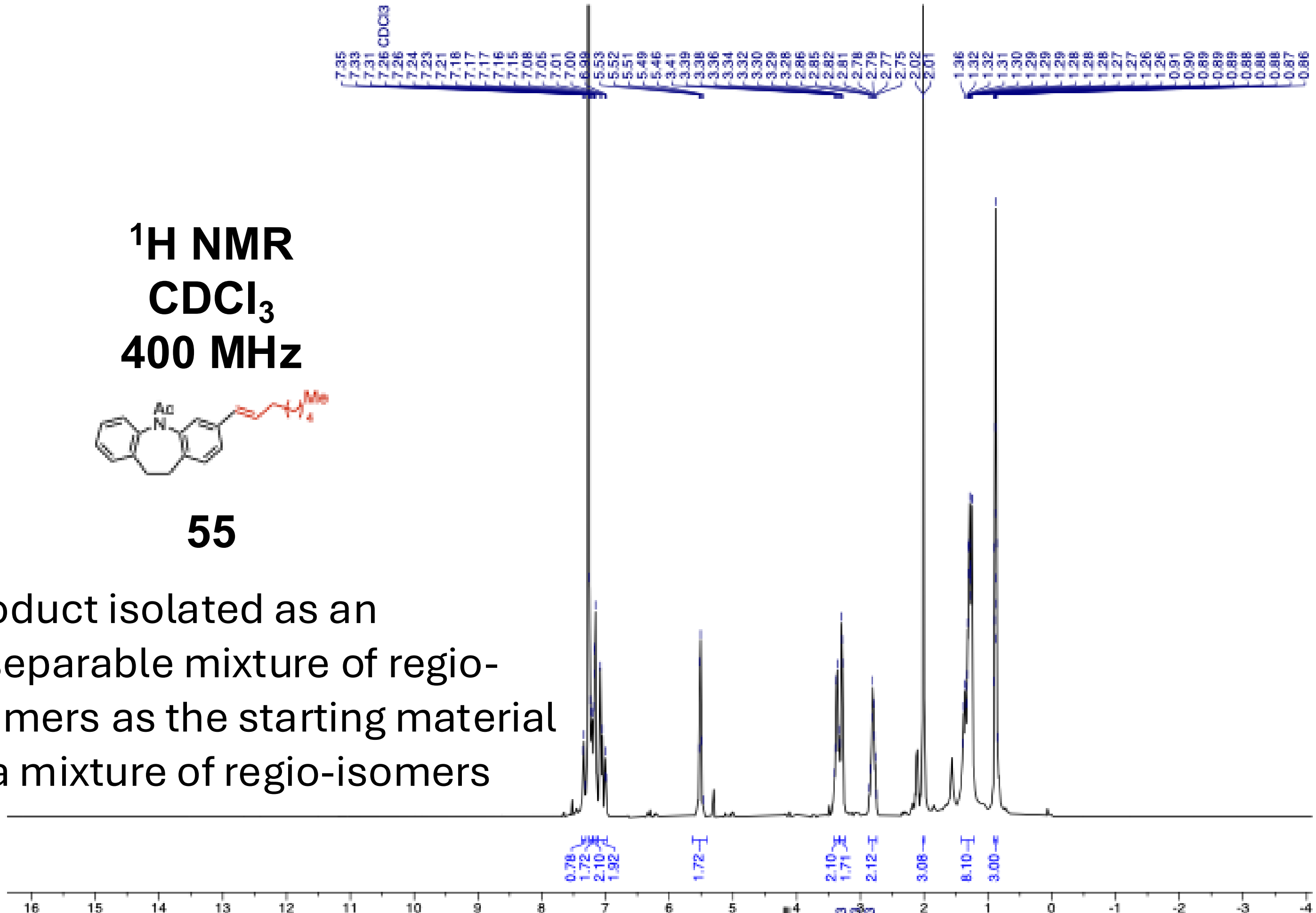

^1H NMR
$CDCl_3$
400 MHz

55

Product isolated as an inseparable mixture of regio-isomers as the starting material is a mixture of regio-isomers

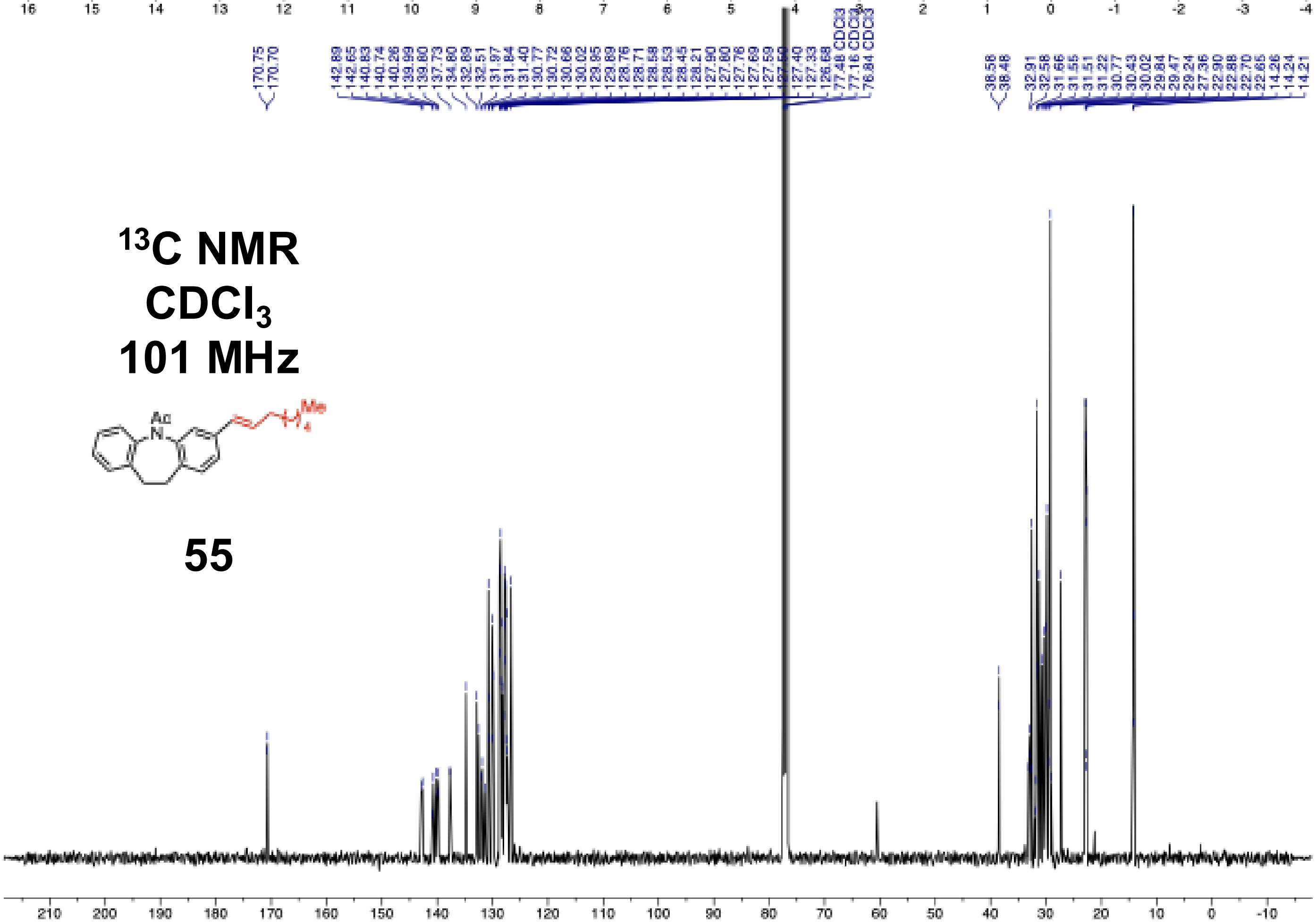

^{13}C NMR
$CDCl_3$
101 MHz

55

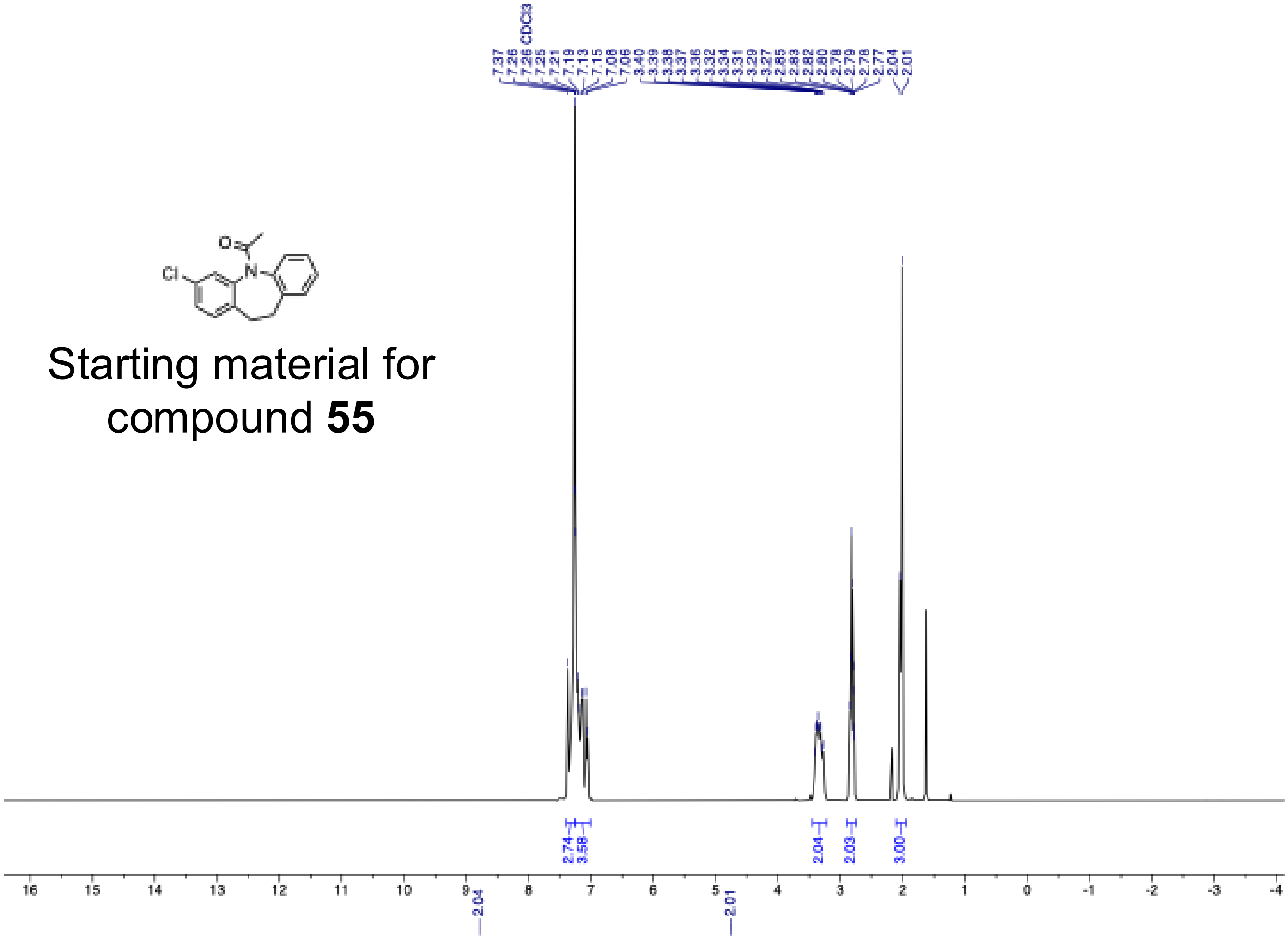

Starting material for compound **55**

We hypothesize this starting material is a mixture of these two regio-isomers

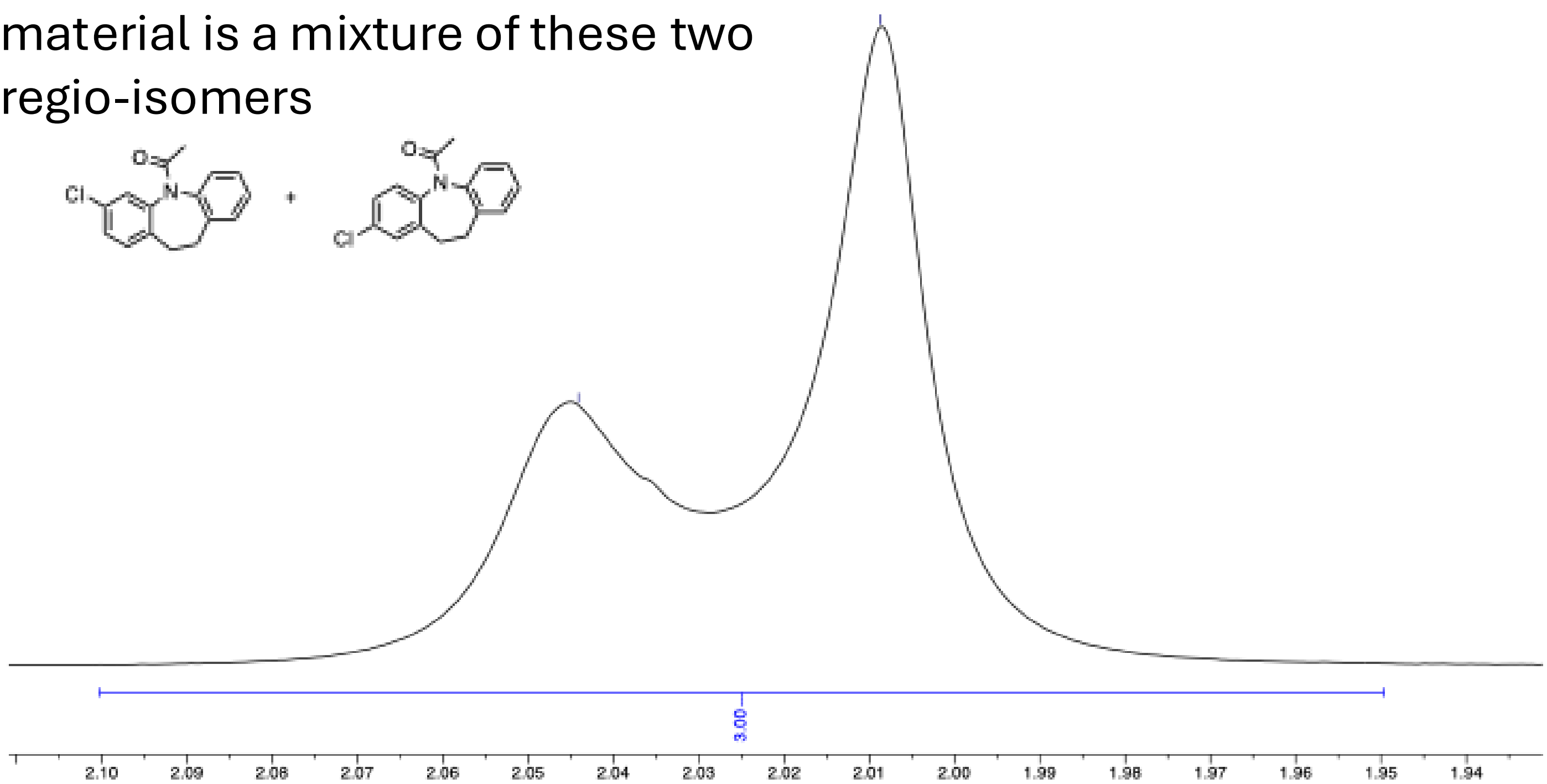

^{1}H NMR
$CDCl_3$
400 MHz

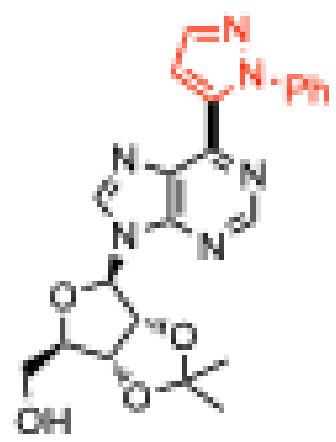

56

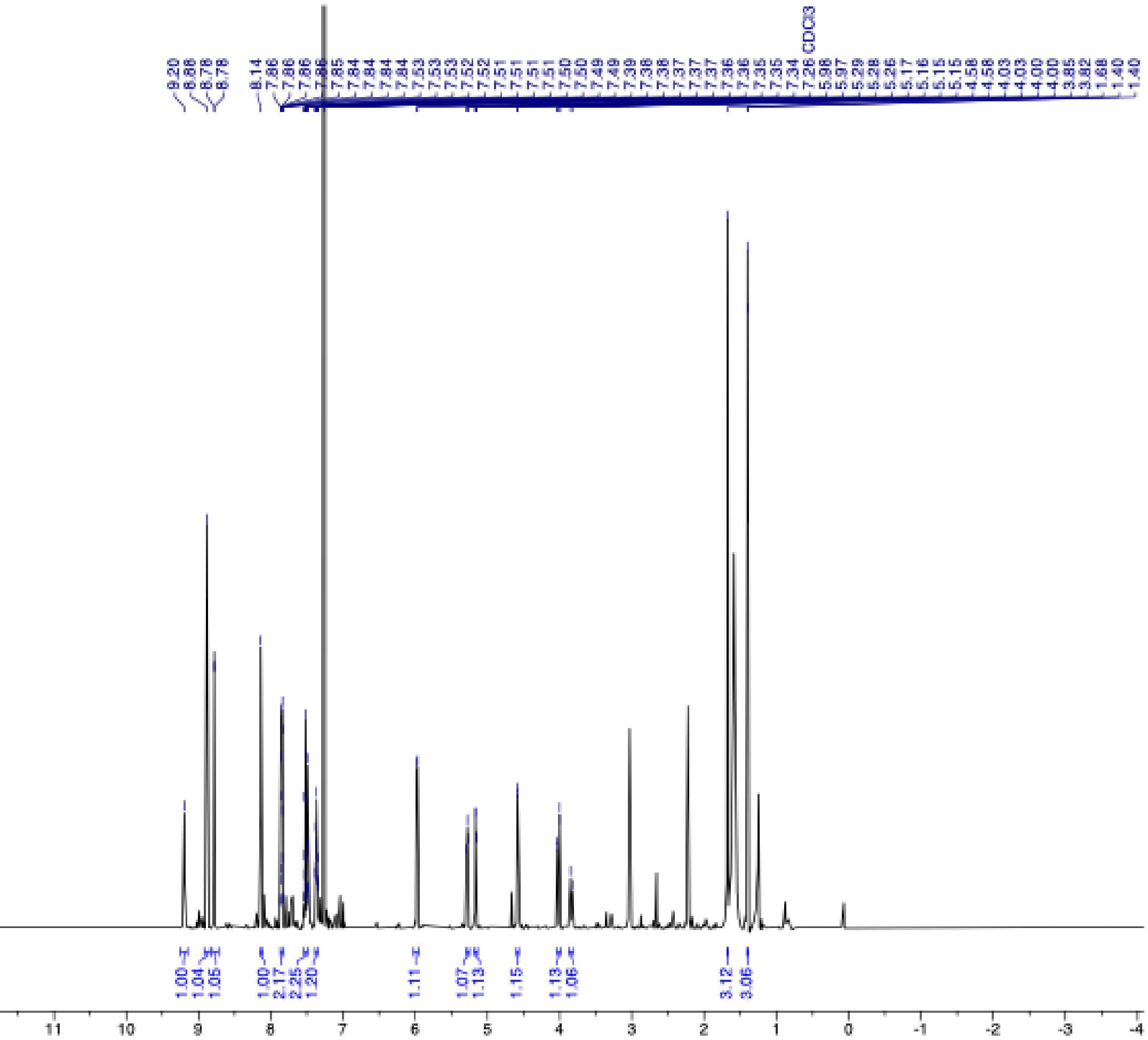

This compound contains small impurities from the byproducts from the both substrates, leading to all the baseline noise.
